# Total Synthesis and Structural Revision of Rhabdobranin Reveals a Cryptic Gram-Negative Antibiotic

**DOI:** 10.1021/jacs.6c09931

**Published:** 2026-08-27

**Authors:** Woonkee S. Jo, Zaynoun Attieh, Jan Johannes Crames, Carl Leonhard Knopp, Julie Connelly, Yi-Ming Shi, Christopher S. Wilson, Maryam Dehghan, Peter Grün, Sunna Chang, Fathima S. Refai, Fabio Piano, Kristin C. Gunsalus, Tanja Schneider, Helge B. Bode, Alan R. Healy

**Affiliations:** † Chemistry Program, 167632 New York University Abu Dhabi, Saadiyat Island, Abu Dhabi 129188, United Arab Emirates (UAE); ‡ Center for Genomics and Systems Biology, New York University Abu Dhabi, Saadiyat Island, Abu Dhabi 129188, UAE; § Department of Natural Products in Organismic Interactions, Max Planck Institute for Terrestrial Microbiology, Marburg 35043, Germany; ∥ Institute for Pharmaceutical Microbiology, 9374 University Hospital Bonn, University of Bonn, Bonn 53113, Germany; ⊥ State Key Laboratory of Quantitative Synthetic Biology, Center for Synthetic Biochemistry, Shenzhen Institute of Synthetic Biology, 85411 Shenzhen Institutes of Advanced Technology, Chinese Academy of Sciences, Shenzhen 518055, China; # Max Planck-Chinese Academy of Sciences Center for Synthetic Biochemistry, Shenzhen 518055, China; ¶ Biology Program, New York University Abu Dhabi, Saadiyat Island, Abu Dhabi 129188, UAE; ∇ Dipartimento di Medicina, Università di Udine, Udine 33100, Italy; ○ Center for Genomics and Systems Biology and Department of Biology, New York University, New York, New York 10003, United States; ⧫ DZIF, German Center for Infectious Research, Partner Site Bonn-Cologne, Bonn 53113, Germany; †† Center for Synthetic Microbiology (SYNMIKRO), Philipps University Marburg, Marburg 35043, Germany; ‡‡ Department of Chemistry, Philipps University Marburg, Marburg 35043, Germany; §§ Max Planck-Chinese Academy of Sciences Center for Synthetic Bichemistry, Marburg 35043, Germany

## Abstract

Gram-negative bacteria present a major clinical challenge but also remain an underexplored source of antibacterial natural products. Resistance-guided genome mining of the entomopathogenic symbiont *Xenorhabdus* identified the *rdb* biosynthetic gene cluster, which encodes a putative prodrug antibiotic, pre-rhabdobranin. However, the inability to isolate the proposed active metabolite, rhabdobranin, has prevented direct functional evaluation. Here we report a convergent total synthesis of the proposed structure of pre-rhabdobranin B, which revealed a stereochemical misassignment at the *N*-terminal arginine residue. Synthesis of both rhabdobranin epimers showed that, although they are nearly indistinguishable by standard analytical methods, inversion at this single stereocenter has a pronounced effect on antibacterial activity. Biological evaluation of the revised rhabdobranin structure revealed potent antibacterial activity against Gram-negative pathogens, including WHO critical-priority carbapenem-resistant *Klebsiella pneumoniae*. Cellular and biochemical profiling implicated inhibition of protein biosynthesis as its principal antibacterial mechanism. We further show that the GNAT-family acetyltransferase RdbK *N*-acetylates rhabdobranin, attenuating its activity and establishing a secondary self-resistance mechanism. These findings validate resistance-gene-guided discovery in Gram-negative symbionts as a strategy for uncovering cryptic antibiotics and identify rhabdobranin as a promising scaffold for Gram-negative antibiotic development.

## Graphical abstract

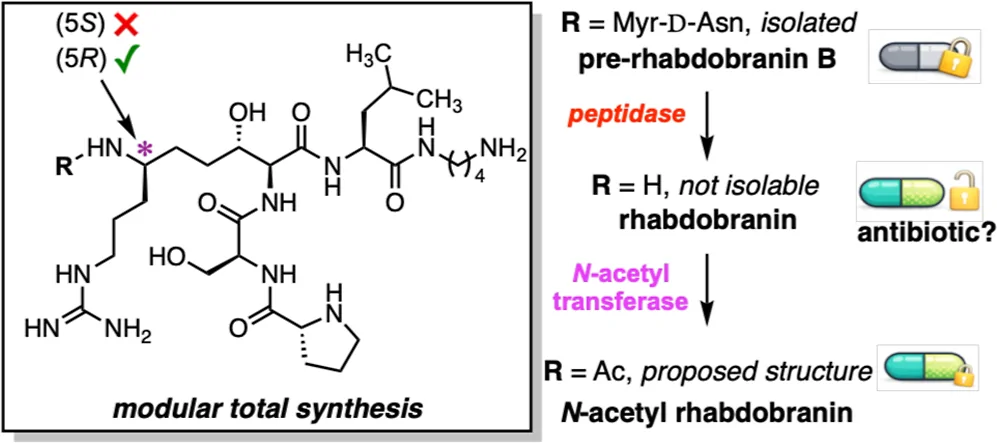

## Introduction

Antimicrobial resistance represents a critical and escalating threat to global health.^1,2^ The most recent global burden analysis estimated that bacterial antimicrobial resistance was associated with 4.71 million deaths in 2021, including 1.14 million deaths directly attributable to resistant infections.^3^ Gram-negative pathogens are principal drivers of this burden, with carbapenem-resistant *Klebsiella pneumoniae* and *Acinetobacter baumannii* designated as critical-priority pathogens by the World Health Organization (WHO).^4^ Their intrinsic resistance, arising from restrictive outer membranes, active efflux, and the rapid dissemination of resistance determinants, has severely limited the effectiveness of existing antibiotics.^5^ Consequently, the discovery of antibacterial agents capable of overcoming Gram-negative permeability and efflux barriers remains a major unmet need.

Paradoxically, Gram-negative bacteria themselves represent an underexplored yet highly promising source of novel antibacterial agents.^6–10^ Historically, most clinically relevant natural product antibiotics have been derived from soil-dwelling actinomycetes, particularly members of the *Streptomyces* genus.^11,12^ This reliance on a narrow taxonomic space has led to high rates of rediscovery, limiting access to fundamentally new chemical scaffolds.^13^

Increasing attention has therefore shifted toward underexplored microbial taxa and ecological niches as sources of new antimicrobial natural products.^14^ Among these entomopathogenic symbionts, particularly Gram-negative bacteria from the genera *Xenorhabdus* and *Photorhabdus*, have emerged as prolific sources of structurally diverse bioactive metabolites.^15,16^ These bacteria reside within infective juvenile nematodes that parasitize soil-dwelling insect larvae. Upon entry into the insect hemolymph, the bacteria are released and rapidly deploy virulence factors that kill the insect, together with a diverse arsenal of antimicrobial metabolites that protect the resulting nutrient-rich cadaver from competing microorganisms (Figure 1A). This intense ecological pressure has driven the evolution of potent antibacterial compounds, including xenocoumacin,^17,18^ amicoumacin,^19^ odilorhabdin,^20^ darobactin,^21^ and dynobactin^22^ (Figure 1B). Genome-wide analyses of *Xenorhabdus* and *Photorhabdus* have revealed extensive biosynthetic capacity, with hundreds of biosynthetic gene clusters (BGCs) encoding diverse natural product families.^15,23^ However, only a small fraction of these BGCs have been experimentally linked to their corresponding metabolites.^24^ Thus, the central challenge is not a lack of biosynthetic diversity,^25,26^ but how to prioritize BGCs for functional investigation. In this context, resistance-gene-guided discovery provides a powerful strategy to identify functionally relevant compounds.^27–30^ Antibiotic-producing organisms frequently employ self-resistance mechanisms that protect the organism from autotoxicity. Consequently, BGCs containing resistance determinants often provide strong genetic signatures for intrinsically bioactive metabolites. This logic is particularly relevant in Gram-negative systems, as these features may identify compounds with activity against Gram-negative pathogens.

**Figure 1:**
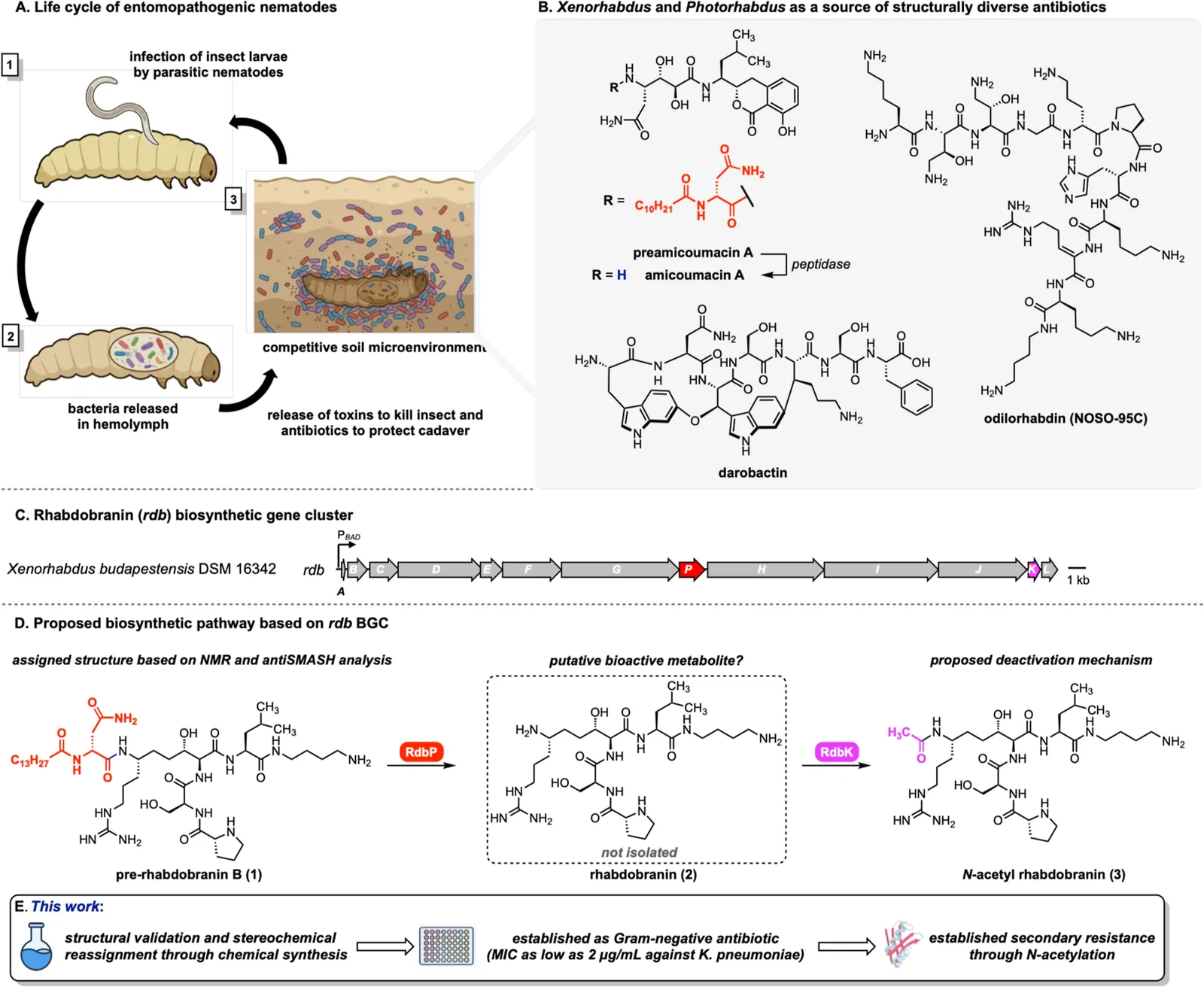
(A) Entomopathogenic nematodes (*Steinernema*, *Heterorhabditis*) infect insect larvae [1] and release their symbiotic bacteria (*Xenorhabdus*, *Photorhabdus*) into the hemolymph [2]. The bacteria proliferate inside the host and secrete toxins that kill the insect, while also producing antibiotics that suppress competing microorganisms and protect the cadaver as a nutrient-rich niche [3]. (B) Representative Gram-negative active metabolites previously isolated from *Xenorhabdus* and *Photorhabdus*. (C) The rdb biosynthetic gene cluster from *Xenorhabdus* *budapestensis* DSM 16342 contains nonribosomal peptid synthetase (NRPS) and polyketide synthase (PKS) enzymes alongside a dedicated peptidase and *N*-acetyltransferase. (D) Pre-rhabdobranin (**1**) was isolated from a Δ*rdbP* mutant strain. It is hypothesized that the RdbP peptidase cleaves the prodrug motif to form the active metabolite **2**, while RdbK serves as a secondary resistance element. (E) Structural revision and biological evaluation of rhabdobranin through total synthesis.

Application of this logic to uncharacterized BGCs from *Xenorhabdus* identified the *rdb* cluster,^23^ which encodes a putative hybrid polyketide–nonribosomal peptide (PK–NRP), termed rhabdobranin, together with a dedicated peptidase (RdbP) and an *N*-acetyltransferase (RdbK) (Figures 1C and S1). The presence of the peptidase strongly suggests a prodrug activation mechanism, analogous to those observed in the xenocoumacin,^17^ amicoumacin,^19^ zwittermicin,^31^ and colibactin^32^ pathways.^33,34^ In these systems, the inactive *N*-acyl-d-asparagine-containing prodrug is synthesized in the cytoplasm, transported across the inner membrane and cleaved by a peptidase to release the active compound in the periplasm immediately prior to export, thereby minimizing intracellular toxicity. The colocalized *N*-acetyltransferase suggests a complementary secondary resistance mechanism (Figure 1D). In related systems, including amicoumacin^19^ and odilorhabdin,^35^ homologous enzymes protect the producing organism by deactivating the antibiotic through *N*-terminal acetylation following re-entry of the active compound. The coexistence of prodrug activation and secondary resistance genes therefore strongly indicates that the encoded metabolite is a cryptic antibiotic.

Because mature rhabdobranin could not be isolated owing to its low production under laboratory conditions, efforts focused on the more stable prodrug precursors. Inactivation of the peptidase followed by activation of the BGC enabled the isolation of pre-rhabdobranins A–D, which feature the characteristic *N*-acyl-d-asparagine motif and an unusual branched-peptide architecture but differ in the identity of their *N*-terminal fatty-acid tails (Figure 1D).^23^

Given the inability to access the mature metabolite directly, chemical synthesis offered a direct route to validate the proposed structure, access rhabdobranin, and interrogate its biological function.^36^ Here we report a convergent synthesis of the proposed structure of pre-rhabdobranin B, which revealed a stereochemical misassignment. Synthesis of the revised mature rhabdobranin metabolite enabled biological evaluation and demonstrated potent activity against Gram-negative pathogens, including WHO critical-priority carbapenem-resistant *K. pneumoniae* (Figure 1E).

## Results

### Retrosynthetic Analysis

Our retrosynthetic analysis is outlined in Scheme 1. We identified orthogonally protected rhabdobranin **4** as a pivotal intermediate, enabling late-stage access to the target rhabdobranin (**2**) through global deprotection. Alternatively, selective removal of the Fmoc protecting group followed by functionalization of the *N*-terminus and subsequent global deprotection would provide pre-rhabdobranin (**1**) and the putative *N*-acetylated rhabdobranin (**3**) (Figure 1D).

**Scheme 1:**
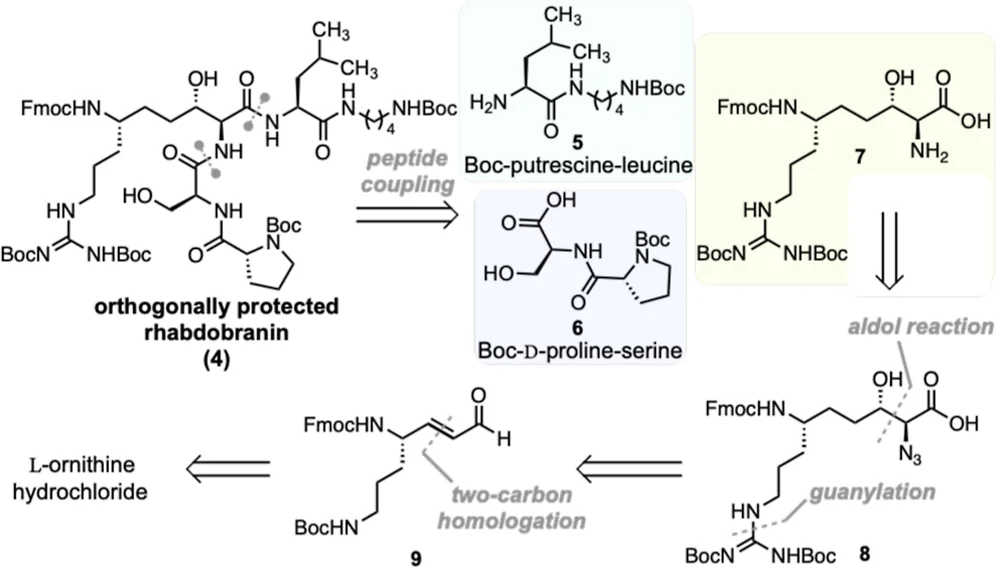
Retrosynthetic Analysis of the Orthogonally Protected Rhabdobranin Backbone

Intermediate **4** was further disconnected into three fragments of comparable complexity: dipeptides **5** and **6**, and amino alcohol **7**. The azide **8** was strategically employed as a latent amine, orthogonal to standard peptide protecting groups, and could be traced retrosynthetically using an aldol disconnection to aldehyde **9**. Installation of the guanidine functionality was deferred to a late stage due to purification challenges associated with arginine-containing intermediates.^37,38^ The unsaturated aldehyde precursor was selected to avoid instability associated with saturated 1,5-amino aldehydes, which readily undergo intramolecular cyclization.^39^ Aldehyde **9** could in turn be accessed from a two-carbon homologation of an l-ornithine-derived precursor.

### Synthesis of the Proposed Pre-rhabdobranin B

The synthesis began with the preparation of allylic aldehyde **9**, the substrate for the key aldol reaction. Initial attempts to access unsaturated ester **12** by partial reduction of *N*-Fmoc ornithine ester **10** followed by olefination were unsuccessful due to rapid intramolecular 1,5-cyclization of the ornithine side-chain amine with the intermediate aldehyde **11**. To circumvent this issue, we employed a one-pot reductive olefination strategy.^40–42^ Partial reduction of the ester **10**, followed by *in situ* trapping with the anion of phosphonoacetate **A**, directly furnished the corresponding unsaturated ester **12** in 60% yield, thereby avoiding isolation of the labile aldehyde intermediate. Subsequent two-step redox manipulation provided allylic aldehyde **9** (Scheme 2).

**Scheme 2:**
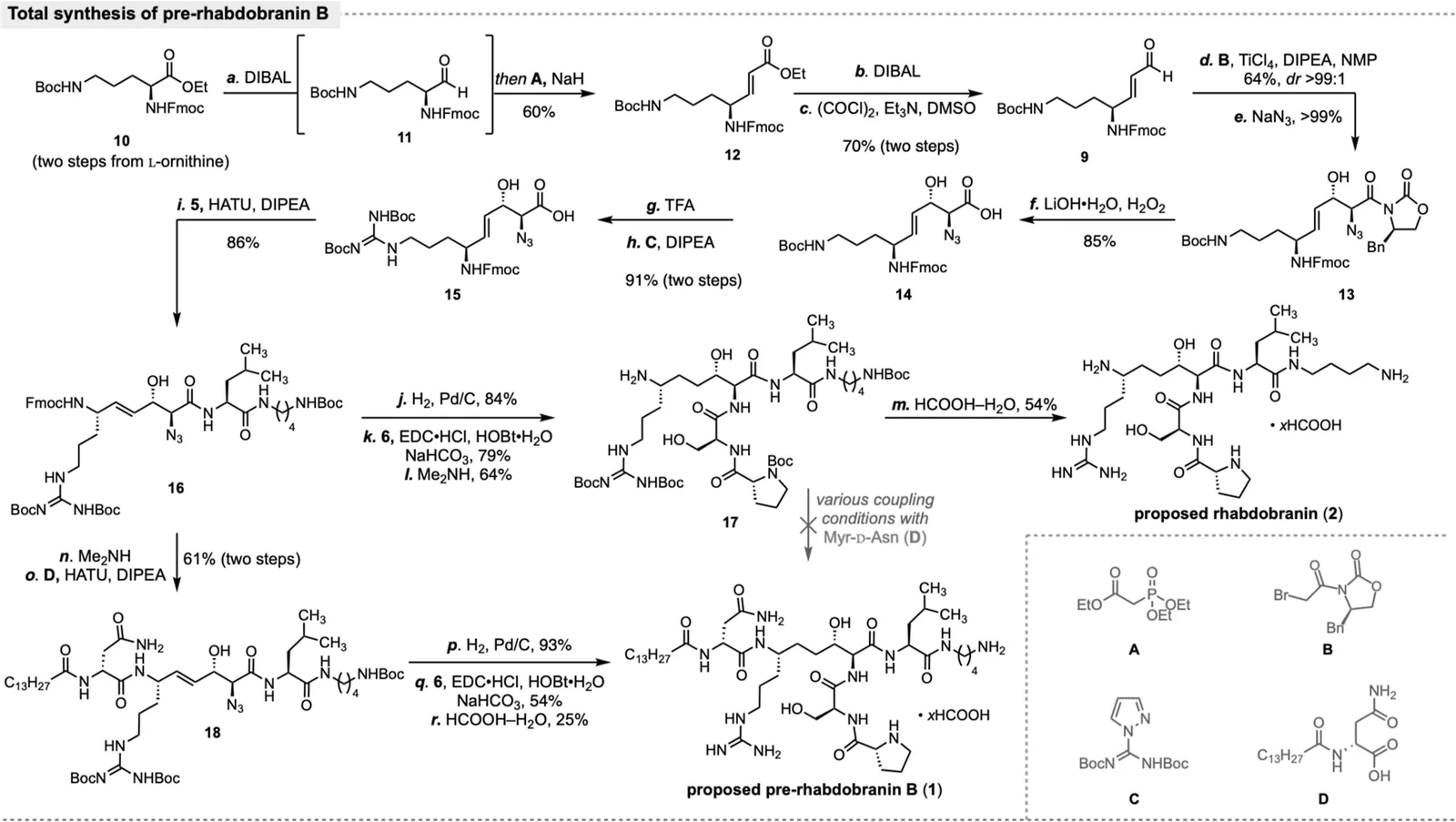
Synthesis of Proposed Pre-rhabdobranin B and Rhabdobranin^a^

a Reagents and conditions: (**a**) DIBAL (2.2 equiv), Et_2_O, −78 °C, 30 min, then A (1.2 equiv), NaH (1.1 equiv), THF, −78 to 23 °C, 6 h, 60% (b) DIBAL (4.0 equiv), THF, −78 °C, 4 h (c) (COCl)_2_ (1.1 equiv), Et_3_N (5.0 equiv), DMSO (2.2 equiv), CH_2_Cl_2_, −78 to 23 °C, 1 h, 70% over two steps (d) B, TiCl_4_ (1.15 equiv), DIPEA (1.2 equiv), NMP (1.1 equiv), CH_2_Cl_2_, 0 °C, 2 h, 64%, *dr* > 99:1 (e) NaN_3_ (2.0 equiv), DMSO, 23 °C, 2 h, quantitative (f) LiOH•H_2_O (2.0 equiv), H_2_O_2_ (6.0 equiv), THF–H_2_O, 0 °C, 1 h, 85% (g) 10% TFA in CH_2_Cl_2_, 23 °C, 2 h (h) *N*,*N*′-di-Boc-1*H*-pyrazole-1-carboxamidine C (1.2 equiv), DIPEA (5.0 equiv), CH_3_CN, 23 °C, 18 h, 91% over two steps (i) **5** (1.3 equiv), HATU (1.4 equiv), DIPEA (3.5 equiv), THF, 0 to 23 °C, 18 h, 86% (j) H_2_, Pd/C, CH_3_OH–THF, 3 h, 84% (k) **6** (1.3 equiv), EDC•HCl (1.2 equiv), HOBt•H_2_O (1.7 equiv), NaHCO_3_ (1.2 equiv), CH_2_Cl_2_, 0 to 23 °C, 16 h, 79% (l) dimethylamine (20 equiv), THF, 23 °C, 5 h, 64% (m) 85% aqueous formic acid, 23 °C, 24 h, 54% (n) dimethylamine (20 equiv), THF, 23 °C, 5 h (o) D (3.0 equiv), HATU (2.6 equiv), DIPEA (3.0 equiv), DMF, 23 °C, 2 h, 61% over two steps (p) H_2_, Pd/C, CH_3_OH–THF, 3 h, 93% (q) **6** (1.3 equiv), EDC•HCl (1.2 equiv), HOBt•H_2_O (1.7 equiv), NaHCO_3_ (1.2 equiv), CH_2_Cl_2_, 0 to 23 °C, 16 h, 54% (r) 85% aqueous formic acid, 23 °C, 24 h, 25%.

Because general *anti*-selective α-azido or α-amino aldol methods remain limited, we adopted a *syn*-selective α-halo aldol reaction followed by stereoinvertive azide displacement. Accordingly, allylic aldehyde **9** was subjected to a titanium-mediated Crimmins halo–aldol reaction with auxiliary **B**,^43^ affording the bromohydrin as a single diastereomer in 64% yield. Treatment with sodium azide in dimethyl sulfoxide effected clean displacement to furnish the *anti-*azido product **13** with no erosion of stereochemical integrity. Auxiliary cleavage using lithium peroxide provided the corresponding α-azidocarboxylic acid **14** in 85% yield. Finally, trifluoroacetic acid-mediated Boc deprotection, followed by guanylation with *N*,*N*′-di-Boc-1*H*-pyrazole-1-carboxamidine (**C**),^44^ delivered intermediate **15** in 91% yield. Fragment coupling with dipeptide **5**, derived from *N*-Boc-putrescine and leucine (see Supporting Information), furnished fragment **16**. Hydrogenation of both the azide and olefin then delivered the protected saturated amino alcohol in a single operation.

Subsequent EDC-mediated coupling with dipeptide **6**, *N*-Boc-d-proline–serine (see Supporting Information), and dimethylamine-mediated Fmoc deprotection gave the fourfold Boc-protected rhabdobranin **17**, setting the stage for the final coupling with *N*-myristoyl-d-asparagine (**D**) to complete the synthesis of pre-rhabdobranin B (**1**).

This transformation, however, proved recalcitrant, affording only trace product under a range of established coupling conditions. We attribute this diminished reactivity, at least in part, to the steric congestion and conformational flexibility of the amine **17**. To address this limitation, we pursued an alternative strategy involving an earlier incorporation of the *N*-Myr-d-Asn motif. In this revised sequence, fragment **16**, a less congested and more rigid substrate, was identified as a viable coupling partner. Accordingly, Fmoc removal followed by HATU-mediated coupling with *N*-Myr-d-Asn (**D**) proceeded smoothly to furnish the corresponding amide **18**.^45^ Subsequent concomitant reduction of the azide and olefin, followed by installation of dipeptide **6**, delivered the fully protected pre-rhabdobranin B. As natural pre-rhabdobranin B was isolated as a formic acid salt following HPLC purification, the final Boc deprotection was carried out using 85% aqueous formic acid to provide the proposed pre-rhabdobranin B (**1**).

### Stereochemical Reassignment of (pre)­rhabdobranin

Comparison of the analytical data (^1^H and ^13^C NMR) for natural and synthetic pre-rhabdobranin B revealed largely congruent spectra; however, small but distinct discrepancies were observed at the arginine-derived *N*-acyl carbon (C–5) and the amino alcohol stereocenters (C–8 and C–9) (Figure 2A and Table S2). These deviations suggested a potential stereochemical misassignment. To investigate this possibility, HPLC–ESI–MS coinjection studies were performed. The observation of two peaks with distinct retention times indicated that the natural and synthetic samples were diastereomeric (Figure 2A).

**Figure 2:**
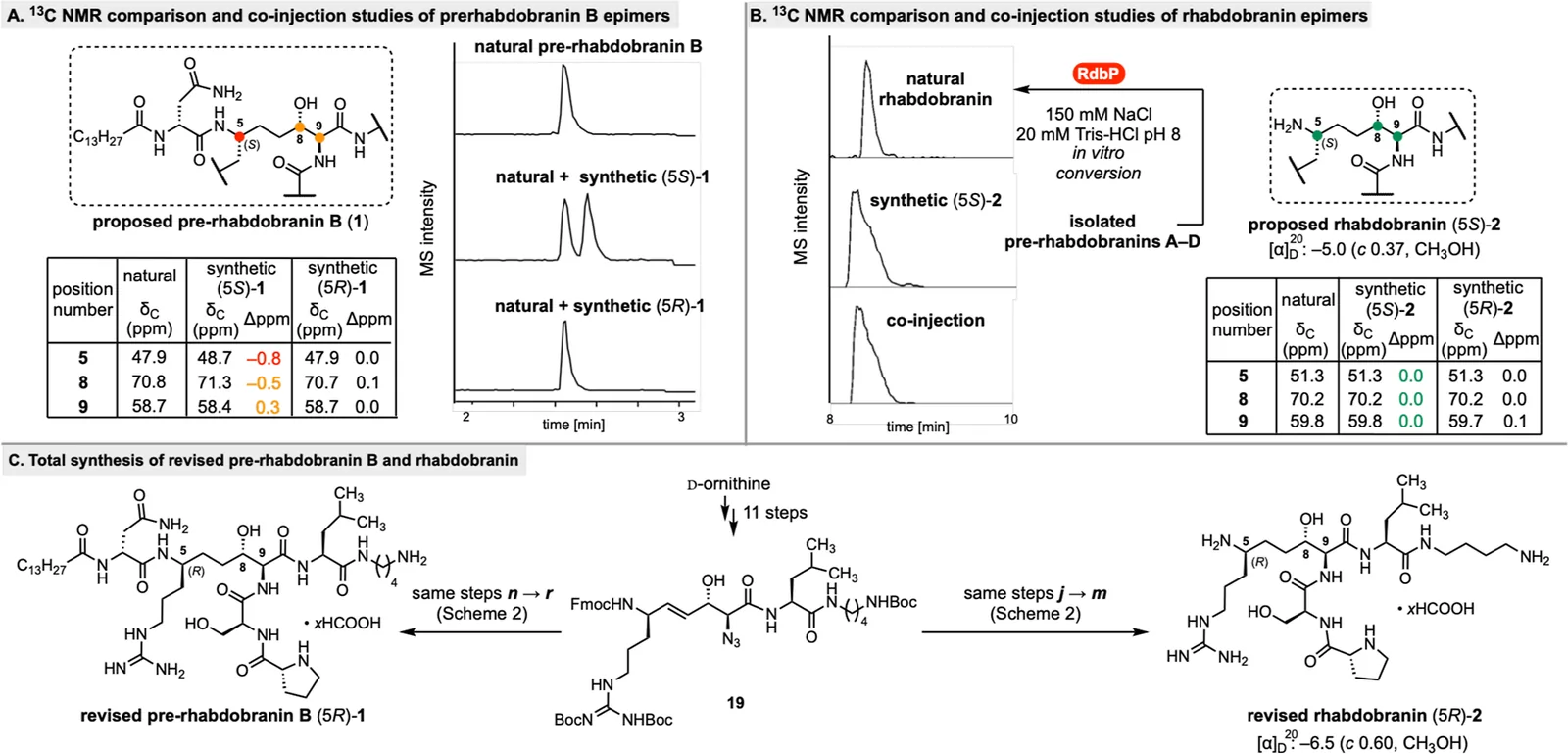
(A) ^13^C NMR shift comparison and coinjection studies of natural pre-rhabdobranin B with both synthetic epimers. (B) In vitro conversion of pre-rhabdobranins A–D to rhabdobranin and subsequent coinjection studies with the originally proposed (5*S*)-**2** and ^13^C NMR shift comparison of natural rhabdobranin (generated from pre-rhabdobranins A–D) with both synthetic epimers. (C) Forward synthesis of revised pre-rhabdobranin B (5*R*)-**1** and rhabdobranin (5*R*)-**2**.

While these data were consistent with misassignment at either the arginine-derived stereocenter or the amino alcohol motif, further evidence was required to distinguish between these possibilities. We reasoned that analysis of rhabdobranin, which possesses a simpler truncated backbone, would provide a more diagnostically informative comparison. Because direct isolation of natural rhabdobranin was unsuccessful from *Xenorhabdus budapestensis* WT due to very low production relative to the production of pre-rhabdobranins in the Δ*rdbP* mutant, we developed an indirect approach involving preparative-scale enzymatic conversion of pre-rhabdobranins A–D using *Escherichia coli* expressing the RdbP peptidase (Figure S2). This approach provided 15 mg of RdbP-generated rhabdobranin, enabling direct comparison with the synthetic material (Figure 2B).

In contrast to pre-rhabdobranin, HPLC analysis of the natural rhabdobranin and originally proposed synthetic sample showed a single peak on coinjection. Likewise, the NMR spectra of natural and synthetic rhabdobranin were also in complete agreement, with no discernible differences at either the amino alcohol or arginine stereocenters (Figure 2B and Table S3). The NMR analysis strongly argued against misassignment of the amino alcohol configuration, as vicinal amino alcohol diastereomers typically exhibit clear and diagnostic spectroscopic differences.^46,47^ Instead, the data pointed to misassignment at the arginine-derived stereocenter as the origin of the discrepancy.

On this basis, we considered revision of the assigned L-configuration to the corresponding D-configuration. Precedent for such a reassignment was reported by Süssmuth and co-workers in the structural revision of prepaenilamicin B2, which features a related prodrug motif.^48^ To test this hypothesis, pre-rhabdobranin B was resynthesized using d-ornithine (Figure 2C). The resulting (5*R*) epimer exhibited complete spectroscopic agreement with the natural product (^1^H and ^13^C NMR). Consistent with this assignment, HPLC–ESI–MS coinjection of the natural sample with the synthetic epimer gave a single peak (Figure 2A and Table S4).

Using the same modular strategy, we also accessed the (5*R*)-rhabdobranin (**2**) (Figure 2C). Strikingly, the two rhabdobranin epimers were virtually indistinguishable across several analytical methods examined, including ^1^H and ^13^C NMR, HPLC–ESI–MS coinjection, and optical rotation (Figure 2B and Table S5). Consequently, synthesis of rhabdobranin alone would likely not have prompted the stereochemical reassignment, highlighting a potentially underappreciated challenge in the structural elucidation of conformationally flexible natural products, where subtle stereochemical differences may elude conventional spectroscopic analysis.^49–52^

Although a similar L-to-D reassignment was reported for prepaenilamicin, the biosynthetic basis for such epimerization remains unclear. Nonproteinogenic amino acids, including d-amino acids, are common features of nonribosomal peptides and hybrid natural products, and typically arise through incorporation of the corresponding l-amino acid followed by epimerization mediated by a dedicated epimerization domain.^53,54^ Importantly, neither the prepaenilamicin BGC nor the pre-rhabdobranin BGC encodes such a domain. To probe the origin of the d-arginine residue, we investigated whether d-arginine could be directly incorporated by the biosynthetic assembly line. RdbD and RdbF, the modules responsible for incorporation of the C14-d-Asn-Arg fragment, were fused to the terminal two modules of the ambactin-producing NRPS by NRPS engineering. Comparison of the resulting product, C14-d-Asn-Arg-d-Trp-l-Lys, with synthetic arginine epimers confirmed incorporation of l-Arg rather than d-Arg (Figure S3). These results suggest that epimerization occurs after substrate loading and during PKS-catalyzed elongation, potentially through a dynamic kinetic resolution-type process.^55^

Following stereochemical reassignment of rhabdobranin, we prepared two additional synthetic analogues, *N*-acetyl rhabdobranin (5*R*)-**3** and linear analogue **20**, from late-stage intermediates in five and three steps, respectively (Figure 3). These derivatives were designed to probe the proposed secondary resistance mechanism and to provide an initial assessment of the functional importance of the unusual branched side chain.

**Figure 3:**
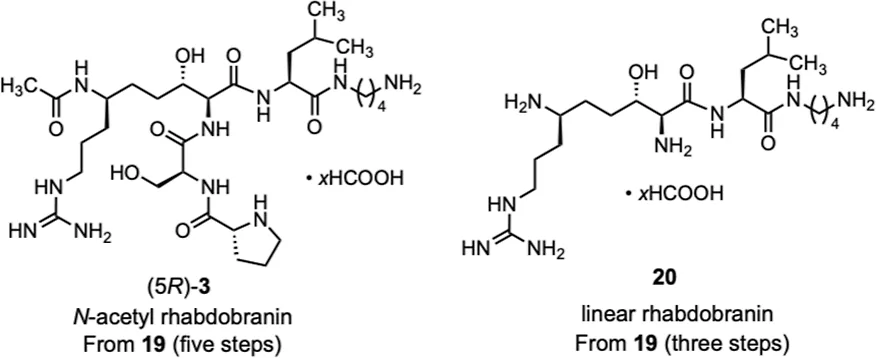
Structures of rhabdobranin derivatives.

### A Secondary Resistance Mechanism

The *rdb* cluster also encodes RdbK, a predicted acetyl-CoA-dependent GNAT-family *N*-acetyltransferase. Because *N*-acetylation frequently operates as part of a dual resistance strategy in prodrug-activated antibiotic systems such as amicoumacin,^19^ zwittermicin A,^56^ paenilamicin,^57^ and edeine,^58^ we hypothesized that RdbK provides a secondary self-resistance mechanism by acetylating rhabdobranin after re-entry into the producing cell.

To test this hypothesis, RdbK from *X. budapestensis* DSM 16342 was heterologously expressed and purified by metal affinity chromatography. In vitro assays revealed efficient acetylation of rhabdobranin in the presence of RdbK and acetyl-CoA, as confirmed by HPLC–HRMS analysis (Figure S4). To assess the functional relevance of this modification in a cellular context, growth assays were performed using *E. coli* BL21­(DE3) expressing *rdbK* or an empty vector control. Both strains grew normally in the absence of rhabdobranin. In contrast, the empty-vector control strain lacking *rdbK* showed complete growth inhibition in the presence of rhabdobranin, whereas the strain expressing *rdbK* reached the same optical density as the control but showed a prolonged lag phase of approximately 6 h (Figure S4). These results support a model in which RdbK-mediated *N*-acetylation attenuates rhabdobranin activity and restores bacterial growth, providing functional evidence for a secondary self-resistance mechanism encoded within the *rdb* locus.

### Bioactivity Analysis

The co-occurrence of two orthogonal self-resistance elements within the *rdb* locus strongly indicated that rhabdobranin is a functionally relevant antimicrobial agent. To evaluate this hypothesis, rhabdobranin was screened against a panel of clinically relevant ESKAPEE pathogens^59^ based on the WHO Bacterial Priority Pathogens List (WHO BPPL).^4^ The panel also included drug-susceptible reference strain *E. coli* ATCC 25922,^60^ and *Serratia marcescens* ATCC 13880, an opportunistic Gram-negative pathogen increasingly implicated in healthcare-associated bloodstream infections and neonatal sepsis.^61^ The screen also included revised rhabdobranin (5*R*)-**2** and its epimer (5*S*)-**2**, *N*-acetyl rhabdobranin (5*R*)-**3**, and linear analogue **20** (Figure 4A). In the primary screen at 128 μg mL^–1^, rhabdobranin (5*R*)-**2** produced near-complete growth inhibition of *Staphylococcus aureus*, *K. pneumoniae*, *E. coli*, *S. marcescens*, and *P. aeruginosa* DSM 24600. In contrast, activity against the two *A. baumannii* isolates and *E. cloacae* was only partial, and *P. aeruginosa* NCTC 13437 remained largely refractory (Figure 4A). Interestingly, rhabdobranin exhibited a conditional phenotype against *Enterococcus faecium*. Under the standard screening condition (BHI, pH 7.3), *E. faecium* NCTC 12204 showed no measurable response to rhabdobranin (Figure 4A). Because the activity of cationic peptide-derived antibiotics is often pH dependent,^62–64^ we assessed the effect of rhabdobranin on *E. faecium* NCTC 12204 in BHI adjusted to pH 9.0. Under these conditions, rhabdobranin completely inhibited bacterial growth. This pH-dependent phenotype was also observed in two additional WHO-relevant resistant *E. faecium* isolates, DSM 13590 (*vanA*-positive VRE) and DSM 25697 (multidrug-resistant) (Figure S5). Importantly, rhabdobranin showed limited mammalian cytotoxicity under the conditions tested: HeLa cell viability was unaffected at 100 μM (63 μg mL^–1^), while HEK293T viability remained above 50% at the same concentration (Figure 4A). Consistent with these findings, concentration–response profiling yielded half-maximal cytotoxic concentration (CC_50_) values above 100 μM in both cell lines (Figure S6).

**Figure 4:**
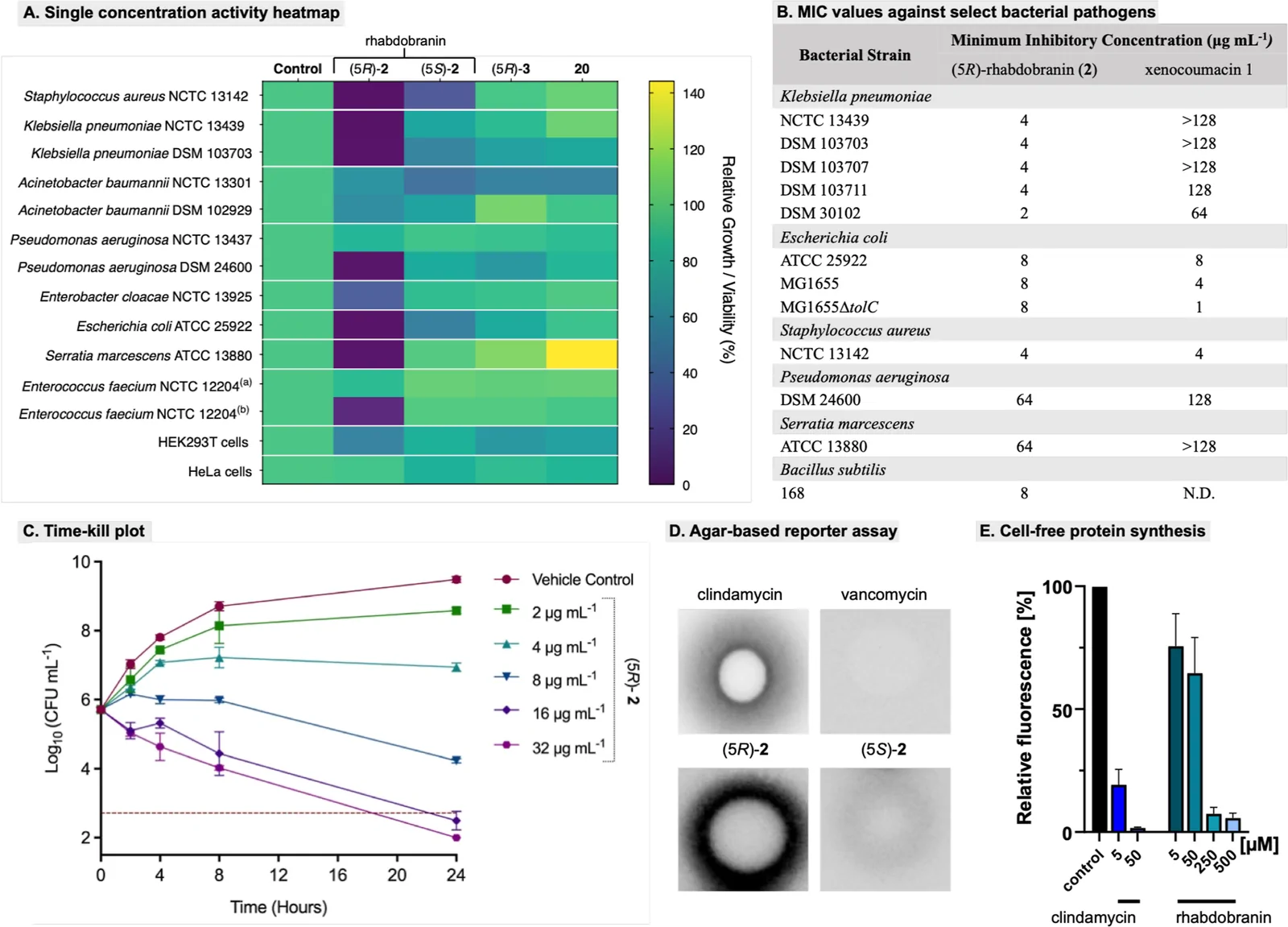
(A) Activity heatmap of rhabdobranin diastereomers alongside analogues (5*R*)-**3** and **20** against select ESKAPEE strains at a single concentration (128 μg mL^–1^). Viability of HeLa and HEK293T cells was assessed in parallel at 100 μM (63 μg mL^–1^). Values are expressed relative to vehicle-treated controls. Relative growth of *Enterococcus faecium* NCTC 12204 in BHI was assessed at both pH = 7.3^(a)^ and pH = 9^(b)^. The complete bacterial strain panel, including documented resistance determinants, strain source, and role in the present study, is summarized in Table S1. (B) Minimum inhibitory concentrations of rhabdobranin (5*R*)-**2** and xenocoumacin 1 against clinically relevant Gram-negative pathogens. Data are representative of three biological replicates. (**C**is summarized in Table S1. (B) Minimum inhibitory concentrations of rhabdobranin (5R)-2 and xenocoumacin 1 against clinically relevant Gram-negative pathogens. Data are representative of three biological replicates. (C) Time-kill kinetics of rhabdobranin (5*R*)-**2** against carbapenem-resistant *Klebsiella pneumoniae* DSM 103707. Bacterial cultures were treated with vehicle control or rhabdobranin (5*R*)-**2** at 2, 4, 8, 16, and 32 μg mL^–1^ (0.5–8 × MIC), and viable counts were determined by CFU enumeration at 0, 2, 4, 8, and 24 h. Data are plotted as mean ± SD of three biological replicates. The red dashed line indicates the bactericidal threshold, defined as a ≥ 3-log_10_ reduction in CFU mL^–1^ relative to the starting inoculum. Values below the limit of detection are plotted at 100 CFU mL^–1^, corresponding to 2.0 log_10_ (CFU mL^–1^). (D) Agar-based screening of rhabdobranin (5*R*)-**2** and derivatives using the *Bacillus subtilis* P_yheI_-lux reporter for protein-biosynthesis stress. Among the tested compounds, rhabdobranin (15 μg) specifically induced the reporter strain. Clindamycin (1.5 μg) and vancomycin served as positive and negative controls, respectively. Images are representative of three independent experiments. (E) Cell-free mNeonGreen synthesis was assessed using the coupled PURExpress in vitro transcription–translation system in the presence of clindamycin (5 and 50 μM) or rhabdobranin (5–500 μM). After incubation at 37 °C for 2 h, fluorescence was measured at 485/535 nm and normalized to the DMSO vehicle control (100%). Data are presented as mean ± SD. N.D. = not determined.

Evaluation of the additional rhabdobranin derivatives demonstrated that subtle structural modifications result in pronounced losses in activity. The originally proposed (5*S*)-**2** displayed only moderate and strain-limited growth inhibition, revealing a sharp activity cliff associated with inversion of the *N*-terminal stereocenter. Likewise, *N*-terminal acetylation effectively suppressed activity, supporting RdbK-mediated acetylation as a secondary resistance mechanism in the producing organism and underscoring the importance of the free *N*-terminal amine for target engagement. Removal of the branched dipeptide similarly resulted in a marked reduction in activity, establishing this structural element as essential for antibacterial function.

To further define the antibacterial profile of rhabdobranin (5*R*)-**2**, a secondary screen was conducted to determine minimum inhibitory concentrations (MICs) against the most responsive strains from the primary screen (Figure 4B). The most prominent signal from the primary screen was the potent activity against carbapenem-resistant *K. pneumoniae*, a WHO critical-priority pathogen ranked as the highest global priority for antibiotic development.^4^ Given the clinical weight of this organism we expanded the *Klebsiella* set to include two additional WHO-relevant carbapenem-resistant clinical isolates, and DSM 30102, a nonpriority within-species comparator strain. Across the four resistant isolates, MIC values were uniformly 4 μg mL^–1^, while the comparator strain exhibited an improved MIC of 2 μg mL^–1^ (Figure 4B). Time-kill analysis against carbapenem-resistant *K. pneumoniae* DSM 103707 revealed concentration-dependent activity of rhabdobranin (5*R*)**-2**. At the MIC (4 μg mL^–1^), rhabdobranin suppressed growth without reducing the initial inoculum, whereas 4 and 8 × MIC produced a ≥3-log_10_ reduction in viable counts after 24 h. Together with a minimum bactericidal concentration (MBC) of 16 μg mL^–1^ (MBC/MIC = 4), these findings establish concentration-dependent bactericidal activity against this strain.^65,66^

Xenocoumacin 1 was evaluated in parallel as a lineage-relevant comparator, as it is a well-characterized *Xenorhabdus* antibiotic and, like rhabdobranin, is released from an *N*-acyl-d-asparagine-capped precursor by a dedicated peptidase (Figure 4B). Rhabdobranin and xenocoumacin 1 displayed comparable activity against *S. marcescens*, *P. aeruginosa* DSM 24600, *S. aureus* NCTC 13142, and *E. coli* ATCC 25922, with MIC values of 4 μg mL^–1^ and 8 μg mL^–1^ observed against the latter two strains, respectively. We next assessed susceptibility to TolC-dependent efflux, a major determinant of Gram-negative intrinsic resistance and whole-cell antibiotic potency. Rhabdobranin exhibited identical MIC values (8 μg mL^–1^) in wild-type *E. coli* MG1655 and the efflux-deficient MG1655 Δ*tolC* strain, whereas xenocoumacin displayed a 4-fold improvement in potency in the Δ*tolC* background (4 to 1 μg mL^–1^). These findings suggest that, unlike xenocoumacin, rhabdobranin is not strongly impacted by TolC-mediated export. The most striking distinction between the two *Xenorhabdus*-derived antibiotics, however, was their activity against *Klebsiella* strains, where xenocoumacin was effectively inactive. Together, these differences raise the possibility that *Xenorhabdus* deploys chemically distinct prodrug antibiotics with complementary antibacterial activities to shape and defend its complex ecological niche in the soil.^17^

### Rhabdobranin Inhibits Protein Biosynthesis

To investigate the mode of action, rhabdobranin (5*R*)-**2** and derivatives were profiled using *Bacillus subtilis* bioreporters responsive to interference with major cellular biosynthesis pathways (cell wall, DNA, RNA and protein synthesis). In an agar-based promoter-*lacZ* screen, rhabdobranin (5*R*)-**2** selectively activated the protein biosynthesis stress reporter P_*yheI*_-*lacZ*, whereas induction of the other pathway-specific reporters was lacking (Figure S7). This response was confirmed using a P_*yheI*_-*lux* reporter, which showed pronounced induction within 18 h, compared to the positive control clindamycin (Figure 4D). Consistent with their loss of antibacterial activity, the linear (**20**) and acetylated (**3**) derivatives produced markedly reduced or no growth inhibition zones. The originally proposed (5*S*)-**2** retained weak protein stress reporter induction but exhibited substantially reduced growth inhibition (Figure S7).

Time-resolved measurements using the corresponding luciferase (*lux*) reporters in liquid media further supported a selective effect on protein biosynthesis (Figure S8). Rhabdobranin (5*R*)-**2** strongly induced P_*yheI*_-*lux* without relevant activation of the cell wall, DNA or RNA stress reporters. The kinetic profile of the luciferase reporter response differed between rhabdobranin and the clindamycin control, with (5*R*)-**2** eliciting pronounced early reporter activation within 2 h, whereas the response to clindamycin developed more gradually during prolonged incubation.

The effect on protein biosynthesis was further examined using a coupled cell-free transcription-translation assay. Rhabdobranin (5*R*)-**2** inhibited mNeonGreen synthesis in a concentration-dependent manner, resulting in reductions of approximately 25% at 5 μM and 40% at 50 μM, with an almost complete inhibition observed at 250 μM (Figure 4E). Together with the pathway-specific bioreporter profiles, these results identify protein biosynthesis as the principal cellular pathway affected by rhabdobranin (5*R*)-**2** and support interference with the bacterial translation machinery.

## Discussion & Conclusions

Resistance-guided genome mining implicated the *rdb* biosynthetic gene cluster as encoding a prodrug-activated antibacterial metabolite, but the inability to isolate mature rhabdobranin had prevented direct evaluation of this hypothesis. Here we report a convergent synthetic strategy that provided access to pre-rhabdobranin, rhabdobranin, and key analogues, enabling both structural revision and biological evaluation of this cryptic antibiotic family.

The synthesis revealed a subtle but biologically decisive stereochemical misassignment. Revision of pre-rhabdobranin established the *N*-terminal arginine-derived stereocenter as (5*R*) rather than (5*S*). Strikingly, the corresponding rhabdobranin epimers were virtually indistinguishable by conventional analytical methods, including NMR spectroscopy, optical rotation, and HPLC analysis. Despite this analytical similarity, inversion at C–5 caused a pronounced loss of antibacterial activity. Thus, analysis of the prodrug precursor proved critical for stereochemical reassignment, as analysis of the mature rhabdobranin alone would likely have missed this error. This finding underscores a broader challenge in the structural elucidation of conformationally flexible natural products, where subtle stereochemical errors can remain spectroscopically hidden yet dominate biological function. Orthogonal approaches,^67^ including MicroED,^68,69^ crystalline-sponge analysis,^70,71^ and advanced chiroptical methods,^72,73^ may therefore be essential for high-confidence assignment of noncrystalline natural products.

Beyond structural revision, the synthetic route enabled biological evaluation and preliminary structure–activity analysis. Rhabdobranin inhibited several clinically relevant members of the ESKAPEE-focused pathogen panel, including *S. aureus*, *E. coli*, and *K. pneumoniae*. It also displayed pH-dependent activity against resistant *E. faecium* isolates, suggesting that modulation of the scaffold’s physicochemical properties may expand activity toward vancomycin-resistant *Enterococci*. Structure–function studies showed that *N*-acetylation, removal of the branched d-Pro–Ser motif, or inversion at C–5 strongly attenuated activity, identifying these elements as key pharmacophores. The loss of activity upon *N*-acetylation also provides functional support for RdbK-mediated acetylation as a secondary self-resistance mechanism, directly linking the resistance logic encoded in the *rdb* locus to whole-cell antibacterial phenotypes.

Secondary antimicrobial evaluation further revealed micromolar MIC values against five *K. pneumoniae* strains, including carbapenem-resistant clinical isolates representative of the WHO critical-priority pathogen category. This activity is notable given the clinical importance of carbapenem-resistant *K. pneumoniae*, which combines hospital adaptation, dissemination of carbapenemase genes, and limited therapeutic options. Together with its comparatively narrow antibacterial spectrum, bactericidal activity, and apparent evasion of TolC-mediated efflux, these findings position rhabdobranin as a promising scaffold for mechanistic and medicinal chemistry studies. Importantly, the synthetic platform developed here provides reliable access to sufficient material for these investigations, including identification of its molecular target and systematic exploration of the structural features governing its antibacterial activity.

Collectively, these findings show how resistance-gene-guided biosynthetic mining, total synthesis, and functional profiling can be integrated to uncover cryptic antibiotics from Gram-negative bacteria. More broadly, this work reinforces entomopathogenic bacteria as a rich source of chemically distinct antibacterial scaffolds and highlights the central role of chemical synthesis in unlocking the biological function of dark-matter natural products.^74^

## Supplementary Material



## Notes

Experimental procedures, compound characterization data and biological assay methods (PDF)

## Abbreviations

ATCC: American type culture collection
BGC: biosynthetic gene cluster
BHI: brain heart infusion
Boc: *tert*-butoxycarbonyl
BPPL: bacterial priority pathogens list
CoA: coenzyme A
CSB: center for synthetic biochemistry
DIBAL: di-*iso*-butylaluminum hydride
DIPEA: *N*,*N*-di-*iso*-propylethylamine
DMF: *N*,*N*-dimethylformamide
DMSO: dimethyl sulfoxide
DSM: Deutsche Sammlung von Mikroorganismen and Zellkulturen
EDC: 1-ethyl-3-(3-(dimethylamino)­propyl)­carbodiimide
ESI–MS: electrospray ionization mass spectrometry
ESKAPEE: *Enterococcus faecium*, *Staphylococcus aureus*, *Klebsiella pneumoniae*, *Acinetobacter baumannii*, *Pseudomonas aeruginosa*, *Enterobacter* spp., and *Escherichia coli*
Fmoc: 9-fluorenylmethoxycarbonyl
GNAT: GCN5-related *N*-acetyltransferase
HATU: 1-[bis­(dimethylamino)­methylene]-1*H*-1,2,3-triazolo­[4,5-*b*]­pyridinium 3-oxide hexafluorophosphate
HEK293T: human embryonic kidney 293T cells
HOBt: 1-hydroxybenzotriazole
HPLC: high-performance liquid chromatography
HRMS: high-resolution mass spectrometry
MBC: minimum bactericidal concentration
MIC: minimum inhibitory concentration
Myr: myristoyl
NCTC: National collection of type cultures
NMP: *N*-methyl-2-pyrrolidone
NMR: nuclear magnetic resonance
NRP: nonribosomal peptide
NRPS: nonribosomal peptide synthetase
PK: polyketide
PKS: polyketide synthase
PK–NRP: polyketide–nonribosomal peptide
SD: standard deviation
TFA: trifluoroacetic acid
THF: tetrahydrofuran
VRE: vancomycin-resistant *Enterococcus*
WHO: world health organization
WT: wild type.
