# Supplementary material for: Total Synthesis and Structural Revision of Rhabdobranin Reveals a Cryptic Gram-Negative Antibiotic
Source: J Am Chem Soc. 2026 Aug 27;148(35):38069–79. doi: 10.1021/jacs.6c09931 (PMC13564427; doi:10.1021/jacs.6c09931)
Supplement: Supplementary file 1 [file ja6c09931_si_001.pdf]

Supporting Information
for

Total Synthesis and Structural Revision of Rhabdobranin Reveals a Cryptic Gram-Negative Antibiotic

Woonkee S. Jo[1], Zaynoun Attieh[2]†, Jan Johannes Crames[3]†, Carl Leonhard Knopp[4], Julie Connelly[2], Yi-Ming Shi[5,6], Christopher S. Wilson[3], Maryam Dehghan[3], Peter Grün[3], Sunna Chang[1], Fathima S. Refai[7,8], Fabio Piano[7,9], Kristin C. Gunsalus[2,7], Tanja Schneider[4,10], Helge B. Bode*[3,11,12,13], Alan R. Healy*[1]

Affiliations:
[1] Chemistry Program, New York University Abu Dhabi, Saadiyat Island, 129188, United Arab Emirates (UAE)
[2] Center for Genomics and Systems Biology, New York University Abu Dhabi, Saadiyat Island, 129188, UAE
[3] Max Planck Institute for Terrestrial Microbiology, Department of Natural Products in Organismic Interactions, Marburg, 35043, Germany
[4] Institute for Pharmaceutical Microbiology, University Hospital Bonn, University of Bonn, Bonn, 53113, Germany
[5] State Key Laboratory of Quantitative Synthetic Biology, Center for Synthetic Biochemistry, Shenzhen Institute of Synthetic Biology, Shenzhen Institutes of Advanced Technology, Chinese Academy of Sciences, Shenzhen, 518055, China
[6] Max Planck–Chinese Academy of Sciences Center for Synthetic Biochemistry, Shenzhen, 518055, China
[7] Biology Program, New York University Abu Dhabi, Saadiyat Island, 129188, UAE
[8] Dipartimento di Medicina, Università di Udine, Udine, 33100, Italy
[9] Center for Genomics and Systems Biology and Department of Biology, New York University, New York, 10003, USA
[10] DZIF, German Center for Infectious Research, partner site Bonn-Cologne, Bonn, 53113, Germany
[11] Center for Synthetic Microbiology (SYNMIKRO), Philipps University Marburg, Marburg, 35043, Germany
[12] Department of Chemistry, Philipps University Marburg, Marburg, 35043, Germany
[13] Max Planck–Chinese Academy of Sciences Center for Synthetic Biochemistry, Marburg, 35043, Germany

*Corresponding authors: helge.bode@mpi-marburg.mpg.de, alan.healy@nyu.edu
† These authors contributed equally to this work

Table of Contents

Plasmid assembly 3

Production of NRPs 3

Analysis of NRPs 3

General Experimental Methods 3

Materials and Methods 4

β-galactosidase reporter assays 4

Agar-based B. subtilis bioluminescence reporter assay 4

Time-resolved liquid B. subtilis pathway-specific bioluminescence reporter assays 5

Construction of pET21b-mNeonGreen and cell-free transcription–translation assay 5

Large-scale *in vitro* conversion of pre-rhabdobranin to rhabdobranin 5

Analysis of A-domain specificity of RdbF 6

Heterologous expression, isolation and characterization of acetyl transferase RdbK and *in vitro* assay conditions 7

Growth curve for the verification of *rdbK* conferred rhabdobranin resistance 8

Bacterial Strains and Culture 8

Antimicrobial Screening 8

Minimum Inhibitory Concentration (MIC) Determination 9

Time–Kill Kinetics and Determination of Minimum Bactericidal Concentration (MBC). ... 9

Cell viability assay 9

Instrumentation 9

Supplementary Figures and Tables 11

Synthetic Procedures of Proposed Rhabdobranin. 44

Synthetic Procedures of Nominal Pre-rhabdobranin B 67

Synthetic Procedures of Revised Pre-rhabdobranin B 75

Synthetic Procedures of Revised Rhabdobranin 90

Synthetic Procedures of Linear Rhabdobranin. 96

Synthetic Procedures of *N*-acetylated Rhabdobranin 98

Synthetic Procedures of L-Asn-L-Arg Pre-rhabdobranin B. 101

Catalog of nuclear magnetic resonance data 109

References 245

Plasmid assembly. DNA stretches of interest and the vector backbones were amplified by PCR using primers with overhangs as described previously.[1] Q5® High-Fidelity DNA Polymerase (NEB) was used for all reactions with the corresponding protocol. PCR products were validated and isolated on 1 % (*w*/*v*) agarose gel. To purify fragments from gel Monarch® DNA Gel Extraction Kit (NEB) was used according to manufacturer's protocol. Fragments of DNA were isolated using agarose gel electrophoresis (120 V, 40 min, 1 % TAE agarose gel). The DNA was purified from the gel by using the Monarch® DNA gel extraction kit (New England Biolabs) according to the company's protocol. All constructs were constructed using Gibson assembly using NEBuilder® HiFi DNA Assembly Cloning Kit (NEB) was used according to the manufacturer's protocol, and the reaction mixture was incubated at 50 °C for 1 h. Plasmid was confirmed using whole plasmid sequencing.

Production of NRPs. Plasmid combinations were transformed into *E. coli* DH10B::*mtaA* for preparation of overnight cultures as described previously.[1] Strains were incubated at 37 °C in lysogeny broth (LB) with respective antibiotics (chloramphenicol (Cm) (34 µg/mL in ethanol), spectinomycin (Spec) (100 µg/mL). After 24 h the overnight cultures were diluted 1:100 so that 100 µL was added to 10 mL of LB containing chloramphenicol (Cm) (34 µg/mL in ethanol), spectinomycin (Spec) (100 µg/mL) and arabinose (0.2 % (*v*/*v*)). Production cultures were incubated at 25 °C for 48 h. Extraction of natural product was performed at the 48 h timepoint whereby a 500 µL aliquot of the culture was mixed with 500 µL of a 1:1 CH_3OH:CH_3CN mixture for 30 minutes. Samples were then centrifuged for 30 minutes at 14,000 rpm. A 100 µL aliquot was then prepared for HPLC-MS analysis.

Analysis of NRPs. High performance liquid chromatography coupled with mass spectrometry (HPLC-MS) analysis was conducted using a Dionex Ultimate 3000 (Thermo Fisher Scientific) HPLC coupled to an AmaZon Speed (Bruker) electrospray ionisation-ion trap (ESI-IT) mass spectrometer as described previously.[1] Briefly, 5 µL of sample was injected and a gradient of 5 %B-95 %B (B: acetonitrile +0.1 % formic acid A: water +0.1 % formic acid) over 16 minutes. The mass spectrometer was set to measure in positive ionization mode with a mass range of *m/z* 100–1200 and a cone voltage of 4500 V. Data was analysed using DataAnalysis (Bruker).

General Experimental Methods. All chemical reactions were magnetically stirred and conducted in a flame-dried single neck round-bottomed flask under a positive pressure of argon (i.e., balloon) unless otherwise noted. Liquid reagents/solvents were transferred with a plastic Air-Tite syringe (glass microsyringe for small scale reactions) or by an oven-dried stainless-steel cannula through rubber septa. All purified products were dried under high vacuum (overnight if necessary). Organic solutions and volatiles were concentrated with a rotary evaporator at a bath temperature of 30 – 32 °C. All organic extracts were dried over anhydrous sodium sulfate. Low temperature reactions were carried out in a Dewar vessel or Cool-it-bowl filled with a cooling agent: acetone/dry ice (–78 °C) or water/ice (0 °C). Alternatively, Julabo FT-902 immersion cooler was used for multi-gram scale transformations. Reactions requiring temperatures above 23 °C were carried out in a heating mantle and external block temperatures were measured. All commercial solvents (dichloromethane, tetrahydrofuran, diethyl ether, acetonitrile, *N*,*N*-dimethylformamide and methanol) were dried with a solvent dispensing system (Inertcorp; model number: PS-MD-7-EN) by passing through activated alumina. Thin layer chromatography was performed with SiliaPlate

silica glass back scored plates (60Å). Visualization of the spots was achieved by UV irradiation at 245/365 nm (Analytikjena, 6W/0.16A) or by potassium permanganate staining then charring. Purification of crude products was done by automated flash column chromatography (Biotage Selekt) using silica (particle size 230-400 mesh) unless otherwise noted. Yields refer to spectroscopically (^{1}H, ^{13}C) pure and homogeneous materials unless otherwise noted. Plasmid construction and cloning followed standard protocols.[1,2]

Materials and Methods. All reagents were purchased from Sigma-Aldrich or Ambeed and used without further purification with the following exceptions. (*R*)-4-benzyl-3-(2-bromoacetyl)oxazolidin-2-one (**B**)[3] and Fmoc-Boc(Orn)-OH (**S8**)[4] were prepared according to the literature. Triethylamine and diisopropylethylamine were distilled by short-path vacuum distillation and dried over potassium hydroxide. DMSO-d_6 was dried over activated (115 °C, 24h) 3 Å molecular sieves. *Xenorhabdus budapestensis* and *Escherichia coli* strains and mutants were obtained and cultivated as described previously.[1]

β-galactosidase reporter assays

B. subtilis β-galactosidase reporter assays were performed as previously described.[5] Briefly, reporter strains carrying promoter fusions specific for cell wall (P_{ypuA}), DNA (P_{yorB}), RNA (P_{yvgS}) and protein synthesis (P_{yheI}) interference were grown in MHB containing 5 µg/mL chloramphenicol at 30 °C to an OD_{600} of 0.5. Subsequently, cells were poured into Müller Hinton Agar supplemented with 5 µg/ml chloramphenicol at a final cell density of 1 × 10^{7} CFU/mL. 5-Bromo-4-chloro-3-indolyl-β-D-galactopyranoside (X-Gal) was added to the agar plates at 75 µg/mL (cell wall reporter), 125 µg/mL (DNA reporter), and 250 µg/mL (RNA and protein reporters), respectively. After solidification of the plates, 60 µg of rhabdobranin (5*R*)-**2** and derivatives were spotted. Control antibiotics selectively inducing the promotors (6 µg vancomycin for cell wall, 0.3 µg ciprofloxacin for DNA, 6 µg rifampicin for RNA, 3 µg clindamycin for protein), and non-inducing controls (3 µg clindamycin for cell wall, DNA and RNA, 6 µg vancomycin for protein) were added. Plates were incubated for 24 h at 30 °C overnight. Results are representative of three independent experiments.

Agar-based B. subtilis bioluminescence reporter assay

The agar-based bioluminescence reporter assay was performed using *B. subtilis* 168 pCHlux CLK4 P_{yheI},[6] which responds to protein biosynthesis stress through expression of the *Photorhabdus luminescens* luxCDABE luciferase gene cluster. Briefly, the reporter strain was grown at 30 °C in Mueller–Hinton (MH) broth supplemented with 5 µg/mL chloramphenicol to an OD_{600} of 0.5. Molten MH agar (40 mL) was cooled to 50 °C and supplemented with 5 µg/mL chloramphenicol. The reporter culture was added to the molten agar at a final density of 1 × 10^{7} CFU/mL. The inoculated agar was poured into Petri dishes and allowed to solidify. Rhabdobranin (5*R*)-**2** and derivatives were applied to the agar surface at 15 µg per spot. Clindamycin (1.5 µg) and vancomycin (3 µg) were included as positive and negative controls, respectively. Plates were incubated at 30 °C for 18 h, after which reporter induction was assessed by bioluminescence imaging (Biorad, Chemidoc).

Time-resolved liquid B. subtilis pathway-specific bioluminescence reporter assays
Luciferase reporter assays were conducted as described previously,[6] using *B. subtilis* bioreporters carrying promoter-*lux* fusions specific for cell-wall (P_{ypuA}), DNA (P_{yorB}), RNA (P_{yvgS}), and protein-biosynthesis (P_{yheI}) stress responses.
Briefly, reporter strains were grown at 30 °C in Mueller–Hinton broth (MHB) containing 5 µg/mL chloramphenicol to exponential growth phase (OD_{600} = 0.4–0.6). Serial dilutions of rhabdobranin (5*R*)-**2** and derivatives, as well as the respective reference antibiotics were prepared in clear 96-well chimney plates in a volume of 50 µL per well. An equal volume of the respective reporter culture was subsequently added to each well. Vancomycin, ciprofloxacin, rifampicin, and clindamycin were used as reference antibiotics for the cell-wall, DNA, RNA, and protein-biosynthesis stress reporters, respectively. Luminescence and OD_{600} were recorded over 10-15 h using a Spark multimode microplate reader (Tecan). Reporter activity was expressed as luminescence normalized to the corresponding OD_{600} value (RLU/OD_{600}).

Construction of pET21b-mNeonGreen and cell-free transcription–translation assay
The pET21b backbone and mNeonGreen insert were amplified using the primer pairs pET21_FW (5′-TGAGATCCGGCTGCTAACA-3′)/pET21_RV (5′-ATGTATATCTCCTTCTTAAAGTTAA-3′) and mNeonGreen_FW (5′-GAAGGAGATATACATATGGTGAGCAAGGGCGAGG-3′)/mNeonGreen_RV (5′-AGCAGCCGGATCTCACTTGTACAGCTCGTCCATGCC-3′), respectively. The fragments were assembled using In-Fusion cloning (Takara Bio, cat. no. 638946) and transformed into *Escherichia coli* DH5α (NEB, cat. no. C2987I). Transformants were selected on LB agar containing 100 µg/mL ampicillin. Plasmid DNA was isolated using the Monarch Plasmid Miniprep Kit (NEB, cat. no. T1110) and quantified using a NanoDrop spectrophotometer.
Coupled cell-free transcription–translation reactions were performed using the PURExpress® In Vitro Protein Synthesis Kit (NEB, cat. no. E6800), with pET21b-mNeonGreen serving as the DNA template. Each 25 µL reaction contained 5 µL Solution A, 3.75 µL Solution B, 1.25 µL murine RNase inhibitor (NEB, cat. no. M0628), 60 ng plasmid DNA, the respective test compound, and nuclease-free water. Clindamycin served as a translation-inhibitor control, and the DMSO concentration was kept constant across corresponding reactions. Following incubation at 37 °C for 2 h, mNeonGreen fluorescence was measured using a Spark microplate reader (Tecan) at 485 nm excitation and 535 nm emission. Values were normalized to the DMSO control, which was set to 100%.

Large-scale *in vitro* conversion of pre-rhabdobranin to rhabdobranin. Natural rhabdobranin was obtained by *in vitro* conversion of pre-rhabdobranin. The heterologous production of rhabdobranin peptidase was conducted using *Escherichia coli* C41(DE3) ΔacrAB pET-29b-rdbP constructed according to standard protocols. Briefly, a ratio of 1:100 of an overnight culture was inoculated into a main culture of three times 1 L LB in 5 L flasks, with 50 µg/mL Km. The culture was incubated at 37 °C and 180 rpm until OD_{600} = 0.6 was reached, and induced with 1 mM IPTG, followed by further cultivation for 12 h. To isolate membranes containing RdbP, cells were harvested at 10,000 rpm for 10 minutes. Further steps were conducted at 4 °C or on ice. Subsequently, the cell pellet is dissolved in 3 mL per gram wet weight of cells in French Press Buffer (20 mM Tris, pH 8, 500 mM NaCl), with the addition of 5 mM $MgCl_2$, 0.25 mg/mL

lysozyme and 10 µg/mL DNase I, under stirring. Cell disruption was performed by French press (Stansted Homogeniser EP FPG12805) at 30 kpsi with three runs. For the removal of cell debris, centrifugation was performed at 14,000 rpm for 20 min (Thermo Scientific, rotor type F14-6 × 250y). The supernatant was then centrifuged at 40,000 rpm for 60 min in the ultracentrifuge (Thermo Scientific, rotor type 45 TI), and the resulting membrane pellet was dissolved in 4 mL per gram wet weight wash buffer (20 mM Tris-HCl pH 7.8, 150 mM NaCl, 10% glycerol, 0.03% Dodecyl-β-D-maltoside). Finally, the RdbP-containing membrane was incubated at 4 °C on the roll-mixer for one hour, aliquoted into 500 µL and stored at –80 °C.

For the production of pre-rhabdobranin, a 6 L LB production culture with 50 µg/mL Km, 4% of Amberlite XAD-16 in six 5 L cultivation flasks was inoculated with an overnight culture of *Xenorhabdus budapestensis* DSM 16342 Δ*rdbP* P_{BAD}-*rdbA* 1:50 and induced with 0.4 % arabinose. The cultivation was performed at 30 °C and 180 rpm for 72 h. The Amberlite XAD-16 was isolated and washed with 1 L of deionized water, to remove hydrophilic compounds remaining from the medium. For the elution of the products from the Amberlite XAD-16, 1 L of methanol was added and incubated at 130 rpm for 30 min, the extract was filtered using a folded filter, grade 3 m/N, dia 240 mm (MUNKTELL & FILTRAK GmbH), and methanol was evaporated. The resulting extracted products (6.93 g) were dissolved under ultrasonication 15 mL methanol and 15 mL of deionized water and centrifuged at 3220 × g for 30 minutes. Subsequently, purification using the 1260 Infinity II system (Agilent) equipped with a 10 Prep-C18-column 250 × 30.0 mm (Agilent), coupled to aG6125B-LC MSD ESI-MS Agilent (Gradient: 5% to 65% acetonitrile to water in 40 minutes with a flow-rate of 40 mL/min, water and acetonitrile were supplemented with 0.1% of formic acid), was carried out. Under this condition, fractions from a retention time of 4 min to 11 min were taken and unified. An HPLC-HR-MS analysis of the unified fractions (1.22 g) was performed. A mass of 0.61 g of the extract was stored and used for further analysis of pre-rhabdobranin.

For the large-scale *in vitro* conversion, the extract (0.61 g) was dissolved in 305 mL IV Buffer (20 mM Tris-HCl pH 8, 150 mM NaCl) and the resulting solution was centrifuged at 4000 xg for 15 minutes. The supernatant was transferred into a sterile 1 L Erlenmeyer flask and 3.05 mL of the thawed prepared membrane was added. The *in vitro* reaction mixture was incubated at 22 °C and 70 rpm for 24 hours, subsequently, the reaction mixture was quenched by adding 152.5 mL of acetonitrile and centrifuged at 4.000 xg for 25 min. The supernatant was lyophilized, and redissolved in 6 mL of water and 1 mL methanol under ultrasonication, and centrifugation at 17.000 xg for 20 min. Subsequently, rhabdobranin was isolated, using the 1260 Infinity II system (Agilent) equipped with a Waters XBridge BEH Amide OBD Prep column, 130 Å, 5 µm, 10 × 250 mm (Agilent), coupled to a G6125B-LC MSD ESI-MS (Agilent) (Gradient: 90% to 61% acetonitrile in 40 minutes at a flow rate of 3 mL/min); Fractions from a retention time of 23.72 min to 26.29 min were taken, unified and lyophilized, resulting in 15 mg of rhabdobranin. NMR analysis was performed to verify the purity of the isolated compound.

Analysis of A-domain specificity of RdbF. In order to elucidate whether RdbF activates and binds L- or D-Arg, a fusion between RdbF and AmbS from *X. indica* was performed based on intein splicing using the NrdJ-1 intein as described previously.[2] Therefore, full length *rdbD* and part of *rdbF* were amplified by PCR from *X. budapestensis* genomic DNA and cloned applying standard procedure generating plasmid pINCW01 that encodes both NRPS genes, resulting in a RdbF fusion to the NrdJ-1_N as shown previously for several other split NRPS.[2] The plasmid was co-transformed

into chemically competent *E. coli* DH10B::*mtaA* already carrying pIN218 (encoding the compatible AmbS) and clones carrying both plasmids were cultivated in 5 mL LB medium with 0.2 % arabinose to induce expression of the NRPS parts. HPLC-MS analysis of the cultures after three days of cultivation revealed production of the expected peptide. The configuration of the Arg residue was compared to synthetic standards carrying either L- or D-Arg based on retention time in the HPLC-MS as well as the MS-MS fragmentation (**Supplementary Figure S3**; *vide infra*). The synthetic standards were synthesized using a microwave-assisted peptide synthesizer (Liberty Prime 2.0, CEM Corporation) equipped with an HT12 high-throughput loader. The 9-fluorenylmethoxycarbonyl (Fmoc) strategy was employed, using *N*,*N*′-diisopropylcarbodiimide/ethyl 2-cyano-2-(hydroxyimino)acetate (DIC/Oxyma) as coupling agents. Both peptides were assembled on Fmoc-L-Lys preloaded Wang resin (50 µmol) through automated iterative cycles consisting of coupling (105 °C, 1 min), deprotection (110 °C, 40 s), and washing steps. Amino acid deprotection was carried out using 25% (*v*/*v*) pyrrolidine in *N*,*N*-dimethylformamide (DMF). Final global deprotection and cleavage were performed at room temperature for 1 h using 3 mL of a cleavage cocktail (trifluoroacetic acid (TFA), H_2O, and triisopropylsilane (TIS), 95:2.5:2.5 (*v*/*v*)).

Heterologous expression, isolation and characterization of acetyl transferase RdbK and *in vitro* assay conditions. For the characterization of acetyltransferase RdbK, the enzyme was produced by heterologous expression and subsequently isolated following standard procedures. For the expression of a SUMO-3-tagged construct of the *N*-acetyltransferase, the pET11a vector was used.[1] The vector backbone was amplified by PCR using primer pET11a_for and pET_SMT3_rev, while the *rdbK* insert with introduced pET-11a-overhangs was amplified from gDNA. Both, the vector backbone and insert were fused using the NEBuilder HiFi DNA Assembly (New England Biolabs). The plasmid was transformed into *Escherichia coli* BL21 (DE3) for expression.

An overnight culture of the strain was inoculated 1:50 in 50 mL LB supplemented with 50 µg/mL ampicillin and cultivated at 37 °C and 180 rpm until OD_{600} = 0.8 was reached. Subsequently, the culture was induced by adding 1 mM IPTG and further incubated at 22 °C and 180 rpm for 12 h. The cells were harvested at 10,000 rpm for 10 minutes, and cell lysis was performed by resuspending the pellet in 5 mL BugBuster® (primary amine-free) Extraction Reagent (NOVAGEN) with 1 µL of Benzonase® Nuclease (Merck Millipore), 14 mg of cOmplete™ EDTA-free protease inhibitor (Roche), and lysozyme (200 µg/mL) (Sigma Aldrich), followed by incubation under shaking at 4 °C for 45 min. All further steps were performed at 4 °C or under cooling on ice. The cell debris was removed by centrifugation at 20,000 xg for 30 min, and the supernatant was carefully poured into a Falcon tube. For the isolation of the protein Ni^{2+} affinity chromatography was applied. Therefore, His SpinTrapTM Ni SepharoseTM High-Performance Spin columns (Cytiva) were used according to the manufacturer's instructions. The columns were equilibrated with 600 µL of the His-Tag Binding Buffer (100 mM Tris-HCl pH8, 150 mM NaCl, 20 mM Imidazole) before the supernatant containing the protein was applied onto the column. Subsequently, the columns were washed with 600 µL of His-Tag Binding Buffer two times. Finally, the His-tagged SUMO-3-RdbK protein was eluted using 200 µL of His-Tag Elution Buffer (100 mM Tris-HCl pH8, 150 mM NaCl, 400 mM imidazole), supplemented with 10% glycerol, and stored at –80 °C. For the verification of a successful protein expression and isolation, a volume of 20 µL of samples was taken at each step and SDS-PAGE was conducted (Laemmli 1970).

For the verification of the acetylation catalyzed by RdbK, an *in vitro* approach was conducted. The 50 µL reaction was performed in 50 mM Tris-HCl pH 8 buffer using a concentration of 10 µM RdbK, 1 mM rhabdobranin and 1 mM acetyl CoA (Acetyl coenzyme A lithium salt, Sigma-Aldrich). Additionally controls either containing RdbK or acetyl CoA were prepared. The reaction mixture and controls were then incubated at 30 °C for 12 hours. After quenching by adding 50 µL of acetonitrile and centrifugation at full speed for 25 min, HR-MS-analysis was performed using the Impact II qTOF (Bruker) system.
In order to investigate the acetylation site of RdbK the reaction mixture was scaled up. Thus, the concentration of rhabdobranin and acetyl CoA dissolved in 50 µL of 50 mM Tris-HCl pH 8 was adjusted to 65 mM and 130 mM. The following steps were conducted as previously described, subsequently the acetylated rhabdobranin was isolated using the 1260 Infinity II system (Agilent) equipped with a Waters XBridge BEH Amide OBD Prep column, 130 Å, 5 µm, 10 × 250 mm (Agilent), coupled to a G6125B-LC MSD ESI-MS (Agilent) (Gradient: 90% to 61% acetonitrile in 40 minutes at a flow rate of 3 mL/min); Fractions from a RT of 23.72 minutes to 26.29 minutes were taken and unified. The solvent of the resulting fractions was evaporated by using a lyophilizer, and the structure was confirmed by NMR.

Growth curve for the verification of *rdbK* conferred rhabdobranin resistance. For the verification of the *rdbK* conferred rhabdobranin resistance, an L-arabinose inducible pSEVA261-P_{BAD} construct expressing *rdbK* was generated and transformed into *Escherichia coli* BL21(DE3). An overnight culture of the cells containing the empty and the *rdbK* expressing pSEVA261-P_{BAD}, was inoculated 1:50 into 5 mL LB with 50 µg/mL kanamycin, either induced with 0.4% L-arabinose or non-induced and grown at 37 °C at 180 rpm for 12 hours. The assay was performed in triplicates using a transparent 96-well plate (Corning). Each well- contained 200 µL LB with 25 µg/mL kanamycin, additionally 0.4% of L-arabinose was added to the induced group. The growth of each induced and non-induced group in the presence and absence of 0.5 mM rhabdobranin was recorded using a Spark® multimode microplate reader (TECAN) at 37 °C and 200 rpm for 24 h.

Bacterial Strains and Culture. Bacterial strains were obtained from the National Collection of Type Cultures (NCTC, UK), the Leibniz Institute DSMZ-German Collection of Microorganisms and Cell Cultures (DSMZ, Germany), and the American Type Culture Collection (ATCC, USA), as detailed in **Supplementary Table S1** (*vide infra*). The panel comprised strains representing World Health Organization 2024 critical- and high-priority bacterial pathogen groups[7], together with reference/comparator strains for benchmarking and comparative analysis. Overnight cultures were conducted in Brain Heart Infusion (BHI) or Luria-Bertani (LB) broth at 37 °C with shaking at 200 rpm.

Antimicrobial Screening. Bacterial cultures were diluted in Cation-Adjusted Mueller-Hinton broth (CAMHB, pH 7.3) to achieve a final inoculum of OD_{600} = 0.0005, with the exception of *E. faecium*, which was tested in BHI buffered to pH 7.3 or pH 9.0. Compounds dissolved in dimethyl sulfoxide (DMSO) were screened at 128 µg/mL (final DMSO: 1% *v*/*v*) in 384-well plates. Plates were incubated statically at 37 °C for 18 h, and bacterial growth was assessed by measuring optical density at 600 nm (OD_{600}). Background-corrected absorbance values were normalized to vehicle control wells (bacteria treated with 1% DMSO) to obtain percentage growth. Screening experiments were performed in biological duplicate, each with three technical replicates.

Minimum Inhibitory Concentration (MIC) Determination. MIC values were determined via two-fold serial dilutions (1–128 µg/mL) using the broth microdilution assay described above, maintaining a final inoculum of OD_{600} = 0.0005 and final DMSO concentration of 1% (*v*/*v*). MIC values were determined as the lowest concentration inhibiting visible growth. MIC experiments were performed in biological triplicate, each with three technical replicates.

Time–Kill Kinetics and Determination of Minimum Bactericidal Concentration (MBC). Time-kill assays were performed using *Klebsiella pneumoniae* DSM 103707. Bacteria were prepared in Cation-Adjusted Mueller-Hinton broth under the same inoculum and DMSO conditions used for MIC testing. Cultures were treated with rhabdobranin (5*R*)-**2** at 2, 4, 8, 16, and 32 µg mL^{-1} (0.5–8 × MIC) or with an equivalent volume of vehicle (final DMSO : 1% *v*/*v*) and incubated at 37 °C. At 0, 2, 4, 8, and 24 h, aliquots were serially diluted ten-fold in Phosphate Buffer Saline (PBS) and plated on compound-free Mueller-Hinton agar for CFU enumeration. The limit of detection was 100 CFU mL^{-1}; samples yielding no colonies at the lowest dilution were assigned this value. Assays were performed in three independent biological replicates. Bactericidal activity was defined as a $\geq$3-$\log_{10}$ reduction in CFU mL^{-1} relative to the starting inoculum, in accordance with CLSI M26-A and the MBC as the lowest concentration achieving this reduction at 24 h.[8] Classification as bactericidal or bacteriostatic was based on the MBC/MIC ratio, with $\leq$4 taken as bactericidal.[9]

Cell viability assay. HEK293T cells (ATCC® CRL-3216™) and HeLa cells (ATCC® CCL2™) kindly provided by the Core Technology Platforms Biobank (New York University Abu Dhabi), were cultured in Dulbecco's Modified Eagle Medium supplemented (DMEM) with 10% fetal bovine serum, penicillin (100 U/mL), and streptomycin (100 µg/mL) at 37 °C, 5% CO_2. Cells were seeded in a 96-well plate at 6×10^4 cells/well and allowed to adhere overnight. Compounds at 100 µM were applied by automated liquid handling and incubated for 24 h. Cell viability was measured using the CCK-8 assay kit (96992, Sigma-Aldrich) according to the manufacturer's instructions. For concentration–response analysis, cells were treated with rhabdobranin (5*R*)-**2** at 10, 25, 50, and 100 µM for 24 h. Viability was expressed as a percentage relative to the vehicle control cells (treated with 1% DMSO) and reported as mean ± SD of three independent biological replicates.

Instrumentation. ^{1}H NMR (500 MHz) and ^{13}C NMR (126 MHz) were recorded at standard temperature and pressure on a Bruker Avance III Spectrometer in the indicated solvent (DMSO-d_6). Chemical shifts (^{1}H) are expressed in parts per million (ppm, δ scale) downfield from tetramethylsilane and are referenced to residual protium in the NMR solvent (DMSO-d_6, 2.50 or CD_3OD, 3.31). Data are represented as follows: chemical shift, multiplicity (s = singlet, d = doublet, t = triplet, q = quartet, m = multiplet and/or multiple resonances, br = broad, app = apparent), coupling constant in Hertz, integration, and assignment. Chemical shifts (^{13}C) are expressed in parts per million (ppm, δ scale) downfield from tetramethylsilane and are referenced to the carbon resonances of the solvent (DMSO-d_6, 39.5 or CD_3OD, 49.0). All NMR δ values are given in ppm, and all J values are in Hz. 2D-NMR spectroscopy such as [^{1}H, ^{1}H] COSY (Correlation Spectroscopy), [1H, 13C] HSQC (Heteronuclear Single Quantum Coherence), were also recorded to unambiguously assign all proton and carbon signals unless otherwise noted. The numbering of the hydrogen and the carbon atoms do *not* correspond to the IUPAC nomenclature.

Analytical liquid chromatography/mass spectrometry (SFC (Supercritical Fluid Chromatography)/MS) was performed on Agilent LC/MS instrument (1260 Infinity II) equipped with an InfinityLab Poroshell 120 HILIC column (2.7 μm particle size, 4.6 × 150 mm), electrospray (ESI) mass spectrometry detector, and photodiode array detector. Samples were eluted with methanol (containing 0.1% formic acid and 0.1% ammonium hydroxide)-carbon dioxide (10% methanol–carbon dioxide for 2 min → linear gradient to 30% methanol–carbon dioxide over 2 min → 30% methanol–carbon dioxide for 2 min) at a flow rate of 2 mL/min. High-resolution mass spectrometry (HRMS) was obtained on an UPLC/HRMS instrument (Agilent 1290 Infinity II) equipped with a Q-TOF (UHD Accurate-Mass) and photodiode array detector. Samples were eluted over a guard column with 50% acetonitrile–water containing 0.1% formic acid for 1 min, at a flow rate of 300 μL/min.

Optical rotations were measured on a polarimeter Anton Paar (MCP 5100) at 20 °C at a wavelength of 589 nm in a 1 mL quartz cell (0.5 dm length). The concentration is given in g/100 mL.

Supplementary Figures and Tables.

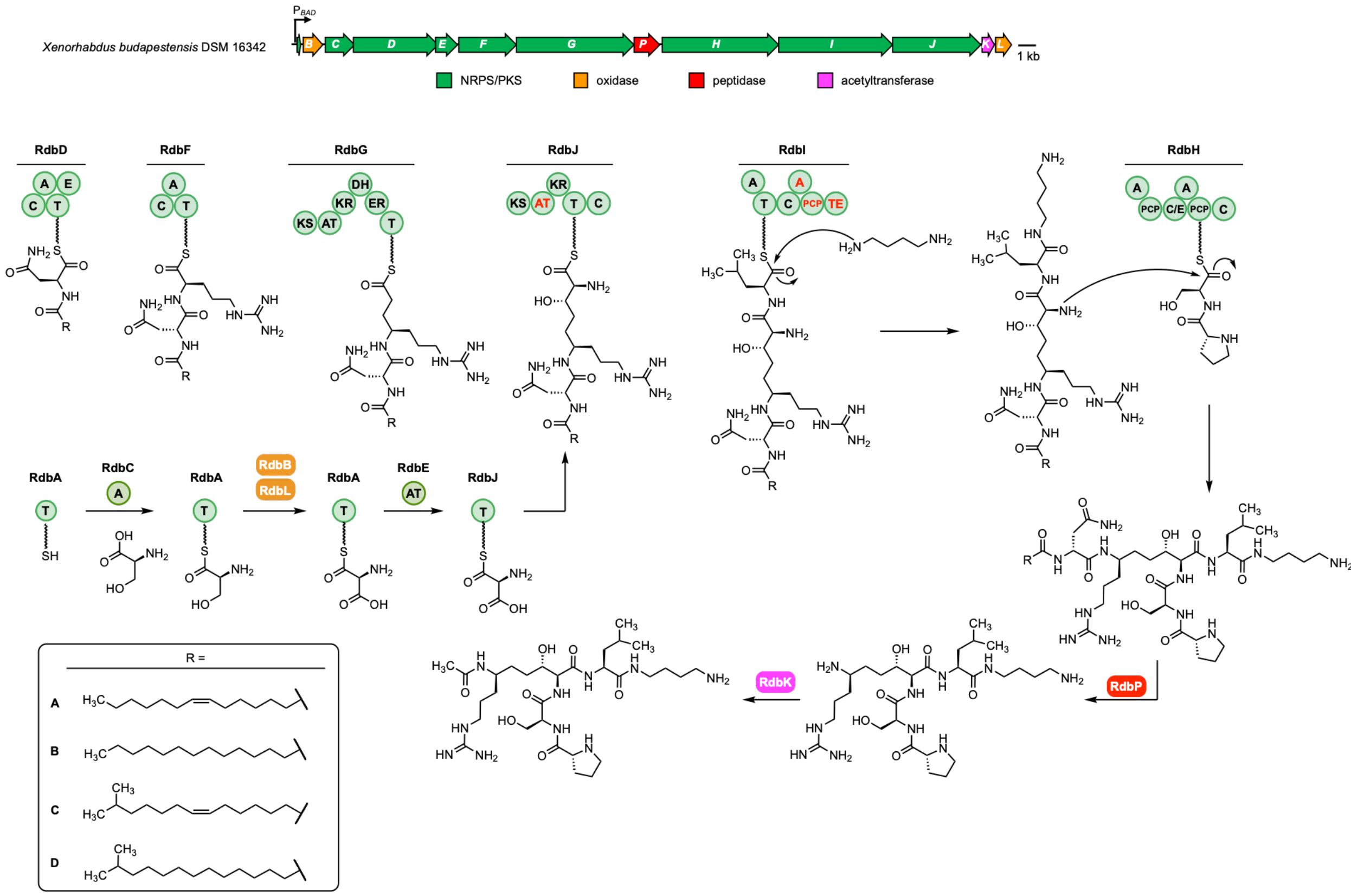

Supplementary Figure S1. Overview of the biosynthesis gene cluster (BGC) from *Xenorhabdus budapestensis* DSM 16342 encoding RdbA-RdbL as well as a proposed biosynthesis pathway highlighting the prodrug cleavage mechanism by RdbP (red) and the acetylation by RdbK (pink). Domain functions are as follows: C (condensation), A (adenylation), T (thiolation), KS (ketosynthase), AT (acyltransferase), KR (ketoreductase), DH (dehydratase), ER (enoylreductase), TE (thioesterase). Inactive domains are denoted in red.

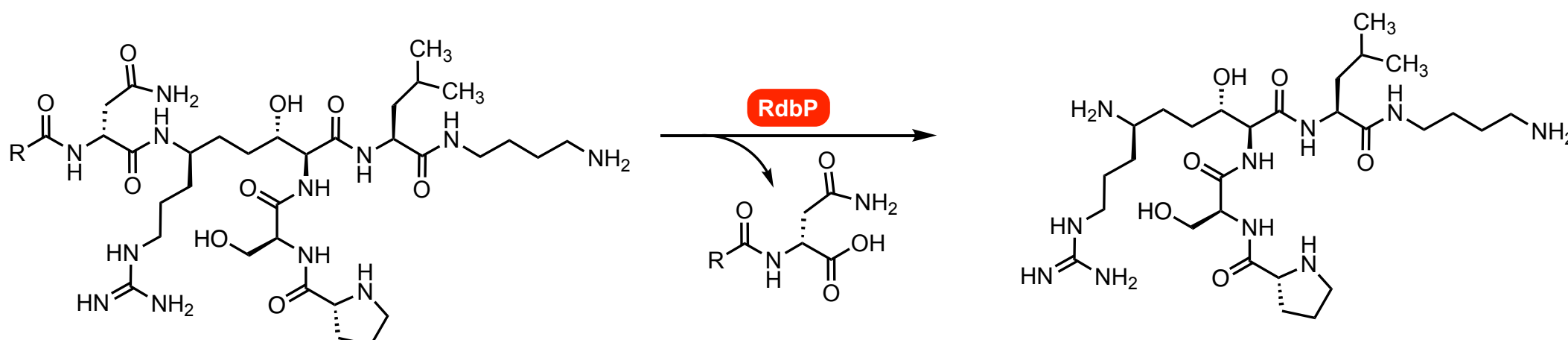

(5*R*)-**2**

Compound	Mass detected	Ion formula	Δppm	R
pre-rhabdobranin A (**I**)	317.895 $[M+3H]^{3+}$	$C_{46}H_{89}N_{12}O_9$	1.9	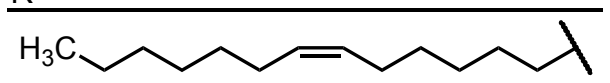
pre-rhabdobranin B (**II**)	318.567 $[M+3H]^{3+}$	$C_{46}H_{91}N_{12}O_9$	0.1	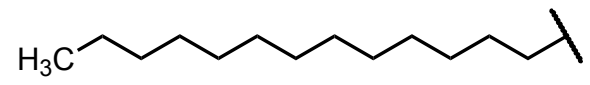
pre-rhabdobranin C (**III**)	322.567 $[M+3H]^{3+}$	$C_{47}H_{91}N_{12}O_9$	1.3	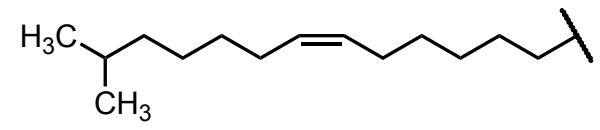
pre-rhabdobranin D (**IV**)	323.239 $[M+3H]^{3+}$	$C_{47}H_{93}N_{12}O_9$	1.4	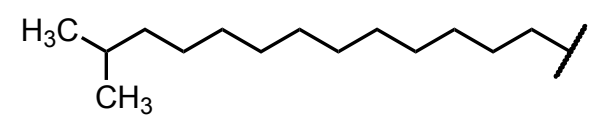

Compound	Mass detected	Ion formula	Δppm
rhabdobranin	315.227 $[M+2H]^{2+}$	$C_{28}H_{57}N_{10}O_6$	–0.2

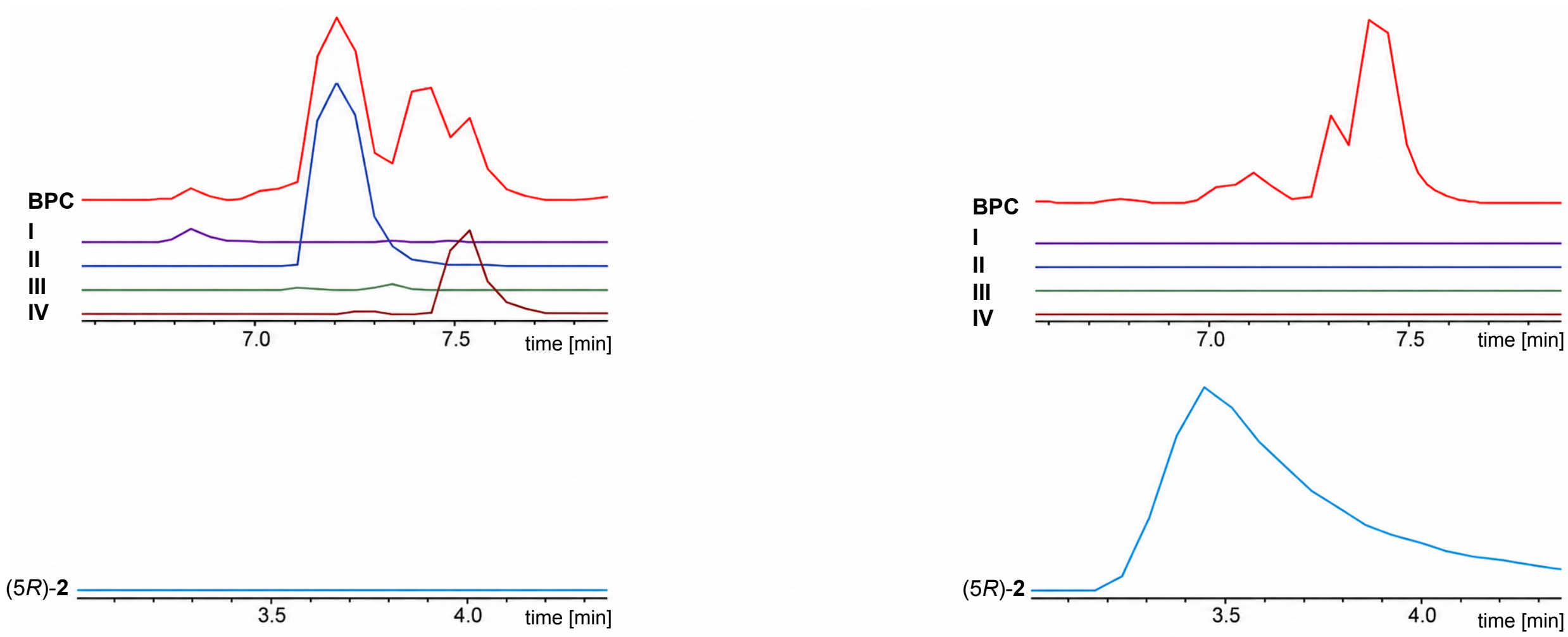

Supplementary Figure S2. Large-scale in vitro conversion of pre-rhabdobranins A, B, C and D (**I**, **II**, **III** and **IV**) isolated from a *Xenorhabdus budapestensis* DSM 16342 ΔrdbP_P_{BAD}_rdbA production culture. Structures and HR-MS data of the *in vitro* converted pre-rhabdobranins (EICs of compound **I**–**IV**) containing fraction to the resulting rhabdobranin (5*R*)-**2** (EIC: extracted ion chromatogram; BPC: base peak chromatogram).

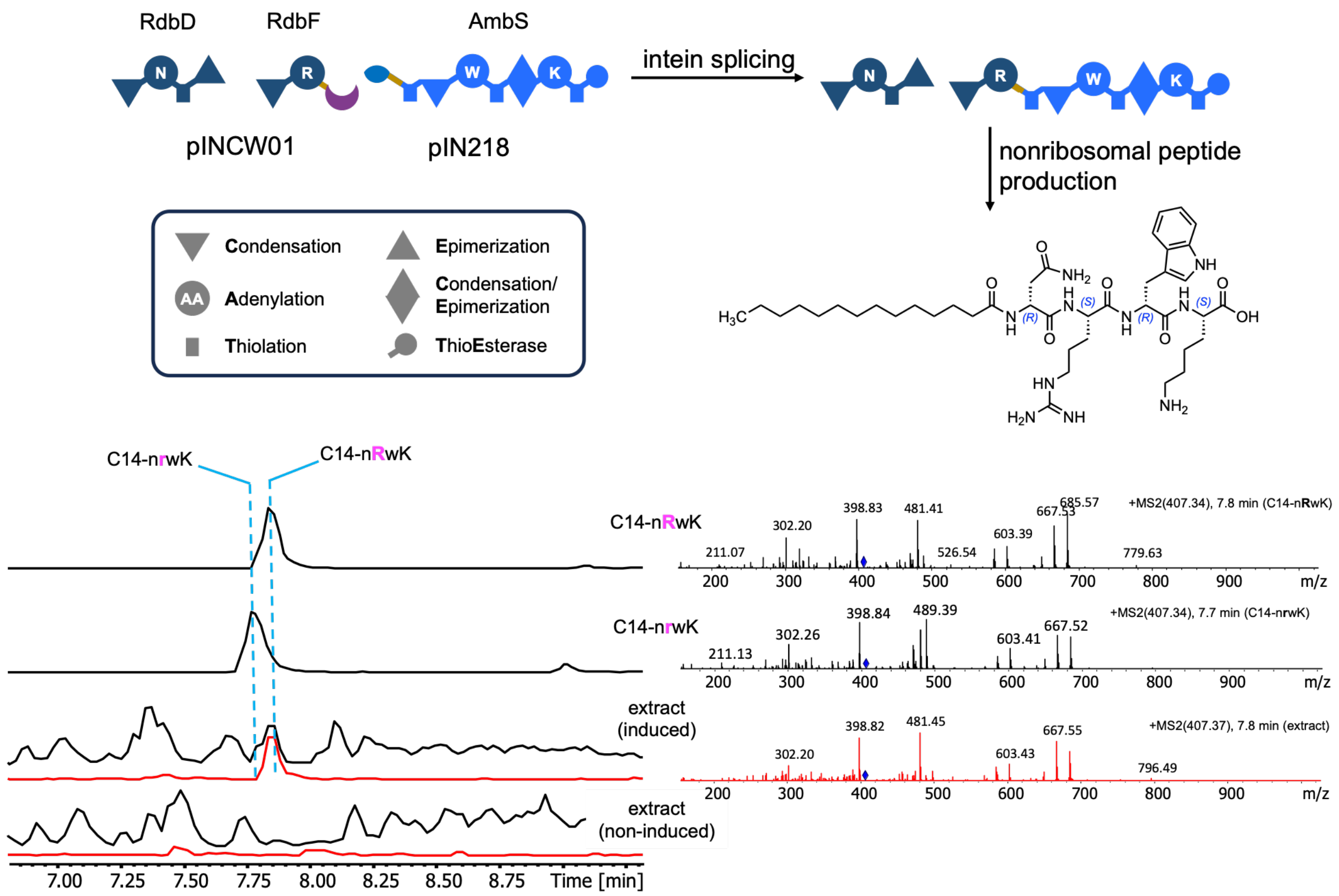

Supplementary Figure S3. Generation of an artificial fusion between plasmid encoded NRPS RdbD-RdbF (pINCW01) and the C-terminal part of the AmbS NRPS (pIN218) based on intein splicing.[2] The resulting NRPS produced the L-Arg containing peptide C14-nRwK (small letters denote D-AAs) as compared with synthetic standards differing in the configuration of the Arg only. MS2 spectra are also shown for the synthetic and natural peptide. Base-peak chromatograms (black) and extracted ion chromatograms (red) of the target mass *m/z* 407.3 $[M+2H]^{2+}$ are shown. The expected mass was only observed in the arabinose induced culture.

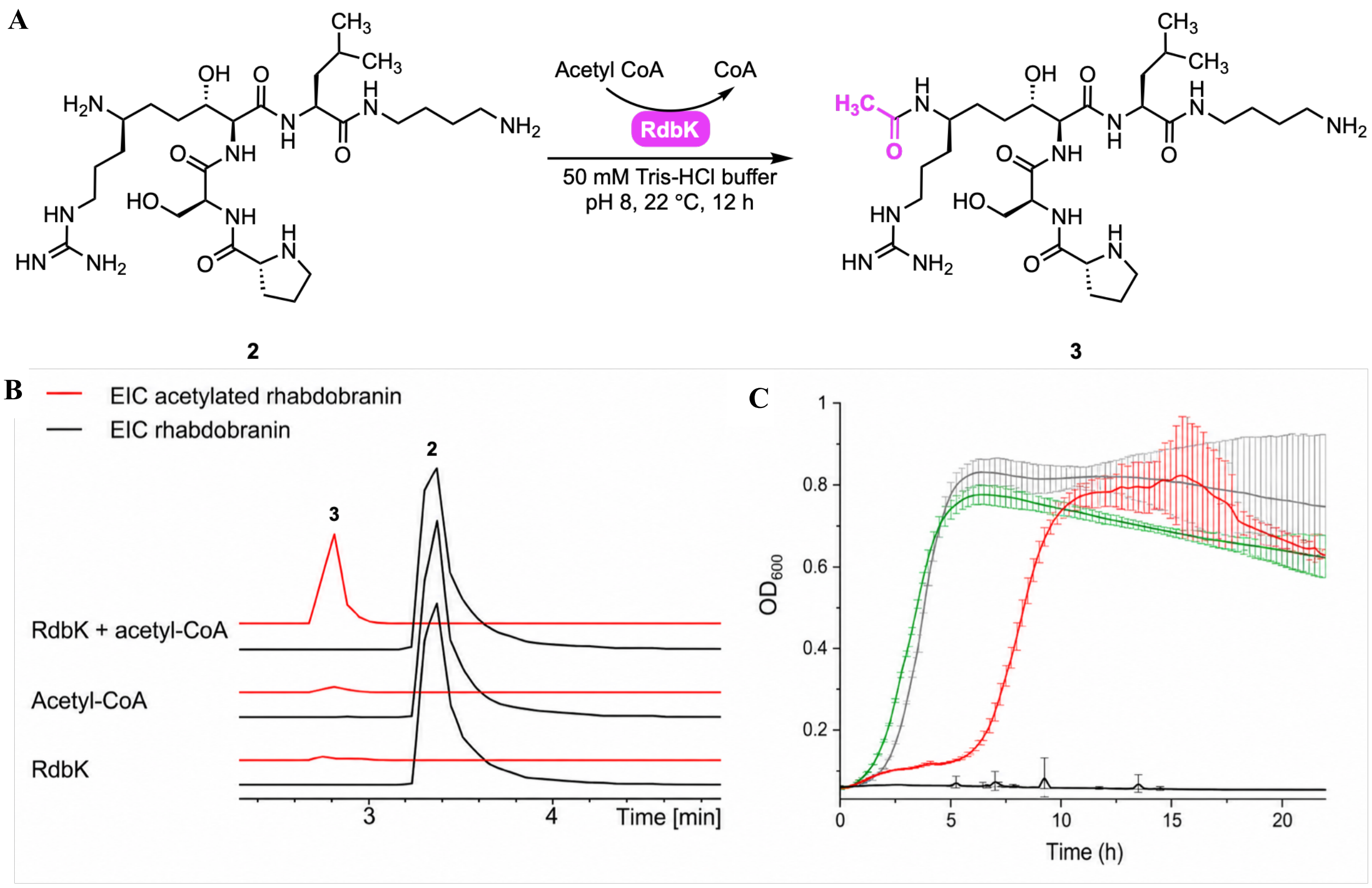

Supplementary Figure S4. Investigation of *N*-acetyltransferase *rdbK* mediated rhabdobranin self-resistance. **A** Conditions and mechanism of *in vitro* RdbK catalyzed acetylation and chemical structure of the resulting *N*-acetyl-rhabdobranin (**3**). **B** HPLC-HR-MS analysis of the RdbK *in vitro* reaction mixture and detection of the *N*-acetyl-rhabdobranin (**3**) (EIC: 671.456 $[M+H]^+$, $C_{30}H_{59}N_{10}O_7$, Δppm: 0.3) in the presence of acetyl CoA. **C** Growth curve of 0.4% L-arabinose induced *Escherichia coli* BL21(DE3) transformed with either pSEVA261-P_{BAD}-rdbK in absence (green line) and presence of 0.5 mM rhabdobranin **2** (red line), or pSEVA261-P_{BAD} empty vector control in absence (gray line) and presence of 0.5 mM rhabdobranin **2** (black line) (Inoculated from 0.4% L-arabinose induced pre-culture, cultured at 37 °C).

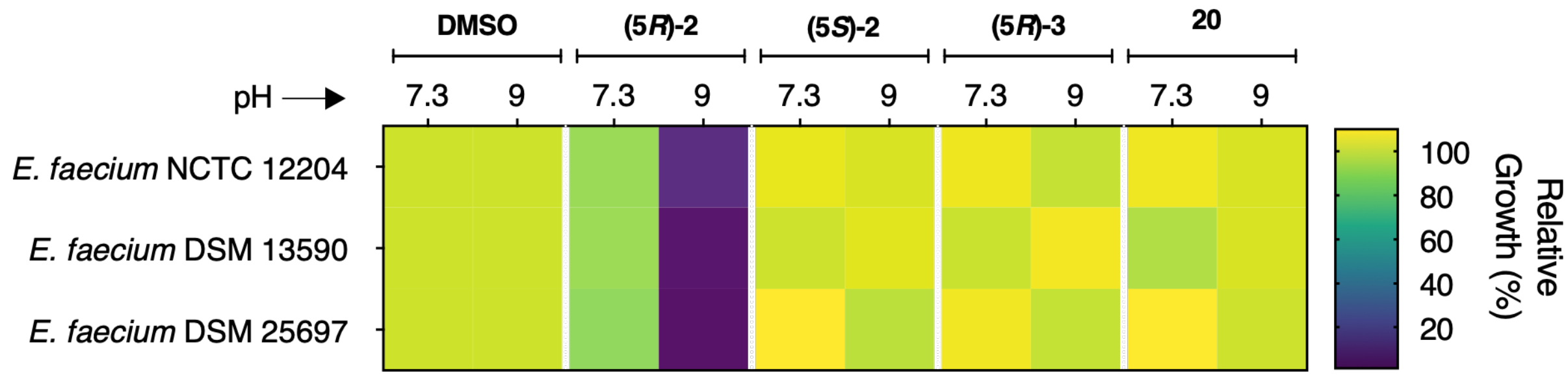

Supplementary Figure S5. **pH-dependent growth inhibition of *Enterococcus faecium* by rhabdobranin and analogues**. Heat map showing relative growth of *Enterococcus faecium* NCTC 12204, DSM 13590, and DSM 25697 in BHI at pH 7.3 or pH 9.0 following treatment with DMSO control, (5*R*) and (5*S*)-**2**, (5*R*)-**3** and **20**. Values are expressed as relative growth (%) normalized to the corresponding DMSO-treated vehicle control at each pH.

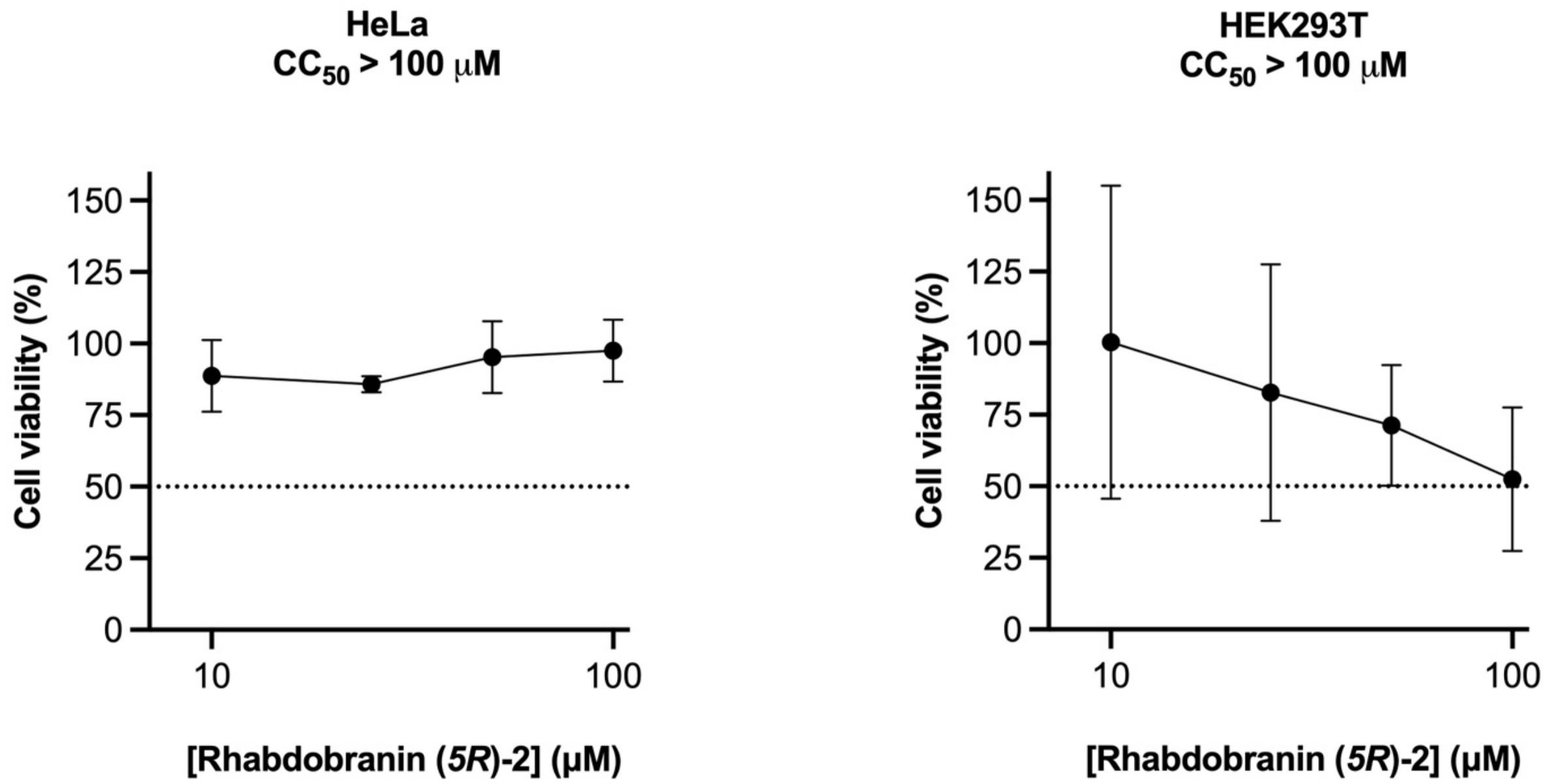

Supplementary Figure S6. Assessment of rhabdobranin (*5R*)-2 selectivity by concentration–response profiling in mammalian cells. Concentration–response analysis of rhabdobranin (*5R*)**-2** cytotoxicity in mammalian cells. HeLa (left) and HEK293T (right) cells were exposed to rhabdobranin (*5R*)**-2** for 24 h. Viability is expressed as a percentage of vehicle-treated controls and plotted as mean ± SD of three biological replicates. Concentrations are shown on a $\log_{10}$ scale; the vehicle control defines 100% viability and is therefore not plotted. Viability remained above 50% at the highest concentration tested in both cell lines, and half-maximal cytotoxic concentration (CC_{50}) values are therefore reported as greater than 100 µM.

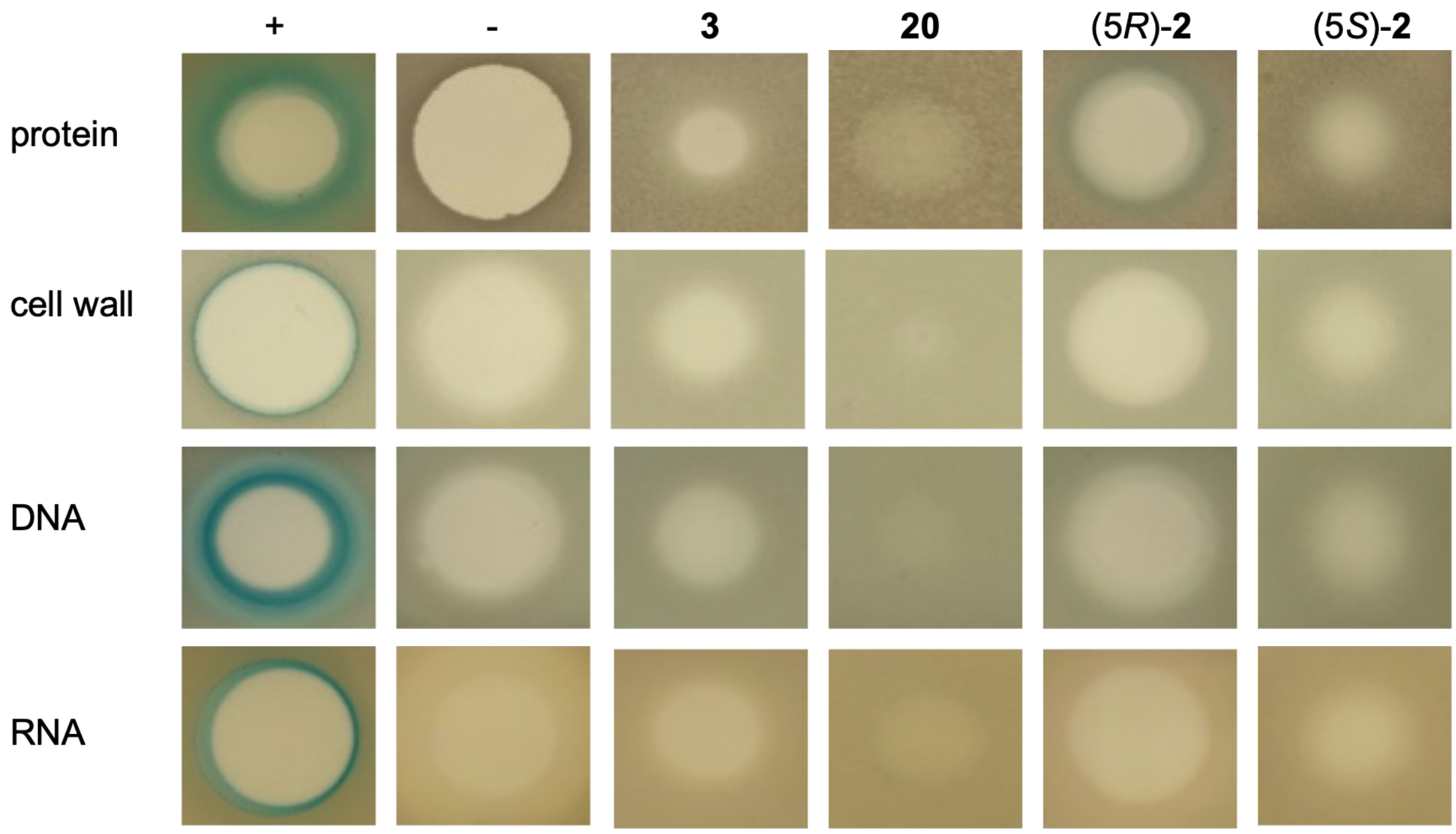

Supplementary Figure S7. **Mode of action profiling of rhabdobranin and derivatives.** Pathway-specific *B. subtilis* promoter–*lacZ* reporter strains were used to detect interference with protein biosynthesis (P_{yheI}-*lacZ*), cell-wall biosynthesis (P_{ypuA}-*lacZ*), DNA replication (P_{yorB}-*lacZ*), and RNA synthesis (P_{yvgS}-*lacZ*). Reporter induction is visualized as a blue halo at the margin of the growth inhibition zone. Rhabdobranin and derivatives were spotted at 30 µg. Clindamycin, vancomycin, ciprofloxacin, and rifampicin served as pathway-specific positive controls.

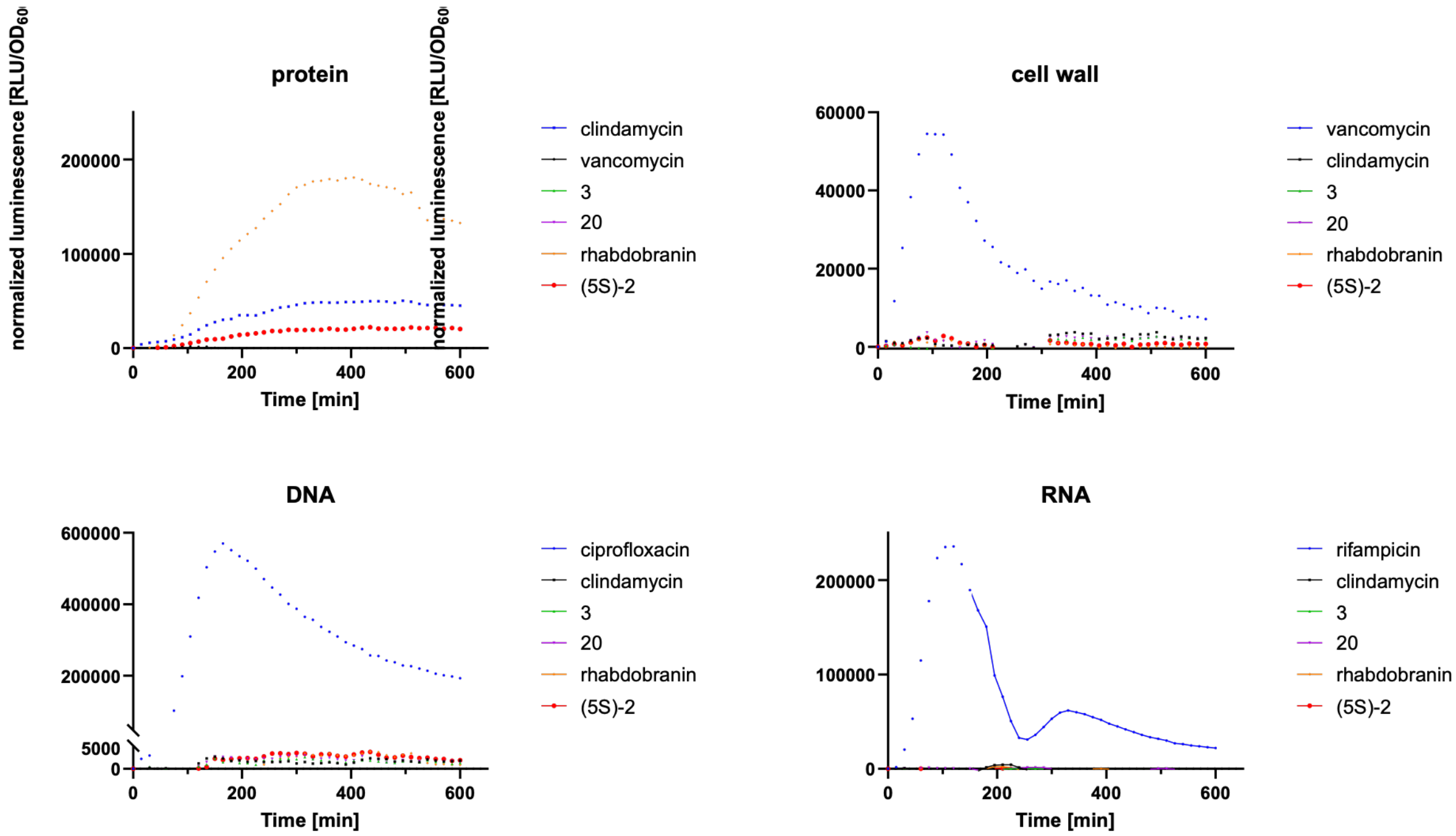

Supplementary Figure S8. Time-resolved profiling of rhabdobranin and derivatives using pathway-specific luciferase reporters. *B. subtilis* P_{yheI}-*lux* (protein biosynthesis), P_{ypuA}-*lux* (cell-wall biosynthesis), P_{yorB}-*lux* (DNA synthesis) and P_{yvgS}-*lux* (RNA synthesis) reporter strains were grown in Mueller–Hinton broth to an OD_{600} of 0.4–0.6 and exposed to serial dilutions of rhabdobranin (5*R*)-**2** and derivatives in 96-well chimney plates. Clindamycin, vancomycin, ciprofloxacin, and rifampicin served as pathway-specific controls. Luminescence and OD_{600} were monitored for 10 h using a Tecan Spark microplate reader and reporter activity is presented as luminescence normalized to OD_{600} (RLU/OD_{600}). Time courses are representative of three independent experiments.

Bacterial Strain	Resistance determinant	Relevance to the study	Source
**WHO 2024 critical-priority pathogen tier**			
Klebsiella pneumoniae			
NCTC 13439	Outbreak-associated resistant strain; VIM-1-positive (Class B metallo-β-lactamase); qnrS1-positive	One of the two screening strains; included in MIC analysis	NCTC, AMR Reference Collection
DSM 103703	Clinical rectal swab isolate; 4MRGN; OXA-48 (Class D serine carbapenemase)	One of the two screening strains; anchors the resistant *Klebsiella* phenotype	DSMZ-WHO Priority Pathogens
DSM 103707	Clinical urine isolate; 4MRGN; OXA-48 -positive	Added during MIC follow-up to broaden the resistant *Klebsiella* set	DSMZ-WHO Priority Pathogens
DSM 103711	Clinical wound swab isolate; 4MRGN; OXA-48-positive	Added during MIC follow-up to broaden the resistant *Klebsiella* set	DSMZ-WHO Priority Pathogens
Acinetobacter baumannii			
NCTC 13301	OXA-23-associated carbapenem-resistant reference strain	Represents carbapenem-resistant *A. baumannii*	NCTC, AMR Reference Collection
DSM 102929	Clinical human skin isolate; 4MRGN; NDM-1-positive (Class B metallo-β-lactamase)	Added to broaden the resistant *A. baumannii* background	DSMZ-WHO Priority Pathogens
Enterobacter cloacae complex			
NCTC 13925	Carbapenem-resistant; 3GCephRE AmpC derepressed; blaIMI-2 positive	Represents the *Enterobacter* component of the ESKAPEE panel	NCTC, AMR Reference Collection
**WHO 2024 high-priority pathogen tier**			
Pseudomonas aeruginosa			
NCTC 13437	ESBL-producing strain; VIM-10-positive; VEB-1-positive	Resistant *P. aeruginosa* comparator in the primary screen	NCTC, AMR Reference Collection
DSM 24600	Clinical tracheal secretion isolate; ESBL producer; VIM-1-positive; weak carbapenem-resistant strain	WHO-relevant strain added to broaden the resistant *P. aeruginosa* background	DSMZ
Staphylococcus aureus			
NCTC 13142	EMRSA-15; SCCmec type IV	Represents MRSA and anchors the Gram-positive pathogen arm of the screen	NCTC, AMR Reference Collection
Enterococcus faecium			
NCTC 12204	Vancomycin-resistant blood isolate; *vanA*-positive	Represents VRE and anchors the Gram-positive pathogen arm of the screen	NCTC, AMR Reference Collection
DSM 13590	Clinical rectal swab isolate; *vanA*-positive VRE	Added to broaden the VRE background	DSMZ
DSM 25697	Clinical human stool isolate;	Added to broaden the VRE background	DSMZ

	Multiple antibiotic resistances; *vanA*-positive VRE		
**Reference and comparator strains**			
Klebsiella pneumoniae			
DSM 30102 (ATCC 13882)	Susceptible type strain; type-strain-associated entry	Added to compare resistant *Klebsiella* isolates with a non-WHO-priority within-species comparator strain	DSMZ
Escherichia coli			
ATCC 25922	Gram-negative reference; Susceptible CLSI/EUCAST quality-control strain	Standard susceptible Gram-negative comparator used to anchor susceptibility under standardized broth microdilution conditions	ATCC
MG1655 (*DSM 18039*)	Isogenic comparator (wild-type); K-12 wild-type	Wild-type comparator for the TolC efflux analysis	DSMZ
MG1655ΔtolC (DSM 104619)	K-12 *tolC* deletion derivative; (efflux-deficient)	Isogenic comparator lacking the outer-membrane protein TolC	DSMZ
Serratia marcescens			
ATCC 13880	Environmental pond water isolate; Type strain; Intrinsic chromosomal AmpC β-lactamase	Opportunistic/environmental Gram-negative comparator	ATCC

Supplementary Table S1. **Bacterial strains used for antimicrobial screening and MIC profiling.** Bacterial strains used for antimicrobial screening and MIC profiling, grouped by clinical priority and comparator role. Strains are organized into three categories: WHO 2024 critical-priority pathogen groups, WHO 2024 high-priority pathogen groups, and reference/comparator strains. WHO priority assignment refers to the pathogen group and resistance phenotype defined in the WHO 2024 Bacterial Priority Pathogens List, whereas NCTC, DSMZ, and ATCC indicate the culture collection source of each strain. Documented resistance/status annotations are based on culture-collection records or strain certificates where available.

Abbreviations: AMR, antimicrobial resistance; 4MRGN, four-class multidrug-resistant Gram-negative rod, according to the German MRGN classification; 3GCephRE, third-generation cephalosporin-resistant Enterobacterales; ATCC, American Type Culture Collection; BHI, Brain Heart Infusion; BPPL, Bacterial Priority Pathogens List; DSMZ, Leibniz Institute DSMZ–German Collection of Microorganisms and Cell Cultures; ESBL, extended-spectrum β-lactamase; MIC, minimum inhibitory concentration; MRSA, methicillin-resistant *Staphylococcus aureus*; NCTC, National Collection of Type Cultures; VRE, vancomycin-resistant *Enterococcus faecium.*

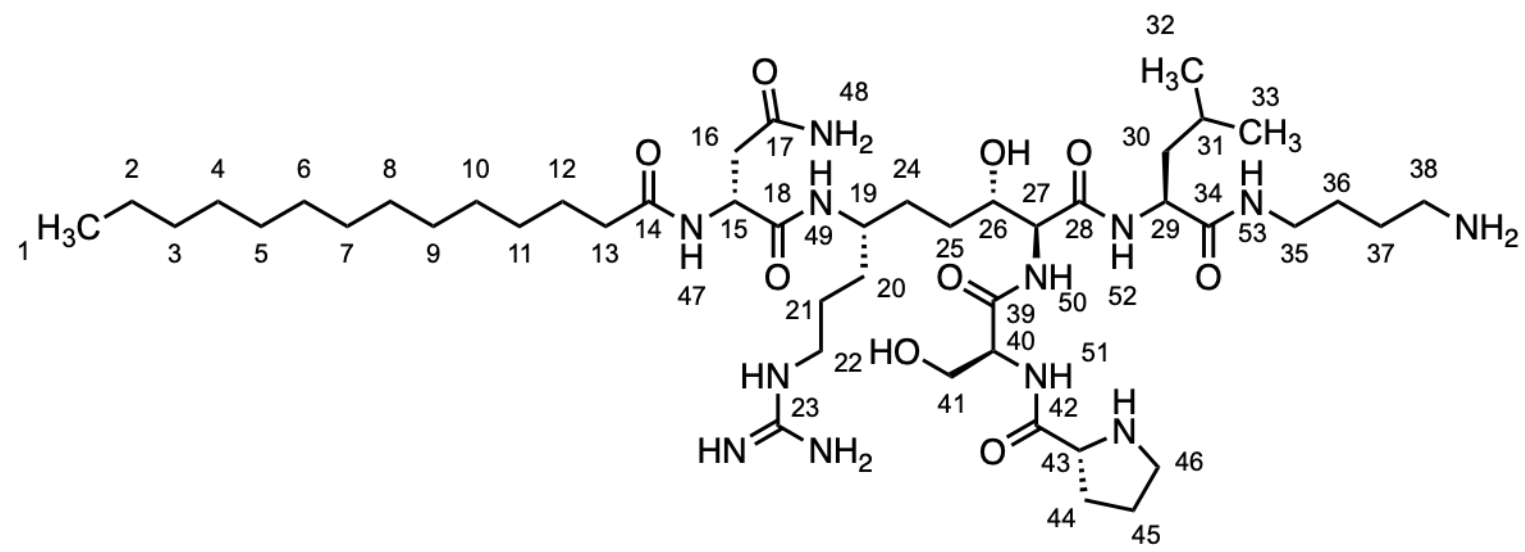

proposed pre-rhabdobranin B
(5*S*)-**1**

Position	^{1}H–NMR (mult., *J*) of *natural*	^{1}H–NMR (mult., *J*) of *synthetic* (5*S*)-(**1**)	Δ (ppm)	^{13}C–NMR of *natural*	^{13}C–NMR of *synthetic* (5*S*)-(**1**)	Δ (ppm)
1	0.84 (overlap)	0.83 (overlap)	0.01	14.5	14.4	0.1
2	1.23 (overlap)	1.23 (overlap)	–	22.6	22.6	0.0
3	1.23 (overlap)	1.23 (overlap)	–	31.9	31.9	0.0
4	1.23 (overlap)	1.23 (overlap)	–	29.2	29.2	0.0
5	1.23 (overlap)	1.23 (overlap)	–	29.8	29.8	0.0
6	1.23 (overlap)	1.23 (overlap)	–	29.6	29.5	0.1
7	1.23 (overlap)	1.23 (overlap)	–	29.6	29.5	0.1
8	1.23 (overlap)	1.23 (overlap)	–	29.6	29.5	0.1
9	1.23 (overlap)	1.23 (overlap)	–	29.5	29.4	0.1
10	1.23 (overlap)	1.23 (overlap)	–	29.4	29.3	0.1
11	1.23 (overlap)	1.23 (overlap)	–	29.2	29.2	0.0
12	1.45 (overlap)	1.54 – 1.35 (overlap)	–	25.7	25.7	0.0
13	2.09 (m)	2.09 (t, 7.6)	0.00	35.7	35.7	0.0
14	–	–	–	172.8	172.7	0.1
15	4.49 (q, 7.4)	4.49 (q, 7.2)	0.00	50.6	50.6	0.0
16	2.38 (m)*	2.38 (dd, 14.9, 8.0)	0.00	38.0	38.2	–0.2

	2.48 (overlap)	–				
17	–	–	–	172.2	172.0	0.2
18	–	–	–	171.4	171.3	0.1
19	–water	–	–	47.9	48.7	–0.8
20	1.43 (overlap)<br>1.30 (overlap)	1.54 – 1.35 (overlap)<br>1.30 (overlap)	–<br>–	31.8	31.8	0.0
21	1.45 (overlap)	1.54 – 1.35 (overlap)	–	22.6	22.6	0.0
22	3.02 (overlap)	3.03 (td, 13.6, 7.2)	–0.01	40.9	41.0	–0.1
23	–	–	–	157.8	157.9	–0.1
24	1.44 (overlap)	1.54 – 1.35 (overlap)	–	30.9	31.1	–0.2
25	1.42 (overlap)	1.54 – 1.35 (overlap)	–	29.8	29.8	0.0
26	water	–	–	70.8	71.3	–0.5
27	4.21 – 4.08 (m)	4.19 (t, 7.1)	–	58.7	58.4	0.3
28	–	–	–	170.9	170.7	0.2
29	4.21 – 4.08 (m)	4.13 (q, 8.2)	–	52.0	52.0	0.0
30	1.48 (overlap)	1.54 – 1.35 (overlap)	–	40.6	40.6	0.0
31	1.61 (overlap)	1.64 – 1.57 (m)	–	24.6	24.6	0.0
32	0.84 (overlap)	0.83 (overlap)	0.01	23.6	23.6	0.0
33	0.84 (overlap)	0.83 (overlap)	0.01	21.5	21.5	0.0
34	–	–	–	172.4	172.3	0.1
35	3.02 (m)	3.03 (td, 13.6, 7.2)	–0.01	38.4	38.4	0.0
36	1.57 (overlap)<br>1.43 (overlap)	1.64 – 1.57 (m)<br>1.54 – 1.35 (overlap)	–<br>–	26.2	26.2	0.0
37	1.48 (overlap)	1.54 – 1.35 (overlap)	–	25.1	25.3	–0.2
38	2.74 (t, 7.3)	2.74 (m)	0.00	39.0	38.9	0.1
39	–	–	–	171.1	171.0	0.1
40	4.36 (d, 6.8)	4.35 (d, 6.7)	0.01	55.0	55.0	0.0

41	water	–	–	62.5	62.4	0.1
	–water	–	–			
42	–	–	–	174.8	174.9	–0.1
43	–water	–	–	60.5	60.5	–0.1
44	1.94 (p, 9.2)<br>1.70 (m)	1.95 (q, 11.1)<br>1.69 (dt, 12.6, 6.2)	–0.01<br>0.01	30.6	30.7	–0.1
45	1.57 (overlap)<br>1.43 (overlap)	1.64 – 1.57 (m)<br>1.54 – 1.35 (overlap)	–<br>–	26.2	26.2	0.0
46	2.88 (q, 7.6)<br>2.79 (q, 5.2)	2.88 (m)<br>obscured	0.00<br>–	47.1<br>–	47.0<br>–	0.1<br>–
47	8.09 (d, 7.7)	8.09 (d, 8.1)	0.00	–	–	–
48	6.93 (s)	6.89 (s)	0.04	–	–	–
	7.43 (s)	7.42 (s)	0.01	–	–	–
49	7.66 (d, 8.7)	7.64 (d, 8.8)	0.02	–	–	–
50	8.25 – 8.17 (m)	8.27 (d, 7.4)	–	–	–	–
51	8.33 (d, 7.7)	8.34 (m)	–0.01	–	–	–
52	8.25 – 8.17 (m)	8.18 (d, 8.1)	–	–	–	–
53	7.72 (m)	7.68 (m)	0.04	–	–	–

Supplementary Table S2 Spectral data (^{1}H and ^{13}C NMR) comparison between natural and synthetic pre-rhabdobranin B (5*S*)-**1**. *Second peak at 2.50 ppm overlaps with DMSO-d_6.

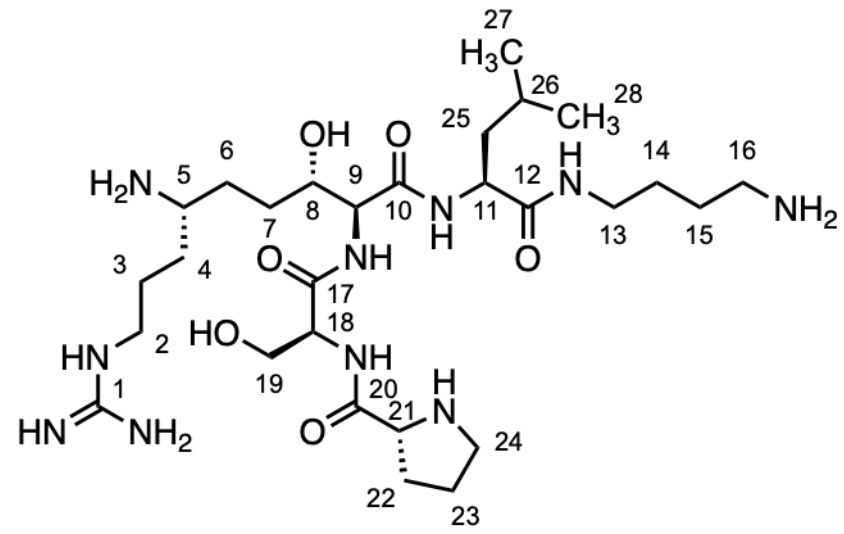

proposed rhabdobranin
(5*S*)-**2**

Position	^{1}H–NMR (mult., *J*) of *natural*	^{1}H–NMR (mult., *J*) of *synthetic* (5*S*)-(**2**)	Δ (ppm)	^{13}C–NMR of *natural*	^{13}C–NMR of *synthetic* (5*S*)-(**2**)	Δ (ppm)
1	–	–	–	156.8	156.7	0.1
2	3.15 (m)	3.08–3.20 (m)	–	40.7	40.7	0.0
3	1.60 (m)	1.72 – 1.40 (m)	–	23.8	23.8	0.0
4	1.60 (m)	1.72 – 1.40 (m)	–	29.0	28.8	0.2
5	3.26 (brs)	3.26 (brs)	0.00	51.3	51.3	0.0
6	1.74 (m)*	1.80 (m)	–0.06	28.0	28.1	–0.1
7	1.52 (m)	1.72 – 1.40 (m)	–	28.3	28.4	–0.1
8	3.89 (m)	3.89 (m)	0.00	70.2	70.2	0.0
9	4.36 (d, 6.7)	4.35 (d, 6.9)	0.01	59.8	59.8	0.0
10	–	–	–	171.4	171.4	0.0
11	4.23 (dd, 9.6, 4.7)	4.21 (dd, 9.8, 4.9)	0.02	52.7	52.7	0.0
12	–	–	–	174.2	174.2	0.0
13	3.12 (m)<br>3.19 (m)	3.08–3.20 (m)<br>3.08–3.20 (m)	–<br>–	38.6	38.6	0.0
14	1.51 (m)	1.72 – 1.40 (m)	–	25.4	25.4	0.0
15	1.60 (overlap)	1.72 – 1.40 (m)	–	23.9	23.8	0.1
16	2.93 (q, 7.5)	2.92 (t, 7.4)	0.01	39.1	39.0	0.1
17	–	–	–	171.6	171.5	0.1
18	4.45 (t, 5.4)	4.44 (t, 5.6)	0.01	55.7	55.6	0.1
19	3.80 (m)	3.79 (m)	0.01	61.1	61.1	0.0

20	–	–	–	170.2 or 170.1**	170.0	0.1 or 0.2
21	4.36 (overlap)	4.39 (m)	–0.03	58.0	58.0	0.0
22	2.40 (m)	2.41 (q, 7.6)	–0.01	29.8	29.8	0.0
23	1.99 (m)	1.99 (dd, 6.4, 3.4)	0.00	24.1	24.1	0.0
24	3.33 (m)	3.35 (dq, 12.9, 5.5)	–0.02	46.5	46.6	–0.1
25	1.55 (m)	1.72 – 1.40 (m)	–	39.7	39.7	0.0
26	1.54 (m)	1.72 – 1.40 (m)	–	24.3	24.3	0.0
27	0.80 (d, 5.6)	0.78 (d, 5.6)	0.02	20.6	20.6	0.0
28	0.86 (d, 5.5)	0.84 (d, 5.6)	0.02	22.1	22.1	0.0

Supplementary Table S3. Spectral data (^{1}H and ^{13}C NMR) comparison between natural and synthetic rhabdobranin (5*S*)-**2**. *Originally assigned as app br s by the Bode lab.[10] **The intensity of C–20 peak in the natural sample is extremely weak so the exact shift is hard to pinpoint (170.14–170.15 ppm). This affects whether the shift is rounded to 170.1 or 170.2 ppm. The natural sample was calibrated to 4.70 ppm instead of 4.79 ppm for D_2O. As such, the synthetic sample was calibrated to 4.70 ppm.

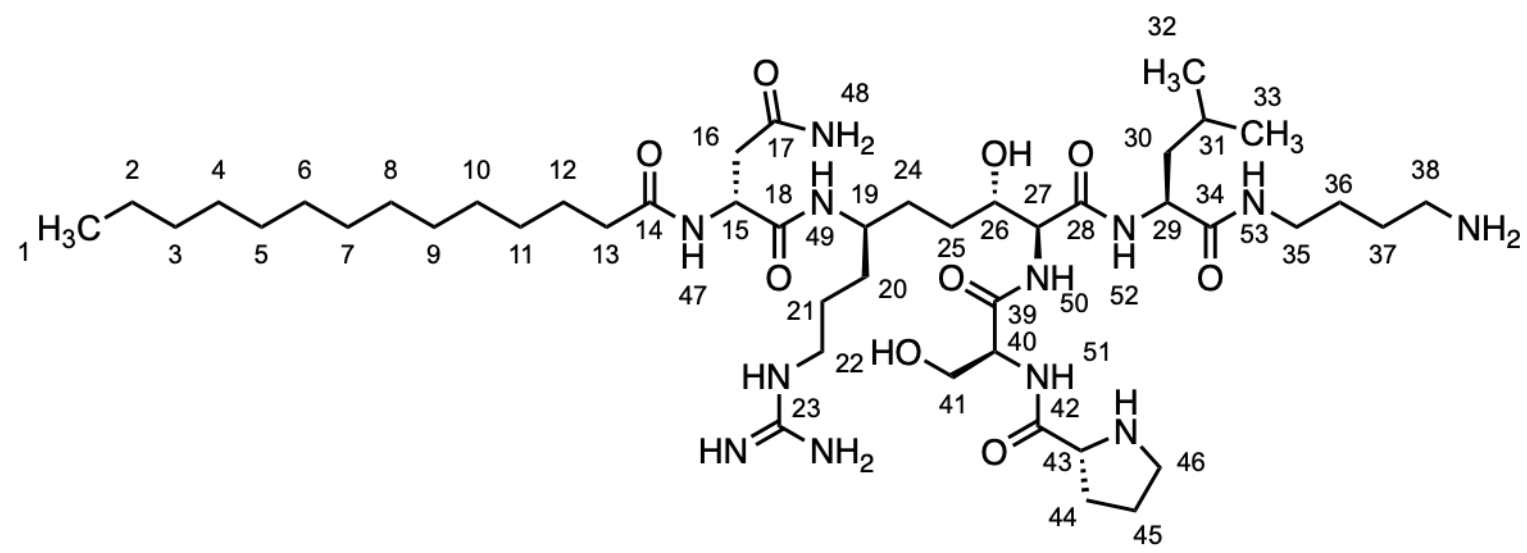

revised pre-rhabdobranin B
(5*R*)-**1**

Position	^{1}H–NMR (mult., *J*) of *natural*	^{1}H–NMR (mult., *J*) of *synthetic* (5*R*)-**1**	Δ (ppm)	^{13}C–NMR of *natural*	^{13}C–NMR of *synthetic* (5*R*)-**1**	Δ (ppm)
1	0.84 (overlap)	0.85 (overlap)	–0.01	14.5	14.4	0.1
2	1.23 (overlap)	1.23 (overlap)	–	22.6	22.6	0.0
3	1.23 (overlap)	1.23 (overlap)	–	31.9	31.9	0.0
4	1.23 (overlap)	1.23 (overlap)	–	29.2	29.2	0.0
5	1.23 (overlap)	1.23 (overlap)	–	29.8	29.8	0.0
6	1.23 (overlap)	1.23 (overlap)	–	29.6	29.5	0.1
7	1.23 (overlap)	1.23 (overlap)	–	29.6	29.5	0.1
8	1.23 (overlap)	1.23 (overlap)	–	29.6	29.5	0.1
9	1.23 (overlap)	1.23 (overlap)	–	29.5	29.4	0.1
10	1.23 (overlap)	1.23 (overlap)	–	29.4	29.3	0.1
11	1.23 (overlap)	1.23 (overlap)	–	29.2	29.1	0.1
12	1.45 (overlap)	1.54 – 1.35 (overlap)	–	25.7	25.7	0.0
13	2.09 (m)	2.09 (t, 7.4)	0.00	35.7	35.7	0.0
14	–	–	–	172.8	172.8	0.0
15	4.49 (q, 7.4)	4.49 (d, 8.7)	0.00	50.6	50.5	0.1
16	2.38 (m)*	2.38* (d, 14.4)	0.00	38.0	38.0	0.0

	2.48 (overlap)	–				
17	–	–	–	172.2	172.2	0.0
18	–	–	–	171.4	171.5	–0.1
19	–water	–	–	47.9	47.9	0.0
20	1.43 (overlap)<br>1.30 (overlap)	1.54 – 1.35 (overlap)<br>1.30 (overlap)	–<br>–	31.8	31.8	0.0
21	1.45 (overlap)	1.46 (overlap)	–0.01	22.6	22.6	0.0
22	3.02 (overlap)	3.03 (app s)	–0.01	40.9	40.9	0.0
23	–	–	–	157.8	157.7	0.1
24	1.44 (overlap)	1.54 – 1.35 (overlap)	–	30.9	30.9	0.0
25	1.42 (overlap)	1.54 – 1.35 (overlap)	–	29.8	29.8	0.0
26	water	–	–	70.8	70.7	0.1
27	4.21 – 4.08 (m)	4.14 (app s)	–	58.7	58.7	0.0
28	–	–	–	170.9	170.8	0.1
29	4.21 – 4.08 (m)	4.14 (app s)	–	52.0	52.0	0.0
30	1.48 (overlap)	1.54 – 1.35 (overlap)	–	40.6	40.5	0.1
31	1.61 (overlap)	1.64 – 1.57 (m)	–	24.6	24.6	0.0
32	0.84 (overlap)	0.85 (overlap)	–0.01	23.6	23.6	0.0
33	0.84 (overlap)	0.85 (overlap)	–0.01	21.5	21.5	0.0
34	–	–	–	172.4	172.4	0.0
35	3.02 (m)	3.03 (app s)	–0.01	38.4	38.3	0.1
36	1.57 (overlap)<br>1.43 (overlap)	1.64 – 1.57 (m)<br>1.54 – 1.35 (overlap)	–<br>–	26.2	26.2	0.0
37	1.48 (overlap)	1.54 – 1.35 (overlap)	–	25.1	25.1	0.0
38	2.74 (t, 7.3)	2.78 (app s)	–0.04	39.0	38.9	0.1
39	–	–	–	171.1	171.0	0.1
40	4.36 (d, 6.8)	4.36 (app s)	0.00	55.0	55.0	0.0
41	water	–	–	62.5	62.4	0.1

	water	–	–			
42	–	–	–	174.8	174.9	–0.1
43	water	–	–	60.5	60.4	0.1
44	1.94 (p, 9.2)<br>1.70 (m)	1.95 (app s)<br>1.72 (d, 23.0)	–0.01<br>–0.02	30.6	30.6	0.0
45	1.57 (overlap)<br>1.43 (overlap)	1.79 – 1.58 (m)<br>1.44 (app s)	–<br>–0.01	26.2	26.2	0.0
46	2.88 (q, 7.6)<br>2.79 (q, 5.2)	2.89 (app s)<br>2.78 (app s)	–0.01<br>0.01	47.1<br>–	47.0<br>–	0.1<br>–
47	8.09 (d, 7.7)	8.07 (app s)	0.02	–	–	–
48	6.93 (s)	6.92 (s)	0.01	–	–	–
	7.43 (s)	7.42 (s)	0.01	–	–	–
49	7.66 (d, 8.7)	7.63 (d, 8.4)	0.03	–	–	–
50	8.25 – 8.17 (m)	8.22 (d, 7.1)	–	–	–	–
51	8.33 (d, 7.7)	8.34 (app s)	–0.01	–	–	–
52	8.25 – 8.17 (m)	8.17 (d, 7.8)	–	–	–	–
53	7.72 (m)	7.70 (app s)	0.02	–	–	–

Supplementary Table S4. Spectral data (^{1}H– and ^{13}C NMR) comparison between natural and synthetic pre-rhabdobranin B (5*R*)-**1**.

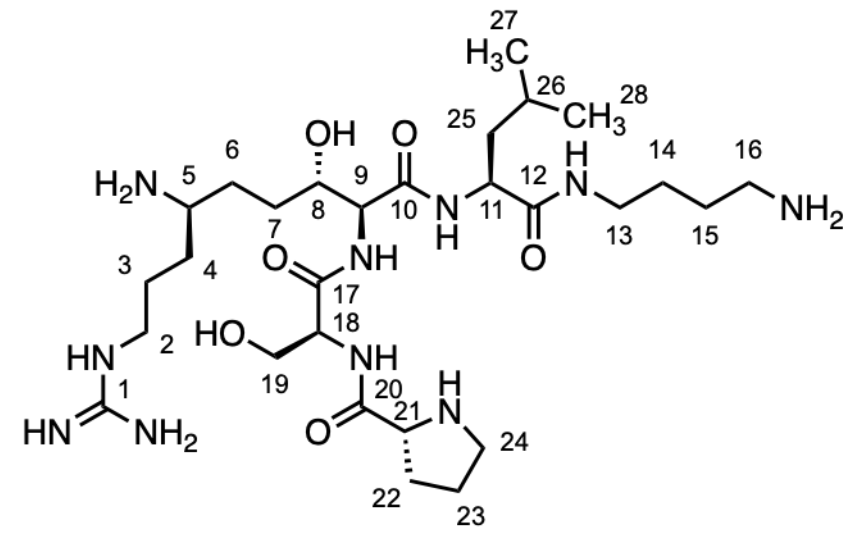

revised rhabdobranin
(5*R*)-**2**

Position	^{1}H–NMR (mult., *J*) of *natural*	^{1}H–NMR (mult., *J*) of *synthetic* (5*R*)-**2**	Δ (ppm)	^{13}C–NMR of *natural*	^{13}C–NMR of *synthetic* (5*R*)-**2**	Δ (ppm)
1	–	–	–	156.8	156.7	0.1
2	3.15 (m, 1H)	3.15 (q, 8.9 Hz)	0.00	40.7	40.6	0.1
3	1.60 (m)	1.68 – 1.47 (m)	–	23.8	23.7	0.1
4	1.60 (m)	1.68 – 1.47 (m)	–	29.0	29.0	0.0
5	3.26 (brs)	3.26 (d, 6.1)	0.00	51.3	51.3	0.0
6	1.73* (m)	1.74 (q, 6.0)	–0.01	28.0	28.0	0.0
7	1.52 (m)	1.68 – 1.47 (m)	–	28.3	28.3	0.0
8	3.89 (m)	3.89 (dd, 14.0, 7.2)	0.00	70.2	70.2	0.0
9	4.36 (d, 6.7)	4.40 (dd, 8.3, 5.9)	–0.04	59.8	59.7	0.1
10	–	–	–	171.4	171.4	0.0
11	4.23 (dd, 9.6, 4.7)	4.25 (dp, 14.6, 5.2)	–0.02	52.7	52.7	0.0
12	–	–	–	174.2	174.2	0.0
13	3.12 (m)<br>3.19 (m)	3.08–3.20 (m)<br>3.08–3.20 (m)	–<br>–	38.6	38.6	0.0
14	1.51 (m)	1.68 – 1.47 (m)	–	25.4	25.4	0.0
15	1.60 (overlap)	1.68 – 1.47 (m)	–	23.9	23.8	0.1
16	2.93 (q, 7.5)	2.93 (t, 7.5)	0.00	39.1	39.0	0.1
17	–	–	–	171.6	171.5	0.1
18	4.45 (t, 5.4)	4.46 (t, 5.6)	–0.01	55.7	55.7	0.0

19	3.80 (m)	3.84 – 3.74 (m)	–	61.1	61.1	0.0
20	–	–	–	170.2 or 170.1**	170.0	0.1 or 0.2
21	4.36 (overlap)	4.36 (d, 7.0)	0.00	58.0	58.0	0.0
22	2.40 (m)	2.41 (t, 7.9)	–0.01	29.8	29.8	0.0
23	1.99 (m)	2.07 – 1.98 (m)	–	24.1	24.1	0.0
24	3.33 (m)	3.37 (ddd, 16.8, 9.8)	–0.04	46.5	46.5	0.0
25	1.55 (m)	1.68 – 1.47 (m)	–	39.7	39.7	0.0
26	1.54 (m)	1.68 – 1.47 (m)	–	24.3	24.3	0.0
27	0.80 (d, 5.6)	0.80 (d, 5.6)	0.00	20.6	20.6	0.0
28	0.86 (d, 5.5)	0.86 (d, 5.6)	0.00	22.1	22.1	0.0

Supplementary Table S5. Spectral data (^{1}H and ^{13}C NMR) comparison between natural and synthetic rhabdobranin (5*R*)-**2**. *Originally assigned as app br s by the Bode lab.[10] **The intensity of C–20 peak in the natural sample is extremely weak so the exact shift is hard to pinpoint (170.14–170.15 ppm). This affects whether the shift is rounded to 170.1 or 170.2 ppm. The natural sample was calibrated to 4.70 ppm instead of 4.79 ppm for D_2O. As such, the synthetic sample was calibrated to 4.70 ppm.

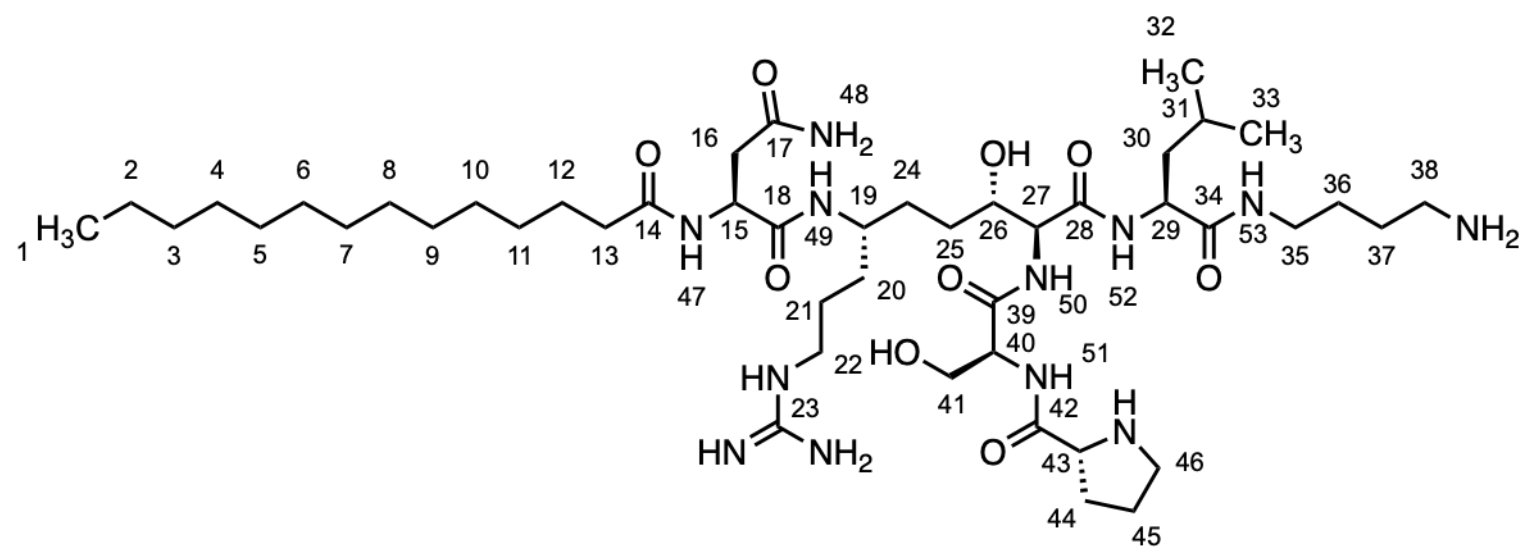

L-Asn-L-Arg pre-rhabdobranin B
S30

Position	^{1}H–NMR (mult., *J*) of *natural*	^{1}H–NMR (mult., *J*) of *synthetic* **S30**	Δ (ppm)	^{13}C–NMR of *natural*	^{13}C–NMR of *synthetic* **S30**	Δ (ppm)
1	0.84 (overlap)	0.83 (overlap)	0.01	14.5	14.4	0.1
2	1.23 (overlap)	1.23 (overlap)	–	22.6	22.6	0.0
3	1.23 (overlap)	1.23 (overlap)	–	31.9	31.8	0.1
4	1.23 (overlap)	1.23 (overlap)	–	29.2	29.2	0.0
5	1.23 (overlap)	1.23 (overlap)	–	29.8	29.9	–0.1
6	1.23 (overlap)	1.23 (overlap)	–	29.6	29.5	0.1
7	1.23 (overlap)	1.23 (overlap)	–	29.6	29.5	0.1
8	1.23 (overlap)	1.23 (overlap)	–	29.6	29.5	0.1
9	1.23 (overlap)	1.23 (overlap)	–	29.5	29.4	0.1
10	1.23 (overlap)	1.23 (overlap)	–	29.4	29.3	0.1
11	1.23 (overlap)	1.23 (overlap)	–	29.2	29.2	0.0
12	1.45 (overlap)	1.52 – 1.39 (overlap)	–	25.7	25.7	0.0
13	2.09 (m)	2.09 (t, 7.6)	0.00	35.7	35.7	0.0
14	–	–	–	172.8	172.8	0.0
15	4.49 (q, 7.4)	4.47 (q, 7.3)	0.02	50.5	50.6	–0.1
16	2.38 (m)*<br>2.48 (overlap)	2.36 (m)<br>2.44 (m)	0.02<br>0.04	38.0	38.0	0.0

17	–	–	–	172.2	172.1	0.1
18	–	–	–	171.4	171.4	0.0
19	–water	–	–	47.9	48.6	–0.7
20	1.43 (overlap)<br>1.30 (overlap)	1.52 – 1.39 (overlap)<br>1.28 (overlap)	–<br>0.02	31.8	31.8	0.0
21	1.45 (overlap)	1.52 – 1.39 (overlap)	–	22.6	22.6	0.0
22	3.02 (overlap)	3.04 (m)	–0.02	40.9	41.0	–0.1
23	–	–	–	157.8	157.8	0.0
24	1.44 (overlap)	1.52 – 1.39 (overlap)	–	30.9	31.1	–0.2
25	1.42 (overlap)	1.52 – 1.39 (overlap)	–	29.8	29.9	–0.1
26	water	–	–	70.8	71.4	–0.6
27	4.21 – 4.08 (m)	4.27 – 4.16 (m)	–	58.7	58.4	0.3
28	–	–	–	170.9	170.7	0.2
29	4.21 – 4.08 (m)	4.27 – 4.16 (m)	–	52.0	51.9	0.1
30	1.48 (overlap)	1.52 – 1.39 (overlap)	–	40.6	40.6	0.0
31	1.61 (overlap)	1.60 (m)	0.01	24.6	24.6	0.0
32	0.84 (overlap)	0.83 (overlap)	0.01	23.6	23.6	0.0
33	0.84 (overlap)	0.83 (overlap)	0.01	21.5	21.6	–0.1
34	–	–	–	172.4	172.3	0.1
35	3.02 (m)	3.04 (m)	–0.02	38.4	38.3	0.1
36	1.57 (overlap)<br>1.43 (overlap)	1.60 (m)<br>1.52 – 1.39 (overlap)	–0.03<br>–	26.2	26.2	0.0
37	1.48 (overlap)	1.52 – 1.39 (overlap)	–	25.1	25.3	–0.2
38	2.74 (t, 7.3)	2.76 – 2.68 (m)	–	39.0	38.9	0.1
39	–	–	–	171.1	171.1	0.0
40	4.36 (d, 6.8)	4.34 (d, 6.4)	0.02	55.0	55.0	0.0
41	water	–	–	62.5	62.3	0.2
	–water	–	–			

42	–	–	–	174.8	174.5	0.3
43	–water	–	–	60.5	60.4	0.1
44	1.94 (p, 9.2)<br>1.70 (m)	1.96 (app dt, 13.3, 6.1)<br>1.69 (app d, 5.9)	–0.02<br>0.01	30.6	30.7	–0.1
45	1.57 (overlap)<br>1.43 (overlap)	1.60 (m)<br>1.52 – 1.39 (overlap)	–0.03<br>–	26.2	26.2	0.0
46	2.88 (q, 7.6)<br>2.79 (q, 5.2)	2.93 – 2.85 (m)<br>2.93 – 2.85 (m)	–<br>–	47.1<br>–	47.0<br>–	0.1<br>–
47	8.09 (d, 7.7)	8.07 (dd, 7.9, 3.3)	0.02	–	–	–
48	6.93 (s)	6.88 (s)	0.05	–	–	–
	7.43 (s)	7.38 (s)	0.05	–	–	–
49	7.66 (d, 8.7)	7.65 (td, 12.4, 4.1)	0.01	–	–	–
50	8.25 – 8.17 (m)	8.22 (dd, 8.4, 4.5)	–	–	–	–
51	8.33 (d, 7.7)	8.45 (t, 9.9)	–0.12	–	–	–
52	8.25 – 8.17 (m)	8.14 (d, 7.7)	–	–	–	–
53	7.72 (m)	7.65 (td, 12.4, 4.1)	0.07	–	–	–

Supplementary Table S6. Spectral data (^{1}H and ^{13}C NMR) comparison between natural and synthetic pre-rhabdobranin B **S30**. *Second peak at 2.50 ppm overlaps with DMSO-d_6.

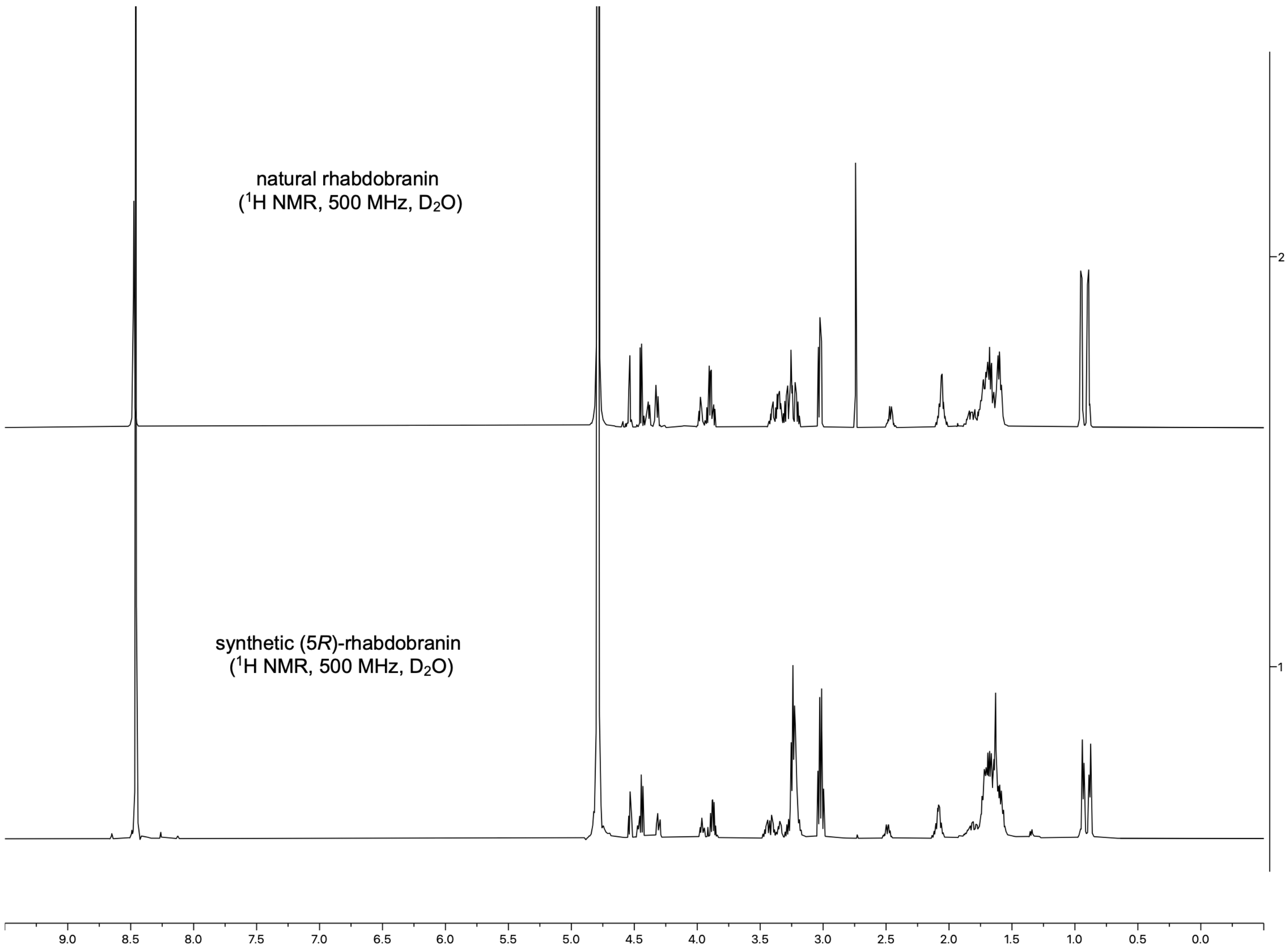

Supplementary Figure S9. Comparison of natural and synthetic (5*R*)-rhabdobranin ^{1}H NMR spectra. The natural sample contains minor co-isolated impurities arising during purification from a complex biological matrix.

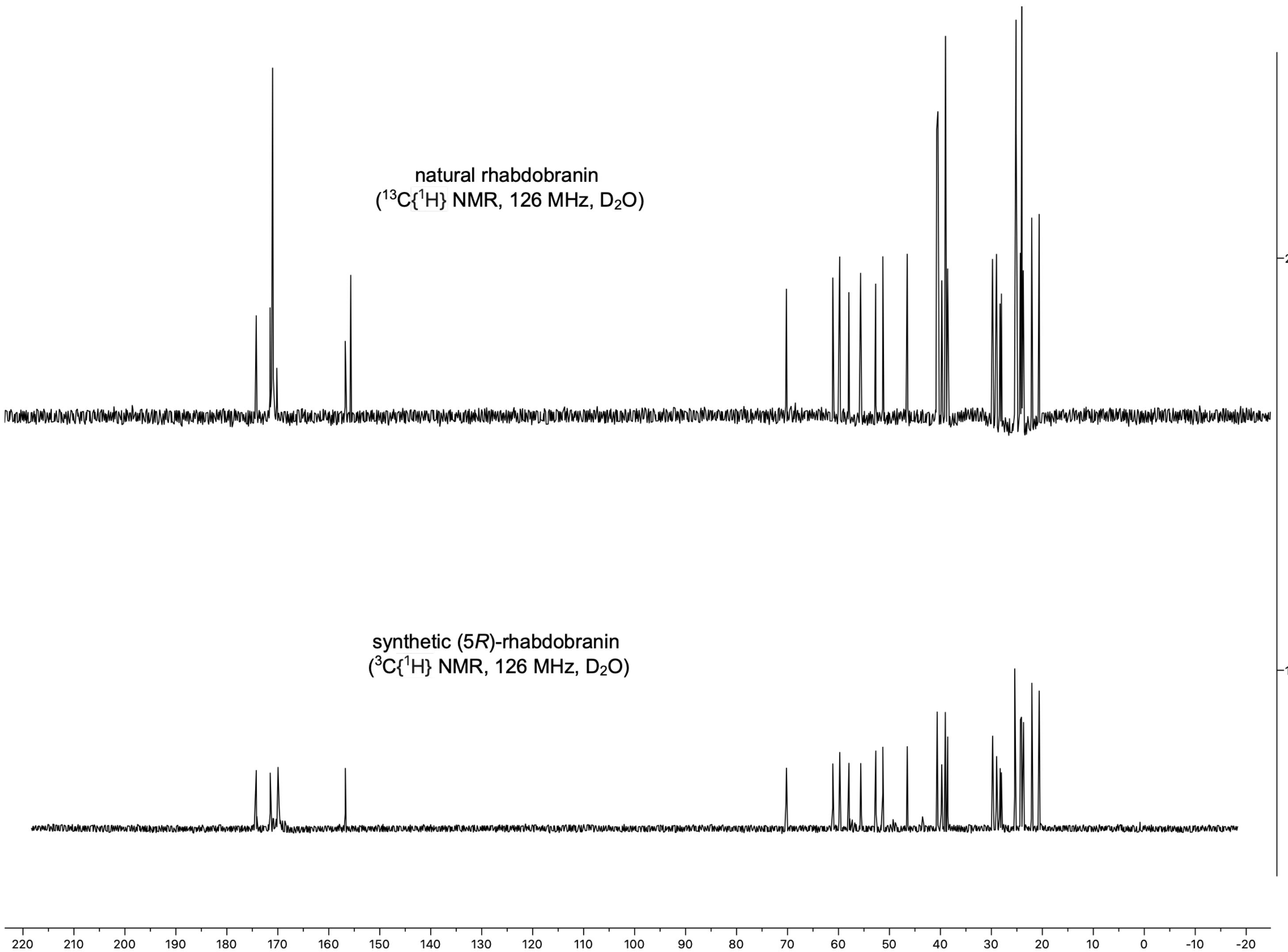

Supplementary Figure S10. Comparison of natural and synthetic (5*R*)-rhabdobranin ^{13}C NMR spectra. The natural sample contains minor co-isolated impurities arising during purification from a complex biological matrix.

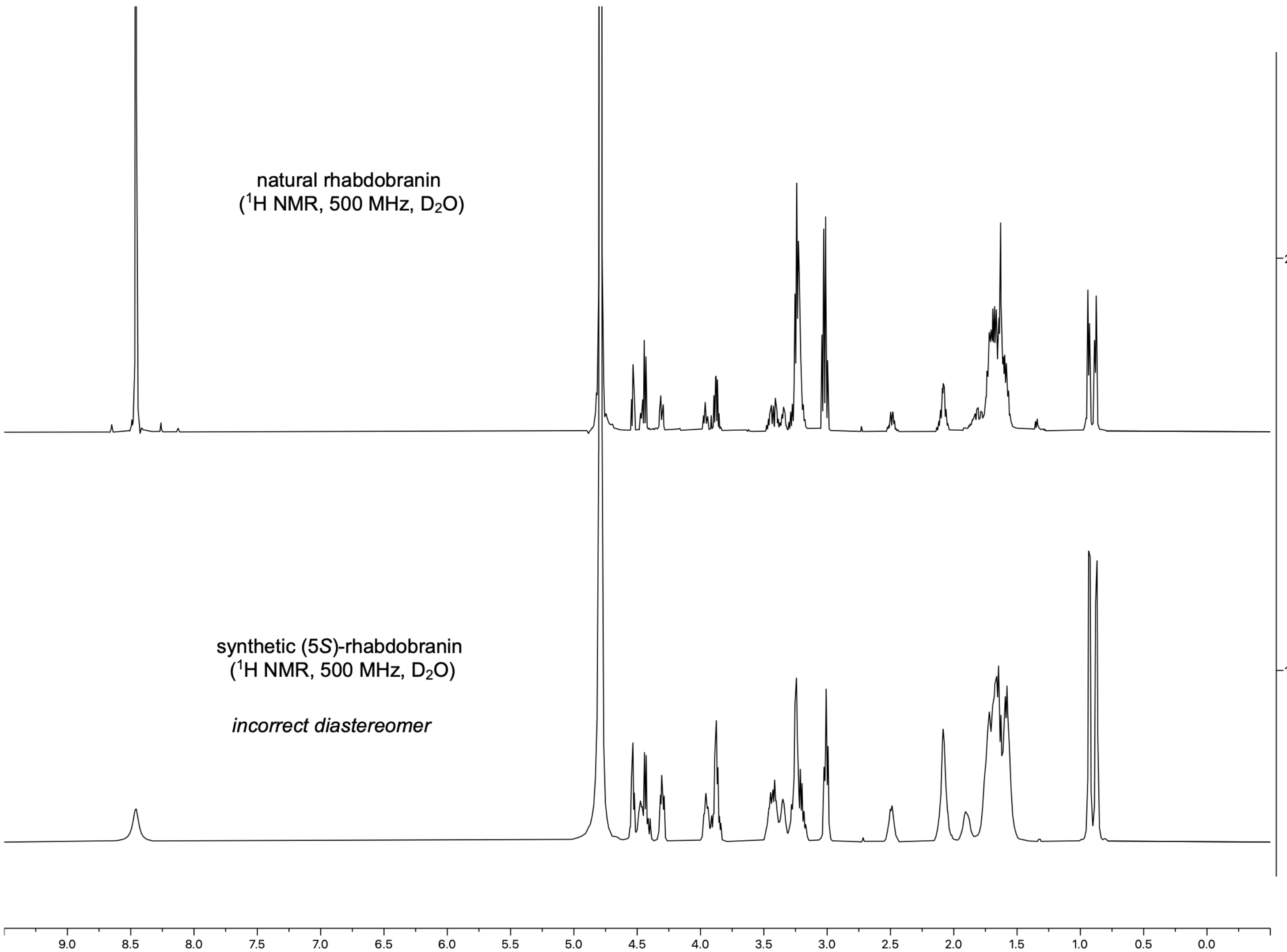

Supplementary Figure S11. Comparison of natural and synthetic (5*S*)-rhabdobranin ^{1}H NMR spectra. The natural sample contains minor co-isolated impurities arising during purification from a complex biological matrix.

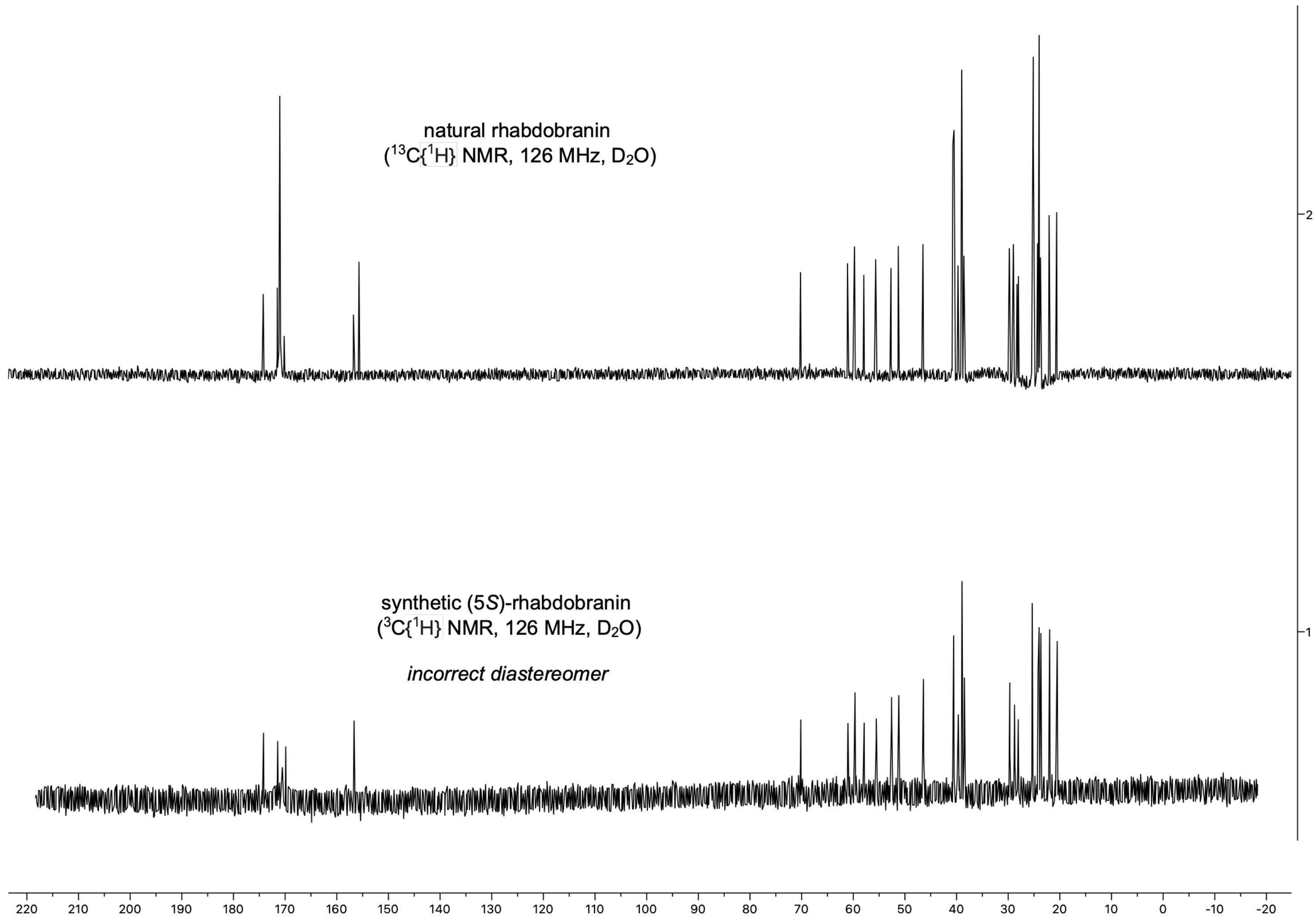

Supplementary Figure S12. Comparison of natural and synthetic (5*S*)-rhabdobranin ^{13}C NMR spectra. The natural sample contains minor co-isolated impurities arising during purification from a complex biological matrix.

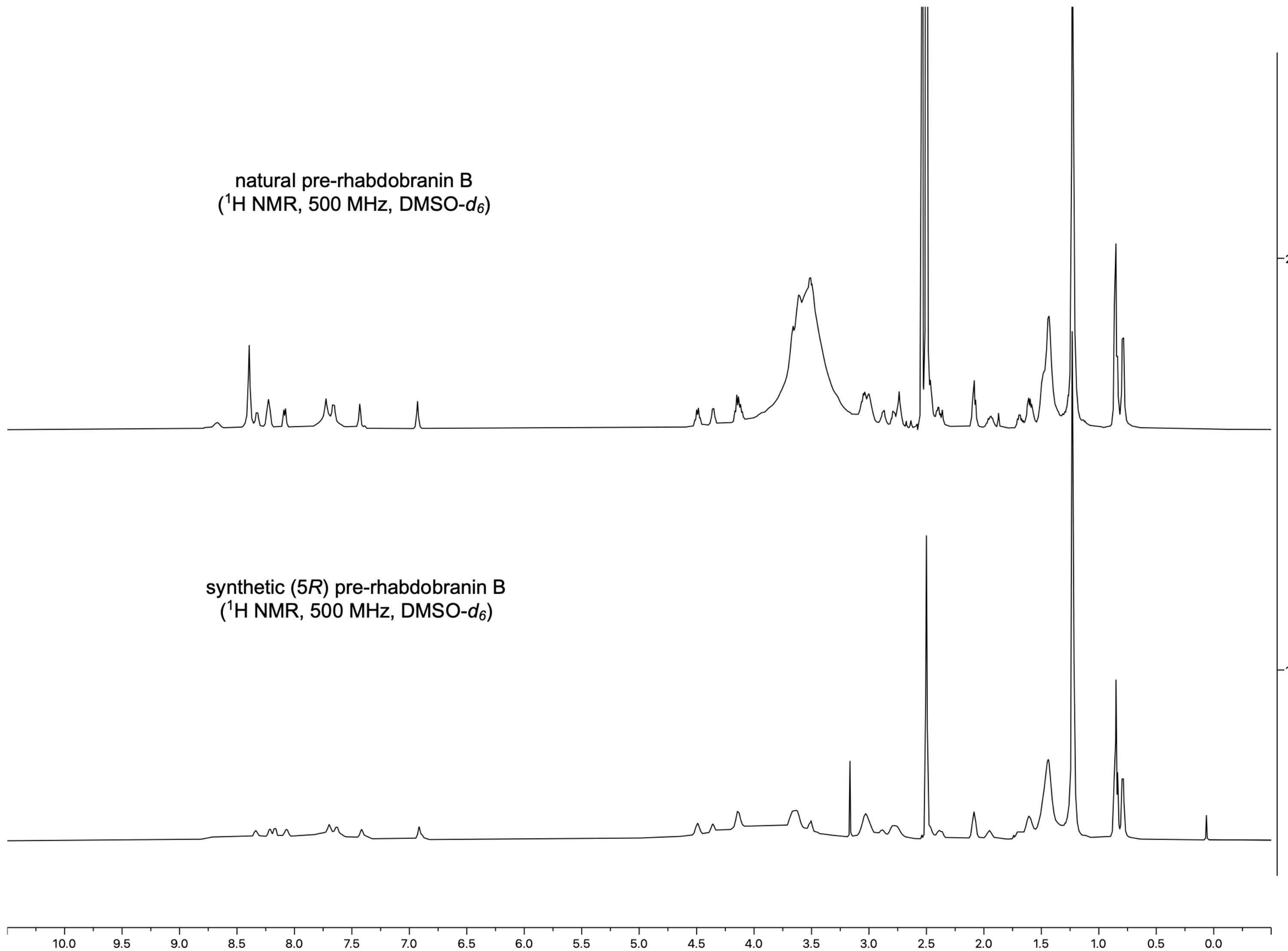

Supplementary Figure S13. Comparison of natural and synthetic (5*R*) pre-rhabdobranin B ^{1}H NMR spectra.

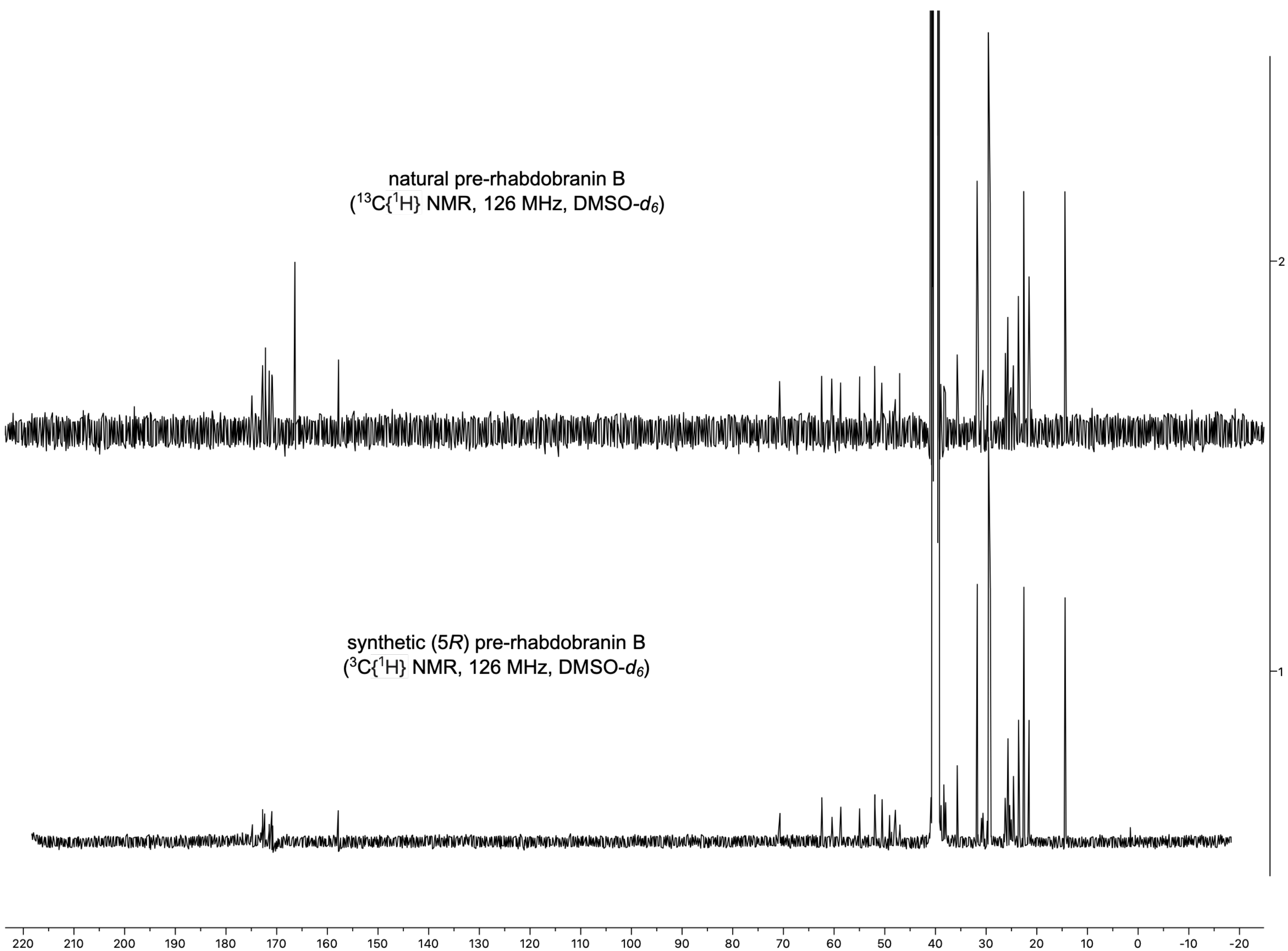

Supplementary Figure S14. Comparison of natural and synthetic (5*R*) pre-rhabdobranin B ^{13}C NMR spectra.

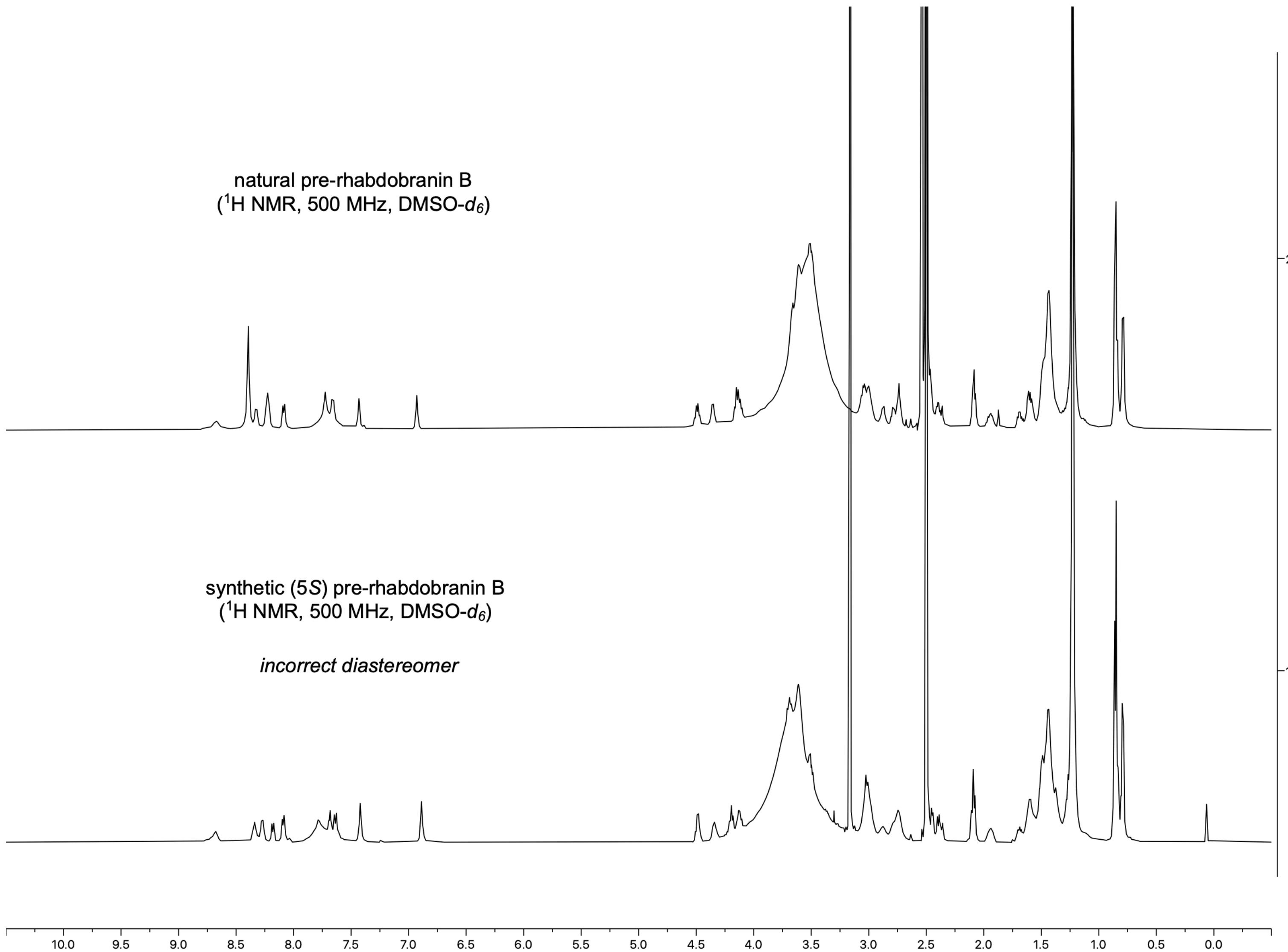

Supplementary Figure S15. Comparison of natural and synthetic (5*S*) pre-rhabdobranin B ^{1}H NMR spectra.

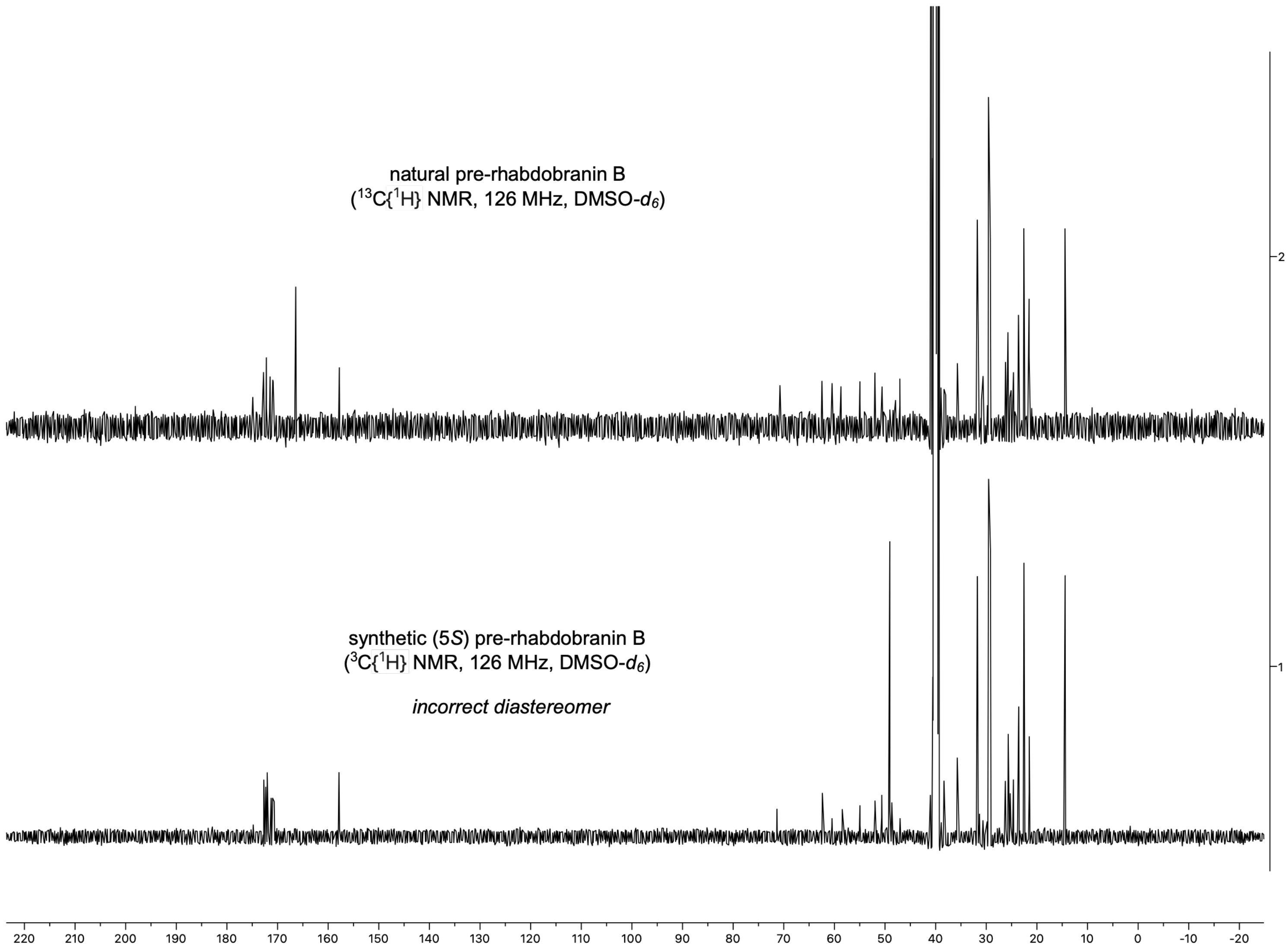

Supplementary Figure S16. Comparison of natural and synthetic (5*S*) pre-rhabdobranin B ^{13}C NMR spectra.

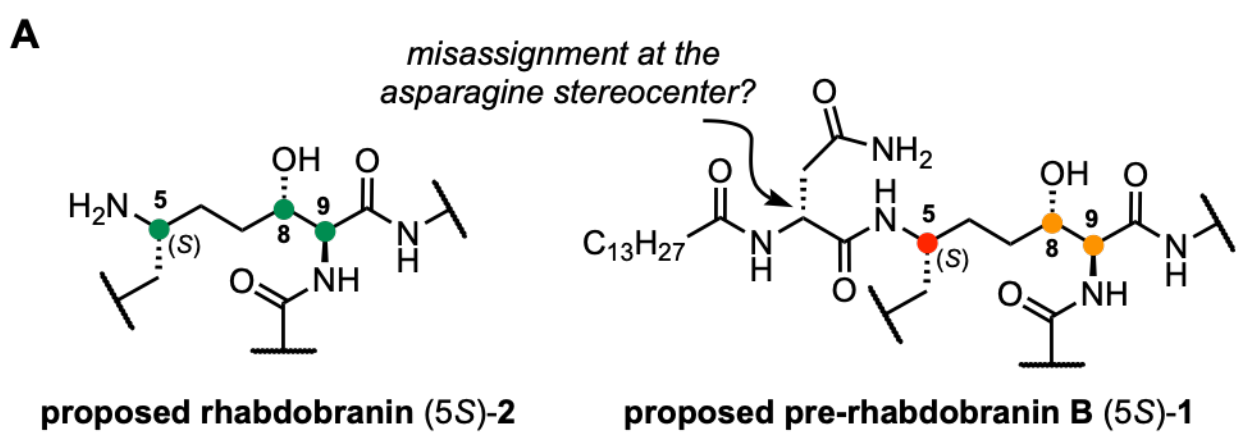

position number	natural δ_C (ppm)	synthetic (5S)-2 δ_C (ppm)	Δppm
**5**	51.3	51.3	0.0
**8**	70.2	70.2	0.0
**9**	59.8	59.8	0.0

position number	natural δ_C (ppm)	synthetic (5S)-1 δ_C (ppm)	Δppm
**5**	47.9	48.7	−0.8
**8**	70.8	71.3	−0.5
**9**	58.7	58.4	0.3

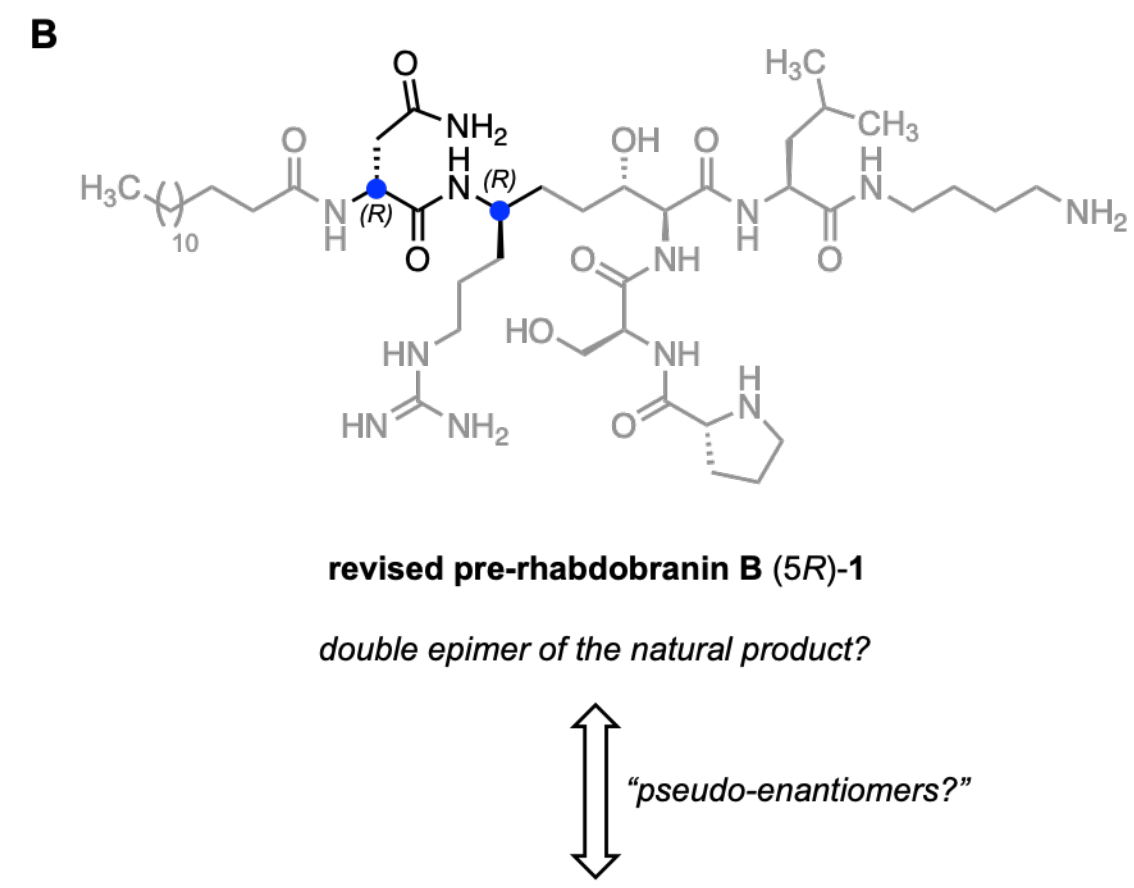

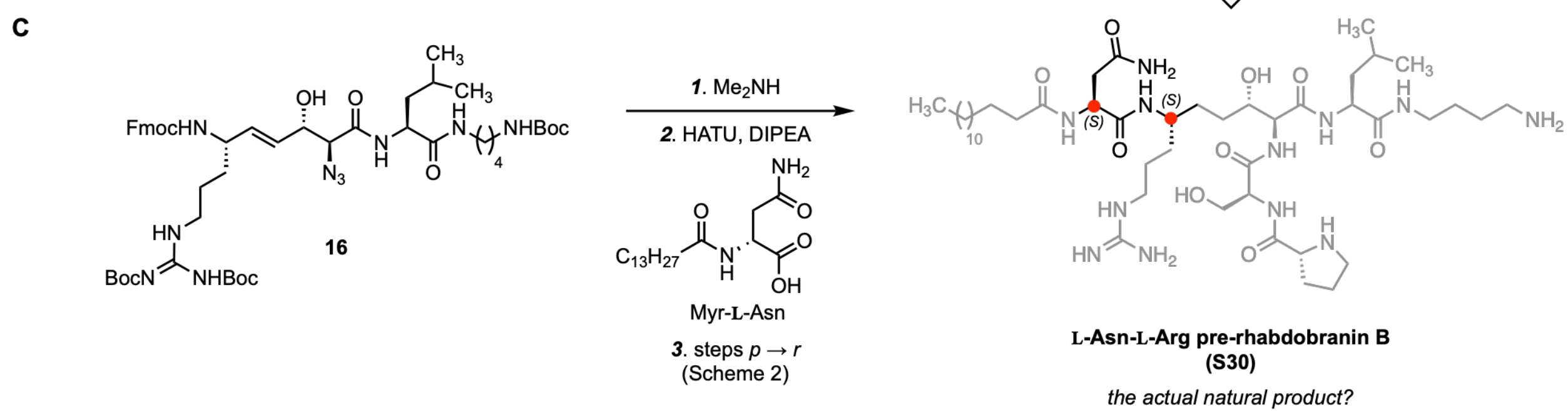

Supplementary Figure S17. **Chemical evaluation of an alternative stereochemical assignment for the Asn–Arg segment of pre-rhabdobranin B**. (**A**) An alternative explanation for the observed spectroscopic discrepancy was that the stereochemical misassignment resided at the asparagine residue rather than at arginine. Because the Myr–Asn prodrug motif is absent from mature rhabdobranin, this hypothesis would be consistent with the excellent agreement between natural and synthetic rhabdobranin, while accounting for the discrepancies that appeared only upon installation of the Myr–Asn unit in pre-rhabdobranin B. (**B**) Under the scenario proposed in (**A**), the revised synthetic structure (5*R*)-**1** containing the D-Asn/D-Arg segment would represent the double epimer of a hypothetical natural product containing L-Asn/L-Arg. Although the two structures are formally diastereomers, their Asn–Arg segments are locally enantiomeric. Given the conformational flexibility of the molecule and the spatial separation of these stereocenters from much of the remaining structure, it was conceivable that the two compounds could exhibit strikingly similar NMR spectra, potentially leading to an incorrect structural assignment. (**C**) To test this possibility directly, the proposed L-Asn/L-Arg diastereomer **S30** was synthesized from a common intermediate **16** and the opposite enantiomer of the dipeptide, namely, Myr-L-Asp. Its spectroscopic data did not match those of the natural pre-rhabdobranin B sample (See **Supplementary Table S6**), excluding the L-Asn/L-Arg assignment and providing direct chemical support for the incorporation of D-Asn in natural pre-rhabdobranin B. The experimental procedures for the preparation of **S30** is found in p. S101–S108. This chemical evidence is further reinforced by two biosynthetic observations: the Asn-incorporating NRPS–PKS module contains an epimerization domain (See **Supplementary Figure S1**), and incorporation of D-Asn is consistent with the established biosynthetic precedent for related prodrug antibiotics such as xenocoumacin.[11]

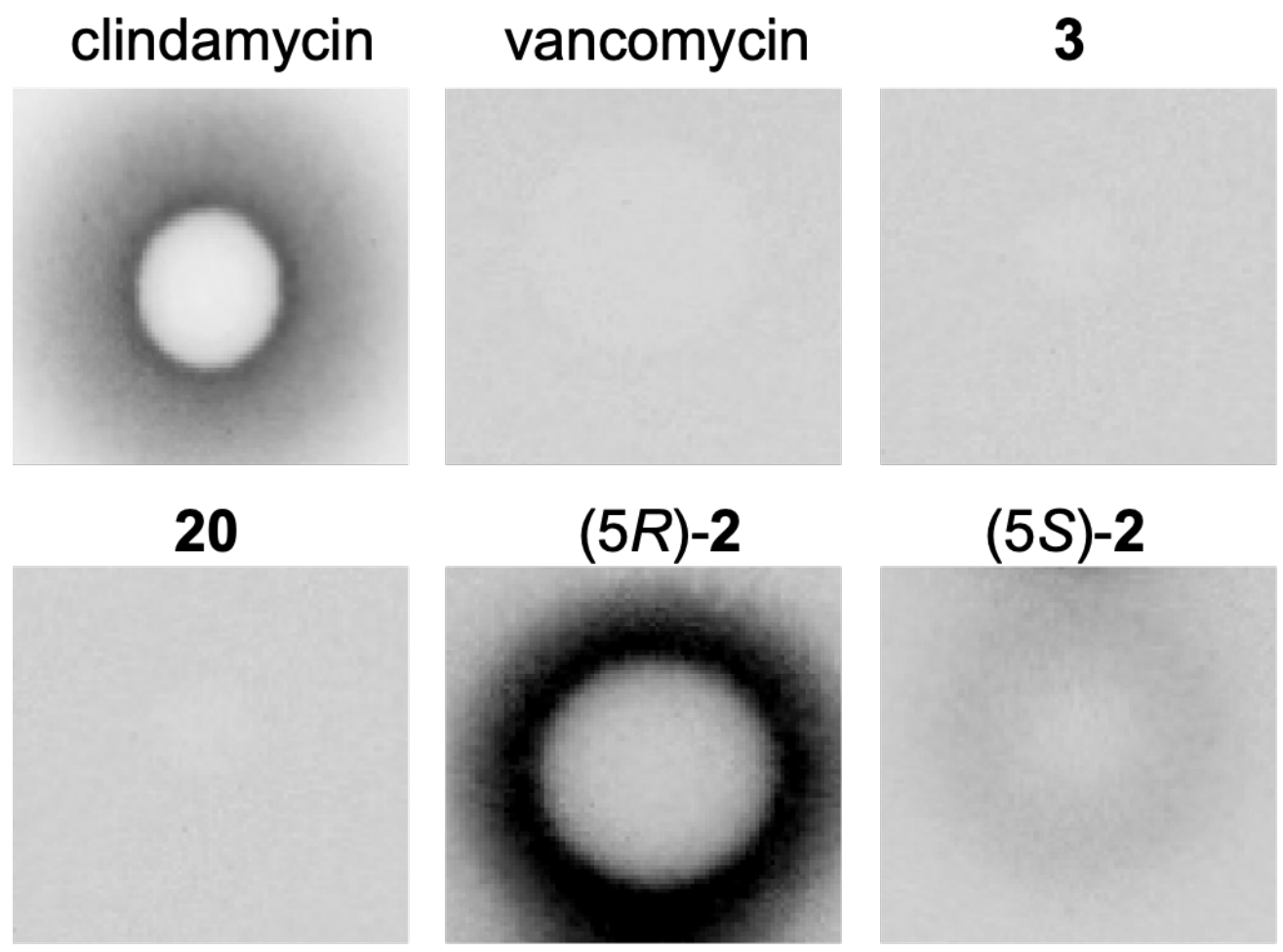

Supplementary Figure S18. Agar-based screening of rhabdobranin (5*R*)-**2** and its synthetic epimer (5*S*)-**2** along with *N*-acetyl rhabdobranin **3** and linear analogue **20** using the *Bacillus subtilis* P_{yheI}-*lux* reporter for protein-biosynthesis stress. Among the tested compounds, rhabdobranin (15 µg) specifically induced the reporter strain. Clindamycin (1.5 µg) and vancomycin served as positive and negative controls, respectively. Images are representative of three independent experiments.

Synthetic Procedures of Proposed Rhabdobranin.

*Synthesis of the Fmoc-Leu-N-Boc-Put **S3**.*

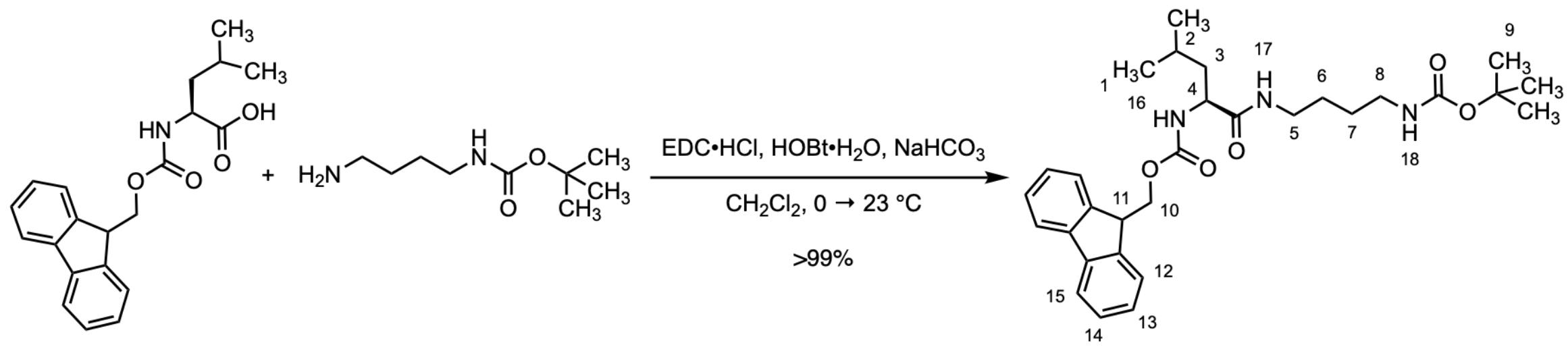

The following procedure has been adapted from the literature.[12] To a cooled (0 °C) solution of *N*-Fmoc-L-leucine **S1** (3.00 g, 8.51 mmol, 1 equiv), sodium hydrogen carbonate (0.870 g, 10.4 mmol, 1.22 equiv), 1-hydroxybenzotriazole hydrate (HOBt•H_2O, 2.23 g, 14.6 mmol, 1.72 equiv) and *N*-Boc-putrescine **S2** (1.63 g, 8.64 mmol, 1.02 equiv) in dichloromethane (42 mL, C = 0.20 M) was added 1-ethyl-3-(3-dimethylaminopropyl)carbodiimide hydrochloride (EDC•HCl, 1.99 g, 10.4 mmol, 1.22 equiv). The cooling bath was removed, and the reaction mixture was stirred for 16 h at 23 °C. The product mixture was diluted with water (100 mL). The biphasic mixture was transferred to a separatory funnel and the layers that formed were separated. The organic layer was washed with 1.0 M aqueous hydrogen chloride solution (50 mL) and a saturated aqueous sodium chloride solution (50 mL). The washed organic extract was dried over sodium sulfate. The dried solution was filtered, and the filtrate was concentrated. The residue was purified by flash column chromatography (eluting with 100% dichloromethane initially, grading to 10 % methanol–dichloromethane) to afford *Fmoc-Leu-N-Boc-putrescine* **S3** as an off-white solid (4.46 g, >99%).

R_f = 0.40 (5% methanol–dichloromethane; UV)

$[\alpha]_D^{20}$: +13.6 (*c* 1.2, $CHCl_3$)

^{1}H NMR (500 MHz, DMSO-*d6*) δ 7.89 (d, *J* = 7.6 Hz, 2H, H_{15}), 7.84 (t, *J* = 5.7 Hz, 1H, H_{17}), 7.73 (dd, *J* = 7.6, 2.8 Hz, 2H, H_{12}), 7.44 – 7.37 (m, 3H, H_{16}, 2 × H_{14}), 7.32 (td, *J* = 7.6, 3.7 Hz, 2H, H_{13}), 6.76 (t, *J* = 5.8 Hz, 1H, H_{18}), 4.33 – 4.26 (m, 1H, H_{11}), 4.25 – 4.17 (m, 2H, H_{10}), 3.98 (td, *J* = 9.1, 5.1 Hz, 1H, H_4), 3.07 – 2.95 (m, 2H, H_8), 3.08 – 2.93 (m, 2H, H_5), 2.91 – 2.84 (m, 2H, H_3), 1.64 – 1.52 (m, 1H, H_2), 1.51 – 1.42 (m, 1H, H_3), 1.41 – 1.29 (m, 14H, 1 × H_3, 2 × H_6, 2 × H_7, 9 × H_9), 0.86 (dd, *J* = 17.6, 6.6 Hz, 6H, H_1).

^{13}C NMR (126 MHz, DMSO-*d6*) δ 172.1 (C), 155.9 (C), 155.6 (C), 143.9 (C), 143.8 (C), 140.7 (2 × C), 127.6 (2 × CH), 127.0 (2 × CH), 125.3 (2 × CH), 120.1 (2 × CH), 77.3 (C), 65.5 (CH_2), 53.1 (CH), 46.7 (CH), 41.0 (CH_2), 38.3 (CH_2), 28.3 (3 × CH_3), 26.9 (CH_2), 26.4 (CH_2), 24.2 (CH), 23.0 (CH_3), 21.5 (CH_3). One carbon obscured by DMSO-*d6* (CH_2).

HRMS-CI (m/z): $[M + H]^+$ calcd for $C_{30}H_{42}N_3O_5$: 524.3119 found: 524.3133

Synthesis of the NH_2-Leu-N-Boc-Put **5**.

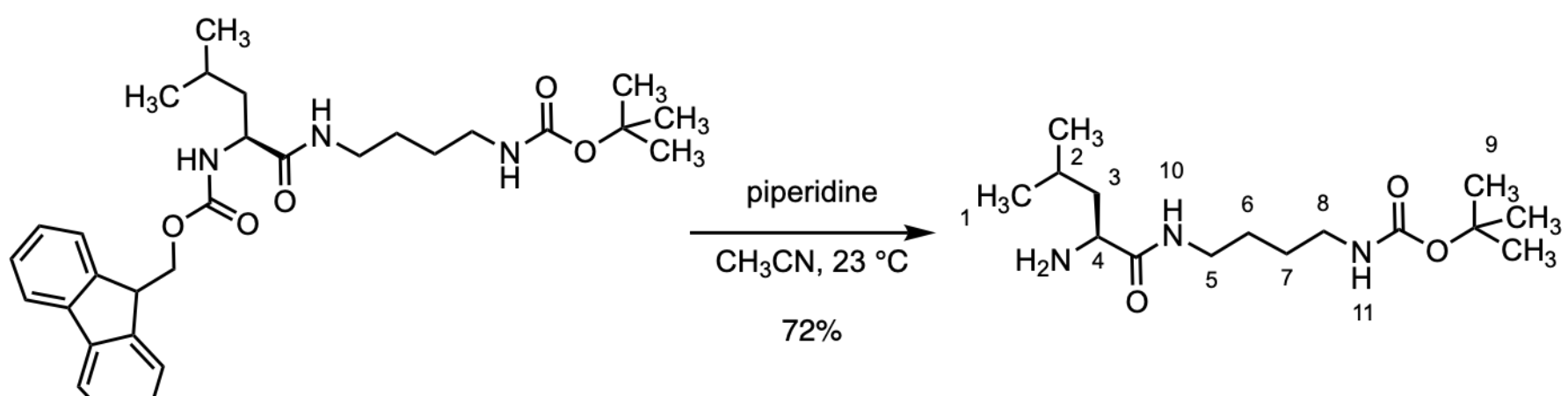

To a solution of Fmoc-leucine-*N*-Boc-putrescine **S3** (6.00 g, 11.5 mmol, 1 equiv) in acetonitrile (115 mL, C = 0.10 M) was added piperidine (4.30 mL, 43.5 mmol, 3.80 equiv) at 23 °C. The reaction mixture was stirred for 16 h at 23 °C. The product mixture was diluted with water (250 mL) and filtered. The filtrate was transferred to a separatory funnel and extracted with dichloromethane (3 × 50 mL). The combined organic extracts were dried over sodium sulfate and then filtered. The filtrate was concentrated to afford *NH_2-Leu-N-Boc-Put* **5** as a yellow oil (2.49 g, 72%). The product obtained in this way was judged to be of >95% purity (^{1}H NMR analysis) and was advanced without purification.
An analytical sample was prepared by flash column chromatography (eluting with 100 % dichloromethane initially, grading to 10 % methanol–dichloromethane + 0.25% ammonium hydroxide).

R_f = 0.32 (10% methanol–dichloromethane + 0.25% ammonium hydroxide; $KMnO_4$)

$[\alpha]_D^{20}$: –11.6 (*c* 1.1, $CHCl_3$)

^{1}H NMR (500 MHz, DMSO-*d₆*) δ 7.82 (t, *J* = 5.9 Hz, 1H, H_{10}), 6.78 (t, *J* = 5.7 Hz, 1H, H_{11}), 3.15 – 3.09 (m, 1H, H_4), 3.09 – 2.96 (m, 2H, H_5), 2.90 (app d, *J* = 6.2 Hz, 2H, H_8), 1.68 (m, 1H, H_2), 1.37 (app s, 14H, 1 × H_3, 2 × H_6, 2 × H_7, 9 × H_9), 1.26 – 1.16 (m, 1H, 1× H_3), 0.86 (dd, *J* = 16.3, 6.6 Hz, 6H, H_1).

^{13}C NMR (126 MHz, DMSO-d_6) δ 175.3 (C), 155.6 (C), 77.3 (C), 53.1 (CH), 44.4 (CH_2), 38.0 (CH_2), 28.3 (3 × CH_3), 27.0 (CH_2), 26.6 (CH_2), 24.1 (CH), 23.2 (CH_3), 21.9 (CH_3).
One carbon obscured by DMSO-d_6 (CH_2).

HRMS-CI (*m/z*): $[M + Na]^+$ calcd for $C_{15}H_{31}N_3O_3Na$: 324.2258 found: 324.2270

*Synthesis of the N-Boc-*D*-Pro-Ser-OMe* ***S6***.

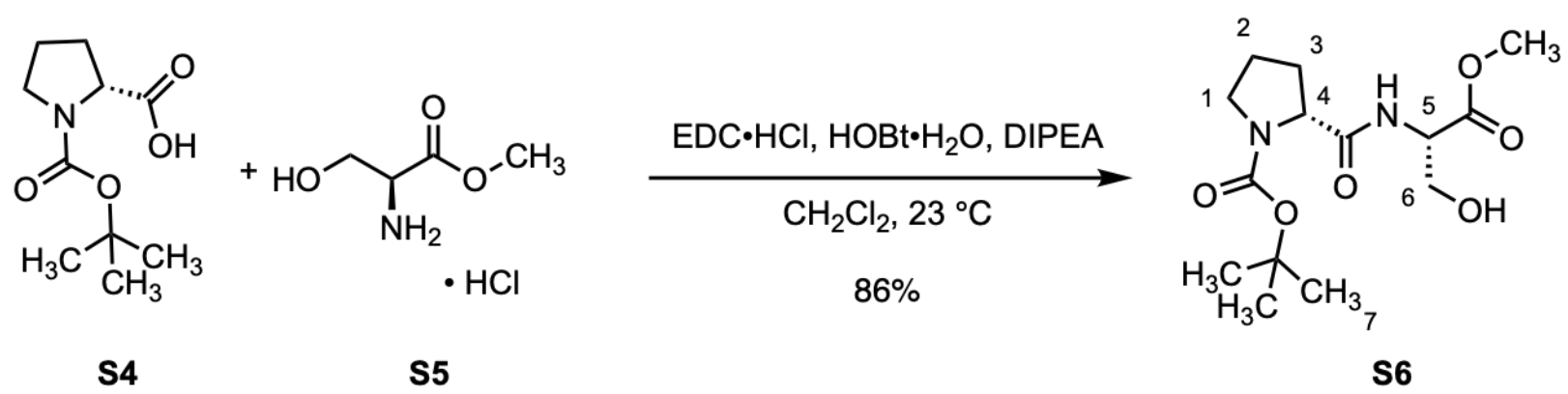

The following procedure has been adapted from the literature.[13] To a solution of *N*-Boc-D-proline **S4** (1.00 g, 4.65 mmol, 1 equiv), L-serine methyl ester hydrochloride **S5** (0.800 g, 5.11 mmol, 1.10 equiv), and 1-hydroxybenzotriazole hydrate (HOBt•H_2O, 1.07 g, 7.00 mmol, 1.50 equiv) in dichloromethane (40 mL, C = 0.12 M), were added *N*,*N*-diisopropylethylamine (DIPEA, 2.10 mL, 12.1 mmol, 2.60 equiv), followed by 1-ethyl-3-(3-dimethylaminopropyl)carbodiimide hydrochloride (EDC•HCl, 1.34 g, 7.00 mmol, 1.50 equiv) at 23 °C. The reaction mixture was stirred for 14 h at 23 °C. The product mixture was diluted with water (40 mL). The diluted product mixture was transferred to a separatory funnel and the layers that formed were separated. The organic layer was washed with 1.0 M aqueous hydrogen chloride solution (30 mL) and a saturated aqueous solution of sodium bicarbonate (30 mL). The washed organic layer was dried over sodium sulfate. The dried solution was filtered, and the filtrate was concentrated. The residue was purified by flash column chromatography (eluting with 100% dichloromethane initially, grading to 10 % methanol–dichloromethane) to afford *N-Boc-D-Pro-Ser-OMe* **S6** as a pale-yellow oil (1.30 g, 86%).

Note: For compounds containing the Boc-Pro-Ser fragment, several NMR resonances were broad or partially resolved. These features are consistent with slow conformational exchange of Boc amino acid rotamers on the NMR timescale; therefore, overlapping or broadened resonances are reported as observed without assigning individual rotameric species.[14]

R_f = 0.38 (5% methanol–dichloromethane; $KMnO_4$)

$[\alpha]_D^{20}$: +92.7 (*c* 0.91, $CHCl_3$)

^{1}H NMR (500 MHz, DMSO-d_6) major rotamer described: δ 8.04 (d, *J* = 7.9 Hz, 1H, –N*H*), 5.04 (app q, *J* = 7.2 Hz, 1H, –O*H*), 4.36 (dt, *J* = 7.9, 5.1 Hz, 1H, H_5), 4.14 (dd, *J* = 8.6, 3.2 Hz, 1H, H_4), 3.66 (ddd, *J* = 11.6, 6.1, 5.1 Hz, 2H, H_6), 3.62 (s, 3H, H_8), 3.39 – 3.23 (m, 2H, H_1), 2.15 – 1.95 (m, 1H, 1 × H_3), 1.86 – 1.68 (m, 3H, 2 × H_2, 1 × H_3), 1.33 (s, 9H, H_7).

^{13}C NMR (126 MHz, DMSO-d_6) δ 172.6 (C), 171.0 (C), 153.4 (C), 78.5 (C), 61.4 (CH_2), 59.4 (CH), 54.5 (CH), 51.8 (CH_3), 46.5 (CH_2), 31.1 (CH_2), 27.9 (3 × CH_3), 23.1 (CH_2).

HRMS-CI (m/z): $[M + Na]^+$ calcd for $C_{14}H_{24}N_2O_6Na$: 339.1527; found: 339.1536

Synthesis of the Boc-D-Pro-Ser-OH **6**.

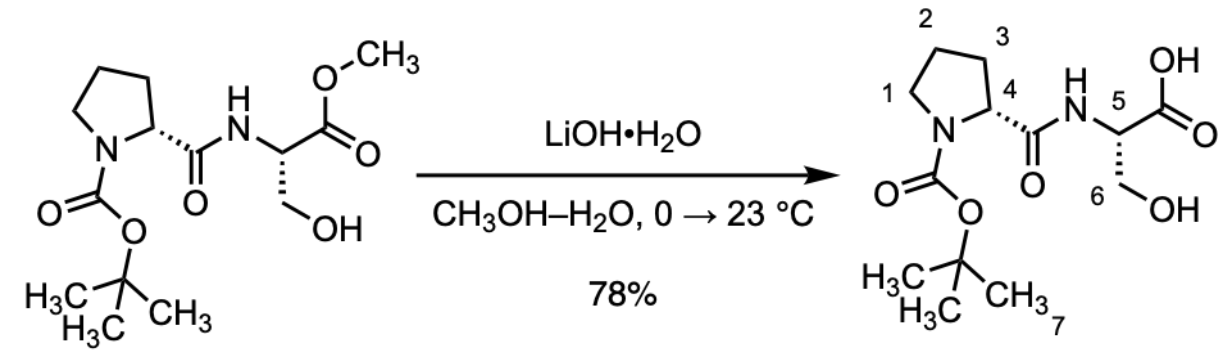

S6 6

To a cooled (0 °C) solution of *N*-Boc-D-Pro-Ser-OMe **S6** (2.16 g, 6.83 mmol, 1 equiv) in methanol (14 mL, C = 0.50 M), was added 1.0 M aqueous lithium hydroxide solution (8.50 mL, 8.53 mmol, 1.25 equiv). The cooling bath was removed, and the reaction mixture was stirred for 12 h at 23 °C. The product mixture was quenched with 1.0 M aqueous hydrogen chloride solution (20 mL). The quenched product mixture was transferred to a separatory funnel and the layers that formed were separated. The aqueous phase was extracted with ethyl acetate (3 × 15 mL). The combined organic extracts were dried over sodium sulfate. The dried solution was filtered, and the filtrate was concentrated. The residue was purified by flash column chromatography (eluting with 100% dichloromethane initially, grading to 20 % methanol–dichloromethane) to afford *Boc-D-Pro-Ser-OH* **6** as a hygroscopic and sticky white foam (1.61 g, 78%).

Note: For compounds containing the Boc-Pro-Ser fragment, several NMR resonances were broad or partially resolved. These features are consistent with slow conformational exchange of Boc amino acid rotamers on the NMR timescale; therefore, overlapping or broadened resonances are reported as observed without assigning individual rotameric species.[14]

R_f = 0.45 (10% methanol–dichloromethane; $KMnO_4$)

$[\alpha]_D^{20}$: +75.7 (*c* 0.46, $CHCl_3$)

^{1}H NMR (500 MHz, DMSO-d_6) major rotamer described: δ 12.55 (s, 1H, –COO*H*), 7.83 (d, *J* = 8.4 Hz, 1H, –N*H*), 4.28 (dt, *J* = 8.4, 4.8 Hz, 1H, H_5), 4.23 – 4.10 (m, 1H, H_4), 3.75 – 3.54 (m, 2H, H_6), 3.39 – 3.22 (m, 2H, H_1), 2.13 – 1.96 (m, 1H, H_3), 1.88 – 1.68 (m, 3H, 2 × H_2, 1 × H_3), 1.33 (s, 9H, H_7).

^{13}C NMR (126 MHz, DMSO-d_6) δ 172.3 (C), 171.9 (C), 153.4 (C), 78.6 (C), 61.6 (CH_2), 59.6 (CH), 54.4 (CH), 46.5 (CH_2), 31.0 (CH_2), 28.1 (3 × CH_3), 23.1 (CH_2).

HRMS-CI (*m/z*): $[M + Na]^+$ calcd for $C_{13}H_{22}N_2O_6Na$: 325.1370; found: 325.1383

*Synthesis of the Myr-D-Asn **D**.*

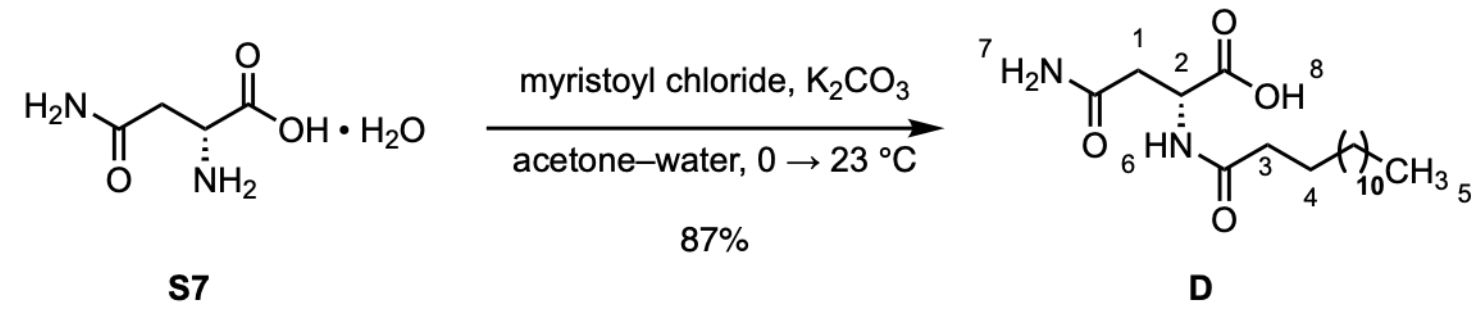

The following procedure has been adapted from the literature.[15] To a cooled (0 °C) solution of D-asparagine monohydrate **S7** (1.50 g, 12.2 mmol, 2.00 equiv) in acetone/water (60 mL, 1:1, *v*/*v*, C = 0.10 M) was added potassium carbonate (2.52 g, 18.2 mmol, 3.00 equiv). Myristoyl chloride (1.70 mL, 6.08 mmol, 1 equiv) was added dropwise at 0 °C. The cooling bath was removed, and the reaction mixture was stirred for 16 h at 23 °C. The product mixture was acidified with 1.0 M hydrochloric acid until pH 1–2 and concentrated. The resulting precipitate was filtered and washed with dichloromethane (3 × 10 mL). The residue was dried under high vacuum to afford *Myr-D-Asn peptide* **D** as a white powder (1.80 g, 87%). The product obtained in this way was judged to be of >95% purity (^{1}H NMR analysis) and was advanced without purification. The spectroscopic data are in agreement with the literature.[16]

^{1}H NMR (500 MHz, DMSO-*d$_6$*) δ 12.43 (br s, 1H, H_8), 7.98 (d, *J* = 7.9 Hz, 1H, H_7), 7.34 (s, 1H, 1 × H_7), 6.87 (s, 1 × H_7), 4.47 (app q, *J* = 6.9 Hz, 1H, H_2), 2.55 – 2.51 (m, 1H, 1 × H_1), 2.43 (dd, *J* = 15.5, 7.3 Hz, 1H, 1 × H_1), 2.07 (t, *J* = 7.4 Hz, 2H, H_3), 1.49 – 1.42 (m, 2H, H_4), 1.31 – 1.14 (m, 20H, 10 × CH_2, myristoyl), 0.85 (t, *J* = 6.8 Hz, 3H, H_5).

^{13}C NMR (126 MHz, DMSO-*d$_6$*) δ 173.0 (C), 172.0 (C), 171.2 (C), 48.7 (CH), 36.8 (CH_2), 35.1 (CH_2), 31.3 (CH_2), 29.1 (4 × CH_2), 29.0 (CH_2), 28.8 (CH_2), 28.7 (CH_2), 28.6 (CH_2), 25.2 (CH_2), 22.1 (CH_2), 14.0 (CH_3).

*Synthesis of the Fmoc-Boc(Orn)-OEt **10**.*

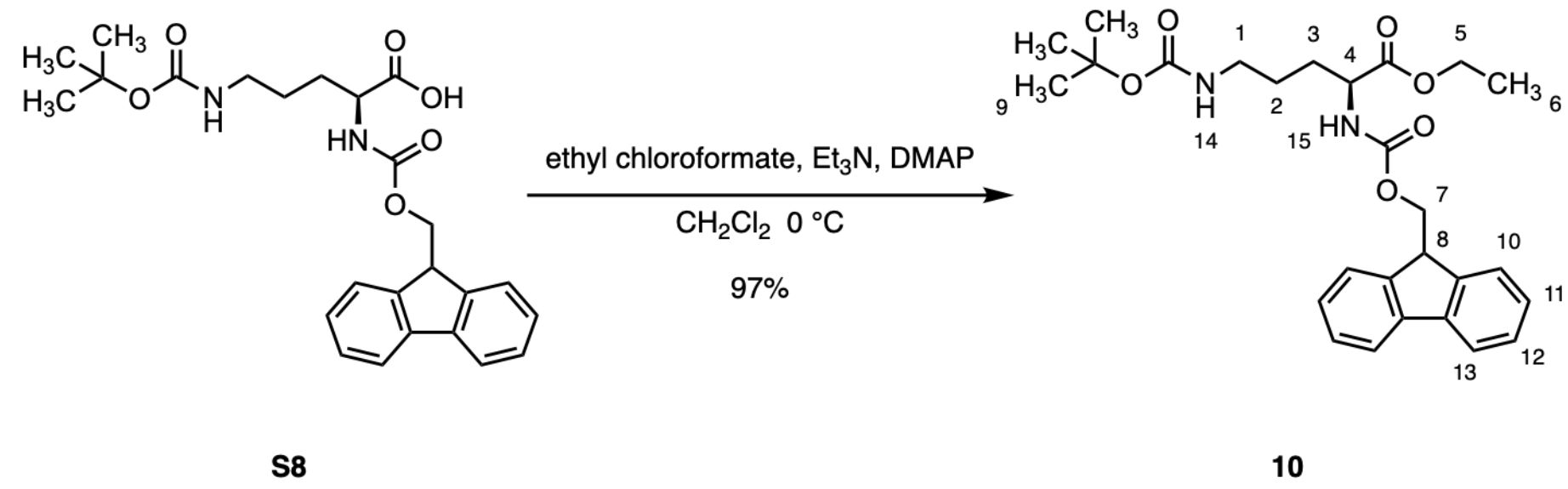

The following procedure has been adapted from the literature.[17] To a cooled (0 °C) solution of Fmoc-Boc(Orn)-OH **S8** (30.0 g, 66.0 mmol, 1 equiv) in dichloromethane (200 mL, C = 0.33 M) were added triethylamine (10.1 mL, 72.6 mmol, 1.10 equiv) and ethyl chloroformate (6.60 mL, 69.3 mmol, 1.05 equiv). The reaction mixture was stirred for 10 min at 0 °C. 4-dimethylaminopyridine (DMAP, 806 mg, 6.60 mmol, 10 mol %) was added in one portion (*mild effervescence observed*) and the reaction mixture was stirred for 4 h at 0 °C. The product mixture was diluted with diethyl ether (300 mL) and transferred to a separatory funnel. The diluted product mixture was washed sequentially with a 1.0 M aqueous hydrogen chloride solution (250 mL), a saturated aqueous sodium bicarbonate solution (100 mL), and a saturated aqueous sodium chloride solution (250 mL). The washed organic layer was dried over sodium sulfate. The dried organic layer was filtered, and the filtrate was concentrated to afford *Fmoc-Boc(Orn)-OEt* **10** as a white solid (30.8 g, 97%). The product obtained in this way was judged to be of >95% purity (^{1}H NMR analysis) and was advanced without purification.

R_f = 0.40 (30% ethyl acetate–hexane; UV)

$[\alpha]_D^{20}$: +6.5 (*c* 1.4, $CHCl_3$)

^{1}H NMR (500 MHz, DMSO-*d*$_6$) δ 7.89 (d, *J* = 7.5 Hz, 2H, H_{13}), 7.75 (d, *J* = 7.8 Hz, 1H, H_{15}), 7.72 (dd, *J* = 7.6, 2.9 Hz, 2H, H_{10}), 7.42 (t, *J* = 7.5 Hz, 2H, H_{12}), 7.33 (t, *J* = 7.4 Hz, 2H, H_{11}), 6.79 (t, *J* = 5.7 Hz, 1H, H_{14}), 4.35 – 4.25 (m, 2H, H_7), 4.22 (t, *J* = 7.1 Hz, 1H, H_8), 4.08 (p, *J* = 6.9 Hz, 2H, H_5), 3.98 (td, *J* = 8.6, 4.9 Hz, 1H, H_4), 2.91 (q, *J* = 6.8 Hz, 2H, H_1), 1.68 (p, *J* = 6.5 Hz, 1H, H_3), 1.58 (qd, *J* = 9.4, 4.9 Hz, 1H, H_3), 1.51 – 1.40 (m, 2H, H_2), 1.37 (s, 9H, H_9), 1.16 (t, *J* = 7.1 Hz, 3H, H_6).

^{13}C NMR (126 MHz, DMSO-*d*$_6$) δ 172.4 (C), 156.1 (C), 155.6 (C), 143.7 (2 × C), 140.7 (2 × C), 127.6 (2 × CH), 127.1 (2 × CH), 125.2 (2 × CH), 120.1 (2 × CH), 77.4 (C), 65.6 (CH_2), 60.4 (CH_2), 53.7 (CH), 46.6 (CH), 28.3 (3 × CH_3), 28.0 (CH_2), 26.1 (CH_2), 14.1 (CH_3). One carbon obscured by DMSO-*d*$_6$ (CH_2).

HRMS-CI (m/z): $[M + H]^+$ calcd for $C_{27}H_{35}N_2O_6$, 483.2490; found, 483.2476.

*Synthesis of the α,β-unsaturated ester **12**.*

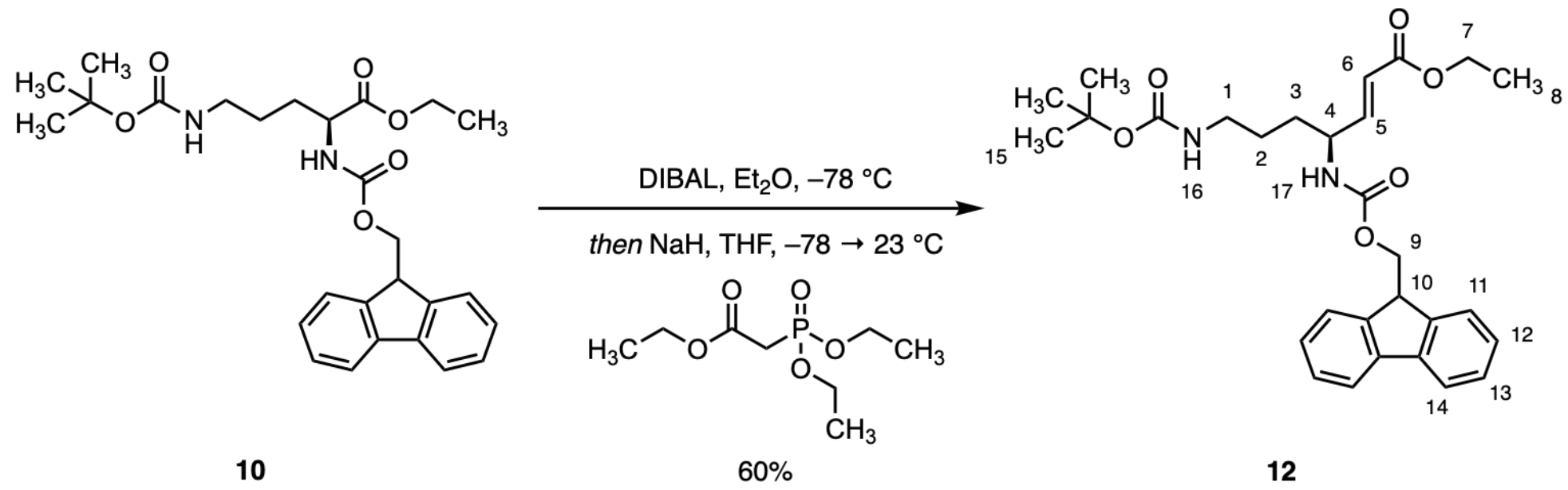

The following procedure has been adapted from the literature with modifications.[18,19] Di-*iso*-butylaluminum hydride (DIBAL, 1.0 M in toluene, 50.0 mL, 50.0 mmol, 2.20 equiv) was slowly added dropwise over 15 min (addition funnel) to a suspension of Fmoc-Boc(Orn)-OEt **10** (11.0 g, 22.8 mmol, 1.0 equiv) in diethyl ether (90 mL, C = 0.25 M) at –78 °C. The mixture was stirred at 300 rpm for 30 min at –78 °C. A solution of the phosphonoacetate anion (*vide infra*) was added *via* cannula at –78 °C. The reaction mixture was removed from the cooling bath and stirred at 800 rpm for 7 h at 23 °C. The product mixture was quenched by the addition of water (110 mL) and a saturated aqueous solution of sodium potassium tartrate (110 mL). The quenched product mixture was vigorously stirred (800 – 1000 rpm) until the phases separated (12 h). The biphasic mixture was transferred to a separatory funnel, and the organic layer was separated. The aqueous layer was extracted with ethyl acetate (3 × 150 mL). The combined organic layers were dried over sodium sulfate. The dried solution was filtered, and the filtrate was concentrated. The residue was purified by flash column chromatography (eluting with 10% ethyl acetate–hexane initially, grading to 35% ethyl acetate–hexane, linear gradient) to afford the *α,β-unsaturated ester* **12** as a white solid (6.98 g, 60%).

Preparation of triethyl phosphonoacetate anion:
Triethyl phosphonoacetate (5.40 mL, 27.4 mmol, 1.20 equiv) was added to a suspension of sodium hydride (1.00 g, 25.1 mmol, 1.10 equiv) in tetrahydrofuran (134 mL, C = 0.17 M) at 0 °C. The mixture was stirred at 0 °C until the evolution of dihydrogen gas ceased (15 min). The clear and homogeneous solution was directly cannulated into the reaction mixture.

Note: The ester **10** was finely ground with a pestle and a mortar before use.

R_f = 0.38 (30% ethyl acetate–hexane; UV)

$[\alpha]_D^{20}$: –5.0 (*c* 0.77, $CHCl_3$)

^{1}H NMR (500 MHz, DMSO-d_6) δ 7.88 (d, *J* = 7.6 Hz, 2H, H_{14}), 7.70 (t, *J* = 7.0 Hz, 2H, H_{11}), 7.56 (d, *J* = 8.5 Hz, 1H, H_{17}), 7.41 (t, *J* = 7.5 Hz, 2H, H_{13}), 7.33 (t, *J* = 7.4 Hz, 2H, H_{12}), 6.79 (dd, *J* = 15.7, 5.6 Hz, 2H, H_5, H_{16}), 5.85 (d, *J* = 15.7 Hz, 1H, H_6), 4.38 – 4.27 (m, 2H, H_9), 4.23 (t, *J* = 7.0 Hz, 1H, H_{10}), 4.16 (d, *J* = 6.2 Hz, 1H, H_4), 4.12 (q, *J* = 7.1 Hz, 2H, H_7), 2.92 (q, *J* = 6.6 Hz, 2H,

H_1), 1.57 – 1.47 (m, 1H, H_3), 1.43 (m, 3H, 2 × H_2, H_3), 1.37 (s, 9H, H_{15}), 1.21 (t, *J* = 7.1 Hz, 3H, H_8).

^{13}C NMR (126 MHz, DMSO-*d₆*) δ 165.6 (C), 155.6 (2 × C), 149.4 (CH), 143.9 (2 × C), 140.8 (2 × C), 127.6 (2 × CH), 127.0 (2 × CH), 125.2 (2 × CH), 120.0 (2 × CH), 119.8 (CH), 77.4 (C), 65.3 (CH_2), 59.9 (CH_2), 51.3 (CH), 46.8 (CH), 30.7 (CH_2), 28.3 (3 × CH_3), 26.1 (CH_2), 14.1 (CH_3). One carbon obscured by DMSO-d_6 (CH_2).

HRMS-CI (m/z): $[M + H]^+$ calcd for $C_{29}H_{37}N_2O_6$, 509.2646; found, 509.2635.

*Synthesis of the allylic alcohol **S9**.*

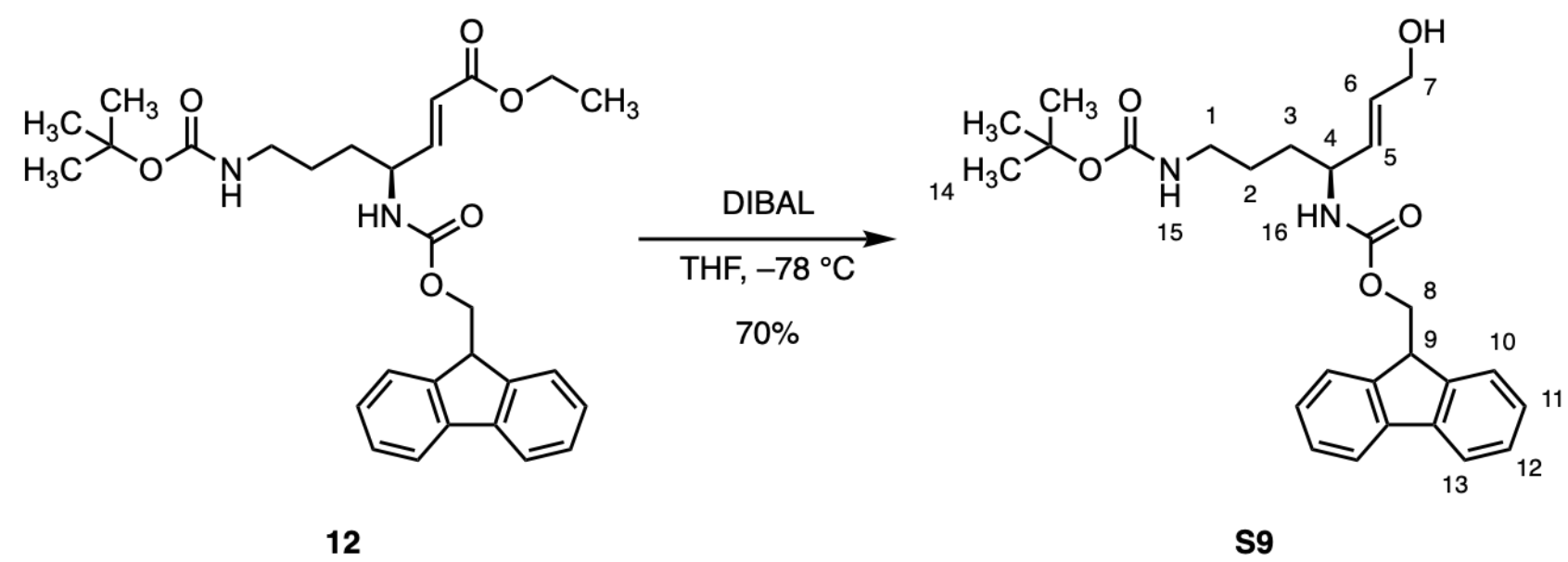

To a solution of α,β-unsaturated ester **12** (1.00 g, 1.97 mmol, 1 equiv) in tetrahydrofuran (5.0 mL, C = 0.40 M) was added di-*iso*-butylaluminum hydride (DIBAL, 1.0 M in toluene, 5.90 mL, 5.90 mmol, 3.00 equiv) dropwise at –78 °C. The reaction mixture was stirred for 2.5 h at –78 °C. An additional portion of di-*iso*-butylaluminum (DIBAL, 1.0 M in toluene, 2.00 mL, 1.97 mmol, 1.00 equiv) was added and the reaction mixture was stirred for 1.5 h at –78 °C. The product mixture was quenched by *careful* addition of methanol (2 mL) and diluted with a half saturated aqueous solution of sodium potassium tartrate (15 mL). The cooling bath was removed, and the quenched product mixture was warmed to 23 °C. The quenched product mixture was stirred at 23 °C until the phases separated and turned homogeneous (3–4 h). The biphasic mixture was transferred to a separatory funnel, and the organic layer was separated. The aqueous phase was extracted with ethyl acetate (3 × 15 mL). The combined organic layers were dried over sodium sulfate. The dried solution was filtered, and the filtrate was concentrated. The residue was purified by flash column chromatography (eluting with 40% ethyl acetate–hexane initially, grading to 70% ethyl acetate–hexane, linear gradient) to afford the *allylic alcohol* **S9** as a white solid (640 mg, 70%).

R_f = 0.31 (60% ethyl acetate–hexane; UV)

$[\alpha]_D^{20}$: +2.5 (*c* 1.2, $CHCl_3$)

^{1}H NMR (500 MHz, DMSO-*d*$_6$) δ 7.89 (d, *J* = 7.6 Hz, 2H, H_{13}), 7.70 (dd, *J* = 7.7, 3.8 Hz, 2H, H_{10}), 7.41 (t, *J* = 7.5 Hz, 2H, H_{12}), 7.33 (q, *J* = 6.5 Hz, 3H, 2 × H_{11}, H_{16}), 6.78 (t, *J* = 6.0 Hz, 1H, H_{15}), 5.54 (qd, *J* = 15.5, 8.3 Hz, 2H, H_5, H_6), 4.68 (t, *J* = 5.4 Hz, 1H, H_{17}), 4.33 – 4.17 (m, 3H, 2 × H_8, H_9), 3.96 – 3.87 (m, 3H, H_4, 2 × H_7), 2.89 (d, *J* = 6.7 Hz, 2H, H_1), 1.37 (s, 13H, 2 × H_2, 2 × H_3, 9 × H_{14}).

^{13}C NMR (126 MHz, DMSO-*d*$_6$) δ 156.0 (2 × C), 144.4 (2 × C), 141.2 (2 × C), 131.0 (CH), 130.5 (CH), 128.1 (2 × CH), 127.5 (2 × CH), 125.7 (2 × CH), 120.6 (2 × CH), 77.8 (C), 65.7 (CH_2), 61.4 (CH_2), 52.4 (CH), 47.3 (CH), 32.5 (CH_2), 28.8 (3 × CH_3), 26.8 (CH_2). One carbon obscured by DMSO-*d*$_6$ (CH_2).

HRMS-CI (m/z): $[M + H]^+$ calcd for $C_{27}H_{35}N_2O_5$, 467.2540; found, 467.2529.

Note: 106 mg (~10%) of the α,β-unsaturated aldehyde **9** was also recovered (*vide infra*).

*Synthesis of the α,β-unsaturated aldehyde **9**.*

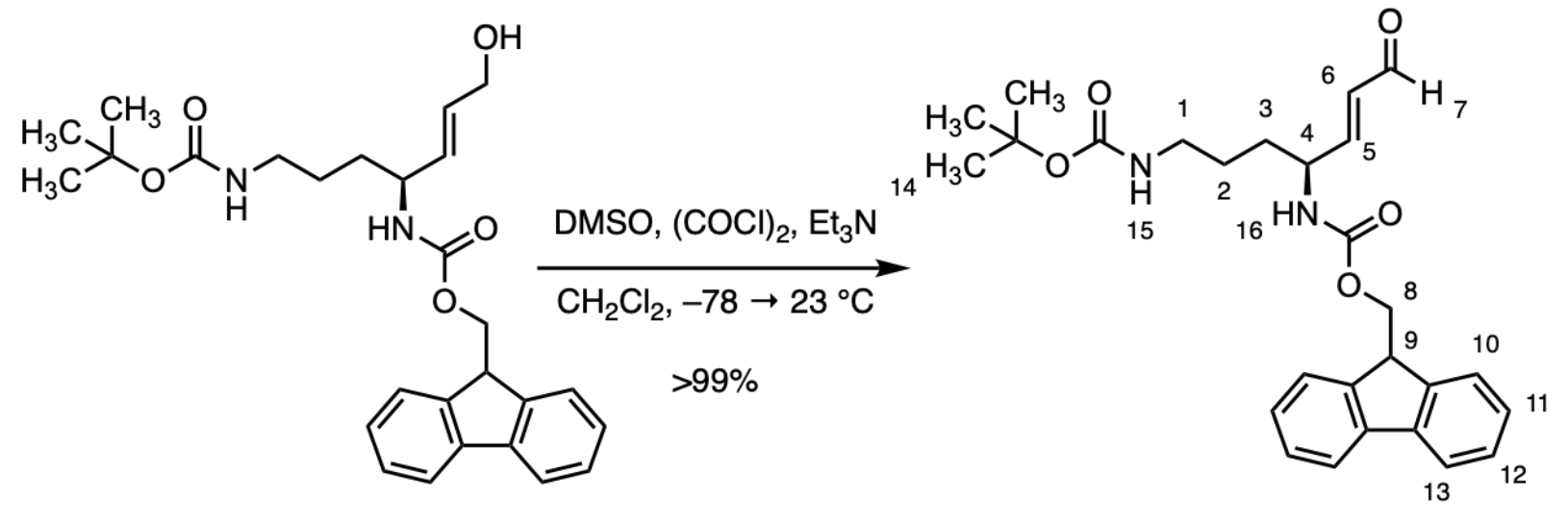

S9 **9**

To a cooled (–78 °C) solution of oxalyl chloride (0.650 mL, 7.54 mmol, 1.10 equiv) in dichloromethane (70 mL, C = 0.10 M) was added dimethylsulfoxide (DMSO, 1.10 mL, 15.1 mmol, 2.20 equiv). The reaction mixture was stirred for 10 min at –78 °C. A solution of allylic alcohol **S9** (3.20 g, 6.86 mmol, 1 equiv) in dichloromethane (20 mL, C = 0.35 M) was added to the reaction mixture and stirred for 15 min at –78 °C. Triethylamine (4.80 mL, 34.3 mmol, 5.00 equiv) was added and the reaction mixture was stirred for 5 min at –78 °C. The cooling bath was removed, and the reaction mixture was stirred for 1 h at 23 °C. The product mixture was quenched with a saturated aqueous ammonium chloride solution (70 mL). The quenched product mixture was transferred to a separatory funnel and the layers that formed were separated. The aqueous layer was extracted with dichloromethane (2 × 40 mL). The combined organic layers were washed with a saturated aqueous sodium bicarbonate solution (2 × 100 mL) and a saturated aqueous sodium chloride solution (100 mL). The washed organic layer was dried over sodium sulfate. The dried solution was filtered, and the filtrate was concentrated to afford *α,β-unsaturated aldehyde* **9** as a white solid (3.18 g, >99%). The product obtained in this way was judged to be of >95% purity (^{1}H NMR analysis) and was advanced without purification.

R_f = 0.44 (50% ethyl acetate–hexane; UV)

$[\alpha]_D^{20}$: –2.7 (*c* 0.79, $CHCl_3$)

^{1}H NMR (500 MHz, DMSO-*d6*) δ 9.53 (d, *J* = 7.9 Hz, 1H, H_7), 7.89 (d, *J* = 7.6 Hz, 2H, H_{13}), 7.70 (t, *J* = 6.9 Hz, 2H, H_{10}), 7.63 (d, *J* = 8.4 Hz, 1H, H_{16}), 7.41 (t, *J* = 7.5 Hz, 2H, H_{12}), 7.33 (t, *J* = 7.4 Hz, 2H, H_{11}), 6.94 (dd, *J* = 15.6, 5.2 Hz, 1H, H_5), 6.81 (t, *J* = 5.8 Hz, 1H, H_{15}), 6.09 – 6.00 (m, 1H, H_6), 4.34 (d, *J* = 7.1 Hz, 2H, H_8), 4.27 (d, *J* = 4.7 Hz, 1H, H_4), 4.23 (t, *J* = 6.9 Hz, 1H, H_9), 2.92 (q, *J* = 6.5 Hz, 2H, H_1), 1.63 – 1.51 (m, 1H, H_3), 1.51 – 1.39 (m, 3H, 2 × H_2, H_3), 1.37 (s, 9H, H_{14}).

^{13}C NMR (126 MHz, DMSO-*d6*) δ 194.8 (CH), 159.5 (C), 156.1 (C), 144.3 (2 × C), 141.2 (2 × C), 130.9 (2 × CH), 128.1 (2 × CH), 127.5 (2 × CH), 125.6 (2 × CH), 120.6 (2 × CH), 77.9 (C), 65.8 (CH_2), 52.1 (CH), 47.2 (CH), 30.9 (CH_2), 28.7 (3 × CH_3), 26.6 (CH_2). One carbon obscured by DMSO-*d6* (CH_2).

HRMS-CI (m/z): $[M + H]^+$ calcd for $C_{27}H_{33}N_2O_5$, 465.2384; found, 465.2368.

*Synthesis of the bromo-aldol adduct **S10**.*

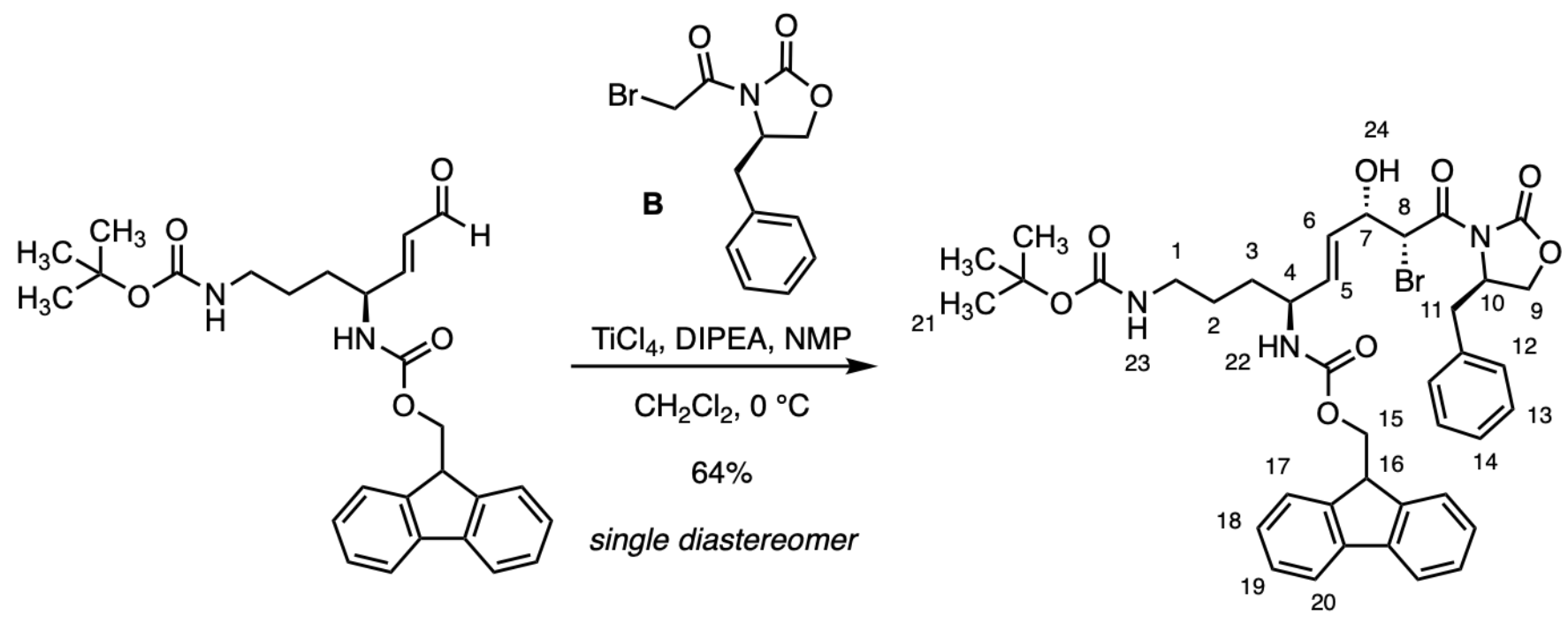

The following procedure has been adapted from the literature with modifications.[20] To a cooled (0 °C) solution of (*R*)-4-benzyl-3-(2-bromoacetyl)oxazolidin-2-one **B** (0.974 g, 3.27 mmol, 1.10 equiv) in dichloromethane (30 mL, C = 0.10 M) was added titanium (IV) chloride (1.0 M in dichloromethane, 3.40 mL, 3.42 mmol, 1.15 equiv). The reaction mixture was stirred for 15 min at 0 °C. To the dark red solution was added *N*,*N*-diisopropylethylamine (DIPEA, 0.620 mL, 3.57 mmol, 1.20 equiv) and the reaction mixture was stirred for 40 min at 0 °C. At this point the solution turned dark purple, and smoke was observed. To this solution was added *N*-methylpyrrolidone (0.310 mL, 3.27 mmol, 1.10 equiv) and the reaction mixture was stirred for an additional 10 min at 0 °C. A solution of α,β-unsaturated aldehyde **9** (1.38 g, 2.97 mmol, 1 equiv) in dichloromethane (8.5 mL, C = 0.36 M) was added dropwise into the titanium enolate solution. The reaction mixture was stirred for 2 h at 0 °C. The solution gradually turned orange during the course of the reaction. The product mixture was quenched with a half-saturated aqueous solution of ammonium chloride (40 mL). The quenched product mixture was transferred to a separatory funnel and the layers that formed were separated. The aqueous layer was extracted with dichloromethane (2 × 30 mL). The combined organic extracts were dried over sodium sulfate. The dried solution was filtered, and the filtrate was concentrated. The brown oily residue was purified by flash column chromatography (eluting with 30 % ethyl acetate–hexane initially, grading to 60 % ethyl acetate–hexane, linear gradient) to afford the *bromo-aldol adduct* **S10** as a light yellow-brown solid (1.44 g, 64%, *dr* >99:1).

R_f = 0.42 (50% ethyl acetate–hexane; UV)

$[\alpha]_D^{20}$: –9.7 (*c* 1.2, $CHCl_3$)

^{1}H NMR (500 MHz, DMSO-d_6) δ 7.91 – 7.86 (m, 2H, H_{20}), 7.66 (dd, *J* = 7.7, 3.2 Hz, 2H, H_{17}), 7.44 – 7.38 (m, 3H, 2 × H_{19}, H_{22}), 7.35 – 7.19 (m, 7H, H_{18}), 6.77 (t, *J* = 5.7 Hz, 1H, H_{23}), 5.74 (d, *J* = 14.8 Hz, 1H, H_{24}), 5.61 (dd, *J* = 15.5, 6.1 Hz, 1H, H_6), 5.53 (d, *J* = 8.5 Hz, 1H, H_8), 5.47 (dd, *J* = 15.5, 6.5 Hz, 1H, H_5), 4.60 (td, *J* = 7.0, 3.3 Hz, 1H, H_{10}), 4.36 (t, *J* = 7.4 Hz, 1H, H_7), 4.32 – 4.10 (m, 5H, 2 × H_9, 2 × H_{15}, H_{16}), 3.95 (t, *J* = 6.9 Hz, 1H, H_4), 3.02 – 2.92 (m, 2H, H_{11}), 2.88 (d, *J* = 6.5 Hz, 2H, H_1), 1.36 (s, 13H, 2 × H_2, 2 × H_3, 9 × H_{21}).

^{13}C NMR (126 MHz, DMSO-*d*$_6$) δ 167.3 (C), 155.6 (C), 155.5 (C), 152.5 (C), 143.8 (2 × C), 140.7 (2 × C), 135.3 (CH), 135.0 (C), 129.6 (2 × CH), 128.6 (2 × CH), 127.6 (3 × CH), 127.1 (2 × CH), 127.0 (CH), 125.1 (2 × CH), 120.1 (2 × CH), 77.4 (C), 71.4 (CH), 66.1 (CH_2), 65.2 (CH_2), 54.5 (CH), 51.8 (CH), 49.0 (CH), 46.7 (CH), 35.8 (CH_2), 31.6 (CH_2), 28.3 (3 × CH_3), 26.2 (CH_2). One carbon obscured by DMSO-*d*$_6$ (CH_2).

HRMS-CI (m/z): $[M + H]^+$ calcd for $C_{39}H_{45}BrN_3O_8$, 762.2385; found, 762.2357.

*Synthesis of the azide **13**.*

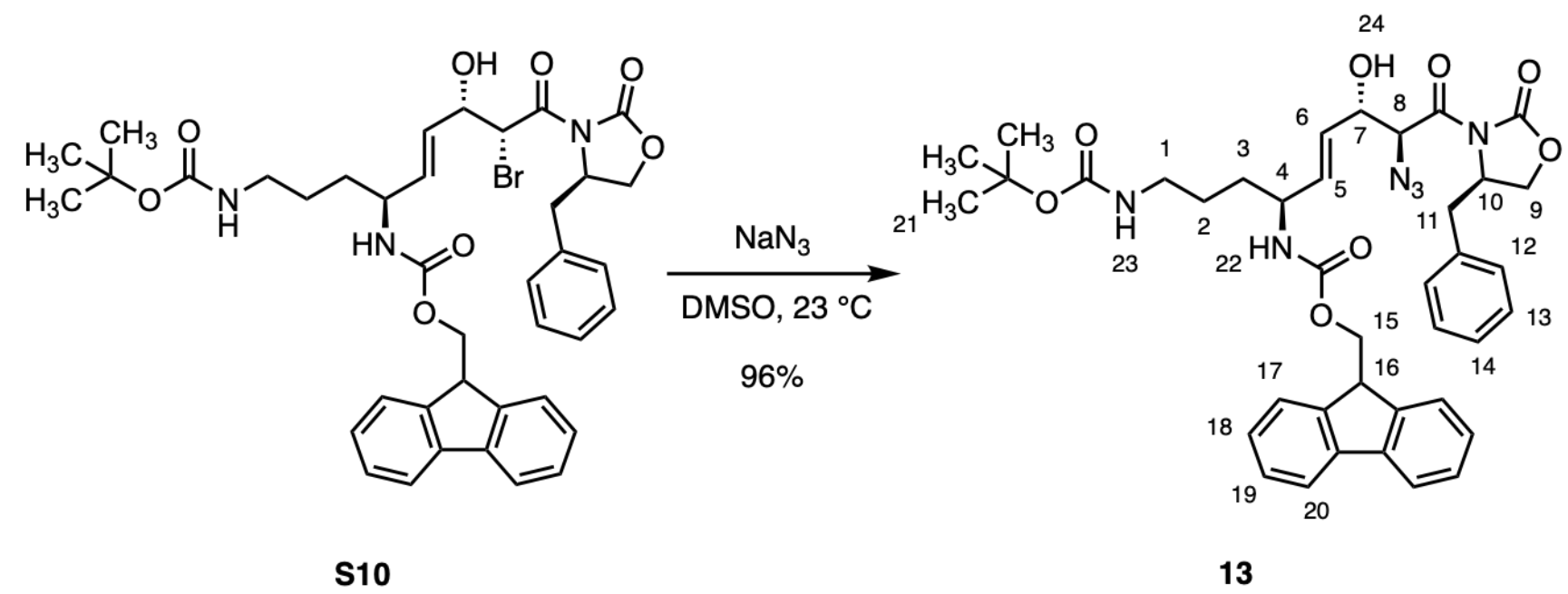

The following procedure has been adapted from the literature.[21] To a solution of bromo-aldol adduct **S10** (3.00 g, 3.93 mmol, 1 equiv) in dimethylsulfoxide (14 mL, C = 0.28 M) was added sodium azide (0.511 g, 7.87 mmol, 2.00 equiv). The reaction mixture was stirred for 2 h at 23 °C. The resultant orange solution was diluted with 900 mL of hexane/dichloromethane (2:1, *v*/*v*) and transferred to a separatory funnel. The diluted product mixture was washed with water (4 × 320 mL). The washed product mixture was dried over sodium sulfate. The dried solution was filtered, and the filtrate was concentrated. 10 mL of dichloromethane was added to the residue, and the solution was concentrated again to afford the *azide* **13** as a brown-yellow foamy solid (2.73 g, 96%). The product obtained in this way was judged to be of >95% purity (^{1}H NMR analysis) and was advanced without purification.

R_f = 0.30 (40% ethyl acetate–hexane; UV)

$[\alpha]_D^{20}$: –5.4 (*c* 0.86, $CHCl_3$)

^{1}H NMR (500 MHz, DMSO-*d*$_6$) δ 7.87 (d, *J* = 7.5 Hz, 2H, H_{20}), 7.68 (t, *J* = 7.2 Hz, 2H, H_{17}), 7.45 (d, *J* = 8.7 Hz, 1H, H_{22}), 7.41 (t, *J* = 7.5 Hz, 2H, H_{19}), 7.36 – 7.20 (m, 7H, 2 × H_{12}, 2 × H_{13}, H_{14}, 2 × H_{18}), 6.76 (t, *J* = 5.7 Hz, 1H, H_{23}), 5.83 (d, *J* = 5.6 Hz, 1H, H_{24}), 5.82 – 5.74 (m, 1H, H_5), 5.67 (dd, *J* = 15.5, 6.4 Hz, 1H, H_6), 5.11 (d, *J* = 6.6 Hz, 1H, H_8), 4.72 (ddt, *J* = 11.9, 8.1, 3.5 Hz, 1H, H_{10}), 4.51 (q, *J* = 6.1 Hz, 1H, H_7), 4.34 (t, *J* = 8.5 Hz, 1H, H_9), 4.27 – 4.13 (m, 4H, H_9, 2 × H_{15}, H_{16}), 4.03 (t, *J* = 7.2 Hz, 1H, H_4), 3.05 (dd, *J* = 13.5, 3.4 Hz, 1H, H_{11}), 2.99 – 2.78 (m, 3H, 2 × H_1, H_{11}), 1.50 – 1.39 (m, 4H, 2 × H_2, 2 × H_3), 1.36 (s, 9H, H_{21}).

^{13}C NMR (126 MHz, DMSO-*d*$_6$) δ 168.4 (C), 155.7 (2 × C), 153.2 (C), 144.0 (2 × C), 140.8 (2 × C), 135.6 (C), 134.6 (CH), 129.6 (CH), 128.7 (CH), 127.7 (d, *J* = 10.8 Hz, CH), 127.1 (CH), 127.0 (CH), 125.3 (t, *J* = 6.7 Hz, CH), 120.2 (CH), 77.5 (C), 71.5 (CH), 66.4 (CH_2), 65.4 (CH_2), 63.0 (CH), 54.5 (CH), 51.8 (CH), 46.8 (CH), 36.6 (CH_2), 31.8 (CH_2), 28.3 (CH_3), 26.4 (CH_2). One carbon obscured by DMSO-*d*$_6$ (CH_2).

HRMS-CI (m/z): $[M + H]^+$ calcd for $C_{39}H_{45}N_6O_8$, 725.3293; found, 725.3266.

*Synthesis of the carboxylic acid **14**.*

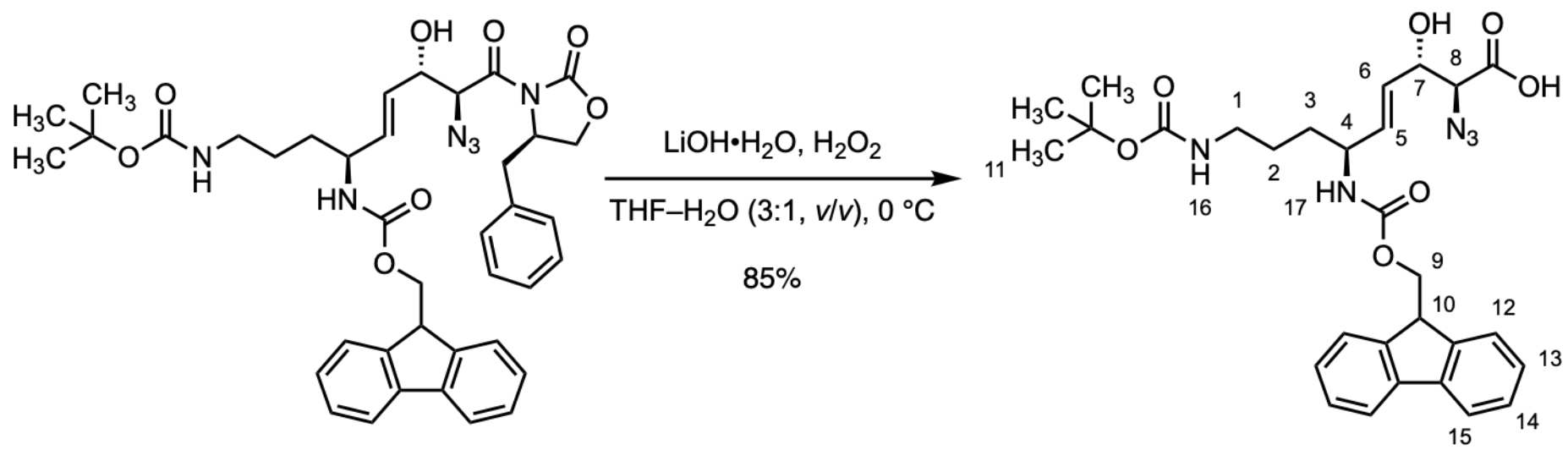

The following procedure has been adapted from the literature with modifications.[22] To a solution of azide **13** (1.58 g, 2.18 mmol, 1 equiv) in tetrahydrofuran/water (50 mL, 3:1 *v*/*v*) was added 30% hydrogen peroxide (1.00 mL, 13.2 mmol, 6.06 equiv) and lithium hydroxide monohydrate (0.184 g, 4.39 mmol, 2.00 equiv) at 0 °C. The reaction mixture was stirred for 2 h at 0 °C. The product mixture was quenched by the addition of 0.55 M aqueous sodium sulphite solution (27 mL) at 0 °C. The pH was adjusted to 9–10 with saturated aqueous sodium bicarbonate solution (5 mL). The quenched product mixture was partially concentrated and transferred to a separatory funnel. The aqueous phase was extracted with diethyl ether (3 × 25 mL) and discarded. The washed aqueous phase was acidified to pH 4 – 5 with 1.0 M aqueous hydrogen chloride solution (20 mL) and extracted with ethyl acetate (3 × 25 mL). The combined organic extracts were dried over sodium sulfate. The dried solution was filtered, and the filtrate was concentrated to afford the *carboxylic acid* **14** as a pale-yellow solid (1.05 g, 85 %). The product obtained in this way was judged to be of >95% purity (^{1}H NMR analysis) and was advanced without purification.

Note: The product was stored in a –20 °C freezer to prevent decomposition and was consumed within a few weeks of preparation for best yields in the upcoming steps (*vide infra*).

R_f = 0.21 (80% ethyl acetate–hexane + 0.1% acetic acid; UV, long streak)

$[\alpha]_D^{20}$: +7.7 (*c* 0.92, $CHCl_3$)

^{1}H NMR (500 MHz, DMSO-*d*$_6$) δ 13.13 (br s, 1H, –COO*H*), 7.90 (d, *J* = 7.6 Hz, 2H, H_{15}), 7.71 (dt, *J* = 7.6, 1.7 Hz, 2H, H_{12}), 7.41 (q, *J* = 6.1 Hz, 3H, 2 × H_{13}, H_{14}), 7.34 (tdd, *J* = 7.3, 3.0, 1.2 Hz, 2H, H_{13}), 6.76 (t, *J* = 5.8 Hz, 1H, H_{16}), 5.67 (dd, *J* = 15.6, 6.1 Hz, 1H, H_6), 5.57 (dd, *J* = 15.5, 5.8 Hz, 1H, H_5), 4.36 (t, *J* = 5.5 Hz, 1H, H_7), 4.32 – 4.17 (m, 3H, 2 × H_9, 1 × H_{10}), 4.00 – 3.96 (m, 1H, H_4), 3.94 (d, *J* = 5.2 Hz, 1H, H_8), 2.90 (d, *J* = 7.1 Hz, 2H, H_1), 1.38 (s, 13H, 2 × H_2, 2 × H_3, 9 × H_{11}).

^{13}C NMR (126 MHz, DMSO-*d*$_6$) δ 169.6 (C), 155.6 (C), 155.5 (C), 143.9 (2 × C), 140.7 (2 × C), 133.5 (CH), 128.0 (CH), 127.6 (2 × CH), 127.1 (2 × CH), 125.3 (2 × CH), 120.1 (2 × CH), 77.4 (C), 71.6 (CH), 66.1 (CH), 65.3 (CH_2), 52.5 (CH), 46.8 (CH), 31.9 (CH_2), 28.3 (3 × CH_3), 26.2 (CH_2). One carbon obscured by DMSO-*d*$_6$ (CH_2).

HRMS-CI (m/z): $[M + Na]^+$ calcd for $C_{29}H_{35}N_5O_7Na$, 588.2429; found, 588.2416.

*Synthesis of the guanylated acid **15**.*

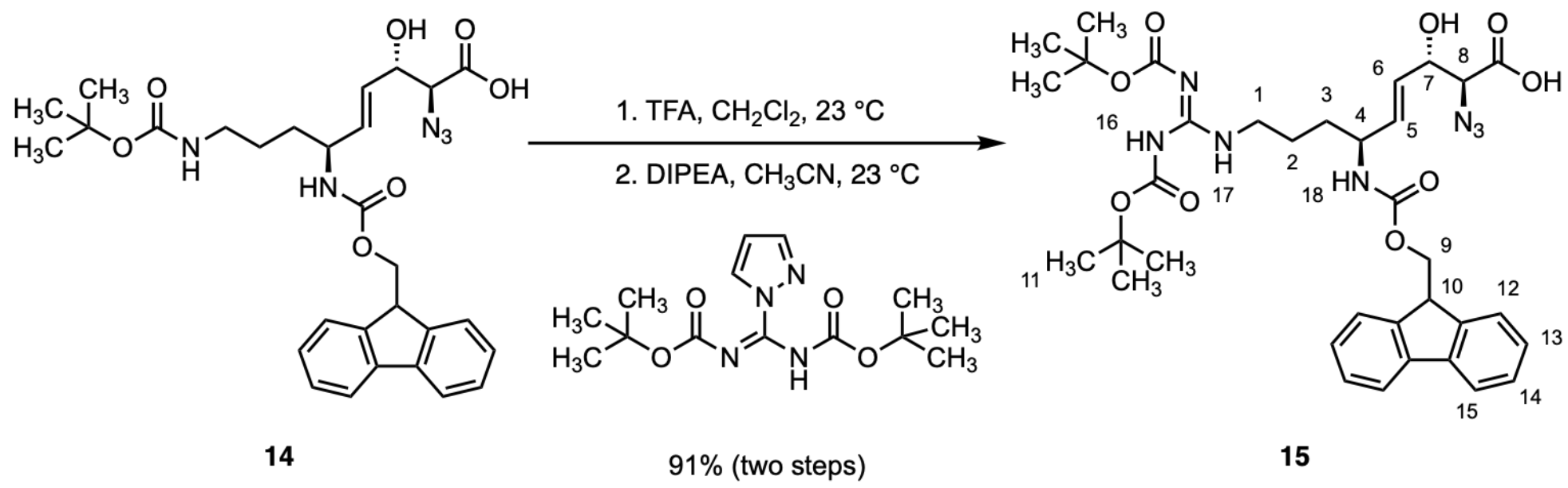

The following procedure has been adapted from the literature with modifications.[23] To a solution of carboxylic acid **14** (600 mg, 1.06 mmol, 1 equiv) in dichloromethane (8.0 mL) was added trifluoroacetic acid (0.80 mL) at 23 °C. The reaction mixture was stirred for 2 h at 23 °C. The resulting dark orange solution was concentrated and redissolved in dichloromethane (5 mL). The solution was concentrated again to remove residual acid. The residue was subjected to high vacuum to afford a brown foamy gum. This material was used directly in the next step without further treatment.

To a solution of the crude product obtained above (nominally 1.06 mmol, 1 equiv) in acetonitrile (5.3 mL) were added *N*,*N*-diisopropylethylamine (0.920 mL, 5.31 mmol, 5.00 equiv) and 1*H*-pyrazole-1-[*N*,*N′*-bis(*tert*-butoxycarbonyl)]-carboxamidine (0.395 g, 1.27 mmol, 1.20 equiv). The reaction mixture was stirred for 18 h at 23 °C. The product mixture was diluted with 5.3 mL of water. The diluted product mixture was acidified to pH 4 – 5 with aqueous 1.0 M hydrogen chloride solution (3.5 mL). The quenched product mixture was transferred to a separatory funnel and the layers that formed were separated. The aqueous phase was extracted with ethyl acetate (3 × 15 mL). The combined organic extracts were washed with water (3 × 20 mL) to remove residual pyrazole. The washed organic extract was dried over sodium sulfate. The dried solution was filtered, and the filtrate was concentrated. The residue was purified by flash column chromatography (eluting with 50 % ethyl acetate–hexane initially, grading to 80 % ethyl acetate–hexane, linear gradient) to afford the *guanylated acid* **15** as a deliquescent pale-yellow solid (680 mg, 91%).

Note 1: An analytical sample was prepared by repurification with 0 → 10% methanol–dichloromethane, linear gradient.

Note 2: Small shifts in the ^{1}H and ^{13}C-NMR were observed between different samples of compound **15** (most likely due to the formation of zwitterions). The reported analytical data correspond to the shifts obtained after the second purification.

Note 3: The solution becomes viscous and produces fumes upon the addition of the base. The reaction mixture eventually homogenizes within 3 – 4 hours after the addition of the guanylating agent **C**.

Note 4: The product was stored in a –20 °C freezer to prevent decomposition and was consumed within a few weeks of preparation for best yields in the upcoming steps (*vide infra*).

R_f = 0.48 (80% ethyl acetate–hexane + 0.1% acetic acid; UV, long streak)

$[\alpha]_D^{20}$: +2.3 (*c* 0.49, $CHCl_3$)

^{1}H NMR (500 MHz, DMSO-d_6) δ 11.50 (s, 1H, H_{16}), 8.28 (t, *J* = 5.6 Hz, 1H, H_{17}), 7.88 (d, *J* = 7.5 Hz, 2H, H_{15}), 7.70 (dd, *J* = 7.6, 4.2 Hz, 2H, H_{12}), 7.43 (d, *J* = 8.6 Hz, 1H, H_{18}), 7.42 – 7.38 (m, 2H, H_{14}), 7.32 (tdd, *J* = 7.4, 3.3, 1.2 Hz, 2H, H_{13}), 5.63 (qd, *J* = 15.2, 5.3 Hz, 2H, 1 × H_5, 1 × H_6), 4.30 (dd, *J* = 9.9, 6.9 Hz, 1H, H_7), 4.26 – 4.17 (m, 3H, 2 × H_9, 1 × H_{10}), 3.98 (app h, *J* = 6.4 Hz, 1H, H_4), 3.57 (d, *J* = 8.5 Hz, 1H, H_8), 3.31 – 3.22 (m, 2H, H_1), 1.46 (s, 13H, 2 × H_2, 2 × H_3, 9 × H_{11}), 1.38 (s, 9H, H_{11}).

^{13}C NMR (126 MHz, DMSO-d_6) δ 171.0 (C), 163.1 (C), 155.5 (C), 155.2 (C), 152.1 (C), 144.0 (C), 143.8 (C), 140.7 (2 × C), 132.1 (CH), 129.7 (CH), 127.6 (2 × CH), 127.0 (2 × CH), 125.2 (2 × CH), 120.1 (2 × CH), 82.9 (C), 78.1 (C), 71.3 (CH), 66.7 (CH), 65.3 (CH_2), 52.1 (CH), 46.8 (CH), 31.9 (CH_2), 28.0 (3 × CH_3), 27.6 (3 × CH_3), 25.4 (CH_2). One carbon obscured by DMSO-d_6 (CH_2).

HRMS-CI (m/z): $[M + H]^+$ calcd for $C_{35}H_{46}N_7O_9$, 708.3352; found, 708.3378.

*Synthesis of the dipeptide **16**.*

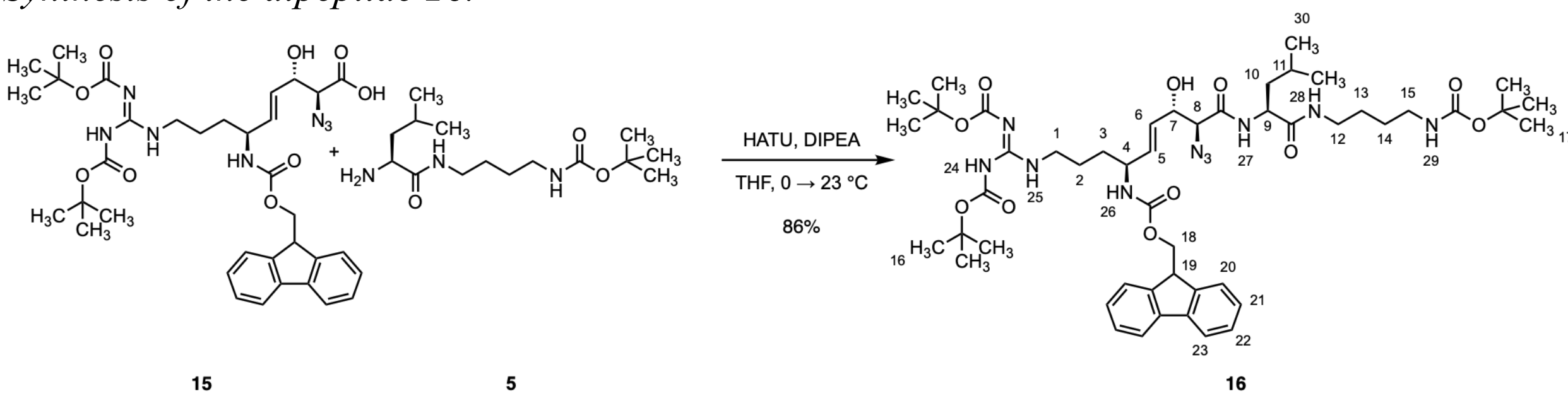

To a cooled (0 °C) solution of guanylated acid **15** (1.00 g, 1.41 mmol, 1 equiv) and NH_2-Leu-*N*-Boc-Put **5** (0.554 g, 1.84 mmol, 1.30 equiv) in tetrahydrofuran (8.3 mL) were added hexafluorophosphate azabenzotriazole tetramethyluronium (HATU, 0.741 g, 1.95 mmol, 1.38 equiv) and *N*,*N*-diisopropylethylamine (0.850 mL, 4.89 mmol, 3.46 equiv). The cooling bath was removed, and the reaction mixture was stirred for 18 h at 23 °C. The product mixture was concentrated then diluted with ethyl acetate (20 mL). The diluted product mixture was transferred to a separatory funnel and washed with 1% aqueous hydrogen chloride solution (10 mL), saturated aqueous sodium bicarbonate solution (10 mL), water (10 mL), and saturated aqueous sodium chloride solution (10 mL). The washed organic layer was dried over sodium sulfate. The dried solution was filtered, and the filtrate was concentrated. The residue was purified by flash column chromatography (eluting with 50 % ethyl acetate–hexane initially, grading to 80 % ethyl acetate, linear gradient) to afford the *dipeptide* **16** as an off-white solid (1.20 g, 86%).

R_f = 0.46 (5% methanol–dichloromethane; UV)

$[\alpha]_D^{20}$: +3.1 (*c* 0.54, $CHCl_3$)

^{1}H NMR (500 MHz, DMSO-d_6) δ 11.51 (s, 1H, H_{24}), 8.29 (t, *J* = 5.7 Hz, 1H, H_{25}), 8.15 (d, *J* = 8.4 Hz, 1H, H_{27}), 7.88 (d, *J* = 7.6 Hz, 2H, H_{23}), 7.85 (t, *J* = 5.6 Hz, 1H, H_{28}), 7.70 (dd, *J* = 7.6, 3.1 Hz, 2H, H_{20}), 7.47 (d, *J* = 8.8 Hz, 1H, H_{26}), 7.40 (t, *J* = 7.5 Hz, 2H, H_{22}), 7.32 (td, *J* = 7.5, 2.9 Hz, 2H, H_{21}), 6.75 (t, *J* = 5.8 Hz, 1H, H_{29}), 5.79 – 5.70 (m, 2H, H_5, –O*H*), 5.58 (dd, *J* = 15.5, 6.2 Hz, 1H, H_6), 4.38 – 4.18 (m, 5H, 1 × H_7, 1 × H_9, 2 × H_{18}, 1 × H_{19}), 4.07 – 3.95 (m, 1H, H_4), 3.75 (d, *J* = 7.3 Hz, 1H, H_8), 3.28 (app s, 2H, H_1), 3.09 – 2.96 (m, 2H, H_{12}), 2.88 (d, *J* = 6.1 Hz, 2H, H_{15}), 1.54 (app td, *J* = 14.0, 7.3 Hz, 1H, H_{11}), 1.46 (br s, 14H, 2 × H_2, 2 × H_3, 1 × H_{10}, 9 × H_{16}), 1.38 (br s, 9H, H_{16}), 1.36 (br s, 10H, 1 × H_{10}, 9 × H_{17}), 1.34 – 1.31 (m, 4H, 2 × H_{13}, 2 × H_{14}), 0.85 (dd, *J* = 16.9, 6.5 Hz, 6H, H_{30}).

^{13}C NMR (126 MHz, DMSO-d_6) δ 171.2 (C), 167.7 (C), 163.2 (C), 155.6 (2 × C), 155.3 (C), 152.1 (C), 143.9 (2 × C), 140.7 (2 × C), 133.6 (CH), 128.4 (CH), 127.6 (2 × CH), 127.0 (2 × CH), 125.2 (2 × CH), 120.1 (2 × CH), 82.9 (C), 78.1 (C), 77.3 (C), 71.2 (CH), 65.3 (CH_2), 65.2 (CH), 51.9 (CH), 51.2 (CH), 46.8 (CH), 40.8 (CH_2), 38.4 (CH_2), 31.8 (CH_2), 28.3 (3 × CH_3), 28.0 (3 × CH_3), 27.6 (3 × CH_3), 26.9 (CH_2), 26.4 (CH_2), 25.4 (CH_2), 24.3 (CH), 23.0 (CH_3), 21.4 (CH_3).
Two carbons obscured by DMSO-d_6 (2 × CH_2).

HRMS-CI (m/z): $[M + H]^+$ calcd for $C_{50}H_{75}N_{10}O_{11}$, 991.5651; found, 991.5618.

*Synthesis of the amino alcohol **S11**.*

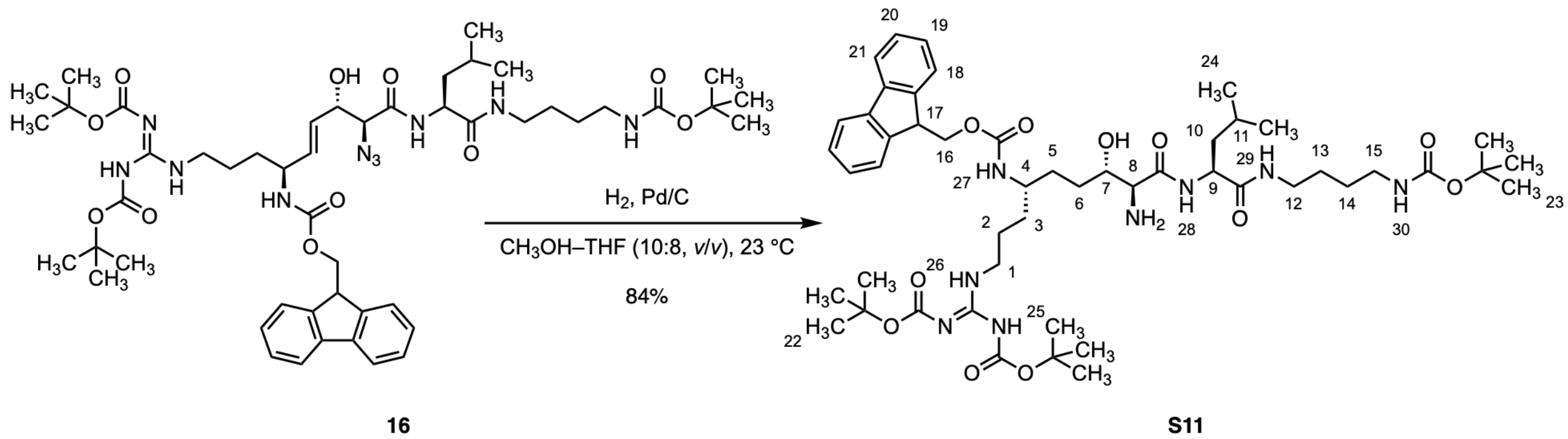

The following procedure has been adapted from the literature.[24] A round-bottom flask was charged with dipeptide **16** (300 mg, 0.303 mmol, 1 equiv) and palladium on carbon (Pd/C, 75 mg, 25 % *w*/*w* loading, dry matrix) then backfilled with argon gas. Methanol/tetrahydrofuran (8.0 mL, 10:8 *v*/*v*) was added and the reaction mixture was purged with dihydrogen gas for 5 min. The reaction mixture was stirred under an atmosphere of dihydrogen (1 atm; balloon) for 3 h at 23 °C. The product mixture was filtered through a pad of celite and was further washed with methanol (2 × 5 mL). The filtrate was concentrated, and the residue was purified by flash column chromatography (eluting with 100% dichloromethane initially, grading to 10% methanol–dichloromethane + 0.1 % triethylamine, linear gradient) to afford *amino alcohol* **S11** as a white solid (245 mg, 84%).

R_f = 0.50 (10% methanol–dichloromethane + 0.1% Et_3N; UV)

$[\alpha]_D^{20}$: –4.7 (*c* 0.60, $CHCl_3$)

^{1}H NMR (500 MHz, DMSO-d_6) δ 11.49 (s, 1H, H_{25}), 8.26 (t, J = 5.6 Hz, 1H, H_{26}), 7.98 (d, J = 8.2 Hz, 1H, H_{28}), 7.93 (t, J = 5.7 Hz, 1H, H_{29}), 7.88 (d, J = 7.5 Hz, 2H, H_{21}), 7.68 (dd, J = 7.5, 5.4 Hz, 2H, H_{18}), 7.40 (tt, J = 7.3, 1.3 Hz, 2H, H_{20}), 7.32 (dtd, J = 7.5, 3.9, 2.1 Hz, 2H, H_{19}), 7.11 (d, J = 8.8 Hz, 1H, H_{27}), 6.75 (t, J = 5.9 Hz, 1H, H_{30}), 4.92 (br s, 1H, –O*H*), 4.41 – 4.27 (m, 1H, H_{17}), 4.26 – 4.09 (m, 3H, 1 × H_9, 2 × H_{16}), 3.53 (app br s, 1H, H_7), 3.34* (br s, 1H, H_4), 3.26 (app d, J = 5.8 Hz, 2H, H_1), 3.22 (d, J = 6.0 Hz, 1H, H_8), 3.00 (app d, J = 12.2 Hz, 2H, H_{12}), 2.88 (t, J = 5.9 Hz, 2H, H_{15}), 1.57 (dt, J = 13.4, 6.4 Hz, 2H, 1 × H_{10}, 1 × H_{11}), 1.46 (s, 14H, 2 × H_2, 2 × H_3, 1 × H_{10}, 9 × H_{22}), 1.38 (s, 10H, 1 × H_2, 9 × H_{22}), 1.35 (s, 12H, 1 × H_2, 2 × H_3, 9 × H_{23}), 1.33 (s, 4H, 2 × H_{13}, 2 × H_{14}), 0.83 (dd, J = 18.3, 6.5 Hz, 6H, H_{24}). *Signal overlaps with residual H_2O peak and was assigned using HSQC analysis.

^{13}C NMR (126 MHz, DMSO-d_6) δ 171.7 (C), 163.2 (C), 155.9 (C), 155.6 (C), 155.2 (C), 152.1 (C), 144.0 (C), 143.8 (C), 140.7 (2 × C), 127.6 (2 × CH), 127.0 (2 × CH), 125.2 (2 × CH), 120.1 (2 × CH), 82.9 (C), 78.1 (C), 77.3 (C), 72.3 (CH), 65.1 (CH_2), 59.2 (CH), 50.9 (2 × CH), 46.9 (CH), 41.1 (CH_2), 40.3 (CH_2), 38.3 (CH_2), 31.7 (CH_2), 31.0 (CH_2), 28.6 (CH_2), 28.3 (3 × CH_3), 28.0 (3 × CH_3), 27.6 (3 × CH_3), 26.9 (CH_2), 26.4 (CH_2), 25.4 (CH_2), 24.2 (CH), 23.0 (CH_3), 21.5 (CH_3). One carbon obscured by DMSO-d_6 (CH_2). One quaternary carbon not observed.

HRMS-CI (m/z): $[M + H]^+$ calcd for $C_{50}H_{79}N_8O_{11}$, 967.5863; found, 967.5810.

*Synthesis of the protected rhabdobranin **4**.*

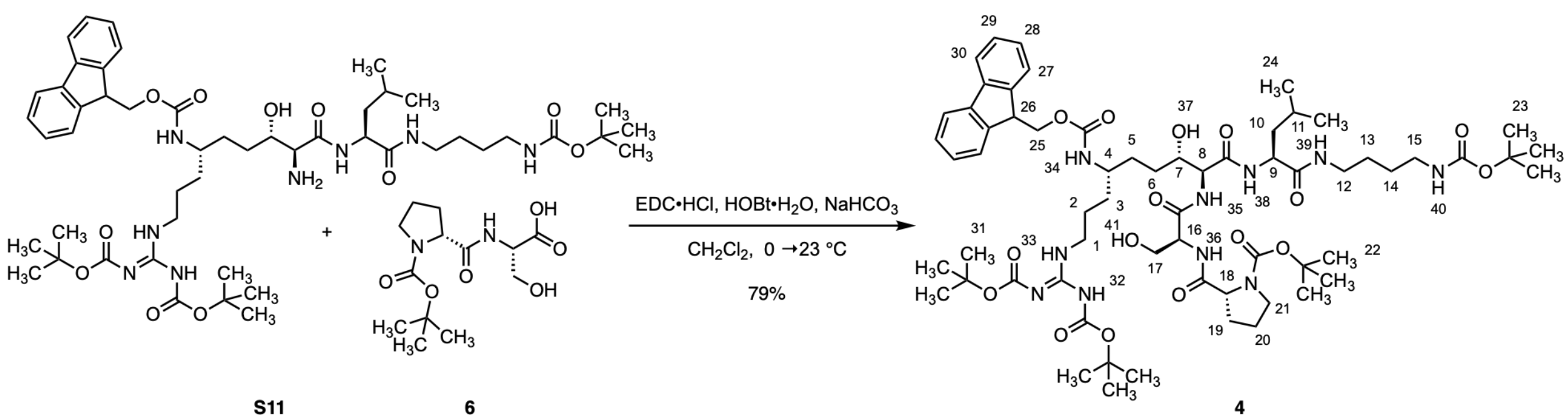

The following procedure has been adapted from the literature.[12] To a solution of amino alcohol **S11** (250 mg, 0.258 mmol, 1 equiv) in dichloromethane (3.1 mL) were added sodium hydrogen carbonate (26.5 mg, 0.315 mmol, 1.22 equiv), 1-hydroxybenzotriazole hydrate (HOBt•H_2O, 68.1 mg, 0.445 mmol, 1.72 equiv) and Boc-D-Pro-Ser **6** (102 mg, 0.336 mmol, 1.30 equiv) at 23 °C. The reaction mixture was cooled to 0 °C and 1-ethyl-3-(3-dimethylaminopropyl) carbodiimide hydrogen chloride (EDC•HCl, 60.5 mg, 0.315 mmol, 1.22 equiv) was added. The cooling bath was removed, and the reaction mixture was stirred for 16 h at 23 °C. The product mixture was diluted with brine (3.1 mL). The diluted product mixture was transferred to a separatory funnel and the layers that formed were separated. The aqueous phase was extracted with dichloromethane (3 × 5 mL). The combined organic extracts were dried over sodium sulfate. The dried solution was filtered, and the filtrate was concentrated. The residue was purified by flash column chromatography (eluting with 100% dichloromethane initially, grading to 10 % methanol–dichloromethane, linear gradient) to afford the *protected nominal rhabdobranin* **4** as a white solid (256 mg, 79%).

Note: For compounds containing the Boc-Pro-Ser fragment, several NMR resonances were broad or partially resolved. These features are consistent with slow conformational exchange of Boc amino acid rotamers on the NMR timescale; therefore, overlapping or broadened resonances are reported as observed without assigning individual rotameric species.[14]

R_f = 0.31 (5% methanol–dichloromethane; UV)

$[\alpha]_D^{20}$: +2.0 (*c* 0.80, CH_3OH)

^{1}H NMR (500 MHz, DMF-d_7) δ 11.66 (s, 1H, H_{32}), 8.39 (t, J = 5.6 Hz, 1H, H_{33}), 8.13 (d, J = 26.0 Hz, 1H, H_{35}), 7.94 (d, J = 7.6 Hz, 4H, 2 × H_{30}, 1 × H_{36}, 1 × H_{38}), 7.75 (t, J = 7.0 Hz, 2H, H_{27}), 7.70 (t, J = 5.7 Hz, 1H, H_{39}), 7.47 – 7.42 (m, 2H, H_{29}), 7.36 (tt, J = 7.4, 1.6 Hz, 2H, H_{28}), 7.14 (dd, J = 8.9, 3.0 Hz, 1H, H_{34}), 6.67 (d, J = 5.7 Hz, 1H, H_{40}), 5.42 (br s, 1H, H_{37} or H_{41}), 4.56 (br s, 1H, H_{37} or H_{41}), 4.36 (td, J = 12.4, 6.4 Hz, 4H, 1 × H_9, 1 × H_{16}, 2 × H_{25}), 4.29 – 4.19 (m, 3H, 1 × H_8, 1 × H_{18}, 1 × H_{26}), 3.93 – 3.71 (m, 3H, 1 × H_7, 2 × H_{17}), 3.51† (s, 1H, H_4), 3.45 – 3.31 (m, 4H, 2 × H_1, 2 × H_{21}), 3.14 (d, J = 6.2 Hz, 2H, H_{12}), 3.04 (q, J = 6.2 Hz, 2H, H_{15}), 2.22 – 2.14 (m, 1H, 1 × H_{20}), 2.22 – 2.14 (m, 2H, 1 × H_{20}, 1 × H_{19}), 2.01 – 1.93 (m, 1H, H_{19}), 1.79 (m, 2H, 1 × H_{19}, 1 × H_{11}), 1.67 – 1.56 (m, 6H, 2 × H_2, 2 × H_3, 2 × H_{10}), 1.51 (s, 11H, 2 × H_2, 9 × H_{31}), 1.43 (s, 16H, 2 × H_3,

2 × H_{13}, 3 × H_{22}, 9 × H_{31}), 1.39 (s, 17H, 2 × H_{14}, 6 × H_{22}, 9 × H_{23}), 0.96 – 0.62 (m, 6H, H_{24}). †Signal overlaps with residual H_2O peak and was assigned using HSQC analysis.

^{13}C NMR (126 MHz, DMF-*d*$_7$) δ 174.1 (C), 173.2 (C), 164.8 (C), 157.4 (C), 157.1 (C), 156.9 (C), 153.8 (C), 145.5 (C), 145.3 (C), 142.2 (2 × C), 128.7 (2 × CH), 128.2 (2 × CH), 126.5 (2 × CH), 121.1 (2 × CH), 84.1 (C), 80.2 (C), 79.3 (C), 78.5 (C), 72.6* (CH), 66.9 (CH_2), 63.3* (CH_2), 61.4* (CH), 59.2* (CH), 55.8 (CH), 53.1 (CH), 52.3 (CH), 48.3 (CH), 47.8* (CH_2), 41.7 (CH_2), 41.6 (CH_2), 41.0 (CH_2), 39.8 (CH_2), 33.3 (CH_2), 32.2 (CH_2), 29.0 (4 × CH_3), 28.8 (3 × CH_3), 28.4 (5 × CH_3), 28.3 (CH_2), 27.7 (CH_2), 26.9 (CH_2), 25.6 (CH), 25.3 (CH_2), 24.5 (CH_2), 23.9 (CH_3), 21.8 (CH_3). Three quaternary carbons not observed. One carbon obscured by DMF- *d*$_7$ (CH_2). *Assigned using HSQC analysis.

HRMS-CI (m/z): $[M + H]^+$ calcd for $C_{63}H_{99}N_{10}O_{16}$, 1251.7235; found, 1251.7213.

Achiral SFC chromatogram trace of compound **4**.

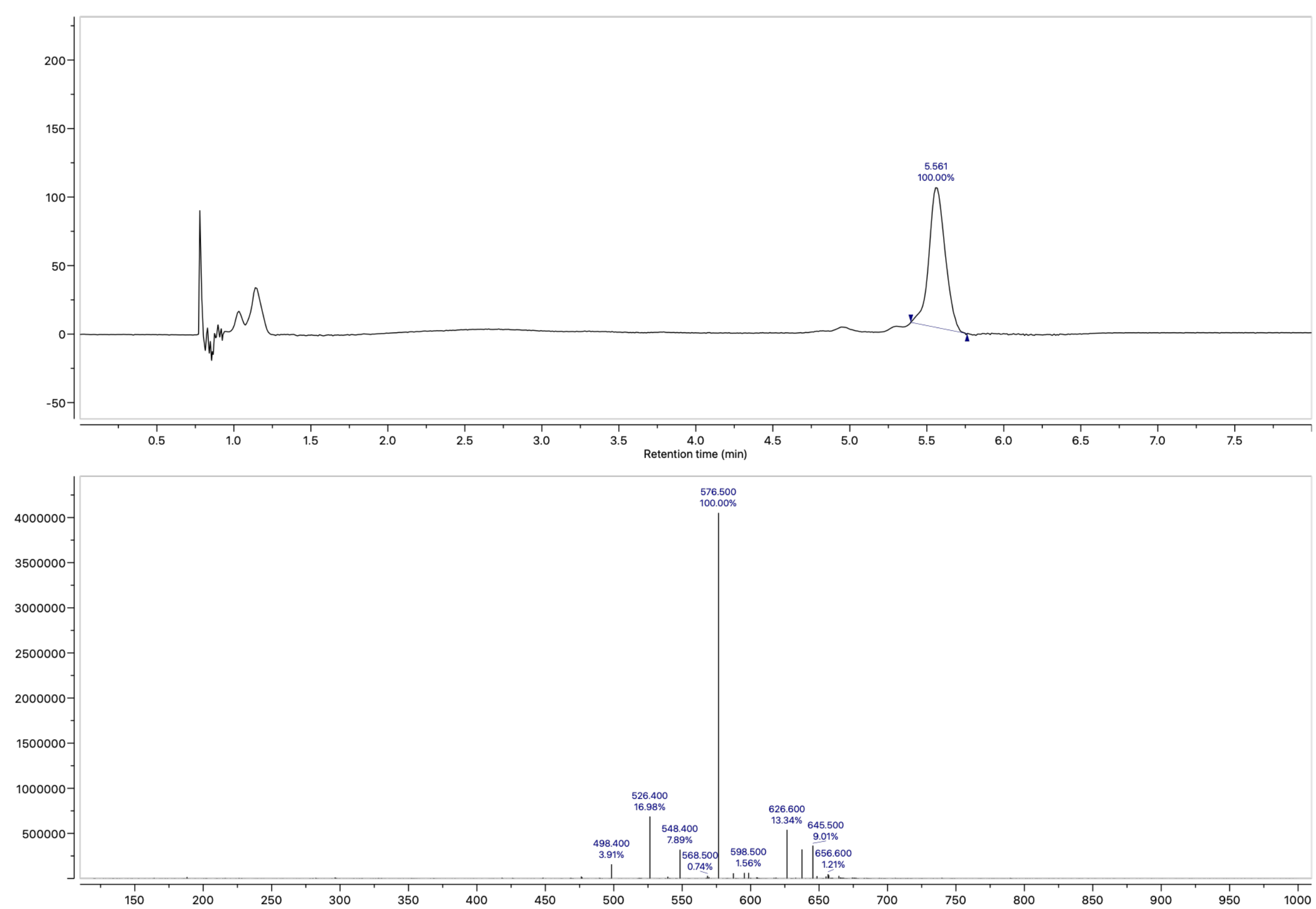

Column type: Agilent ZORBAX RX-SIL 4.6 × 150 mm, 5 µm.

Flow rate: 2 mL/min

Method: Gradient

Time (min)	CO_2 (%)	Methanol (%)
0.00	95.0	5.0
0.05	95.0	5.0
4.00	80.0	20.0
4.50	80.0	20.0
5.00	95.0	5.0
8.00	95.0	5.0

Retention time: 5.56 min at λ = 254 nm.

Observed masses: 626 [M/2 + H^+] and 576 [(M–Boc)/2 + H^+].

*Synthesis of the free amine **17**.*

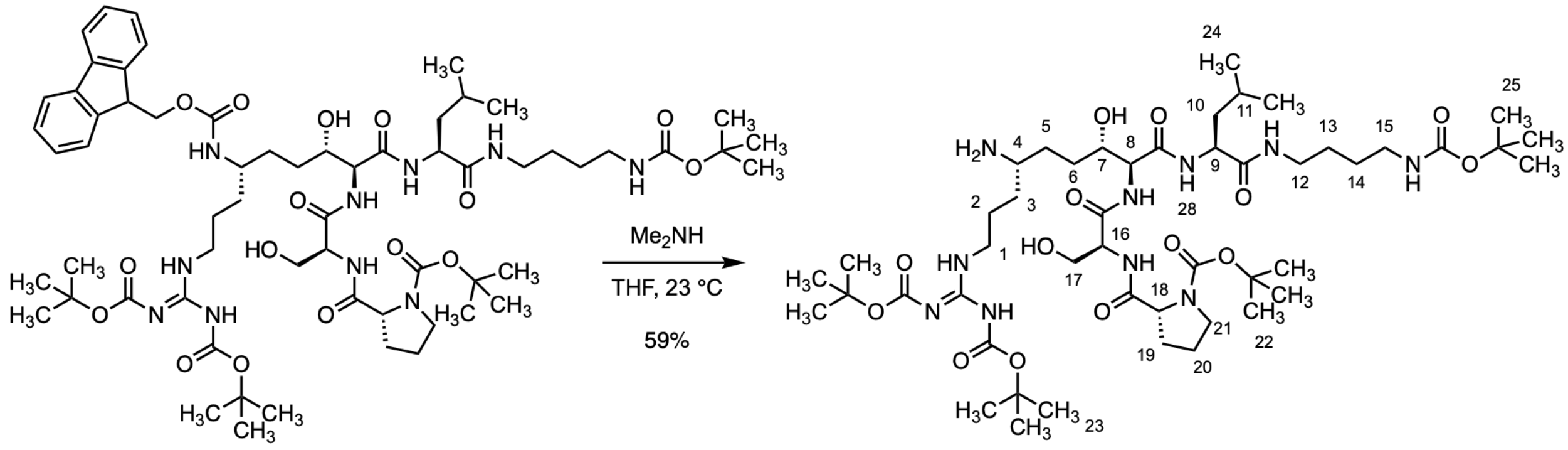

4 **17**

To a solution of protected nominal rhabdobranin **4** (70.0 mg, 56.0 μmol, 1 equiv) in tetrahydrofuran (1.4 mL, C = 0.040 M) was added dimethylamine (2.0 M solution in tetrahydrofuran, 0.560 mL, 1.12 mmol, 20.0 equiv). The reaction mixture was stirred for 5 h at 23 °C. The product mixture was concentrated, and the residue was purified by flash column chromatography (eluting with 100% dichloromethane initially, grading to 25 % methanol–dichloromethane, linear gradient) to afford the *free amine* **17** as a white solid (34.2 mg, 59%).

Note: For compounds containing the Boc-Pro-Ser fragment, several NMR resonances were broad or partially resolved. These features are consistent with slow conformational exchange of Boc amino acid rotamers on the NMR timescale; therefore, overlapping or broadened resonances are reported as observed without assigning individual rotameric species.[14]

R_f = 0.41 (10% methanol–dichloromethane; $KMnO_4$)

$[\alpha]_D^{20}$: –7.0 (*c* 0.74, CH_3OH)

^{1}H NMR (500 MHz, CD_3OD) δ 4.58 (s, 2H, –N*H₂*), 4.35 (m, 3H, 1 × H_8, 1 × H_9, 1 × H_{16}), 4.25 (dd, *J* = 8.4, 4.0 Hz, 1H, H_{18}), 4.00 – 3.70 (m, 3H, 1 × H_7, 2 ×H_{17}), 3.57 – 3.47 (m, 1H, 1 × H_{21}), 3.46 – 3.33 (m, 3H, 2 × H_1, 1 × H_{21}), 3.28 (app br s, 1H, H_4), 3.17 (d, *J* = 7.1 Hz, 2H, H_{12}), 3.04 (d, *J* = 7.0 Hz, 2H, H_{15}), 2.23 (app br s, 1H, 1 × H_{19}), 1.97 (app br s, 2H, 1 × H_{19}, 1 × H_{20}), 1.88 (app br s, 2H, 1 × H_{20}, 1 × H_2), 1.71 (app t, *J* = 7.8 Hz, 5H, 1 × H_2, 2 × H_3, 1 × H_5, 1 × H_{11}), 1.65 – 1.55 (m, 5H, 1 × H_5, 2 × H_6, 2 × H_{10}), 1.53 (s, 11H, 2 × H_{13}, 9 × H_{23}), 1.47 (d, *J* = 3.5 Hz, 17H, 2 × H_{14}, 6 × H_{22}, 9 × H_{23}), 1.43 (s, 12H, 3 × H_{22}, 9 × H_{25}), 1.01 – 0.79 (m, 6H, H_{24}).

^{13}C NMR (126 MHz, CD_3OD) δ 175.9 (C), 174.7 (C), 164.3 (C), 158.6 (C), 157.8 (C), 154.1 (C), 84.6 (C), 81.7 (C), 80.7 (C), 79.9 (C), 72.1* (CH), 62.8* (CH_2), 61.7 (CH), 58.9* (CH), 53.6 (CH), 52.6 (CH), 48.0 (CH_2), 41.5 (CH_2), 41.0 (CH_2), 40.8 (CH_2), 40.1 (CH_2), 32.5 (CH_2), 31.4 (CH_2), 30.5 (CH_2), 29.9 (CH_2), 28.8 (4 × CH_3), 28.6 (3 × CH_3), 28.2 (5 × CH_3), 27.5 (CH_2), 25.9 (CH_2, CH), 25.5 (CH_2), 24.7 (CH_2), 23.6 (CH_3), 21.8 (CH_3). Three quaternary carbons not observed. *Assigned using HSQC analysis.
HRMS-CI (m/z): $[M + H]^+$ calcd for $C_{48}H_{89}N_{10}O_{14}$, 1029.6554; found, 1029.6534.

*Synthesis of the proposed rhabdobranin **2**.*

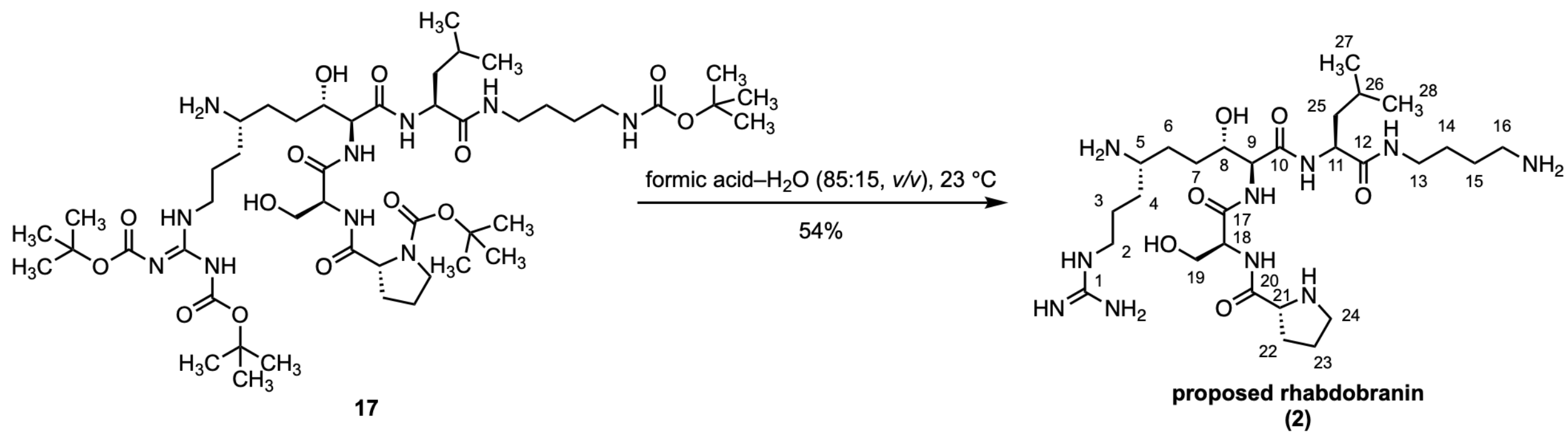

To free amine **17** (30.0 mg, 29.1 μmol, 1 equiv), was added 85% (*v*/*v*) aqueous formic acid solution (0.49 mL, C = 0.060 M). The reaction mixture was stirred for 24 h at 23 °C. The product mixture was lyophilized, diluted with methanol, and purified by reverse-phase HPLC with a HILIC column (XBridge BEH amide, 130Å, 5 μm, 10 mm × 250 mm) using an acetonitrile–water gradient (+0.1% formic acid) for 0–25 min, 94–60%, 3 $mLmin^{-1}$ to afford the *proposed rhabdobranin* **2** as a deliquescent white solid (10 mg, 54%).

Method:

0–2.0 min, 94% acetonitrile;
2.0–19.0 min, 94%–60%;
19.0–19.5 min, 60%–5%;
19.5–20.0 min, 5%;
20.0-–20.5min, 5%–94%;
20.5–25.0, 94%.

Retention time: 13.5 min.

$[\alpha]_D^{20}$: –5.0 (*c* 0.37, CH_3OH)

^{1}H NMR (500 MHz, D_2O) δ 4.47 (t, *J* = 5.6 Hz, 1H, H_{18}), 4.41 (app br s, 1H, H_{21}), 4.37 (d, *J* = 6.9 Hz, 1H, H_9), 4.24 (dd, *J* = 9.8, 4.9 Hz, 1H, H_{11}), 3.93 – 3.86 (m, 1H, H_8), 3.86 – 3.76 (m, 2H, H_{19}), 3.37 (dq, *J* = 12.9, 5.5 Hz, 2H, H_{24}), 3.28 (app br s, 1H, H_5), 3.17 (qq, *J* = 13.6, 5.9 Hz, 4H, 2 × H_2, 2 × H_{13}), 2.94 (t, *J* = 7.4 Hz, 2H, H_{16}), 2.43 (q, *J* = 7.6 Hz, 1H, 1 × H_{22}), 2.02 (dd, *J* = 6.4, 3.4 Hz, 3H, 1 × H_{22}, 2 × H_{23}), 1.83 (m, 1H, 1 × H_6), 1.75 – 1.42 (m, 14H, 2 × H_3, 2 × H_4, 1 × H_6, 2 × H_7, 2 × H_{14}, 2 × H_{15}, 2 × H_{25}, 1 × H_{26}), 0.86 (d, *J* = 5.6 Hz, 3H, H_{28}), 0.81 (d, *J* = 5.6 Hz, 3H, H_{27}).

^{13}C NMR (126 MHz, D_2O) δ 174.2 (C), 171.5 (C), 171.4 (C), 170.0 (C), 156.7 (C), 70.2 (CH), 61.1 (CH_2), 59.8 (CH), 58.0 (CH), 55.6 (CH), 52.7 (CH), 51.3 (CH), 46.6 (CH_2), 40.7 (CH_2), 39.7 (CH_2), 39.0 (CH_2), 38.6 (CH_2), 29.8 (CH_2), 28.8 (CH_2), 28.4 (CH_2), 28.1 (CH_2), 25.4 (CH_2), 24.3 (CH), 24.1 (CH_2), 23.8 (CH_2), 23.7 (CH_2), 22.1 (CH_3), 20.6 (CH_3).

HRMS-CI (m/z): $[M + H]^+$ calcd for $C_{28}H_{57}N_{10}O_6$, 629.4457; found, 629.4410.

Synthetic Procedures of Nominal Pre-rhabdobranin B.

*Synthesis of the Myr-D-Asn azido alcohol **18**.*

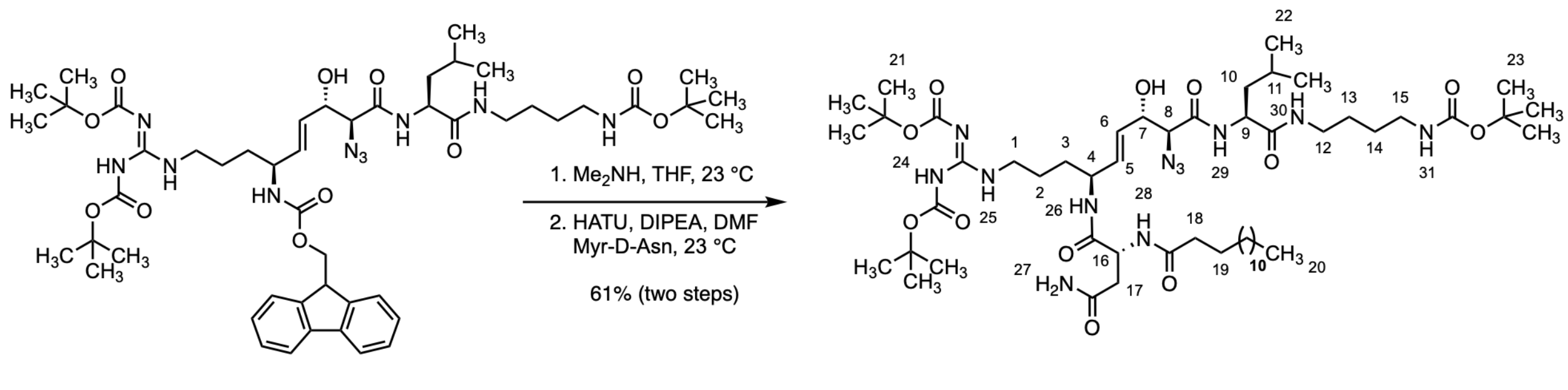

To a solution of dipeptide **16** (300 mg, 0.303 mmol, 1 equiv) in tetrahydrofuran (7.4 mL, C = 0.040 M) was added dimethylamine (2.0 M solution in tetrahydrofuran, 3.00 mL, 6.05 mmol, 20.0 equiv) and stirred for 5 h at 23 °C. The product mixture was concentrated and dried under high vacuum to afford a brown-yellow foam which was used in the next step without further purification. To a solution of the crude material obtained above (nominally 0.303 mmol, 1 equiv) in *N*,*N*-dimethylformamide (16 mL, C = 0.020 M) were sequentially added myristic acid-D-asparagine peptide (Myr-D-Asn, 319 mg, 0.932 mmol, 3.08 equiv), hexafluorophosphate azabenzotriazole tetramethyluronium (HATU, 295 mg, 0.777 mmol, 2.56 equiv) and *N*,*N*-diisopropylethylamine (DIPEA, 162 μL, 0.932 mmol, 3.08 equiv). The reaction mixture was stirred for 2 h at 23 °C. The product mixture was diluted with ethyl acetate (50 mL) and quenched with 1.0 M aqueous hydrogen chloride solution (20 mL). The quenched product mixture was transferred to a separatory funnel and the layers that formed were separated. The organic layer was washed with water (5 × 20 mL) and a saturated aqueous sodium chloride solution (20 mL). The combined organic layers were dried over sodium sulfate. The dried solution was filtered, and the filtrate was concentrated. The residue was purified by flash column chromatography (washed with 100 % ethyl acetate initially, then eluting with 0 → 10 % methanol–dichloromethane, linear gradient) to afford the *Myr-D-Asn azido alcohol* **18** as an off-white solid film (203 mg, 61%).
Note: the residue was concentrated again from 10% ethyl acetate–hexane (4 mL) to induce precipitation.

R_f = 0.29 (5% methanol–dichloromethane; UV)

$[\alpha]_D^{20}$: +1.0 (*c* 0.42, CH_3OH)

^{1}H NMR (500 MHz, DMSO-d_6) δ 11.49 (s, 1H, H_{24}), 8.25 (t, *J* = 5.6 Hz, 1H, H_{25}), 8.16 (d, *J* = 8.3 Hz, 1H, H_{29}), 7.88 (d, *J* = 8.0 Hz, 1H, H_{28}), 7.83 (t, *J* = 5.6 Hz, 1H, H_{30}), 7.77 (d, *J* = 8.6 Hz, 1H, H_{26}), 7.25 (d, *J* = 2.4 Hz, 1H, 1 × H_{27}), 6.84 (d, *J* = 2.4 Hz, 1H, 1 × H_{27}), 6.74 (t, *J* = 5.8 Hz, 1H, H_{31}), 5.79 (d, *J* = 4.5 Hz, 1H, –O*H*), 5.74 – 5.69 (m, 1H, H_5), 5.55 (dd, *J* = 15.1, 8.4 Hz, 1H, H_6), 4.54 (td, *J* = 8.0, 5.9 Hz, 1H, H_{16}), 4.32 (t, *J* = 5.9 Hz, 1H, H_7), 4.25 (td, *J* = 9.0, 5.4 Hz, 2H, 1 × H_4, 1 × H_9), 3.71 (d, *J* = 7.5 Hz, 1H, H_8), 3.24 (q, *J* = 6.9 Hz, 2H, H_1), 2.99 (dt, *J* = 13.3, 6.6 Hz, 2H, H_{12}), 2.88 (d, *J* = 6.7 Hz, 2H, H_{15}), 2.46 (dd, *J* = 15.2, 5.9 Hz, 1H, 1 × H_{17}), 2.35 (dd, *J* = 15.1,

8.1 Hz, 1H, 1 × H_{17}), 2.08 (t, *J* = 7.5 Hz, 2H, H_{18}), 1.56 (td, *J* = 12.3, 5.7 Hz, 1H, H_{11}), 1.46 (s, 15H, 9 × H_{21}, 2 × H_{19}, 2 × H_2, 2 × H_3), 1.39 (s, 10H, 9 × H_{21}, 1 × H_{10}), 1.36 (s, 10H, 9 × H_{23}, 1 × H_{10}), 1.34 – 1.31 (m, 4H, 2 × H_{13}, 2 × H_{14}), 1.22 (d, *J* = 4.2 Hz, 20H, 10 × CH_2), 0.91 – 0.73 (m, 9H, 6 × H_{22}, 3 × H_{20}).

^{13}C NMR (126 MHz, DMSO-*d₆*) δ 172.1 (C), 171.4 (C), 171.2 (C), 170.4 (C), 167.8 (C), 163.1 (C), 155.6 (C), 155.2 (C), 152.1 (C), 133.2 (CH), 128.7 (CH), 82.9 (C), 78.1 (C), 77.3 (C), 71.2 (CH), 65.1 (CH), 51.2 (CH), 49.9 (CH), 49.4 (CH), 40.7 (CH_2), 38.4 (CH_2), 37.7 (CH_2), 35.2 (CH_2), 31.7 (CH_2), 31.3 (CH_2), 29.1 (4 × CH_2), 29.0 (2 × CH_2), 28.8 (CH_2), 28.7 (CH_2), 28.3 (3 × CH_3), 28.0 (3 × CH_3), 27.6 (3 × CH_3), 26.9 (CH_2), 26.4 (CH_2), 25.2 (CH_2), 25.0 (CH_2), 24.3 (CH), 23.0 (CH_3), 22.1 (CH_2), 21.4 (CH_3), 14.0 (CH_3). Two carbons obscured by DMSO-d_6 (2 × CH_2).

HRMS-CI (m/z): $[M + H]^+$ calcd for $C_{53}H_{97}N_{12}O_{12}$, 1093.7343; found, 1093.7346.

*Synthesis of the Myr-D-Asn amino alcohol **S12**.*

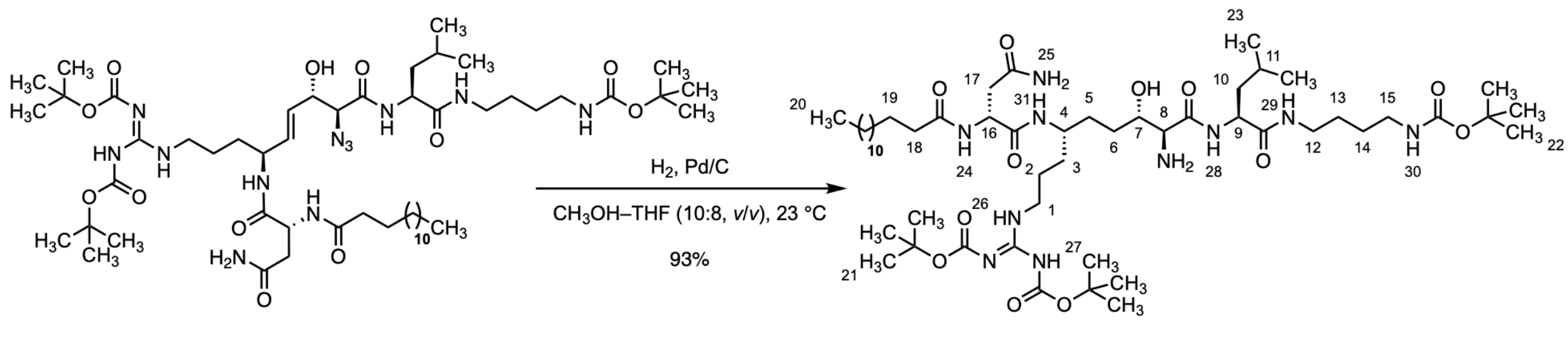

18 **S12**

The following procedure has been adapted from the literature.[24] A round-bottom flask was charged with Myr-D-Asn azido alcohol **18** (160 mg, 0.146 mmol, 1 equiv) and palladium on carbon (Pd/C, 40 mg, 25 % *w*/*w* loading, dry matrix) then backfilled with argon gas. Methanol/tetrahydrofuran (3.7 mL, 10:8 *v*/*v*) was added and the reaction mixture was purged with dihydrogen gas for 5 min. The reaction mixture was stirred under an atmosphere of dihydrogen (1 atm; balloon) for 3 h at 23 °C. The product mixture was filtered through a pad of celite and was further washed with methanol (2 × 5 mL). The filtrate was concentrated, and the residue was redissolved in 10% ethyl acetate–hexane (4 mL). The product mixture was concentrated again to afford *Myr-D-Asn amino alcohol* **S12** as a white solid film (145 mg, 93 %). The product obtained in this way was judged to be of >95% purity (^{1}H NMR analysis) and was advanced without purification.

R_f = 0.36 (5% methanol–dichloromethane + 0.1% Et_3N; UV, long streak)

$[\alpha]_D^{20}$: +8.3 (*c* 0.53, CH_3OH)

^{1}H NMR (500 MHz, DMSO-d_6) δ 11.49 (s, 1H, H_{27}), 8.23 (t, *J* = 5.6 Hz, 1H, H_{26}), 7.97 – 7.94 (m, 1H, H_{28}), 7.92 (t, *J* = 5.6 Hz, 1H, H_{29}), 7.87 (d, *J* = 7.8 Hz, 1H, H_{24}), 7.47 (d, *J* = 8.8 Hz, 1H, H_{31}), 7.27 (s, 1H, 1 × H_{25}), 6.90 – 6.83 (s, 1H, 1 × H_{25}), 6.75 (t, *J* = 5.8 Hz, 1H, H_{15}), 4.88 (s, 1H, –O*H*), 4.47 (td, *J* = 7.8, 5.9 Hz, 1H, H_{16}), 4.21 (m, 1H, H_9), 3.62 (m, 1H, H_4), 3.44 (m, 1H, H_7), 3.26 – 3.18 (m, 2H, H_1), 3.11 (d, *J* = 6.6 Hz, 1H, H_8), 3.00 (m, 2H, H_{12}), 2.96 – 2.84 (m, 2H, H_{15}), 2.45 (dd, *J* = 15.1, 6.0 Hz, 1H, 1 × H_{17}), 2.36 (dd, *J* = 15.1, 7.9 Hz, 1H, 1 × H_{17}), 2.08 (t, *J* = 7.5 Hz, 2H, H_{18}), 1.58 (dq, *J* = 13.4, 6.7 Hz, 1H, H_{11}), 1.47 (s, 15H, 2 × H_2, 2 × H_3, 2 × H_{10}, 9 × H_{21}), 1.39 (s, 10H, 1 × H_{19}, 9 × H_{21}), 1.36 (s, 10H, 1 × H_{19}, 9 × H_{22}), 1.32 (d, *J* = 3.9 Hz, 4H, 2 × H_{13}, 2 × H_{14}), 1.23 (s, 24 H, 2 × H_5, 2 × H_6, 20 × CH_2), 0.89 – 0.79 (m, 9H, 3 × H_{20}, 6 × H_{23}).

^{13}C NMR (126 MHz, DMSO-d_6) δ 173.6 (C), 172.1 (C), 171.8 (C), 171.5 (C), 170.6 (C), 163.1 (C), 155.6 (C), 155.2 (C), 152.1 (C), 82.9 (C), 78.1 (C), 77.3 (C), 73.0 (CH), 59.4 (CH), 50.9 (CH), 50.1 (CH), 48.5 (CH), 41.0 (CH_2), 40.2 (CH_2), 38.3 (CH_2), 37.7 (CH_2), 35.2 (CH_2), 31.3 (CH_2), 30.8 (CH_2), 29.1 (5 × CH_2), 29.0 (2 × CH_2), 28.9 (CH_2), 28.7 (2 × CH_2), 28.3 (3 × CH_3), 28.0 (3 × CH_3), 27.6 (3 × CH_3), 26.9 (CH_2), 26.4 (CH_2), 25.2 (CH_2), 25.1 (CH_2), 24.2 (CH), 23.1 (CH_3), 22.1 (CH_2), 21.5 (CH_3), 14.0 (CH_3). One carbon obscured by DMSO-d_6 (CH_2).

HRMS-CI (m/z): $[M + H]^+$ calcd for $C_{53}H_{101}N_{10}O_{12}$, 1069.7595; found, 1069.7565.

Synthesis of the protected pre-rhabdobranin B ***S13***.

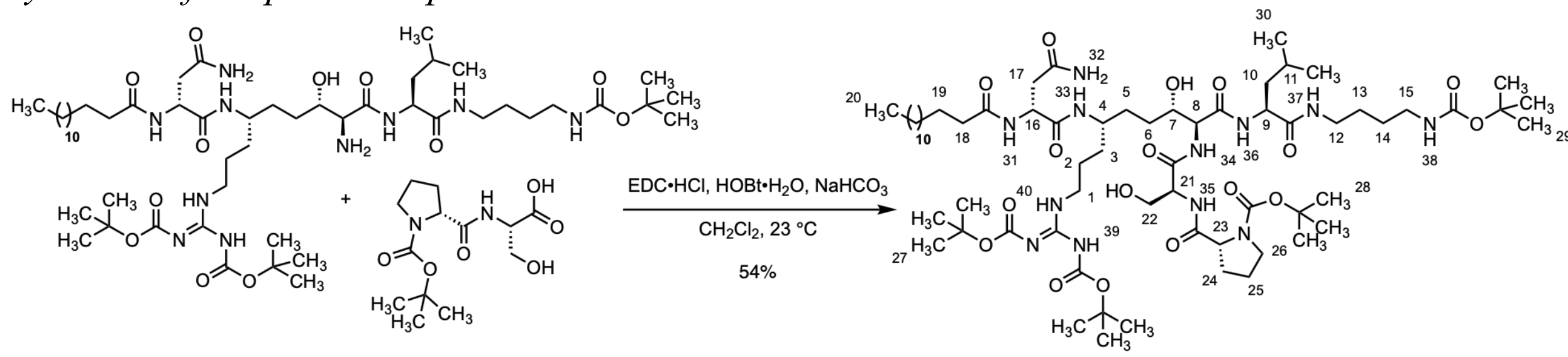

S12 **6** **S13**

The following procedure has been adapted from the literature.[12] To a solution of Myr-D-Asn amino alcohol **S12** (57.5 mg, 53.8 μmol, 1 equiv) in dichloromethane (0.67 mL, C = 0.080 M) were added sodium hydrogen carbonate (5.50 mg, 65.6 μmol, 1.22 equiv), 1-hydroxybenzotriazole hydrate (HOBt•H_2O, 14.2 mg, 92.5 μmol, 1.72 equiv) and Boc-D-Pro-Ser **6** (21.1 mg, 69.9 μmol, 1.30 equiv) at 23 °C. The reaction mixture was cooled to 0 °C and 1-ethyl-3-(3-dimethylaminopropyl) carbodiimide hydrogen chloride (EDC•HCl, 12.6 mg, 65.6 μmol, 1.22 equiv) was added. The cooling bath was removed, and the reaction mixture was stirred for 16 h at 23 °C. The product mixture was diluted with brine (1 mL). The diluted product mixture was transferred to a separatory funnel and the layers that formed were separated. The aqueous phase was extracted with dichloromethane (3 × 2 mL). The combined organic extracts were dried over sodium sulfate. The dried solution was filtered, and the filtrate was concentrated. The residue was purified by flash column chromatography (eluting with 100% dichloromethane initially, grading to 10 % methanol–dichloromethane, linear gradient) to afford the *protected pre-rhabdobranin B* **S13** as a white solid (39.2 mg, 54%).

Note: For compounds containing the Boc-Pro-Ser fragment, several NMR resonances were broad or partially resolved. These features are consistent with slow conformational exchange of Boc amino acid rotamers on the NMR timescale; therefore, overlapping or broadened resonances are reported as observed without assigning individual rotameric species.[14]

R_f = 0.40 (5% methanol–dichloromethane; $KMnO_4$)

$[\alpha]_D^{20}$: +4.7 (*c* 0.83, $CHCl_3$)

^{1}H NMR (600 MHz, DMF-d_7) δ 11.67 (s, 1H, H_{39}), 8.37 (app q, *J* = 6.6 Hz, 1H, H_{40}), 8.11 (d, *J* = 7.8 Hz, 1H, H_{31}), 7.70 (d, *J* = 27.8 Hz, 1H, H_{36}), 7.56 (s, 1H, 1 × H_{32}), 7.50 (d, *J* = 8.2 Hz, 1H), 6.95 (d, *J* = 2.7 Hz, 1H, 1 × H_{32}), 6.72 (app d, *J* = 5.8 Hz, 1H, H_{38}), 5.53 (d, *J* = 24.6 Hz, 1H, –CH_2O*H*, or –CHO*H*), 4.69 (d, *J* = 6.6 Hz, 1H, H_{16}), 4.54 – 4.47 (m, 1H, H_{21}), 4.41 – 4.20 (m, 3H, H_8, H_9, H_{23}), 3.92 – 3.87 (m, 1H, 1 × H_{22}), 3.83 – 3.77 (m, 2H, H_4, H_7), 3.71 (d, *J* = 8.1 Hz, 1H, 1 × H_{22}), 3.47 – 3.41 (m, 1H, 1 × H_{26}), 3.36 (q, *J* = 6.7 Hz, 3H, 2 × H_1, 1 × H_{26}), 3.13 (d, *J* = 4.0 Hz, 2H, H_{12}), 3.04 (d, *J* = 6.4 Hz, 2H, H_{15}), 2.66 (t, *J* = 7.2 Hz, 2H, H_{17}), 2.23 (d, *J* = 7.4 Hz, 2H, H_{18}), 2.00 – 1.93 (m, 2H), 1.81 (d, *J* = 2.7 Hz, 1H), 1.73 (dq, *J* = 12.6, 5.8 Hz, 2H), 1.58 (dddd, *J* = 16.6, 13.2, 7.2, 3.2 Hz, 7H), 1.51 (s, 8H), 1.44 (s, 18H), 1.40 (s, 15H), 1.27 (s, 22H), 0.92 – 0.80 (m, 9H, 3 × H_{20}, 6 × H_{29}).

Owing to substantial signal broadening, attributed primarily to conformational exchange associated with the four Boc-protecting groups, together with extensive signal overlap in this high-molecular-weight compound, unambiguous assignment of all resonances was not possible. Accordingly, only resonances that could be confidently assigned on the basis of COSY and HSQC correlations are reported; the remaining signals (H_2, H_3, H_5, H_6, H_{10}, H_{11}, H_{13}, H_{14}, H_{19}, H_{24}, H_{25}, H_{27}, H_{28}, H_{30}, H_{33}, H_{34}, H_{35}, and H_{37}) were left unassigned.

^{13}C NMR (126 MHz, DMF-d_7) δ 174.0 (C), 173.3 (C), 172.2 (C), 164.8 (C), 157.1 (C), 156.9 (C), 153.8 (C), 84.1 (C), 80.1 (C), 79.3 (C), 78.5 (C), 72.6 (CH), 63.1 (CH_2), 61.4 (CH), 59.7* (CH), 56.1 (CH), 53.2 (CH), 51.8 (CH), 50.1 (CH), 48.0 (CH_2), 46.8 (CH_2), 41.7 (CH_2), 41.6 (CH_2), 41.0 (CH_2), 39.7 (CH_2), 38.8 (CH_2), 36.8 (CH_2), 32.8 (CH_2), 32.0 (CH_2), 29.0 (5 × CH_3), 28.8 (4 × CH_3), 28.4 (3 × CH_3), 28.3 (CH_2), 27.7 (CH_2), 26.6 (CH_2), 25.6 (CH), 24.0 (CH_3), 23.5 (CH_2), 21.8 (CH_3), 14.7 (CH_3). Five quaternary carbons not observed. Twelve carbons obscured by DMF-d_7 or not clearly observed (CH_2). *Assigned using HSQC analysis.

HRMS-CI (m/z): $[M + H]^+$ calcd for $C_{66}H_{121}N_{12}O_{17}$, 1353.8967; found, 1353.8950.

Achiral SFC trace chromatogram of compound **S13**.

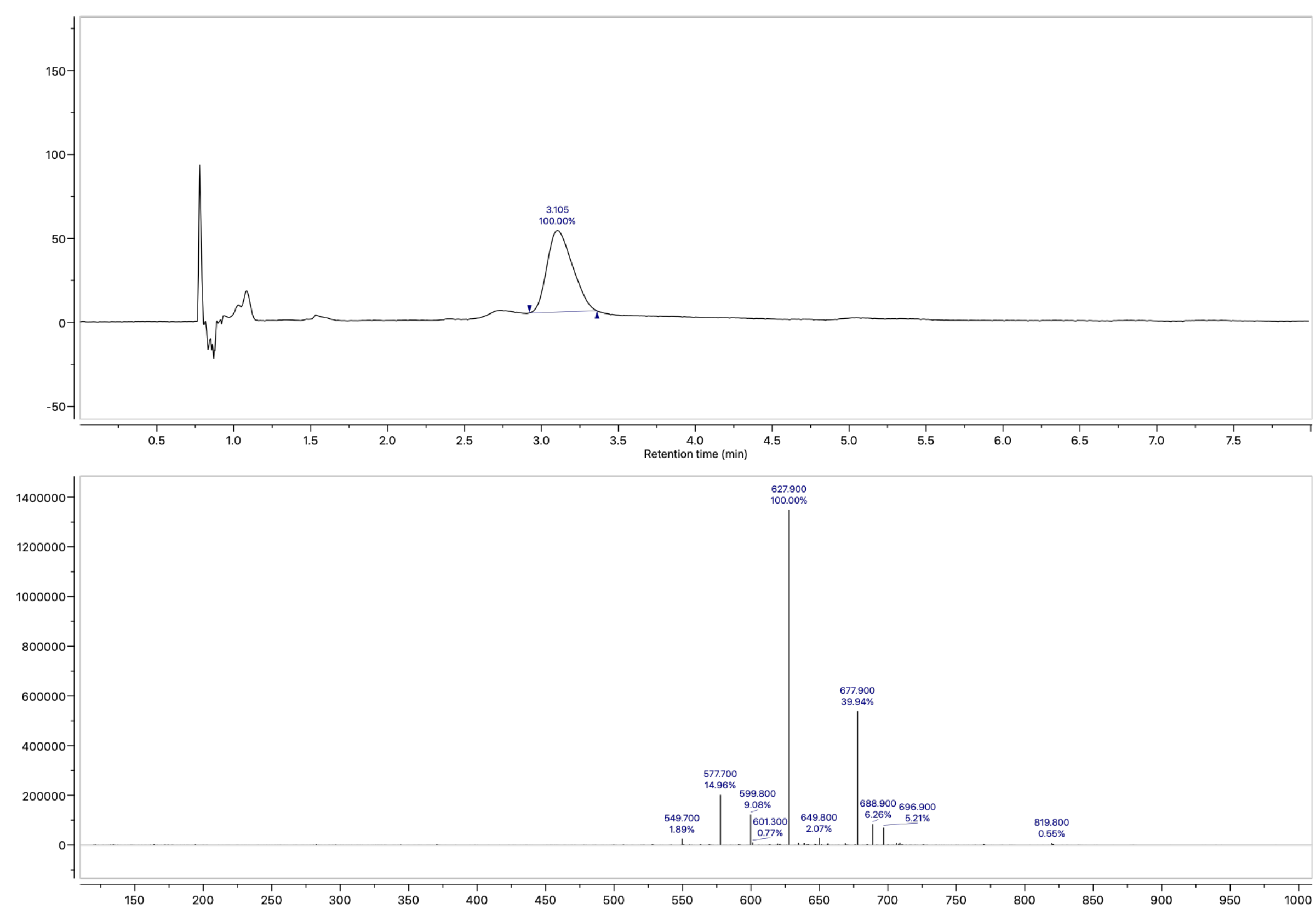

Column type: Agilent ZORBAX RX-SIL 4.6 × 150 mm, 5 µm.

Flow rate: 2 mL/min

Method: Isocratic

Time (min)	CO_2 (%)	Methanol (%)
0.00	80.0	20.0
8.00	80.0	20.0

Retention time: 3.11 min at λ = 254 nm.

Observed masses: 678 [M/2 + H^+] and 628 [(M–Boc)/2 + H^+].

*Synthesis of the proposed pre-rhabdobranin B **1**.*

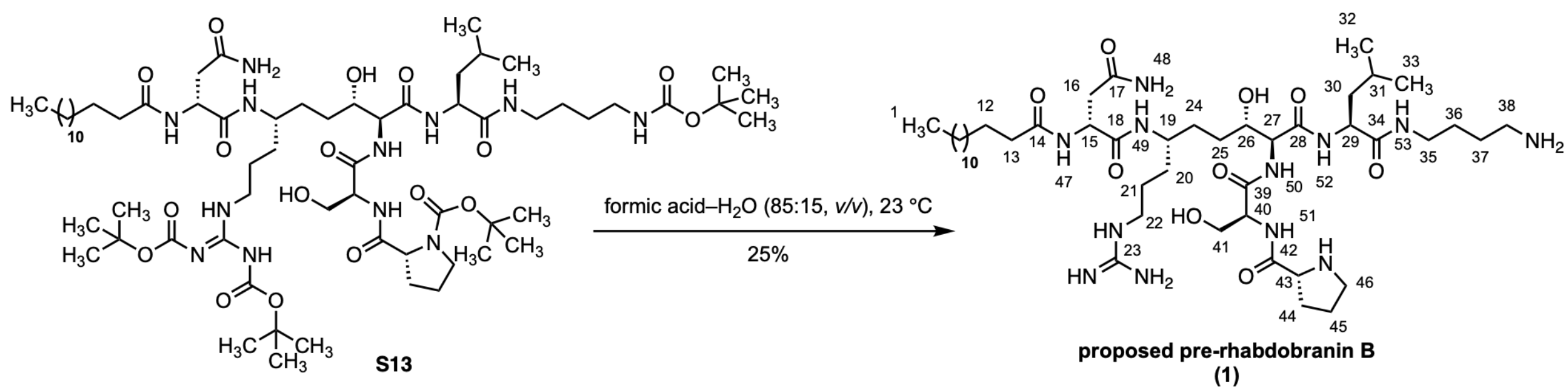

To protected pre-rhabdobranin B **S13** (35.0 mg, 25.9 μmol, 1 equiv), was added 85% (*v*/*v*) aqueous formic acid solution (0.43 mL, C = 0.060 M). The reaction mixture was stirred for 24 h at 23 °C. The product mixture was lyophilized, diluted with methanol, and purified by reverse-phase HPLC with an XBridge BE C18 column (130Å, 5 μm, 10 mm × 250 mm) using an acetonitrile–water gradient (+0.1% formic acid) for 0–30 min, 40–60%, 3 mLmin^{-1} to afford *proposed pre-rhabdobranin B* **1** as a deliquescent white solid (6.2 mg, 25%).

Method:

0–20.0 min, 40%–60% acetonitrile;
20.0–20.5 min, 60%–100%;
20.5–25.0 min, 100%;
25.0–25.5 min, 100%–40%;
25.5–30.0, 40%.

Retention time: 11.0 min.

***Note*:** The deprotection step using formic acid[25] proceeds quantitatively to the desired product. The low yield likely reflects the challenges associated with purifying a polycationic species with low UV activity. Nonetheless, as this procedure furnished sufficient material for complete spectroscopic characterization, the deprotection and purification sequence was not further optimized.

^{1}H NMR (500 MHz, DMSO-*d$_6$*) δ 8.34 (m, 1H, H_{51}), 8.27 (d, J = 7.4 Hz, 1H, H_{50}), 8.18 (d, J = 8.1 Hz, 1H, H_{52}), 8.09 (d, J = 8.1 Hz, 1H, H_{47}), 7.74 – 7.65 (m, 1H, H_{53}), 7.64 (d, J = 8.7 Hz, 1H, H_{49}), 7.42 (s, 1H, 1 × H_{48}), 6.89 (s, 1H, H_{48}), 4.49 (q, J = 7.2 Hz, 1H, H_{15}), 4.35 (d, J = 6.7 Hz, 1H, H_{40}), 4.19 (t, J = 7.1 Hz, 1H, H_{27}), 4.13 (q, J = 8.2 Hz, 1H, H_{29}), 3.12 – 2.92 (m, 4H, 2 × H_{22}, 2 × H_{35}), 2.88 (m, 1H, 1 × H_{46}), 2.74 (m, 3H, 2 × H_{38}, 1 × H_{46}), 2.49 – 2.42* (m, 1H, H_{16}), 2.38 (dd, J = 14.9, 8.0 Hz, 1H, H_{16}), 2.09 (t, J = 7.6 Hz, 2H, H_{13}), 1.95 (app q, J = 11.1 Hz, 1H, H_{44}), 1.69 (app q, J = 6.2 Hz, 1H, H_{44}), 1.64 – 1.57 (m, 3H, 1 × H_{31}, 1 × H_{36}, 1 × H_{45}), 1.52 – 1.30 (m, 18H, 2 × H_2, 2 × H_{12}, 2 × H_{20}, 2 × H_{21}, 2 × H_{24}, 2 × H_{25}, 2 × H_{30}, 1 × H_{36}, 2 × H_{37}, 1 × H_{45}), 1.23 (app d, J = 4.2 Hz, 20H, 10 × CH_2, myristoyl), 0.85 (app t, J = 6.6 Hz, 6H, 3 × H_1, 3 × H_{32} or H_{33}), 0.79 (d, J = 6.3 Hz, 3H, 3 × H_{32} or H_{33}).*Partially overlaps with residual H_2O peak.

^{13}C NMR (126 MHz, DMSO-d_6) δ 174.9 (C), 172.7 (C), 172.3 (C), 172.0 (C), 171.3 (C), 171.0 (C), 170.7 (C), 157.9 (C), 71.3 (CH), 62.4 (CH_2), 60.5 (CH), 58.4 (CH), 55.0 (CH), 52.0 (CH), 50.6 (CH), 48.7 (CH), 47.0 (CH_2), 41.0 (CH_2), 40.6 (CH_2), 38.9 (CH_2), 38.4 (CH_2), 38.2 (CH_2), 35.7 (CH_2), 31.8 (2 × CH_2), 31.1 (CH_2), 30.7 (CH_2), 29.8 (2 × CH_2), 29.5 (3 × CH_2), 29.4 (CH_2), 29.3 (CH_2), 29.2 (2 × CH_2), 26.2 (2 × CH_2), 25.7 (CH_2), 25.3 (CH_2), 24.6 (CH), 23.6 (CH_3), 22.6 (2 × CH_2), 21.5 (CH_3), 14.4 (CH_3).

HRMS-CI (m/z): $[M + H]^+$ calcd for $C_{46}H_{89}N_{12}O_9$, 953.6870; found, 953.6830.

Synthetic Procedures of Revised Pre-rhabdobranin B

Synthesis of the epi-bromo-aldol adduct ***S15***.

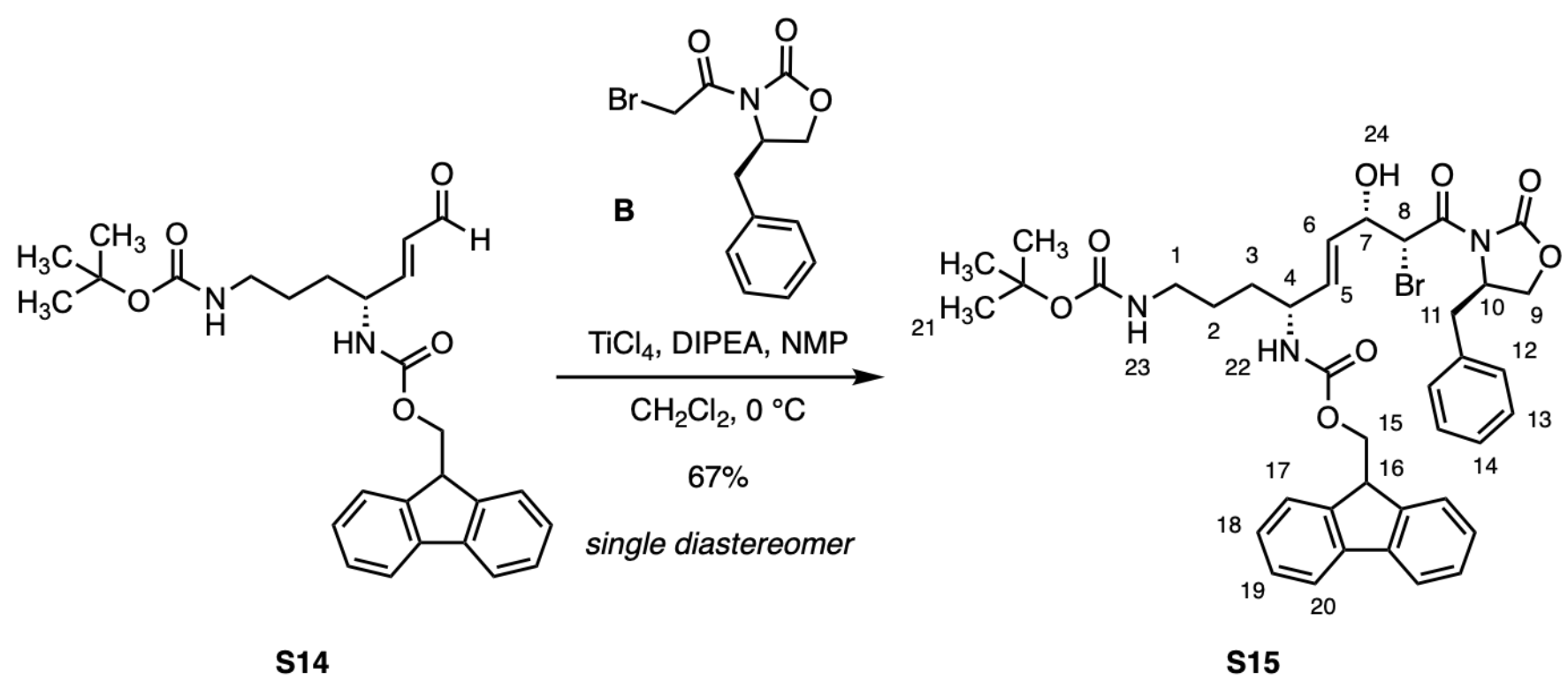

To a cooled (0 °C) solution of (*R*)-4-benzyl-3-(2-bromoacetyl)oxazolidin-2-one **B** (5.86 g, 19.7 mmol, 1.10 equiv) in dichloromethane (180 mL, C = 0.10 M) was added titanium (IV) chloride (1.0 M in dichloromethane, 20.5 mL, 20.5 mmol, 1.15 equiv). The reaction mixture was stirred for 15 min at 0 °C. To the dark red solution was added *N*,*N*-diisopropylethylamine (DIPEA, 3.70 mL, 21.4 mmol, 1.20 equiv) and the reaction mixture was stirred for 40 min at 0 °C. At this point the solution turned dark purple, and smoke was observed. To this solution was added *N*-methylpyrrolidone (1.90 mL, 19.7 mmol, 1.10 equiv) and the reaction mixture was stirred for an additional 10 min at 0 °C. A solution of *epi*-α,β-unsaturated aldehyde **S14** (8.30 g, 17.9 mmol, 1 equiv) in dichloromethane (50 mL, C = 0.36 M) was added dropwise into the titanium enolate solution. The reaction mixture was stirred for 2 h at 0 °C. The solution gradually turned orange during the course of the reaction. The product mixture was quenched with a half-saturated aqueous solution of ammonium chloride (220 mL). The quenched product mixture was transferred to a separatory funnel and the layers that formed were separated. The aqueous layer was extracted with dichloromethane (2 × 75 mL). The combined organic extracts were dried over sodium sulfate. The dried solution was filtered, and the filtrate was concentrated. The brown oily residue was purified by flash column chromatography (eluting with 30 % ethyl acetate–hexane initially, grading to 60 % ethyl acetate–hexane, linear gradient) to afford the *epi-bromo-aldol adduct* **S15** as a light yellow-brown solid (9.11 g, 67 %, *dr* >99:1).

Note: **S14** was prepared from D-ornithine hydrochloride following the same synthetic steps reported for α,β-unsaturated aldehyde **9**.

R_f = 0.43 (50% ethyl acetate–hexane; UV)

$[\alpha]_D^{20}$: –26.8 (*c* 1.0, $CHCl_3$)

^{1}H NMR (500 MHz, DMSO-d_6) δ 7.88 (d, *J* = 7.5 Hz, 2H), 7.67 (dd, *J* = 10.0, 7.5 Hz, 2H), 7.47 – 7.38 (m, 3H, 2 × H, 1 × H_{22}), 7.36 – 7.29 (m, 4H), 7.26 (td, *J* = 6.5, 1.7 Hz, 3H), 6.80 (t, *J* = 5.7 Hz, 1H, H_{23}), 5.77 (d, *J* = 6.5 Hz, 1H, H_{24}), 5.62 (dd, *J* = 15.4, 6.1 Hz, 1H, H_5), 5.53 (d, *J* = 8.7

Hz, 1H, H_8), 5.43 (dd, *J* = 15.5, 6.8 Hz, 1H, H_6), 4.65 (dq, *J* = 6.6, 3.4 Hz, 1H, H_{10}), 4.42 (t, *J* = 8.5 Hz, 1H, 1 × H_9), 4.36 (q, *J* = 7.2 Hz, 1H, H_7), 4.33 – 4.21 (m, 3H, 1 × H_9, 2 × H_{15}), 4.19 (q, *J* = 5.2 Hz, 1H, H_{16}), 3.88 (h, *J* = 7.8 Hz, 1H, H_4), 2.99 (qd, *J* = 13.6, 5.0 Hz, 2H, H_{11}), 2.88 (d, *J* = 6.2 Hz, 2H, H_1), 1.36 (s, 13H, 2 × H_2, 2 × H_3, 9 × H_{21}).

^{13}C NMR (126 MHz, DMSO-*d₆*) δ 167.5 (C), 155.6 (2 × C), 152.7 (C), 144.1 (C), 143.8 (C), 140.8 (2 × C), 135.3 (CH), 135.0 (C), 129.7 (2 × CH), 128.6 (2 × CH), 127.7 (2 × CH), 127.3 (CH), 127.1 (2 × CH), 125.2 (3 × CH), 120.2 (2 × CH), 77.4 (C), 71.6 (CH), 66.1 (CH_2), 65.2 (CH_2), 54.5 (CH), 51.9 (CH), 48.9 (CH), 46.8 (CH), 35.9 (CH_2), 31.3 (CH_2), 28.3 (3 × CH_3), 26.3 (CH_2). One carbon obscured by DMSO-d_6 (CH_2).

HRMS-CI (m/z): $[M + H]^+$ calcd for $C_{39}H_{45}BrN_3O_8$, 762.2385; found, 762.2408.

*Synthesis of the epi-azide **S16**.*

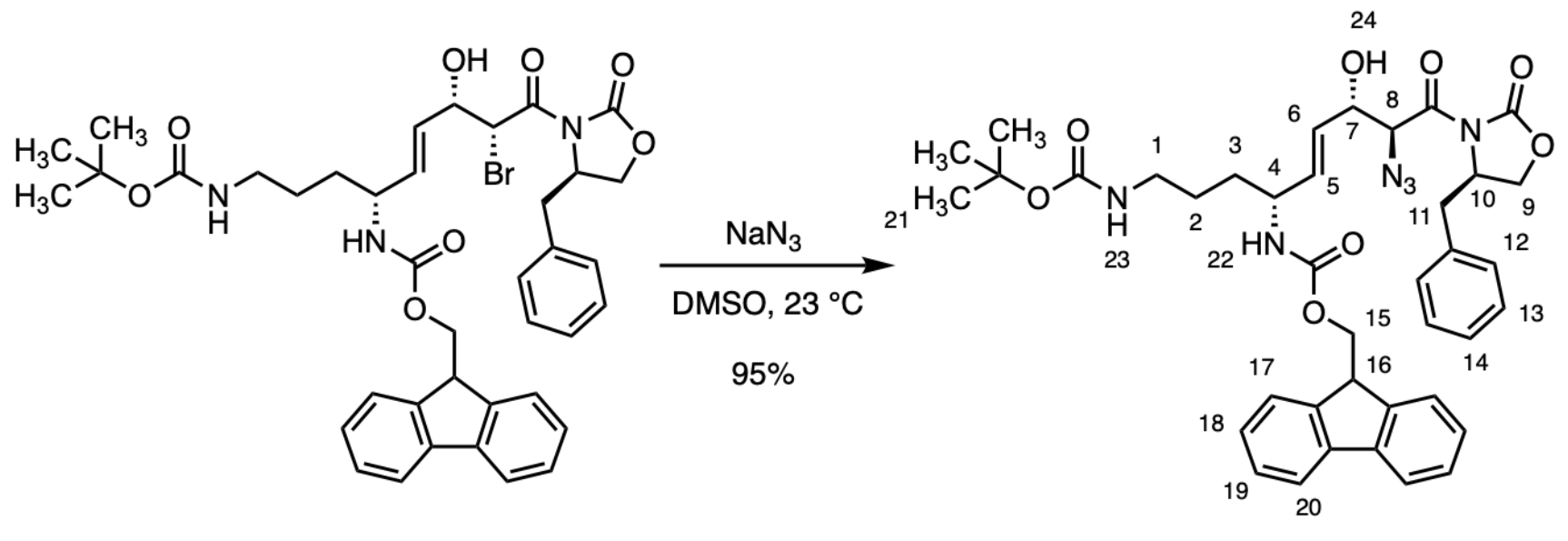

To a solution of *epi*-bromo-aldol adduct **S15** (8.60 g, 11.3 mmol, 1 equiv) in dimethylsulfoxide (40 mL, C = 0.28 M) was added sodium azide (1.47 g, 22.6 mmol, 2.00 equiv). The reaction mixture was stirred for 2 h at 23 °C. The resultant orange solution was diluted with 2 L of hexane/dichloromethane (2:1, *v*/*v*) and transferred to a separatory funnel. The diluted product mixture was washed with water (4 × 800 mL). The washed product mixture was dried over sodium sulfate. The dried solution was filtered, and the filtrate was concentrated. 35 mL of dichloromethane was added to the residue, and the solution was concentrated again to afford the *epi-azide* **S16** as a brown-yellow foamy solid (7.73 g, 95%). The product obtained in this way was judged to be of >95% purity (^{1}H NMR analysis) and was advanced without purification.

R_f = 0.44 (50% ethyl acetate–hexane; UV)

$[\alpha]_D^{20}$: –14.7 (*c* 1.0, $CHCl_3$)

^{1}H NMR (500 MHz, DMSO-d_6) δ 7.88 (d, *J* = 7.5 Hz, 2H), 7.68 (q, *J* = 8.0 Hz, 2H), 7.46 (d, *J* = 8.7 Hz, 1H, H_{22}), 7.41 (d, *J* = 7.5 Hz, 2H), 7.35 – 7.21 (m, 7H, 5 × $H_{12\text{-}14}$, 2 × H), 6.85 – 6.74 (m, 1H, H_{23}), 5.85 (d, *J* = 5.3 Hz, 1H, H_{24}), 5.81 – 5.70 (m, 1H, H_5), 5.64 (dd, *J* = 15.4, 6.9 Hz, 1H, H_6), 5.08 (d, *J* = 6.6 Hz, 1H, H_8), 4.71 (tt, *J* = 7.9, 3.4 Hz, 1H, H_{10}), 4.47 (q, *J* = 6.2 Hz, 1H, H_7), 4.36 (q, *J* = 9.3 Hz, 1H, 1 × H_9), 4.29 – 4.15 (m, 4H, 1 × H_9, 2 × H_{15}, 1 × H_{16}), 3.99 (t, *J* = 7.4 Hz, 1H, H_4), 3.10 – 2.97 (m, 1H, 1 × H_{11}), 2.87 (dq, *J* = 21.8, 7.4 Hz, 3H, 2 × H_1, 1 × H_{11}), 1.36 (s, 13H, 2 × H_2, 2 × H_3, 9 × H_{21}).

^{13}C NMR (126 MHz, DMSO-d_6) δ 168.4 (C), 155.6 (2 × C), 153.2 (C), 143.9 (2 × C), 140.8 (2 × C), 135.6 (CH), 134.4 (CH), 129.6 (2 × CH), 128.6 (2 × CH), 128.3 (CH), 127.6 (2 × CH), 127.1 (2 × CH), 127.0 (CH), 125.3 (2 × CH), 120.2 (2 × CH), 77.4 (C), 71.5 (CH), 66.4 (CH_2), 65.3 (CH_2), 63.1 (CH), 54.5 (CH), 52.0 (CH), 46.8 (CH), 36.6 (CH_2), 32.0 (CH_2), 28.3 (3 × CH_3), 26.3 (CH_2). One carbon obscured by DMSO-d_6 (CH_2).

HRMS-CI (m/z): $[M + H]^+$ calcd for $C_{39}H_{45}N_6O_8$, 725.3293; found, 725.3309.

*Synthesis of the epi-carboxylic acid **S17**.*

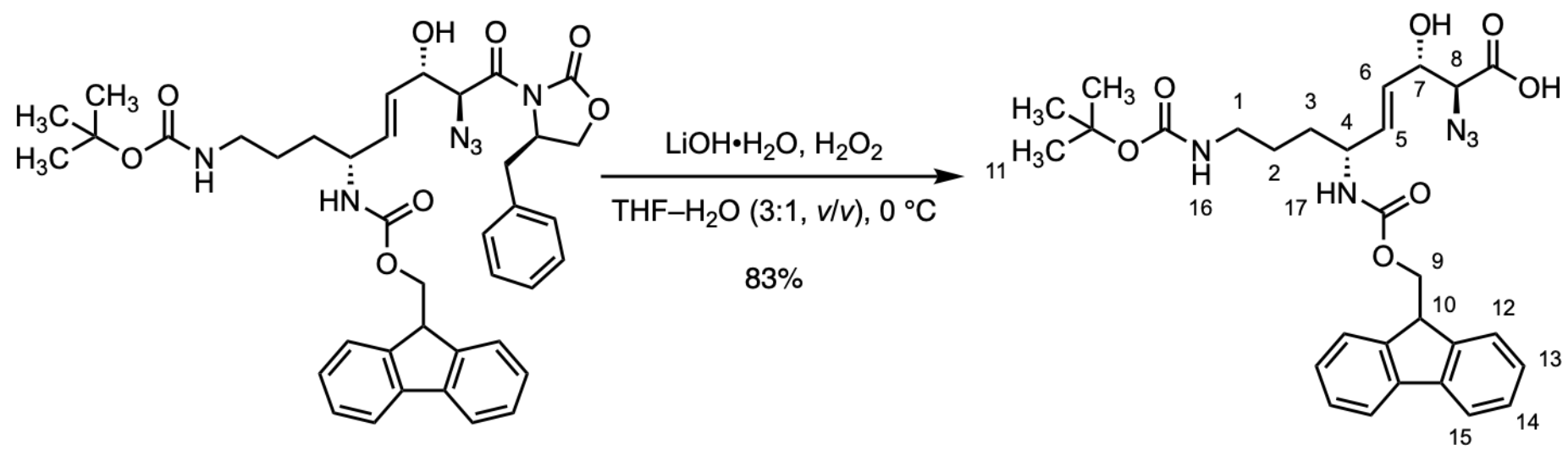

S16 **S17**

To a solution of *epi*-azide **S16** (7.50 g, 10.3 mmol, 1 equiv) in tetrahydrofuran/water (240 mL, 3:1 v/v) was added 30% hydrogen peroxide (4.90 mL, 62.7 mmol, 6.06 equiv) and lithium hydroxide monohydrate (0.868 g, 20.7 mmol, 2.00 equiv) at 0 °C. The reaction mixture was stirred for 2 h at 0 °C. The product mixture was quenched by the addition of 0.55 M aqueous sodium sulphite solution (130 mL) at 0 °C. The pH was adjusted to 9–10 with saturated aqueous sodium bicarbonate solution (25 mL). The quenched product mixture was partially concentrated and transferred to a separatory funnel. The aqueous phase was extracted with diethyl ether (3 × 125 mL) and discarded. The washed aqueous phase was acidified to pH 4 – 5 with a 1.0 M aqueous hydrogen chloride solution (100 mL) and extracted with ethyl acetate (3 × 125 mL). The combined organic extracts were dried over sodium sulfate. The dried solution was filtered, and the filtrate was concentrated to afford *epi-carboxylic acid* **S17** as a pale-brown solid (4.85 g, 83 %). The product obtained in this way was judged to be of >95% purity (^{1}H NMR analysis) and was advanced without purification. An analytical sample was prepared by flash column chromatography (eluting with 100 % dichloromethane initially, grading to 10% methanol–dichloromethane).

R_f = 0.30 (80% ethyl acetate–hexane + 0.1% acetic acid; UV, long streak)

$[\alpha]_D^{20}$: +3.0 (*c* 1.9, $CHCl_3$)

^{1}H NMR (500 MHz, DMSO-*d*$_6$) δ 7.89 (d, *J* = 7.7 Hz, 2H, H_{15}), 7.70 (d, *J* = 7.5 Hz, 2H, H_{12}), 7.41 (t, *J* = 7.4 Hz, 3H, 2 × H_{14}, 1 × H_{17}), 7.33 (tdd, *J* = 7.6, 3.4, 1.3 Hz, 2H, H_{13}), 6.80 (t, *J* = 5.7 Hz, 1H, H_{16}), 5.62 – 5.41 (m, 2H, H_5, H_6), 4.30 – 4.18 (m, 3H, 2 × H_9, 1 × H_{10}), 4.11 (t, *J* = 6.2 Hz, 1H, H_7), 3.92 (d, *J* = 7.4 Hz, 1H, H_4), 3.38* (m, 1H, H_8), 2.89 (q, *J* = 6.5 Hz, 2H, H_1), 1.36 (s, 13H, 2 × H_2, 2 × H_3, 9 × H_{11}). *Overlaps with H_2O signal.

^{13}C NMR (126 MHz, DMSO-*d*$_6$) δ 171.5 (C), 155.6 (2 × C), 144.0 (2 × C), 140.8 (2 × C), 132.0 (CH), 130.2 (CH), 127.6 (2 × CH), 127.1 (2 × CH), 125.3 (2 × CH), 120.2 (2 × CH), 77.4 (C), 71.5 (CH), 66.7 (CH), 65.3 (CH_2), 52.2 (CH), 46.8 (CH), 32.2 (CH_2), 28.3 (3 × CH_3), 26.3 (CH_2). One carbon obscured by DMSO-*d*$_6$ (CH_2).

HRMS-CI (m/z): $[M + H]^+$ calcd for $C_{29}H_{36}N_5O_7$, 566.2609; found, 566.2647.

*Synthesis of the epi-guanylated acid **S18**.*

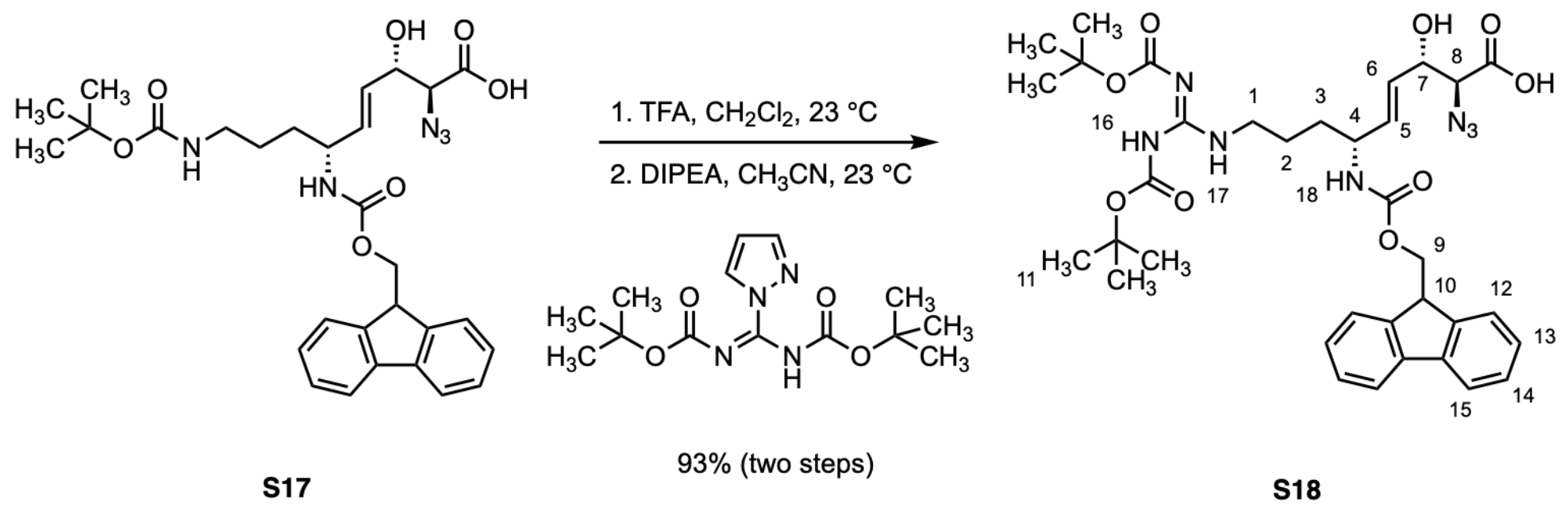

To a solution of *epi*-carboxylic acid **S17** (6.75 g, 11.9 mmol, 1 equiv) in dichloromethane (90 mL, C = 0.13 M) was added trifluoroacetic acid (9.1 mL, C = 1.3 M) at 23 °C. The reaction mixture was stirred for 2 h at 23 °C. The resulting dark orange solution was concentrated and redissolved in dichloromethane (50 mL). The solution was concentrated again to remove residual acid. The residue was subjected to high vacuum to afford a brown foamy gum. This material was used directly in the next step without further treatment.

To a solution of the crude product obtained above (nominally 11.9 mmol, 1 equiv) in acetonitrile (60 mL, C = 0.20 M) were added *N*,*N*-diisopropylethylamine (10.4 mL, 59.7 mmol, 5.00 equiv) and 1*H*-pyrazole-1-[*N*,*N'*-bis(*tert*-butoxycarbonyl)]-carboxamidine (4.45 g, 14.3 mmol, 1.20 equiv). The reaction mixture was stirred for 18 h at 23 °C. The product mixture was diluted with 60 mL of water. The diluted product mixture was acidified to pH 4 – 5 with 1.0 M aqueous hydrogen chloride solution (60 mL). The quenched product mixture was transferred to a separatory funnel and the layers that formed were separated. The aqueous phase was extracted with ethyl acetate (3 × 100 mL). The combined organic extracts were washed with water (3 × 120 mL) to remove residual pyrazole. The washed organic extract was dried over sodium sulfate. The dried solution was filtered, and the filtrate was concentrated. The residue was purified by flash column chromatography (eluting with 50 % ethyl acetate–hexane initially, grading to 80 % ethyl acetate–hexane, linear gradient) to afford the *epi-guanylated acid* **S18** as a deliquescent off-white solid (7.88 g, 93%).

Note 1: An analytical sample was prepared by repurification with 0 → 10% methanol–dichloromethane, linear gradient.
Note 2: Small shifts in the ^{1}H and ^{13}C-NMR were observed between different samples of compound **S18** (most likely due to the formation of zwitterions). The reported analytical data correspond to the shifts obtained after the second purification.
Note 3: The solution becomes viscous and produces fumes upon the addition of the base. The reaction mixture eventually homogenizes within 3 – 4 hours after the addition of the guanylating agent **C**.
Note 4: The product was stored in a –20 °C freezer to prevent decomposition and was consumed within a few weeks of preparation for best yields in the upcoming steps (*vide infra*).

R_f = 0.40 (80% ethyl acetate–hexane + 0.1% acetic acid; UV, long streak)

$[\alpha]_D^{20}$: +5.7 (*c* 1.0, $CHCl_3$)

^{1}H NMR (500 MHz, DMSO-d_6) δ 11.51 (s, 1H, H_{16}), 8.28 (app d, *J* = 5.8 Hz, 1H, H_{17}), 7.88 (d, *J* = 7.6 Hz, 2H, H_{15}), 7.70 (dd, *J* = 7.6, 3.7 Hz, 2H, H_{12}), 7.45 (d, *J* = 8.7 Hz, 1H, H_{18}), 7.40 (t, *J* = 7.4 Hz, 2H, H_{14}), 7.32 (dtd, *J* = 7.4, 4.4, 2.3 Hz, 2H, H_{13}), 5.67 – 5.51 (m, 2H, H_5, H_6), 4.33 – 4.12 (m, 4H, 1 × H_7, 2 × H_9, 1 × H_{10}), 4.00 – 3.91 (m, 1H, H_4), 3.64 (d, *J* = 6.5 Hz, 1H, H_8), 3.26 (p, *J* = 7.8 Hz, 2H, H_1), 1.46 (s, 13H, 2 × H_2, 2 × H_3, 9 × H_{11}), 1.38 (s, 9H, 9 × H_{11}).

^{13}C NMR (126 MHz, DMSO-d_6) δ 170.7 (C), 163.2 (C), 155.5 (C), 155.3 (C), 152.1 (C), 144.0 (C), 143.9 (C), 140.8 (2 × C), 132.5 (CH), 129.7 (CH), 127.6 (2 × CH), 127.1 (2 × CH), 125.3 (2 × CH), 120.2 (2 × CH), 82.9 (C), 78.2 (C), 71.7 (CH), 66.4 (CH), 65.3 (CH_2), 52.2 (CH), 46.8 (CH), 32.1 (CH_2), 28.0 (3 × CH_3), 27.7 (3 × CH_3), 25.4 (CH_2).

HRMS-CI (m/z): $[M + H]^+$ calcd for $C_{35}H_{46}N_7O_9$, 708.3352; found, 708.3384.

*Synthesis of the epi-dipeptide **19**.*

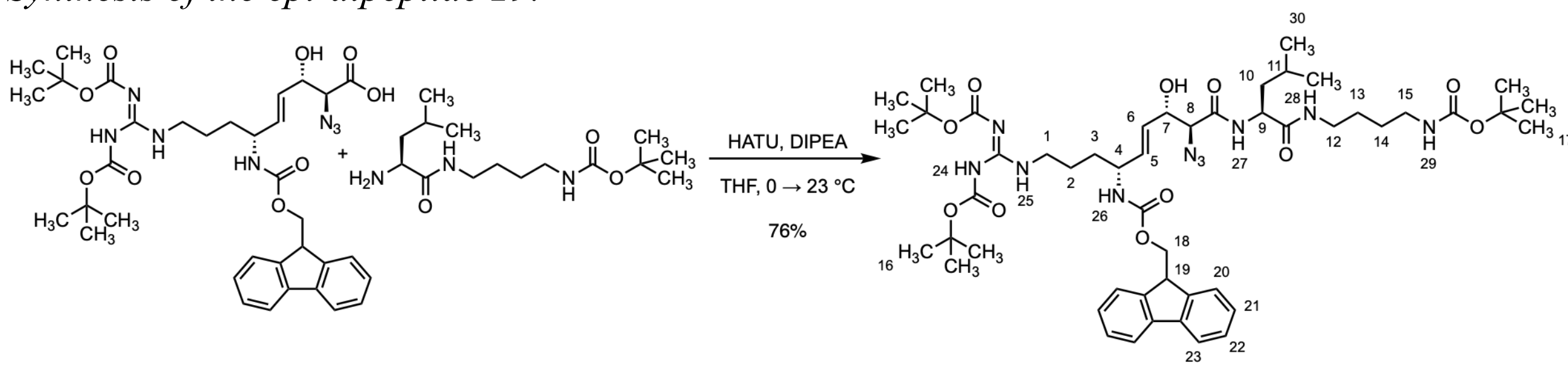

S18 5 19

To a cooled (0 °C) solution of *epi*-guanylated acid **S18** (4.50 g, 6.36 mmol, 1 equiv) and NH_2-Leu-*N*-Boc-Put **5** (2.49 g, 8.27 mmol, 1.30 equiv) in tetrahydrofuran (37 mL, C = 0.17 M) were added hexafluorophosphate azabenzotriazole tetramethyluronium (HATU, 3.34 g, 8.77 mmol, 1.38 equiv) and *N*,*N*-diisopropylethylamine (3.80 mL, 22.0 mmol, 3.46 equiv). The cooling bath was removed, and the reaction mixture was stirred for 18 h at 23 °C. The product mixture was concentrated then diluted with ethyl acetate (100 mL). The diluted product mixture was transferred to a separatory funnel and washed with 1% aqueous hydrogen chloride solution (50 mL), saturated aqueous sodium bicarbonate solution (50 mL), water (50 mL), and saturated aqueous sodium chloride solution (50 mL). The washed organic layer was dried over sodium sulfate. The dried solution was filtered, and the filtrate was concentrated. The residue was purified by flash column chromatography (eluting with 50 % ethyl acetate–hexane initially, grading to 80 % ethyl acetate, linear gradient) to afford the *epi-dipeptide* **19** as an off-white solid (4.76 g, 76%).

R_f = 0.40 (5% methanol–dichloromethane; UV)

$[\alpha]_D^{20}$: –4.5 (*c* 0.89, $CHCl_3$)

^{1}H NMR (500 MHz, DMSO-*d₆*) δ 11.51 (s, 1H, H_{24}), 8.29 (t, *J* = 5.8 Hz, 1H, H_{25}), 8.24 (d, *J* = 8.3 Hz, 1H, H_{27}), 7.89 (d, *J* = 7.6 Hz, 2H), 7.86 – 7.83 (m, 1H, H_{28}), 7.70 (d, *J* = 7.7 Hz, 2H), 7.47 (d, *J* = 8.7 Hz, 1H, H_{26}), 7.40 (td, *J* = 6.8, 3.2 Hz, 2H), 7.32 (ddd, *J* = 10.9, 5.5, 2.7 Hz, 2H), 6.78 (t, *J* = 5.8 Hz, 1H, H_{29}), 5.81 (d, *J* = 4.5 Hz, 1H, –O*H*), 5.68 (dd, *J* = 15.8, 6.3 Hz, 1H, H_5), 5.57 (dd, *J* = 15.5, 6.6 Hz, 1H, H_6), 4.43 – 4.09 (m, 5H, 1 × H_7, 1 × H_9, 2 × H_{18}, 1 × H_{19}), 3.98 (d, *J* = 8.1 Hz, 1H, H_4), 3.68 (d, *J* = 7.6 Hz, 1H, H_8), 3.26 (d, *J* = 6.3 Hz, 2H, H_1), 3.00 (q, *J* = 7.1 Hz, 2H, H_{12}), 2.89 (t, *J* = 6.2 Hz, 2H, H_{15}), 1.55 (app t, *J* = 6.9 Hz, 1H, H_{11}), 1.46 (s, 14H, 2 × H_2, 2 × H_3, 1 × H_{10}, 9 × H_{16}), 1.38 (s, 9H, H_{16}), 1.36 (s, 10H, 1 × H_{10}, 9 × H_{17}), 1.34 – 1.31 (m, 4H, 2 × H_{13}, 2 × H_{14}), 0.92 – 0.72 (m, 6H, H_{30}).

^{13}C NMR (126 MHz, DMSO-*d₆*) δ 171.2 (C), 167.9 (C), 163.2 (C), 155.6 (C), 155.5 (C), 155.3 (C), 152.1 (C), 143.9 (2 × C), 140.8 (2 × C), 133.6 (CH), 129.2 (CH), 127.6 (2 × CH), 127.1 (2 × CH), 125.2 (2 × CH), 120.2 (2 × CH), 82.9 (C), 78.1 (C), 77.4 (C), 71.4 (CH), 65.3 (CH_2), 64.9 (CH), 52.2 (CH), 51.2 (CH), 46.8 (CH), 40.8 (CH_2), 38.3 (CH_2), 32.1 (CH_2), 28.3 (3 × CH_3), 28.0 (3 × CH_3), 27.6 (3 × CH_3), 27.0 (CH_2), 26.4 (CH_2), 25.4 (CH_2), 24.3 (CH), 23.0 (CH_3), 21.4 (CH_3). Two carbons obscured by DMSO-*d₆* (2 × CH_2).

HRMS-CI (m/z): $[M + H]^+$ calcd for $C_{50}H_{75}N_{10}O_{11}$, 991.5651; found, 991.5664.

Synthesis of the epi-Myr-D-Asn azido alcohol ***S19***.

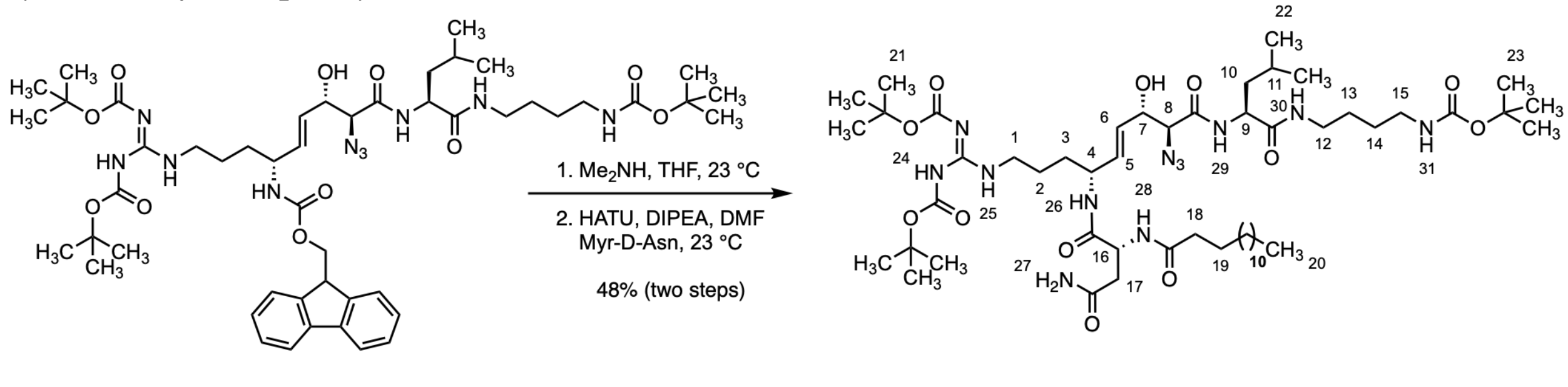

19 **S19**

To a solution of *epi*-dipeptide **19** (750 mg, 0.757 mmol, 1 equiv) in tetrahydrofuran (18 mL, C = 0.040 M) was added dimethylamine (2.0 M solution in tetrahydrofuran, 7.60 mL, 15.1 mmol, 20.0 equiv) and stirred for 5 h at 23 °C. The product mixture was concentrated and dried under high vacuum to afford a brown-yellow foam which was used in the next step without further purification. To a solution of the crude material obtained above (nominally 0.757 mmol, 1 equiv) in *N*,*N*-dimethylformamide (39 mL, C = 0.020 M) were sequentially added myristic acid-D-asparagine peptide (Myr-D-Asn, 798 mg, 2.33 mmol, 3.08 equiv), hexafluorophosphate azabenzotriazole tetramethyluronium (HATU, 738 mg, 1.94 mmol, 2.56 equiv) and *N*,*N*-diisopropylethylamine (DIPEA, 0.406 mL, 2.33 mmol, 3.08 equiv). The reaction mixture was stirred for 2 h at 23 °C. The product mixture was diluted with ethyl acetate (300 mL) and quenched with a 1.0 M aqueous hydrogen chloride solution (250 mL). The quenched product mixture was transferred to a separatory funnel and the layers that formed were separated. The organic layer was washed with water (5 × 100 mL) and a saturated aqueous sodium chloride solution (100 mL). The combined organic layers were dried over sodium sulfate. The dried solution was filtered, and the filtrate was concentrated. The residue was purified by flash column chromatography (washed with 100 % ethyl acetate initially, then eluting with 0 → 10 % methanol–dichloromethane, linear gradient) to afford *epi-Myr-D-Asn azido alcohol* **S19** as a pale orange film (400 mg, 48%).

Note: the residue was concentrated again from 10% ethyl acetate–hexane (10 mL) to induce precipitation.

R_f = 0.25 (5% methanol–dichloromethane; UV)

$[\alpha]_D^{20}$: +5.0 (*c* 0.89, CH_3OH)

^{1}H NMR (500 MHz, DMSO-*d*$_6$) δ 11.52 (s, 1H, H_{24}), 8.45 – 8.13 (m, 2H, H_{25}, H_{29}), 7.97 (d, *J* = 8.1 Hz, 1H, H_{28}), 7.86 (t, *J* = 5.8 Hz, 1H, H_{30}), 7.77 (d, *J* = 8.7 Hz, 1H, H_{26}), 7.27 (s, 1H, H_{27}), 6.85 (s, 1H, H_{27}), 6.78 (d, *J* = 6.0 Hz, 1H, H_{31}), 5.83 (d, *J* = 4.3 Hz, 1H, –O*H*), 5.69 (dd, *J* = 15.4, 6.3 Hz, 1H, H_5), 5.55 (dd, *J* = 15.5, 7.0 Hz, 1H, H_6), 4.52 (q, *J* = 7.4 Hz, 1H, H_{16}), 4.27 (app d, *J* = 7.6 Hz, 3H, H_4, H_7, H_9), 3.64 (d, *J* = 7.8 Hz, 1H, H_8), 3.30 – 3.19 (m, 2H, H_1), 3.00 (m, 2H, H_{12}), 2.94 – 2.81 (m, 2H, H_{15}), 2.45* (m, 1H, 1 × H_{17}), 2.33 (dd, *J* = 15.3, 7.9 Hz, 1H, 1 × H_{17}), 2.14 – 2.04 (m, 2H, H_{18}), 1.61 – 1.52 (m, 1H, H_{11}), 1.46 (s, 15H, 9 × H_{21}, 2 × H_{19}, 2 × H_2, 2 × H_3), 1.38 (s, 10H, 9 × H_{21}, 1 × H_{10}), 1.36 (s, 10H, 9 × H_{23}, 1 × H_{10}), 1.33 (s, 4H, 2 × H_{13}, 2 × H_{14}), 1.22 (d, *J*

= 6.3 Hz, 20H, 10 × CH_2), 0.91 – 0.68 (m, 9H, 6 × H_{22}, 3 × H_{20}). *Partially overlaps with residual H_2O peak.

^{13}C NMR (126 MHz, DMSO-*d*$_6$) δ 172.3 (C), 171.5 (C), 171.2 (C), 170.4 (C), 168.0 (C), 163.2 (C), 155.6 (C), 155.3 (C), 152.1 (C), 133.4 (CH), 129.3 (CH), 82.9 (C), 78.1 (C), 77.4 (C), 71.4 (CH), 64.8 (CH), 51.2 (CH), 49.8 (CH), 49.6 (CH), 40.8 (CH_2), 38.4 (CH_2), 37.2 (CH_2), 35.3 (CH_2), 32.0 (CH_2), 31.4 (CH_2), 29.1 (4 × CH_2), 29.0 (CH_2), 28.9 (CH_2), 28.8 (CH_2), 28.7 (CH_2), 28.3 (3 × CH_3), 28.0 (3 × CH_3), 27.6 (3 × CH_3), 27.0 (CH_2), 26.4 (CH_2), 25.3 (CH_2), 25.1 (CH_2), 24.3 (CH), 23.0 (CH_3), 22.2 (CH_3), 21.4 (CH_3), 14.0 (CH_3). Two carbons obscured by DMSO-*d*$_6$ (2 × CH_2).

HRMS-CI (m/z): $[M + H]^+$ calcd for $C_{53}H_{97}N_{12}O_{12}$, 1093.7343; found, 1093.7415.

*Synthesis of the epi-Myr-D-Asn amino alcohol **S20**.*

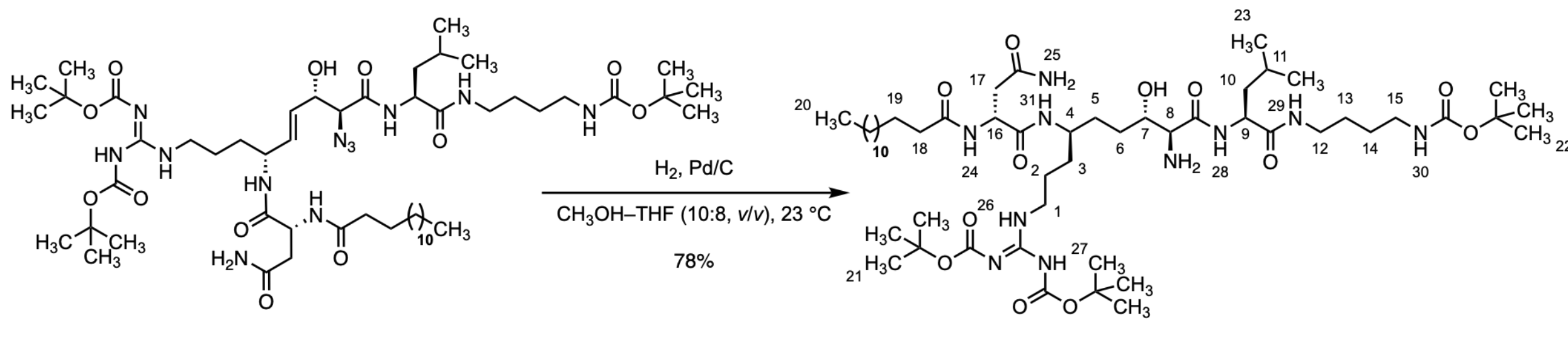

A round-bottom flask was charged with *epi*-Myr-D-Asn azido alcohol **S19** (300 mg, 0.274 mmol, 1 equiv) and palladium on carbon (Pd/C, 75 mg, 25 % *w*/*w* loading, dry matrix) then backfilled with argon gas. Methanol/tetrahydrofuran (7.0 mL, 10:8 *v*/*v*) was added and the reaction mixture was purged with dihydrogen gas for 5 min. The reaction mixture was stirred under an atmosphere of dihydrogen (1 atm; balloon) for 3 h at 23 °C. The product mixture was filtered through a pad of celite and was further washed with methanol (2 × 10 mL). The filtrate was concentrated, and the residue was redissolved in 10% ethyl acetate–hexane (10 mL). The product mixture was concentrated again to afford the *epi-Myr-D-Asn amino alcohol* **S20** as a white solid film (230 mg, 78%). The product obtained in this way was judged to be of >95% purity (^{1}H NMR analysis) and was advanced without purification.

R_f = 0.38 (5% methanol–dichloromethane + 0.1% Et_3N; UV, long streak)

$[\alpha]_D^{20}$: –14.6 (*c* 0.47, CH_3OH)

^{1}H NMR (500 MHz, DMSO-d_6) δ 11.51 (s, 1H, H_{27}), 8.22 (t, *J* = 5.6 Hz, 1H, H_{26}), 7.95 (app dq, *J* = 12.1, 7.1 Hz, 3H, H_{24}, H_{28}, H_{29}), 7.47 (d, *J* = 9.1 Hz, 1H, H_{31}), 7.28 (s, 1H, H_{25}), 6.86 (s, 1H, H_{25}), 6.78 (t, *J* = 5.8 Hz, 1H, H_{30}), 5.01 – 4.81 (br s, 1H, –O*H*), 4.57 – 4.38 (m, 1H, H_{16}), 4.21 (q, *J* = 7.9 Hz, 1H, H_9), 3.65 (m, 1H, H_4), 3.46 (app t, *J* = 8.0 Hz, 1H, H_7), 3.21 (q, *J* = 6.7 Hz, 2H, H_1), 3.11 (d, *J* = 6.6 Hz, 1H, H_8), 3.00 (d, *J* = 6.0 Hz, 1H, H_{12}), 2.87 (d, *J* = 5.9 Hz, 2H, H_{15}), 2.48 – 2.41* (m, 1H, 1 × H_{17}), 2.33 (dd, *J* = 15.1, 7.8 Hz, 1H, 1 × H_{17}), 2.06 (td, *J* = 7.3, 4.0 Hz, 2H, H_{18}), 1.56 (dq, *J* = 13.3, 6.5 Hz, 1H, H_{11}), 1.46 (s, 15H, 2 × H_2, 2 × H_3, 2 × H_{10}, 9 × H_{21}), 1.38 (s, 10H, 1 × H_{19}, 9 × H_{21}), 1.36 (s, 10H, 1 × H_{19}, 9 × H_{22}), 1.34 – 1.31 (m, 4H, 2 × H_{13}, 2 × H_{14}), 1.21 (s, 24H, 2 × H_5, 2 × H_6, 20 × CH_2), 0.84 (dt, *J* = 13.3, 6.4 Hz, 9H, 3 × H_{20}, 6 × H_{23}). *Partially overlaps with residual H_2O peak.

^{13}C NMR (126 MHz, DMSO-d_6) δ 173.6 (C), 172.2 (C), 171.8 (C), 171.6 (C), 170.7 (C), 163.2 (C), 155.6 (C), 155.2 (C), 152.2 (C), 82.9 (C), 78.1 (C), 77.4 (C), 72.5 (CH), 59.2 (CH), 50.9 (CH), 50.0 (CH), 48.0 (CH), 41.0 (CH_2), 40.4 (CH_2), 38.3 (CH_2), 37.4 (CH_2), 35.2 (CH_2), 32.0 (CH_2), 31.4 (CH_2), 30.7 (CH_2), 29.1 (4 × CH_2), 29.0 (CH_2), 28.9 (CH_2), 28.8 (CH_2), 28.7 (CH_2), 28.3 (CH_2), (3 × CH_3), 28.0 (3 × CH_3), 27.6 (3 × CH_3), 27.0 (CH_2), 26.5 (CH_2), 25.3 (CH_2), 25.2 (CH_2), 24.3 (CH), 23.1 (CH_3), 22.2 (CH_3), 21.5 (CH_2), 14.0 (CH_3). One carbon obscured by DMSO-d_6 (CH_2).
HRMS-CI (m/z): $[M + H]^+$ calcd for $C_{53}H_{101}N_{10}O_{12}$, 1069.7595; found, 1069.7649.

*Synthesis of the protected pre-rhabdobranin B **S21**.*

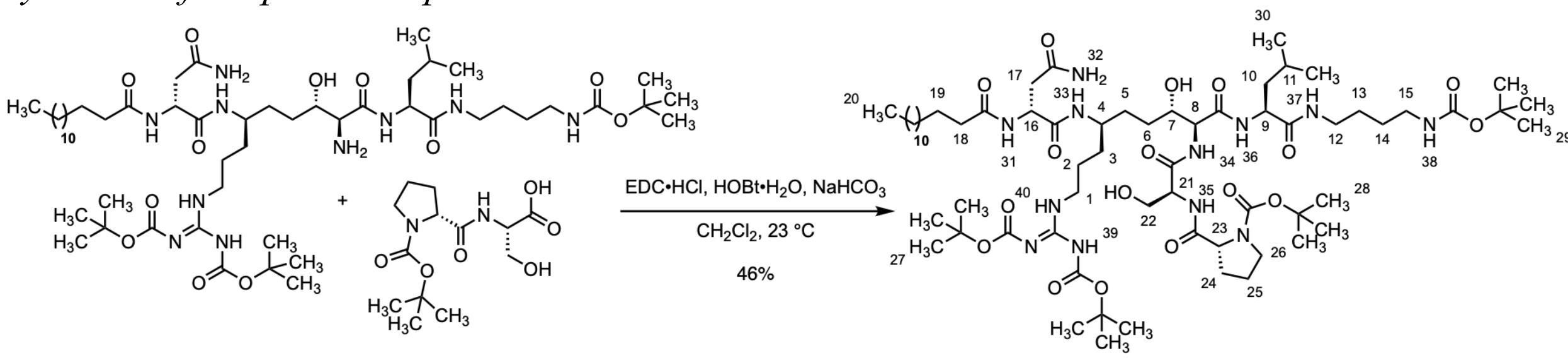

S20 **6** **S21**

The following procedure has been adapted from the literature.[12] To a solution of *epi*-Myr-D-Asn amino alcohol **S20** (190 mg, 0.178 mmol, 1 equiv) in dichloromethane (2.2 mL, C = 0.080 M) were added sodium hydrogen carbonate (18.2 mg, 0.217 mmol, 1.22 equiv), 1-hydroxybenzotriazole hydrate (HOBt•H_2O, 46.8 mg, 0.306 mmol, 1.72 equiv) and Boc-D-Pro-Ser **6** (69.8 mg, 0.231 mmol, 1.30 equiv) at 23 °C. The reaction mixture was cooled to 0 °C and 1-ethyl-3-(3-dimethylaminopropyl) carbodiimide hydrogen chloride (EDC•HCl, 41.6 mg, 0.217 mmol, 1.22 equiv) was added. The cooling bath was removed, and the reaction mixture was stirred for 16 h at 23 °C. The product mixture was diluted with brine (2.5 mL). The diluted product mixture was transferred to a separatory funnel and the layers that formed were separated. The aqueous phase was extracted with dichloromethane (3 × 5 mL). The combined organic extracts were dried over sodium sulfate. The dried solution was filtered, and the filtrate was concentrated. The residue was purified by flash column chromatography (eluting with 100% dichloromethane initially, grading to 10 % methanol–dichloromethane, linear gradient) to afford the *protected pre-rhabdobranin B* **S21** as a white solid (110 mg, 46%).

Note: For compounds containing the Boc-Pro-Ser fragment, several NMR resonances were broad or partially resolved. These features are consistent with slow conformational exchange of Boc amino acid rotamers on the NMR timescale; therefore, overlapping or broadened resonances are reported as observed without assigning individual rotameric species.[14]

R_f = 0.32 (5% methanol–dichloromethane; $KMnO_4$)

$[\alpha]_D^{20}$: +6.8 (*c* 0.22, $CHCl_3$)

^{1}H NMR (600 MHz, DMF-d_7) δ 11.66 (s, 1H, H_{39}), 8.35 (t, *J* = 5.6 Hz, 1H, H_{40}), 8.24 – 8.09 (m, 1H, H_{31}), 7.77 (s, 1H, H_{36}), 7.64 – 7.57 (m, 1H, 1 × H_{32}), 7.55 – 7.43 (m, 1H), 7.11 – 6.86 (m, 1H, 1 × H_{32}), 6.69 (s, 1H, H_{38}), 5.49 – 5.21 (m, 1H, –CH_2O*H* or –CHO*H*), 4.69 (d, *J* = 9.7 Hz, 1H, H_{16}), 4.60 – 4.45 (m, 1H, H_{21}), 4.33 (app s, 3H, H_8, H_9, H_{23}), 3.86 (app s, 4H, H_4, H_7, H_{22}), 3.48 – 3.42 (m, 1H, 1 × H_{26}), 3.36 (d, *J* = 8.2 Hz, 3H, 2 × H_1, 1 × H_{26}), 3.14 (d, *J* = 5.8 Hz, 2H, H_{12}), 3.05 (d, *J* = 9.2 Hz, 2H, H_{15}), 2.66 – 2.56 (m, 2H, H_{17}), 2.22 (t, *J* = 7.6 Hz, 2H, H_{18}), 2.03 – 1.94 (m, 2H), 1.81 (d, *J* = 5.0 Hz, 1H), 1.76 – 1.69 (m, 2H), 1.67 – 1.56 (m, 7H), 1.52 (s, 8H), 1.44 (s, 18H), 1.40 (s, 15H), 1.28 (s, 22H), 0.88 (ddd, *J* = 17.7, 10.5, 4.8 Hz, 9H, 3 × H_{20}, 6 × H_{29}).

Owing to substantial signal broadening, attributed primarily to conformational exchange associated with the four Boc-protecting groups, together with extensive signal overlap in this high-molecular-weight compound, unambiguous assignment of all resonances was not possible. Accordingly, only resonances that could be confidently assigned on the basis of COSY and HSQC correlations are reported; the remaining signals (H_2, H_3, H_5, H_6, H_{10}, H_{11}, H_{13}, H_{14}, H_{19}, H_{24}, H_{25}, H_{27}, H_{28}, H_{30}, H_{33}, H_{34}, H_{35}, and H_{37}) were left unassigned.

^{13}C NMR (151 MHz, DMF-*d*$_7$) δ 173.8 (C), 173.3 (C), 172.2 (C), 164.8 (C), 157.1 (C), 156.9 (C), 153.8 (C), 84.1 (C), 80.2 (C), 79.3 (C), 78.5 (C), 71.6* (CH), 63.1 (CH_2), 61.3 (CH), 59.5* (CH), 56.0 (CH), 53.0 (CH), 51.6 (CH), 49.2 (CH), 41.7 (CH_2), 41.0 (CH_2), 39.8 (CH_2), 38.5 (CH_2), 36.7 (CH_2), 33.6 (CH_2), 32.8 (CH_2), 32.3 (CH_2), 29.0 (3 × CH_3), 28.8 (5 × CH_3), 28.4 (4 × CH_3), 27.7 (CH_2), 26.7 (CH_2), 25.6 (CH), 24.5 (CH_2), 24.0 (CH_3), 23.5 (CH_2), 21.9 (CH_3), 14.7 (CH_3). Five quaternary carbons not observed. Five quaternary carbons not observed. 14 carbons obscured by DMF-*d*$_7$ or not clearly observed (CH_2). *Assigned using HSQC analysis.
HRMS-CI (m/z): [M + H]$^+$ calcd for $C_{66}H_{121}N_{12}O_{17}$, 1353.8967; found, 1353.9044.

Achiral SFC trace chromatogram of compound **S21**.

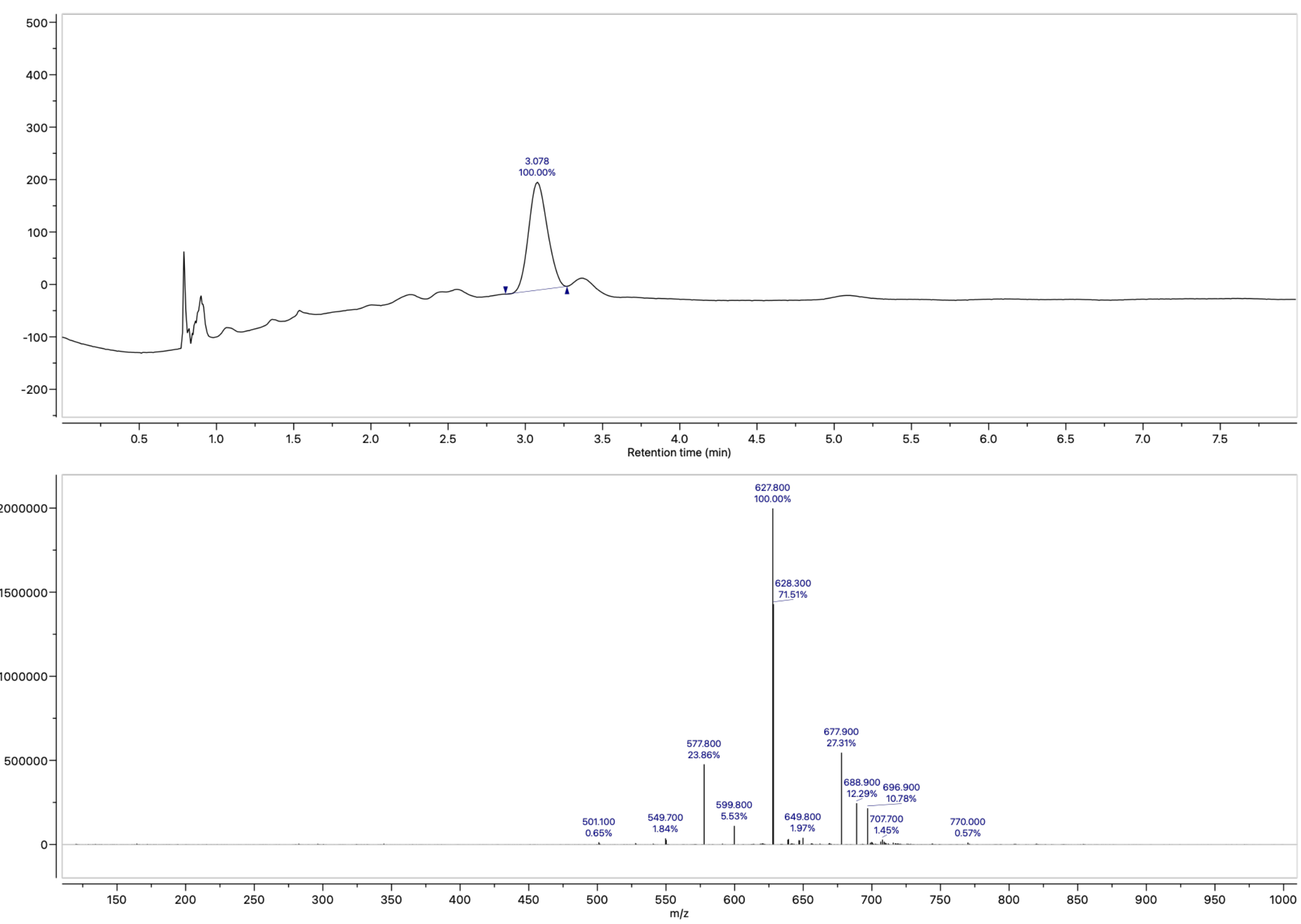

Column type: Agilent ZORBAX RX-SIL 4.6 × 150 mm, 5 μm.

Flow rate: 2 mL/min

Method: Isocratic

Time (min)	CO_2 (%)	Methanol (%)
0.00	80.0	20.0
8.00	80.0	20.0

Retention time: 3.08 min at λ = 210 nm.

Observed masses: 678 [M/2 + H^+] and 628 [(M–Boc)/2 + H^+].

*Synthesis of the revised pre-rhabdobranin B (5R)-**1**.*

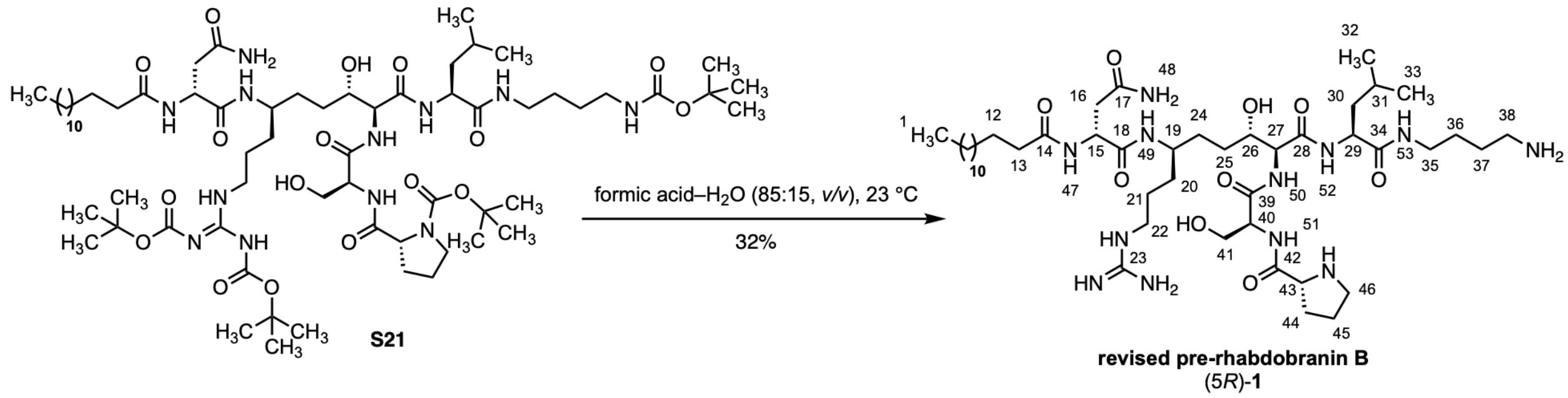

To protected pre-rhabdobranin B **S21** (85.0 mg, 62.8 μmol, 1 equiv), was added 85% (*v*/*v*) aqueous formic acid solution (1.0 mL, C = 0.060 M). The reaction mixture was stirred for 24 h at 23 °C. The product mixture was lyophilized to afford 52.0 mg of crude material as a white solid. 24.7 mg of the material was purified by reverse-phase HPLC purification with an XBridge BEH C18 column (130Å, 5 μm, 10 mm × 250 mm) using an acetonitrile–water gradient (+0.1% formic acid) for 0–30 min, 40–60%, 3 $mLmin^{-1}$ to afford *revised pre-rhabdobranin B* (5*R*)-**1** as a pale-yellow solid (9.0 mg, 32%).

Method:

0–20.0 min, 40%–60% acetonitrile;
20.0–20.5 min, 60%–100%;
20.5–25.0 min, 100%;
25.0–25.5 min, 100%–40%;
25.5–30.0, 40%.

Retention time: 11.0 min.

***Note*:** The deprotection step using formic acid[25] proceeds quantitatively to the desired product. The low yield likely reflects the challenges associated with purifying a polycationic species with low UV activity. Nonetheless, as this procedure furnished sufficient material for complete spectroscopic characterization, the deprotection and purification sequence was not further optimized.

^{1}H NMR (500 MHz, DMSO-d_6) δ 8.34 (s, 1H, H_{51}), 8.22 (d, J = 7.1 Hz, 1H, H_{50}), 8.17 (d, J = 7.8 Hz, 1H, H_{52}), 8.07 (app s, 1H, H_{47}), 7.70 (app s, 1H, H_{53}), 7.63 (d, J = 8.4 Hz, 1H, H_{49}), 7.42 (s, 1H, 1 × H_{48}), 6.92 (s, 1H, 1 × H_{48}), 4.49 (d, J = 8.7 Hz, 1H, H_{15}), 4.36 (app s, 1H, H_{40}), 4.14 (app s, 2H, H_{27}, H_{29}), 3.64 (d, J = 23.3 Hz, 3H, H_{19}, H_{26}, 1 × H_{41}), 3.50 (app s, 1H, 1 × H_{41}), 3.03 (app s, 4H, 2 × H_{22}, 2 × H_{35}), 2.89 (app s, 1H, 1 × H_{46}), 2.78 (s, 3H, 2 × H_{38}, 1 × H_{46}), 2.49 – 2.42* (m, 1H, 1 × H_{16}), 2.38 (d, J = 14.4 Hz, 1H, H_{16}, 1 × H_{16}), 2.09 (t, J = 7.4 Hz, 2H, H_{13}), 1.95 (app s, 1H, H_{44}), 1.72 (d, J = 23.0 Hz, 1H, H_{44}), 1.61 (app s, 3H, 1 × H_{31}, 1 × H_{36}, 1 × H_{45}), 1.44 (app s, 18H, 2 × H_2, 2 × H_{12}, 2 × H_{20}, 2 × H_{21}, 2 × H_{24}, 2 × H_{25}, 2 × H_{30}, 1 × H_{36}, 2 × H_{37}, 1 × H_{45}), 1.23 (app d, J = 4.6 Hz, 20H, 10 × CH_2, myristoyl), 0.85 (app t, J = 6.8 Hz, 6H, 3 × H_1, 3 × H_{32} or H_{33}), 0.79 (d, J = 6.3 Hz, 3H, 3 × H_{32} or H_{33}). *Partially overlaps with residual H_2O peak.

^{13}C NMR (126 MHz, DMSO-d_6) δ 174.8 (C), 172.8 (C), 172.4 (C), 172.2 (C), 171.5 (C), 171.0 (C), 170.8 (C), 157.8 (C), 70.7 (CH), 62.4 (CH_2), 60.4 (CH), 58.7 (CH), 55.0 (CH), 52.0 (CH), 50.5 (CH), 47.9 (CH), 47.0 (CH_2), 40.9 (CH_2), 40.6 (CH_2), 38.9 (CH_2), 38.3 (CH_2), 38.0 (CH_2), 35.7 (CH_2), 31.7 (2 × CH_2), 30.9 (CH_2), 30.6 (CH_2), 29.8 (2 × CH_2), 29.5 (3 × CH_2), 29.4 (CH_2), 29.3 (CH_2), 29.2 (2 × CH_2), 26.2 (2 × CH_2), 25.7 (CH_2), 25.4 (CH_2), 24.6 (CH), 23.6 (CH_3), 22.5 (2 × CH_2), 21.5 (CH_3), 14.4 (CH_3).

HRMS-CI (m/z): $[M + H]^+$ calcd for $C_{46}H_{89}N_{12}O_9$, 953.6870; found, 953.6895.

Synthetic Procedures of Revised Rhabdobranin

*Synthesis of the epi-amino alcohol **S22**.*

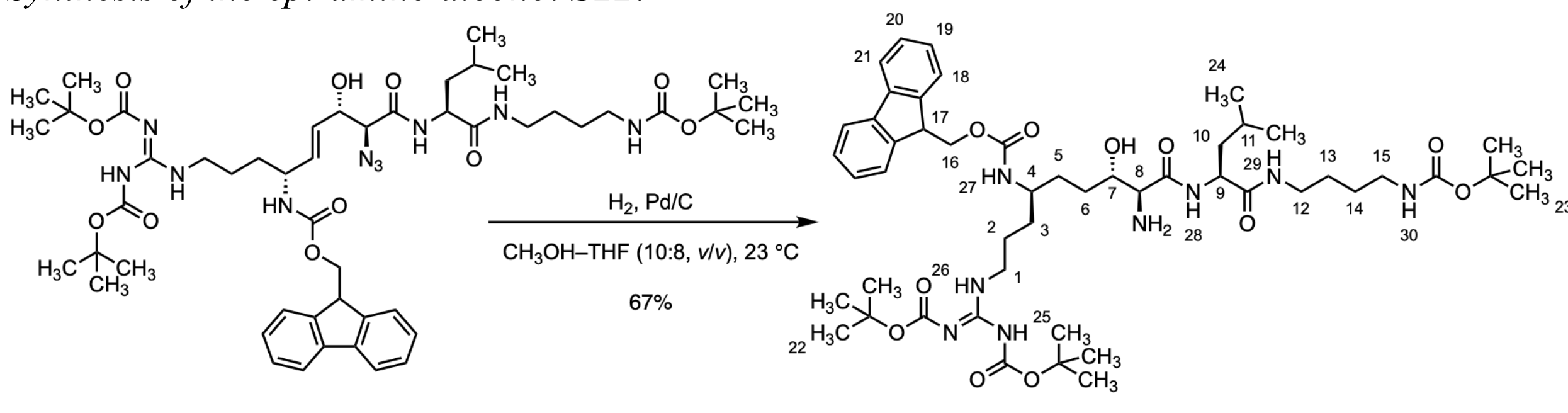

19 **S22**

The following procedure has been adapted from the literature.[24] A round-bottom flask was charged with *epi*-dipeptide **19** (2.00 g, 2.02 mmol, 1 equiv) and palladium on carbon (Pd/C, 500 mg, 25 % *w*/*w* loading, dry matrix) then backfilled with argon gas. Methanol/tetrahydrofuran (50 mL, 10:8 *v*/*v*) was added and the reaction mixture was purged with dihydrogen gas for 5 min. The reaction mixture was stirred under an atmosphere of dihydrogen (1 atm; balloon) for 3 h at 23 °C. The product mixture was filtered through a pad of celite and was further washed with methanol (2 × 20 mL). The filtrate was concentrated, and the residue was purified by flash column chromatography (eluting with 100% dichloromethane initially, grading to 10% methanol–dichloromethane + 0.1 % triethylamine, linear gradient) to afford *epi-amino alcohol* **S22** as a white solid (1.30 g, 67%).
R_f = 0.41 (10% methanol–dichloromethane + 0.1% Et_3N; UV)

$[\alpha]_D^{20}$: –10.3 (*c* 0.45, $CHCl_3$)

^{1}H NMR (500 MHz, DMSO-*d$_6$*) δ 11.50 (s, 1H, H_{25}), 8.26 (t, *J* = 5.7 Hz, 1H, H_{26}), 7.98 – 7.90 (m, 2H, H_{28}, H_{29}), 7.88 (d, *J* = 7.6 Hz, 2H, H_{21}), 7.69 (dd, *J* = 7.6, 4.4 Hz, 1H, H_{18}), 7.40 (tt, *J* = 7.6, 1.4 Hz, 2H, H_{20}), 7.32 (tdd, *J* = 7.4, 3.9, 1.3 Hz, 2H, H_{19}), 7.08 (d, *J* = 9.0 Hz, 1H, H_{27}), 6.75 (t, *J* = 5.7 Hz, 1H, H_{30}), 4.91 (br s, 1H, –O*H*), 4.32 (dd, *J* = 9.7, 6.6 Hz, 1H, 1 × H_{16}), 4.25 – 4.16 (m, 3H, 1 × H_9, 1 × H_{16}, 1 × H_{17}), 3.52 (app br s, 1H, H_7), 3.34* (br s, 1H, H_4), 3.25 (q, *J* = 6.8 Hz, 1H, H_1), 3.18 (d, *J* = 6.2 Hz, 1H, H_8), 3.00 (d, *J* = 6.6 Hz, 2H, H_{12}), 2.88 (d, *J* = 5.9 Hz, 2H, H_{15}), 1.56 (hept, *J* = 5.8 Hz, 2H, 1 × H_{10}, 1 × H_{11}), 1.46 (s, 14H, 2 × H_2, 2 × H_3, 1 × H_{10}, 9 × H_{22}), 1.39 (d, *J* = 5.5 Hz, 10H, 1 × H_2, 9 × H_{22}), 1.36 (s, 12H, 1 × H_2, 2 × H_3, 9 × H_{23}), 1.33 (s, 4H, 2 × H_{13}, 2 × H_{14}), 0.83 (dd, *J* = 14.6, 6.5 Hz, 6H, H_{24}). *Signal overlaps with residual H_2O peak and was assigned using HSQC analysis.

^{13}C NMR (126 MHz, DMSO-*d$_6$*) δ 171.7 (C), 163.2 (C), 155.9 (C), 155.6 (C), 155.2 (C), 152.1 (C), 144.0 (C), 143.9 (C), 140.7 (2 × C), 127.6 (2 × CH), 127.0 (2 ×CH), 125.2 (2 × CH), 120.1 (2 × CH), 82.9 (C), 78.1 (C), 77.3 (C), 72.3 (CH), 65.0 (CH_2), 59.2 (CH), 50.8 (CH), 50.7 (CH), 46.9 (CH), 41.1 (CH_2), 40.3 (CH_2), 38.3 (CH_2), 32.1, 30.9, 28.8, 28.3 (3 × CH_3), 28.0 (3 × CH_3), 27.6 (3 × CH_3), 26.9 (CH_2), 26.4 (CH_2), 25.5 (CH_2), 24.2 (CH), 23.0 (CH_3), 21.5 (CH_3). One carbon obscured by DMSO-*d$_6$* (CH_2). One quaternary carbon not observed.
HRMS-CI (m/z): $[M + H]^+$ calcd for $C_{50}H_{79}N_8O_{11}$, 967.5863; found, 967.5890.

*Synthesis of the protected rhabdobranin **S23**.*

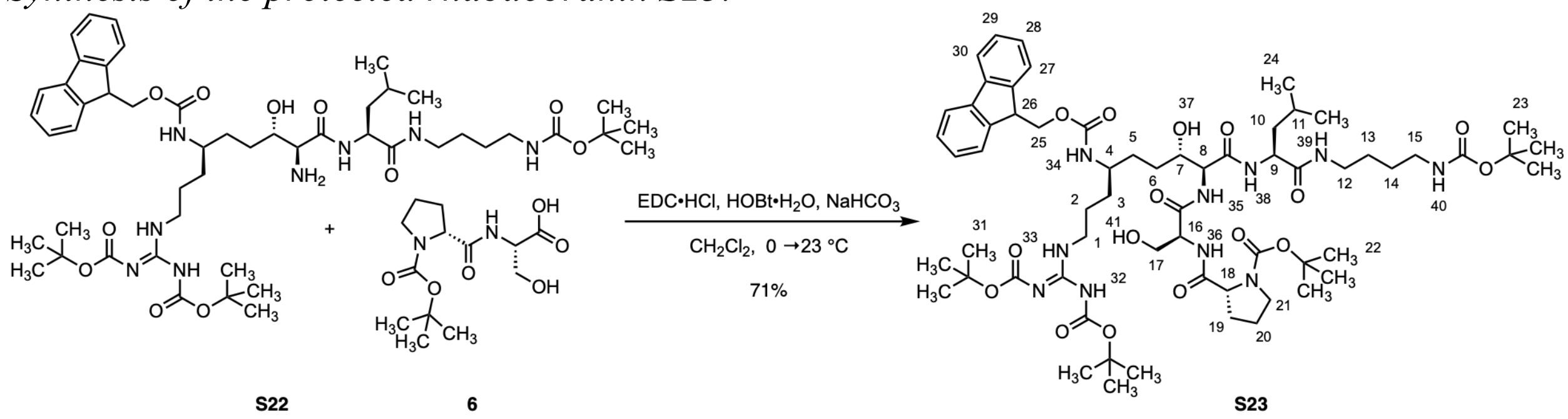

To a solution of *epi*-amino alcohol **S22** (750 mg, 0.775 mmol, 1 equiv) in dichloromethane (9.3 mL) were added sodium hydrogen carbonate (79.5 mg, 0.946 mmol, 1.22 equiv), 1-hydroxybenzotriazole hydrate (HOBt•H_2O, 204 mg, 1.33 mmol, 1.72 equiv) and Boc-D-Pro-Ser **6** (305 mg, 1.01 mmol, 1.30 equiv) at 23 °C. The reaction mixture was cooled to 0 °C and 1-ethyl-3-(3-dimethylaminopropyl) carbodiimide hydrogen chloride (EDC•HCl, 181 mg, 0.946 mmol, 1.22 equiv) was added. The cooling bath was removed, and the reaction mixture was stirred for 16 h at 23 °C. The product mixture was diluted with brine (9.3 mL). The diluted product mixture was transferred to a separatory funnel and the layers that formed were separated. The aqueous phase was extracted with dichloromethane (3 × 15 mL). The combined organic extracts were dried over sodium sulfate. The dried solution was filtered, and the filtrate was concentrated. The residue was purified by flash column chromatography (eluting with 100% dichloromethane initially, grading to 10 % methanol–dichloromethane, linear gradient) to afford the *protected rhabdobranin* **S23** as a white solid (710 mg, 71%).

Note: For compounds containing the Boc-Pro-Ser fragment, several NMR resonances were broad or partially resolved. These features are consistent with slow conformational exchange of Boc amino acid rotamers on the NMR timescale; therefore, overlapping or broadened resonances are reported as observed without assigning individual rotameric species.[14]

R_f = 0.36 (5% methanol–dichloromethane; UV)

$[\alpha]_D^{20}$: –51.0 (*c* 0.22, CH_3OH)

¹H NMR (500 MHz, DMF-d_7) δ 11.66 (app d, J = 2.6 Hz, 1H, H_{32}), 8.39 (d, J = 5.8 Hz, 1H, H_{33}), 8.15 (d, J = 29.0 Hz, 1H, H_{35}), 7.94 (d, J = 7.6 Hz, 4H, 2 × H_{30}, 1 × H_{36}, 1 × H_{38}), 7.76 (t, J = 7.8 Hz, 2H, H_{27}), 7.44 (tt, J = 6.2, 1.3 Hz, 2H, H_{29}), 7.36 (tdd, J = 7.3, 3.2, 1.1 Hz, 2H, H_{28}), 7.14 (dt, J = 8.6, 3.8 Hz, 1H, H_{34}), 6.67 (d, J = 6.5 Hz, 1H, H_{40}), 5.38 (app d, J = 18.0 Hz, 1H, H_{37} or H_{41}), 4.62 (d, J = 50.7 Hz, 1H, H_{37} or H_{41}), 4.34 (dd, J = 9.0, 6.7 Hz, 4H, 1 × H_9, 1 × H_{16}, 2 × H_{25}), 4.31 – 4.21 (m, 3H, 1 × H_8, 1 × H_{18}, 1 × H_{26}), 3.90 (dt, J = 11.1, 5.7 Hz, 2H, 2 × H_{17}), 3.76 (dt, J = 11.0, 5.7 Hz, 1H, 1 × H_7), 3.51† (s, 1H, H_4), 3.47 – 3.27 (m, 4H, 2 × H_1, 2 × H_{21}), 3.14 (d, J = 6.1 Hz, 2H, H_{12}), 3.04 (d, J = 6.2 Hz, 2H, H_{15}), 2.18 (m, 2H, 1 × H_{20}, 1 × H_{19}), 1.97 (m, 1H, H_{19}), 1.78 (d, J = 24.6 Hz, 2H, 1 × H_{19}, 1 × H_{11}), 1.73 – 1.57 (m, 6H, 2 × H_2, 2 × H_3, 2 × H_{10}), 1.52 – 1.50 (m, 11H, 2 × H_2, 9 × H_{31}), 1.44 (s, 16H, 2 × H_3, 2 × H_{13}, 3 × H_{22}, 9 × H_{31}), 1.40 (s, 17H, 2 × H_{14}, 6 ×

H_{22}, 9 × H_{23}), 0.99 – 0.79 (m, 6H, H_{24}). †Signal overlaps with residual H_2O peak and was assigned using HSQC analysis.

^{13}C NMR (126 MHz, DMF-*d*$_7$) δ 174.2 (C), 173.3 (C), 164.9 (C), 157.5 (C), 157.1 (C), 156.9 (C), 153.8 (C), 145.5 (C), 145.3 (C), 142.2 (2 × C), 128.7 (2 × CH), 128.2 (2 × CH), 126.5 (2 × CH), 121.1 (2 × CH), 84.1 (C), 80.1 (C), 79.3 (C), 78.5 (C), 72.6* (CH), 66.9 (CH_2), 63.3* (CH_2), 61.4* (CH), 59.2* (CH), 56.0* (CH), 53.0 (CH), 52.1 (CH), 48.3 (CH), 47.8* (CH_2), 41.7 (2 × CH_2), 41.0 (CH_2), 39.8 (CH_2), 33.5 (CH_2), 32.2 (CH_2), 29.0 (4 × CH_3), 28.8 (3 × CH_3), 28.4 (6 × CH_3), 27.7 (CH_2), 27.0 (CH_2), 25.6 (CH), 25.3 (CH_2), 24.4 (CH_2), 23.9 (CH_3), 21.9 (CH_3). Three quaternary carbons not observed. One carbon obscured by DMF- *d*$_7$ (CH_2). *Assigned using HSQC analysis.

HRMS-CI (m/z): $[M + H]^+$ calcd for $C_{63}H_{99}N_{10}O_{16}$, 1251.7235; found, 1251.7278.

Achiral SFC chromatogram trace of compound **S23**.

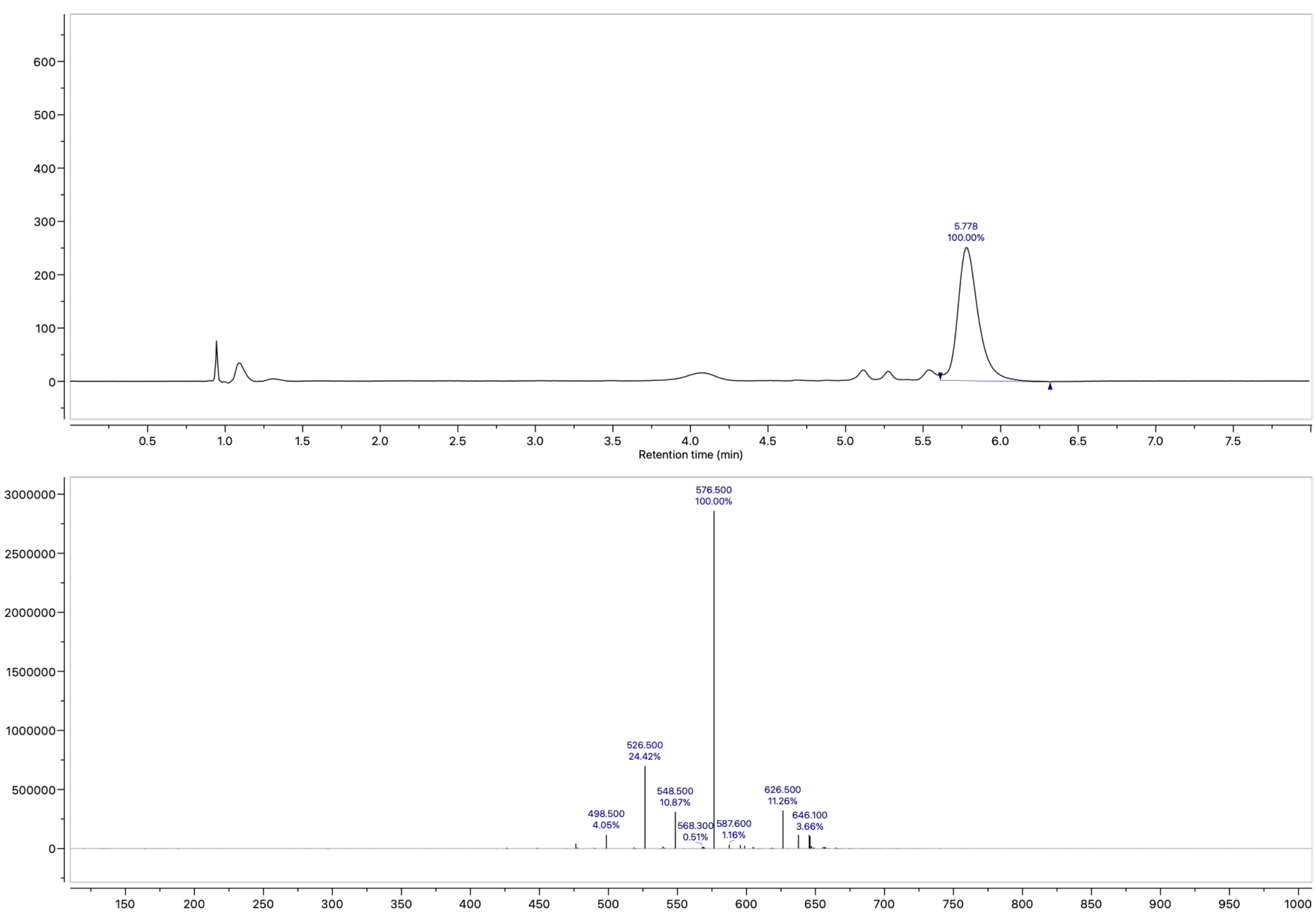

Column type: Agilent ZORBAX RX-SIL 4.6 × 150 mm, 5 μm.

Flow rate: 2 mL/min

Method: Gradient

Time (min)	CO_2 (%)	Methanol (%)
0.00	95.0	5.0
0.05	95.0	5.0
4.00	80.0	20.0
4.50	80.0	20.0
5.00	95.0	5.0
8.00	95.0	5.0

Retention time: 5.78 min at λ = 254 nm.

Observed masses: 626 [M/2 + H^+] and 576 [(M–Boc)/2 + H^+].

*Synthesis of the epi-free amine **S24**.*

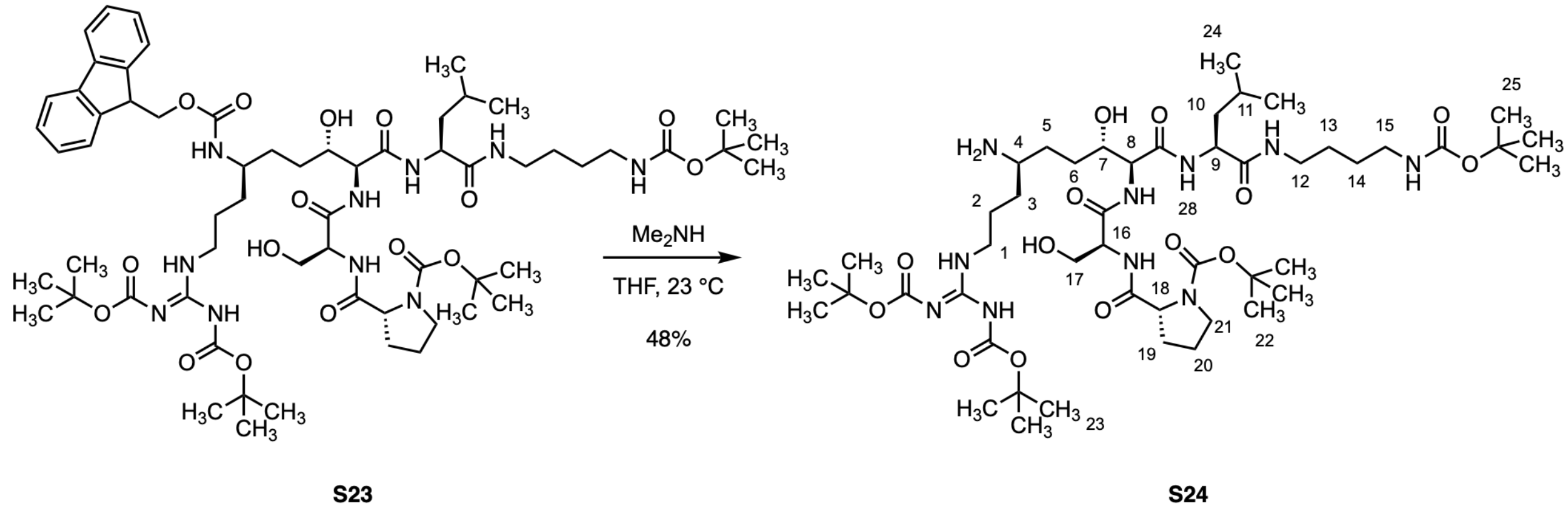

To a solution of protected rhabdobranin **S23** (640 mg, 0.511 mmol, 1 equiv) in tetrahydrofuran (12 mL, C = 0.040 M) was added dimethylamine (2.0 M solution in tetrahydrofuran, 5.10 mL, 10.2 mmol, 20.0 equiv). The reaction mixture was stirred for 5 h at 23 °C. The product mixture was concentrated, and the residue was purified by flash column chromatography (eluting with 100% dichloromethane initially, grading to 25 % methanol–dichloromethane, linear gradient) to afford *epi-free amine* **S24** as a white solid (251 mg, 48%).

Note: For compounds containing the Boc-Pro-Ser fragment, several NMR resonances were broad or partially resolved. These features are consistent with slow conformational exchange of Boc amino acid rotamers on the NMR timescale; therefore, overlapping or broadened resonances are reported as observed without assigning individual rotameric species.[14]

R_f = 0.33 (10% methanol–dichloromethane; $KMnO_4$)

$[\alpha]_D^{20}$: –43.3 (*c* 0.25, CH_3OH)

^{1}H NMR (500 MHz, CD_3OD) δ 4.35 (dd, *J* = 23.5, 17.3 Hz, 3H, 1 × H_8, 1 × H_9, 1 × H_{16}), 4.24 (dd, *J* = 8.4, 4.0 Hz, 1H, H_{18}), 3.95 – 3.70 (m, 3H, 1 ×H_7, 2 × H_{17}), 3.52 (q, *J* = 7.0 Hz, 1H, 1 × H_{21}), 3.48 – 3.32 (m, 3H, 2 × H_1, 1 × H_{21}), 3.17 (t, *J* = 6.3 Hz, 2H, H_{12}), 3.04 (td, *J* = 6.6, 2.8 Hz, 2H, H_{15}), 2.95 – 2.78 (m, 1H, H_4), 2.33 – 2.15 (m, 1H, 1 × H_{19}), 1.99 (d, *J* = 11.8 Hz, 2H, 1 × H_{19}, 1 × H_{20}), 1.88 (d, *J* = 8.3 Hz, 2H, 1 × H_{20}, 1 × H_2), 1.79 – 1.63 (m, 5H, 1 × H_2, 2 × H_3, 1 × H_5, 1 × H_{11}), 1.62 – 1.57 (m, 5H, 1 × H_5, 2 × H_6, 2 × H_{10}), 1.53 (s, 11H, 2 × H_{13}, 9 × H_{23}), 1.47 (d, *J* = 2.4 Hz, 17H, 2 × H_{14}, 6 × H_{22}, 9 × H_{23}), 1.43 (s, 12H, 3 × H_{22}, 9 × H_{25}), 1.08 – 0.67 (m, 6H, H_{24}).

^{13}C NMR (126 MHz, CD_3OD) δ 175.8 (C), 174.7 (C), 172.9 (C), 164.5 (C), 158.5 (C), 157.6 (C), 154.2 (C), 84.5 (C), 81.7 (C), 80.5 (C), 79.9 (C), 72.4* (CH), 63.0* (CH_2), 61.8* (CH), 59.4 (CH), 53.6 (CH), 52.2 (CH), 48.0 (CH_2), 41.5 (CH_2), 41.4 (CH_2), 41.0 (CH_2), 40.1 (CH_2), 32.4 (CH_2), 31.3 (CH_2), 28.8 (4 × CH_3), 28.7* (2 × CH_2), 28.6 (3 × CH_3), 28.2 (5 × CH_3), 27.5 (CH_2), 26.5 (CH_2), 25.9 (CH_2, CH), 25.5 (CH_2), 24.7 (CH_2), 23.6 (CH_3), 21.6 (CH_3). Two quaternary carbons not observed. *Assigned using HSQC analysis.

HRMS-CI (m/z): $[M + H]^+$ calcd for $C_{48}H_{89}N_{10}O_{14}$, 1029.6554; found, 1029.6572.

*Synthesis of the revised rhabdobranin (5R)-**2**.*

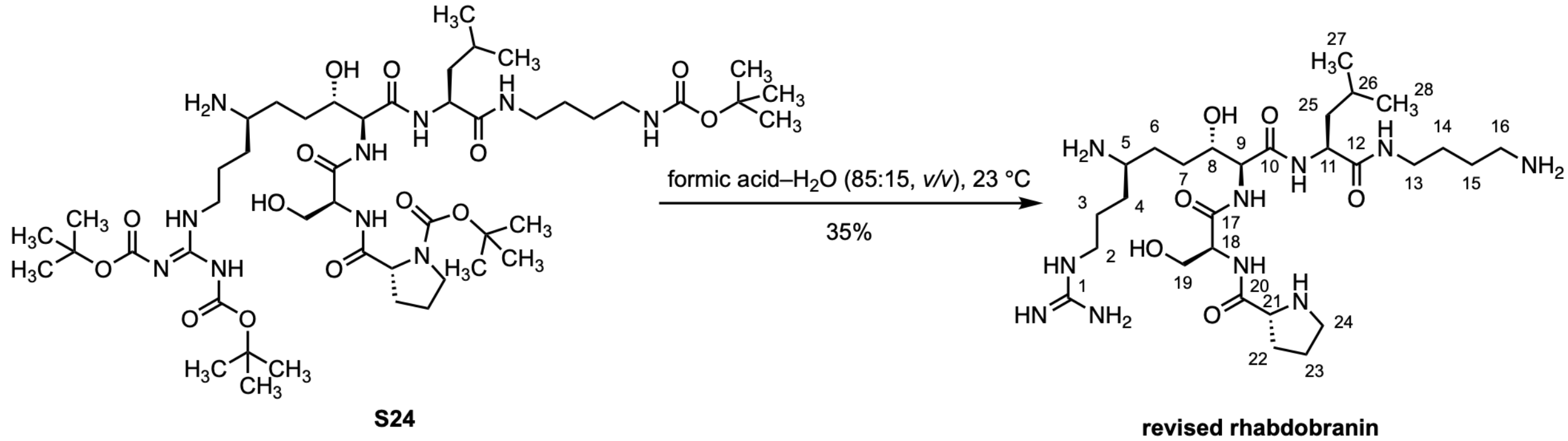

To *epi*-free amine **S24** (42.2 mg, 41.0 µmol, 1 equiv), was added 85% (*v*/*v*) aqueous formic acid solution (0.68 mL, C = 0.060 M). The reaction mixture was stirred for 24 h at 23 °C. The product mixture was lyophilized, diluted with methanol, and purified by reverse-phase HPLC with a HILIC column (XBridge BEH amide, 130Å, 5 µm, 10 mm × 250 mm) using an acetonitrile–water gradient (+0.1% formic acid) for 0–25 min, 94–60%, 3 $mLmin^{-1}$ to afford *revised rhabdobranin* (5*R*)-**2** as a deliquescent white solid (8.9 mg, 35%).
Method:

0–2.0 min, 94% acetonitrile;
2.0–19.0 min, 94%–60%;
19.0–19.5 min, 60%–5%;
19.5–20.0 min, 5%;
20.0-–20.5min, 5%–94%;
20.5–25.0, 94%.

Retention time: 13.5 min.

$[\alpha]_D^{20}$: –6.5 (*c* 0.60, CH_3OH)

^{1}H NMR (500 MHz, D_2O) δ 4.46 (t, *J* = 5.6 Hz, 1H, H_{18}), 4.40 (dd, *J* = 8.3, 5.9 Hz, 1H, H_{21}), 4.36 (d, *J* = 7.0 Hz, 1H, H_9), 4.25 (dp, *J* = 14.6, 5.2 Hz, 1H, H_{11}), 3.89 (dd, *J* = 14.0, 7.2 Hz, 1H, H_8), 3.84 – 3.74 (m, 2H, H_{19}), 3.37 (ddd, *J* = 16.8, 9.8, 3.5 Hz, 2H, H_{24}), 3.26 (d, *J* = 6.1 Hz, 1H, H_5), 3.15 (q, *J* = 8.9 Hz, 4H, 2 × H_2, 2 × H_{13}), 2.93 (t, *J* = 7.5 Hz, 2H, H_{16}), 2.41 (t, *J* = 7.9 Hz, 1H, 1 × H_{22}), 2.07 – 1.98 (m, 3H, 1 × H_{22}, 1 × H_{23}), 1.74 (q, *J* = 6.0 Hz, 1H, H_6), 1.68 – 1.47 (m, 14H, 2 × H_3, 2 × H_4, 1 × H_6, 2 × H_7, 2 × H_{14}, 2 × H_{15}, 2 × H_{25}, 1 × H_{26}), 0.86 (d, *J* = 5.6 Hz, 3H, H_{28}), 0.80 (d, *J* = 5.6 Hz, 3H, H_{29}).

^{13}C NMR (126 MHz, D_2O) δ 174.2 (C), 171.5 (C), 171.4 (C), 170.0 (C), 156.7 (C), 70.2 (CH), 61.1 (CH_2), 59.7 (CH), 58.0 (CH), 55.7 (CH), 52.7 (CH), 51.3 (CH), 46.5 (CH_2), 40.6 (CH_2), 39.7 (CH_2), 39.0 (CH_2), 38.6 (CH_2), 29.8 (CH_2), 29.0 (CH_2), 28.3 (CH_2), 28.0 (CH_2), 25.4 (CH_2), 24.3 (CH), 24.1 (CH_2), 23.8 (CH_2), 23.7 (CH_2), 22.1 (CH_3), 20.6 (CH_3).

HRMS-CI (m/z): $[M + H]^+$ calcd for $C_{28}H_{57}N_{10}O_6$, 629.4457; found, 629.4512.

Synthetic Procedures of Linear Rhabdobranin.

*Synthesis of the protected linear rhabdobranin **S25**.*

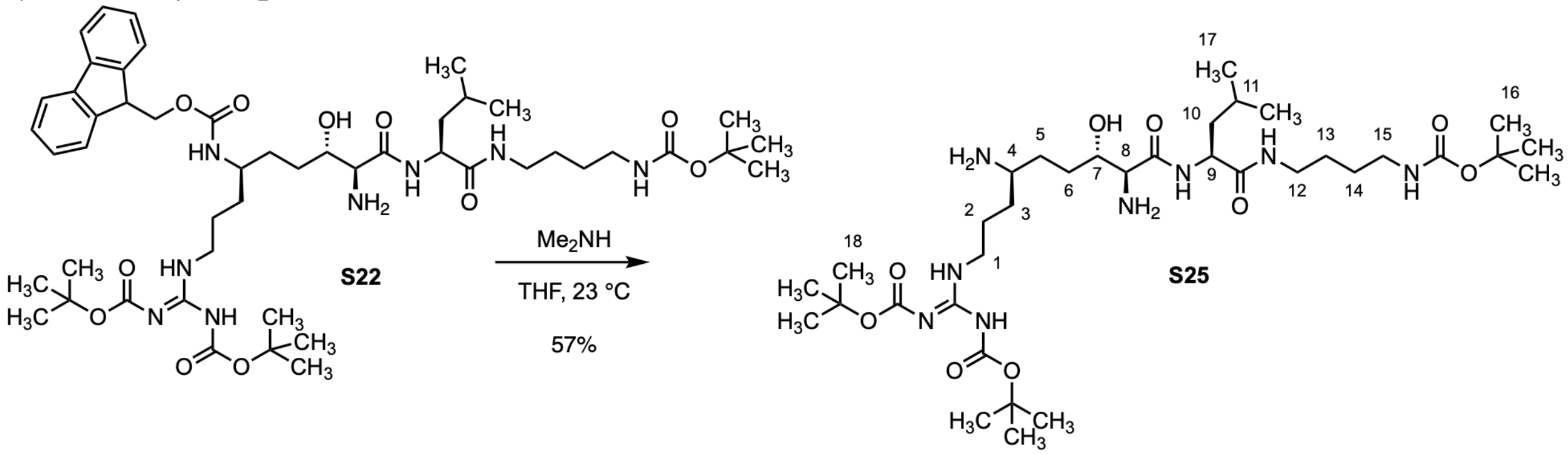

To a solution of *epi*-amino alcohol **S22** (120 mg, 124 μmol, 1 equiv) in tetrahydrofuran (3.0 mL, C = 0.040 M) was added dimethylamine (2.0 M solution in tetrahydrofuran, 1.20 mL, 2.48 mmol, 20.0 equiv). The reaction mixture was stirred for 5 h at 23 °C. The product mixture was concentrated, and the residue was purified by flash column chromatography (eluting with 100% dichloromethane initially, grading to 30 % methanol–dichloromethane, linear gradient) to afford *protected linear rhabdobranin* **S25** as a white solid (52.4 mg, 57%).

R_f = 0.39 (10% methanol–dichloromethane; $KMnO_4$)

$[\alpha]_D^{20}$: –7.1 (*c* 0.88, CH_3OH)

^{1}H NMR (500 MHz, CD_3OD) δ 4.37 (dd, *J* = 10.7, 4.3 Hz, 1H, H_9), 3.59 (ddd, *J* = 8.9, 7.2, 2.4 Hz, 1H, H_7), 3.43 – 3.34 (m, 2H, H_1), 3.28 (d, *J* = 7.2 Hz, 1H, H, H_8), 3.16 (t, *J* = 6.3 Hz, 2H, H_{12}), 3.03 (t, *J* = 6.5 Hz, 2H, H_{15}), 2.74 (tq, *J* = 12.2, 6.3 Hz, 1H, H_4), 1.81 – 1.57 (m, 8H, 2 × H_2, 2 × H_5, 1 × H_6, 2 × H_{10}, 1 × H_{11}), 1.53 (s, 10H, 1 × H_6, 9 × H_{18}), 1.47 (s, 14H, 1 × H_3, 2 × H_{13}, 2 × H_{14}, 9 × H_{18}), 1.43 (d, *J* = 3.5 Hz, 10H, 1 × H_3, 9 × H_{16}), 0.94 (dd, *J* = 17.6, 6.4 Hz, 6H, H_{17}).

^{13}C NMR (126 MHz, CD_3OD) δ 176.4 (C), 174.7 (C), 164.6 (C), 158.5 (C), 157.6 (C), 154.2 (C), 84.5 (C), 80.4 (C), 79.9 (C), 75.3 (CH), 60.7 (CH), 53.4 (CH), 52.2 (CH), 41.7 (2 × CH_2), 41.0 (CH_2), 40.1 (CH_2), 35.9 (CH_2), 34.3 (CH_2), 31.3 (CH_2), 28.8 (3 × CH_3), 28.6 (3 × CH_3), 28.2 (3 × CH_3), 27.5 (CH_2), 26.7 (CH_2), 25.9 (CH), 23.6 (CH_3), 21.7 (CH_3).

HRMS-CI (m/z): $[M + H]^+$ calcd for $C_{35}H_{69}N_8O_9$, 745.5182; found, 745.5238.

*Synthesis of the linear rhabdobranin **20**.*

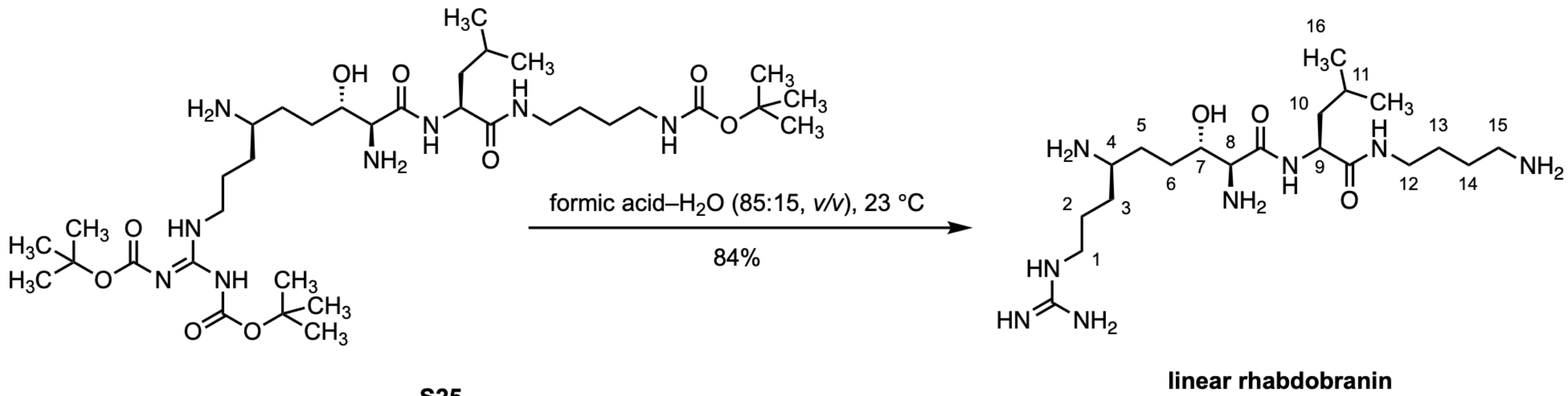

To protected linear rhabdobranin **S25** (40.0 mg, 53.7 μmol, 1 equiv), was added 85% (*v*/*v*) aqueous formic acid solution (0.900 mL, C = 0.060 M). The reaction mixture was stirred for 24 h at 23 °C. The product mixture was lyophilized to afford *linear rhabdobranin* **20** as a deliquescent pale brown solid (20 mg, 84%). The residue was judged to be >95% purity by ^{1}H NMR analysis.

$[\alpha]_D^{20}$: –30.4 (*c* 0.21, CH_3OH)

^{1}H NMR (500 MHz, D_2O) δ 4.34 (dd, *J* = 9.0, 5.7 Hz, 1H, H_9), 4.10 (app s, 1H, H_8), 4.07 (d, *J* = 3.8 Hz, 1H, H_7), 3.37 – 3.27 (m, 2H, H_4, 1 × H_{12}), 3.24 (q, *J* = 4.5 Hz, 2H, H_1), 3.21 – 3.10 (m, 1H, 1 × H_{12}), 3.01 (t, *J* = 7.5 Hz, 2H, H_{15}), 1.93 – 1.84 (m, 1H, 1 × H_5), 1.83 – 1.53 (m, 14H, 2 × H_2, 2 × H_3, 1 × H_5, 2 × H_6, 2 × H_{10}, 1 × H_{11}, 2 × H_{13}, 2 × H_{14}), 0.93 (dd, *J* = 18.0, 6.1 Hz, 6H)

^{13}C NMR (126 MHz, D_2O) δ 173.9 (C), 166.7 (C), 156.7 (C), 69.1 (CH), 56.8 (CH), 52.9 (CH), 51.2 (CH), 40.7 (CH_2), 39.9 (CH_2), 39.1 (CH_2), 38.7 (CH_2), 29.0 (CH_2), 28.5 (CH_2), 27.1 (CH_2), 25.4 (CH_2), 24.3 (CH), 24.2 (CH_2), 23.8 (CH_2), 21.9 (CH_3), 20.9 (CH_3).

HRMS-CI (m/z): $[M + H]^+$ calcd for $C_{20}H_{45}N_8O_3$, 445.3609; found, 445.3632.

Synthetic Procedures of *N*-acetylated Rhabdobranin.
Synthesis of the protected N-acetyl rhabdobranin ***S26***.

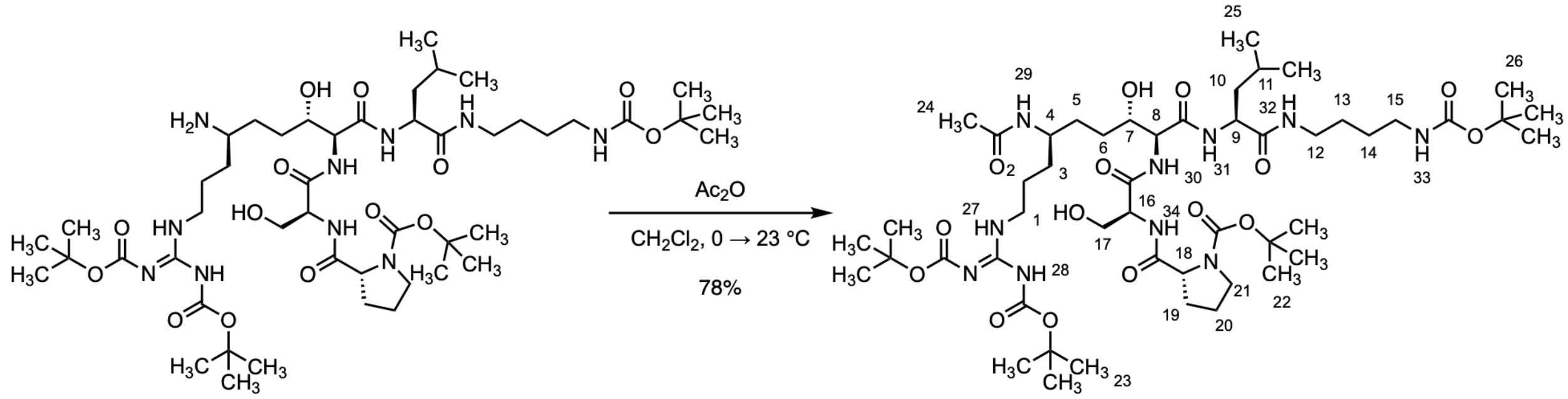

S24 S26

To a cooled (0 °C) solution of *epi*-free amine **S24** (10.0 mg, 9.70 μmol, 1 equiv) in dichloromethane (50.0 μL, C = 0.20 M) was added acetic anhydride (2.80 μL, 29.1 μmol, 3.00 equiv) and the reaction mixture was stirred for 1 h at 0 °C. The cooling bath was removed, and the reaction mixture was stirred for 3 h at 23 °C. The product mixture was quenched with a saturated aqueous sodium carbonate solution (0.2 mL). The aqueous layer was extracted with dichloromethane (3 × 0.5 mL). The combined organic extracts were dried over sodium sulfate and filtered. The filtrate was concentrated to afford *protected N-acetyl rhabdobranin* **S26** as a white solid (8.1 mg, 78%). The residue was judged to be of >95 % purity (^{1}H NMR analysis) and was advanced without purification.

Note: For compounds containing the Boc-Pro-Ser fragment, several NMR resonances were broad or partially resolved. These features are consistent with slow conformational exchange of Boc amino acid rotamers on the NMR timescale; therefore, overlapping or broadened resonances are reported as observed without assigning individual rotameric species.[14]

R_f = 0.50 (10% methanol–dichloromethane; $KMnO_4$)

$[\alpha]_D^{20}$: –24.9 (*c* 0.21, $CHCl_3$)

^{1}H NMR (500 MHz, DMSO-*d₆*) δ 11.49 (s, 1H, H_{28}), 8.25 (d, *J* = 6.1 Hz, 1H, H_{27}), 7.95 (d, *J* = 7.9 Hz, 1H, H_{32}), 7.88 (d, *J* = 16.5 Hz, 1H, H_{29}), 7.82 (s, 1H, H_{34}), 7.63 (s, 1H, H_{30}), 7.56 (d, *J* = 10.5 Hz, 1H, H_{32}), 6.75 (t, *J* = 6.0 Hz, 1H, H_{33}), 5.11 (d, *J* = 44.8 Hz, 1H, –CHO*H*), 4.39 (s, 1H, H_{16}), 4.27 – 4.07 (m, 3H, H_8, H_9, H_{18}), 3.75 – 3.48 (m, 4H, H_4, H_7, H_{17}), 3.24 (q, *J* = 7.0 Hz, 4H, H_1, H_{21}), 3.06 – 2.96 (m, 2H, H_{12}), 2.88 (d, *J* = 6.1 Hz, 2H, H_{15}), 2.17 (d, *J* = 5.6 Hz, 1H, 1 × H_{19}), 1.80 (d, *J* = 10.3 Hz, 6H, 1 × H_{19}, 2 × H_{20}, 3 × H_{24}), 1.63 – 1.56 (m, 1H, H_{11}), 1.47 (s, 13H, 1 × H_2, 2 × H_3, 1 × H_{10}, 9 × H_{23}), 1.39 (s, 15H, 1 × H_2, 1 × H_5, 1 × H_6, 1 × H_{10}, 1 × H_{13}, 1 × H_{14}, 9 × H_{23}), 1.36 (s, 9H, H_{26}), 1.31 (s, 13H, 1 × H_5, 1 × H_6, 1 × H_{13}, 1 × H_{14}, 9 × H_{22}), 0.83 (dd, *J* = 31.1, 6.5 Hz, 6H, H_{25}).

^{13}C NMR (126 MHz, DMSO-*d₆*) δ 172.0 (C), 171.7 (C), 170.3 (C), 163.1 (C), 155.6 (C), 155.2 (C), 152.1 (C), 82.9 (C), 78.5* (C), 78.1 (C), 77.3 (C), 71.0* (CH), 62.0* (CH_2), 59.6* (CH), 57.8* (CH), 51.3 (CH), 48.3* (CH), 46.5* (CH_2), 40.4 (CH_2), 40.2 (CH_2), 38.3 (CH_2), 31.9 (CH_2), 31.3* (CH_2), 31.0* (CH_2), 30.7* (CH_2), 29.9* (CH_2), 29.0* (CH_2), 28.3 (3 × CH_3), 28.1 (3 × CH_3), 28.0

(3 × CH_3), 27.6 (3 × CH_3), 26.8 (CH_2), 26.4 (CH_2), 25.5 (CH_2), 24.1 (CH), 23.1 (CH_3), 22.7 (CH_3), 21.1 (CH_3). Three quaternary carbons not observed. One carbon obscured by DMSO-d_6 (CH_2). *Assigned using HSQC analysis.

HRMS-CI (m/z): $[M + H]^+$ calcd for $C_{50}H_{91}N_{10}O_{15}$, 1071.6660; found, 1071.6752.

Achiral SFC trace chromatogram of compound **S26**.

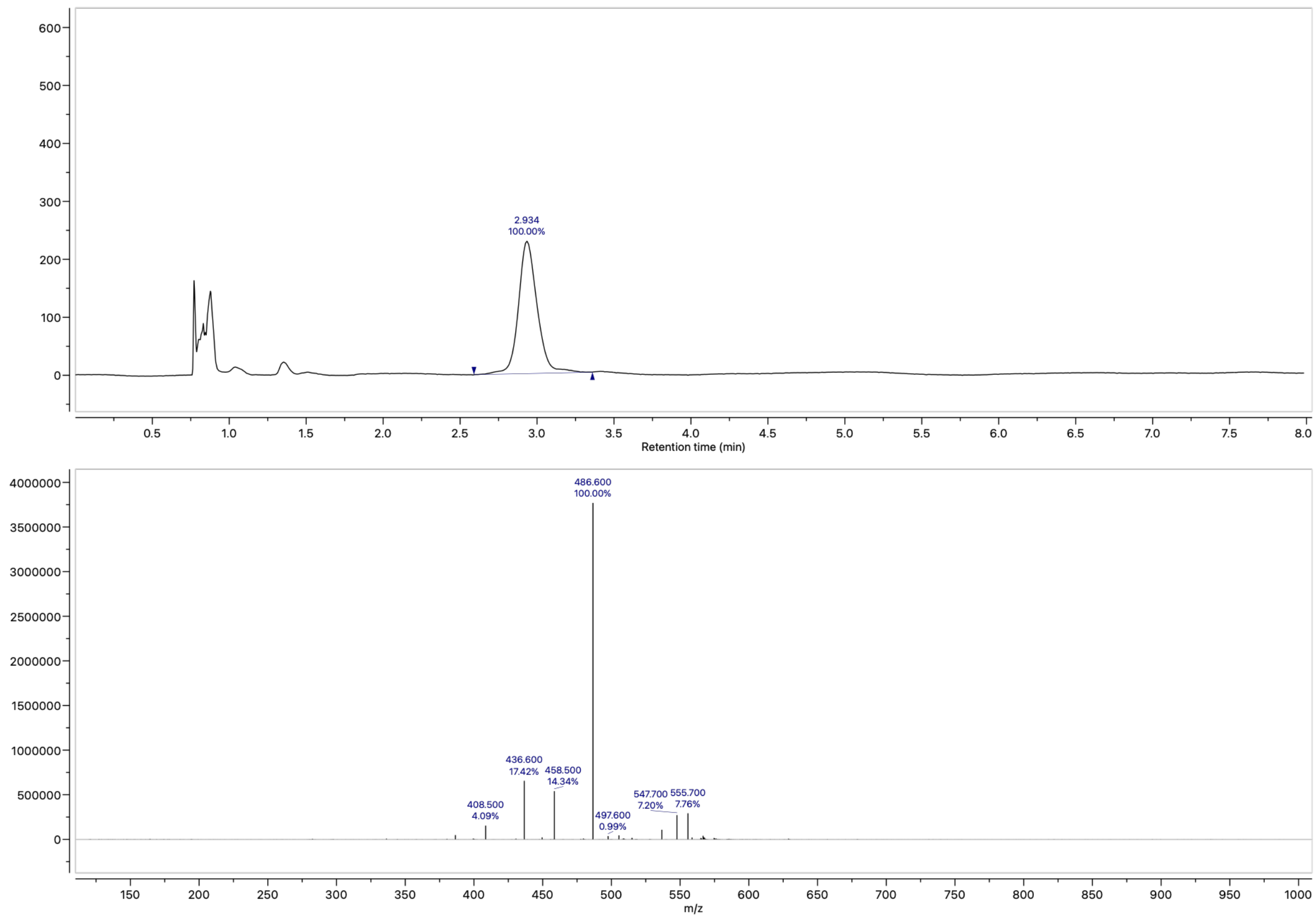

Column type: Agilent ZORBAX RX-SIL 4.6 × 150 mm, 5 µm.

Flow rate: 2 mL/min
Method: Isocratic

Time (min)	CO_2 (%)	Methanol (%)
0.00	80.0	20.0
8.00	80.0	20.0

Retention time: 2.93 min at λ = 210 nm.

Observed masses: 486 [(M–Boc)/2 + H^+].

*Synthesis of the N-acetyl rhabdobranin (5R)-**3**.*

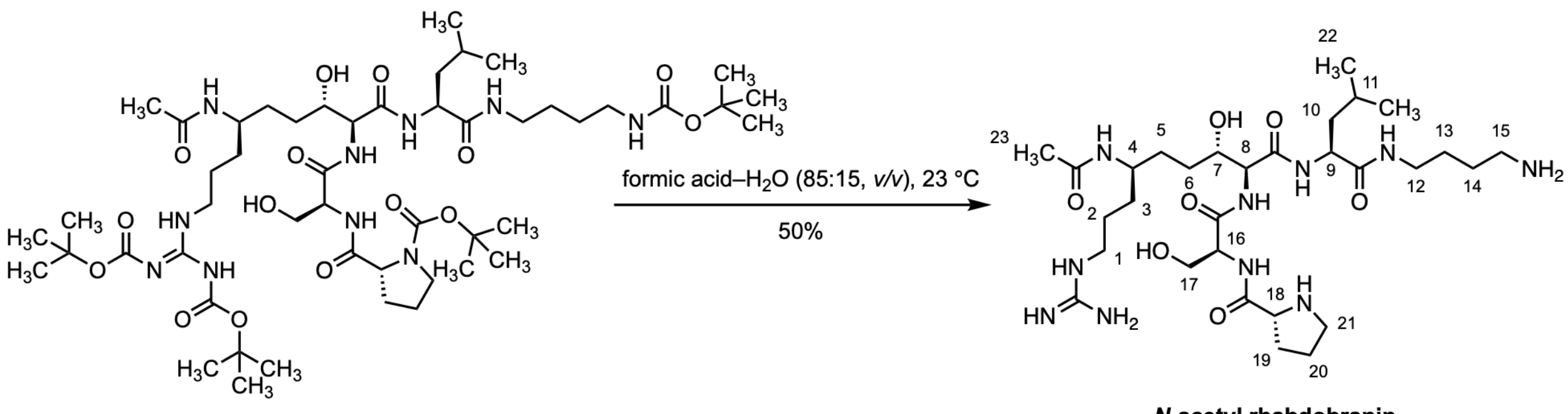

To protected *N*-acetyl rhabdobranin **S26** (61.7 mg, 57.6 μmol, 1 equiv), was added 85% (*v*/*v*) aqueous formic acid solution (0.960 mL, C = 0.060 M). The reaction mixture was stirred for 24 h at 23 °C. The product mixture was lyophilized, diluted with methanol, and purified by reverse-phase HPLC with a HILIC column (XBridge BEH amide, 130Å, 5 μm, 10 mm × 250 mm) using an acetonitrile–water gradient (+0.1% formic acid) for 0–15 min, 95–85%, 3 $mLmin^{-1}$ to afford the *N-acetyl rhabdobranin* (5*R*)-**3** as a yellowish foam (19.2 mg, 50%).

Method:

0-5.0 min, 95% acetonitrile;
5.0-10.0 min, 95%-85%;
10.0-10.5 min, 85%-5%;
10.5-11.5 min, 5%;
11.5-12.0 min, 5%-98%;
12.0-15.0, 98%.

Retention time: 6.93 min.

$[\alpha]_D^{20}$: –54.8 (*c* 0.10, CH_3OH)

^{1}H NMR (500 MHz, D_2O) δ 4.53 (t, *J* = 5.5 Hz, 1H, H_{16}), 4.47 (dd, *J* = 8.5, 6.1 Hz, 1H, H_{18}), 4.38 (d, *J* = 7.0 Hz, 1H, H_8), 4.31 (p, *J* = 4.9 Hz, 1H, H_9), 3.98 – 3.83 (m, 3H, H_7, H_{17}), 3.79 (app br s, 1H, H_4), 3.56 – 3.31 (m, 2H, H_{21}), 3.31 – 3.08 (m, 4H, H_1, H_{12}), 3.01 (t, *J* = 7.5 Hz, 2H, H_{15}), 2.49 (d, *J* = 9.4 Hz, 1H, 1 × H_{19}), 2.16 – 2.03 (m, 3H, 1 × H_{19}, 2 × H_{20}), 2.01 (s, 3H, H_{23}), 1.69 – 1.52 (m, 13H, 2 × H_2, 2 × H_3, 1 × H_5, 1 × H_6, 2 × H_{10}, 1 × H_{11}, 2 × H_{13}, 2 × H_{14}), 1.45 (d, *J* = 9.7 Hz, 2H, 1 × H_5, 1 × H_6), 1.02 – 0.74 (m, 6H, H_{22}).

^{13}C NMR (126 MHz, D_2O) δ 174.2 (C), 173.8 (C), 171.6 (C), 169.9 (C), 162.6 (C), 156.7 (C), 70.2 (CH), 61.1 (CH_2), 59.8 (CH), 58.3 (CH), 55.6 (CH), 52.6 (CH), 48.8 (CH), 46.5 (CH_2), 40.8 (CH_2), 39.7 (CH_2), 39.0 (CH_2), 38.5 (CH_2), 31.4 (CH_2), 30.2 (CH_2), 29.8 (CH_2), 29.1 (CH_2), 25.4 (CH_2), 24.5 (CH), 24.3 (CH_2), 24.1 (CH_2), 23.7 (CH_2), 22.1 (CH_3), 22.0 (CH_3), 20.6 (CH_3).
HRMS-CI (m/z): $[M + Hs]^+$ calcd for $C_{30}H_{59}N_{10}O_7$, 671.4563; found, 671.4589.

Synthetic Procedures of L-Asn-L-Arg Pre-rhabdobranin B.

*Synthesis of the L-Asn-L-Arg-azido alcohol **S27**.*

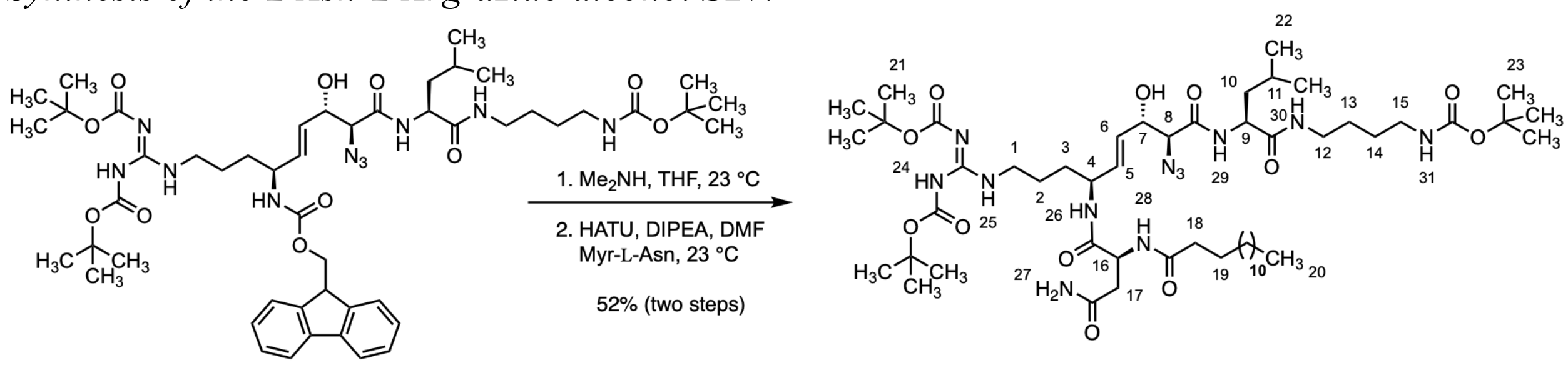

To a solution of dipeptide **16** (180 mg, 0.182 mmol, 1 equiv) in tetrahydrofuran (4.4 mL, C = 0.040 M) was added dimethylamine (2.0 M solution in tetrahydrofuran, 1.82 mL, 3.63 mmol, 20.0 equiv) and stirred for 5 h at 23 °C. The product mixture was concentrated and dried under high vacuum to afford a brown-yellow foam which was used in the next step without further purification. To a solution of the crude material obtained above (nominally 0.182 mmol, 1 equiv) in *N*,*N*-dimethylformamide (9.4 mL, C = 0.020 M) were sequentially added myristic acid-L-asparagine peptide (Myr-L-Asn, 192 mg, 0.560 mmol, 3.08 equiv), hexafluorophosphate azabenzotriazole tetramethyluronium (HATU, 177 mg, 0.467 mmol, 2.56 equiv) and *N*,*N*-diisopropylethylamine (DIPEA, 97.5 µL, 0.560 mmol, 3.08 equiv). The reaction mixture was stirred for 2 h at 23 °C. The product mixture was diluted with ethyl acetate (50 mL) and quenched with a 1.0 M aqueous hydrogen chloride solution (40 mL). The quenched product mixture was transferred to a separatory funnel and the layers that formed were separated. The organic layer was washed with water (5 × 20 mL) and a saturated aqueous sodium chloride solution (20 mL). The combined organic layers were dried over sodium sulfate. The dried solution was filtered, and the filtrate was concentrated. The residue was purified by flash column chromatography (washed with 100 % ethyl acetate initially, then eluting with 0 → 10 % methanol–dichloromethane, linear gradient) to afford *L-Asn-L-Arg-azido alcohol* **S27** as an off-white film (104 mg, 52%).

Note: the residue was concentrated again from 10% ethyl acetate–hexane (2 mL) to induce precipitation.

R_f = 0.34 (5% methanol–dichloromethane; $KMnO_4$)

$[\alpha]_D^{20}$: –7.61 (*c* 0.30, CH_3OH)

^{1}H NMR (500 MHz, DMSO-d_6) δ 11.51 (s, 1H, H_{24}), 8.25 (t, J = 5.6 Hz, 1H, H_{25}), 8.18 (d, J = 8.4 Hz, 1H, H_{29}), 7.95 (d, J = 8.0 Hz, 1H, H_{28}), 7.82 (t, J = 5.7 Hz, 1H, H_{30}), 7.74 (d, J = 8.7 Hz, 1H, H_{26}), 7.25 (s, 1H, 1 × H_{27}), 6.82 (s, 1H, 1 × H_{27}), 6.76 (t, J = 5.7 Hz, 1H, H_{31}), 5.78 (d, J = 4.4 Hz, 1H, –O*H*), 5.73 (dd, J = 15.4, 5.6 Hz, 1H, H_5), 5.54 (ddd, J = 15.5, 6.2, 1.3 Hz, 1H, H_6), 4.53 (td, J = 8.0, 5.8 Hz, 1H, H_{16}), 4.37 – 4.18 (m, 3H, H_4, H_7, H_9), 3.70 (d, J = 7.4 Hz, 1H, H_8), 3.28 – 3.20 (m, 2H, H_1), 3.00 (m, 2H, H_{12}), 2.89 (m, 2H, H_{15}), 2.48 – 2.42 (m, 1H, 1 × H_{17}), 2.34 (dd, J = 15.2, 8.0 Hz, 1H, 1 × H_{17}), 2.09 (dtt, J = 14.1, 9.2, 4.7 Hz, 2H, H_{18}), 1.56 (dd, J = 13.7, 6.8 Hz, 1H, H_{11}),

1.47 (s, 15H, 9 × H_{21}, 2 × H_{19}, 2 × H_2, 2 × H_3), 1.39 (s, 10H, 9 × H_{21}, 1 × H_{10}), 1.36 (s, 10H, 9 × H_{23}, 1 × H_{10}), 1.35 – 1.32 (m, 4H, 2 × H_{13}, 2 × H_{14}), 1.22 (t, *J* = 4.7 Hz, 20H, 10 × CH_2), 0.88 – 0.71 (m, 9H, 6 × H_{22}, 3 × H_{20}).

^{13}C NMR (126 MHz, DMSO-*d_6*) δ 172.3 (C), 171.4 (C), 171.2 (C), 170.5 (C), 167.8 (C), 163.1 (C), 155.6 (C), 155.2 (C), 152.1 (C), 133.4 (CH), 128.6 (CH), 82.9 (C), 78.1 (C), 77.3 (C), 71.3 (CH), 65.0 (CH), 51.3 (CH), 49.8 (CH), 49.2 (CH), 40.7 (CH_2), 38.4 (CH_2), 37.2 (CH_2), 35.2 (CH_2), 31.6 (CH_2), 31.3 (CH_2), 29.1 (4 × CH_2), 29.0 (2 × CH_2), 28.8 (CH_2), 28.7 (CH_2), 28.3 (3 × CH_3), 28.0 (3 × CH_3), 27.6 (3 × CH_3), 26.9 (CH_2), 26.4 (CH_2), 25.2 (CH_2), 25.1 (CH_2), 24.3 (CH), 23.0 (CH_3), 22.1 (CH_2), 21.4 (CH_3), 14.0 (CH_3). Two carbons obscured by DMSO-d_6 (2 × CH_2).

HRMS-CI (m/z): [M + H]+ calcd for $C_{53}H_{97}N_{12}O_{12}$, 1093.7343; found, 1093.7398.

*Synthesis of the L-Asn-L-Arg-amino alcohol **S28**.*

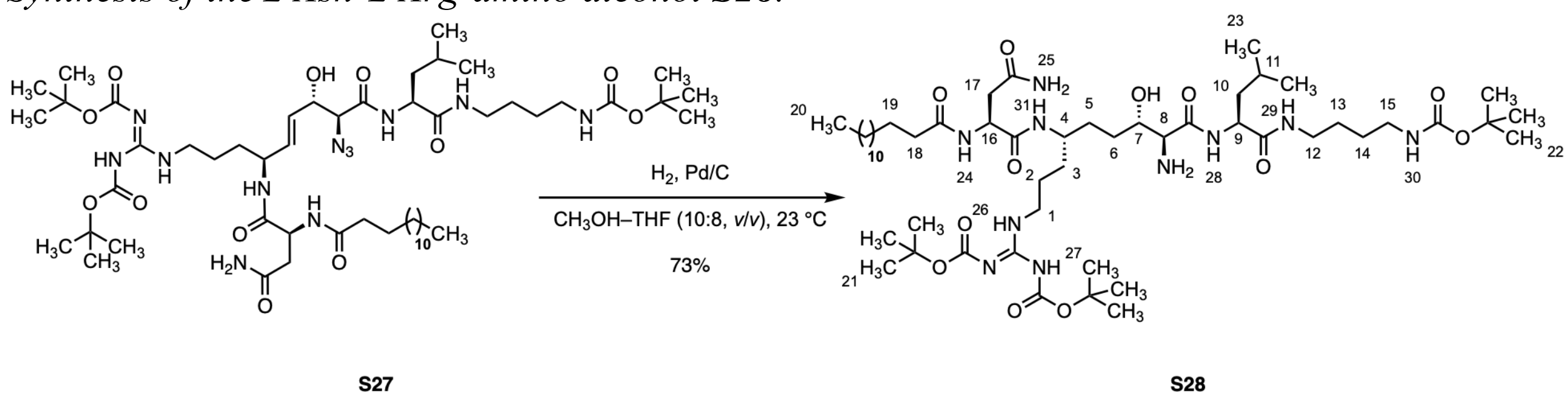

A round-bottom flask was charged with L-Asn-L-Arg-azido alcohol **S27** (82.5 mg, 75.5 µmol, 1 equiv) and palladium on carbon (Pd/C, 22.0 mg, 25 % *w*/*w* loading, dry matrix) then backfilled with argon gas. Methanol/tetrahydrofuran (1.9 mL, 10:8 *v*/*v*) was added and the reaction mixture was purged with dihydrogen gas for 5 min. The reaction mixture was stirred under an atmosphere of dihydrogen (1 atm; balloon) for 3 h at 23 °C. The product mixture was filtered through a pad of celite and was further washed with methanol (2 × 5 mL). The filtrate was concentrated, and the residue was redissolved in 10% ethyl acetate–hexane (5 mL). The product mixture was concentrated again to afford *the L-Asn-L-Arg-amino alcohol* **S28** as a white solid film (58.5 mg, 73%). The product obtained in this way was judged to be of >95% purity (^{1}H NMR analysis) and was advanced without purification.

R_f = 0.54 (10% methanol–dichloromethane + 0.1% Et_3N; UV, long streak)

$[\alpha]_D^{20}$: –10.6 (*c* 0.32, CH_3OH)

^{1}H NMR (500 MHz, DMSO-*d*$_6$) δ 11.50 (s, 1H, H_{27}), 8.21 (t, *J* = 5.5 Hz, 1H, H_{26}), 8.02 – 7.84 (m, 3H, H_{24}, H_{28}, H_{29}), 7.46 (d, *J* = 8.9 Hz, 1H, H_{31}), 7.25 (d, *J* = 9.0 Hz, 1H, 1 × H_{25}), 6.93 – 6.82 (m, 1H, 1 × H_{25}), 6.75 (t, *J* = 5.8 Hz, 1H, H_{15}), 4.88 (s, 1H, –O*H*), 4.48 (q, *J* = 7.3 Hz, 1H, H_{16}), 4.22 (q, *J* = 7.3 Hz, 1H, H_9), 3.64 (app s, 1H, H_4), 3.44 (app s, 1H, H_7), 3.22 (d, *J* = 6.3 Hz, 2H, H_1), 3.13 (d, *J* = 6.5 Hz, 1H, H_8), 3.00 (t, *J* = 5.4 Hz, 2H, H_{12}), 2.88 (d, *J* = 6.1 Hz, 2H, H_{15}), 2.48 – 2.40 (m, 1H, 1 × H_{17}), 2.33 (dd, *J* = 15.1, 7.6 Hz, 1H, 1 × H_{17}), 2.06 (td, *J* = 6.1, 3.3 Hz, 2H, H_{18}), 1.58 (dq, *J* = 12.9, 6.1 Hz, 1H, H_{11}), 1.46 (s, 15H, 2 × H_2, 2 × H_3, 2 × H_{10}, 9 × H_{21}), 1.38 (s, 10H, 1 × H_{19}, 9 × H_{21}), 1.36 (s, 10H, 1 × H_{19}, 9 × H_{22}), 1.35 – 1.30 (m, 4H, 2 × H_{13}, 2 × H_{14}), 1.22 (24H, 2 × H_5, 2 × H_6, 20 × CH_2), 0.85 (dt, *J* = 12.7, 6.3 Hz, 9H, 3 × H_{20}, 6 × H_{23}).

^{13}C NMR (126 MHz, DMSO-*d*$_6$) δ 172.2 (C), 171.8 (C), 171.5 (C), 170.6 (C), 163.1 (C), 155.6 (C), 155.2 (C), 152.2 (C), 82.9 (C), 78.1 (C), 77.4 (C), 72.9 (CH), 59.3 (CH), 50.9 (CH), 50.0 (CH), 48.4 (CH), 41.1 (CH_2), 40.4 (CH_2), 38.3 (CH_2), 37.3 (CH_2), 35.2 (CH_2), 31.3 (CH_2), 31.0 (CH_2), 29.1 (5 × CH_2), 29.0 (2 × CH_2), 28.9 (CH_2), 28.7 (2 × CH_2), 28.3 (3 × CH_3), 28.0 (3 × CH_3), 27.6 (3 × CH_3), 26.9 (CH_2), 26.4 (CH_2), 25.3 (CH_2), 25.2 (CH_2), 24.2 (CH), 23.1 (CH_3), 22.1 (CH_2), 21.5 (CH_3), 14.0 (CH_3). One carbon obscured by DMSO-*d*$_6$ (CH_2). One quaternary carbon not observed.

HRMS-CI (m/z): [M + H]+ calcd for $C_{53}H_{101}N_{10}O_{12}$, 1069.7595; found, 1069.7546.

*Synthesis of the protected L-Asn-L-Arg pre-rhabdobranin B **S29**.*

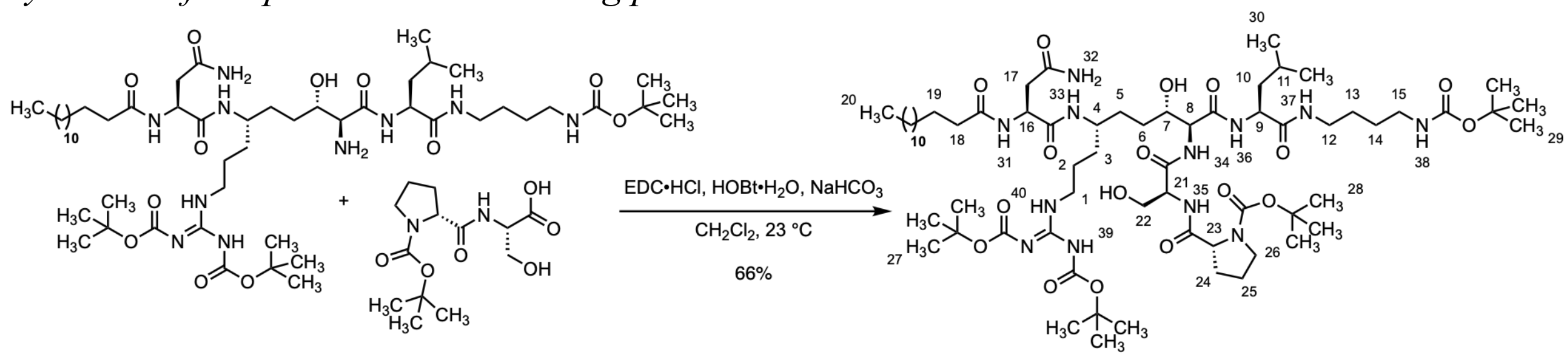

S28 **6** **S29**

The following procedure has been adapted from the literature.[12] To a solution of L-Asn-L-Arg-amino alcohol **S28** (28.0 mg, 26.2 µmol, 1 equiv) in dichloromethane (0.33 mL, C = 0.080 M) were added sodium hydrogen carbonate (2.70 mg, 31.9 µmol, 1.22 equiv), 1-hydroxybenzotriazole hydrate (HOBt•H2O, 6.90 mg, 45.0 µmol, 1.72 equiv) and Boc-D-Pro-Ser **6** (10.3 mg, 34.0 µmol, 1.30 equiv) at 23 °C. The reaction mixture was cooled to 0 °C and 1-ethyl-3-(3-dimethylaminopropyl) carbodiimide hydrogen chloride (EDC•HCl, 6.10 mg, 31.9 µmol, 1.22 equiv) was added. The cooling bath was removed, and the reaction mixture was stirred for 16 h at 23 °C. The product mixture was diluted with brine (0.5 mL). The diluted product mixture was transferred to a separatory funnel and the layers that formed were separated. The aqueous phase was extracted with dichloromethane (3 × 1 mL). The combined organic extracts were dried over sodium sulfate. The dried solution was filtered, and the filtrate was concentrated. The residue was purified by flash column chromatography (eluting with 100% dichloromethane initially, grading to 10 % methanol– dichloromethane, linear gradient) to afford the *protected L-Asn-L-Arg pre-rhabdobranin B* **S29** as a white solid (23.2 mg, 66%).

Note: For compounds containing the Boc-Pro-Ser fragment, several NMR resonances were broad or partially resolved. These features are consistent with slow conformational exchange of Boc amino acid rotamers on the NMR timescale; therefore, overlapping or broadened resonances are reported as observed without assigning individual rotameric species.[14]

R_f = 0.28 (5% methanol–dichloromethane; UV)

$[\alpha]_D^{20}$: –6.5 (*c* 0.24, $CHCl_3$)

^{1}H NMR (500 MHz, DMF-d_7) δ 11.66 (s, 1H, H_{39}), 8.35 (t, *J* = 5.8 Hz, 1H, H_{40}), 8.05 (s, 1H, H_{31}), 7.74 (d, *J* = 6.6 Hz, 1H, H_{36}), 7.57 (dd, *J* = 21.4, 14.1 Hz, 2H, 1 × H_{32}), 6.96 (d, *J* = 28.8 Hz, 1H, 1 × H_{32}), 6.68 (s, 1H, H_{38}), 5.42 (app q, *J* = 38.4 Hz, 1H, –CH_2OH or –CHO*H*), 4.73 – 4.62 (m, 1H, H_{16}), 4.41 – 4.23 (m, 4H, H_8, H_9, H_{21}, H_{23}), 3.91 (s, 1H, 1 × H_{22}), 3.81 (s, 3H, H_4, H_7, 1 × H_{22}), 3.36 (d, *J* = 6.7 Hz, 3H, 2 × H_1, 1 × H_{26}*), 3.14 (d, *J* = 6.2 Hz, 2H, H_{12}), 3.04 (d, *J* = 6.5 Hz, 2H, H_{15}), 2.72 – 2.52 (m, 2H, H_{17}), 2.23 (t, *J* = 7.7 Hz, 2H, H_{18}), 1.63 – 1.56 (m, 6H), 1.52 (s, 15H), 1.44 (s, 18H), 1.40 (s, 15H), 1.28 (s, 22H), 0.88 (tt, *J* = 12.0, 4.5 Hz, 9H, 3 × H_{20}, 6 × H_{29}). *Second H_{26} shift obscured by H_2O signal.

Owing to substantial signal broadening, attributed primarily to conformational exchange associated with the four Boc-protecting groups, together with extensive signal overlap in this high-molecular-weight compound, unambiguous assignment of all resonances was not possible. Accordingly, only resonances that could be confidently assigned on the basis of COSY and HSQC correlations are reported; the remaining signals (H_2, H_3, H_5, H_6, H_{10}, H_{11}, H_{13}, H_{14}, H_{19}, H_{24}, H_{25}, H_{27}, H_{28}, H_{30}, H_{33}, H_{34}, H_{35}, and H_{37}) were left unassigned.

^{13}C NMR (126 MHz, DMF) δ 173.8 (C), 173.3 (C), 173.2 (C), 173.0 (C), 172.1 (C), 164.7 (C), 157.1 (C), 156.8 (C), 153.7 (C), 84.0 (C), 80.3 (C), 79.2 (C), 78.4 (C), 72.6* (CH), 63.3* (CH_2), 61.2* (CH), 59.5* (CH), 53.1 (CH), 51.6 (CH), 49.8 (CH), 47.9 (CH_2), 41.6 (CH_2), 41.5 (CH_2), 40.9 (CH_2), 39.7 (CH_2), 38.4 (CH_2), 36.6 (CH_2), 32.7 (CH_2), 28.9 (5 × CH_3), 28.7 (4 × CH_3), 28.3 (3 × CH_3), 27.6 (CH_2), 26.6 (CH_2), 25.5 (CH), 24.4 (CH_2), 23.9 (CH_3), 23.4 (CH_2), 21.9 (CH_2), 21.3 (CH_3), 14.6 (CH_3). Three quaternary carbons not observed. 14 carbons obscured by DMF-d_7 or not clearly observed (CH_2). *Assigned using HSQC analysis.
HRMS-CI (m/z): [M + H]+ calcd for $C_{66}H_{121}N_{12}O_{17}$, 1353.8967; found, 1353.8901.

Achiral SFC trace chromatogram of compound **S29**.

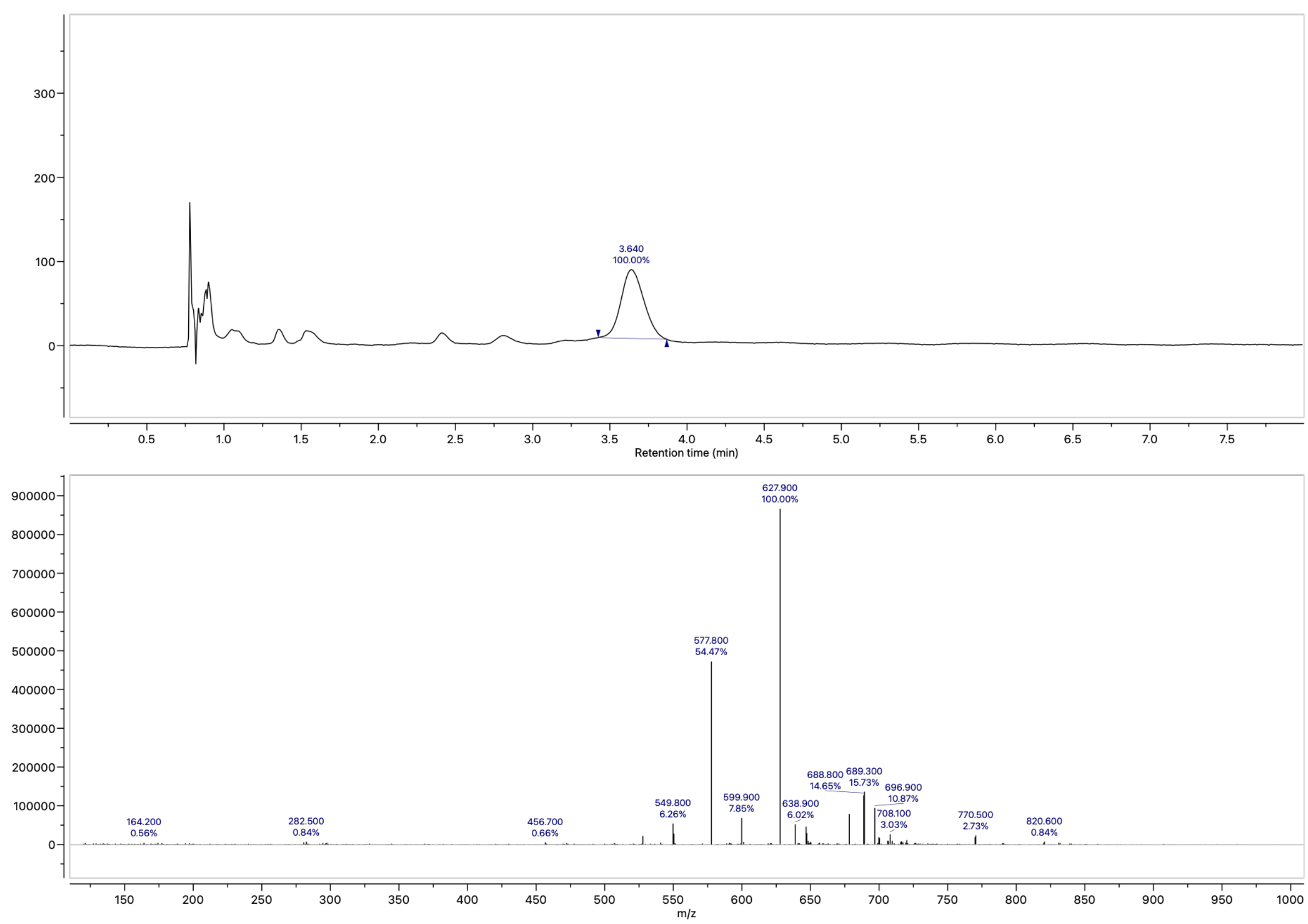

Column type: Agilent ZORBAX RX-SIL 4.6 × 150 mm, 5 μm.

Flow rate: 2 mL/min

Method: Isocratic

Time (min)	CO_2 (%)	Methanol (%)
0.00	80.0	20.0
8.00	80.0	20.0

Retention time: 3.64 min at λ = 210 nm.

Observed masses: 628 [(M–Boc)/2 + H^+] and 579 [(M–2Boc)/2 + H^+].

Synthesis of the L*-Asn-*L*-Arg pre-rhabdobranin B* ***S30****.*

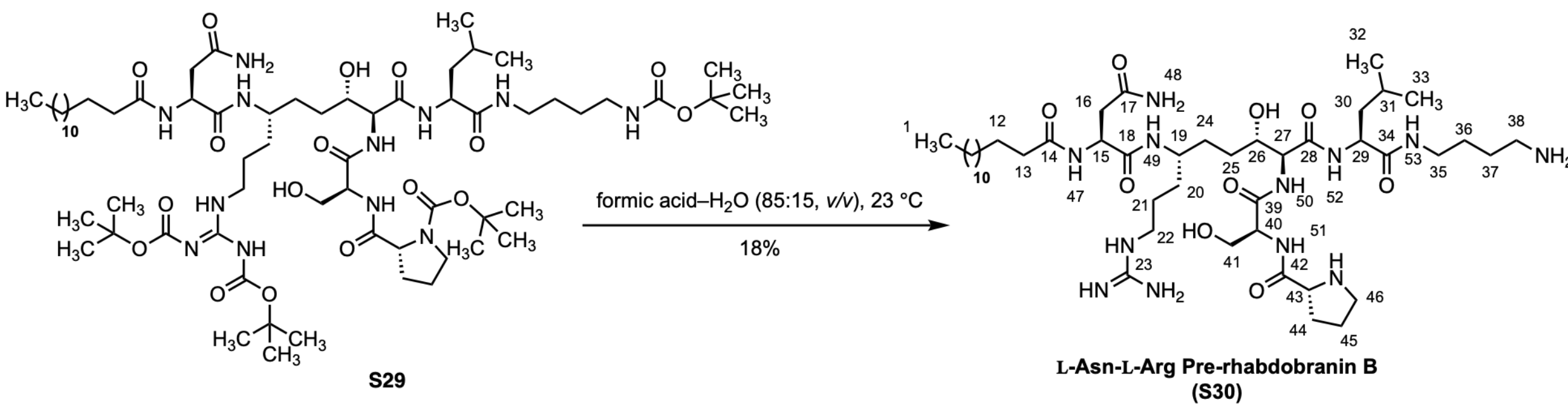

To protected L-Asn-L-Arg pre-rhabdobranin B **S29** (20.8 mg, 15.4 μmol, 1 equiv), was added 85% (*v*/*v*) aqueous formic acid solution (0.26 mL, C = 0.060 M). The reaction mixture was stirred for 24 h at 23 °C. The product mixture was lyophilized, diluted with methanol, and purified by reverse-phase HPLC with a Puriflash C18 column (15 μm, 10 mm × 150 mm) using an acetonitrile–water gradient (+0.1% formic acid) for 0–40 min, 5–53%, 3 mLmin^{-1} to afford the *L-Asn-L-Arg pre-rhabdobranin B* **S30** as a white solid (2.7 mg, 18%).

Method:

0–5.5 min, 5% acetonitrile;
5.5–24.0 min, 5%–53%;
24.0–40.0 min, 53%.

Retention time: 25.0 min.

Note**:** The deprotection step using formic acid[25] proceeds quantitatively to the desired product. The low yield likely reflects the challenges associated with purifying a polycationic species with low UV activity. Nonetheless, as this procedure furnished sufficient material for complete spectroscopic characterization, the deprotection and purification sequence was not further optimized.

^{1}H NMR (500 MHz, DMSO-d_6) δ 8.45 (t, J = 9.9 Hz, 1H, H_{51}), 8.22 (dd, J = 8.4, 4.5 Hz, 1H, H_{50}), 8.14 (d, J = 7.7 Hz, 1H, H_{52}), 8.07 (dd, J = 7.9, 3.3 Hz, 1H, H_{47}), 7.65 (td, J = 12.4, 4.1 Hz, 2H, 1 × H_{49}, 1 × H_{53}), 7.47 – 7.28 (m, 1H, 1 × H_{48}), 6.88 (s, 1H, 1 × H_{48}), 4.47 (q, J = 7.3 Hz, 1H, H_{15}), 4.34 (d, J = 6.4 Hz, 1H, H_{40}), 4.27 – 4.16 (m, 2H, 1 × H_{27}, 1 × H_{29}), 3.04 (d, J = 9.4 Hz, 4H, 2 × H_{22}, 2 × H_{35}), 2.93 – 2.85 (m, 1H, 1 × H_{46}), 2.83 – 2.78 (m, 1H, 1 × H_{46}), 2.76 – 2.68 (m, 2H, 2 × H_{38}), 2.46 – 2.42 (m, 1H, 1 × H_{16}), 2.41 – 2.32 (m, 1H, 1 × H_{16}), 2.09 (t, J = 7.6 Hz, 2H, 2 × H_{13}), 1.96 (dt, J = 13.3, 6.1 Hz, 1H, 1 × H_{44}), 1.69 (d, J = 5.9 Hz, 1H, 1 × H_{44}), 1.60 (ddd, J = 11.0, 7.5, 3.9 Hz, 3H, 1 × H_{31}, 1 × H_{36}, 1 × H_{45}), 1.47 (d, J = 29.9 Hz, 15H, 2 × H_{12}, 1 × H_{20}, 2 × H_{21}, 2 × H_{24}, 2 × H_{25}, 2 × H_{30}, 1 × H_{36}, 2 × H_{37}, 1 × H_{45}), 1.23 (d, J = 3.9 Hz, 20H, 10 × CH_2, myristoyl), 0.94 – 0.62 (m, 9H, 3 × H_1, 3 × H_{32}, 3 × H_{33}).

^{13}C NMR (126 MHz, DMSO-d_6) δ 174.5 (C), 172.8 (C), 172.3 (C), 172.1 (C), 171.4 (C), 171.1 (C), 170.7 (C), 157.8 (C), 71.4 (CH), 62.3 (CH_2), 60.4 (CH_2), 58.4 (CH), 55.0 (CH), 51.9 (CH), 50.6 (CH), 48.6 (CH), 47.0 (CH_2), 41.0 (CH_2), 40.6 (CH_2), 38.9 (CH_2), 38.3 (CH_2), 38.0 (CH_2), 35.7 (CH_2), 31.8 (2 × CH_2), 31.1 (CH_2), 30.7 (CH_2), 29.9 (2 × CH_2), 29.5 (3 × CH_2), 29.4 (CH_2), 29.3 (CH_2), 29.2 (2 × CH_2), 26.2 (2 × CH_2), 25.7 (CH_2), 25.3 (CH_2), 24.6 (CH), 23.6 (CH_3), 22.6 (2 × CH_2), 21.6 (CH_3), 14.4 (CH_3).

HRMS-CI (m/z): $[M + H]^+$ calcd for $C_{46}H_{89}N_{12}O_9$, 953.6870; found, 953.6876.

Catalog of nuclear magnetic resonance data

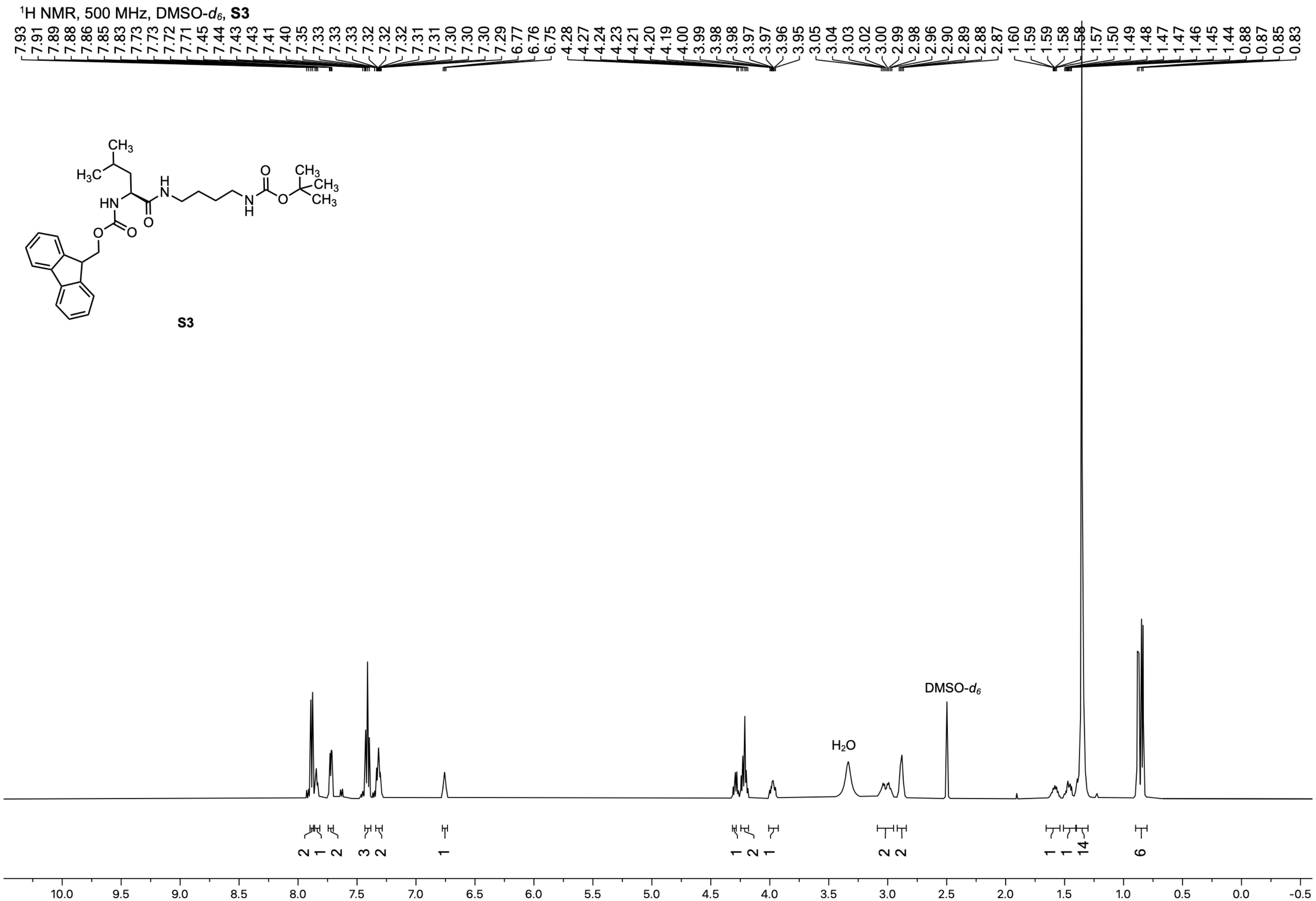

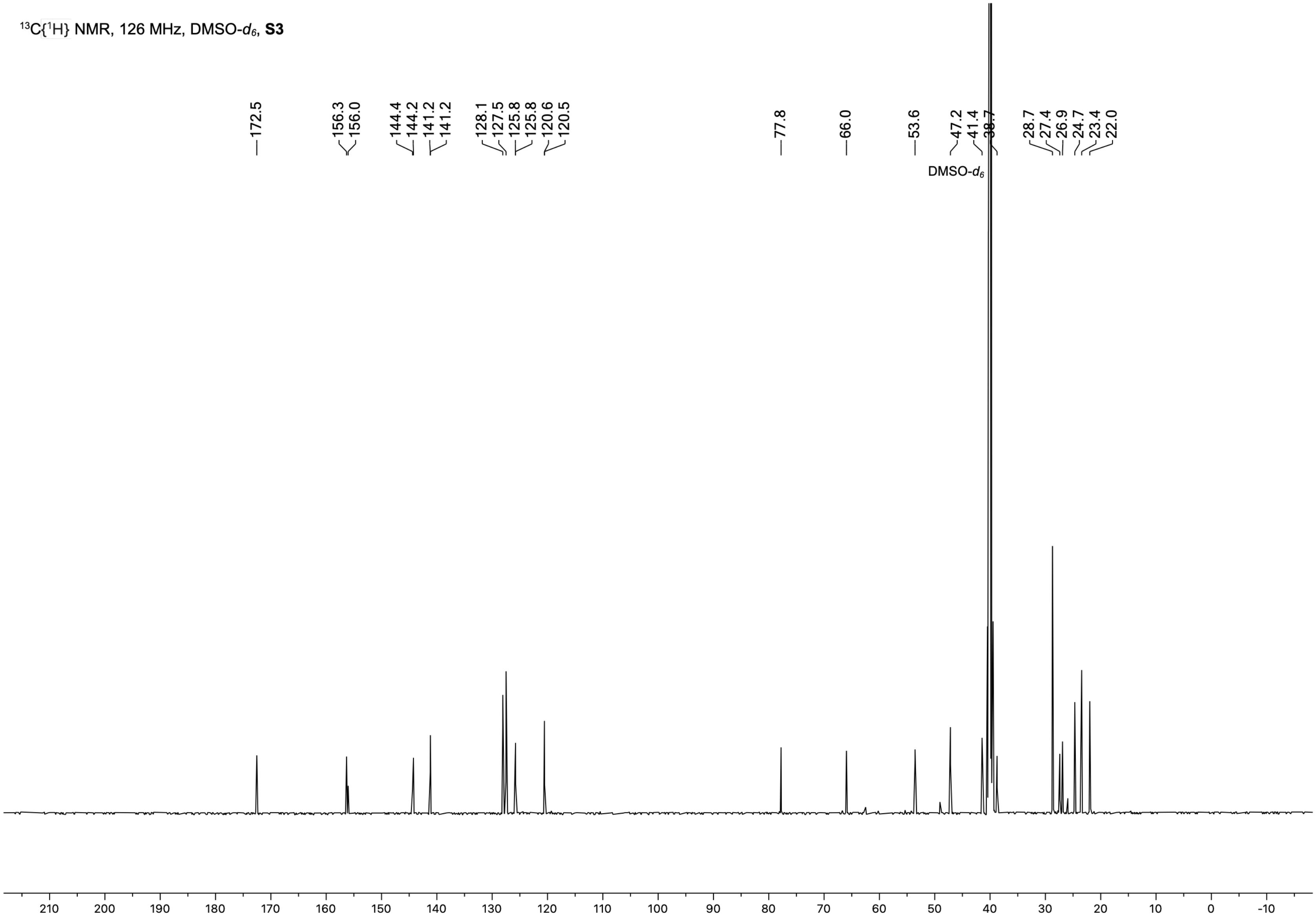
$^{13}C\{^1H\}$ NMR, 126 MHz, DMSO-d_6, **S3**
172.5
156.3
156.0
144.4
144.2
141.2
141.2
128.1
127.5
125.8
125.8
120.6
120.5
77.8
66.0
53.6
47.2
41.4
38.7
28.7
27.4
26.9
24.7
23.4
22.0
DMSO-d_6
210 200 190 180 170 160 150 140 130 120 110 100 90 80 70 60 50 40 30 20 10 0 -10

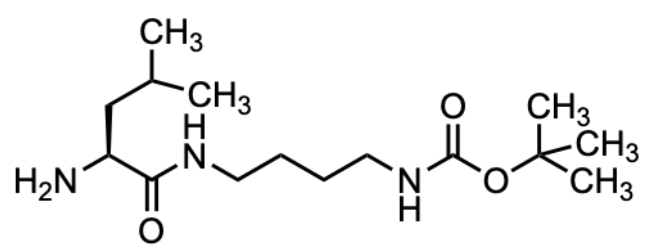

5

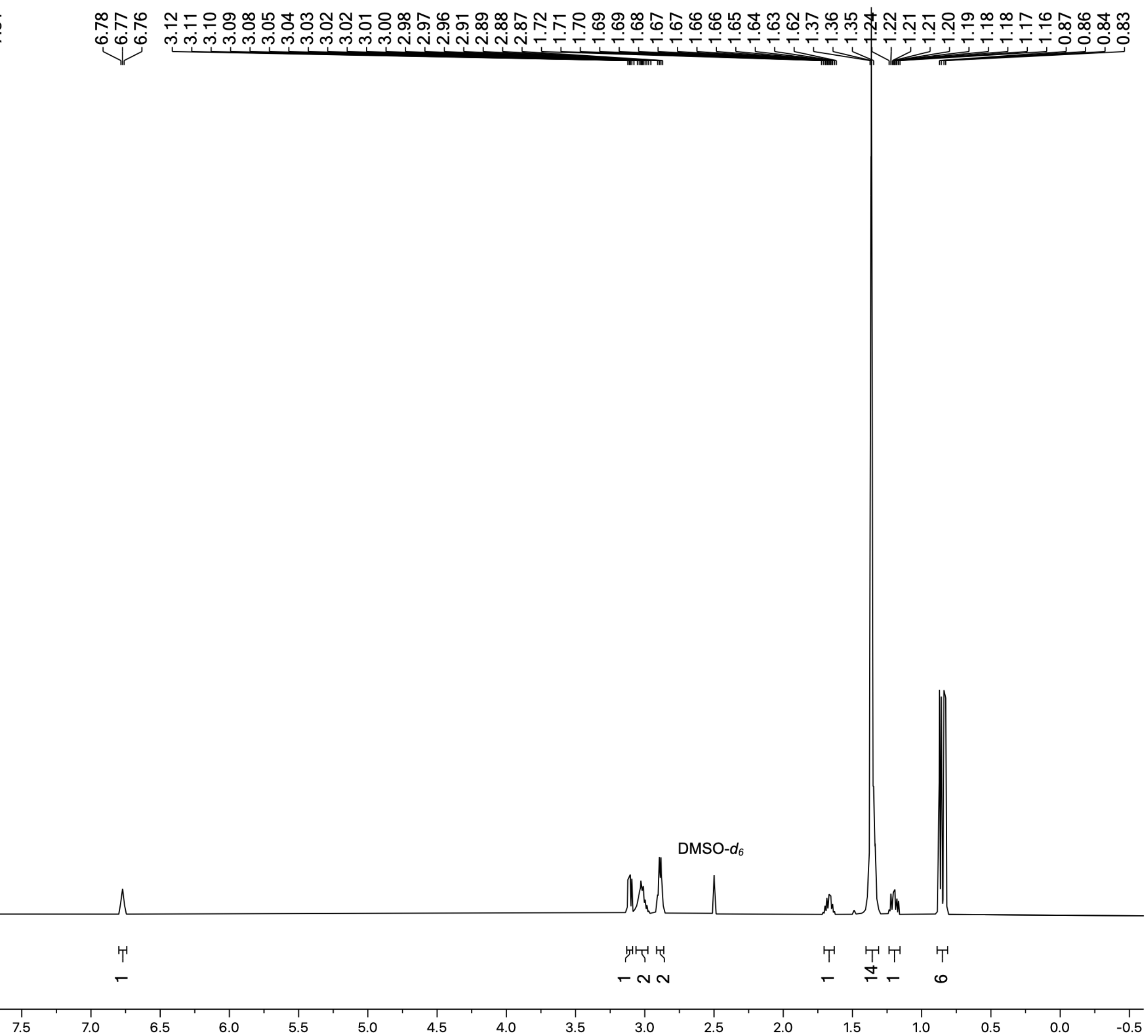

^{1}H NMR, 500 MHz, DMSO-d_6, **5**
7.83
7.82
7.81
6.78
6.77
6.76
3.12
3.11
3.10
3.09
3.08
3.05
3.04
3.03
3.02
3.02
3.01
3.00
2.98
2.97
2.96
2.91
2.89
2.88
2.87
1.72
1.71
1.70
1.69
1.69
1.68
1.67
1.67
1.66
1.65
1.64
1.63
1.62
1.37
1.36
1.35
1.24
1.22
1.21
1.21
1.20
1.19
1.18
1.18
1.17
1.16
0.87
0.86
0.84
0.83
DMSO-d_6
1
1
1
2
2
1
14
1
6
10.0
9.5
9.0
8.5
8.0
7.5
7.0
6.5
6.0
5.5
5.0
4.5
4.0
3.5
3.0
2.5
2.0
1.5
1.0
0.5
0.0
-0.5

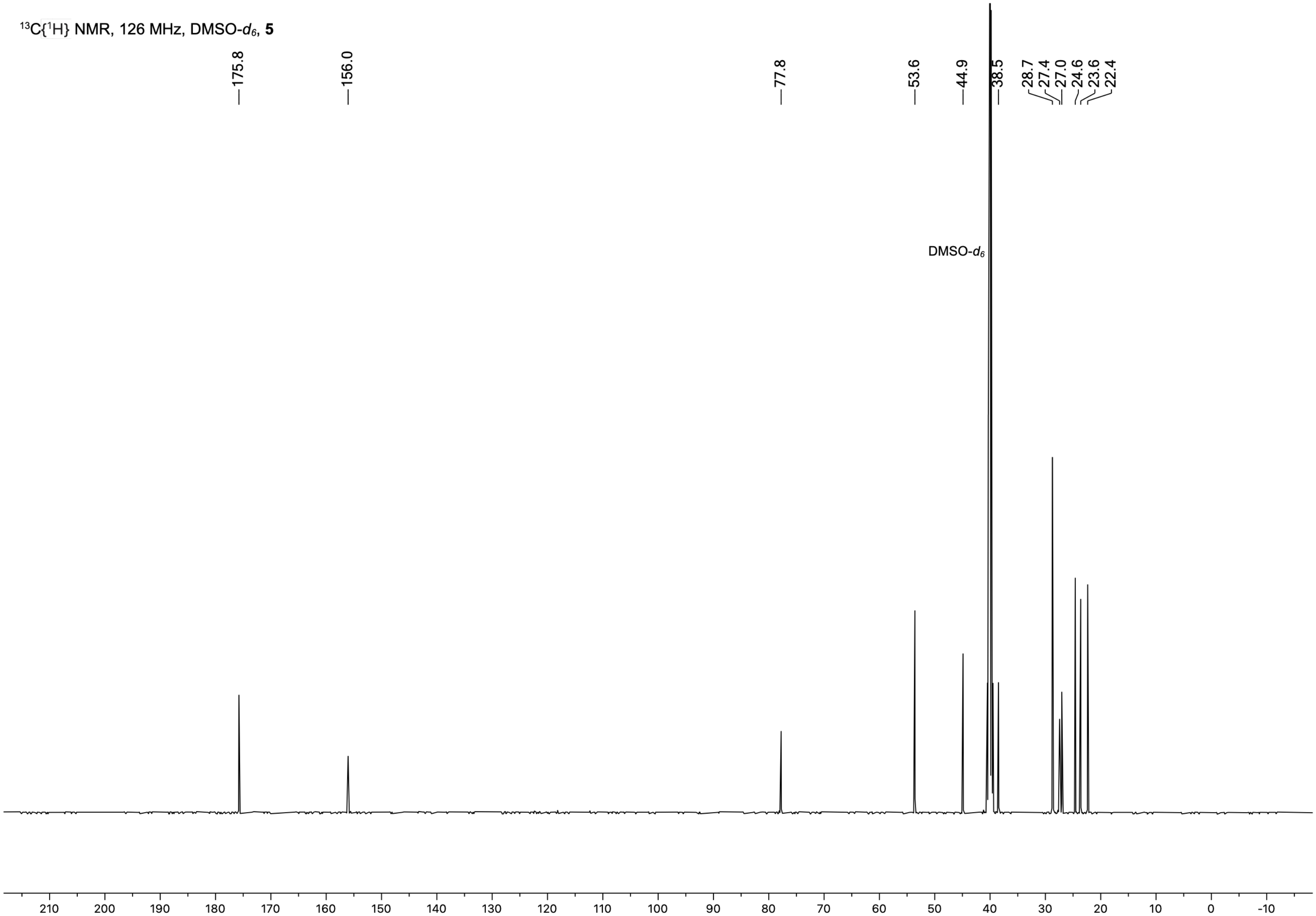

$^{13}C\{^1H\}$ NMR, 126 MHz, DMSO-d_6, **5**
175.8
156.0
77.8
53.6
44.9
38.5
28.7
27.4
27.0
24.6
23.6
22.4
DMSO-d_6
210 200 190 180 170 160 150 140 130 120 110 100 90 80 70 60 50 40 30 20 10 0 -10

^{1}H NMR, 500 MHz, DMSO-d_6, **S6**

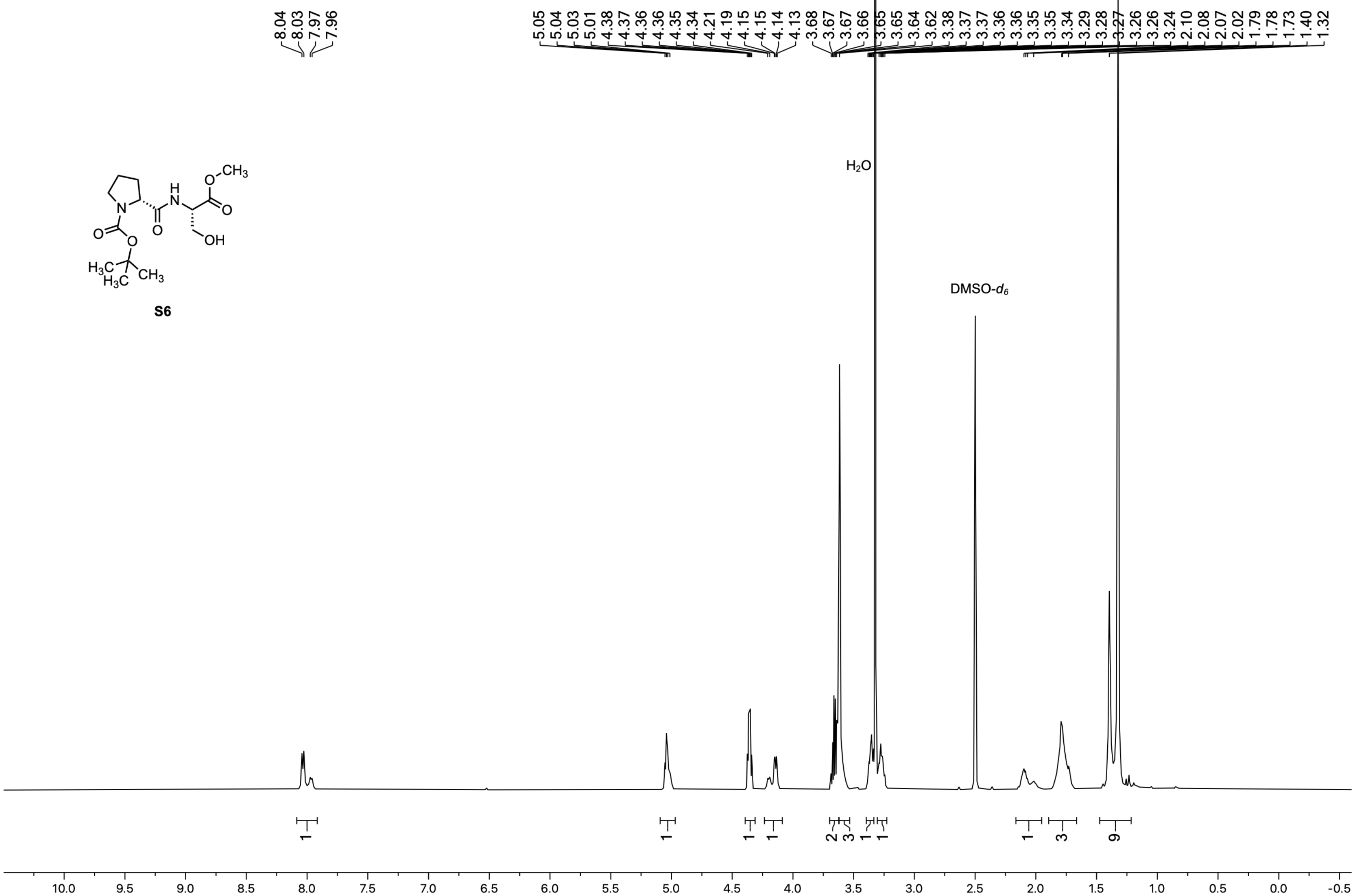

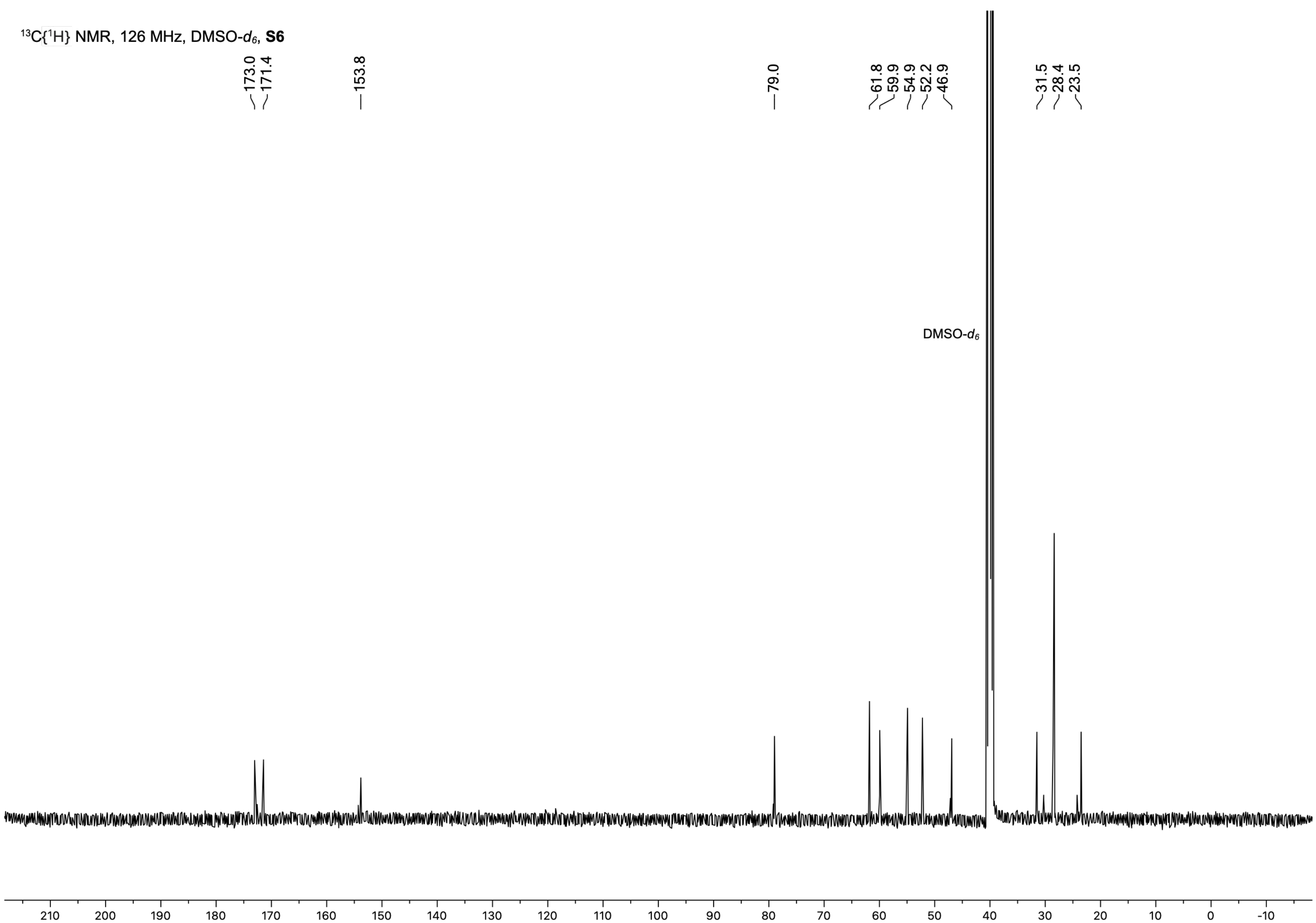

$^{13}C\{^{1}H\}$ NMR, 126 MHz, DMSO-d_6, **S6**
173.0
171.4
153.8
79.0
61.8
59.9
54.9
52.2
46.9
31.5
28.4
23.5
DMSO-d_6
210
200
190
180
170
160
150
140
130
120
110
100
90
80
70
60
50
40
30
20
10
0
-10

^{1}H NMR, 500 MHz, DMSO-d_6, **6**

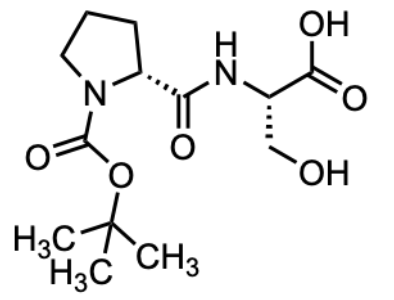

6

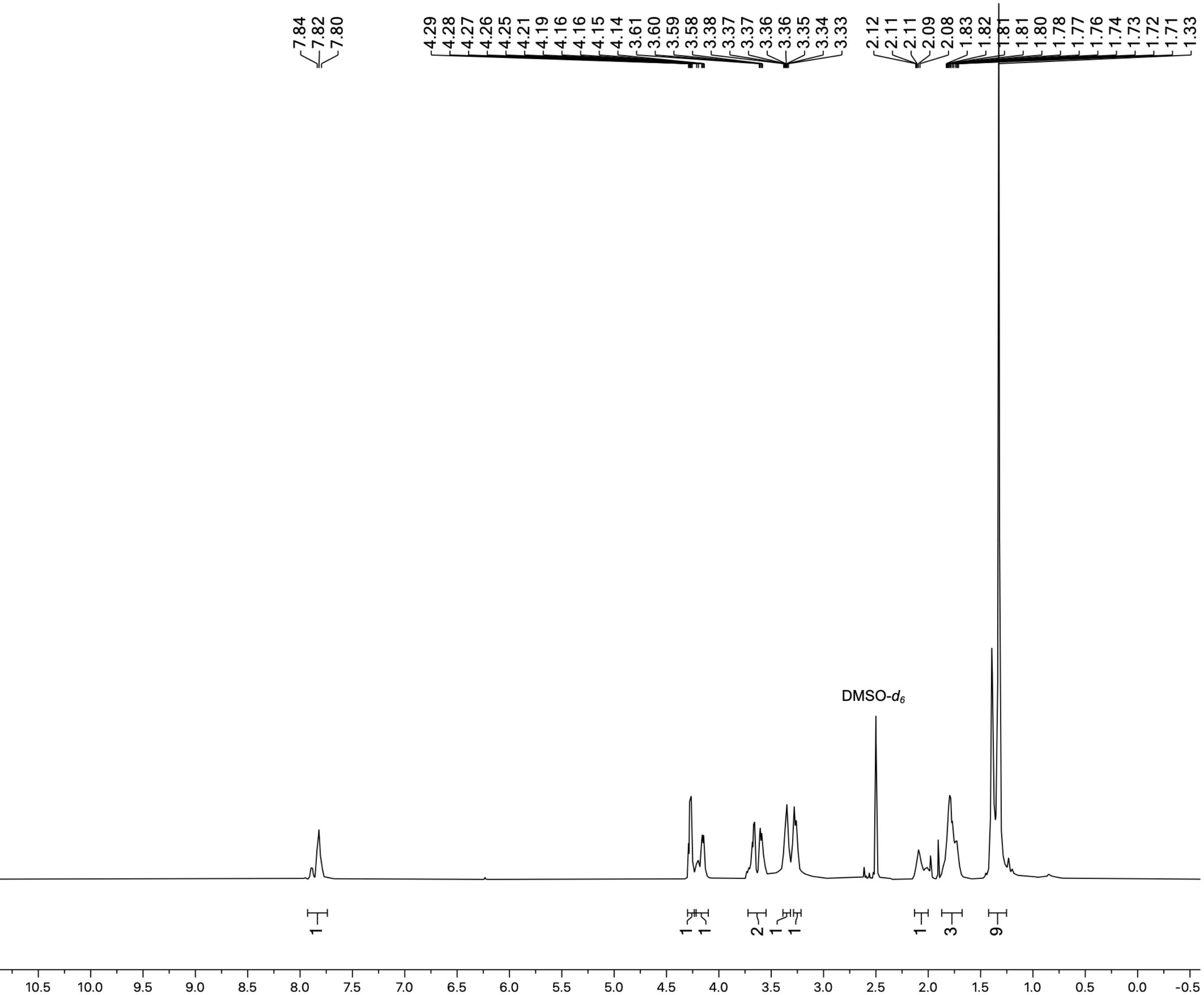

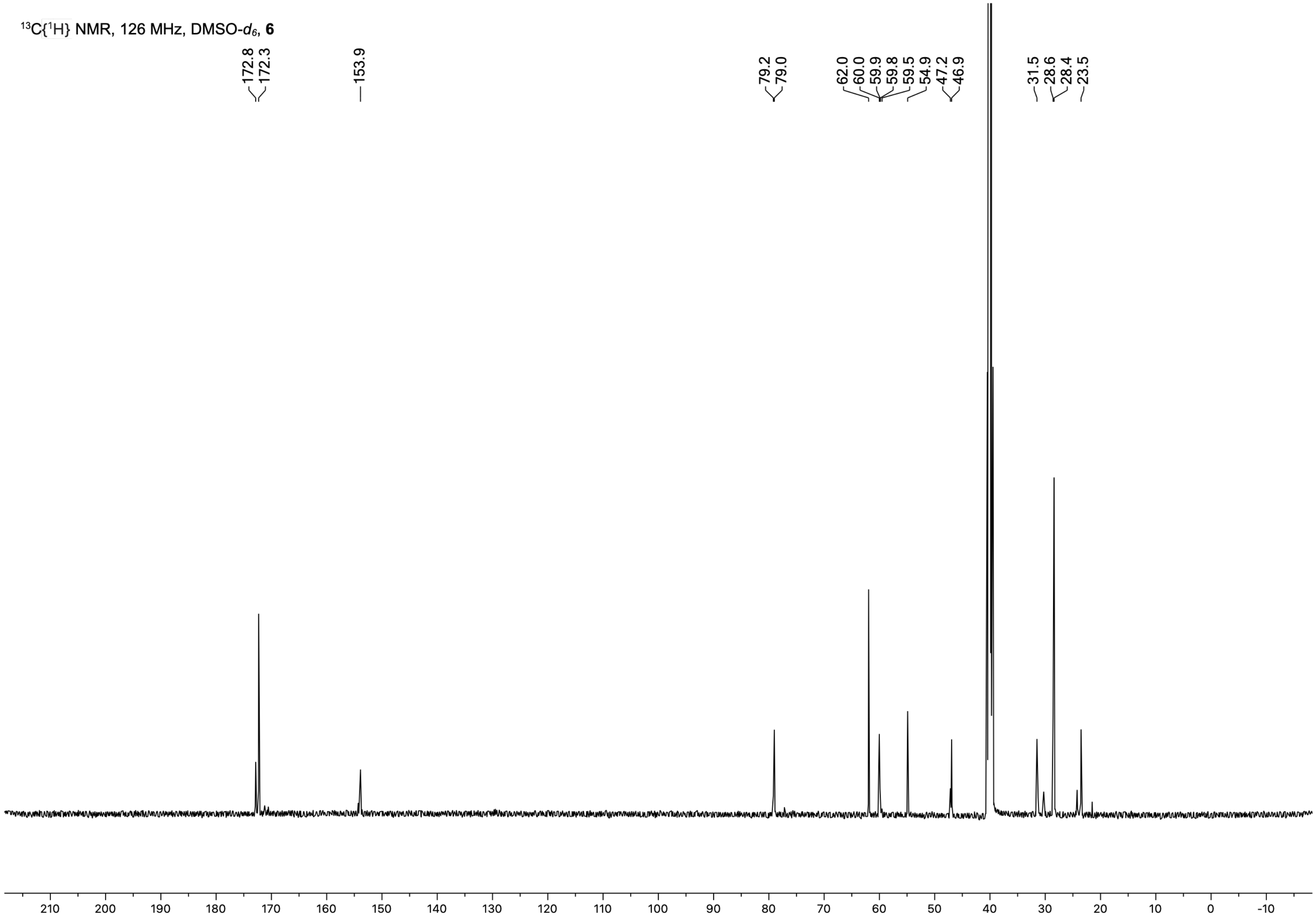

$^{13}C\{^1H\}$ NMR, 126 MHz, DMSO-d_6, **6**
172.8
172.3
153.9
79.2
79.0
62.0
60.0
59.9
59.8
59.5
54.9
47.2
46.9
31.5
28.6
28.4
23.5
210
200
190
180
170
160
150
140
130
120
110
100
90
80
70
60
50
40
30
20
10
0
-10

^{1}H NMR, 500 MHz, DMSO-d_6, **10**

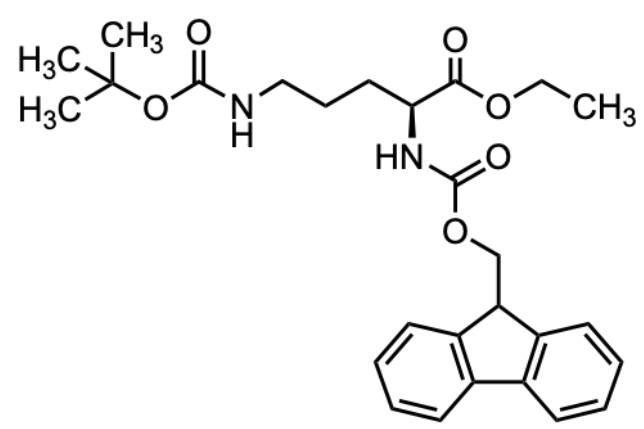

10

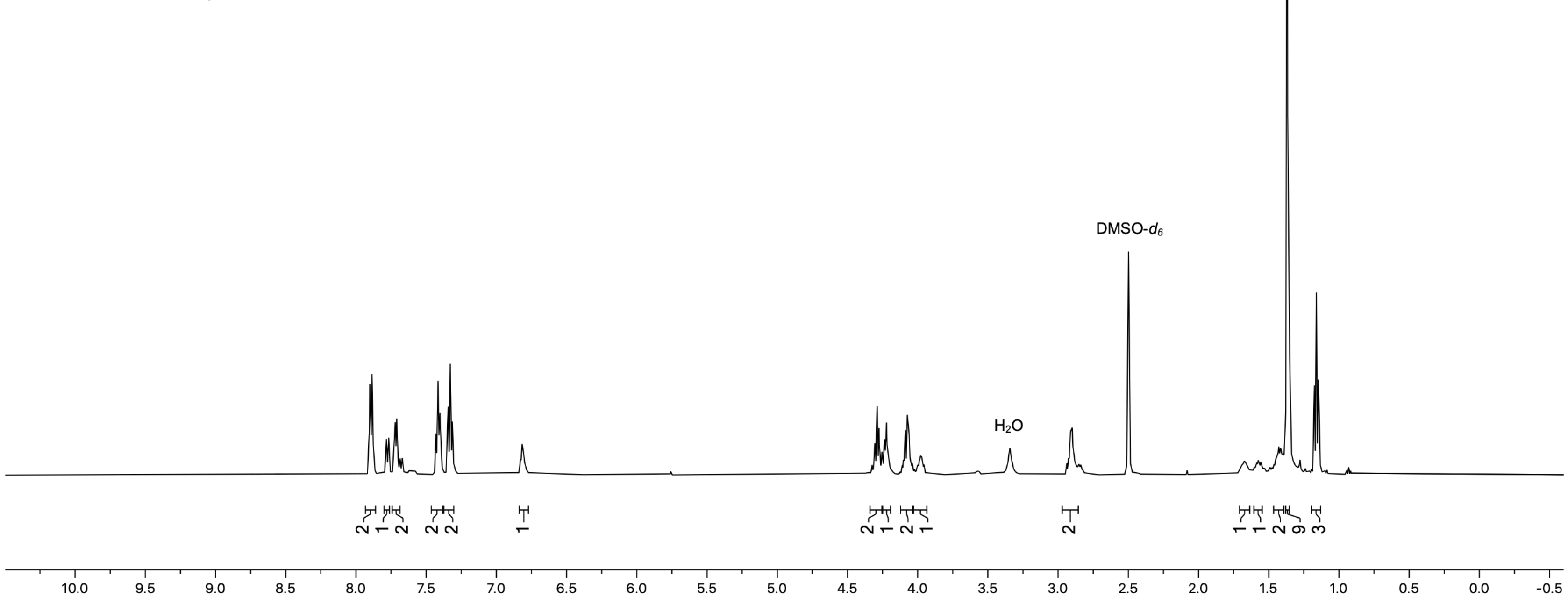

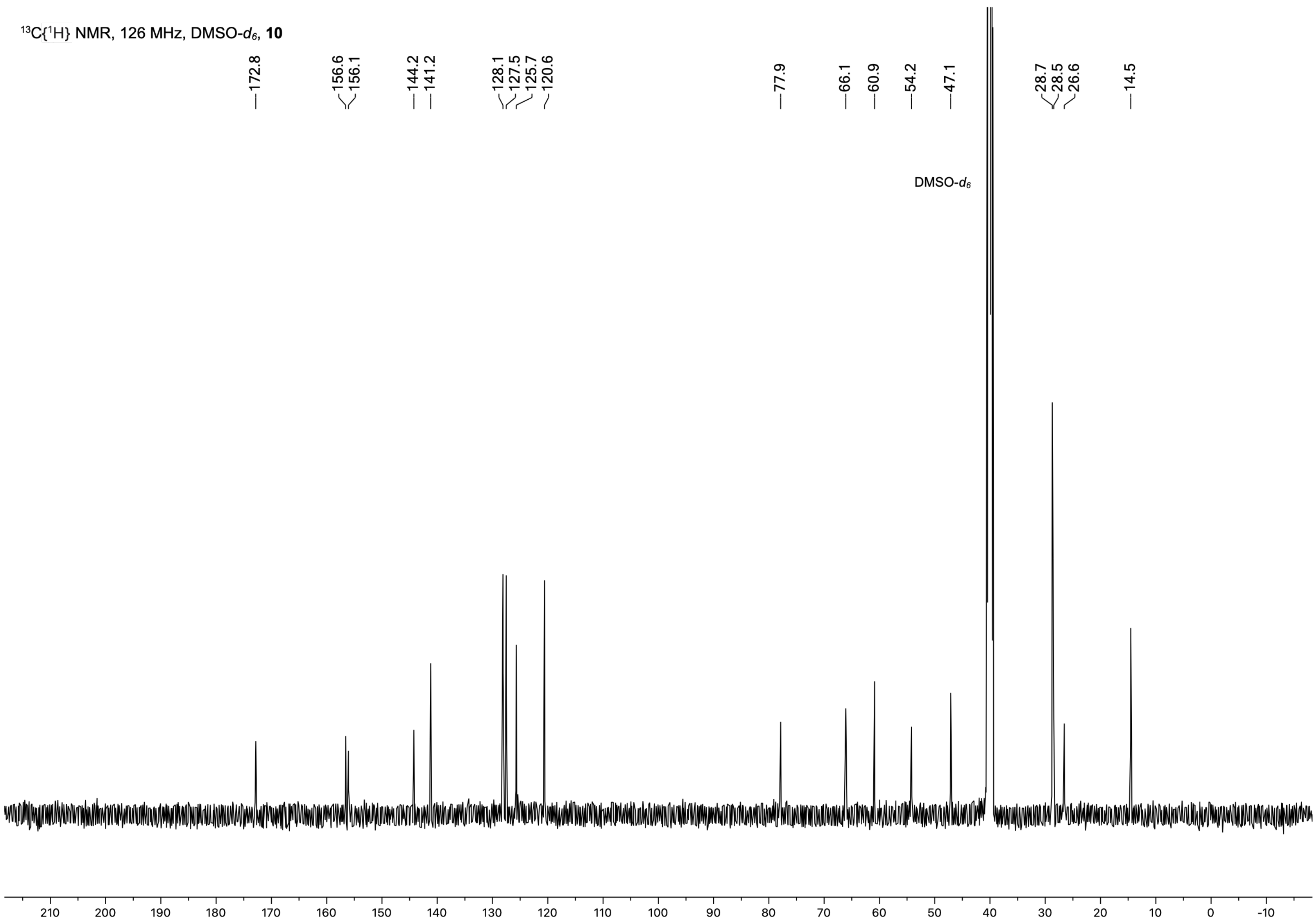

$^{13}C\{^1H\}$ NMR, 126 MHz, DMSO-d_6, **10**
172.8
156.6
156.1
144.2
141.2
128.1
127.5
125.7
120.6
77.9
66.1
60.9
54.2
47.1
28.7
28.5
26.6
14.5
DMSO-d_6
210 200 190 180 170 160 150 140 130 120 110 100 90 80 70 60 50 40 30 20 10 0 -10

^{1}H NMR, 500 MHz, DMSO-d_6, **12**

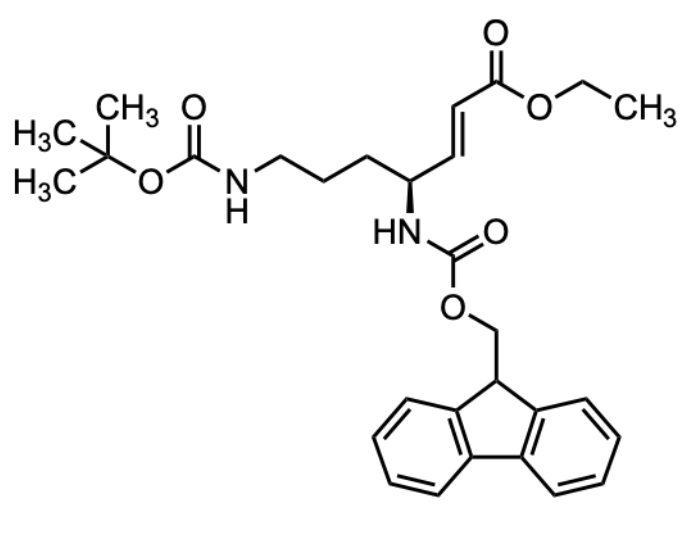

12

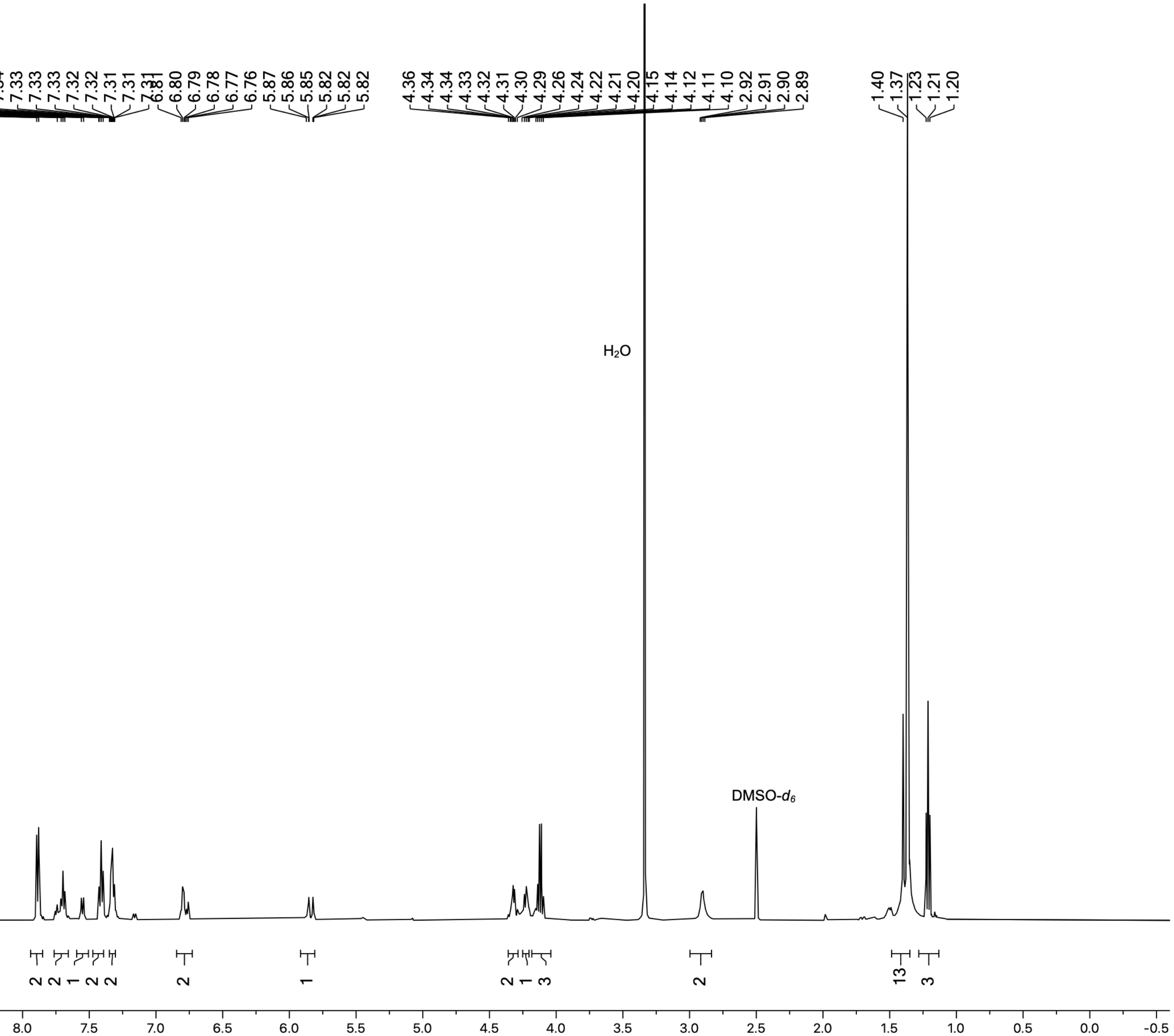

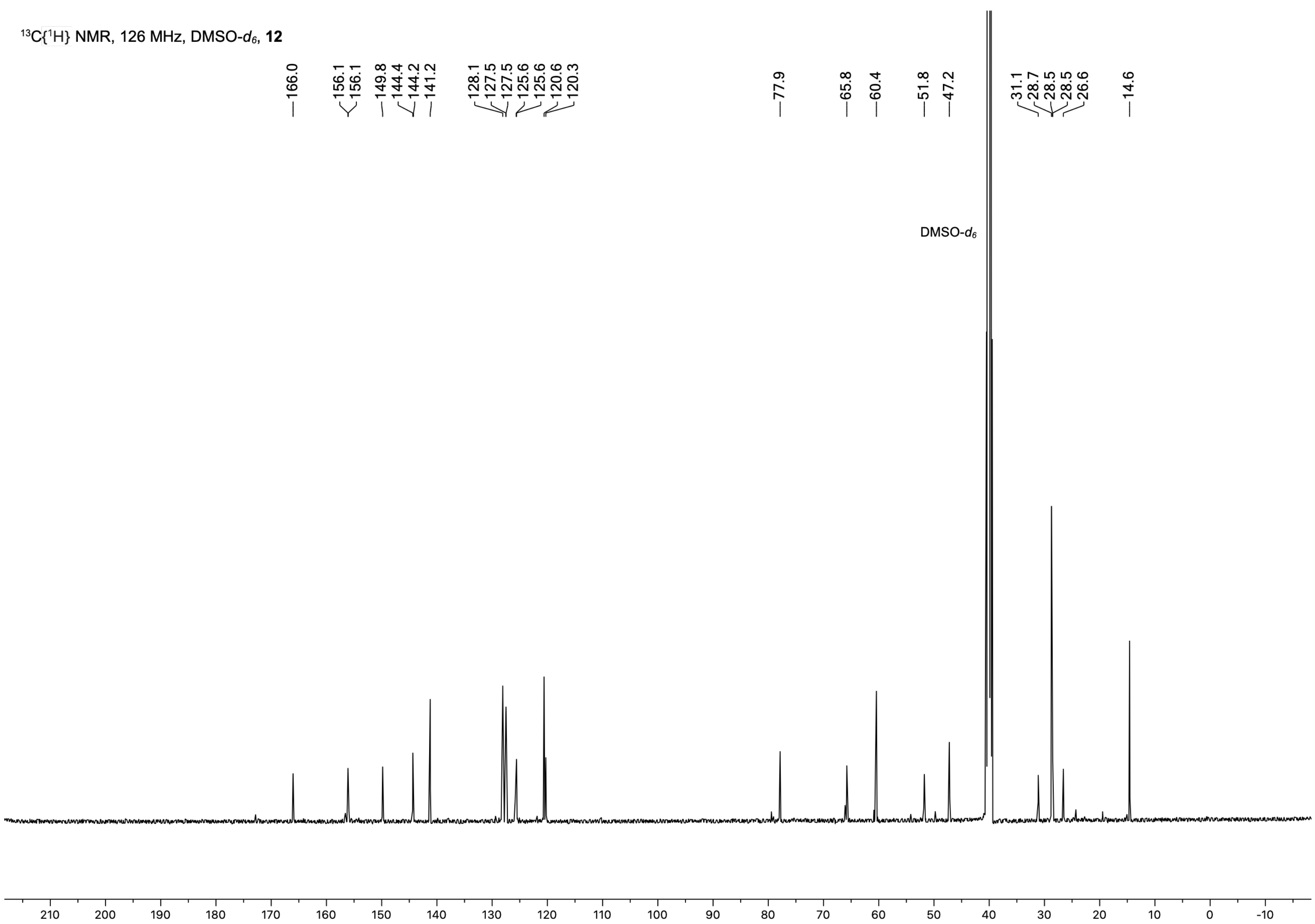

$^{13}C\{^1H\}$ NMR, 126 MHz, DMSO-d_6, **12**
166.0
156.1
156.1
149.8
144.4
144.2
141.2
128.1
127.5
127.5
125.6
125.6
120.6
120.3
77.9
65.8
60.4
51.8
47.2
31.1
28.7
28.5
28.5
26.6
14.6
DMSO-d_6
210 200 190 180 170 160 150 140 130 120 110 100 90 80 70 60 50 40 30 20 10 0 -10

^{1}H NMR, 500 MHz, DMSO-d_6, **S9**

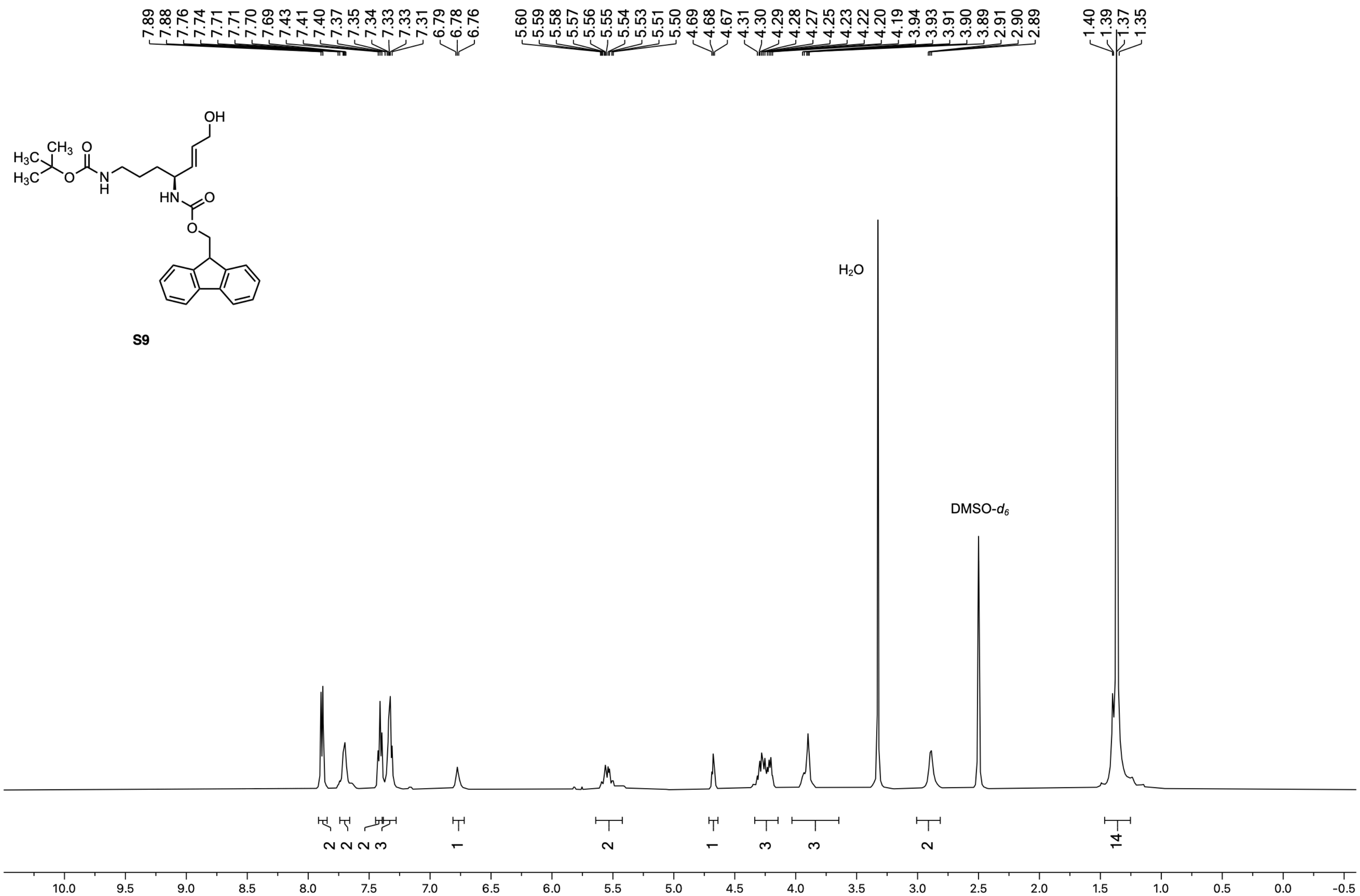

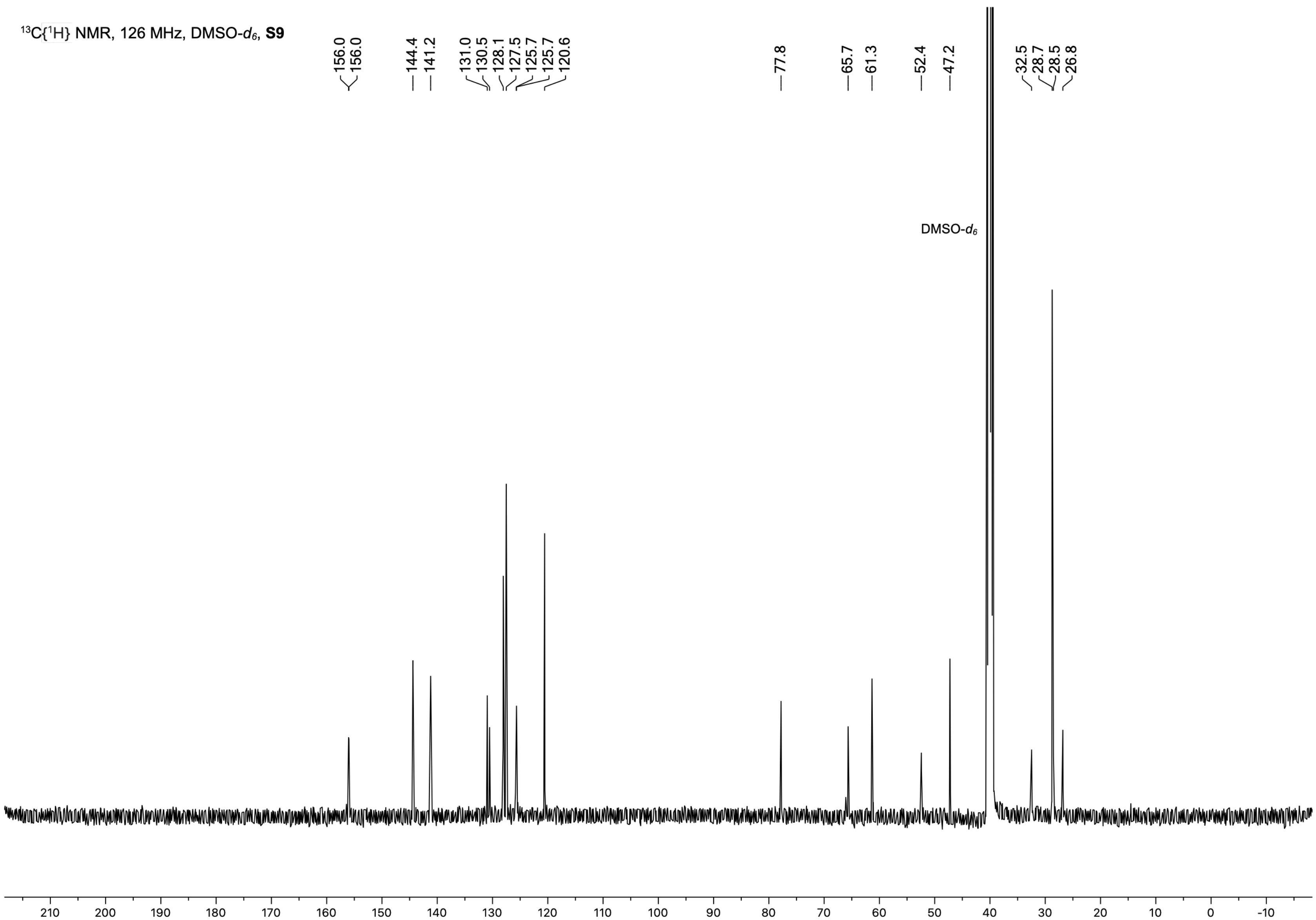

$^{13}C\{^{1}H\}$ NMR, 126 MHz, DMSO-d_6, **S9**
156.0
156.0
144.4
141.2
131.0
130.5
128.1
127.5
125.7
125.7
120.6
77.8
65.7
61.3
52.4
47.2
32.5
28.7
28.5
26.8
DMSO-d_6
210
200
190
180
170
160
150
140
130
120
110
100
90
80
70
60
50
40
30
20
10
0
-10

^1H NMR, 500 MHz, DMSO-d_6, **9**

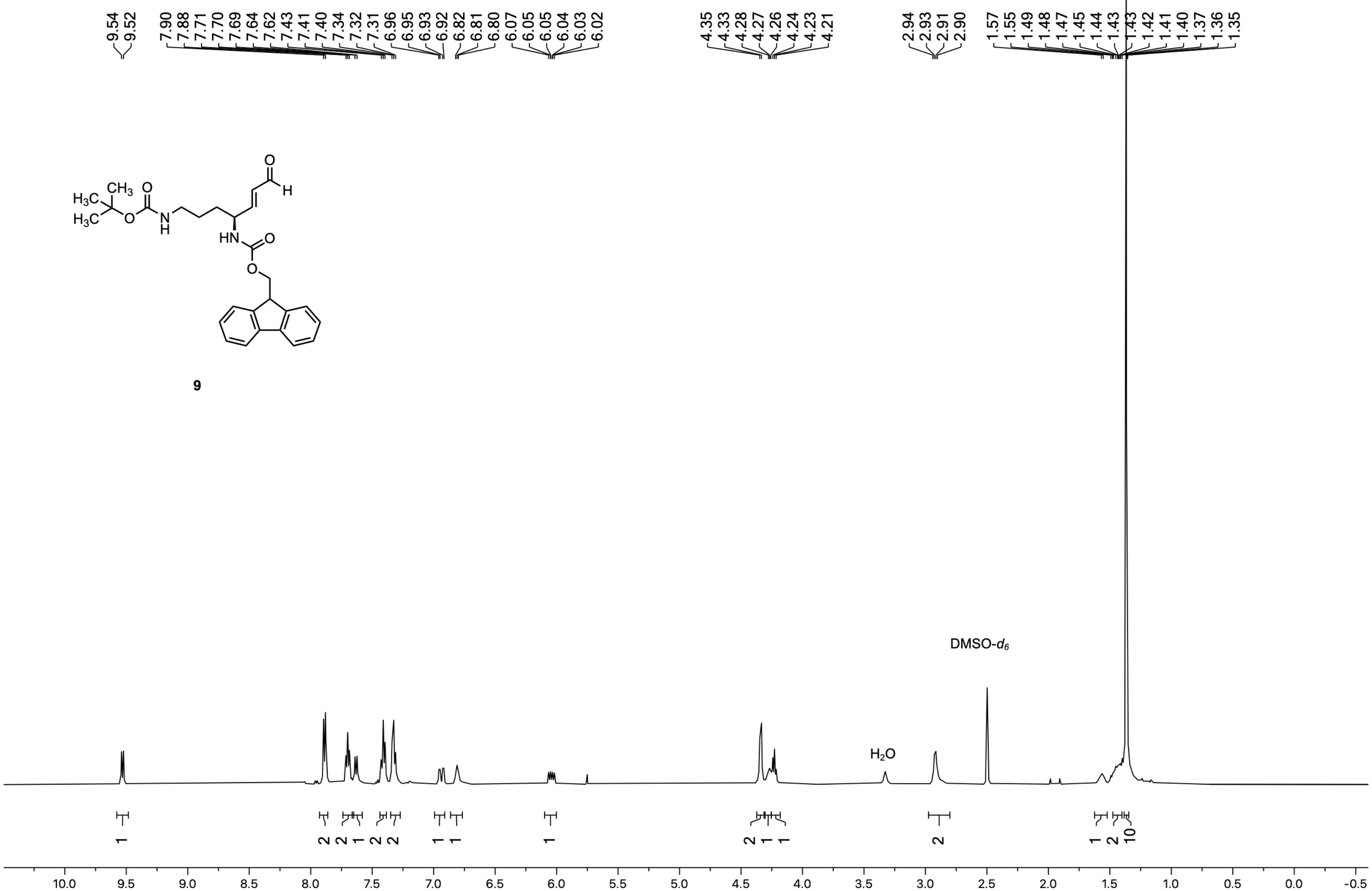

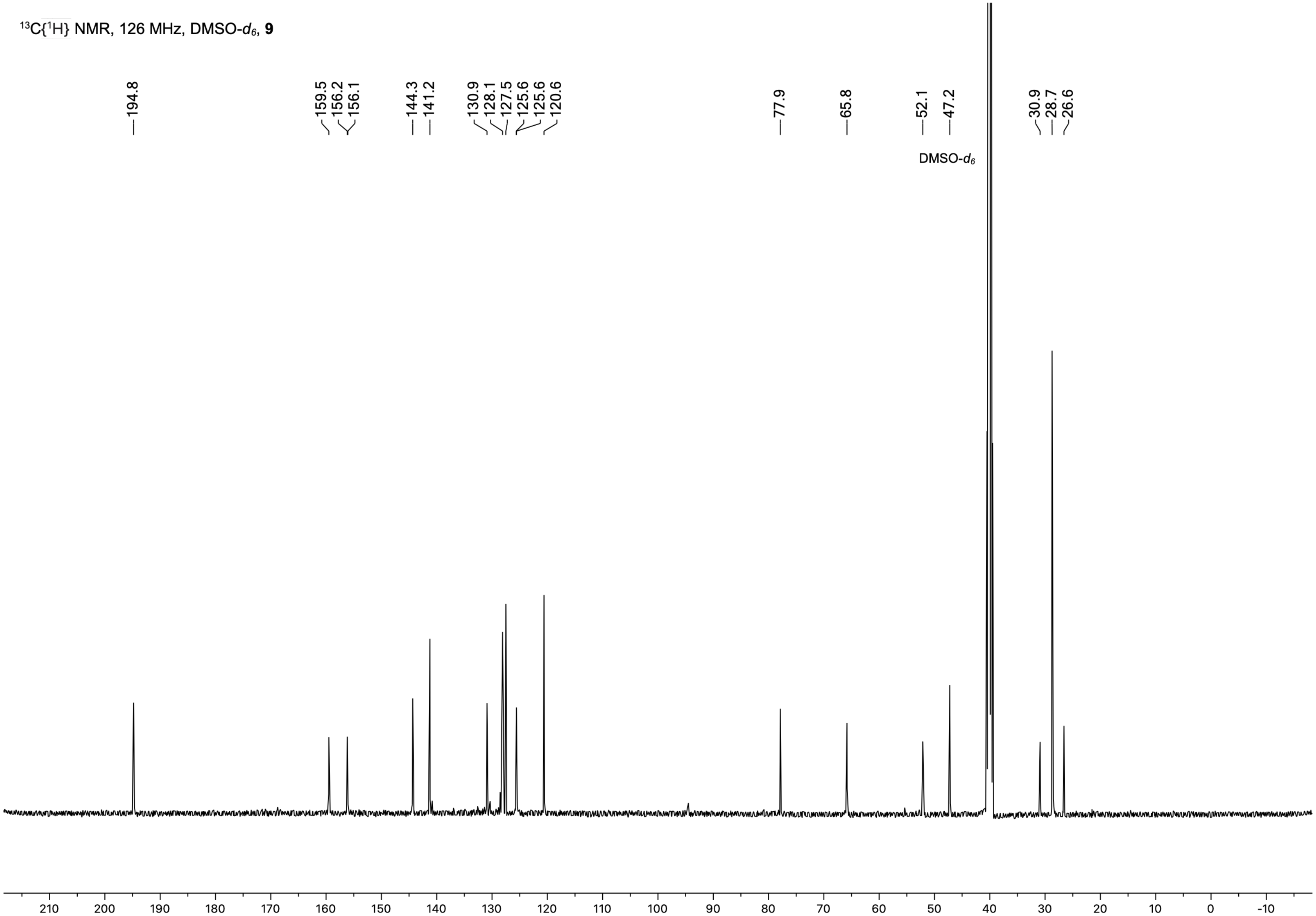

$^{13}C\{^1H\}$ NMR, 126 MHz, DMSO-d_6, **9**
194.8
159.5
156.2
156.1
144.3
141.2
130.9
128.1
127.5
125.6
125.6
120.6
77.9
65.8
52.1
47.2
30.9
28.7
26.6
DMSO-d_6
210 200 190 180 170 160 150 140 130 120 110 100 90 80 70 60 50 40 30 20 10 0 -10

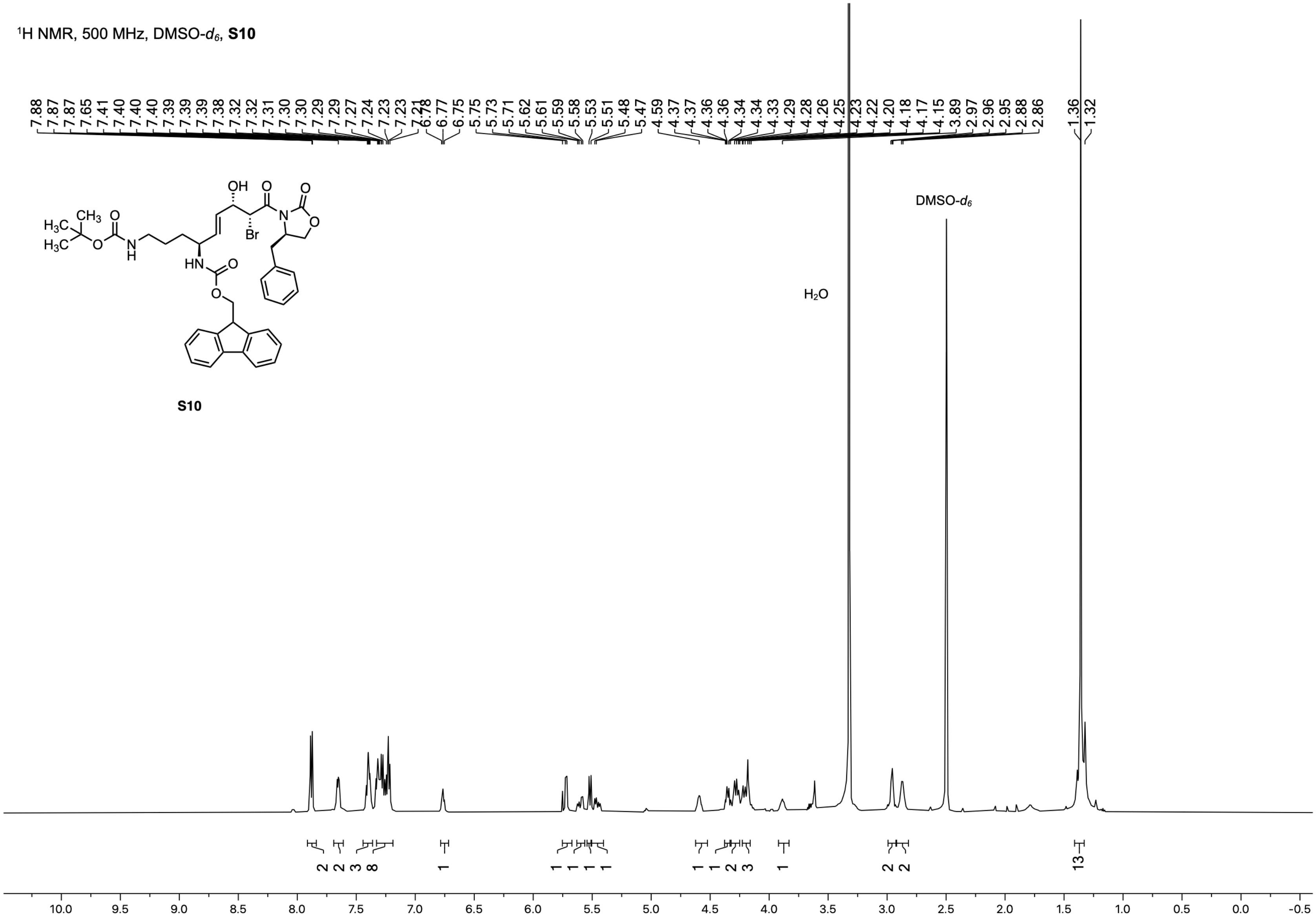

¹H NMR, 500 MHz, DMSO-d6, S10
S10
DMSO-d6
H2O

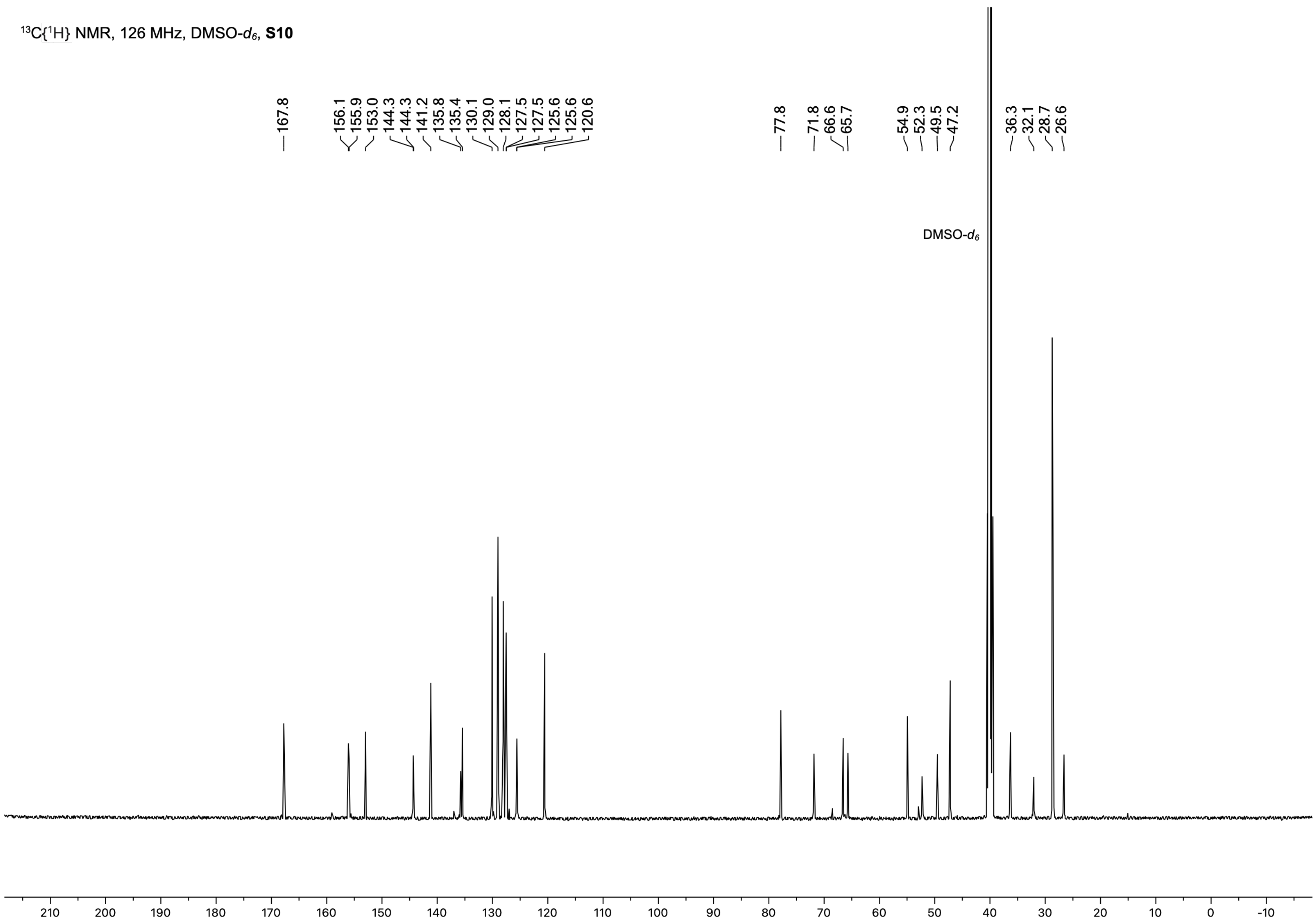
$^{13}C\{^1H\}$ NMR, 126 MHz, DMSO-d_6, **S10**
167.8
156.1
155.9
153.0
144.3
144.3
141.2
135.8
135.4
130.1
129.0
128.1
127.5
127.5
125.6
125.6
120.6
77.8
71.8
66.6
65.7
54.9
52.3
49.5
47.2
36.3
32.1
28.7
26.6
DMSO-d_6
210
200
190
180
170
160
150
140
130
120
110
100
90
80
70
60
50
40
30
20
10
0
-10

^{1}H NMR, 500 MHz, DMSO-d_6, **13**

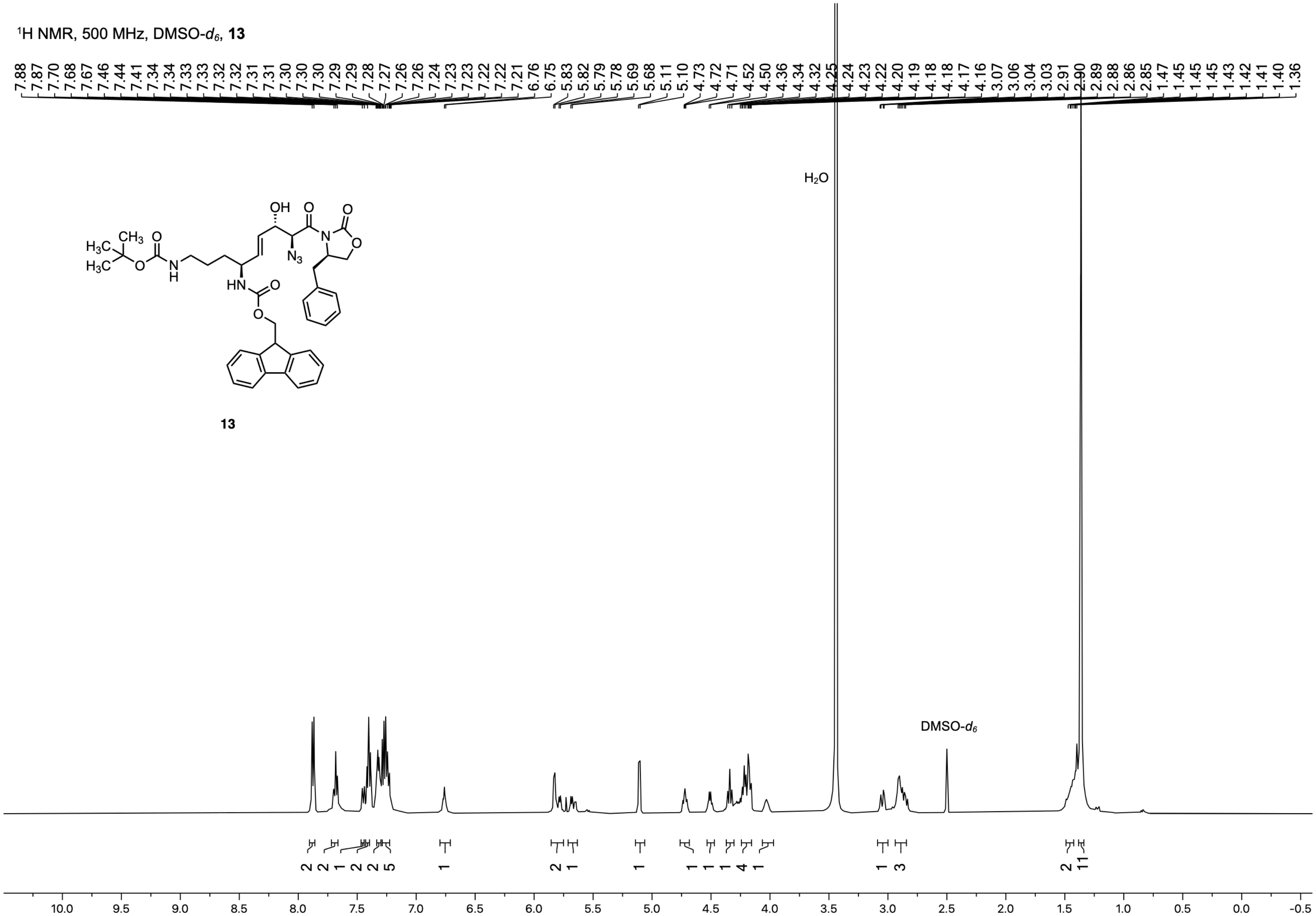

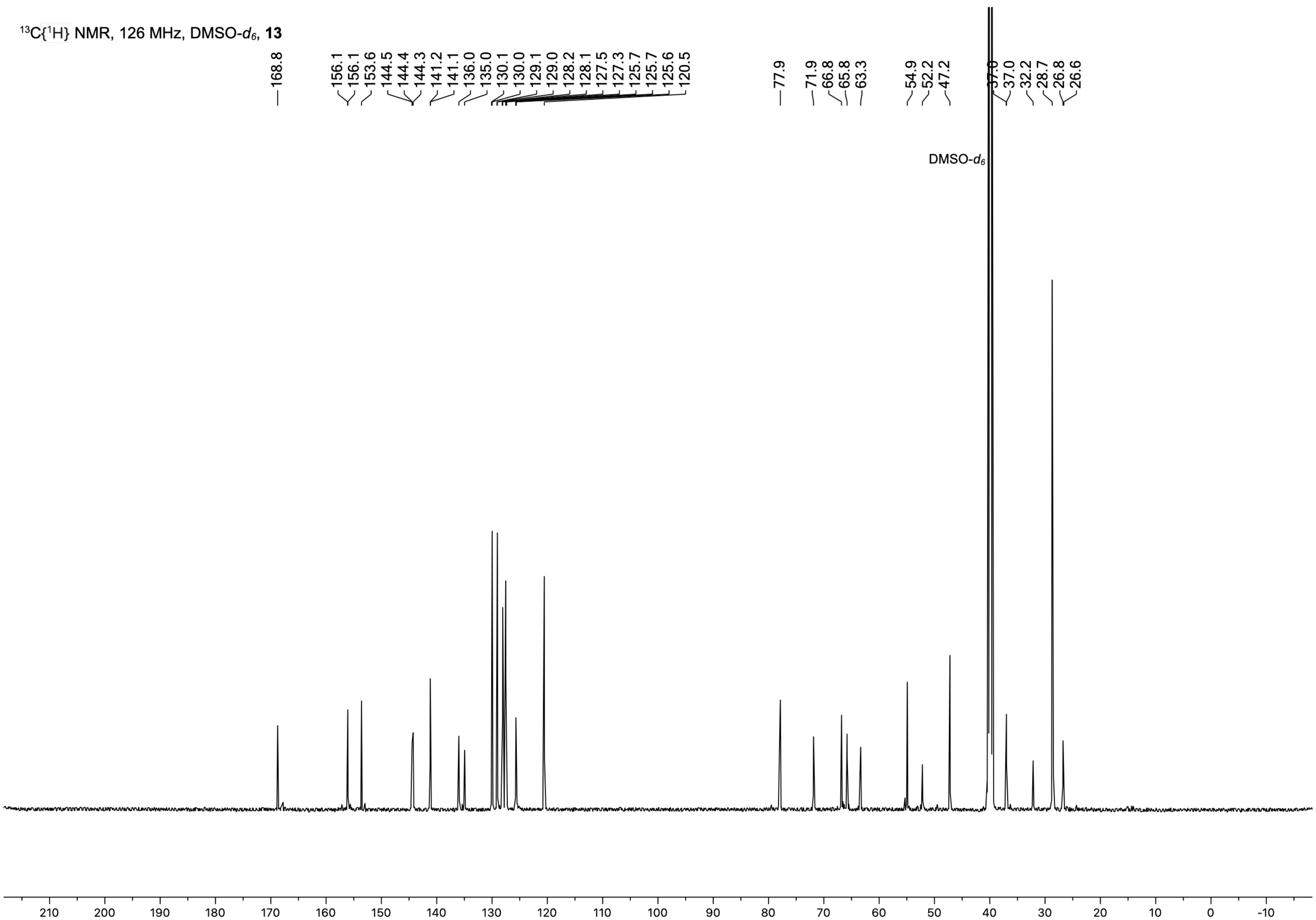

$^{13}C\{^1H\}$ NMR, 126 MHz, DMSO-d_6, **13**
168.8
156.1
156.1
153.6
144.5
144.4
144.3
141.2
141.1
136.0
135.0
130.1
130.0
129.1
129.0
128.2
128.1
127.5
127.3
125.7
125.7
125.6
120.5
77.9
71.9
66.8
65.8
63.3
54.9
52.2
47.2
37.0
37.0
32.2
28.7
26.8
26.6
DMSO-d_6
210
200
190
180
170
160
150
140
130
120
110
100
90
80
70
60
50
40
30
20
10
0
-10

^{1}H NMR, 500 MHz, DMSO-d_6, **14**

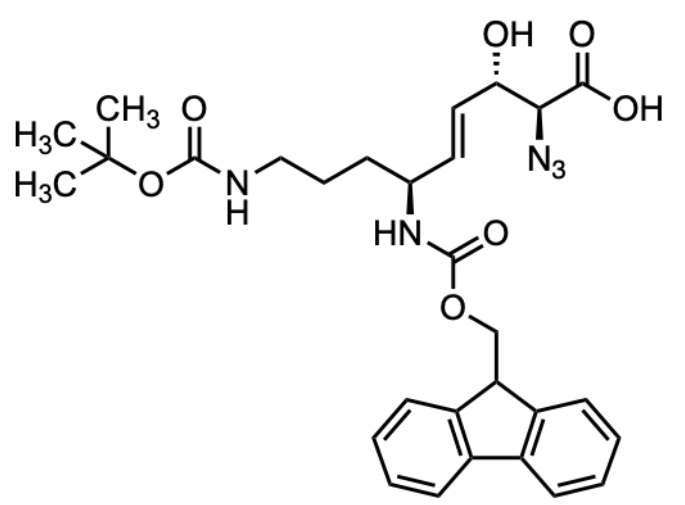

14

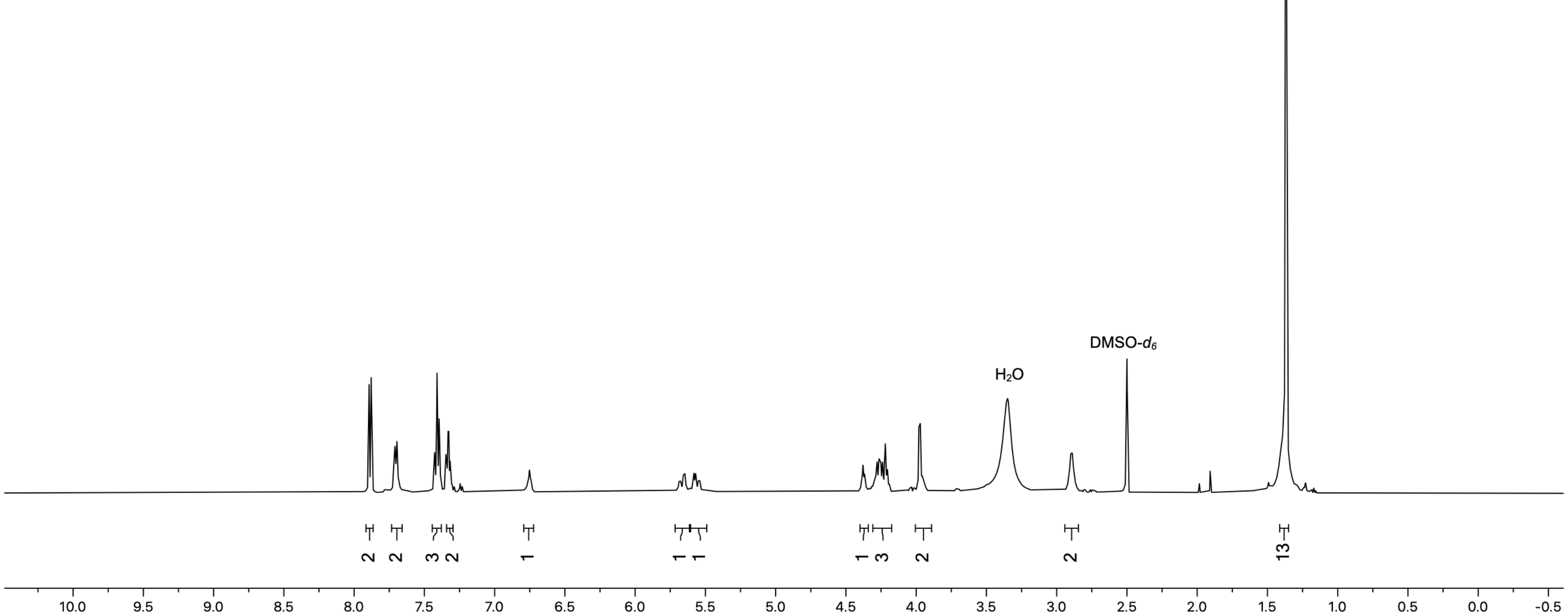

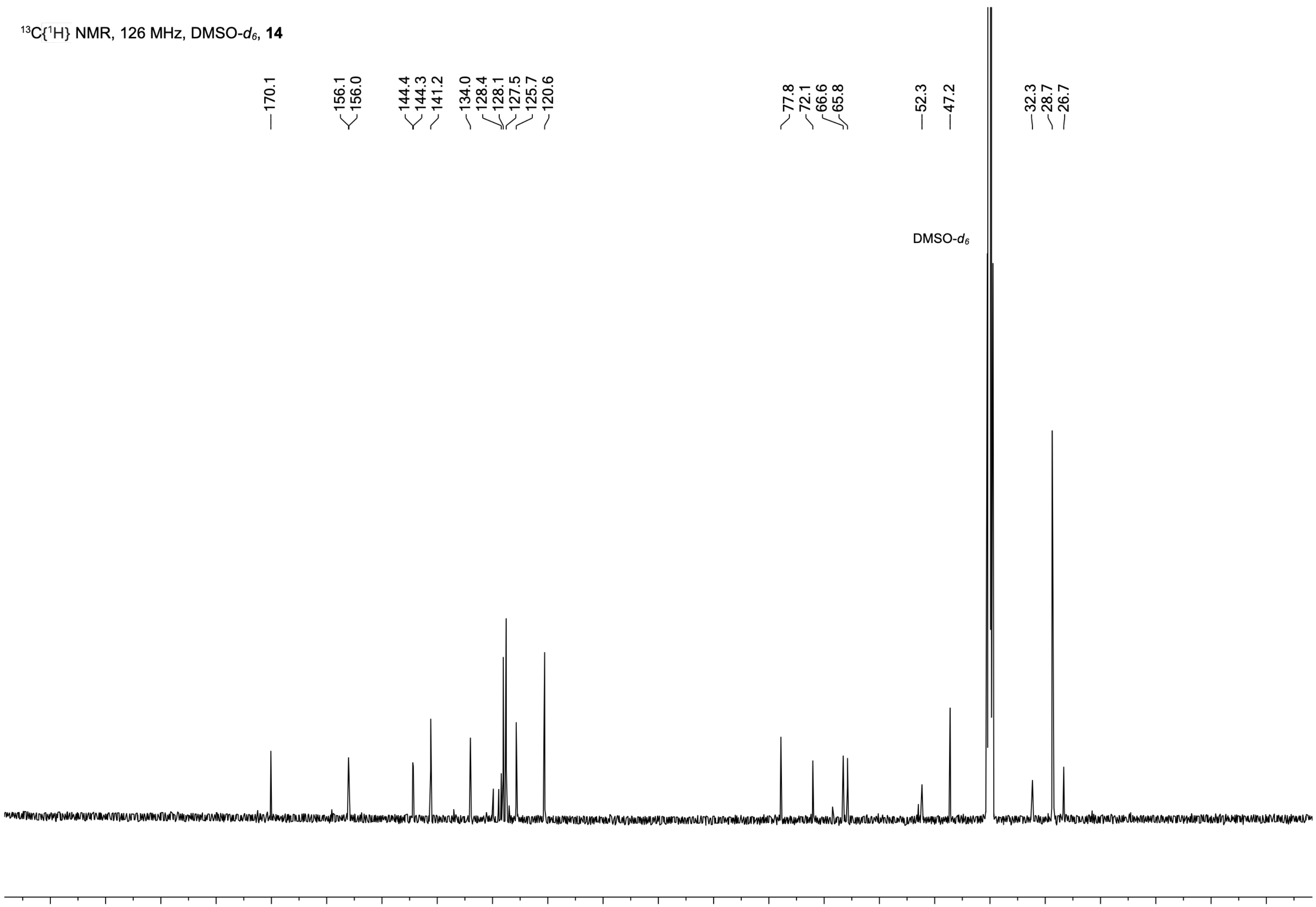

$^{13}C\{^1H\}$ NMR, 126 MHz, DMSO-d_6, **14**
170.1
156.1
156.0
144.4
144.3
141.2
134.0
128.4
128.1
127.5
125.7
120.6
77.8
72.1
66.6
65.8
52.3
47.2
32.3
28.7
26.7
DMSO-d_6
210
200
190
180
170
160
150
140
130
120
110
100
90
80
70
60
50
40
30
20
10
0
-10

^{1}H NMR, 500 MHz, DMSO-d_6, **15**

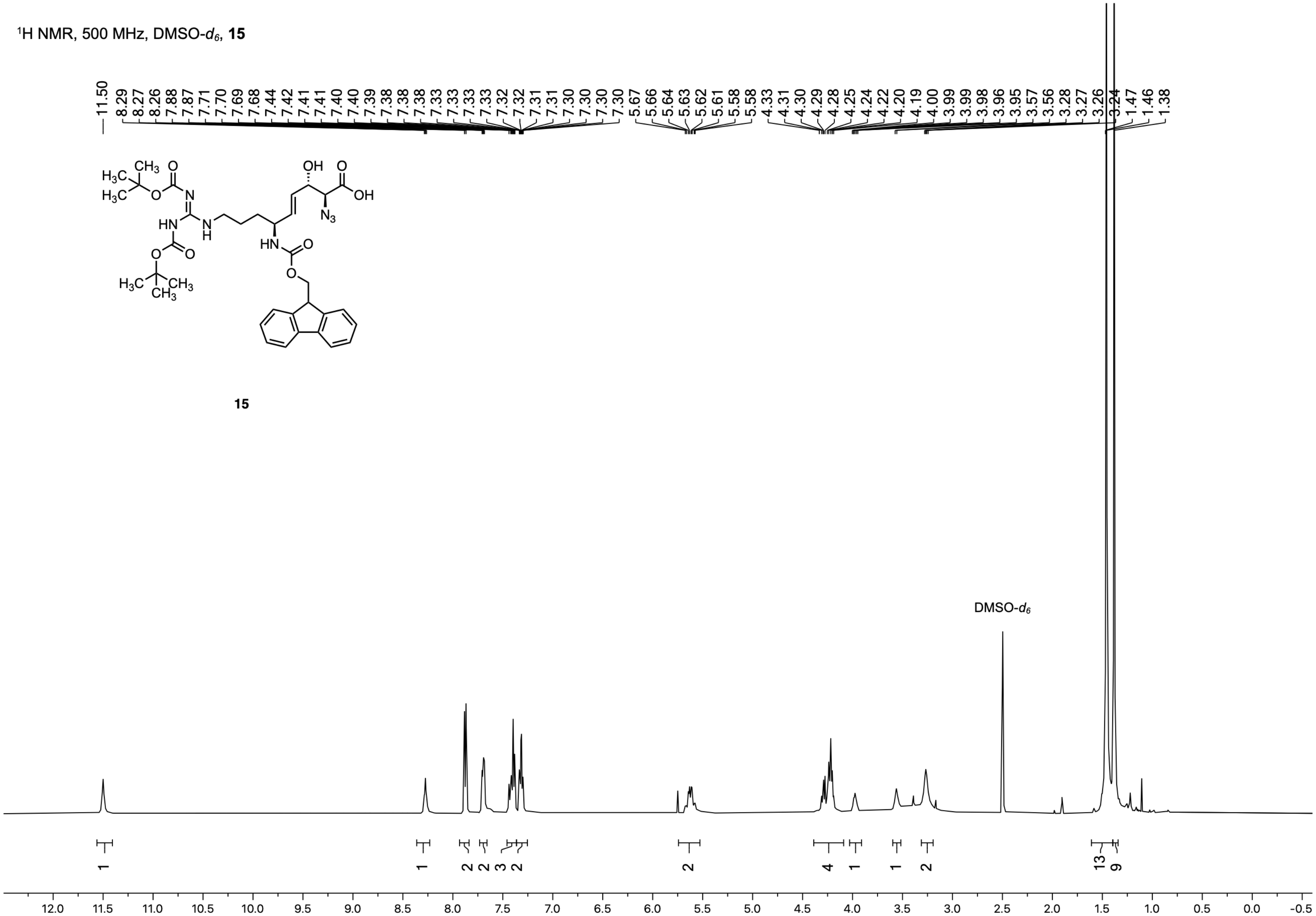

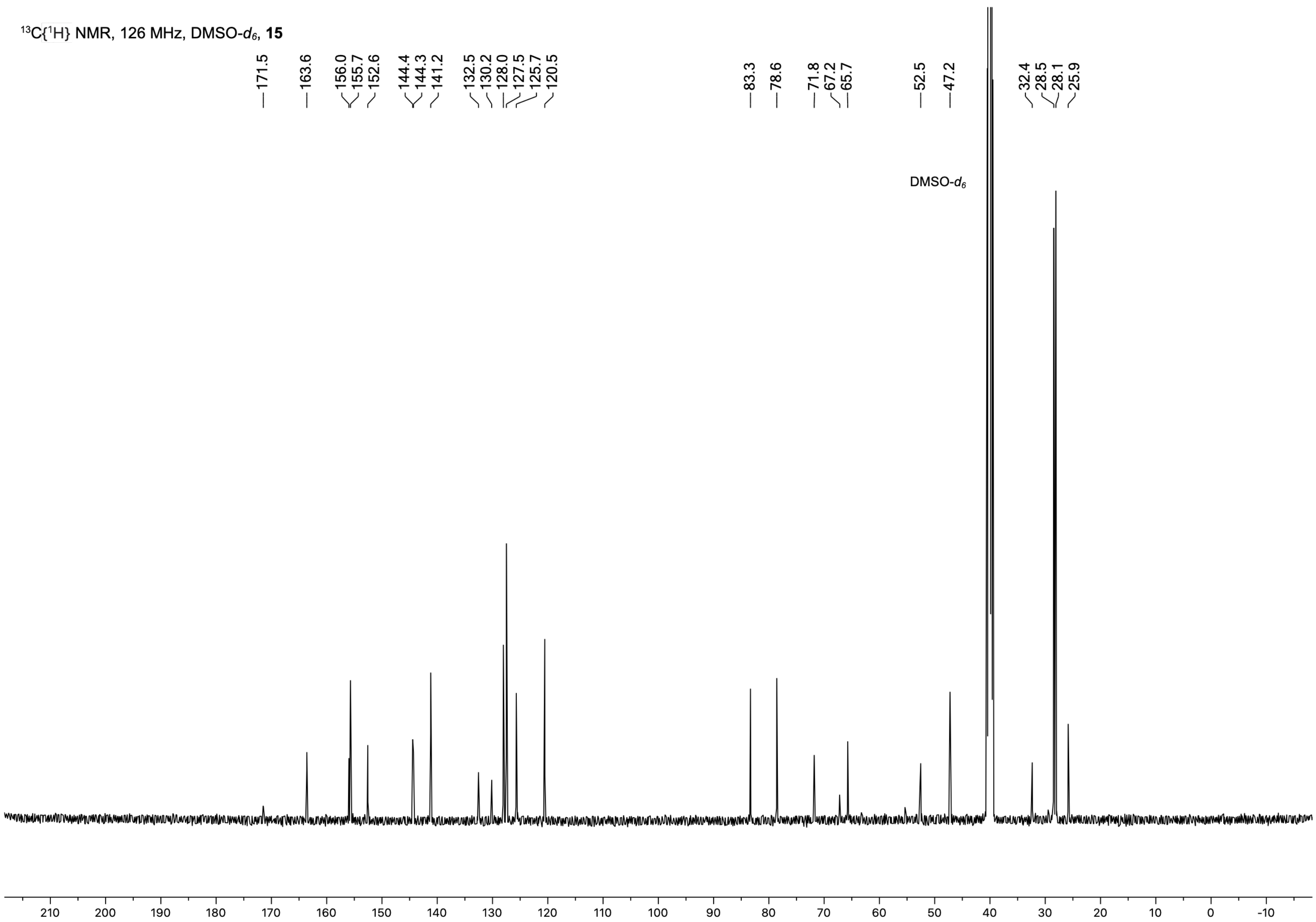

$^{13}C\{^1H\}$ NMR, 126 MHz, DMSO-d_6, **15**
171.5
163.6
156.0
155.7
152.6
144.4
144.3
141.2
132.5
130.2
128.0
127.5
125.7
120.5
83.3
78.6
71.8
67.2
65.7
52.5
47.2
32.4
28.5
28.1
25.9
DMSO-d_6
210 200 190 180 170 160 150 140 130 120 110 100 90 80 70 60 50 40 30 20 10 0 -10

^{1}H NMR, 500 MHz, DMSO-d_6, **16**

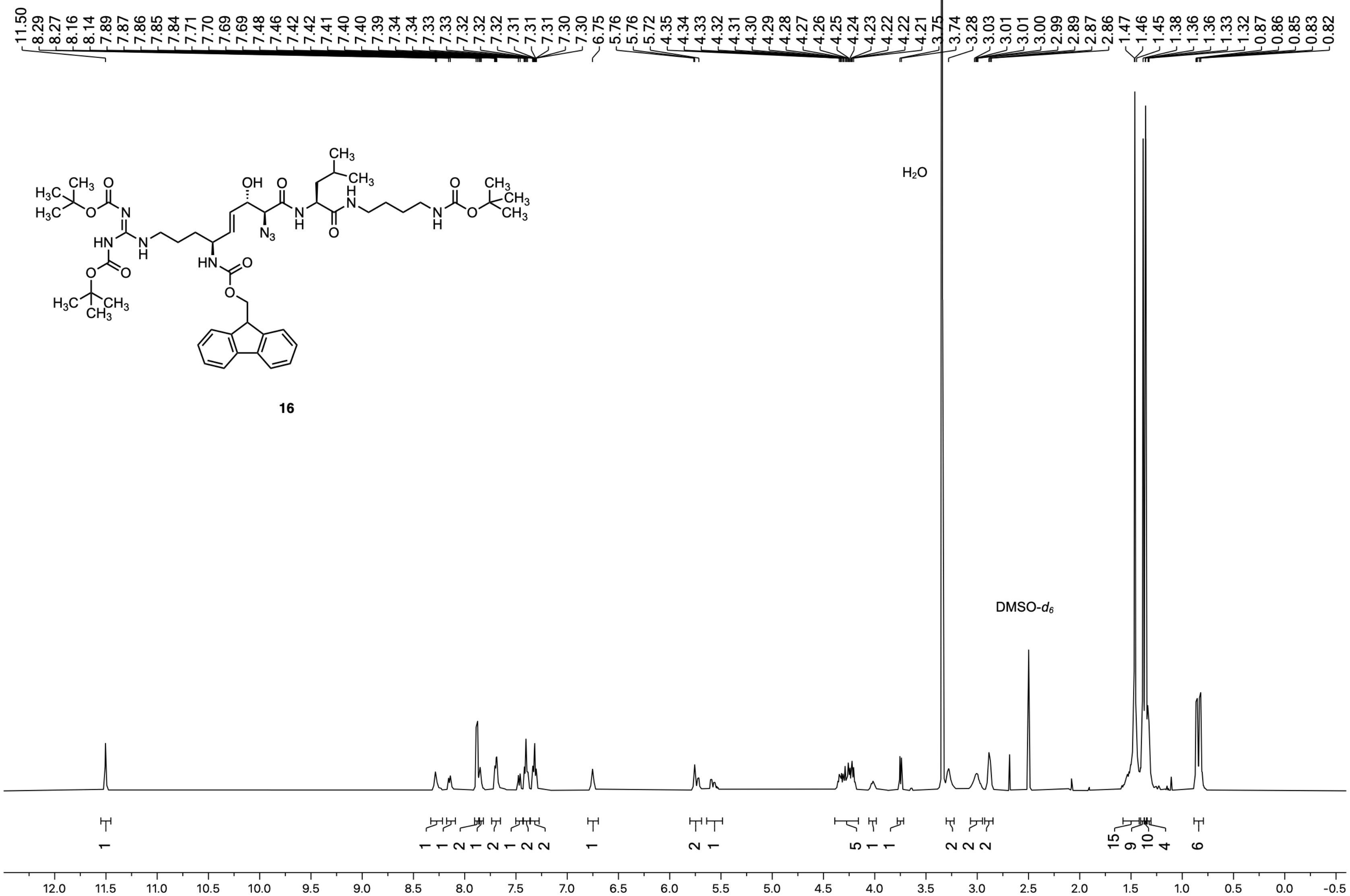

COSY, DMSO-d_6, **16**

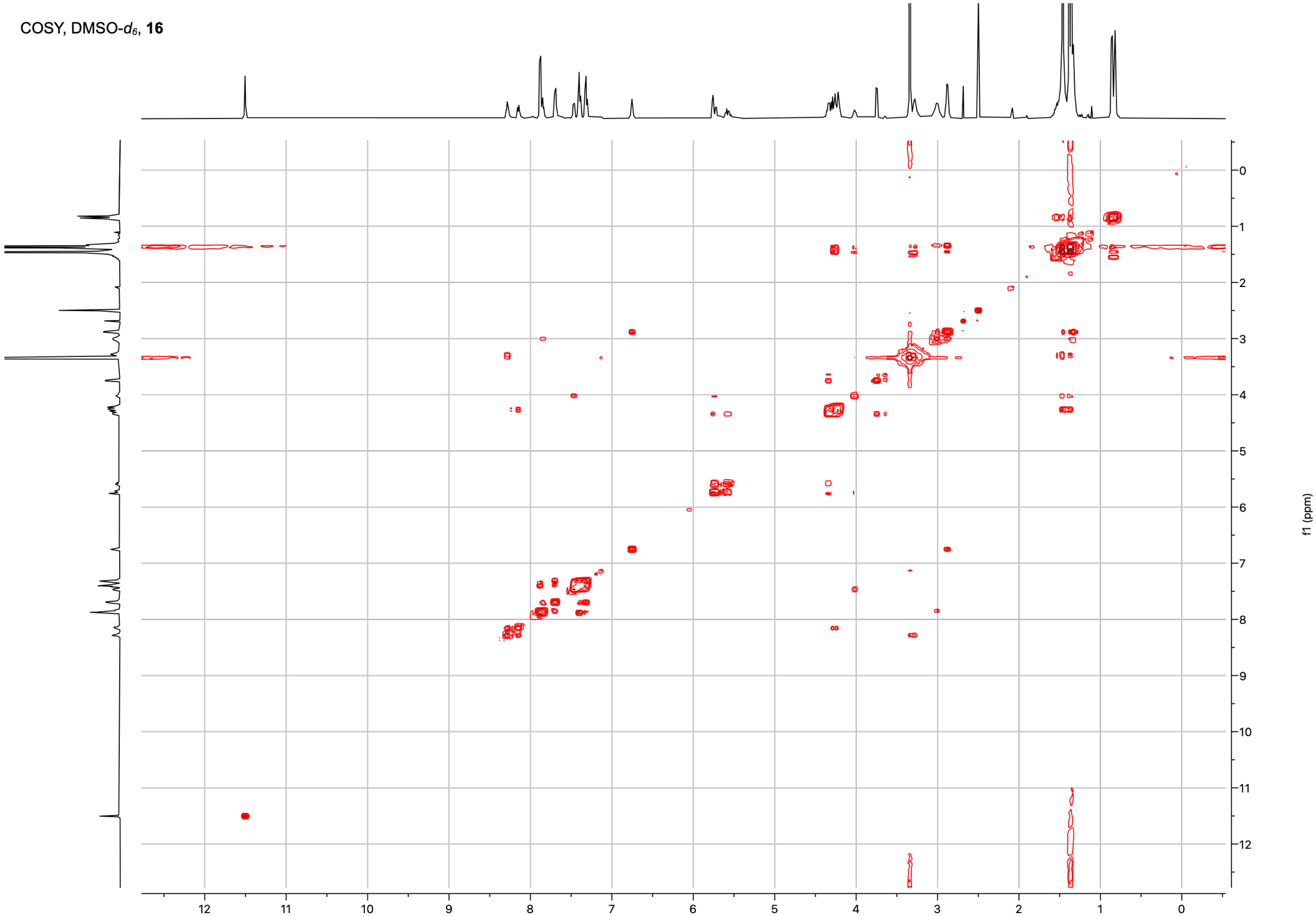

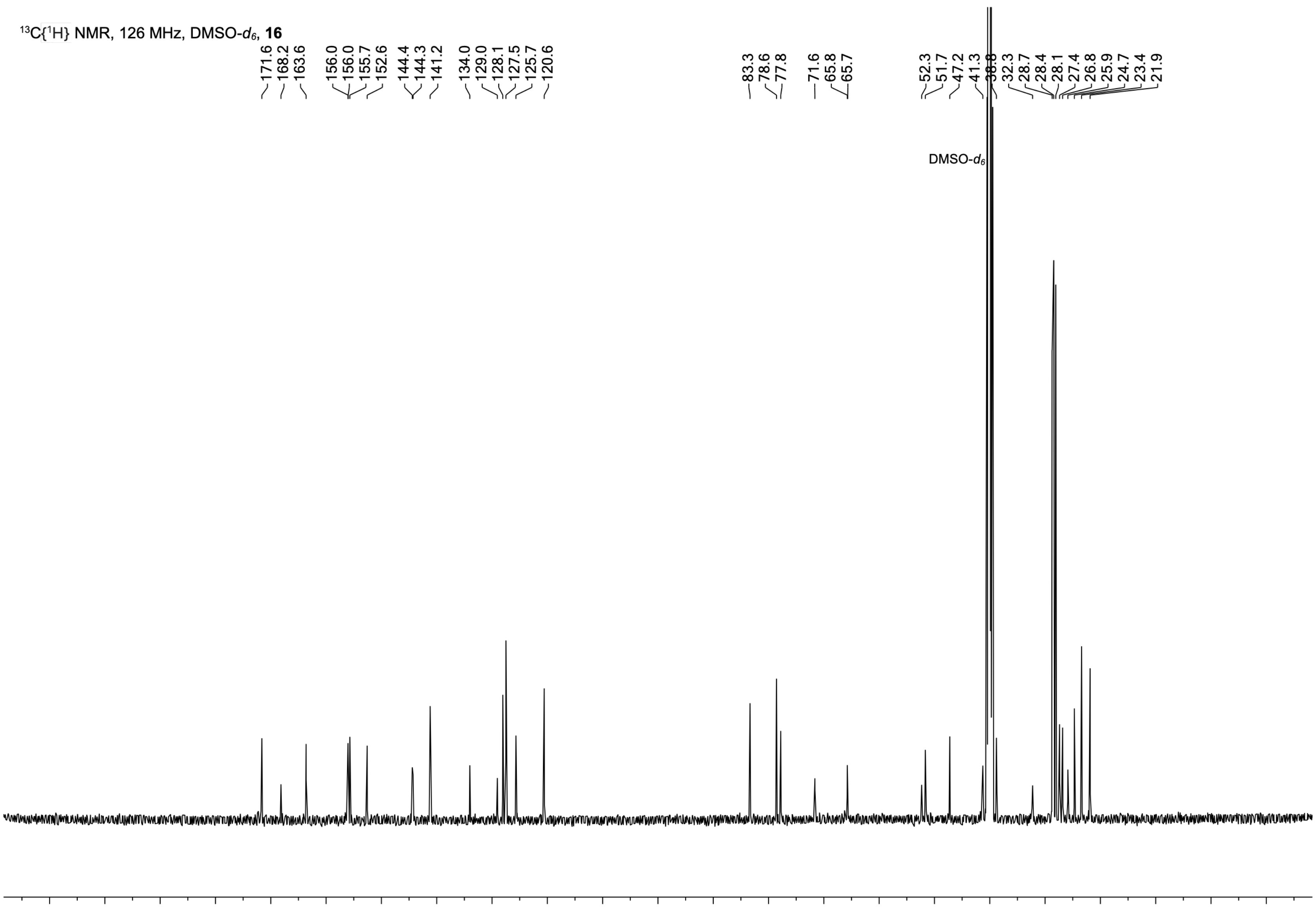

$^{13}C\{^1H\}$ NMR, 126 MHz, DMSO-d_6, **16**
171.6
168.2
163.6
156.0
156.0
155.7
152.6
144.4
144.3
141.2
134.0
129.0
128.1
127.5
125.7
120.6
83.3
78.6
77.8
71.6
65.8
65.7
52.3
51.7
47.2
41.3
38.8
32.3
28.7
28.4
28.1
27.4
26.8
25.9
24.7
23.4
21.9
DMSO-d_6
210
200
190
180
170
160
150
140
130
120
110
100
90
80
70
60
50
40
30
20
10
0
-10

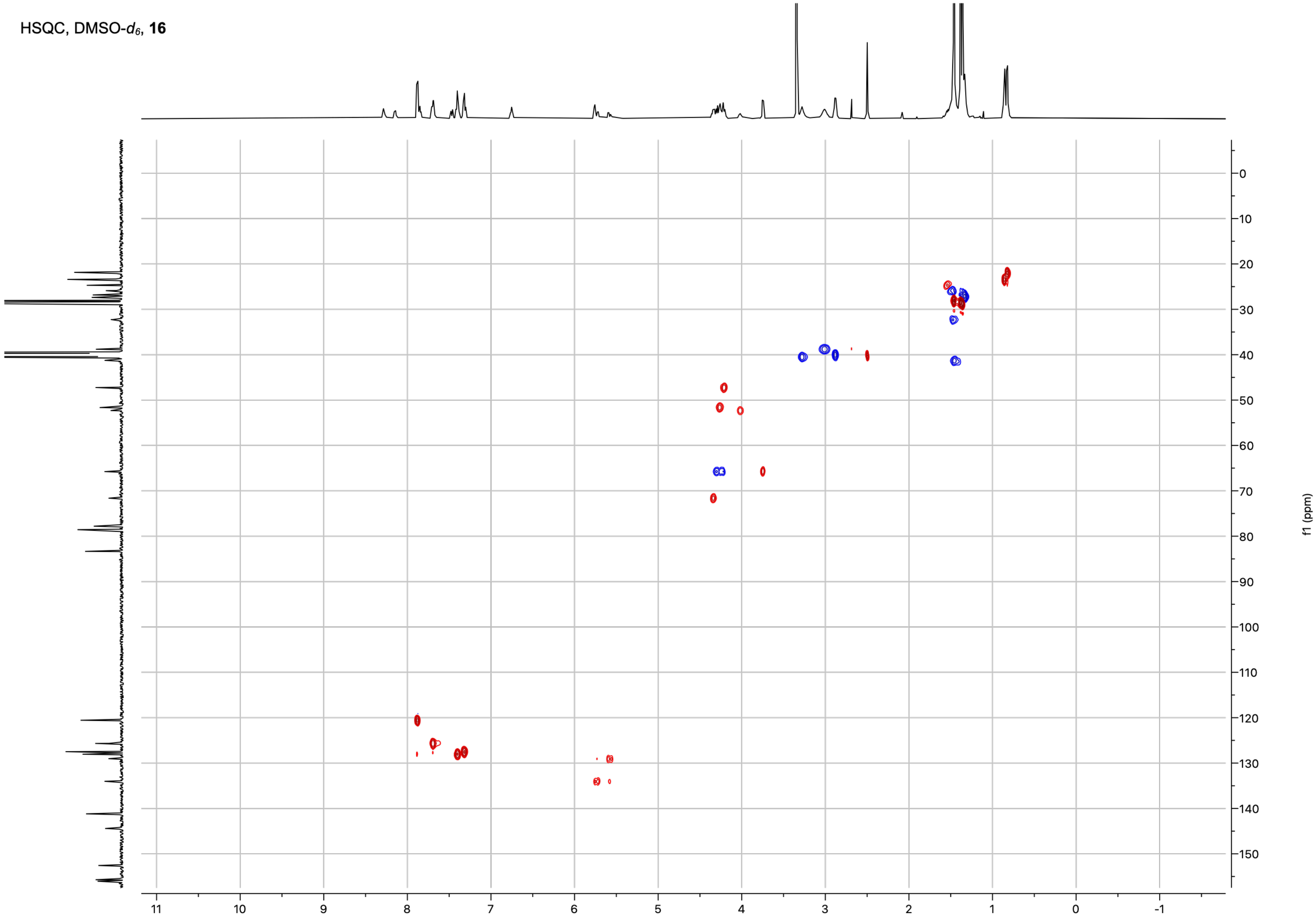
HSQC, DMSO-d_6, **16**
f1 (ppm)
11
10
9
8
7
6
5
4
3
2
1
0
-1
0
10
20
30
40
50
60
70
80
90
100
110
120
130
140
150

^{1}H NMR, 500 MHz, DMSO-d_6, **S11**

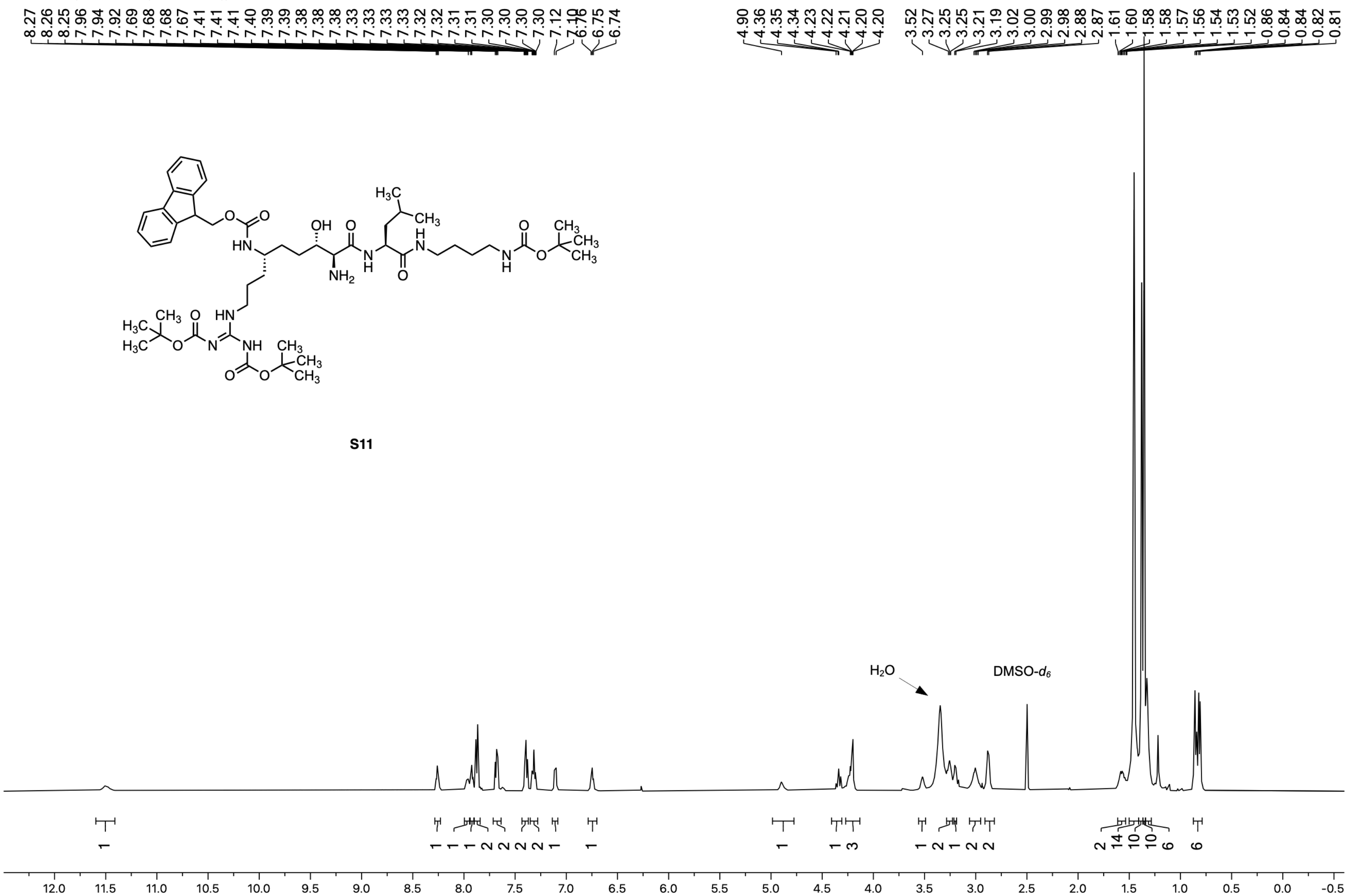

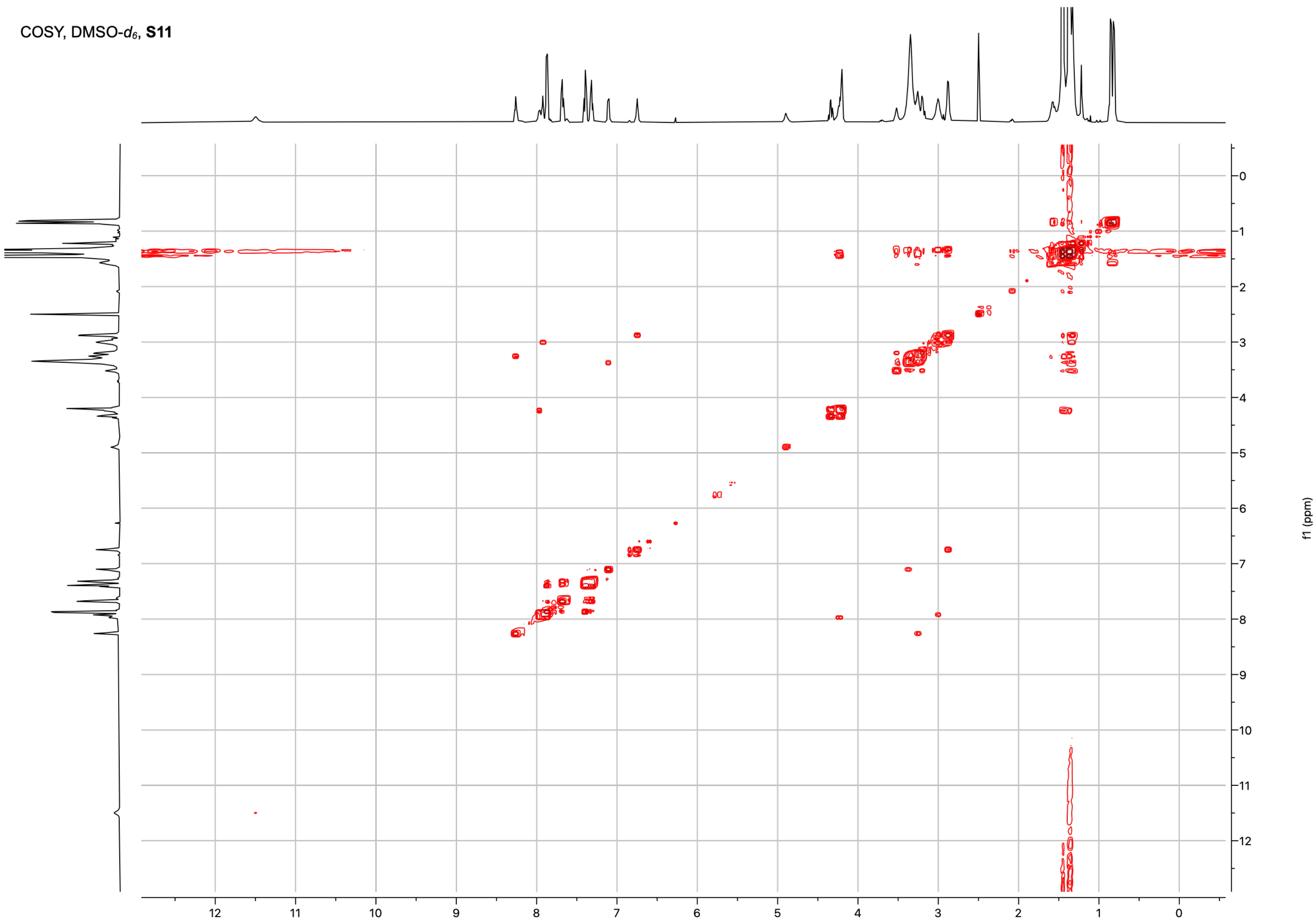

COSY, DMSO-d_6, **S11**
12
11
10
9
8
7
6
5
4
3
2
1
0
f1 (ppm)

13C{1H} NMR, 126 MHz, DMSO-d_6, **S11**

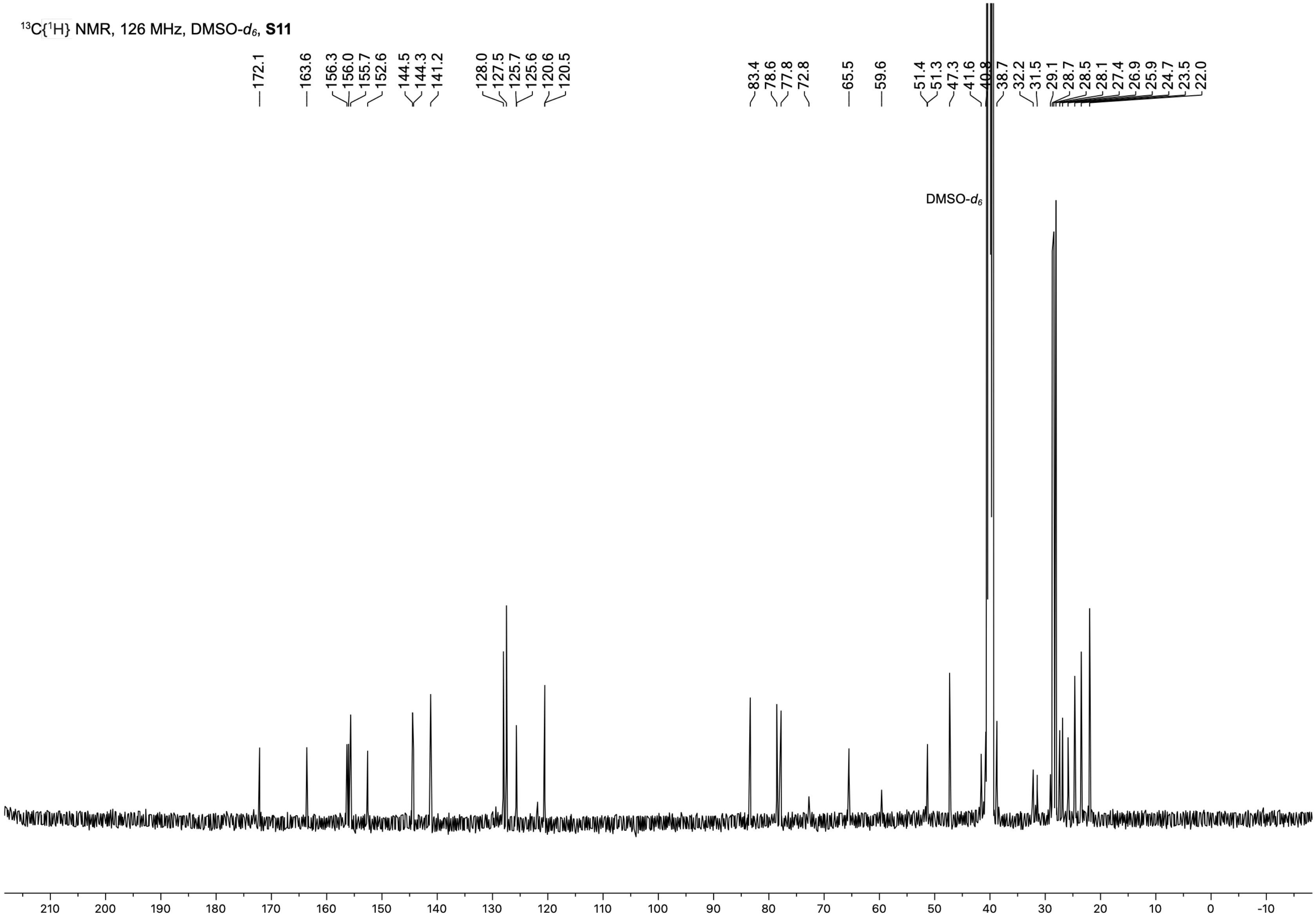

HSQC, DMSO-d_6, **S11**

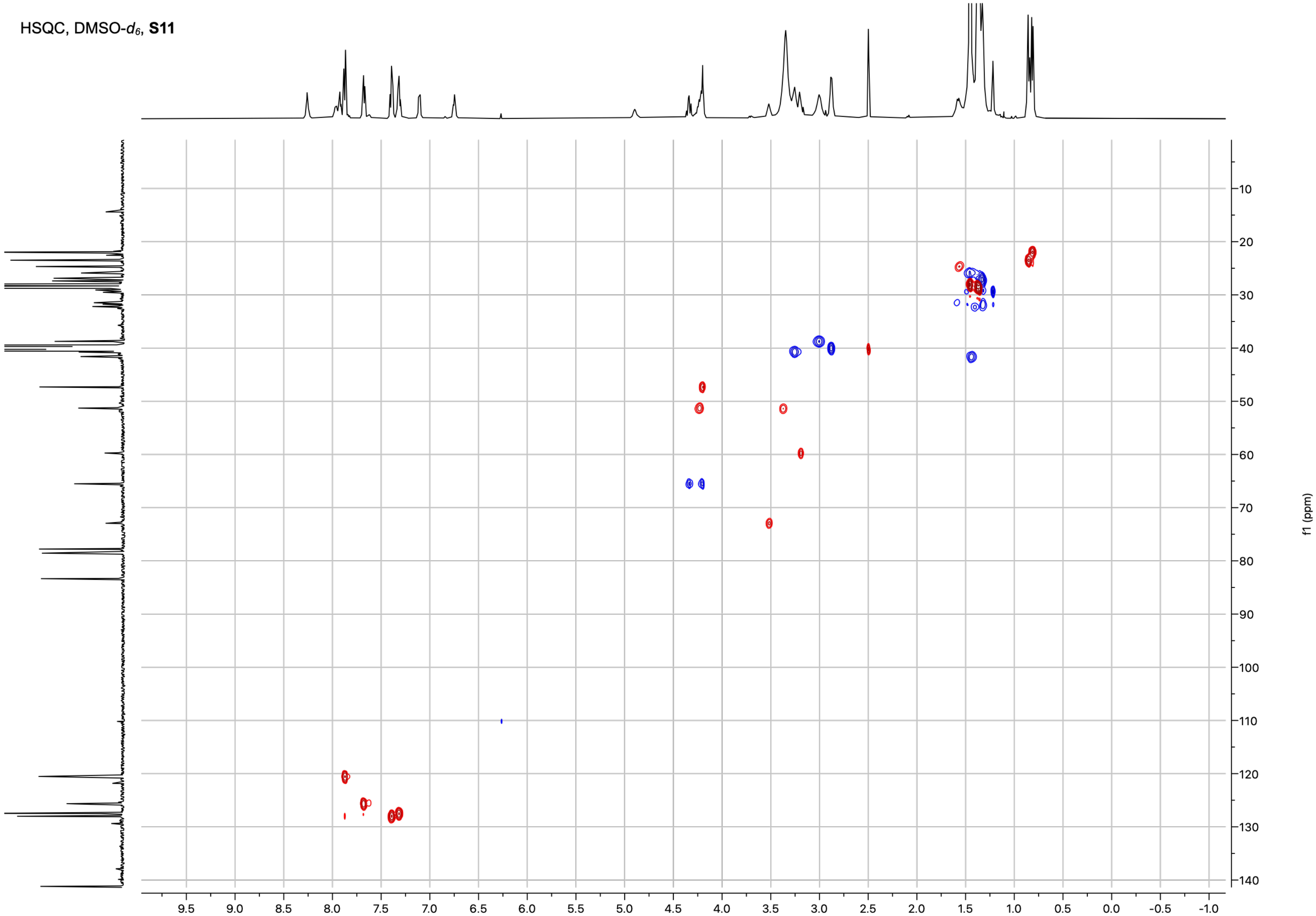

^{1}H NMR, 500 MHz, DMF-d_7, **4**

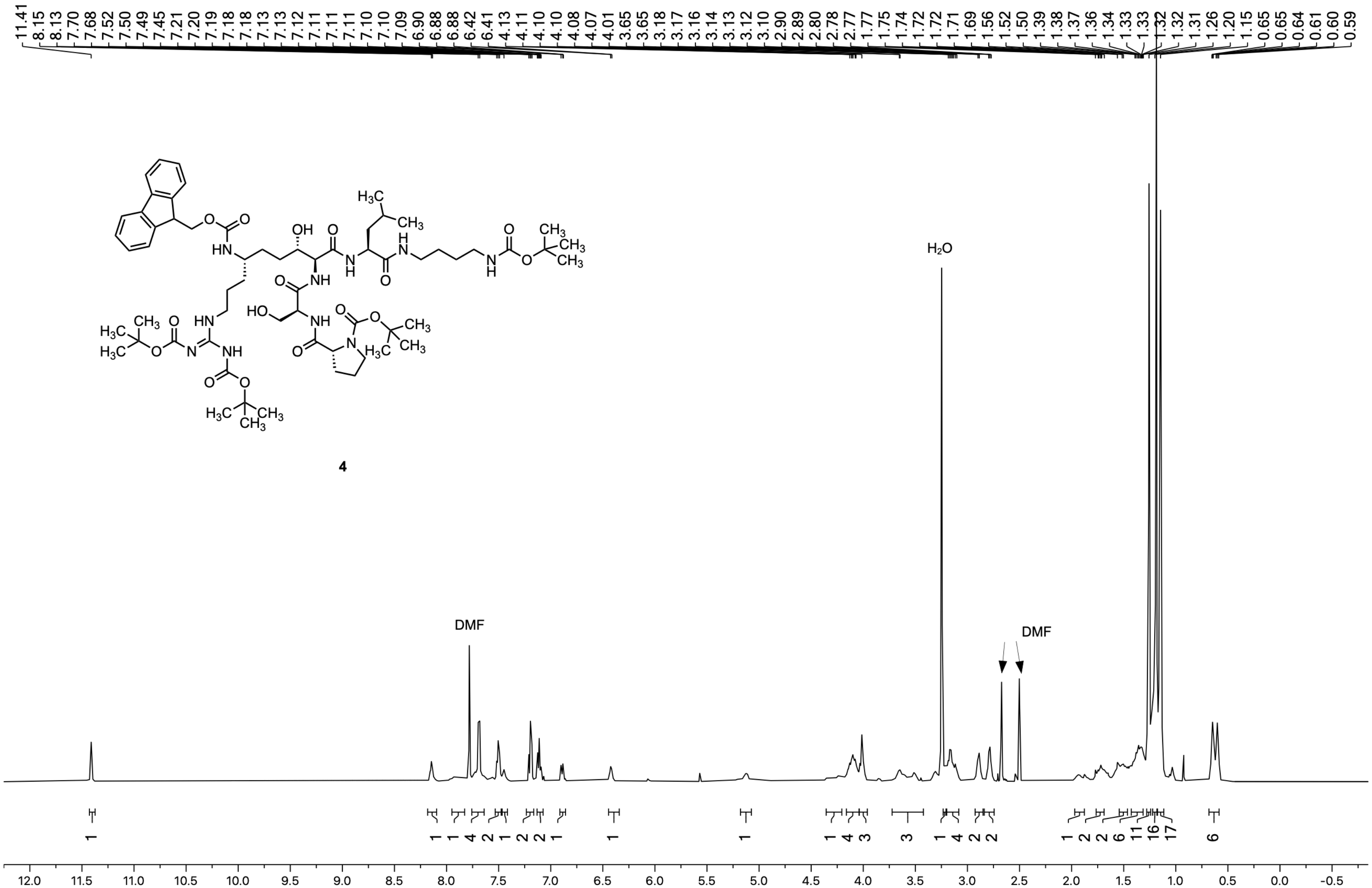

4

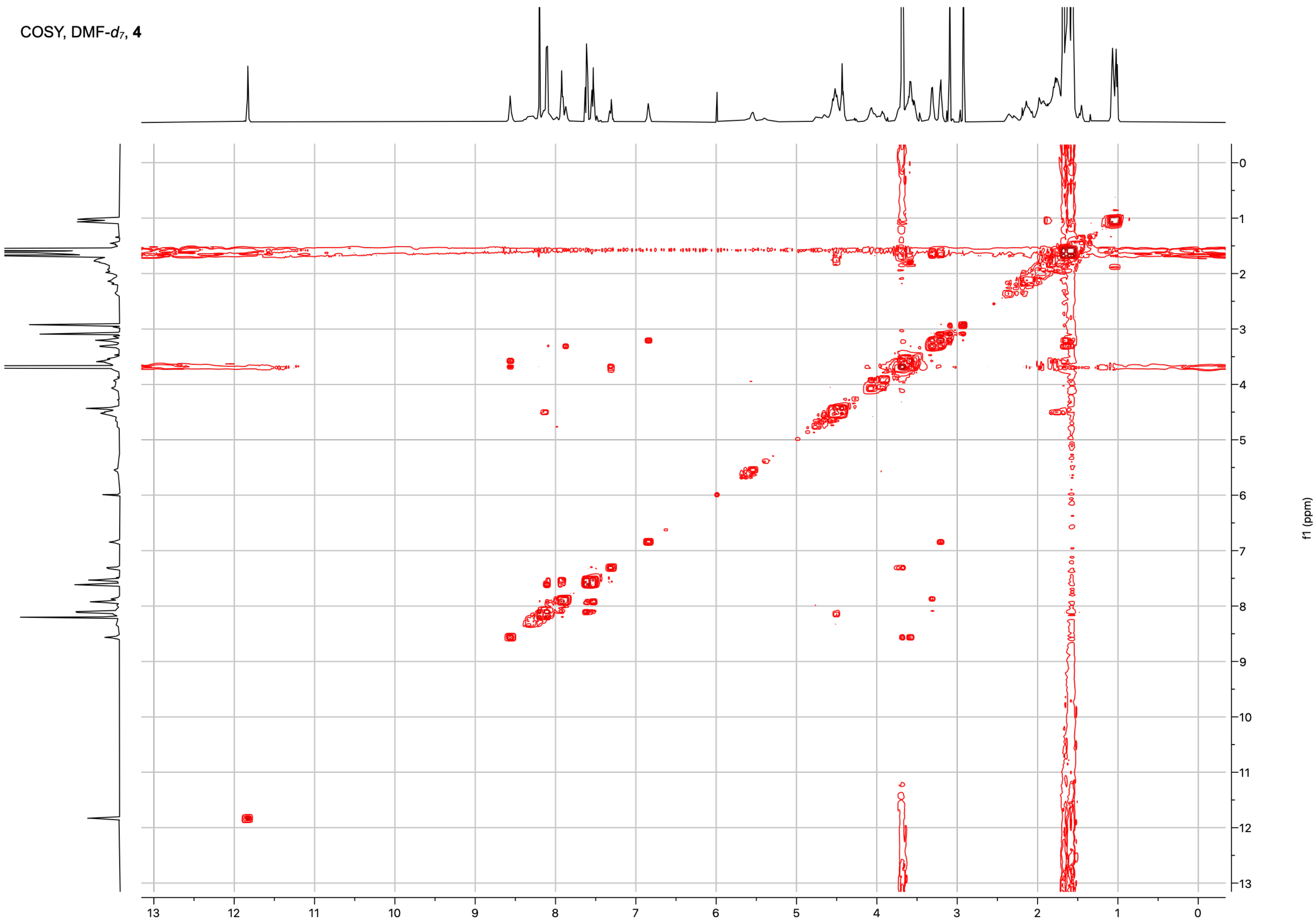
COSY, DMF-d_7, **4**
f1 (ppm)

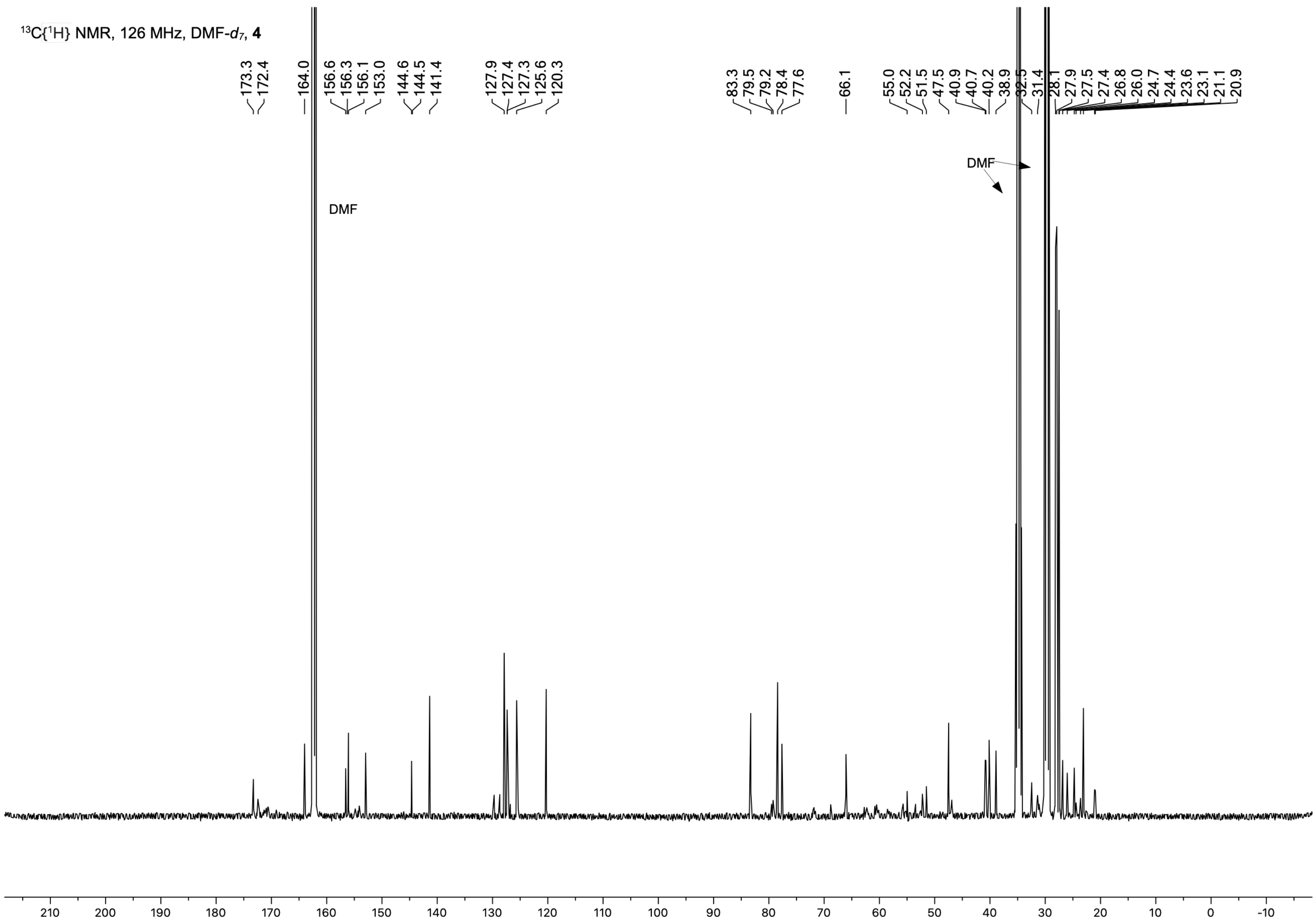

$^{13}C\{^{1}H\}$ NMR, 126 MHz, DMF-d_7, **4**
173.3
172.4
164.0
156.6
156.3
156.1
153.0
144.6
144.5
141.4
127.9
127.4
127.3
125.6
120.3
83.3
79.5
79.2
78.4
77.6
66.1
55.0
52.2
51.5
47.5
40.9
40.7
40.2
38.9
32.5
31.4
28.1
27.9
27.5
27.4
26.8
26.0
24.7
24.4
23.6
23.1
21.1
20.9
DMF
DMF
210
200
190
180
170
160
150
140
130
120
110
100
90
80
70
60
50
40
30
20
10
0
-10

HSQC, DMF-d_7, **4**

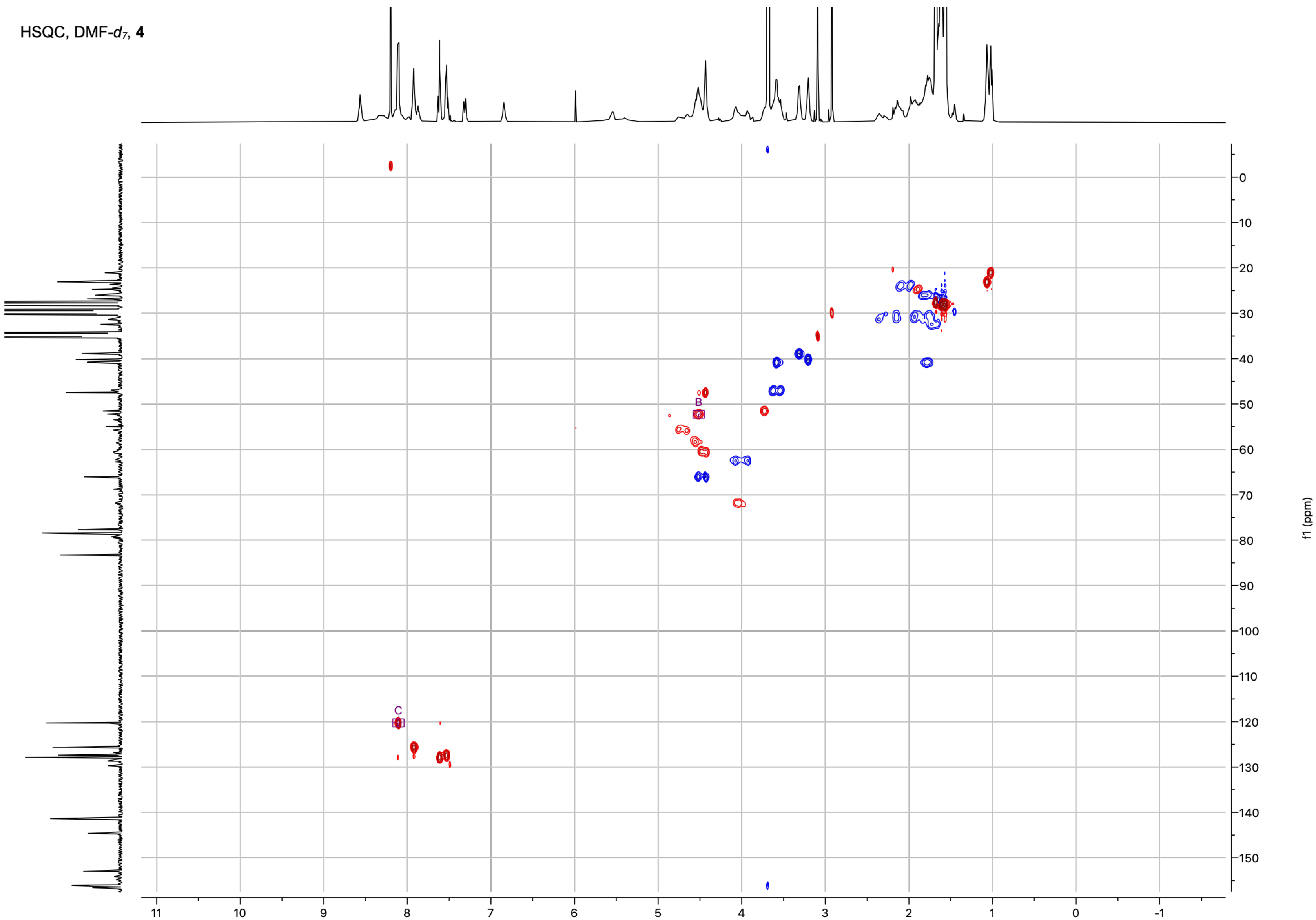

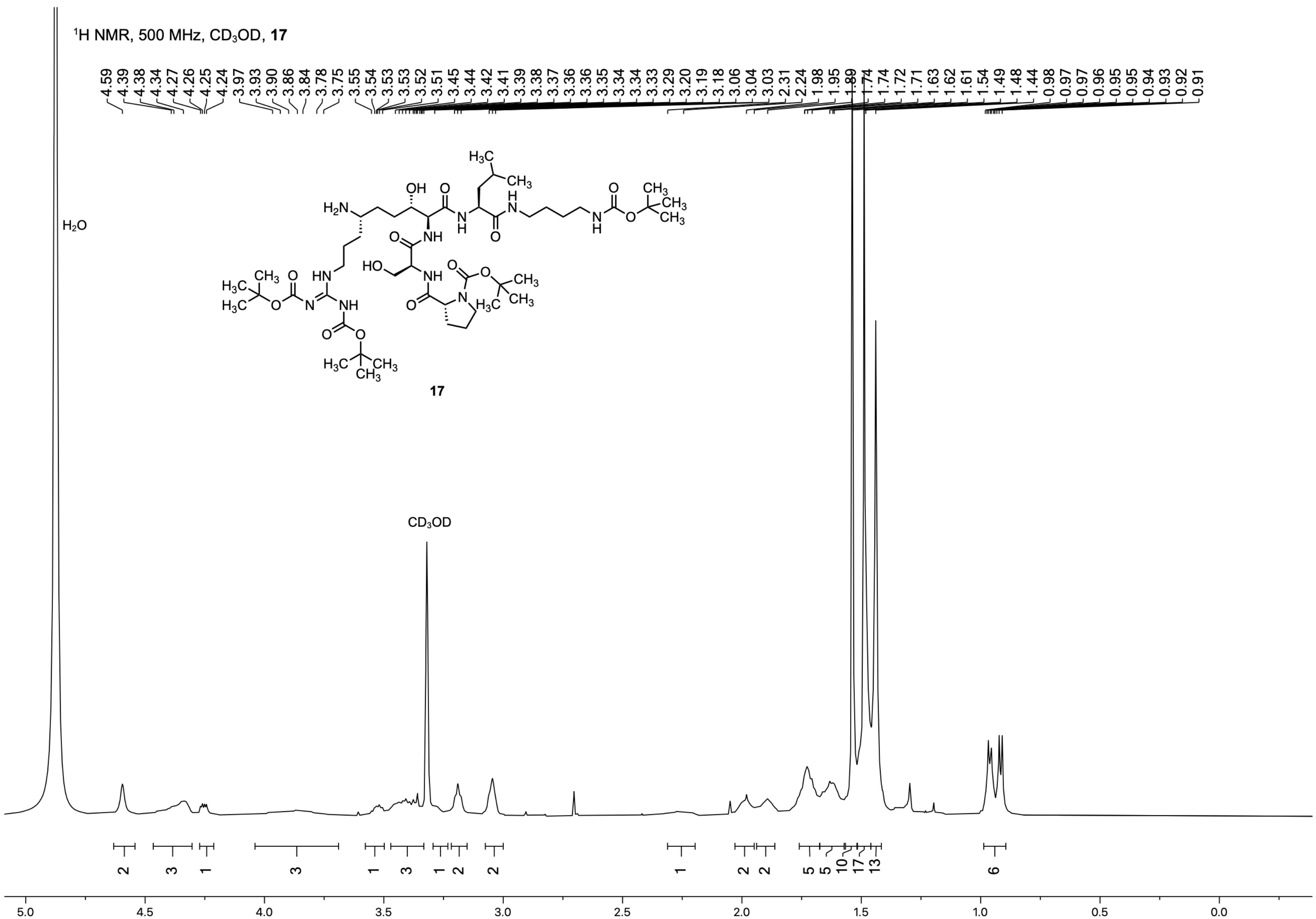

1H NMR, 500 MHz, CD3OD, 17
4.59 4.39 4.38 4.34 4.27 4.26 4.25 4.24 3.97 3.93 3.90 3.86 3.84 3.78 3.75 3.55 3.54 3.53 3.53 3.52 3.51 3.45 3.44 3.42 3.41 3.39 3.38 3.37 3.36 3.36 3.35 3.34 3.34 3.33 3.29 3.20 3.19 3.18 3.06 3.04 3.03 2.31 2.24 1.98 1.95 1.89 1.74 1.74 1.72 1.71 1.63 1.62 1.61 1.54 1.49 1.48 1.44 0.98 0.97 0.97 0.96 0.95 0.95 0.94 0.93 0.92 0.91
H2O
CD3OD
17
2 3 1 3 1 3 1 2 2 1 2 2 5 5 10 17 13 6
5.0 4.5 4.0 3.5 3.0 2.5 2.0 1.5 1.0 0.5 0.0

COSY, CD_3OD, **17**

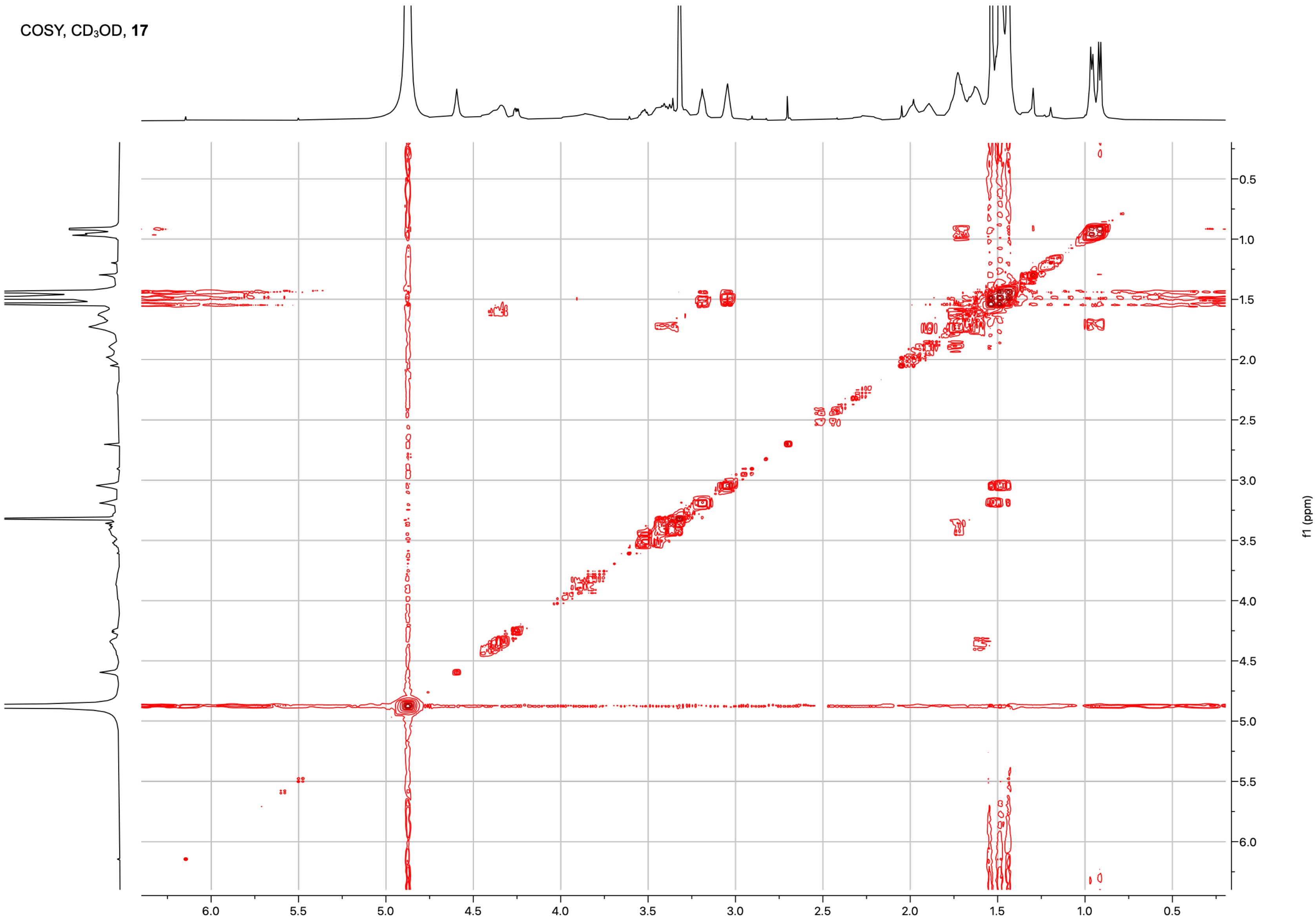

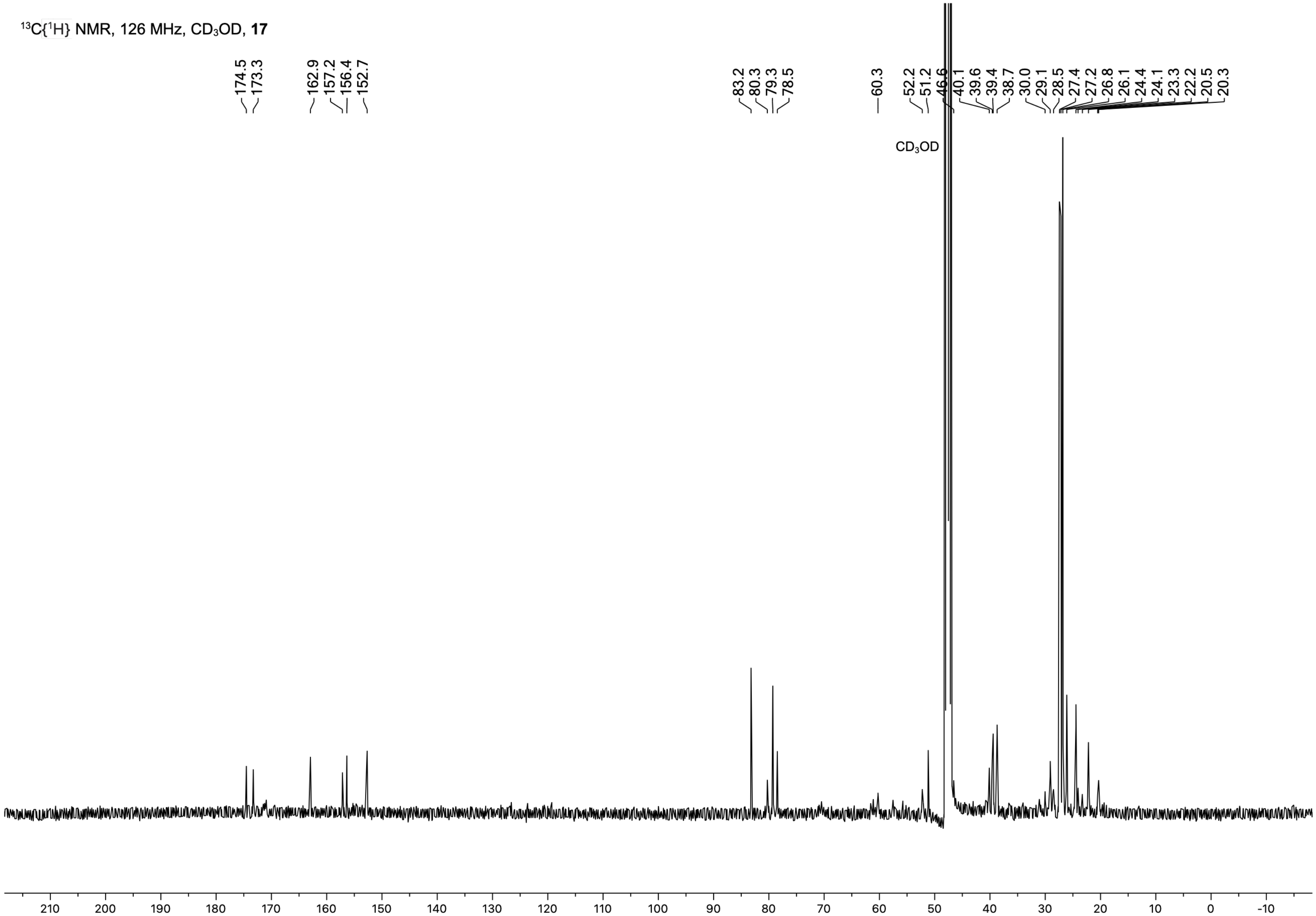

$^{13}C\{^1H\}$ NMR, 126 MHz, CD_3OD, **17**
174.5
173.3
162.9
157.2
156.4
152.7
83.2
80.3
79.3
78.5
60.3
52.2
51.2
46.6
40.1
39.6
39.4
38.7
30.0
29.1
28.5
27.4
27.2
26.8
26.1
24.4
24.1
23.3
22.2
20.5
20.3
CD_3OD
210
200
190
180
170
160
150
140
130
120
110
100
90
80
70
60
50
40
30
20
10
0
-10

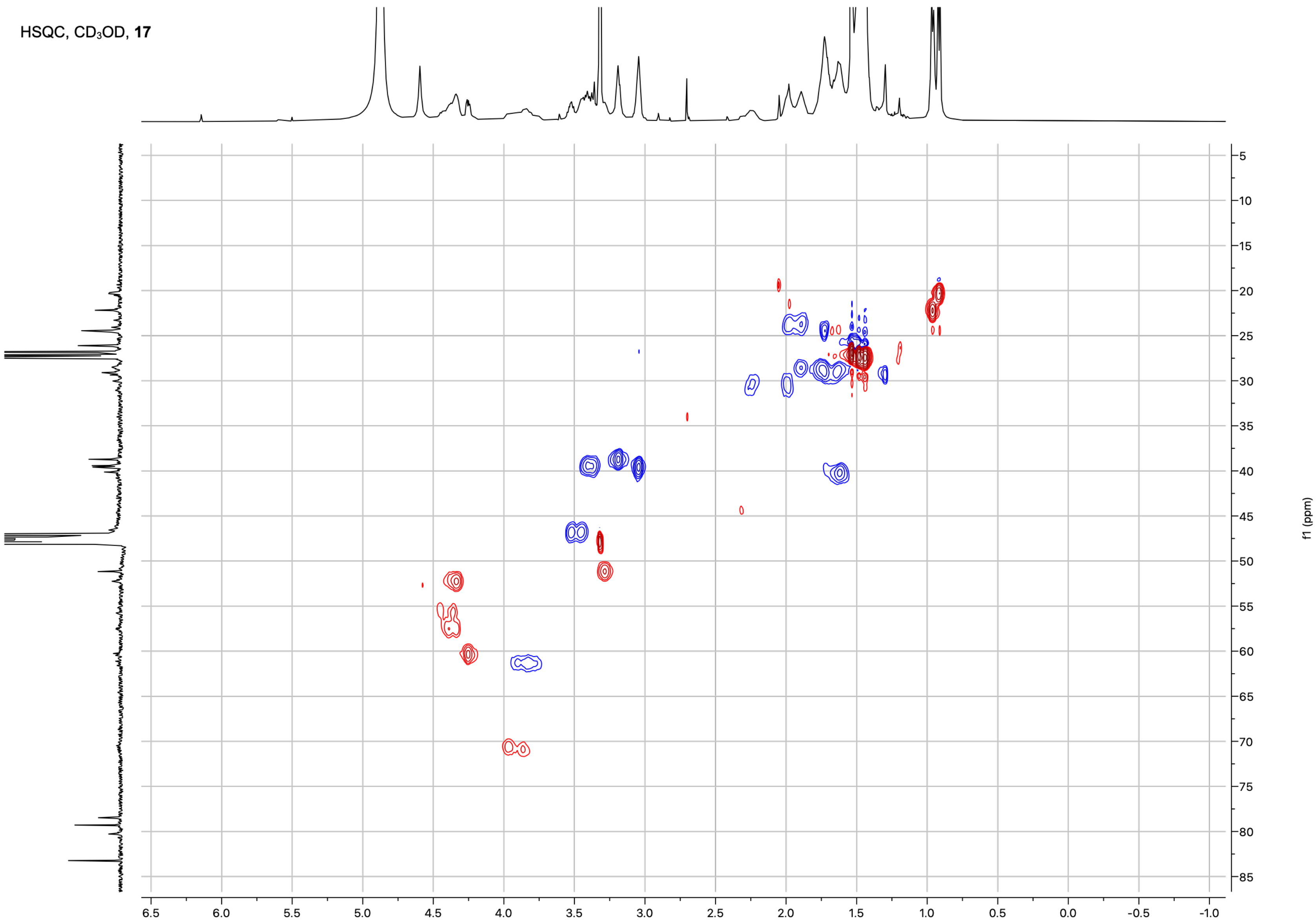
HSQC, CD3OD, 17
f1 (ppm)

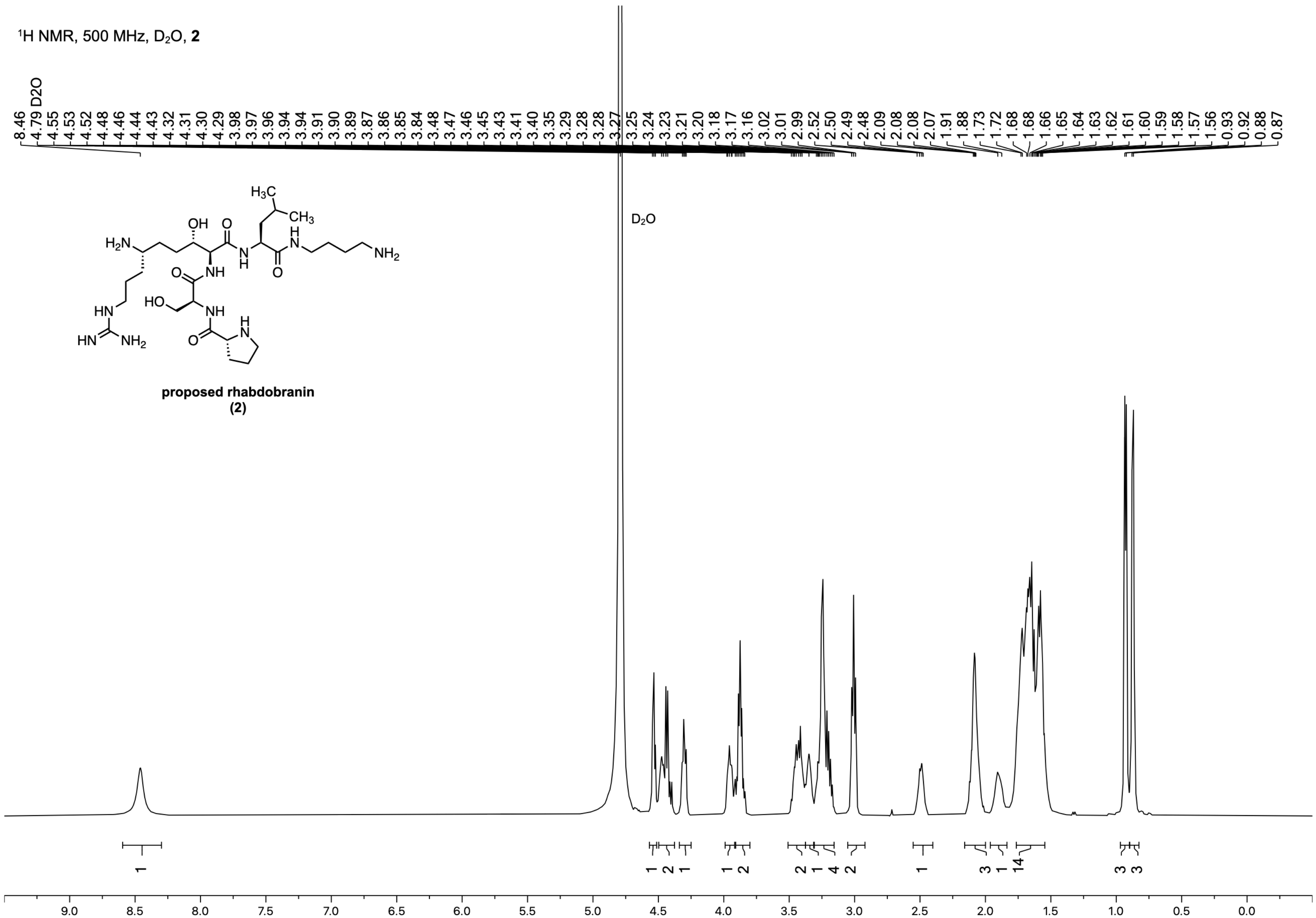

1H NMR, 500 MHz, D2O, 2
8.46 D2O 4.79 4.55 4.53 4.52 4.48 4.46 4.44 4.43 4.32 4.31 4.30 4.29 3.98 3.97 3.96 3.94 3.94 3.91 3.90 3.89 3.87 3.86 3.85 3.84 3.48 3.47 3.46 3.45 3.43 3.41 3.40 3.35 3.29 3.28 3.28 3.27 3.25 3.24 3.23 3.21 3.20 3.18 3.17 3.16 3.02 3.01 2.99 2.52 2.50 2.49 2.48 2.09 2.08 2.08 2.07 1.91 1.88 1.73 1.72 1.68 1.68 1.66 1.65 1.64 1.63 1.62 1.61 1.60 1.59 1.58 1.57 1.56 0.93 0.92 0.88 0.87
D2O
proposed rhabdobranin
(2)

COSY, D_2O, **2**

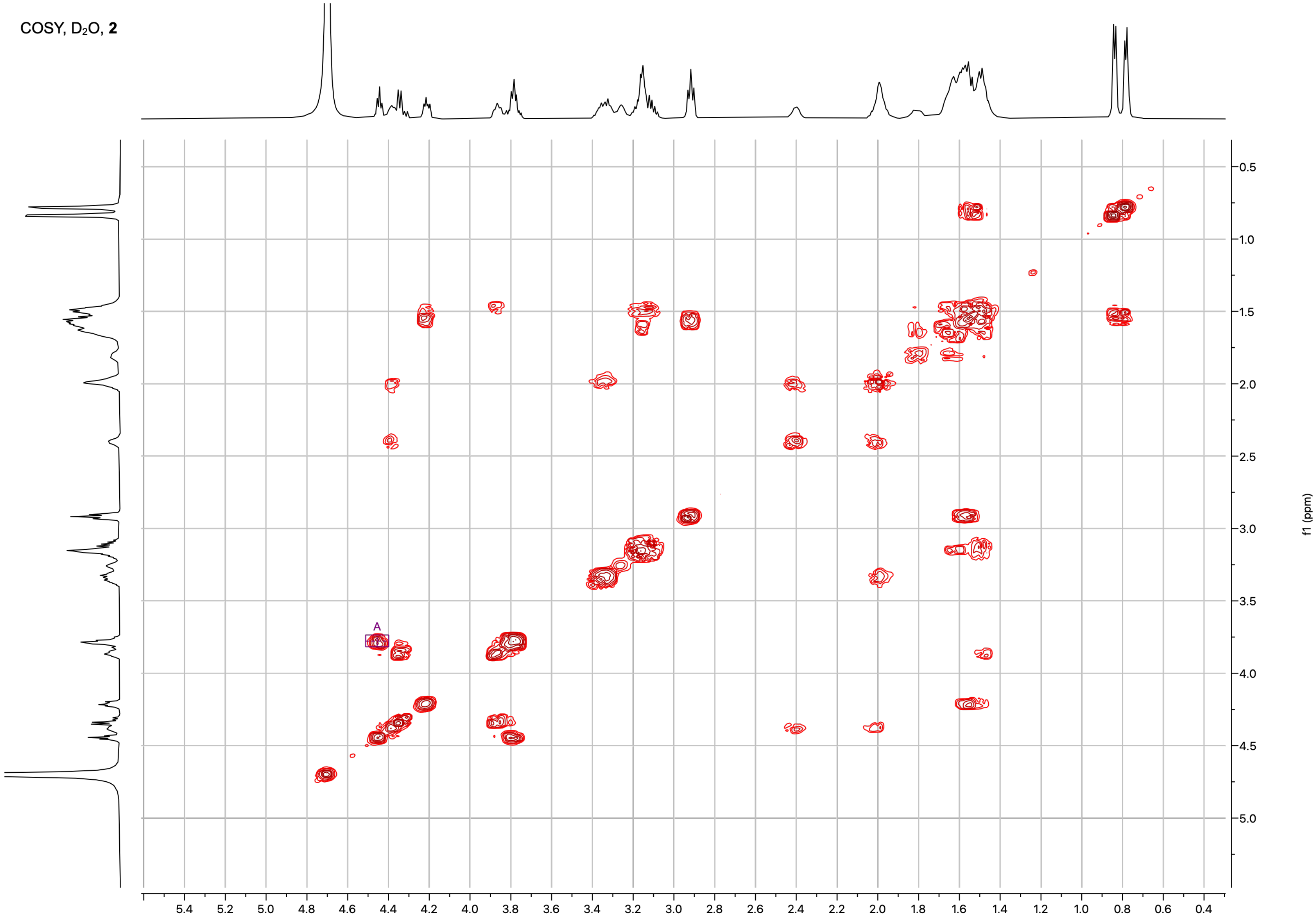

A
f1 (ppm)
0.5
1.0
1.5
2.0
2.5
3.0
3.5
4.0
4.5
5.0
5.4
5.2
5.0
4.8
4.6
4.4
4.2
4.0
3.8
3.6
3.4
3.2
3.0
2.8
2.6
2.4
2.2
2.0
1.8
1.6
1.4
1.2
1.0
0.8
0.6
0.4

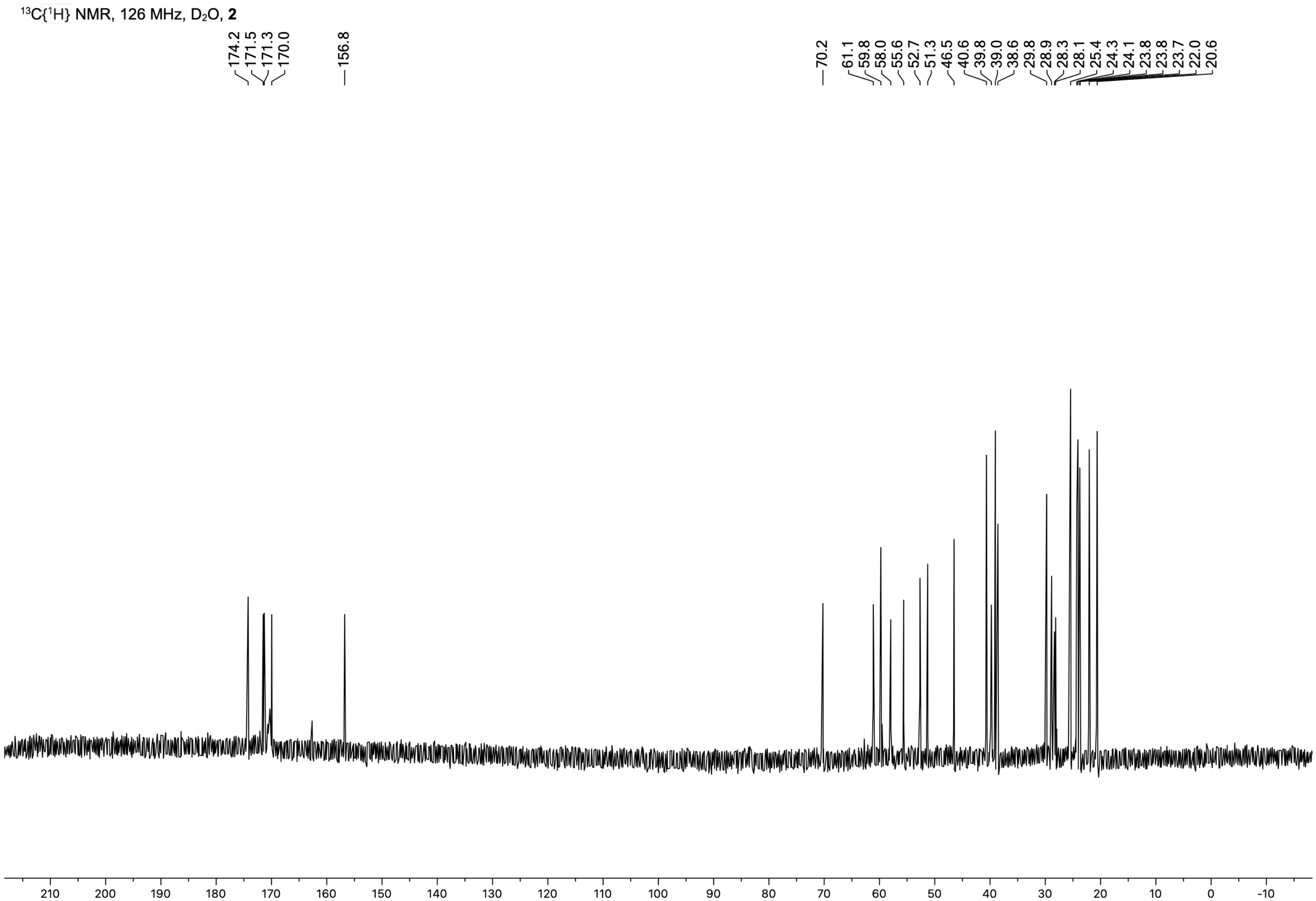

$^{13}C\{^1H\}$ NMR, 126 MHz, D_2O, **2**
174.2
171.5
171.3
170.0
156.8
70.2
61.1
59.8
58.0
55.6
52.7
51.3
46.5
40.6
39.8
39.0
38.6
29.8
28.9
28.3
28.1
25.4
24.3
24.1
23.8
23.8
23.7
22.0
20.6
210
200
190
180
170
160
150
140
130
120
110
100
90
80
70
60
50
40
30
20
10
0
-10

HSQC, D_2O, **2**

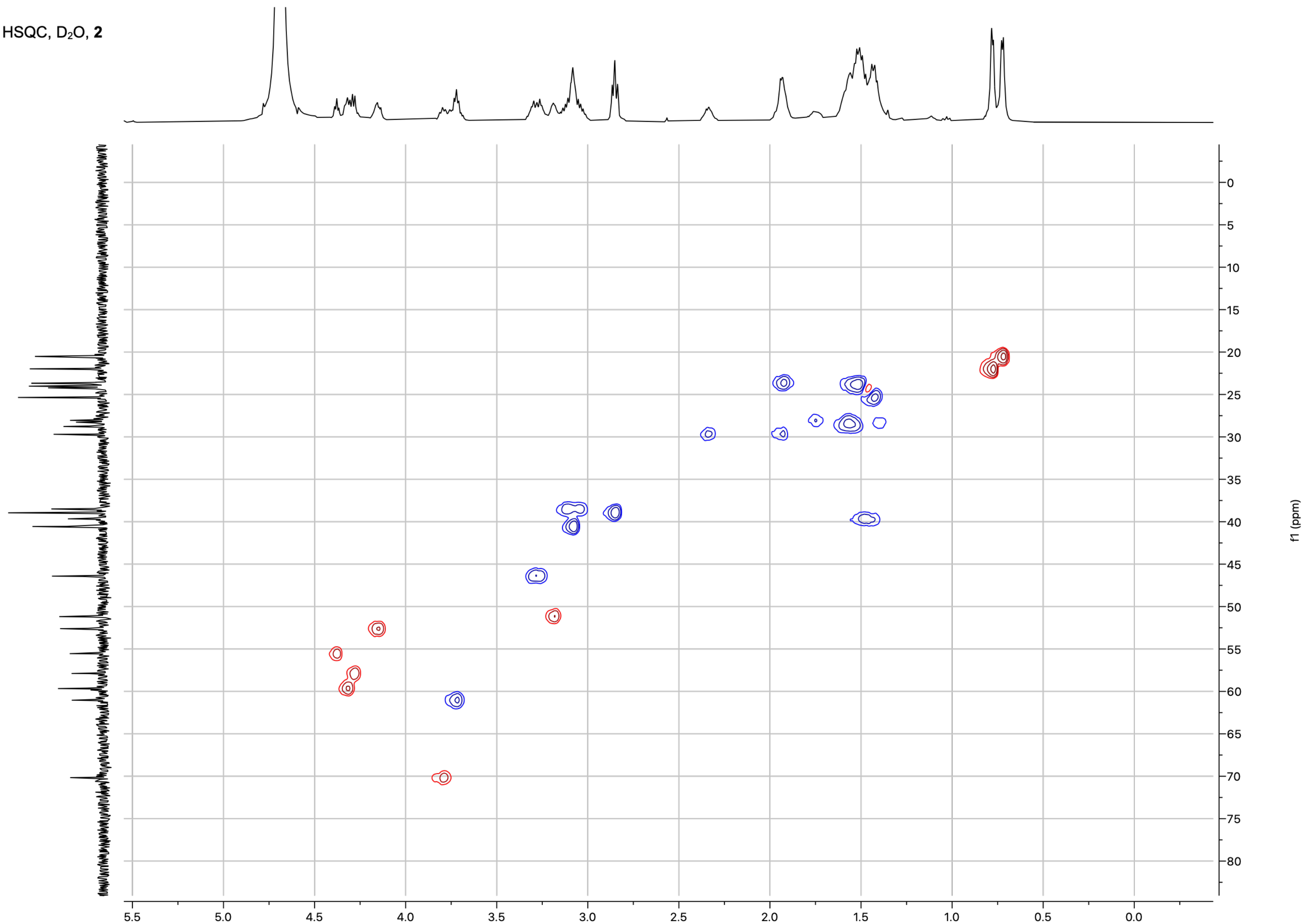

5.5
5.0
4.5
4.0
3.5
3.0
2.5
2.0
1.5
1.0
0.5
0.0
0
5
10
15
20
25
30
35
40
45
50
55
60
65
70
75
80
f1 (ppm)

^{1}H NMR, 500 MHz, DMSO-d_6, **18**

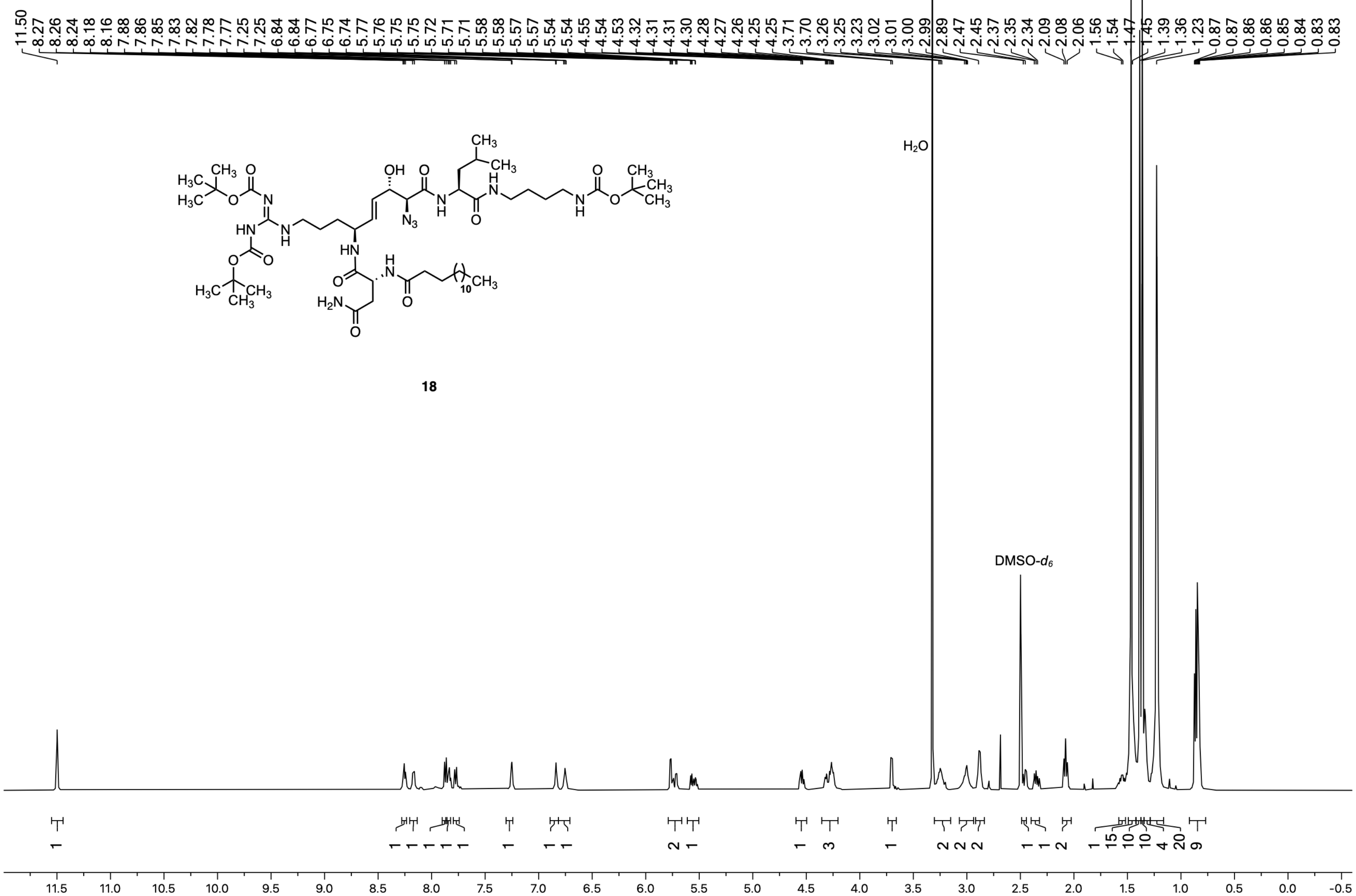

COSY, DMSO-d_6, **18**

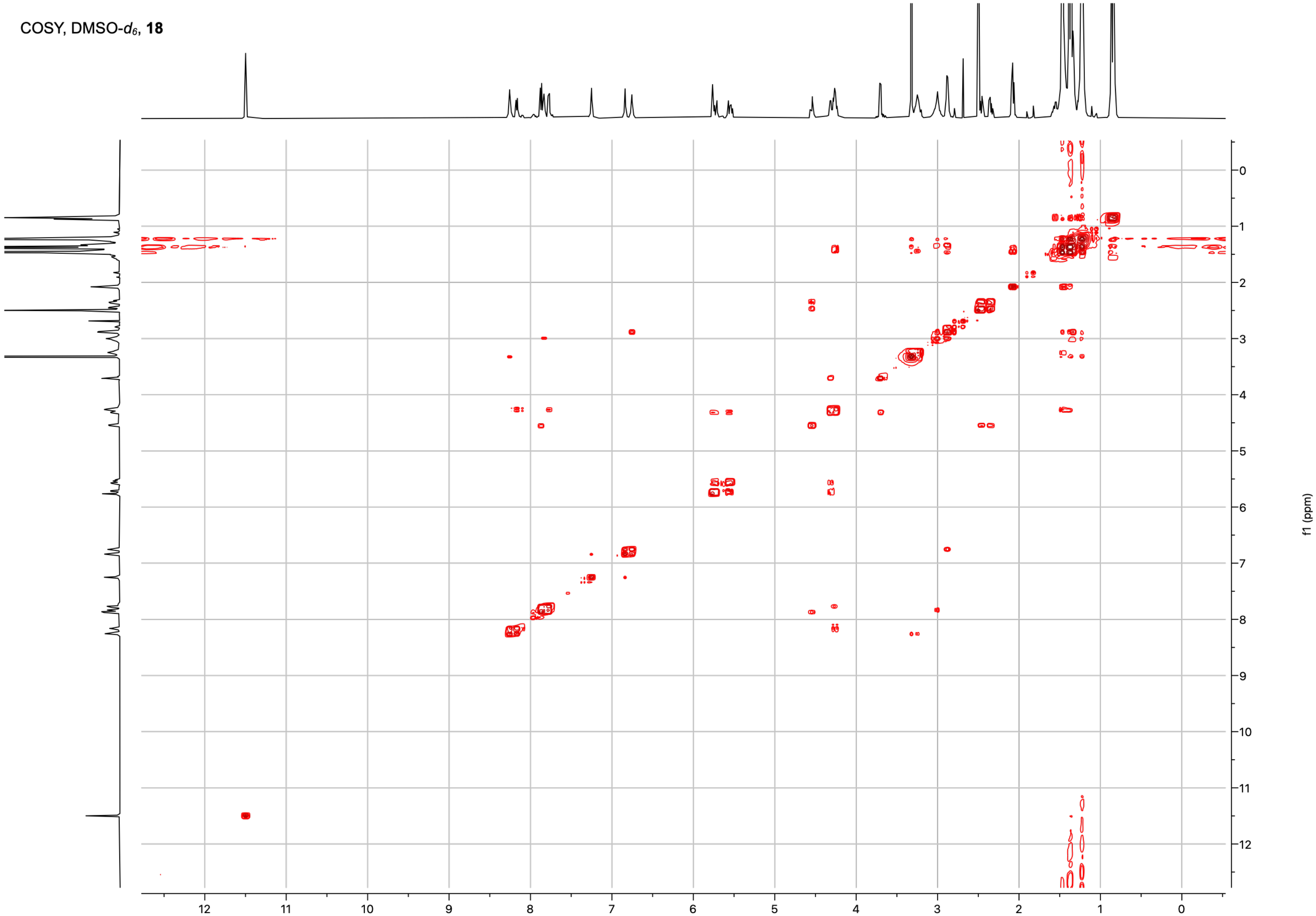

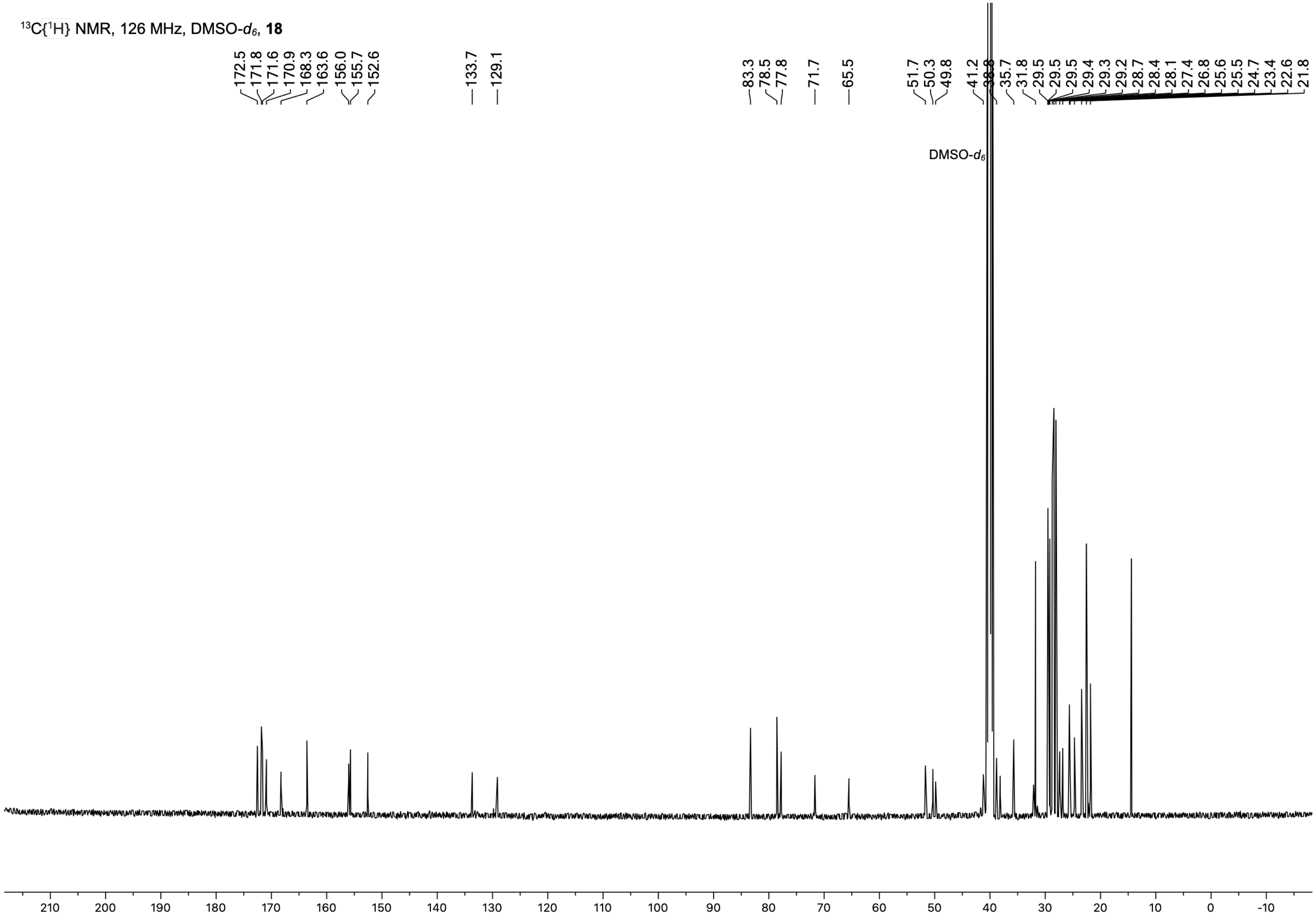

$^{13}C\{^1H\}$ NMR, 126 MHz, DMSO-d_6, **18**
172.5
171.8
171.6
170.9
168.3
163.6
156.0
155.7
152.6
133.7
129.1
83.3
78.5
77.8
71.7
65.5
51.7
50.3
49.8
41.2
38.8
35.7
31.8
29.5
29.5
29.5
29.4
29.3
29.2
28.7
28.4
28.1
27.4
26.8
25.6
25.5
24.7
23.4
22.6
21.8
DMSO-d_6
210 200 190 180 170 160 150 140 130 120 110 100 90 80 70 60 50 40 30 20 10 0 -10

HSQC, DMSO-d_6, **18**

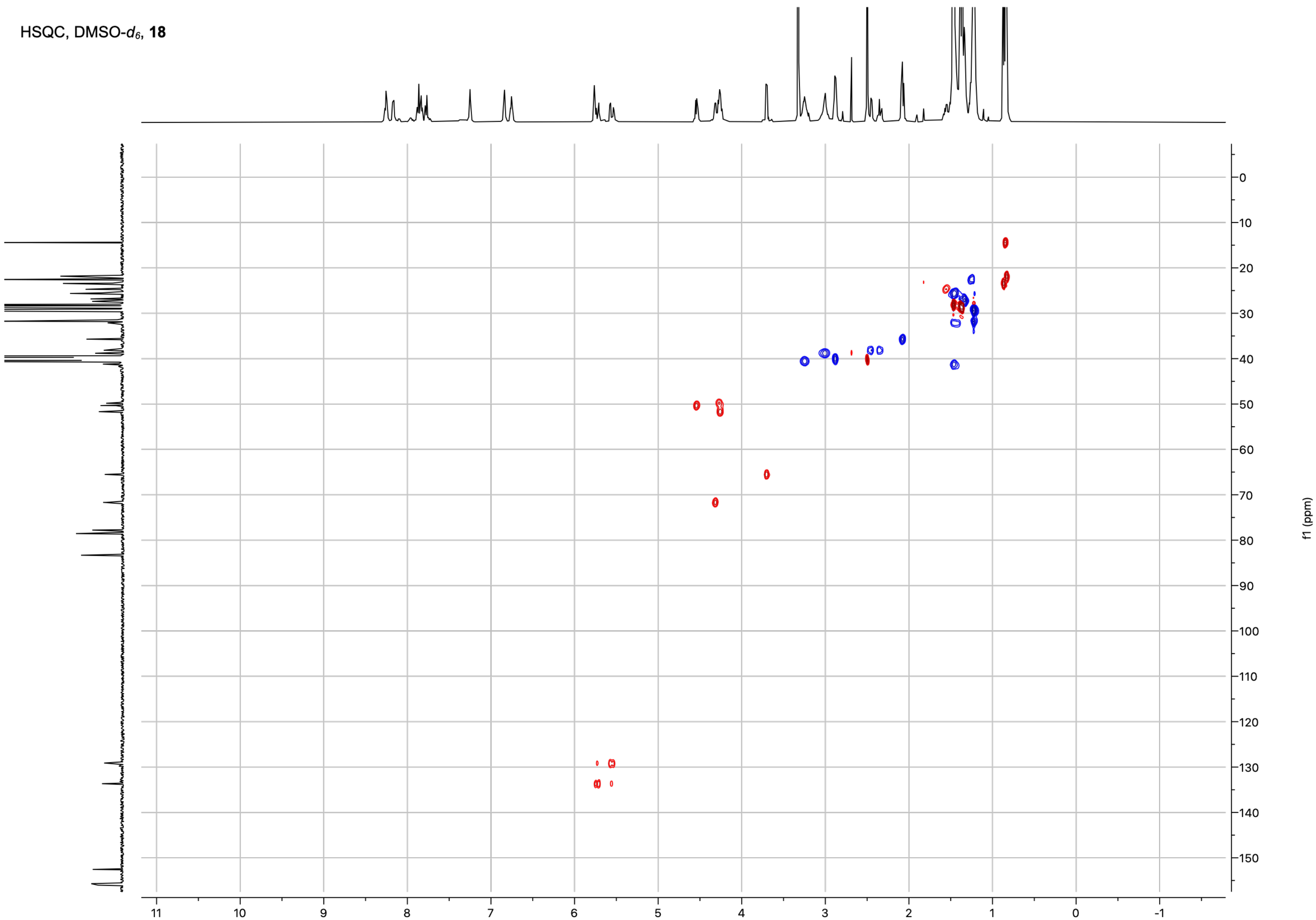

^{1}H NMR, 500 MHz, DMSO-d_6, **S12**

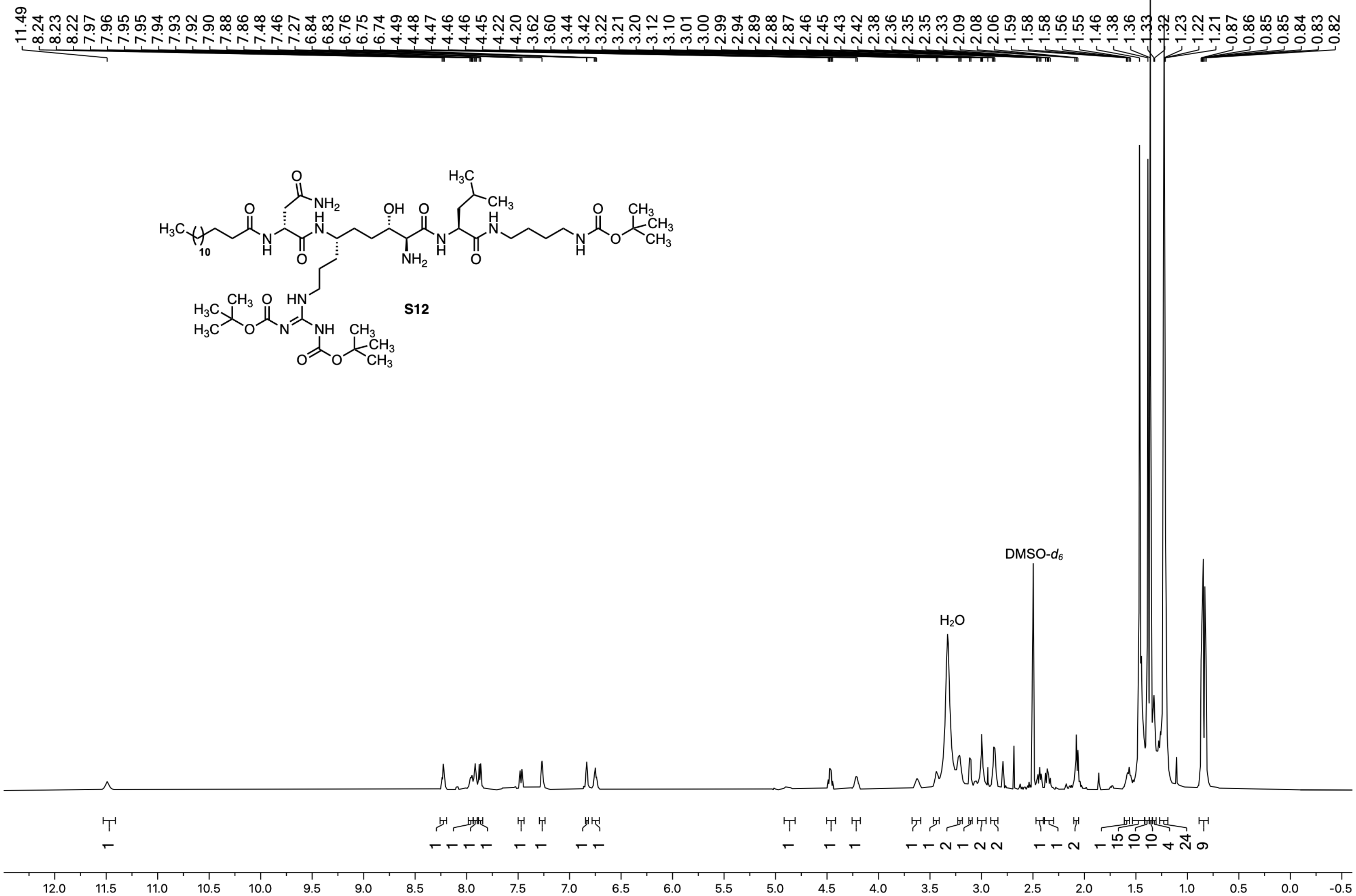

COSY, DMSO-d_6, **S12**

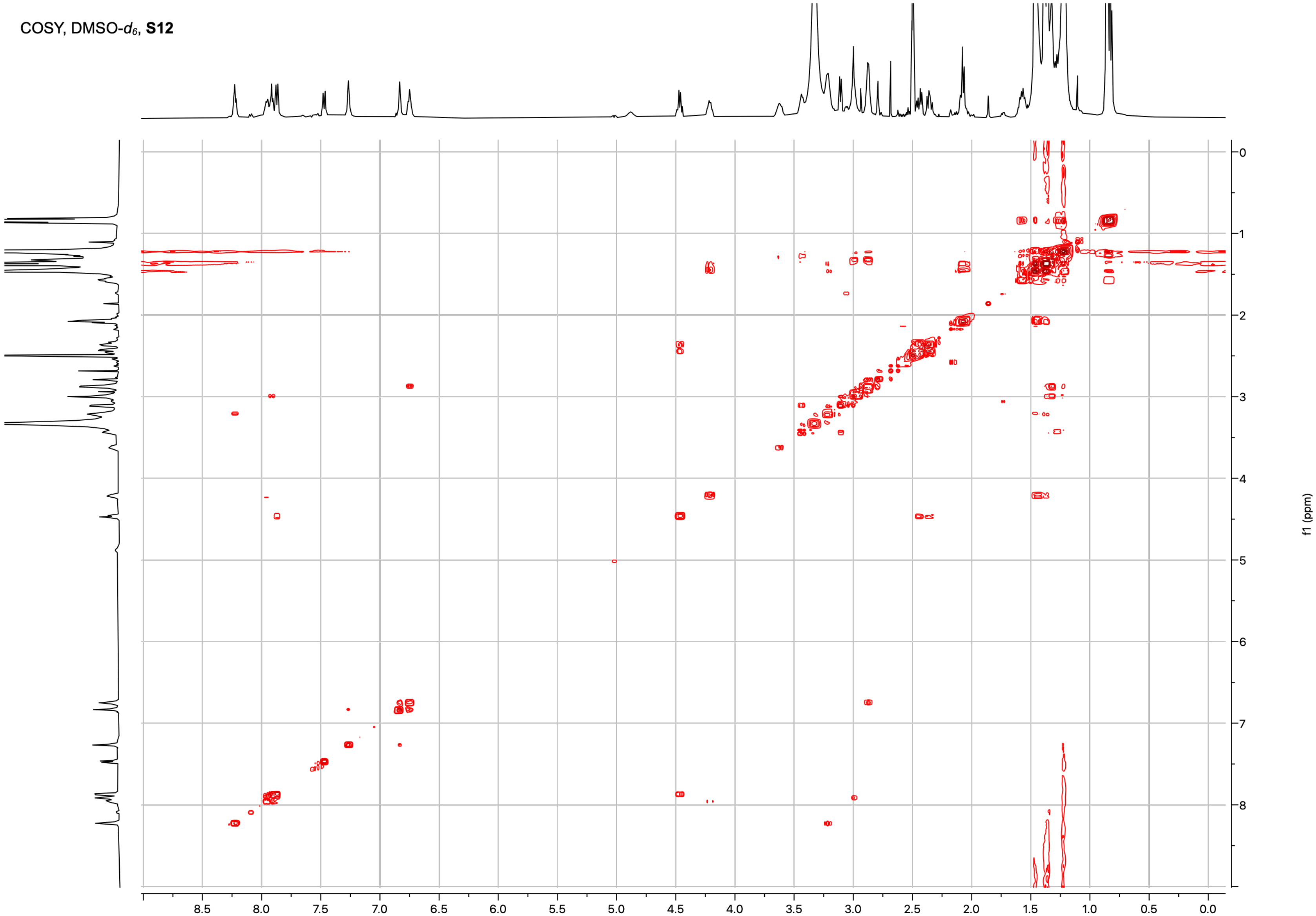

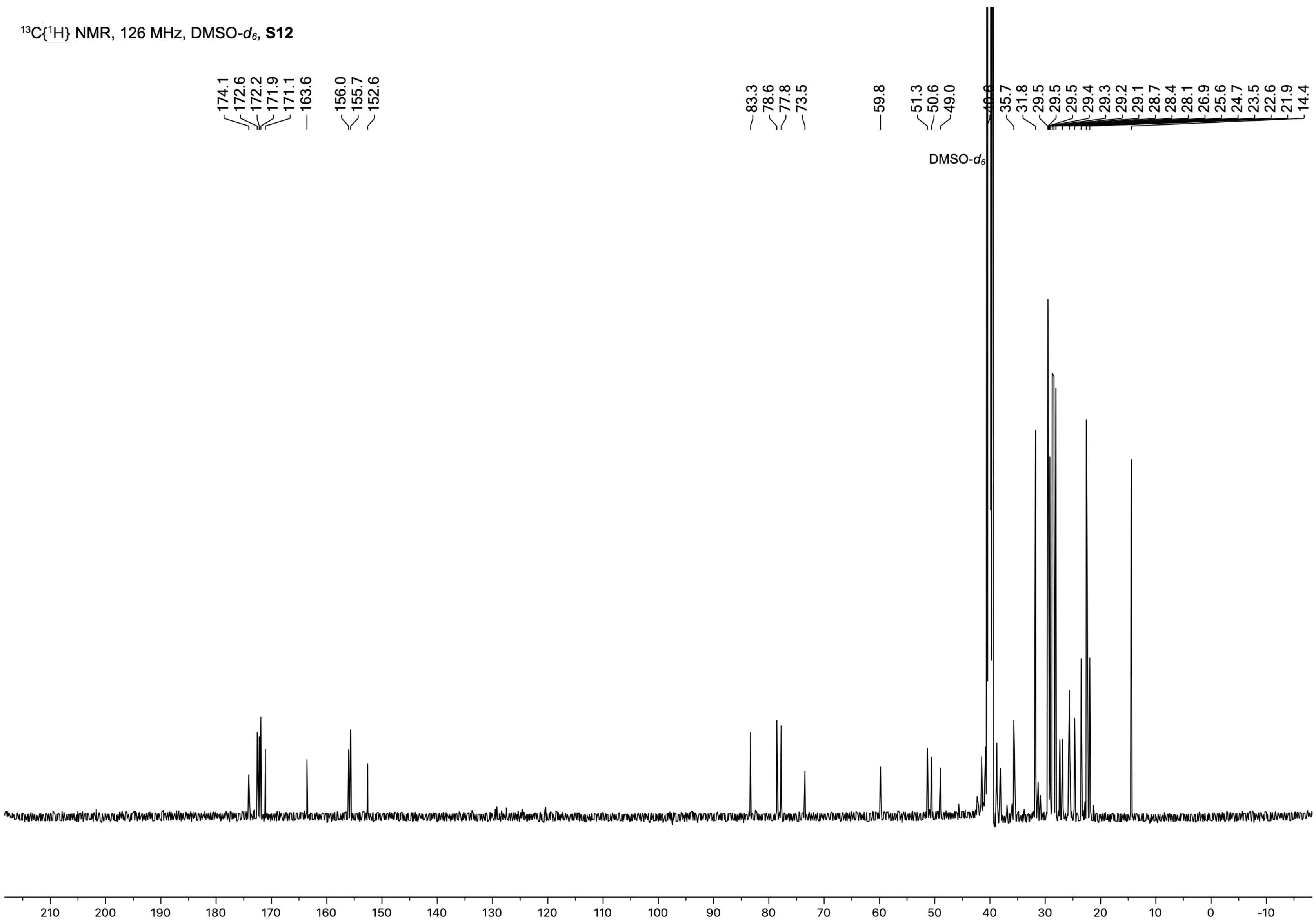

$^{13}C\{^1H\}$ NMR, 126 MHz, DMSO-d_6, **S12**
174.1
172.6
172.2
171.9
171.1
163.6
156.0
155.7
152.6
83.3
78.6
77.8
73.5
59.8
51.3
50.6
49.0
35.7
31.8
29.5
29.5
29.5
29.4
29.3
29.2
29.1
28.7
28.4
28.1
26.9
25.6
24.7
23.5
22.6
21.9
14.4
DMSO-d_6
210
200
190
180
170
160
150
140
130
120
110
100
90
80
70
60
50
40
30
20
10
0
-10

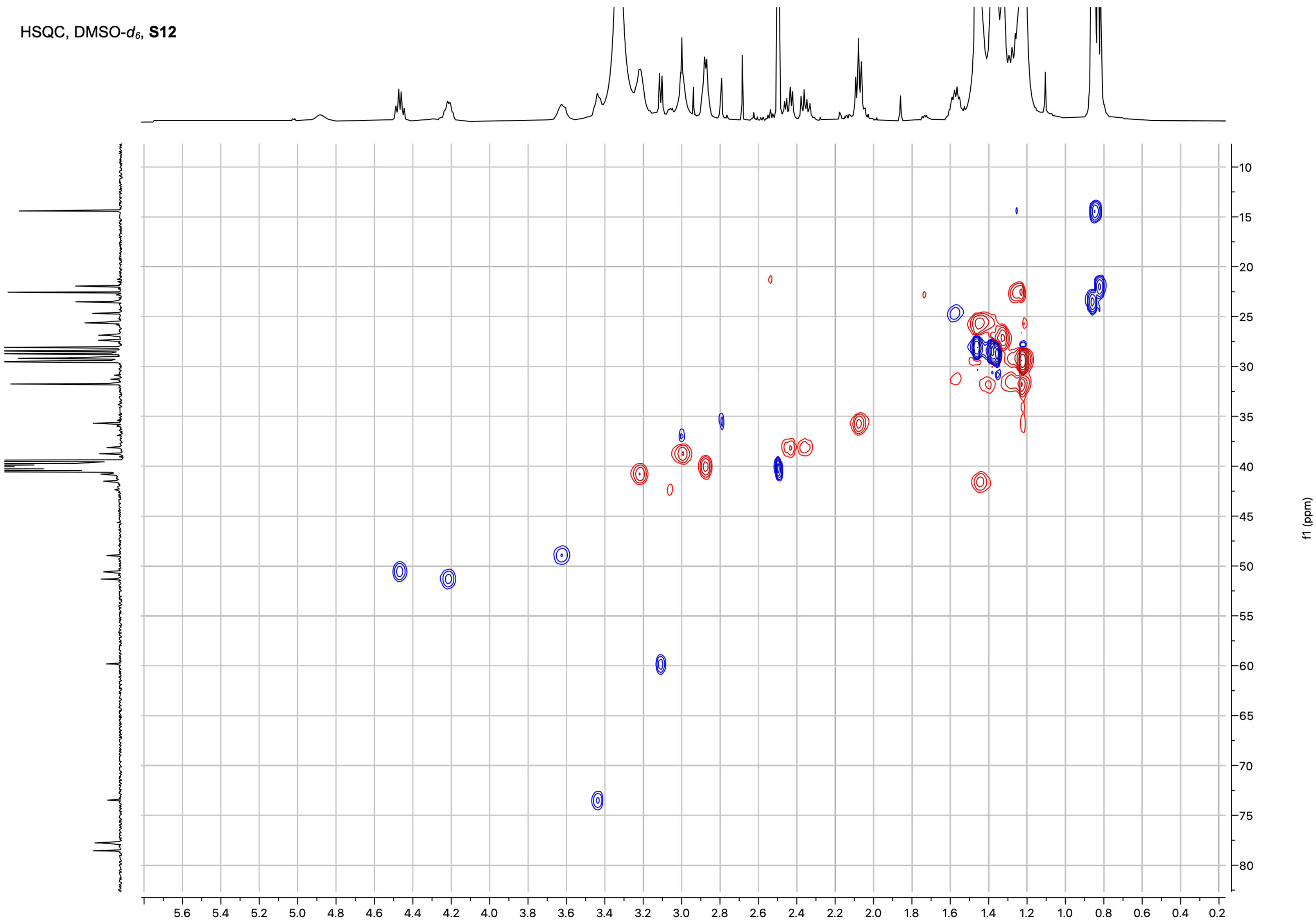
HSQC, DMSO-d_6, **S12**
f1 (ppm)
10
15
20
25
30
35
40
45
50
55
60
65
70
75
80
5.6
5.4
5.2
5.0
4.8
4.6
4.4
4.2
4.0
3.8
3.6
3.4
3.2
3.0
2.8
2.6
2.4
2.2
2.0
1.8
1.6
1.4
1.2
1.0
0.8
0.6
0.4
0.2

^{1}H NMR, 600 MHz, DMF-d_7, **S13**

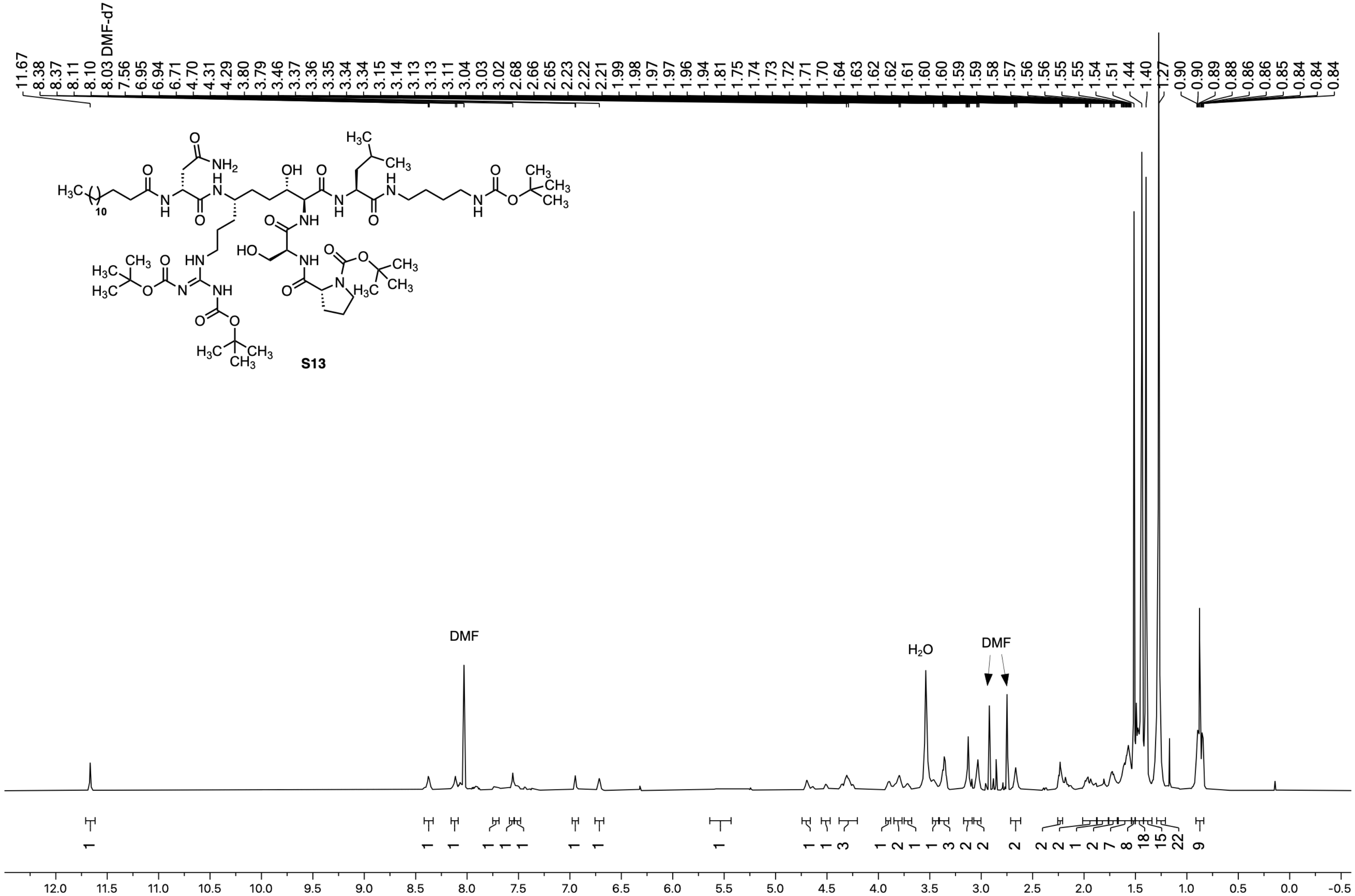

COSY, DMF-d_7, **13**

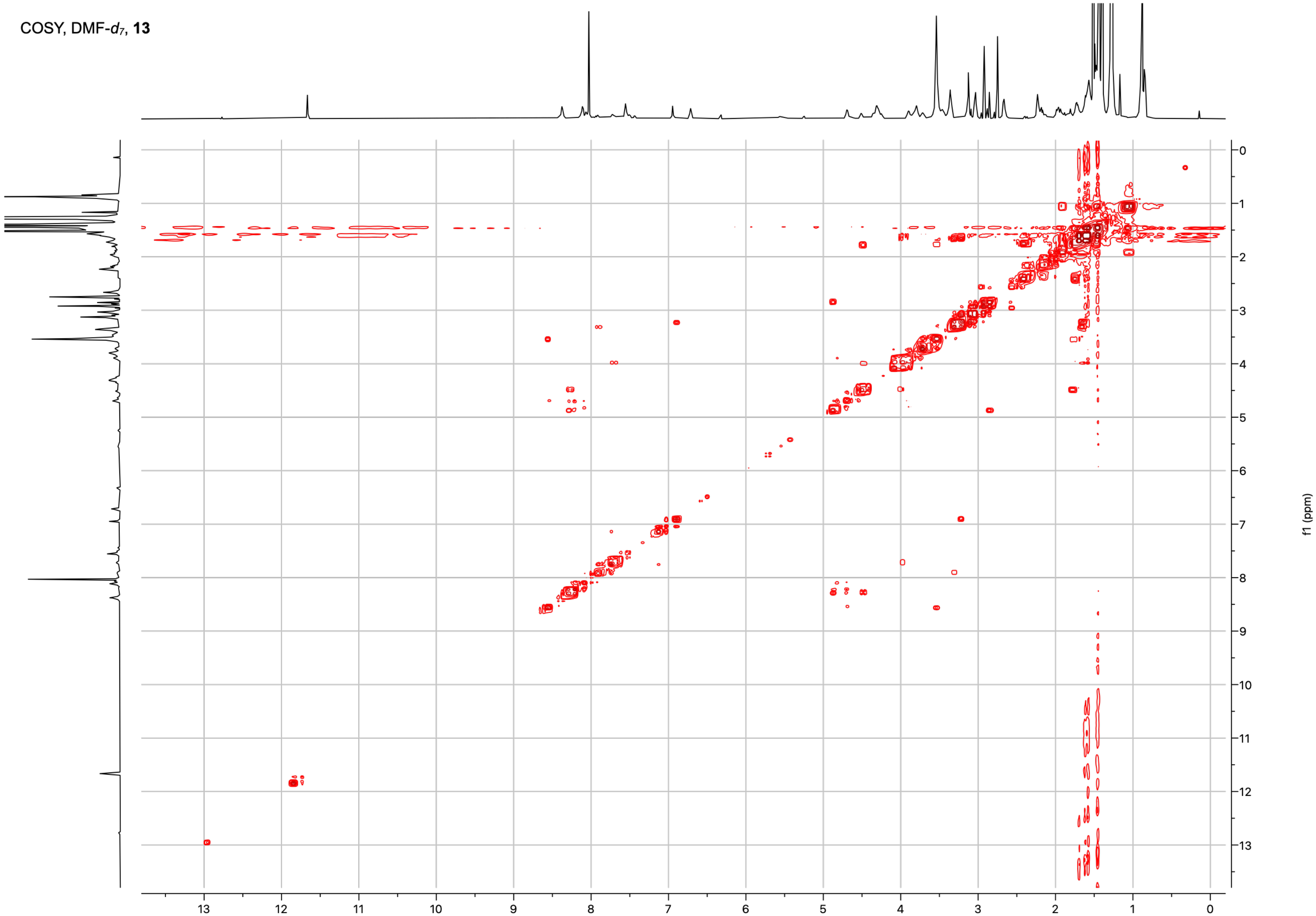

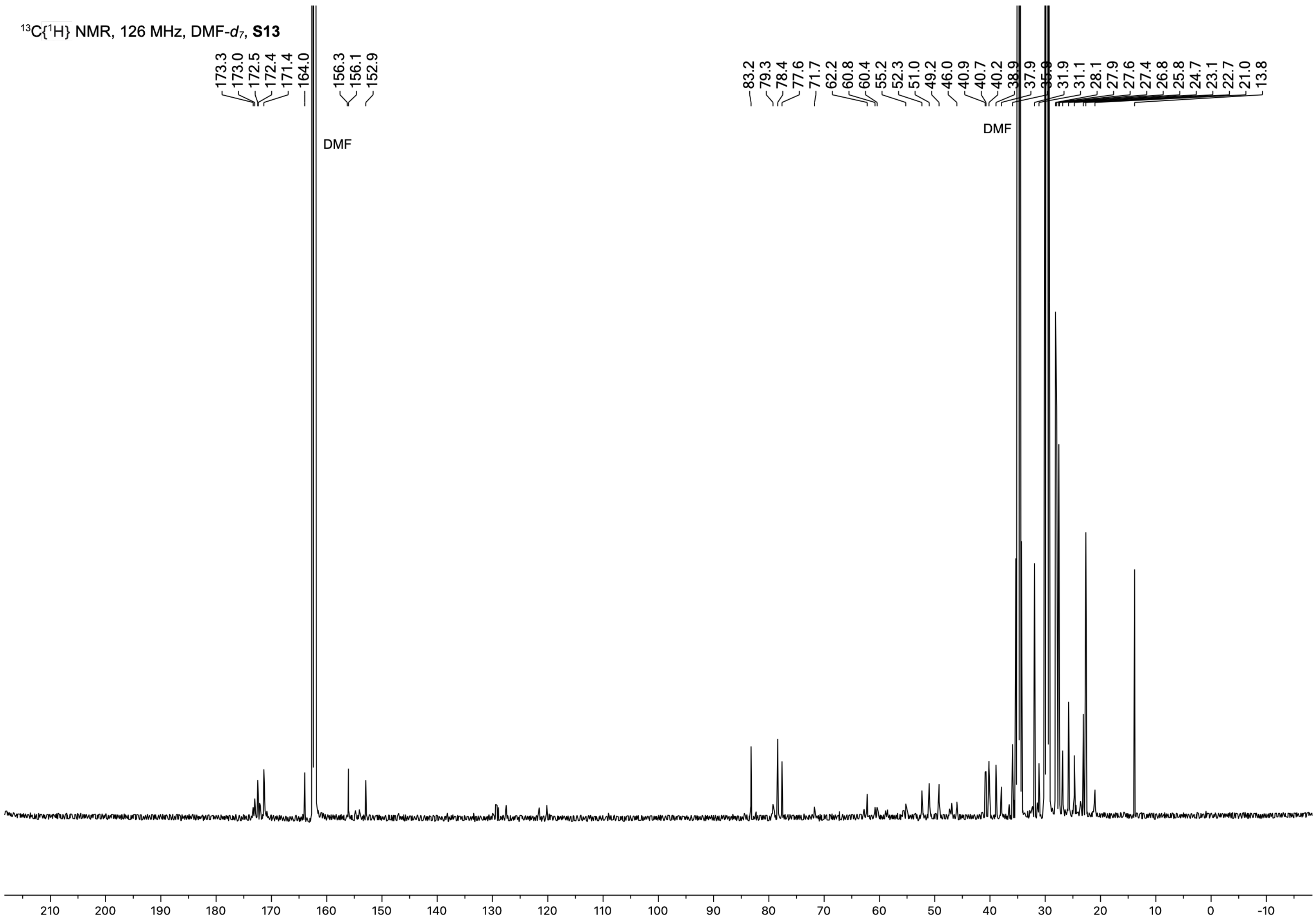

$^{13}C\{^1H\}$ NMR, 126 MHz, DMF-d_7, **S13**
173.3
173.0
172.5
172.4
171.4
164.0
156.3
156.1
152.9
DMF
83.2
79.3
78.4
77.6
71.7
62.2
60.8
60.4
55.2
52.3
51.0
49.2
46.0
40.9
40.7
40.2
38.9
37.9
31.9
31.1
28.1
27.9
27.6
27.4
26.8
25.8
24.7
23.1
22.7
21.0
13.8
DMF
210
200
190
180
170
160
150
140
130
120
110
100
90
80
70
60
50
40
30
20
10
0
-10

HSQC, DMF-d_7, **S13**

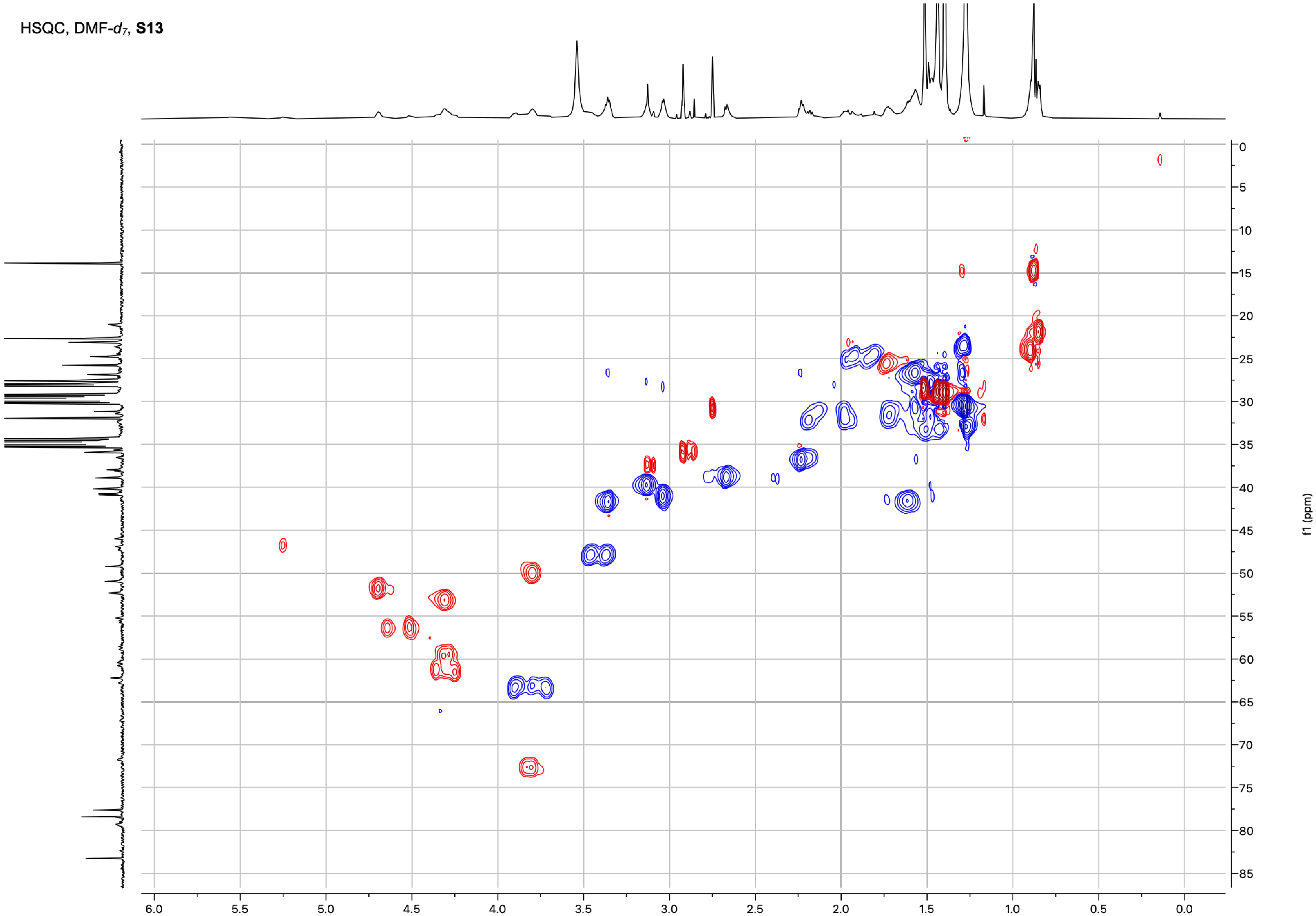

^{1}H NMR, 500 MHz, DMSO-d_6, **1**

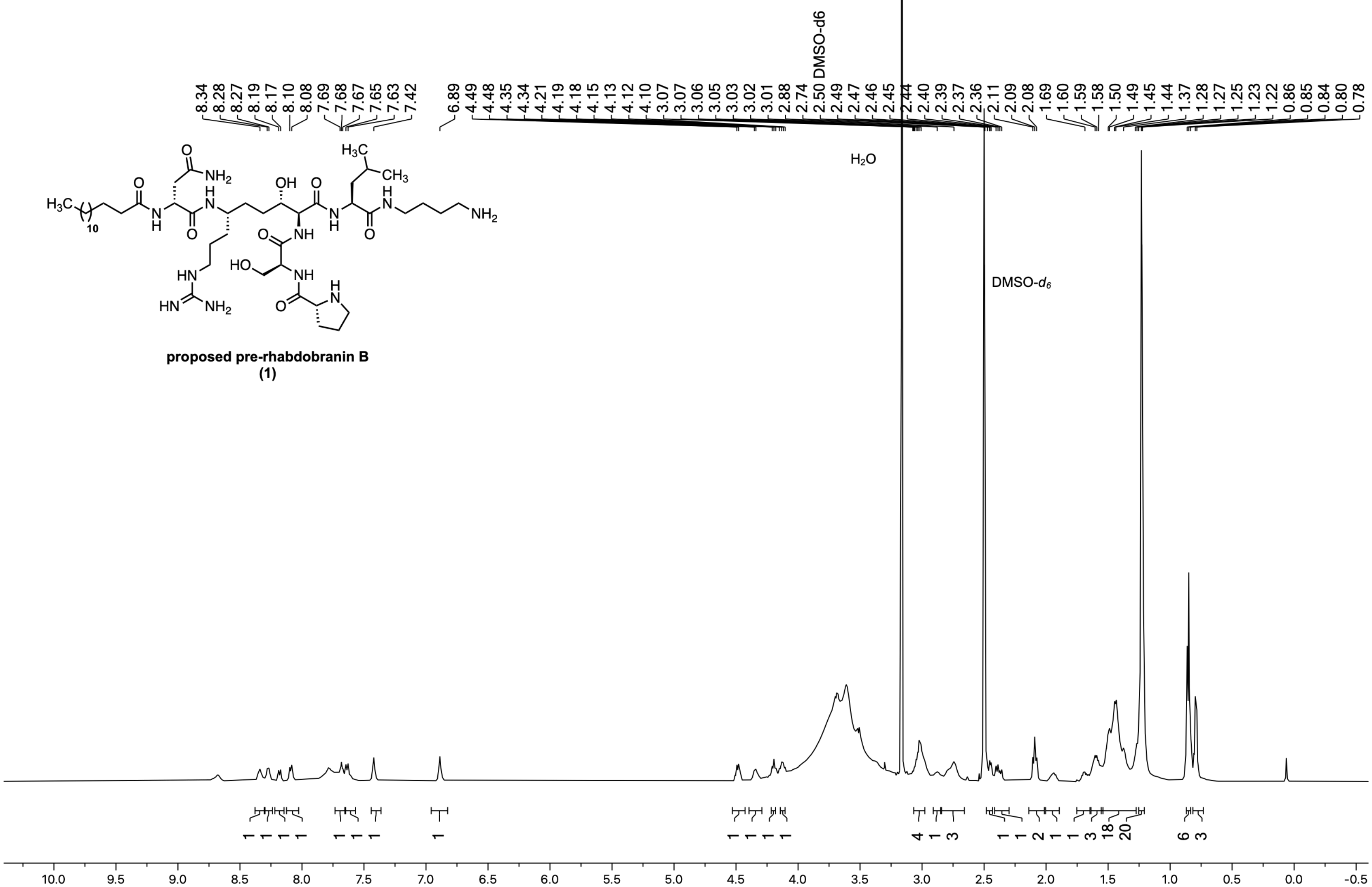

COSY, DMSO-d_6, **1**

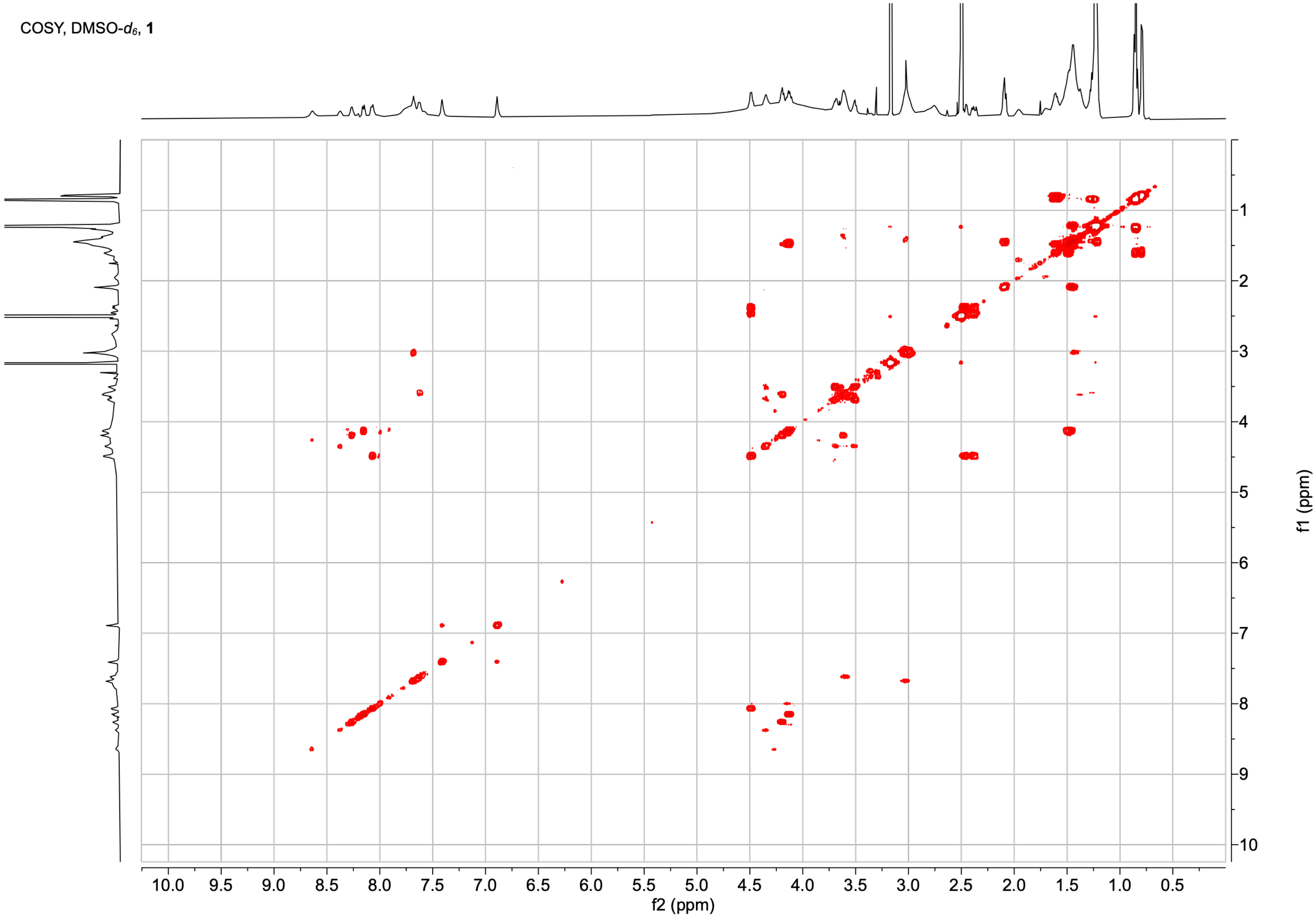

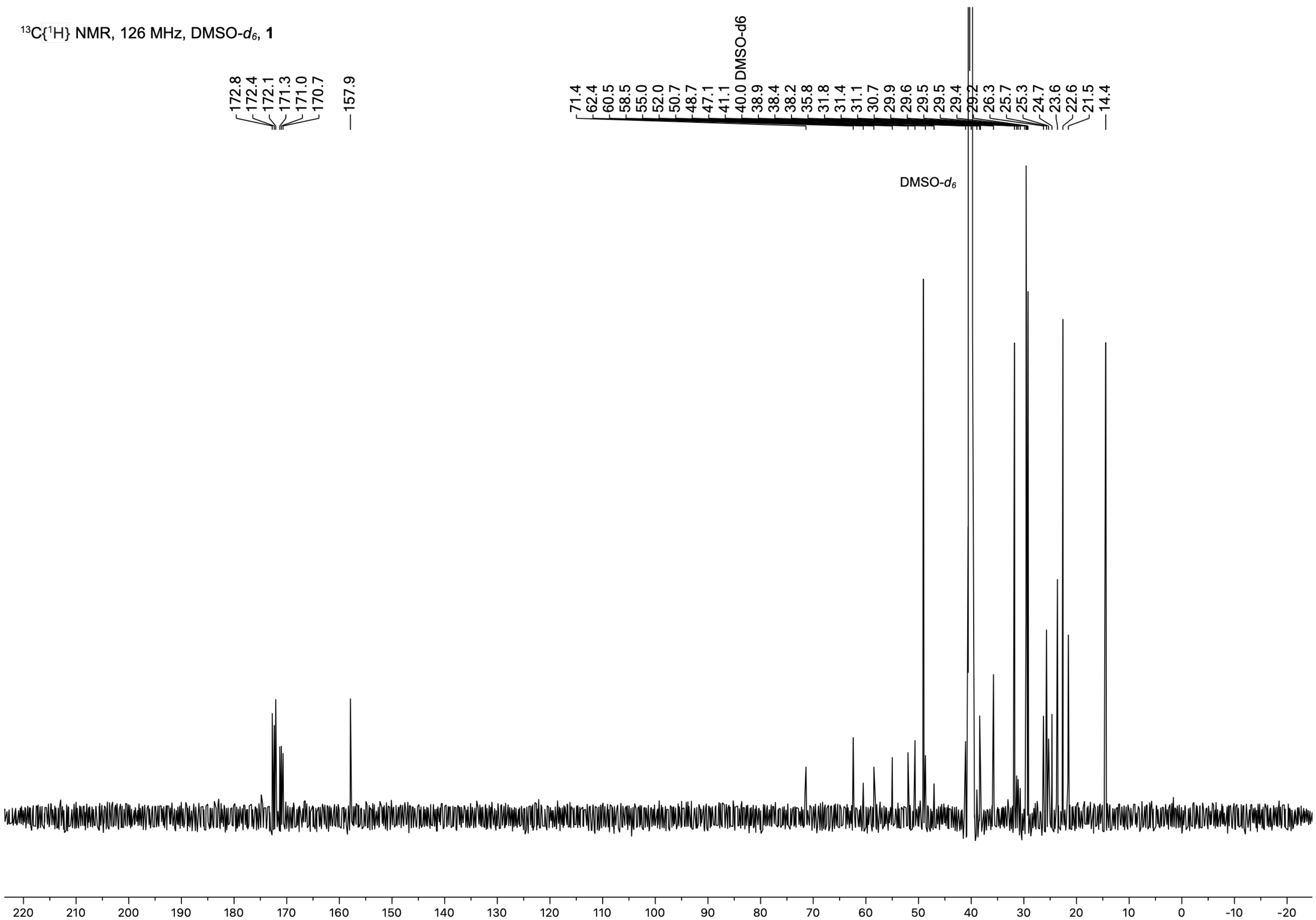

$^{13}C\{^{1}H\}$ NMR, 126 MHz, DMSO-d_6, **1**
172.8
172.4
172.1
171.3
171.0
170.7
157.9
71.4
62.4
60.5
58.5
55.0
52.0
50.7
48.7
47.1
41.1
40.0 DMSO-d6
38.9
38.4
38.2
35.8
31.8
31.4
31.1
30.7
29.9
29.6
29.5
29.5
29.4
29.2
26.3
25.7
25.3
24.7
23.6
22.6
21.5
14.4
DMSO-d_6
220 210 200 190 180 170 160 150 140 130 120 110 100 90 80 70 60 50 40 30 20 10 0 -10 -20

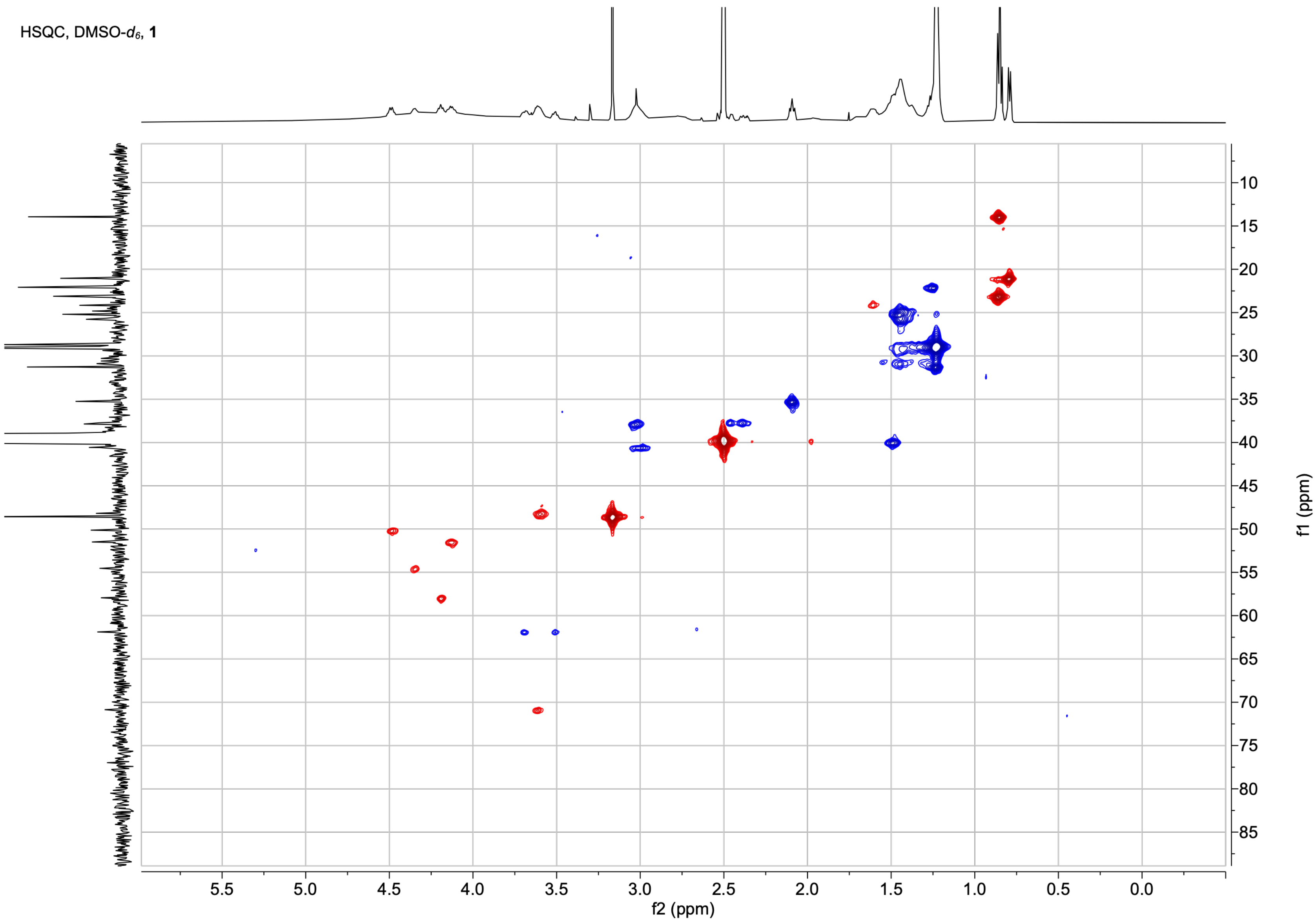

HSQC, DMSO-d_6, **1**
f2 (ppm)
f1 (ppm)
5.5
5.0
4.5
4.0
3.5
3.0
2.5
2.0
1.5
1.0
0.5
0.0
10
15
20
25
30
35
40
45
50
55
60
65
70
75
80
85

^{1}H NMR, 500 MHz, DMSO-d_6, **S15**

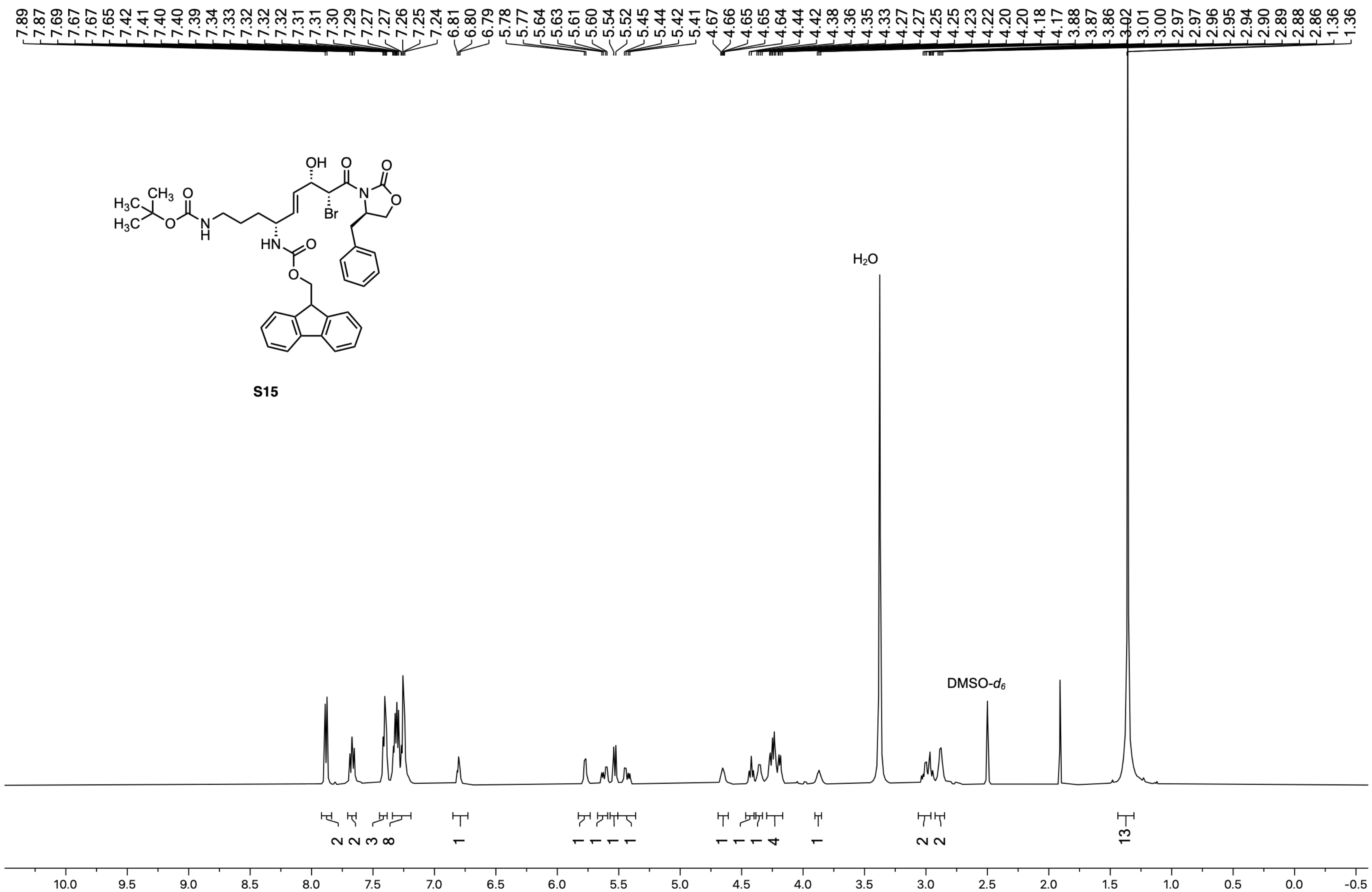

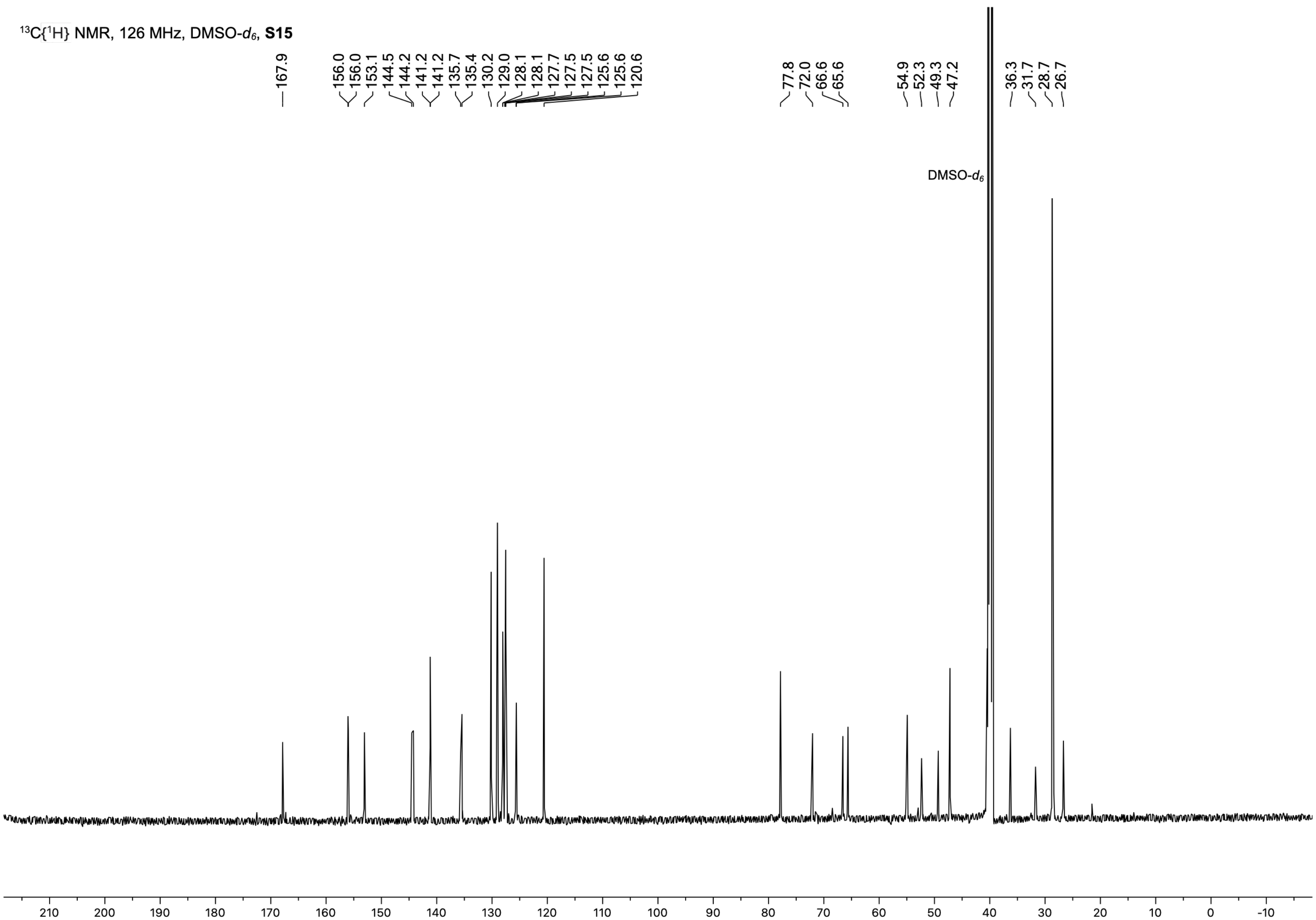

$^{13}C\{^1H\}$ NMR, 126 MHz, DMSO-d_6, **S15**
167.9
156.0
156.0
153.1
144.5
144.2
141.2
141.2
135.7
135.4
130.2
129.0
128.1
128.1
127.7
127.5
127.5
125.6
125.6
120.6
77.8
72.0
66.6
65.6
54.9
52.3
49.3
47.2
36.3
31.7
28.7
26.7
DMSO-d_6
210
200
190
180
170
160
150
140
130
120
110
100
90
80
70
60
50
40
30
20
10
0
-10

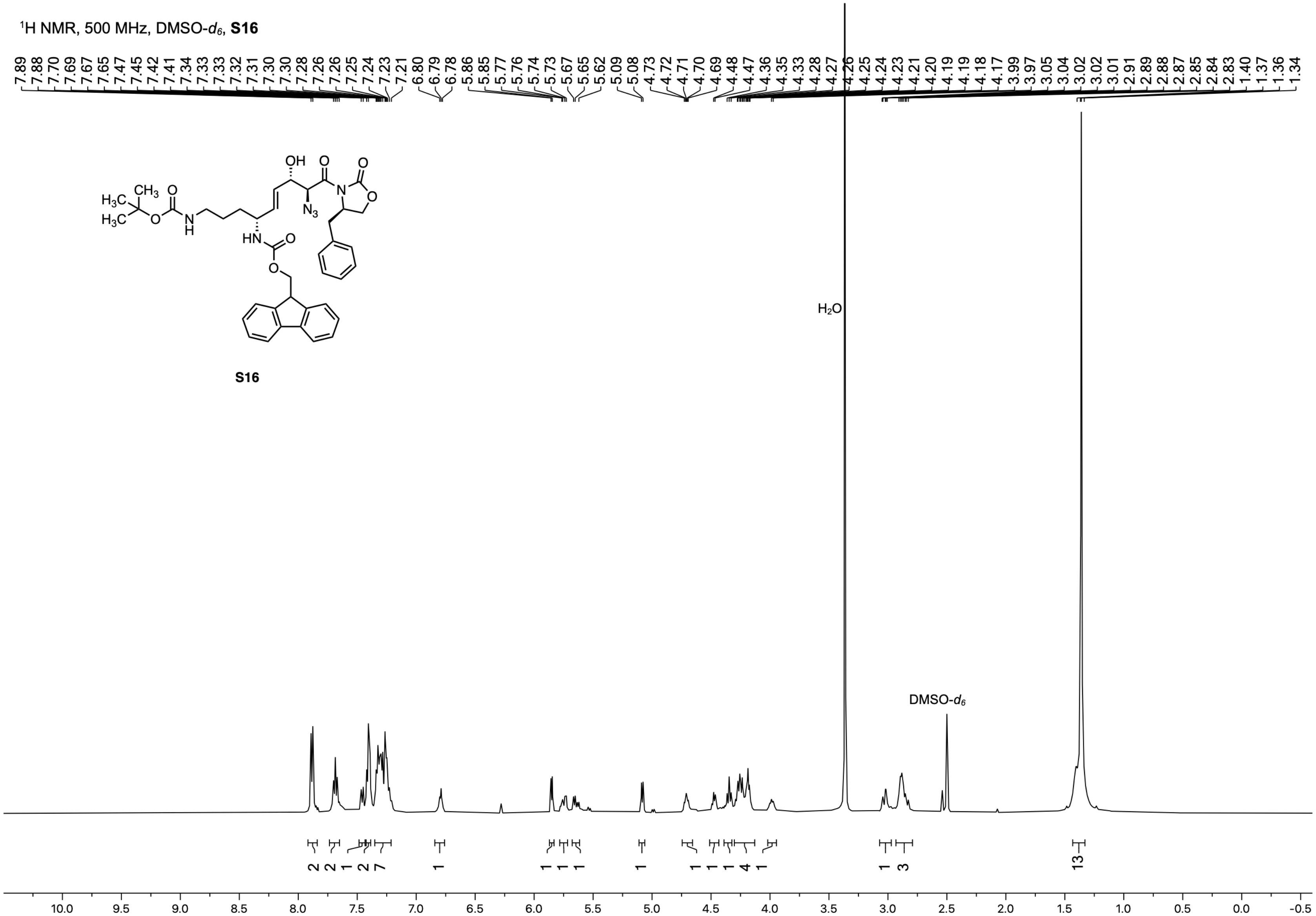

^{1}H NMR, 500 MHz, DMSO-d_6, **S16**
S16
H2O
DMSO-d6

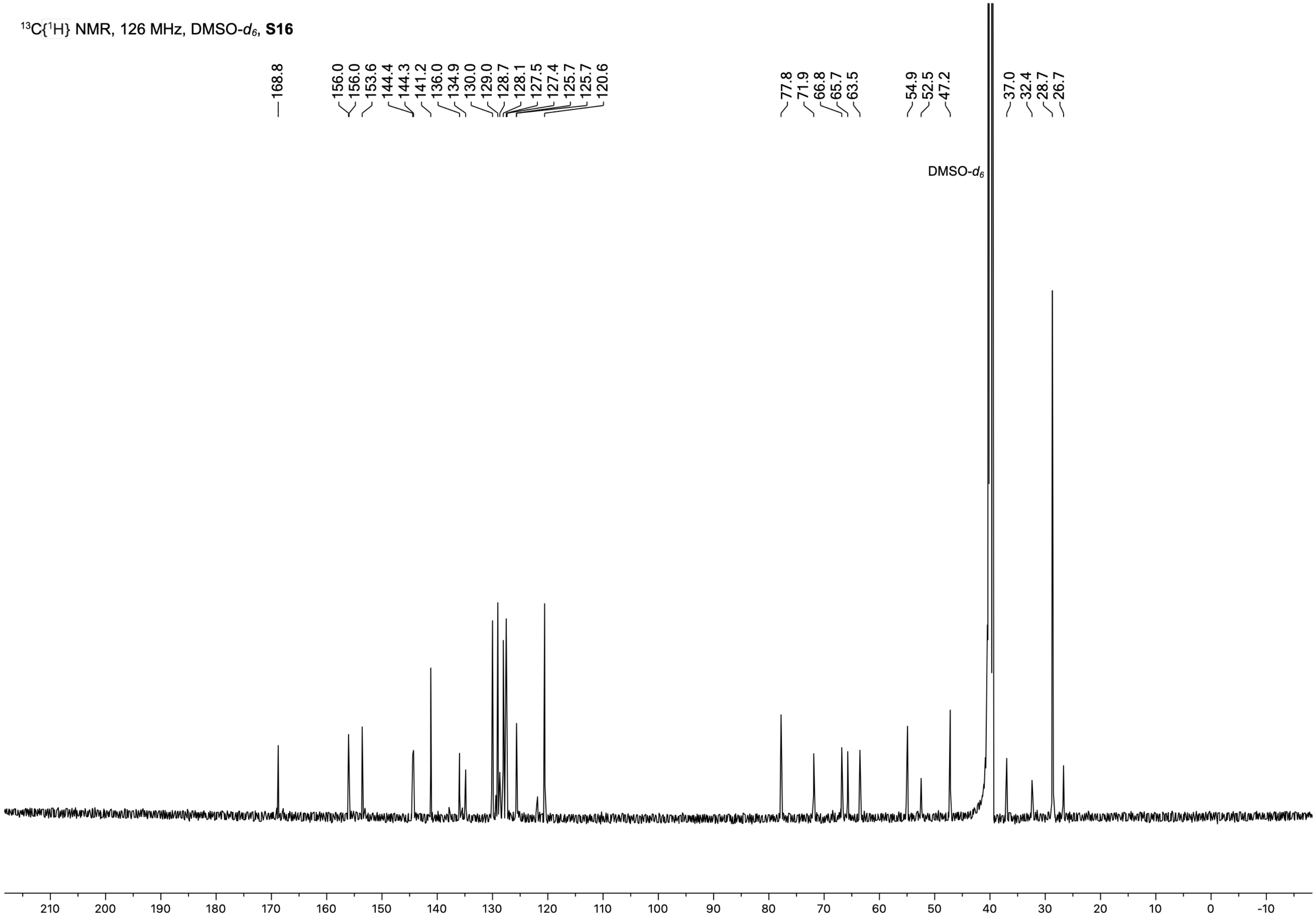

$^{13}C\{^1H\}$ NMR, 126 MHz, DMSO-d_6, **S16**
168.8
156.0
156.0
153.6
144.4
144.3
141.2
136.0
134.9
130.0
129.0
128.7
128.1
127.5
127.4
125.7
125.7
120.6
77.8
71.9
66.8
65.7
63.5
54.9
52.5
47.2
37.0
32.4
28.7
26.7
DMSO-d_6
210
200
190
180
170
160
150
140
130
120
110
100
90
80
70
60
50
40
30
20
10
0
-10

^{1}H NMR, 500 MHz, DMSO-d_6, **S17**

7.89, 7.88, 7.71, 7.69, 7.42, 7.40, 7.39, 7.34, 7.34, 7.34, 7.33, 7.33, 7.32, 7.32, 7.31, 7.31, 7.31, 7.30, 6.81, 6.80, 6.78, 5.61, 5.60, 5.58, 5.57, 5.57, 5.56, 5.55, 5.54, 5.53, 4.29, 4.28, 4.27, 4.25, 4.25, 4.24, 4.22, 4.20, 4.19, 4.11, 4.10, 4.09, 3.92, 3.91, 3.90, 3.40, 3.38, 3.36, 2.90, 2.89, 2.87, 2.86, 1.38, 1.36, 1.34, 1.34

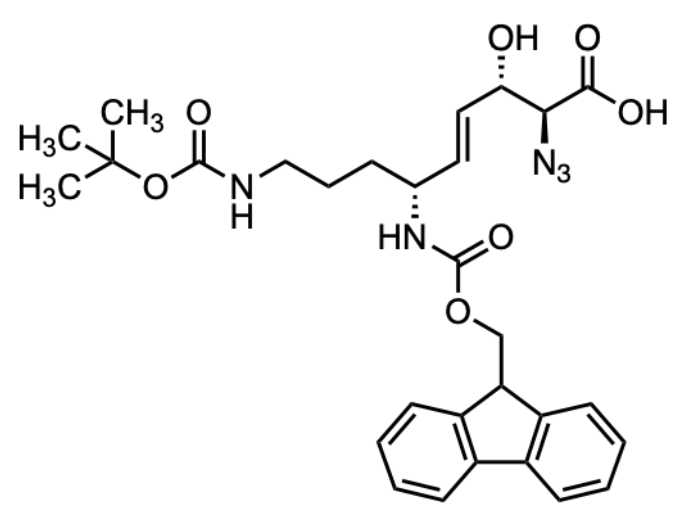

S17

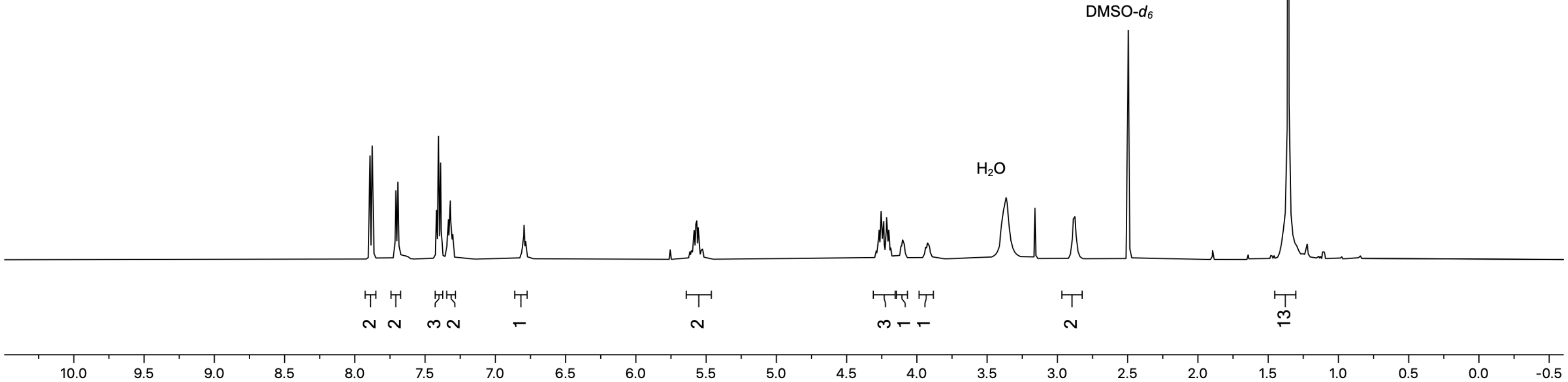

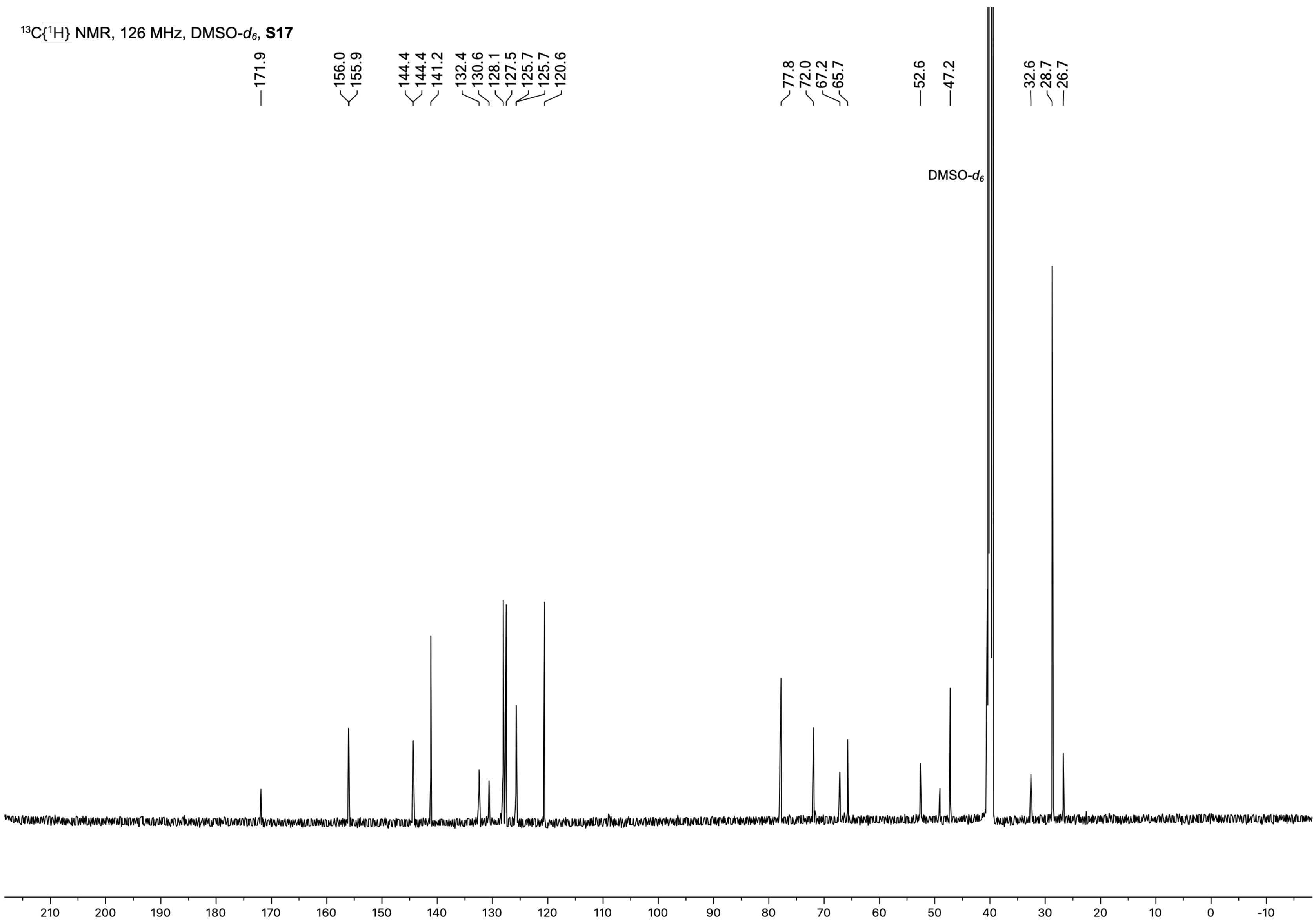

$^{13}C\{^{1}H\}$ NMR, 126 MHz, DMSO-d_6, **S17**
171.9
156.0
155.9
144.4
144.4
141.2
132.4
130.6
128.1
127.5
125.7
125.7
120.6
77.8
72.0
67.2
65.7
52.6
47.2
32.6
28.7
26.7
DMSO-d_6
210 200 190 180 170 160 150 140 130 120 110 100 90 80 70 60 50 40 30 20 10 0 -10

^{1}H NMR, 500 MHz, DMSO-d_6, **S18**

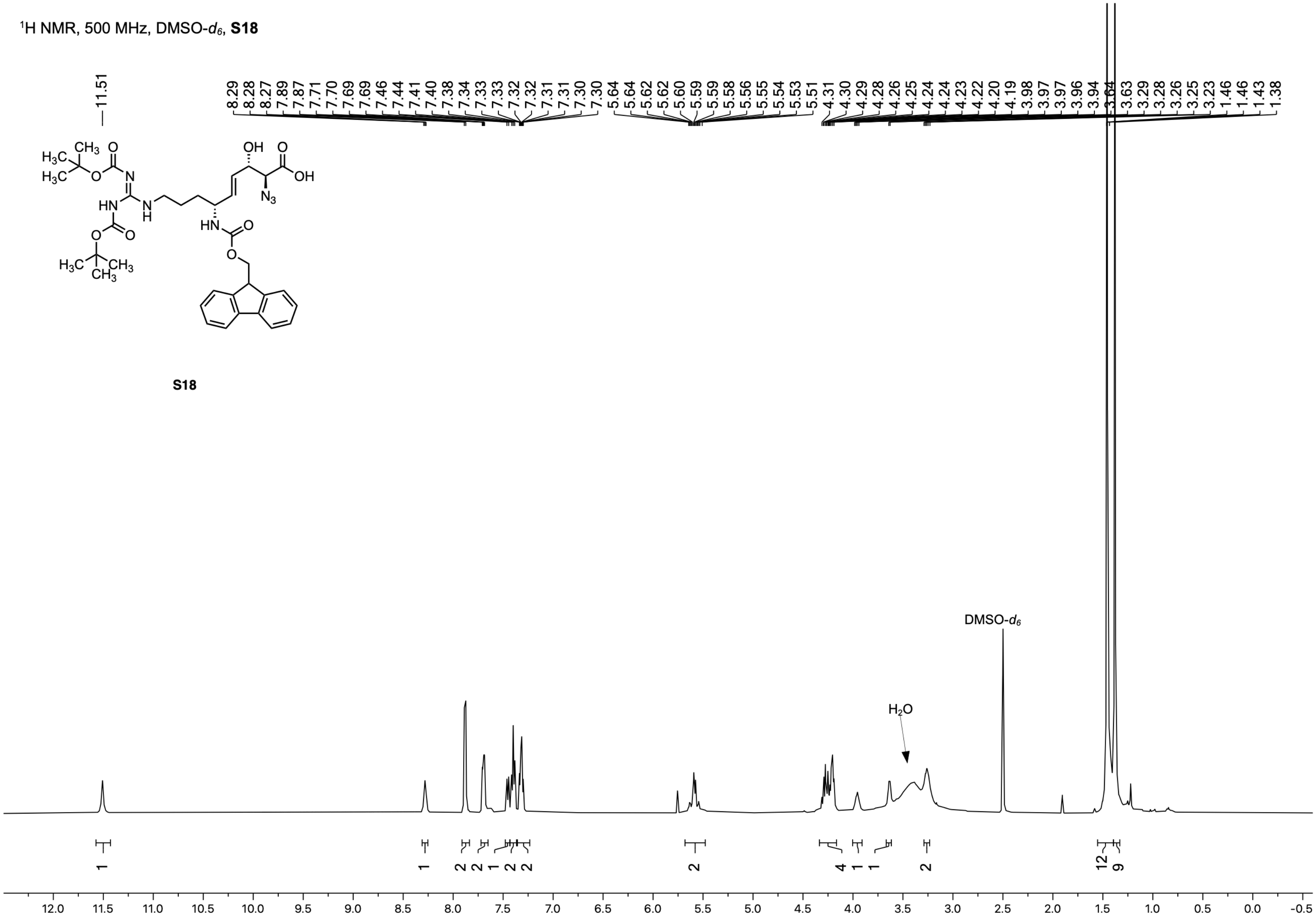

S18

$^{13}C\{^1H\}$ NMR, 126 MHz, DMSO-d_6, **S18**

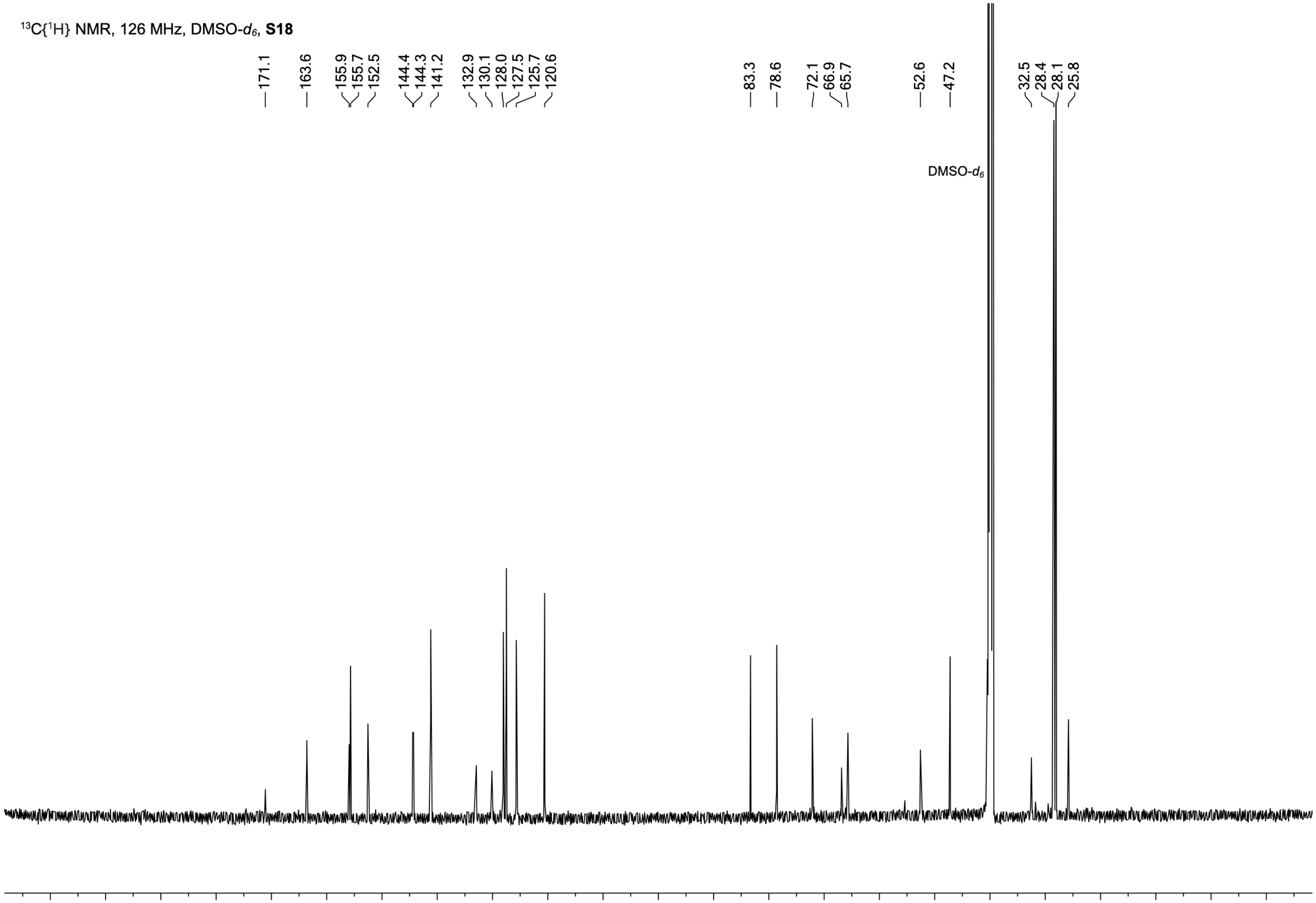

^{1}H NMR, 500 MHz, DMSO-d_6, **19**

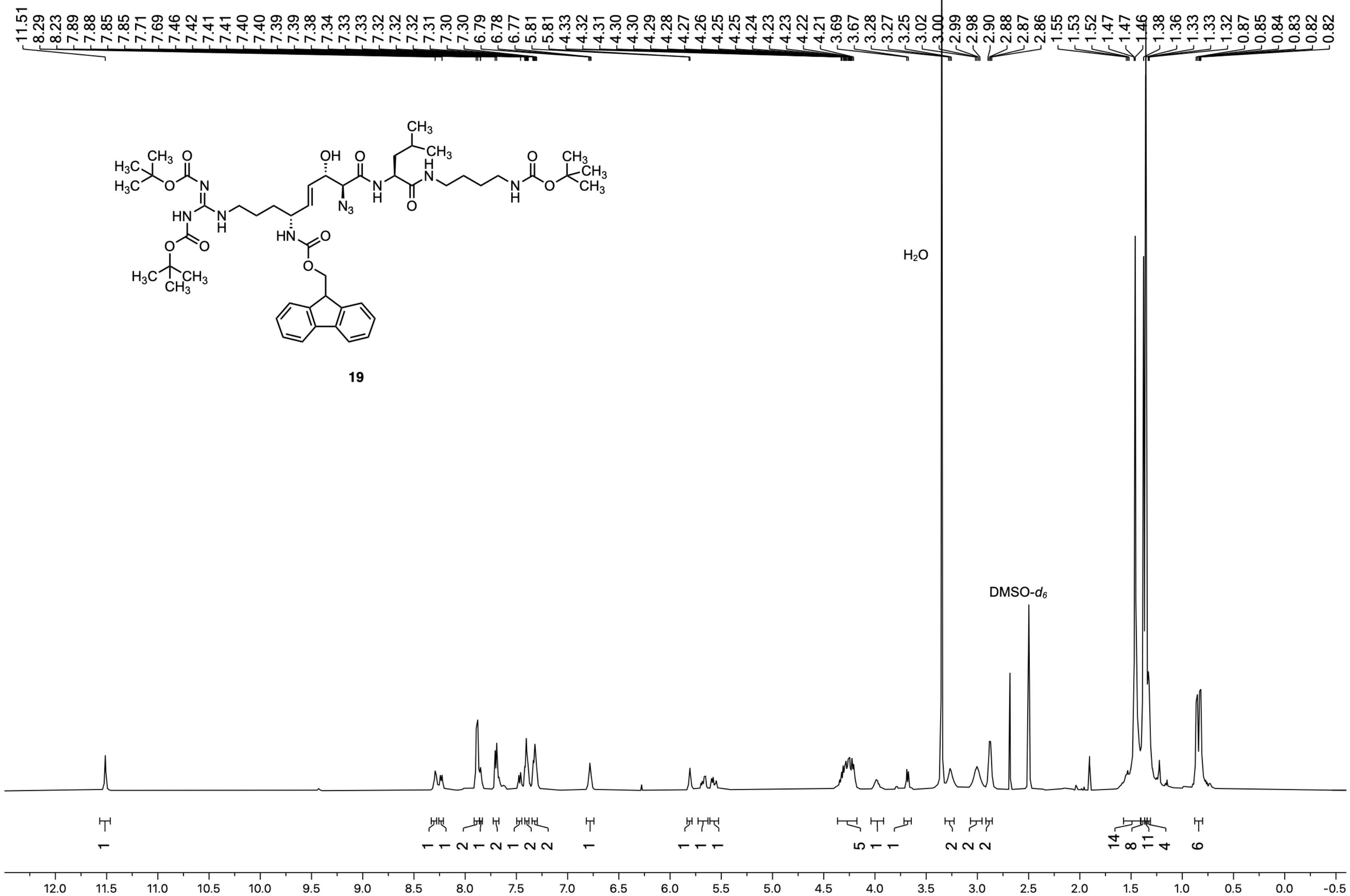

COSY, DMSO-d_6, **19**

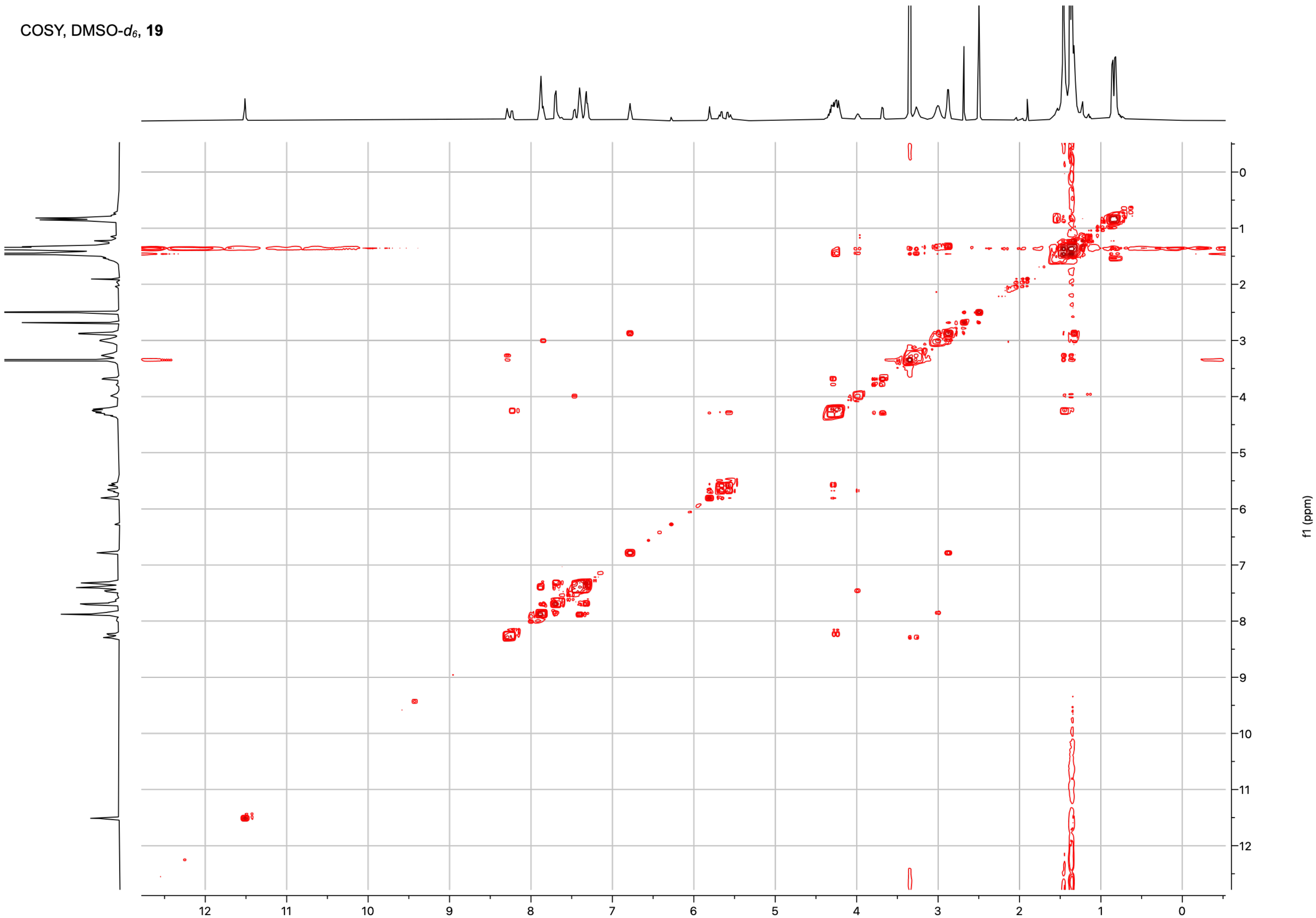

$^{13}C\{^1H\}$ NMR, 126 MHz, DMSO-d_6, **19**

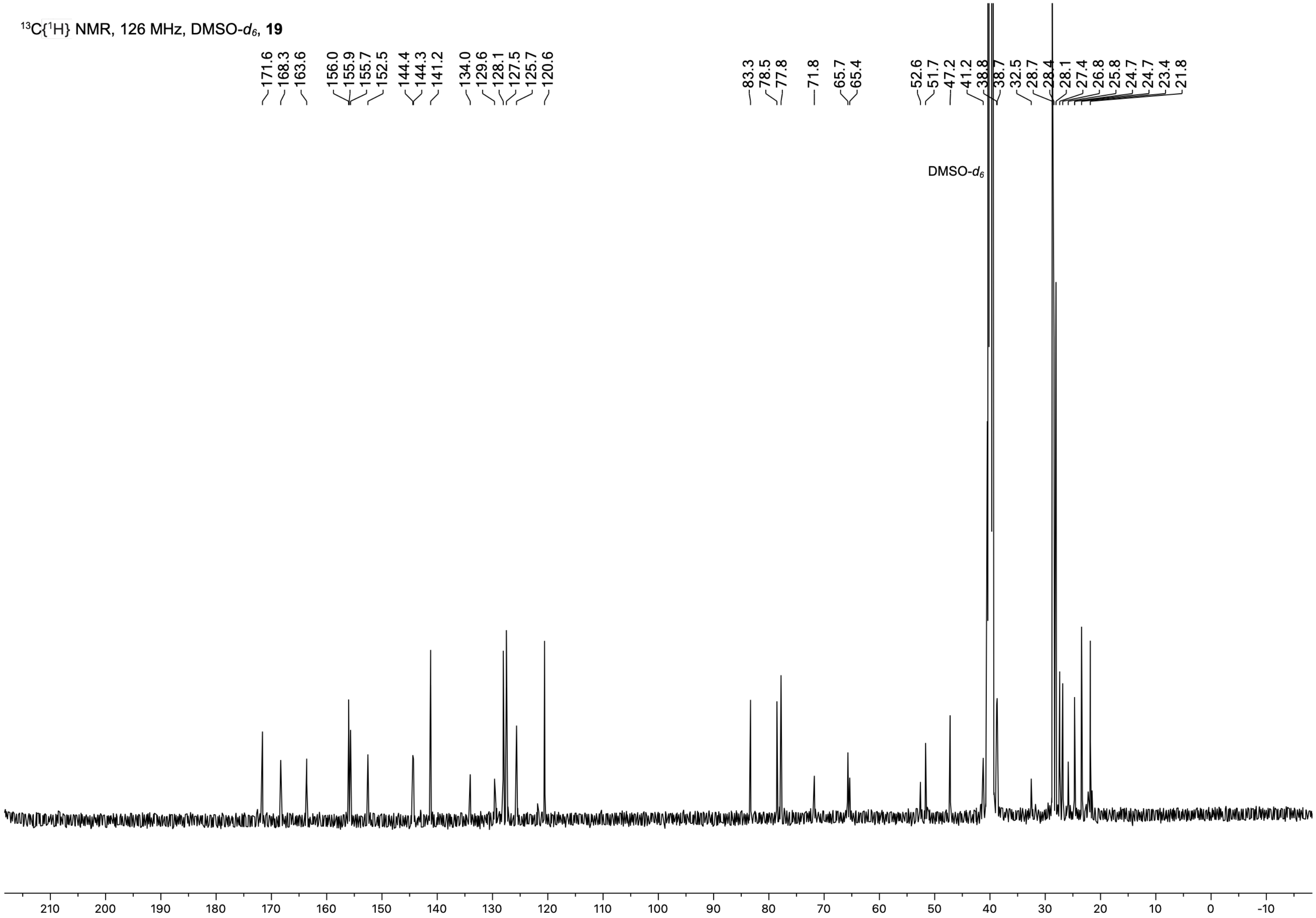

HSQC, DMSO-d_6, **19**

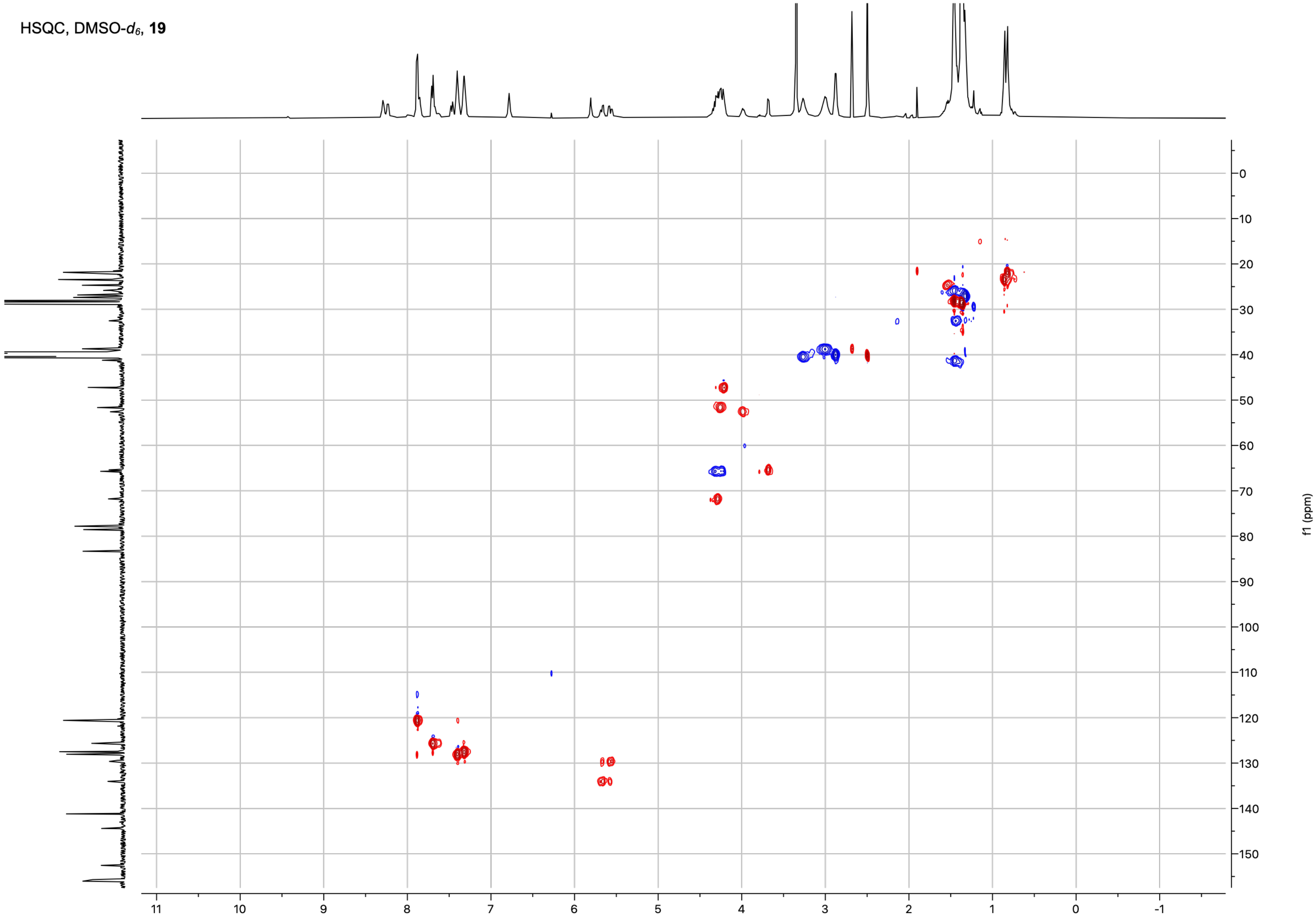

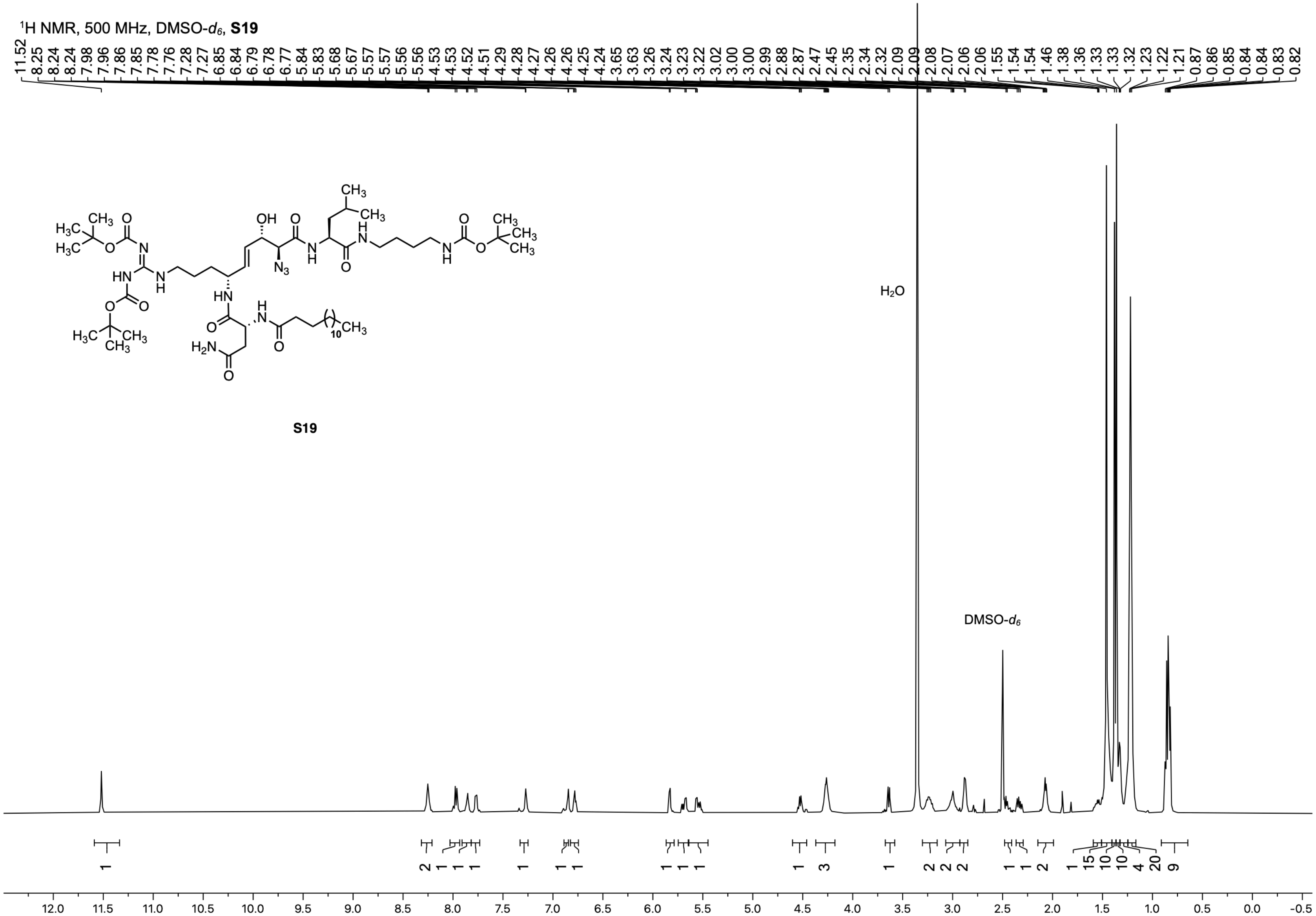

1H NMR, 500 MHz, DMSO-d6, S19
11.52 8.25 8.24 8.24 7.98 7.96 7.86 7.85 7.78 7.76 7.28 7.27 6.85 6.84 6.79 6.78 6.77 5.84 5.83 5.68 5.67 5.57 5.57 5.56 5.56 4.53 4.53 4.52 4.51 4.29 4.28 4.27 4.26 4.26 4.25 4.24 3.65 3.63 3.26 3.24 3.23 3.22 3.02 3.00 3.00 2.99 2.88 2.87 2.47 2.45 2.35 2.34 2.32 2.09 2.09 2.08 2.07 2.06 2.06 1.55 1.54 1.54 1.46 1.38 1.36 1.33 1.33 1.32 1.23 1.22 1.21 0.87 0.86 0.85 0.84 0.84 0.83 0.82
S19
H2O
DMSO-d6
1 2 1 1 1 1 1 1 1 1 1 1 3 1 2 2 2 1 1 2 1 15 10 10 4 20 9
12.0 11.5 11.0 10.5 10.0 9.5 9.0 8.5 8.0 7.5 7.0 6.5 6.0 5.5 5.0 4.5 4.0 3.5 3.0 2.5 2.0 1.5 1.0 0.5 0.0 -0.5

COSY, DMSO-d_6, **S19**

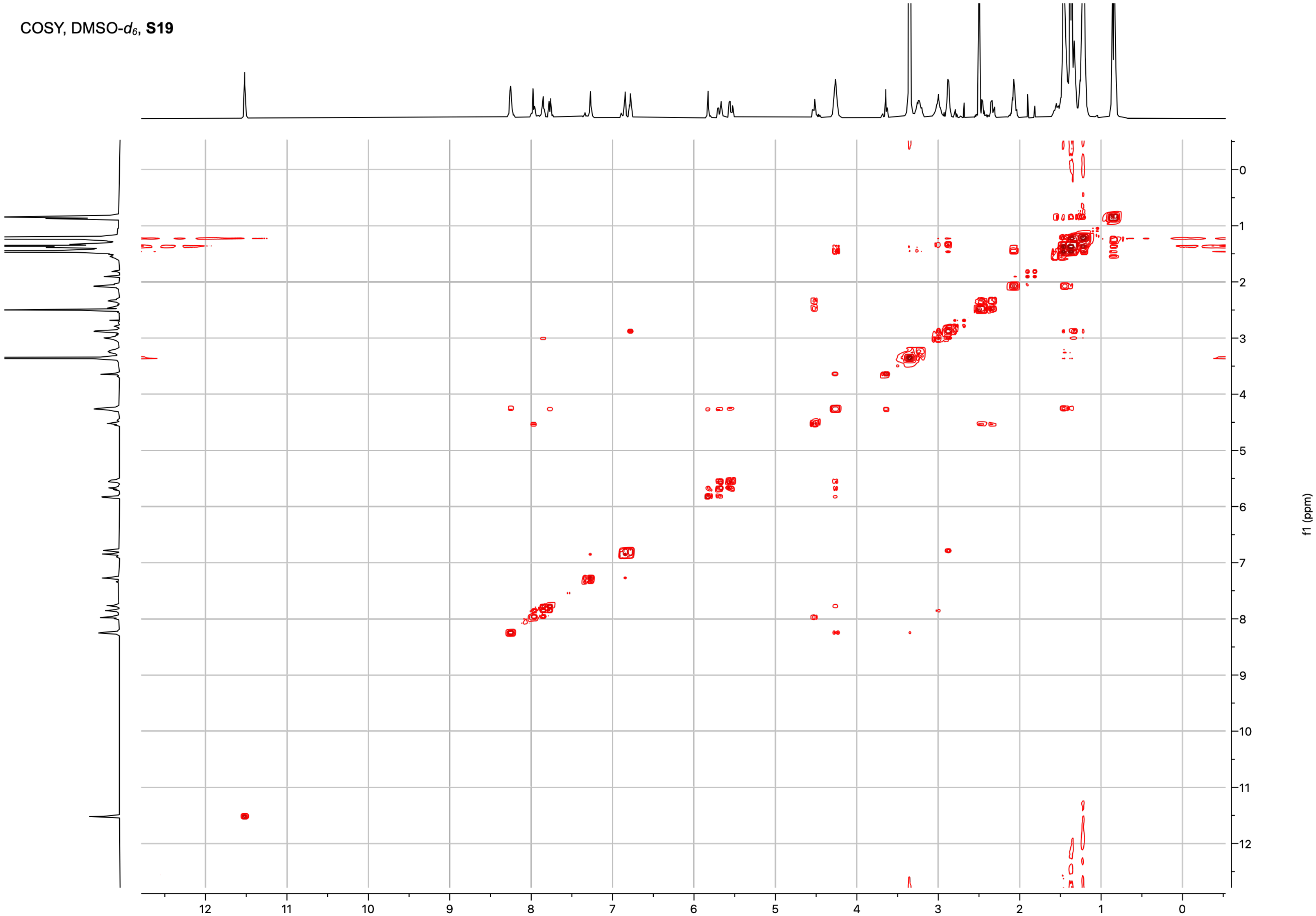

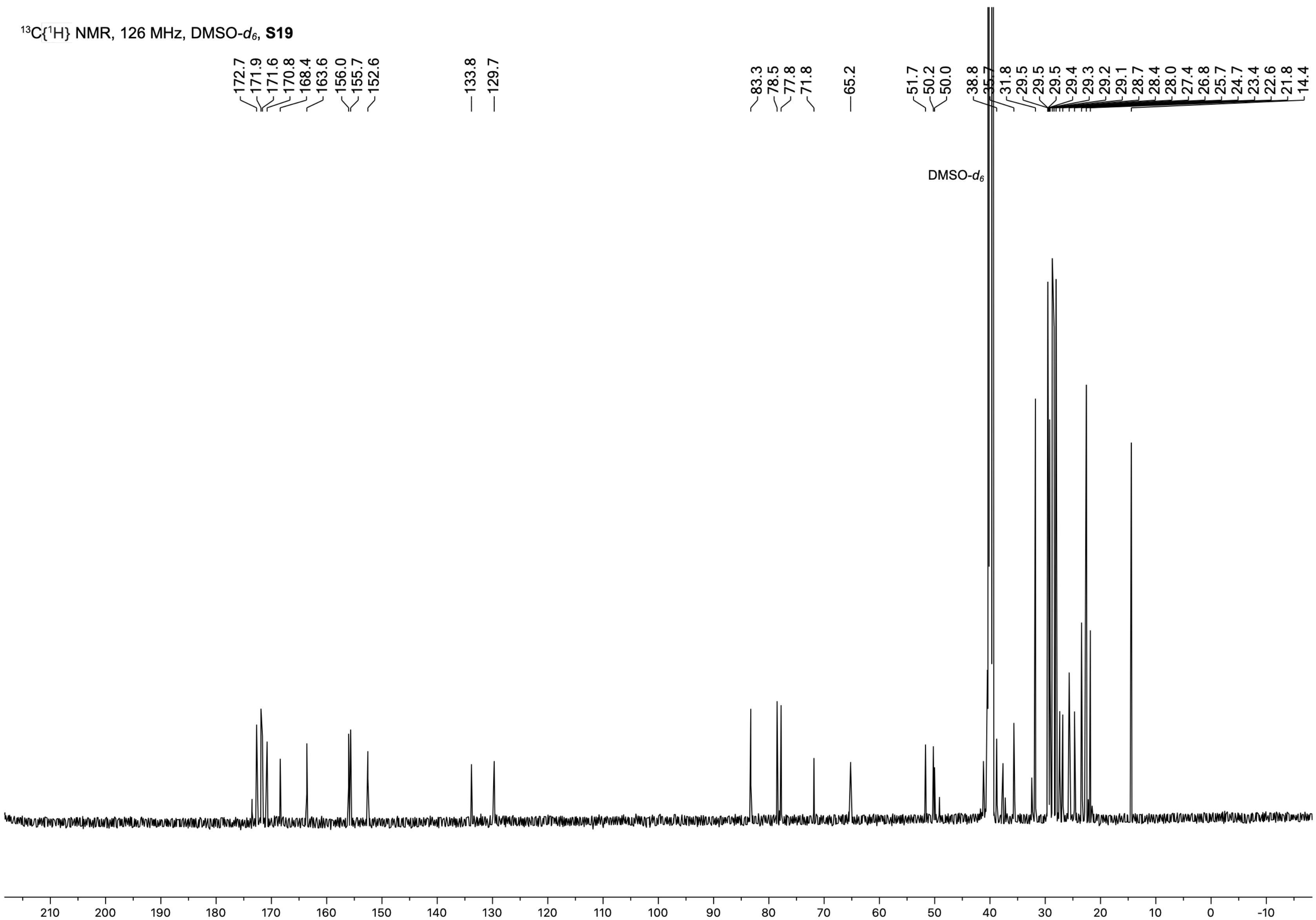

$^{13}C\{^1H\}$ NMR, 126 MHz, DMSO-d_6, **S19**
172.7
171.9
171.6
170.8
168.4
163.6
156.0
155.7
152.6
133.8
129.7
83.3
78.5
77.8
71.8
65.2
51.7
50.2
50.0
38.8
35.7
31.8
29.5
29.5
29.4
29.3
29.2
29.1
28.7
28.4
28.0
27.4
26.8
25.7
24.7
23.4
22.6
21.8
14.4
DMSO-d_6

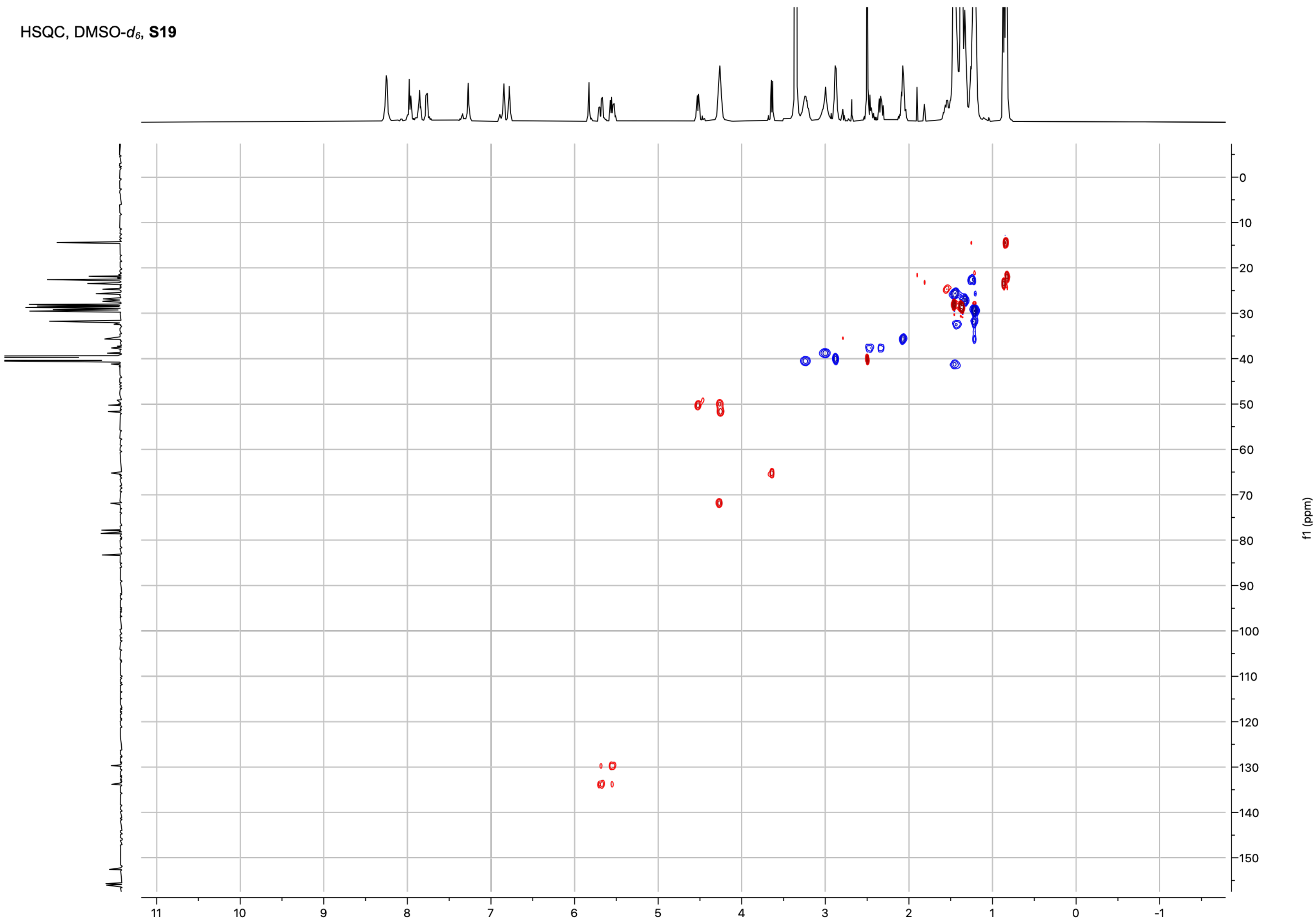
HSQC, DMSO-d_6, **S19**
f1 (ppm)

^{1}H NMR, 500 MHz, DMSO-d_6, **S20**

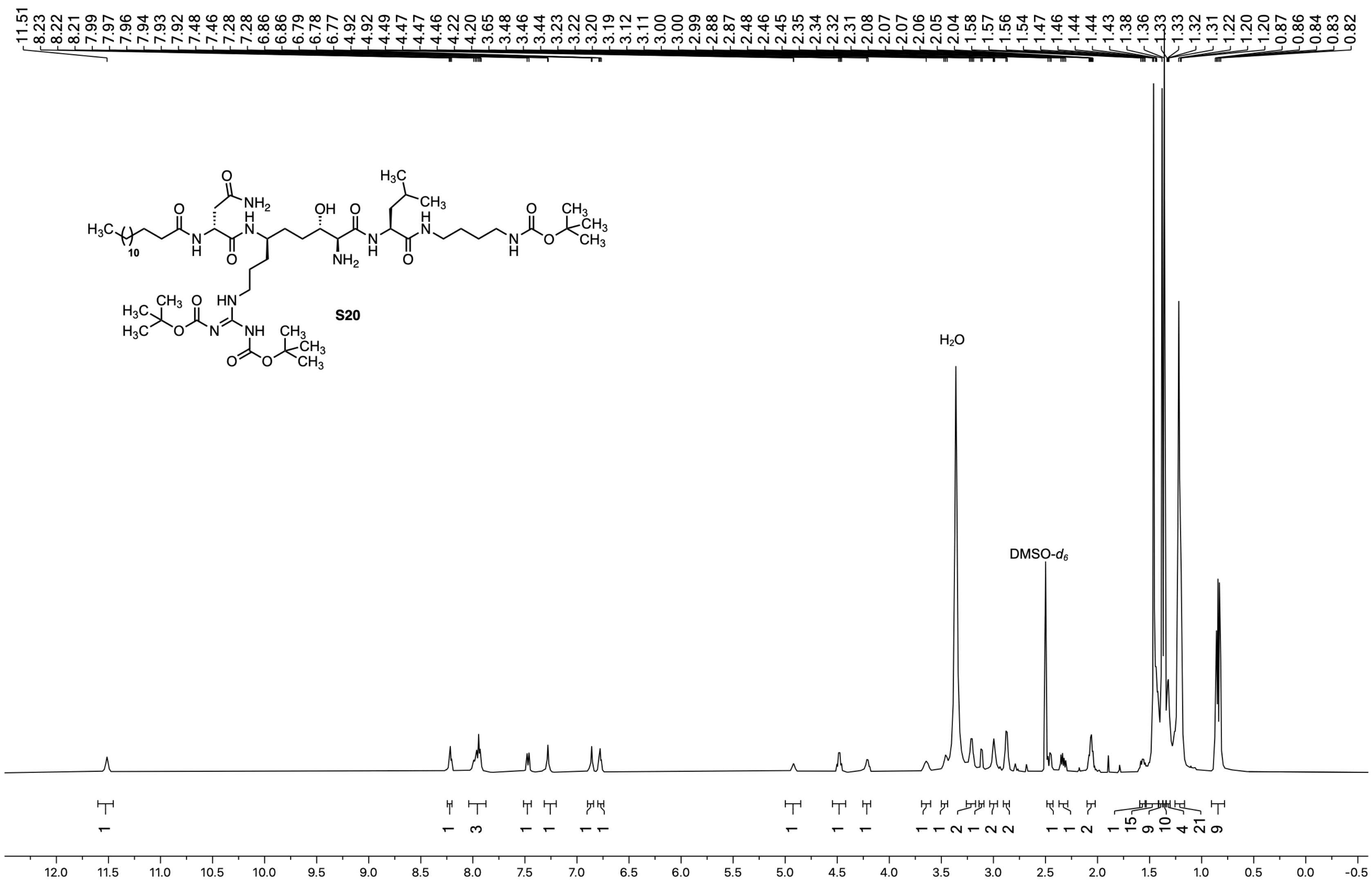

COSY, DMSO-d_6, **S20**

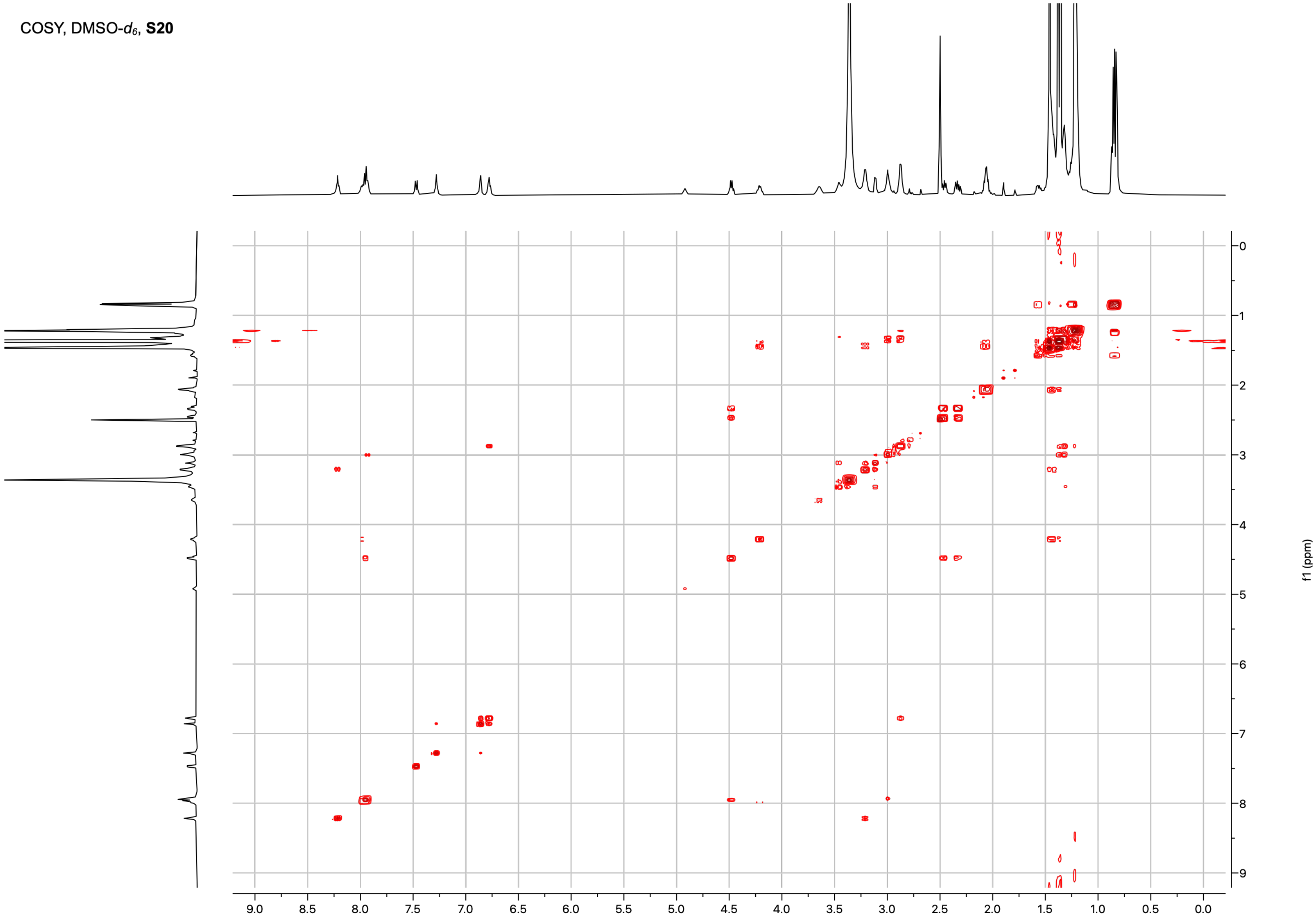

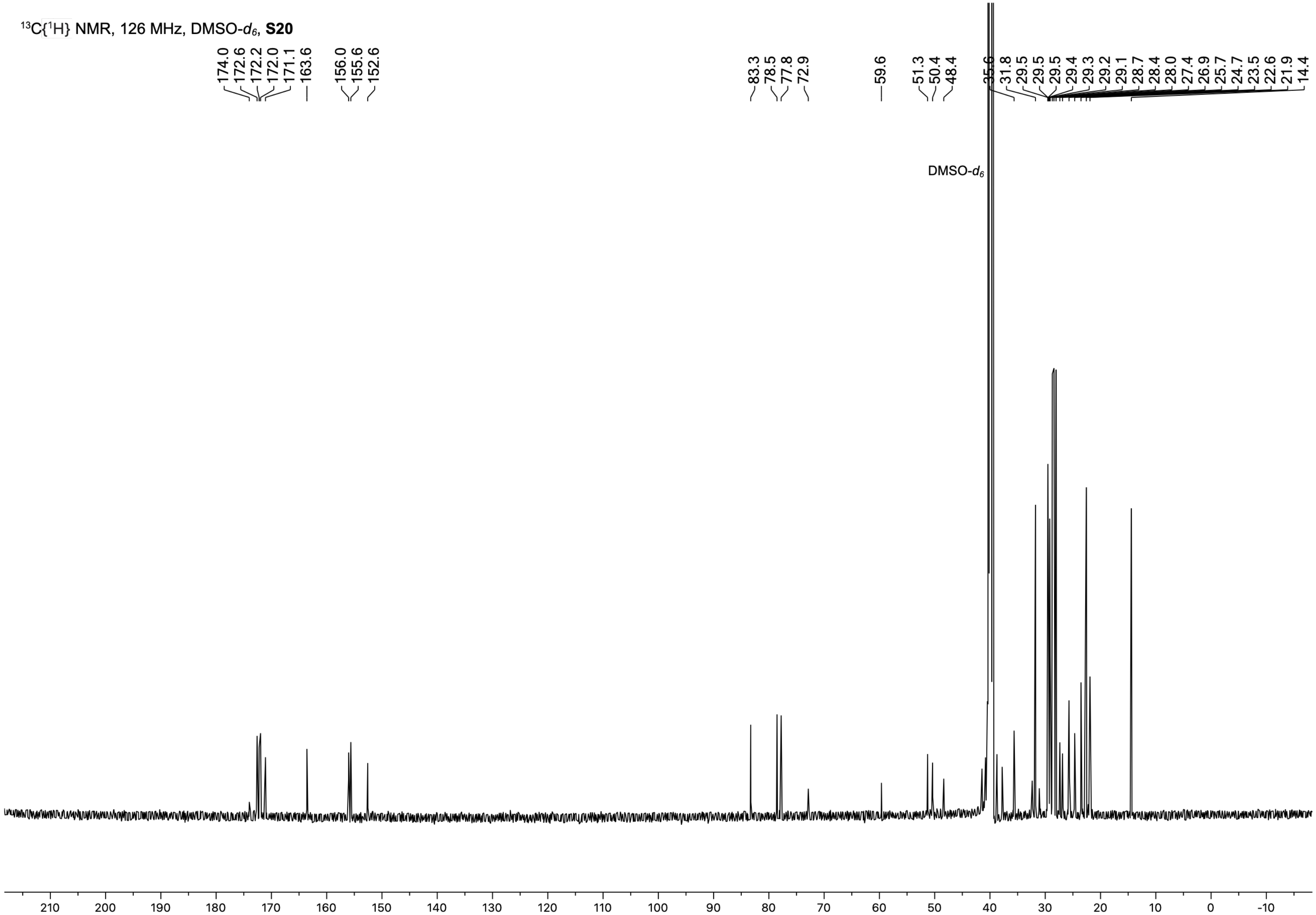

$^{13}C\{^1H\}$ NMR, 126 MHz, DMSO-d_6, **S20**
174.0
172.6
172.2
172.0
171.1
163.6
156.0
155.6
152.6
83.3
78.5
77.8
72.9
59.6
51.3
50.4
48.4
35.6
31.8
29.5
29.5
29.5
29.4
29.3
29.2
29.1
28.7
28.4
28.0
27.4
26.9
25.7
24.7
23.5
22.6
21.9
14.4
DMSO-d_6
210 200 190 180 170 160 150 140 130 120 110 100 90 80 70 60 50 40 30 20 10 0 -10

HSQC, DMSO-d_6, **S20**

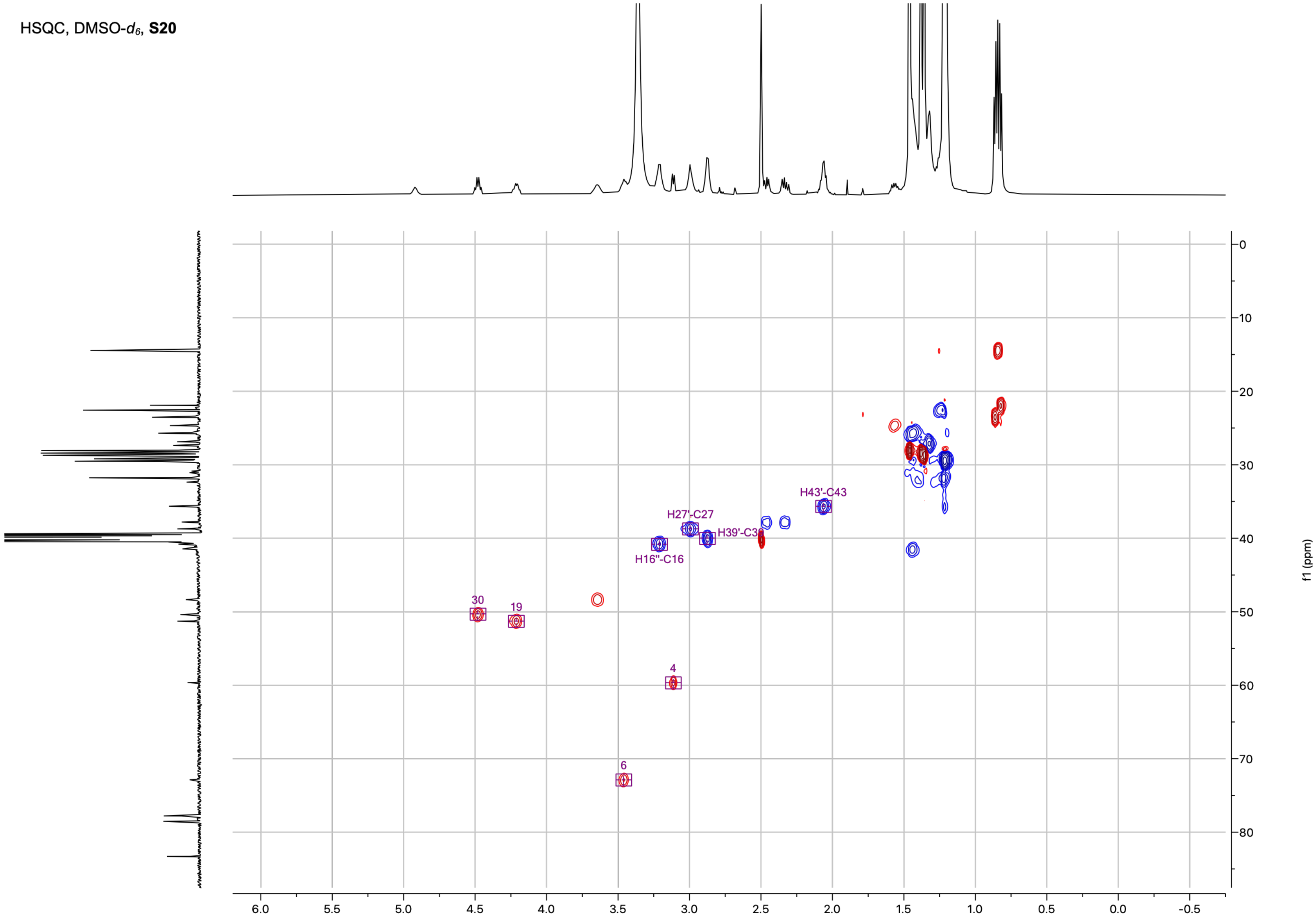

^{1}H NMR, 600 MHz, DMF-d_7, **S21**

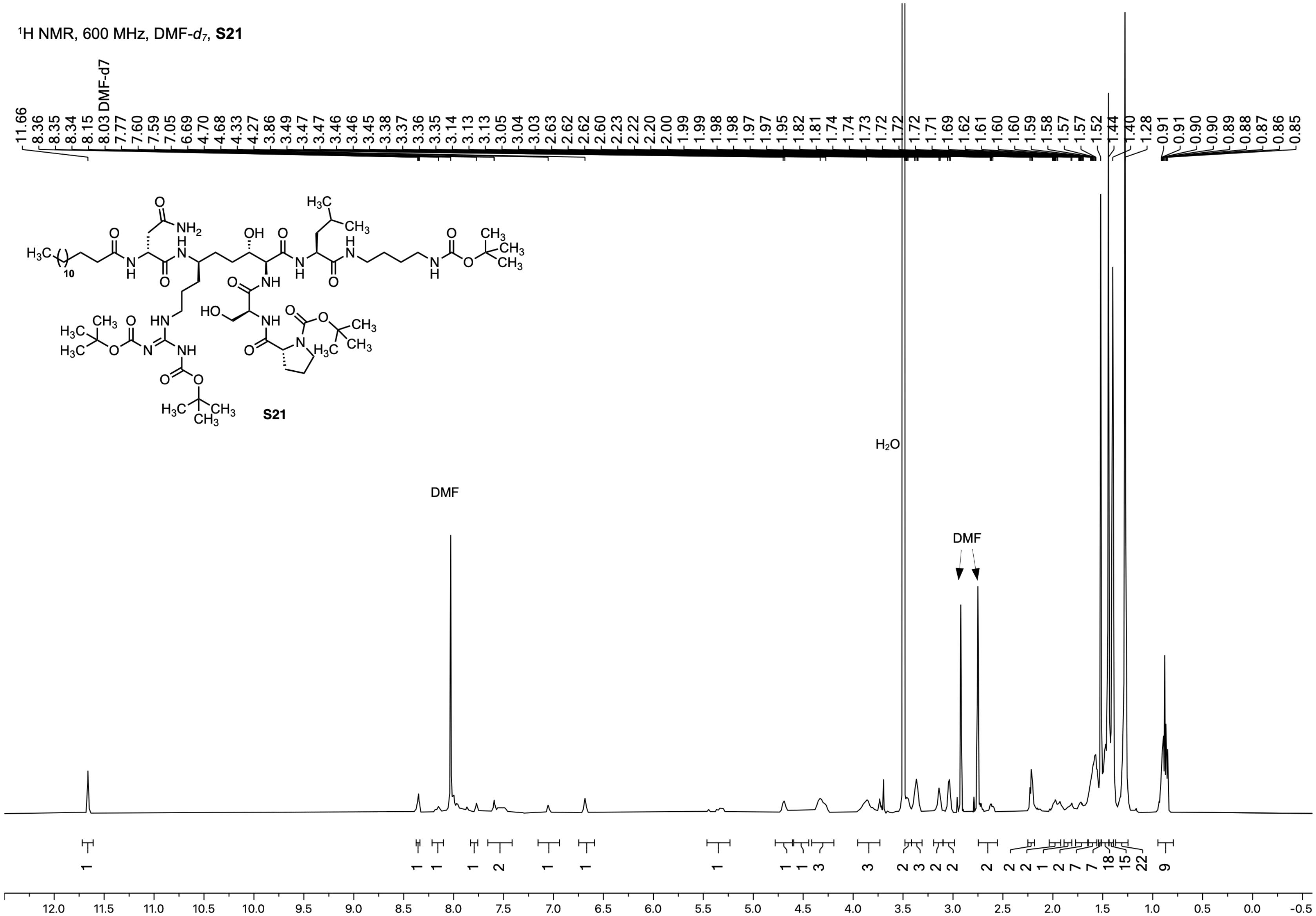

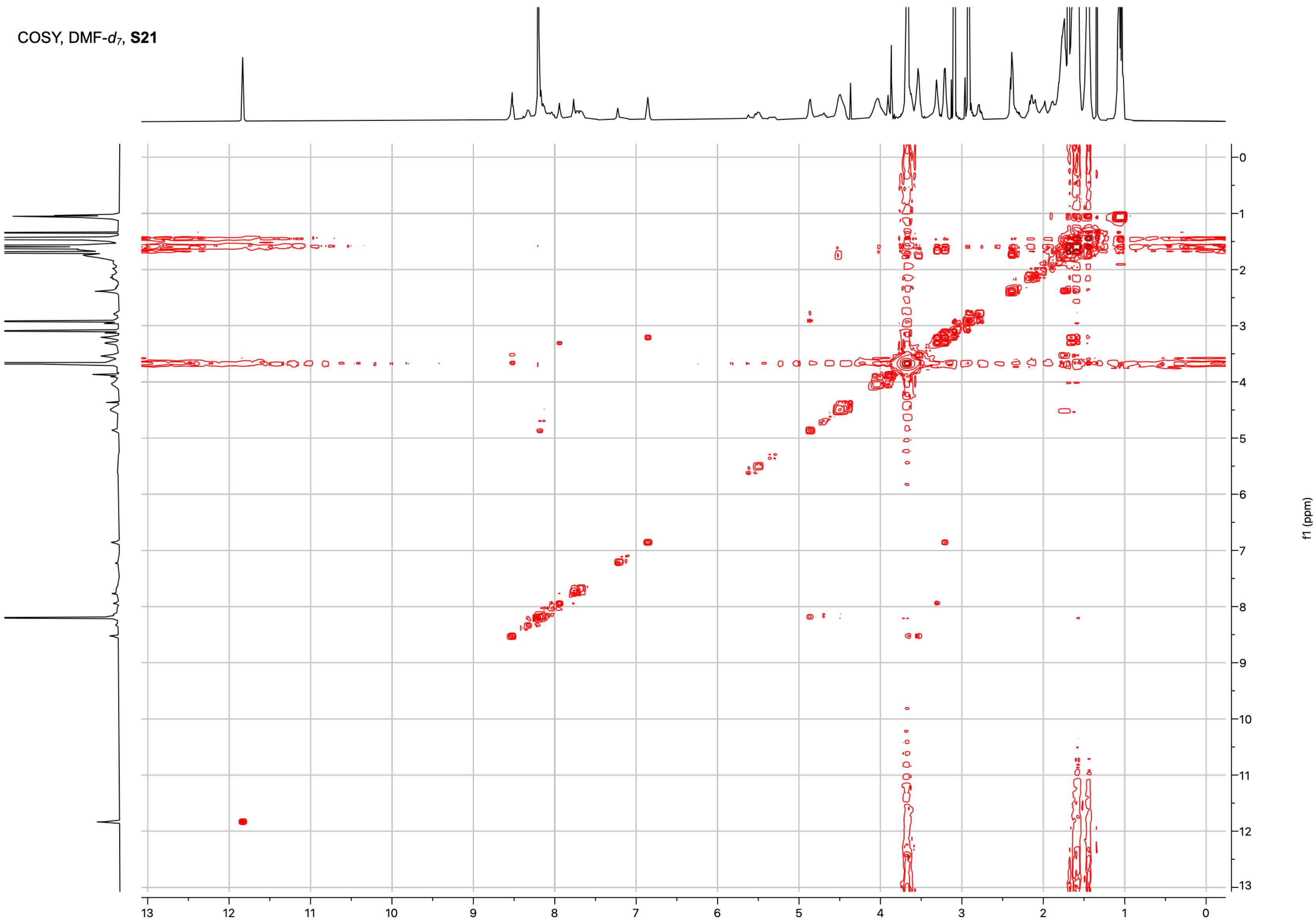
COSY, DMF-d_7, **S21**
f1 (ppm)
13
12
11
10
9
8
7
6
5
4
3
2
1
0

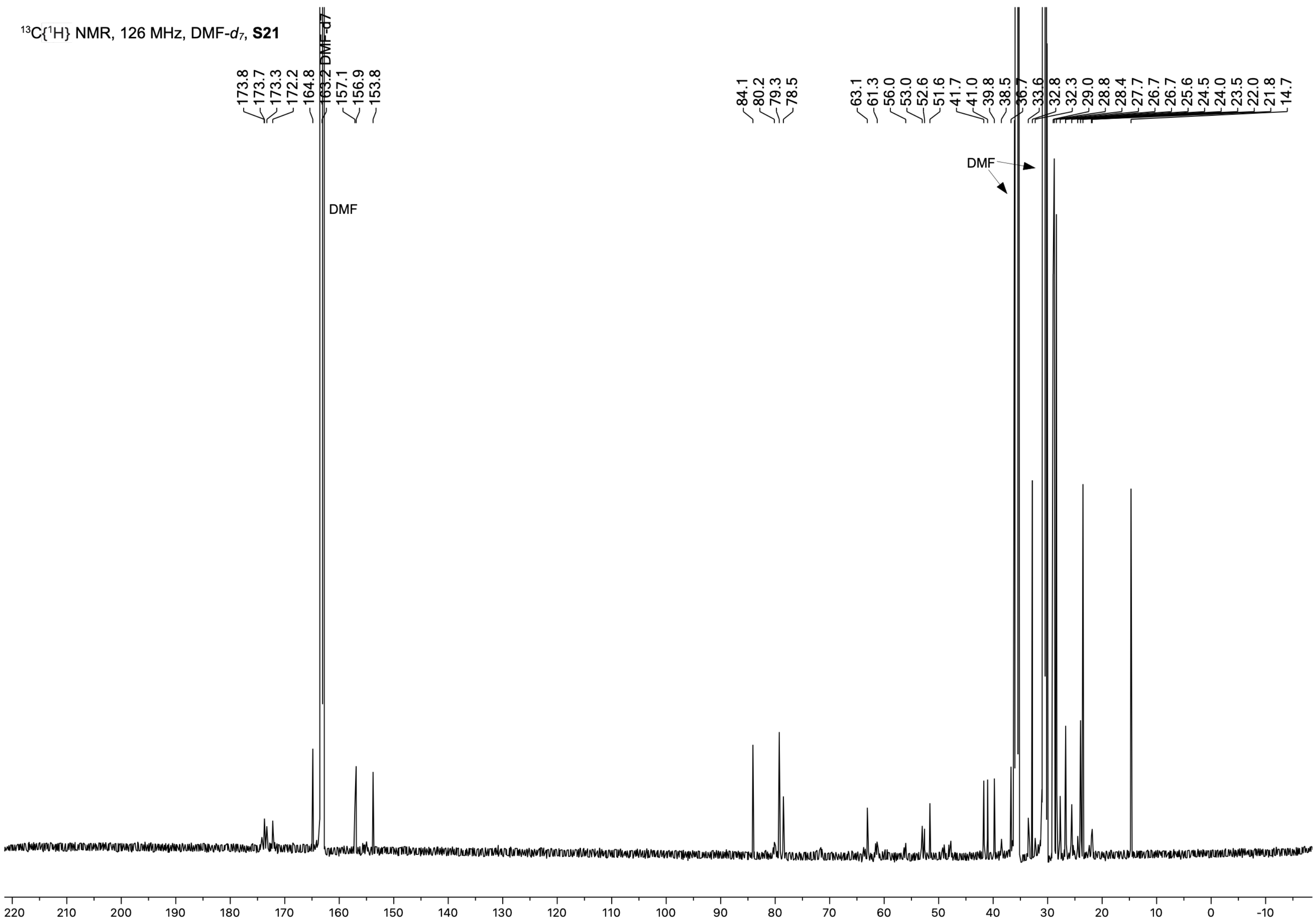

$^{13}C\{^{1}H\}$ NMR, 126 MHz, DMF-d_7, **S21**
173.8
173.7
173.3
172.2
164.8
163.2
DMF-d7
157.1
156.9
153.8
84.1
80.2
79.3
78.5
63.1
61.3
56.0
53.0
52.6
51.6
41.7
41.0
39.8
38.5
36.7
33.6
32.8
32.3
29.0
28.8
28.4
27.7
26.7
26.7
25.6
24.5
24.0
23.5
22.0
21.8
14.7
DMF
DMF
220
210
200
190
180
170
160
150
140
130
120
110
100
90
80
70
60
50
40
30
20
10
0
-10

HSQC, DMF-d_7, **S21**

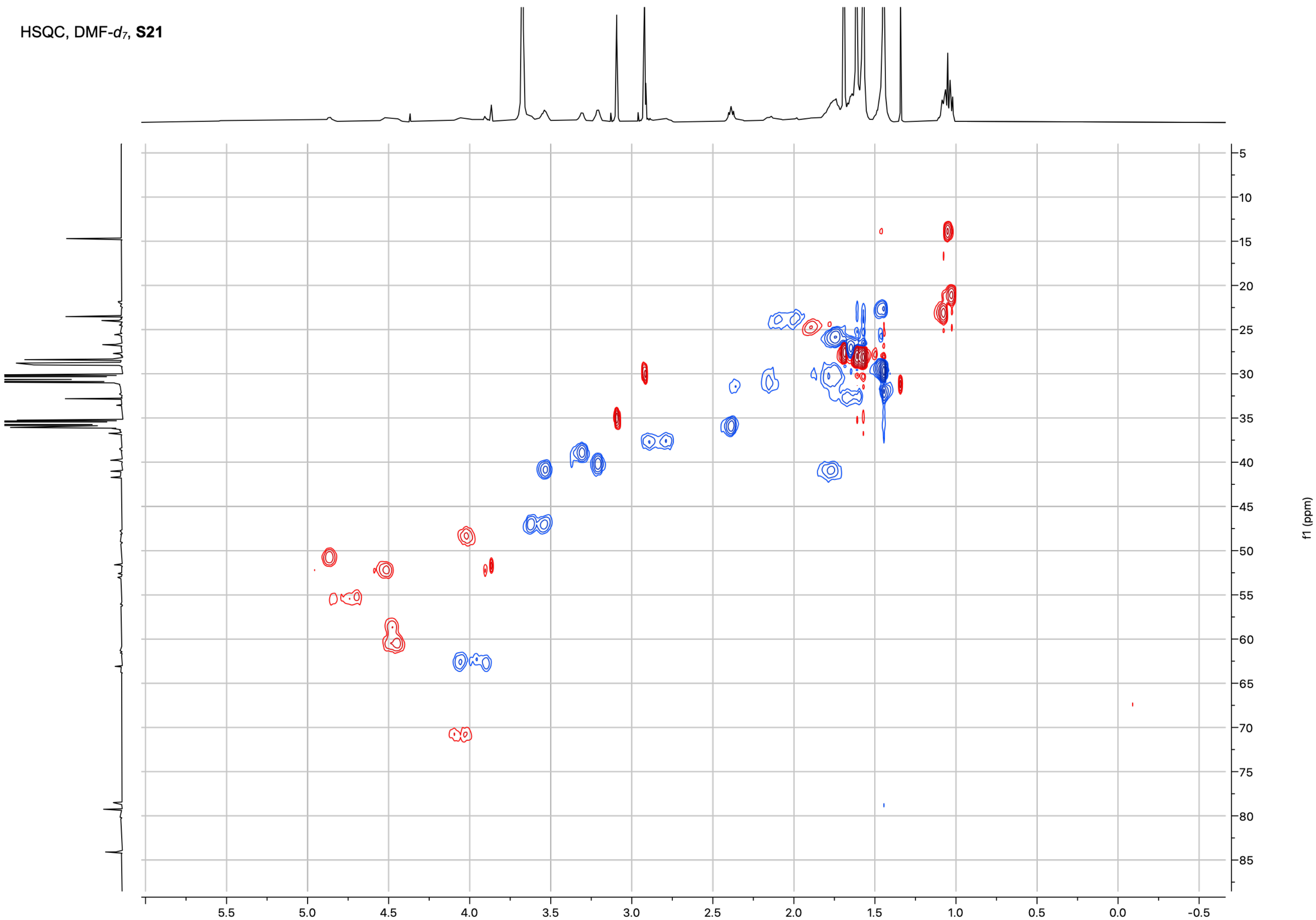

^{1}H NMR, 500 MHz, DMSO-d_6, (5*R*)-**1**

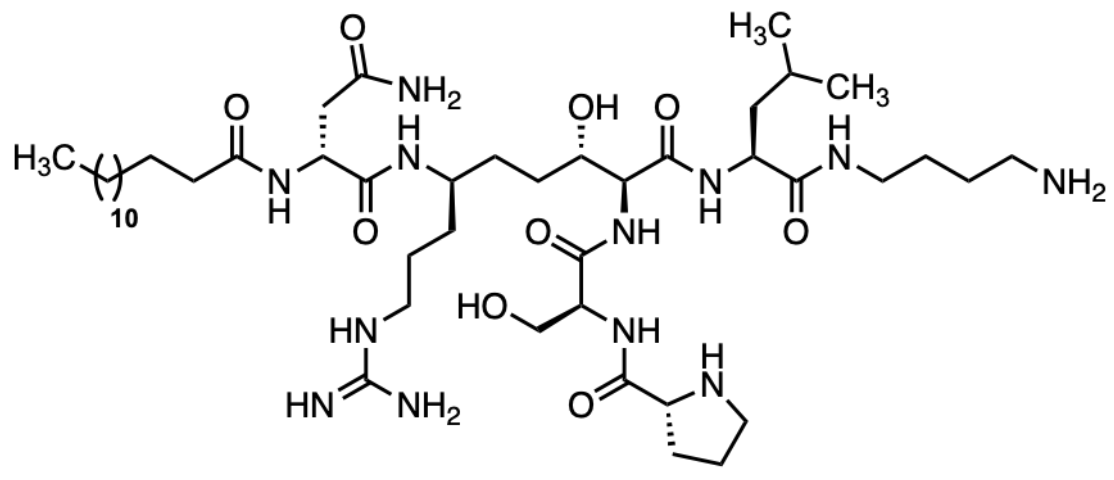

revised pre-rhabdobranin B
(5*R*)-**1**

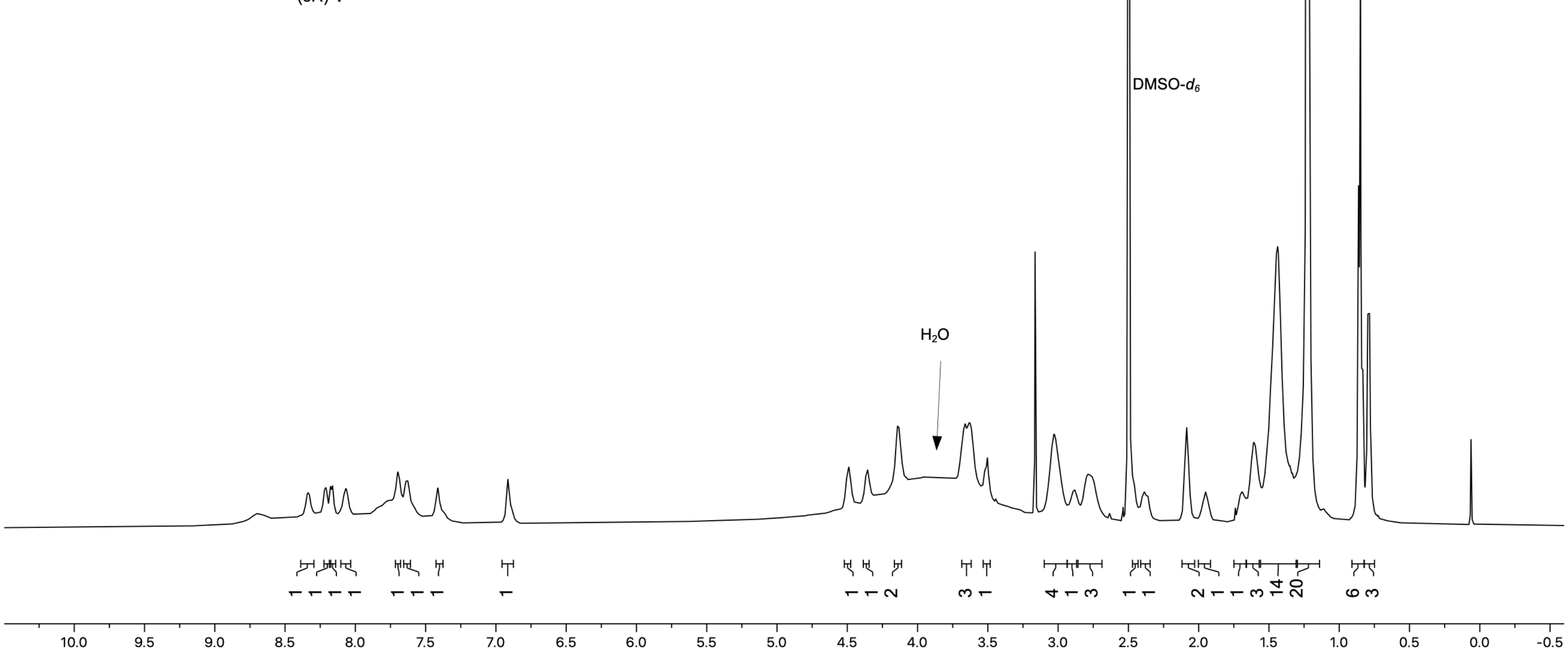

COSY, DMSO-d_6, (5*R*)-**1**

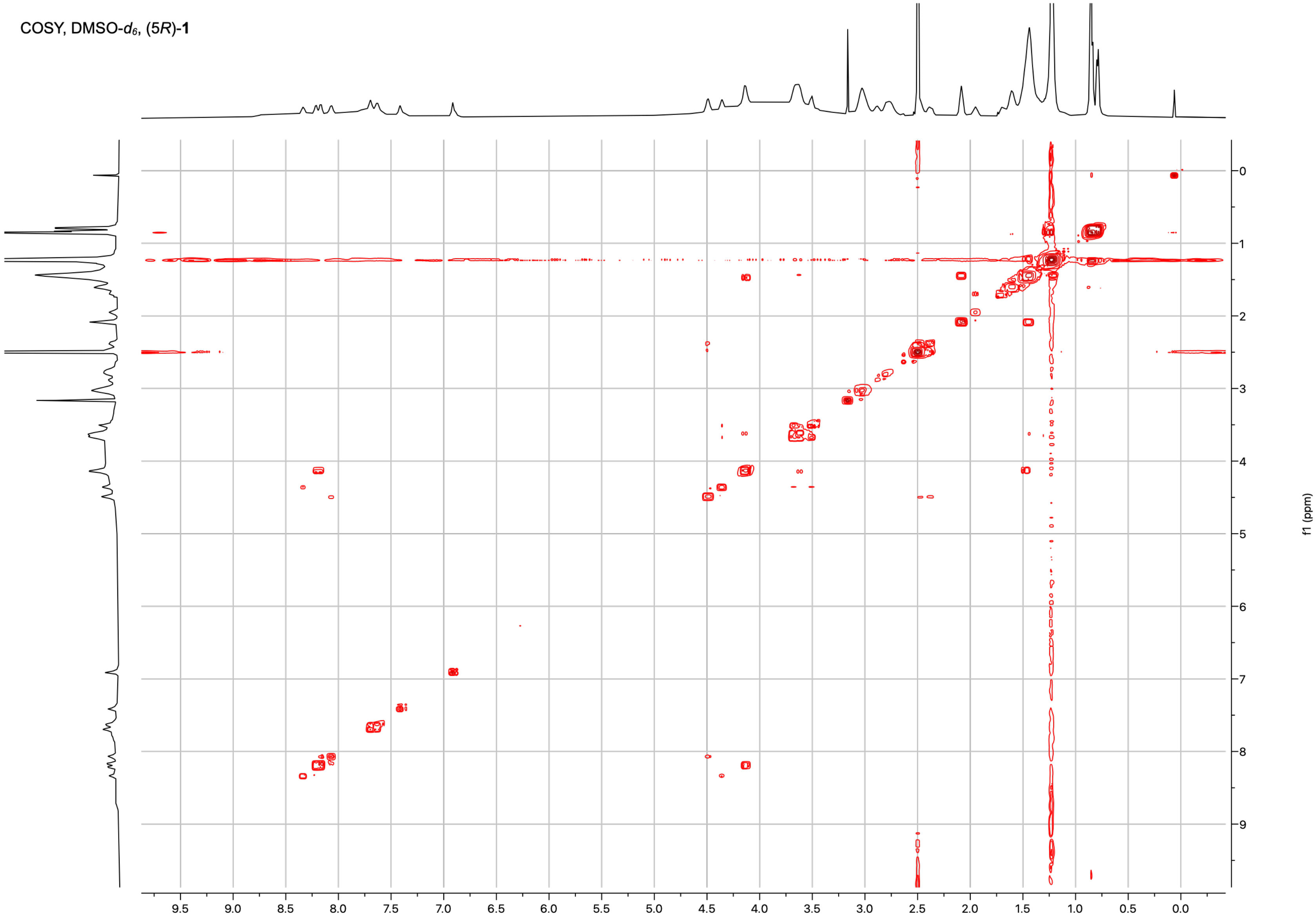

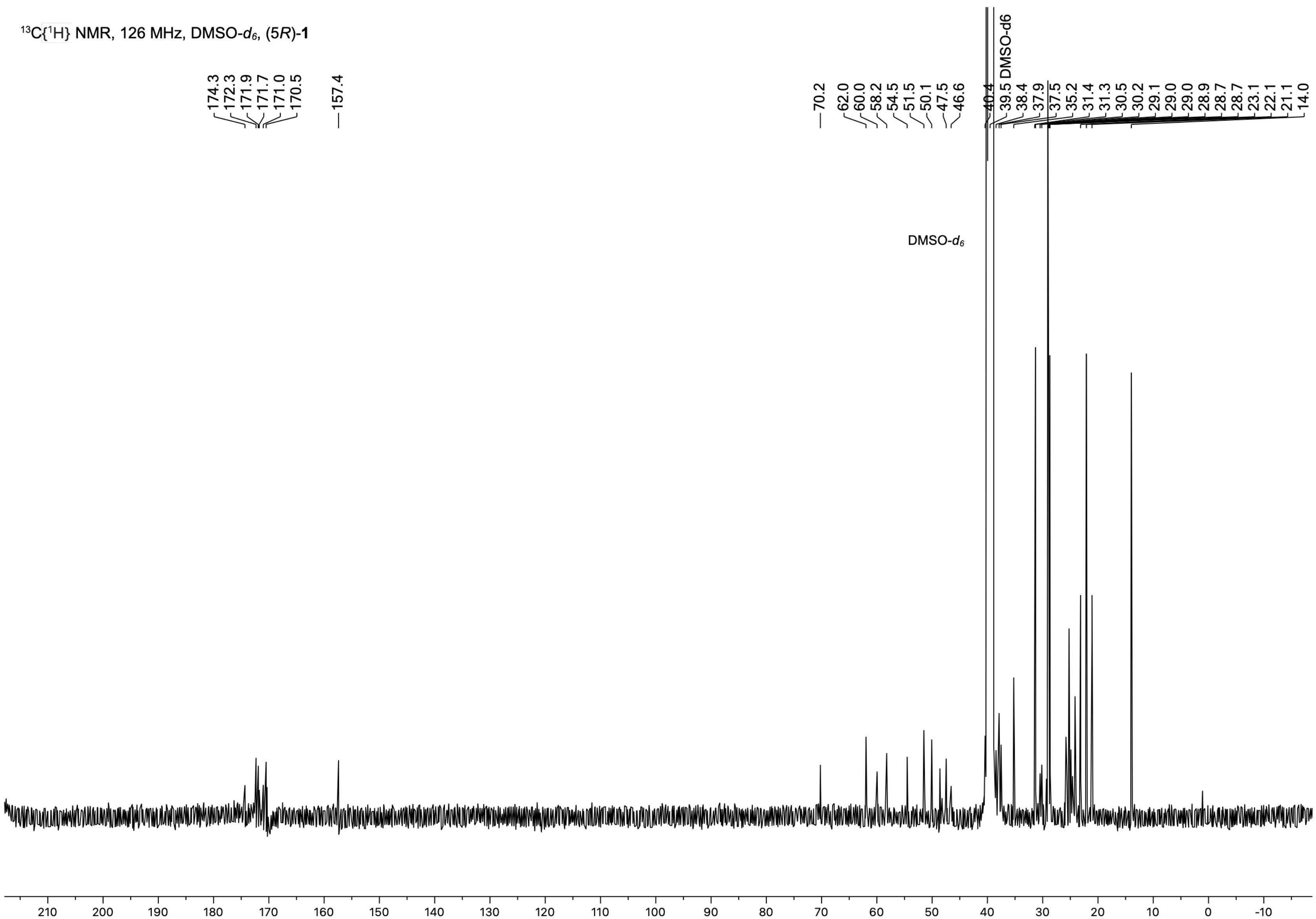

$^{13}C\{^{1}H\}$ NMR, 126 MHz, DMSO-d_6, (5*R*)-**1**
174.3
172.3
171.9
171.7
171.0
170.5
157.4
70.2
62.0
60.0
58.2
54.5
51.5
50.1
47.5
46.6
40.4
39.5 DMSO-d6
38.4
37.9
37.5
35.2
31.4
31.3
30.5
30.2
29.1
29.0
28.9
28.7
28.7
23.1
22.1
21.1
14.0
DMSO-d_6
210
200
190
180
170
160
150
140
130
120
110
100
90
80
70
60
50
40
30
20
10
0
-10

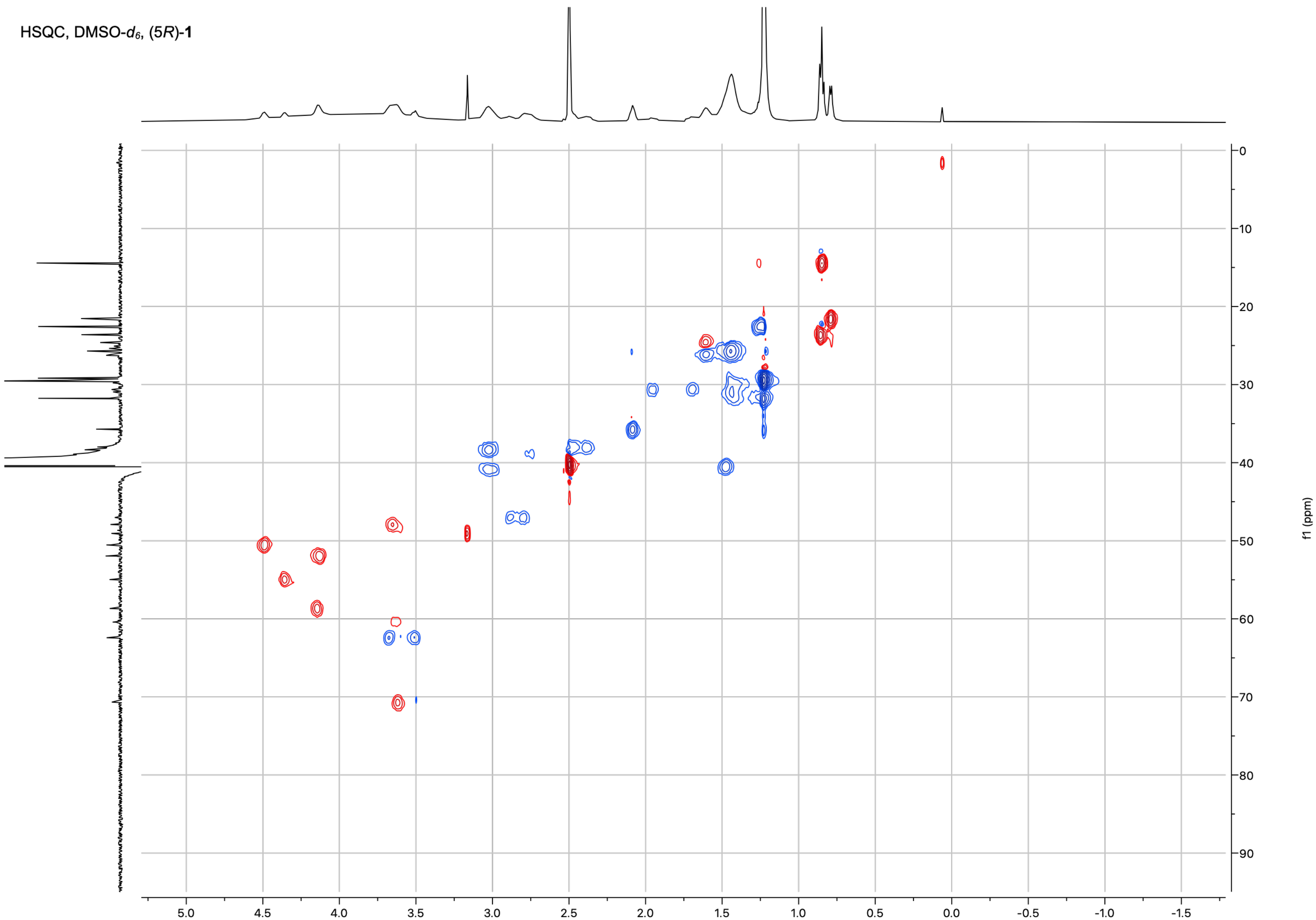
HSQC, DMSO-d_6, (5R)-**1**
f1 (ppm)
5.0
4.5
4.0
3.5
3.0
2.5
2.0
1.5
1.0
0.5
0.0
-0.5
-1.0
-1.5
0
10
20
30
40
50
60
70
80
90

^{1}H NMR, 500 MHz, DMSO-d_6, **S22**

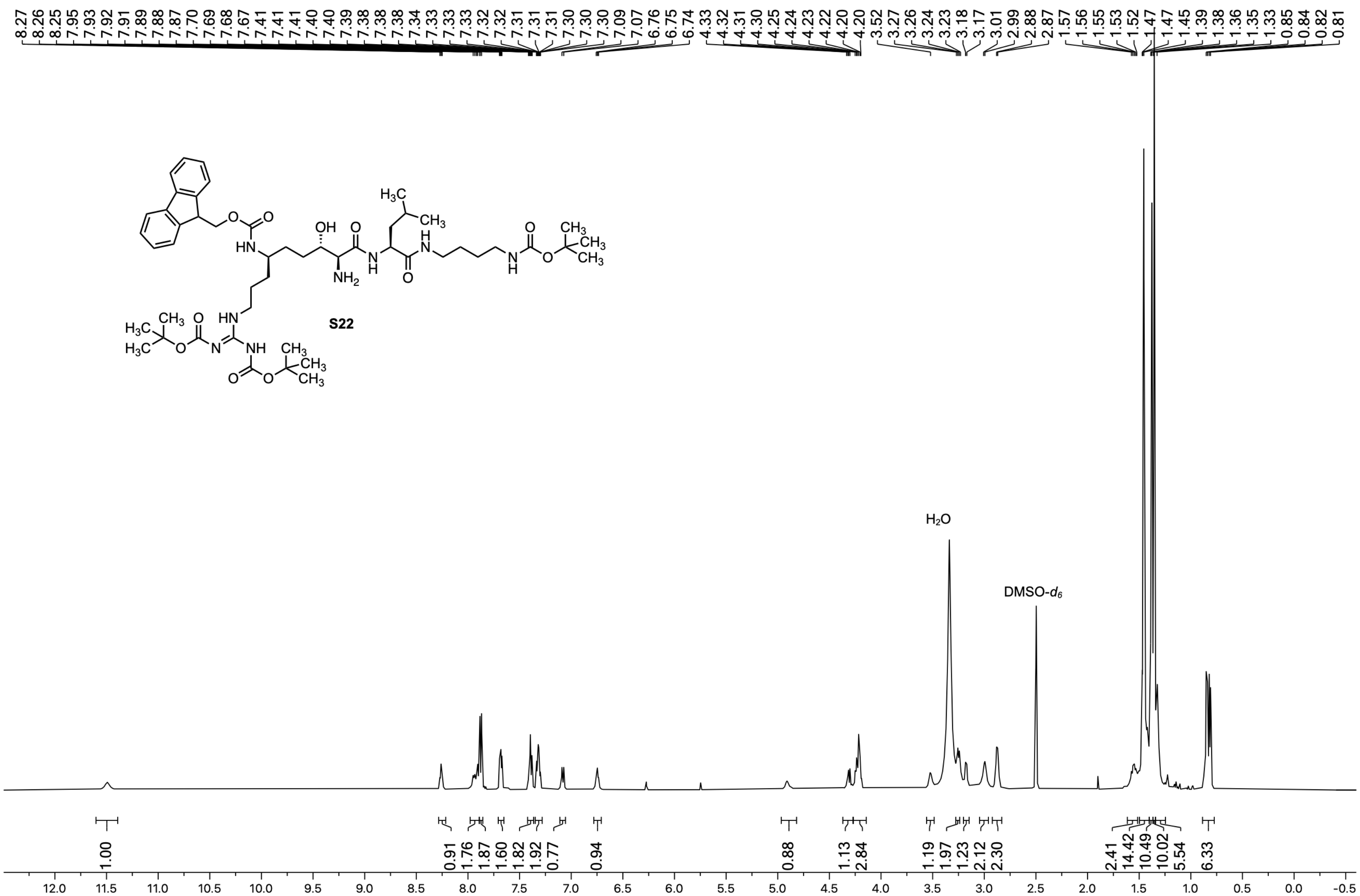

COSY, DMSO-d_6, **S22**

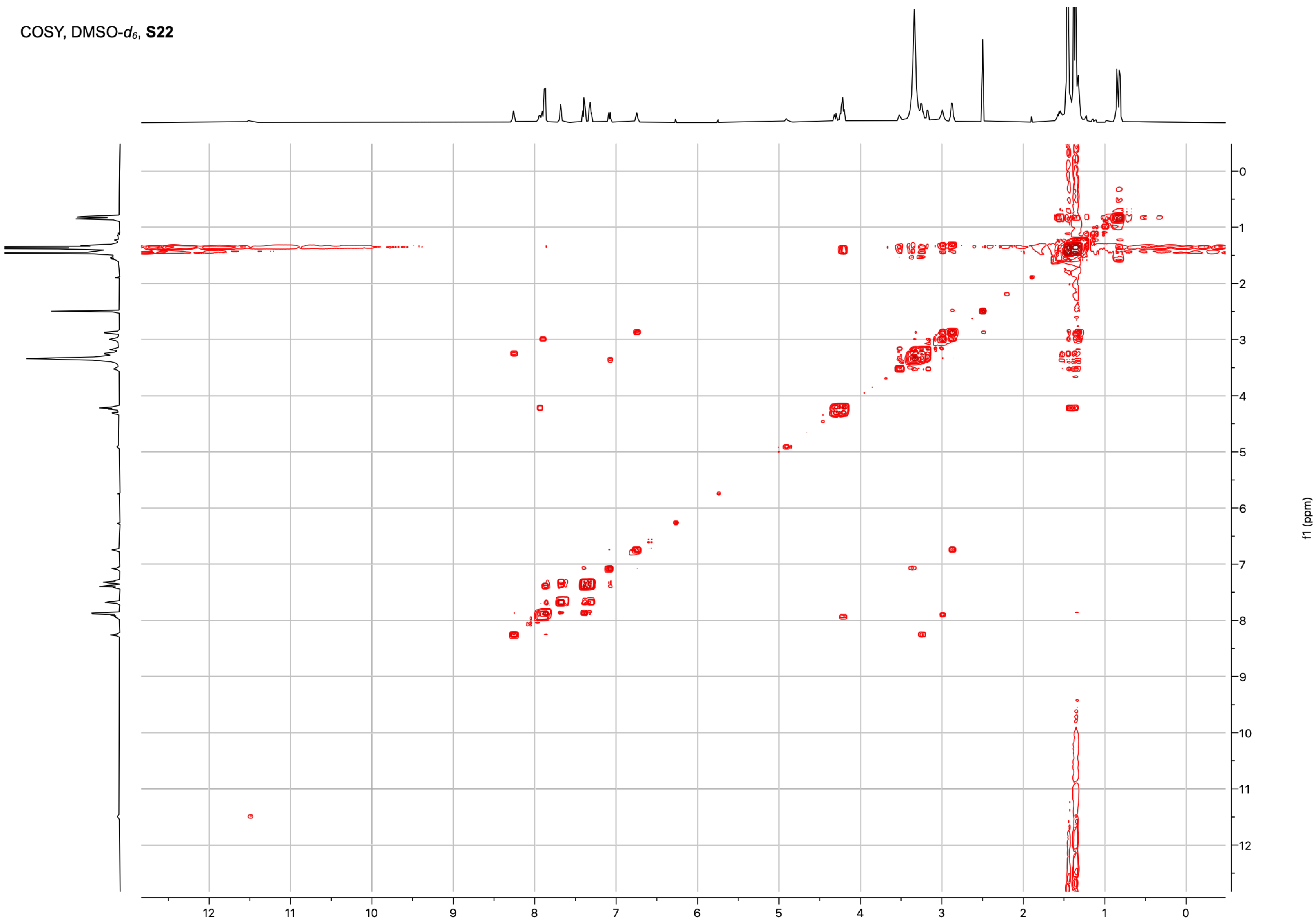

$^{13}C\{^{1}H\}$ NMR, 126 MHz, DMSO-d_6, **S22**

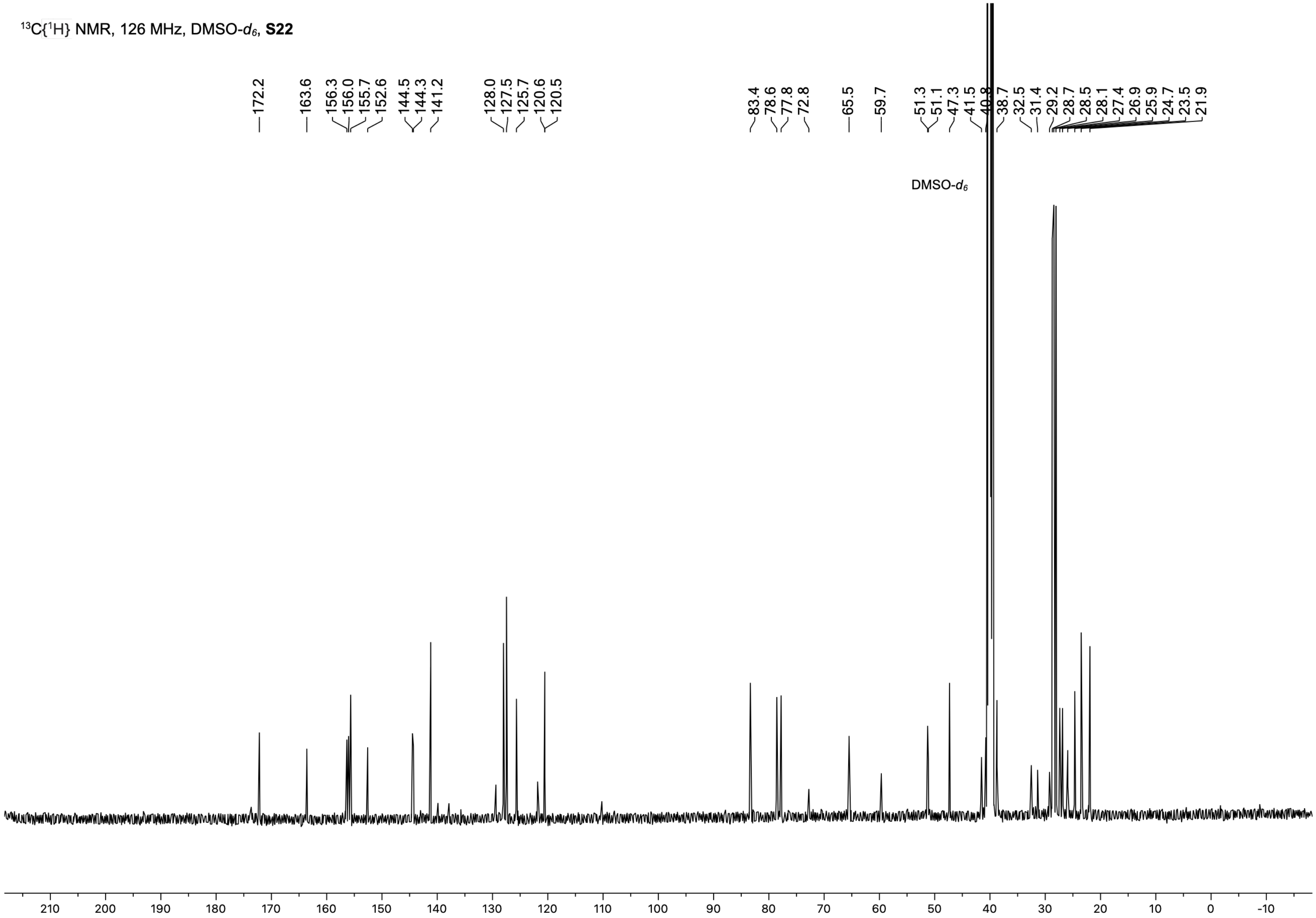

HSQC, DMSO-d_6, **S22**

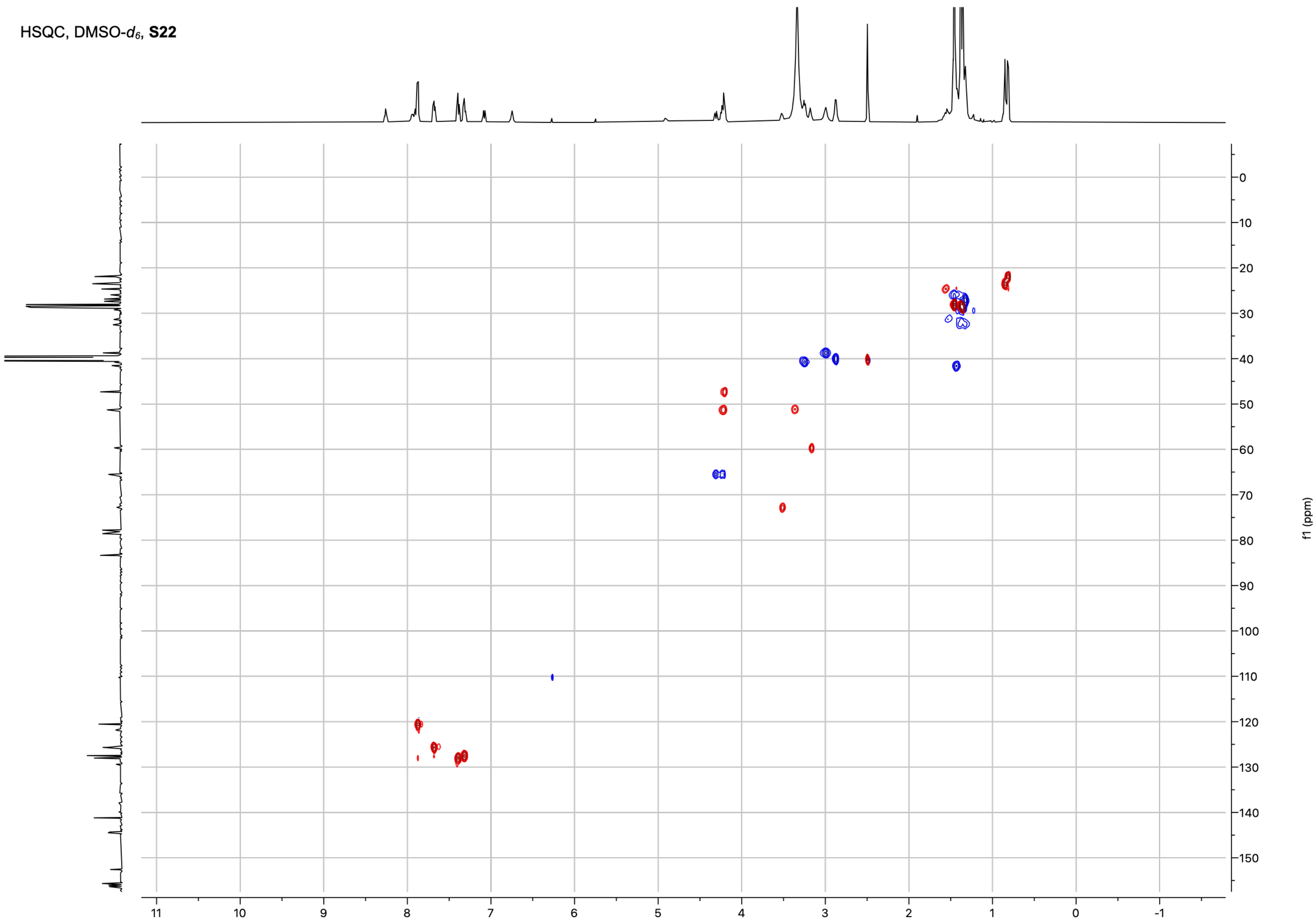

^1H NMR, 600 MHz, DMF-d_7, **S23**

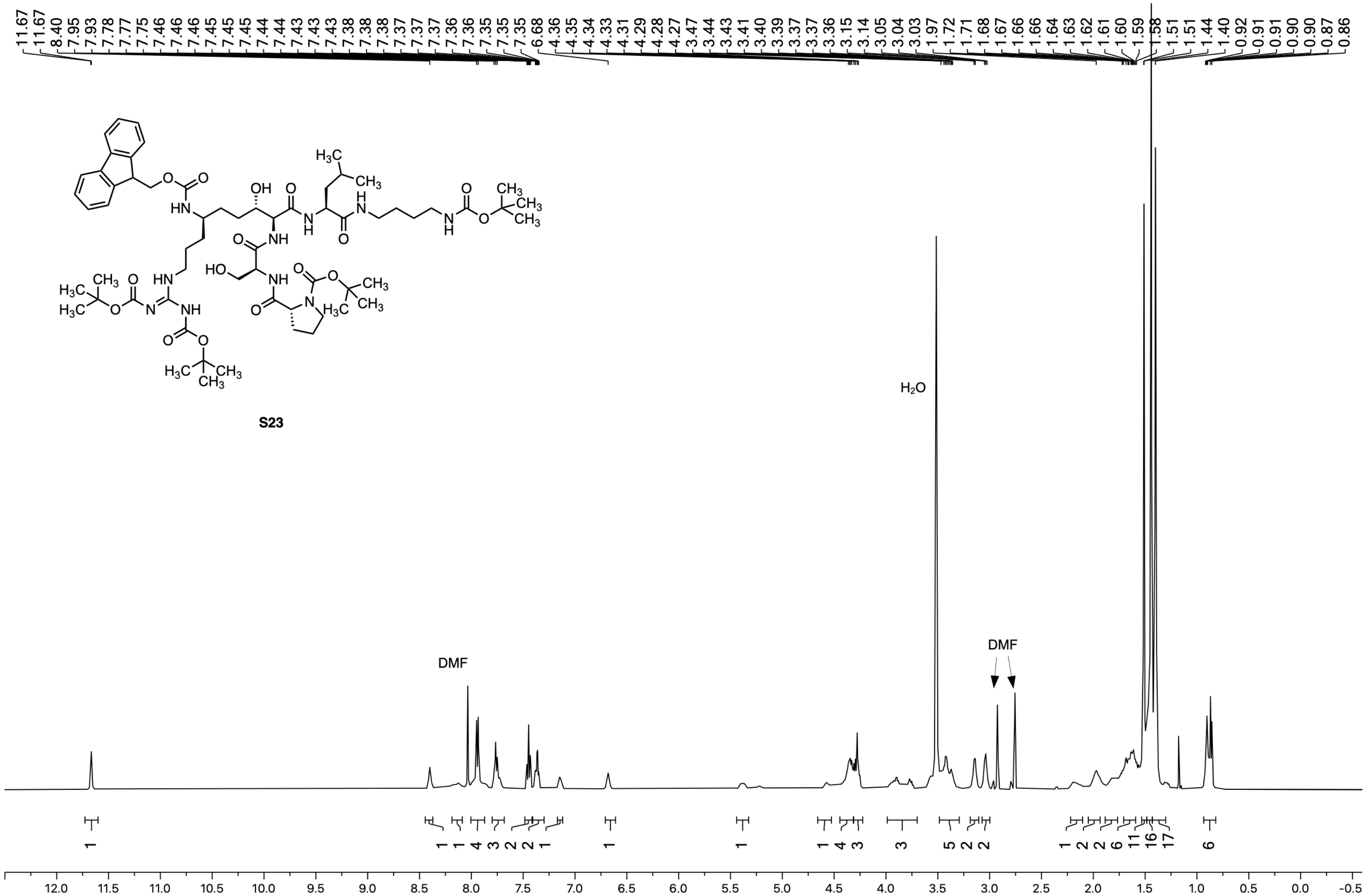

S23

COSY, DMF-d_7, **S23**

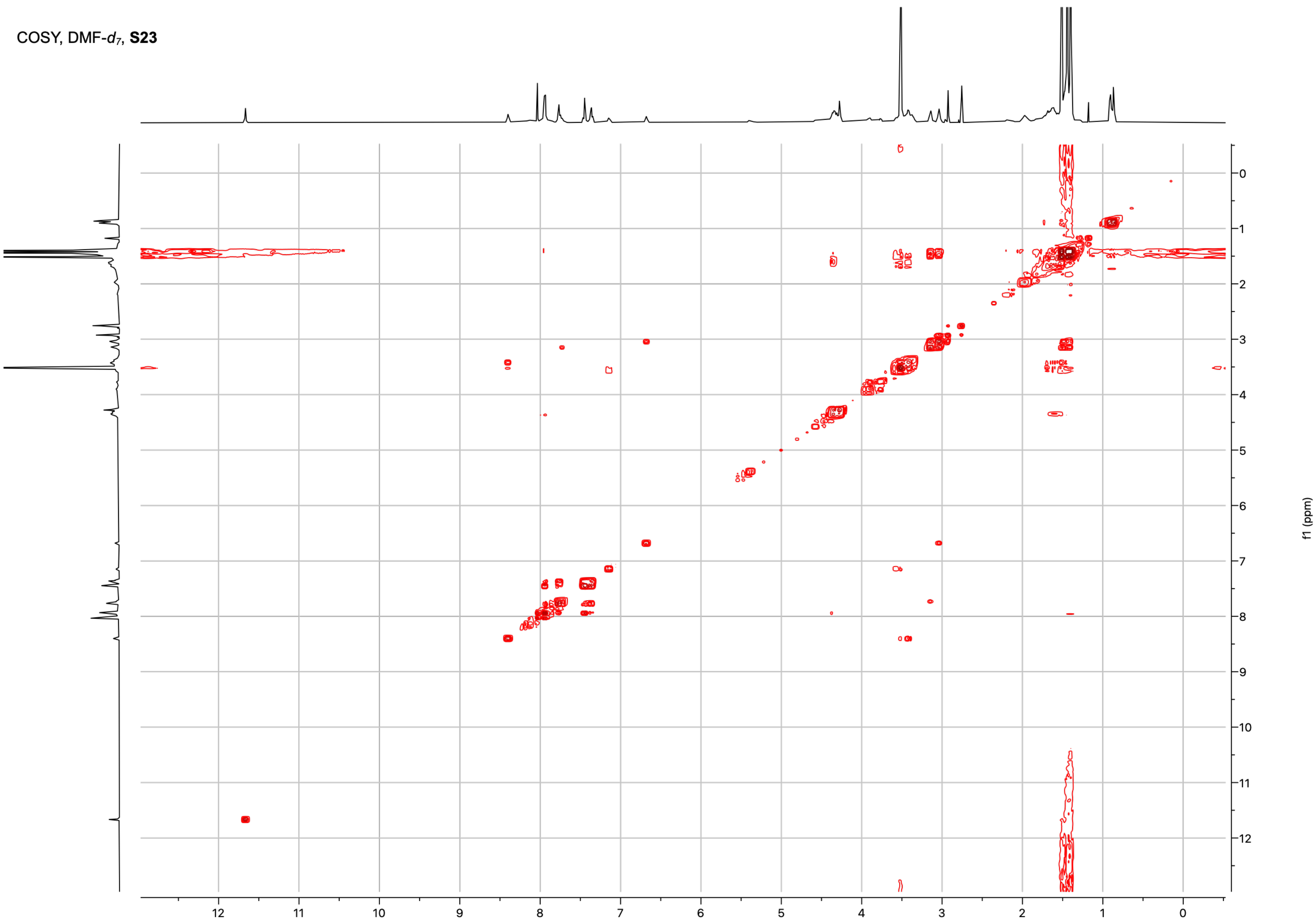

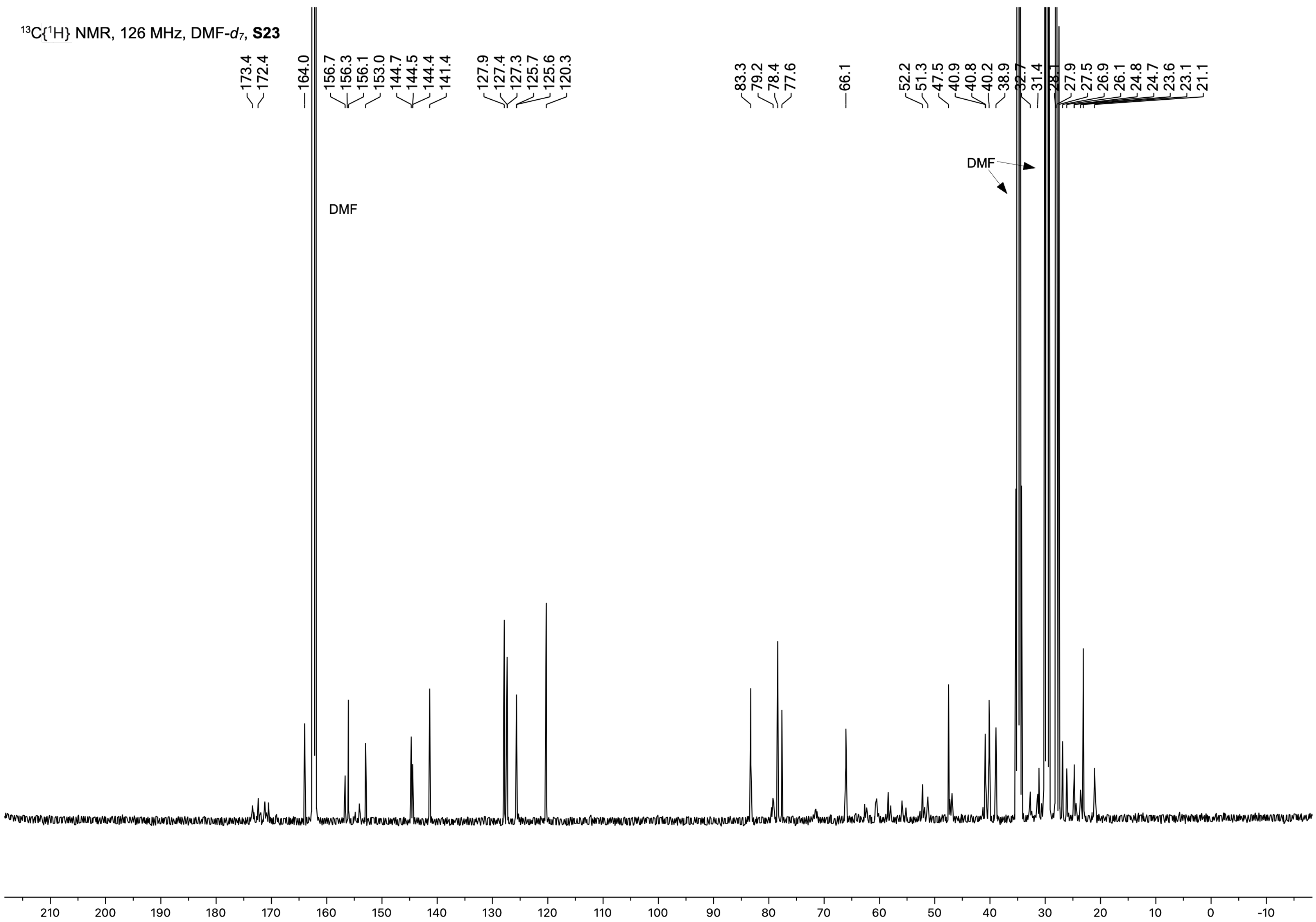
$^{13}C\{^{1}H\}$ NMR, 126 MHz, DMF-d_7, **S23**
173.4
172.4
164.0
156.7
156.3
156.1
153.0
144.7
144.5
144.4
141.4
127.9
127.4
127.3
125.7
125.6
120.3
83.3
79.2
78.4
77.6
66.1
52.2
51.3
47.5
40.9
40.8
40.2
38.9
32.7
31.4
28.1
27.9
27.5
26.9
26.1
24.8
24.7
23.6
23.1
21.1
DMF
DMF
210
200
190
180
170
160
150
140
130
120
110
100
90
80
70
60
50
40
30
20
10
0
-10

HSQC, DMF-d_7, **S23**

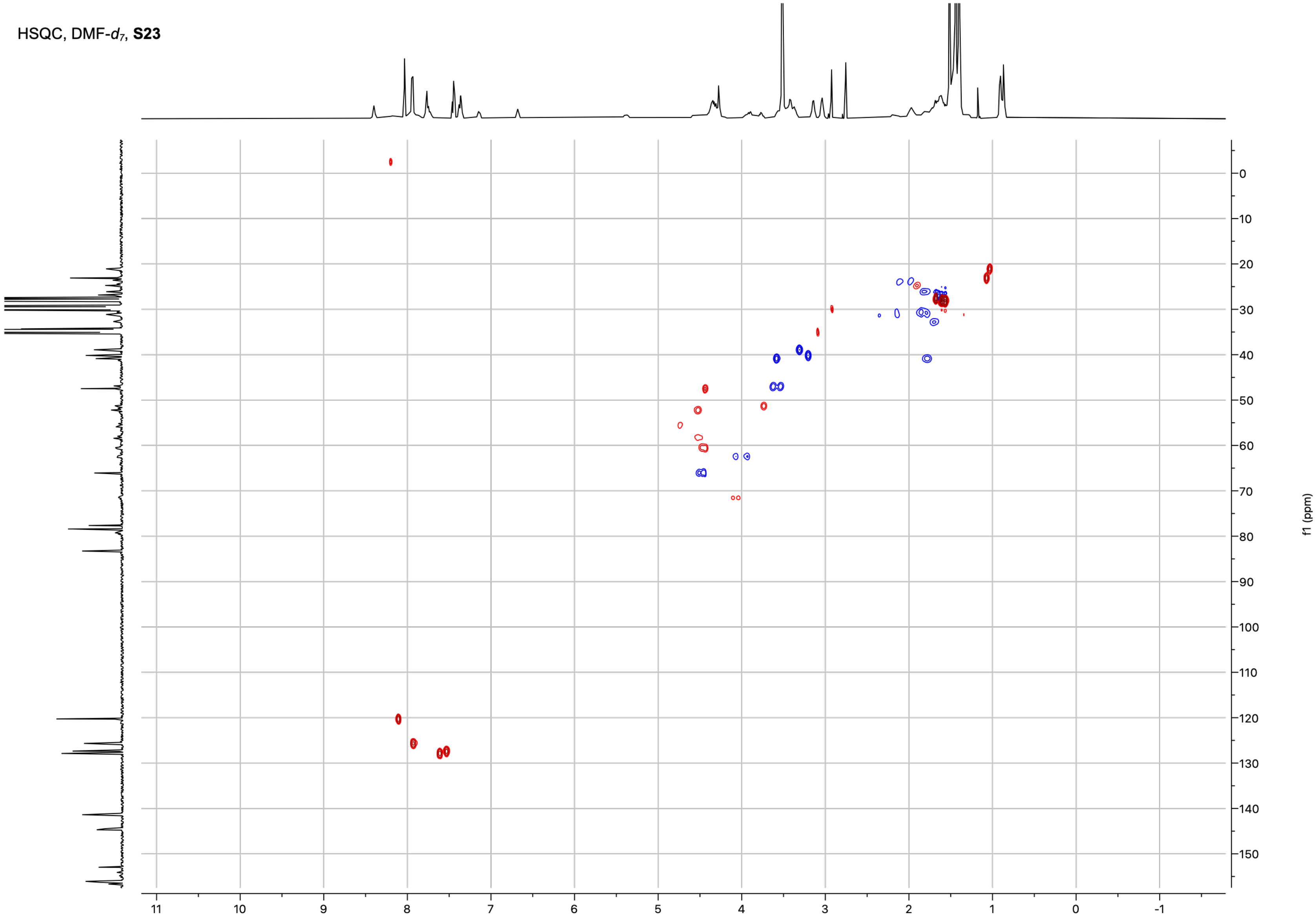

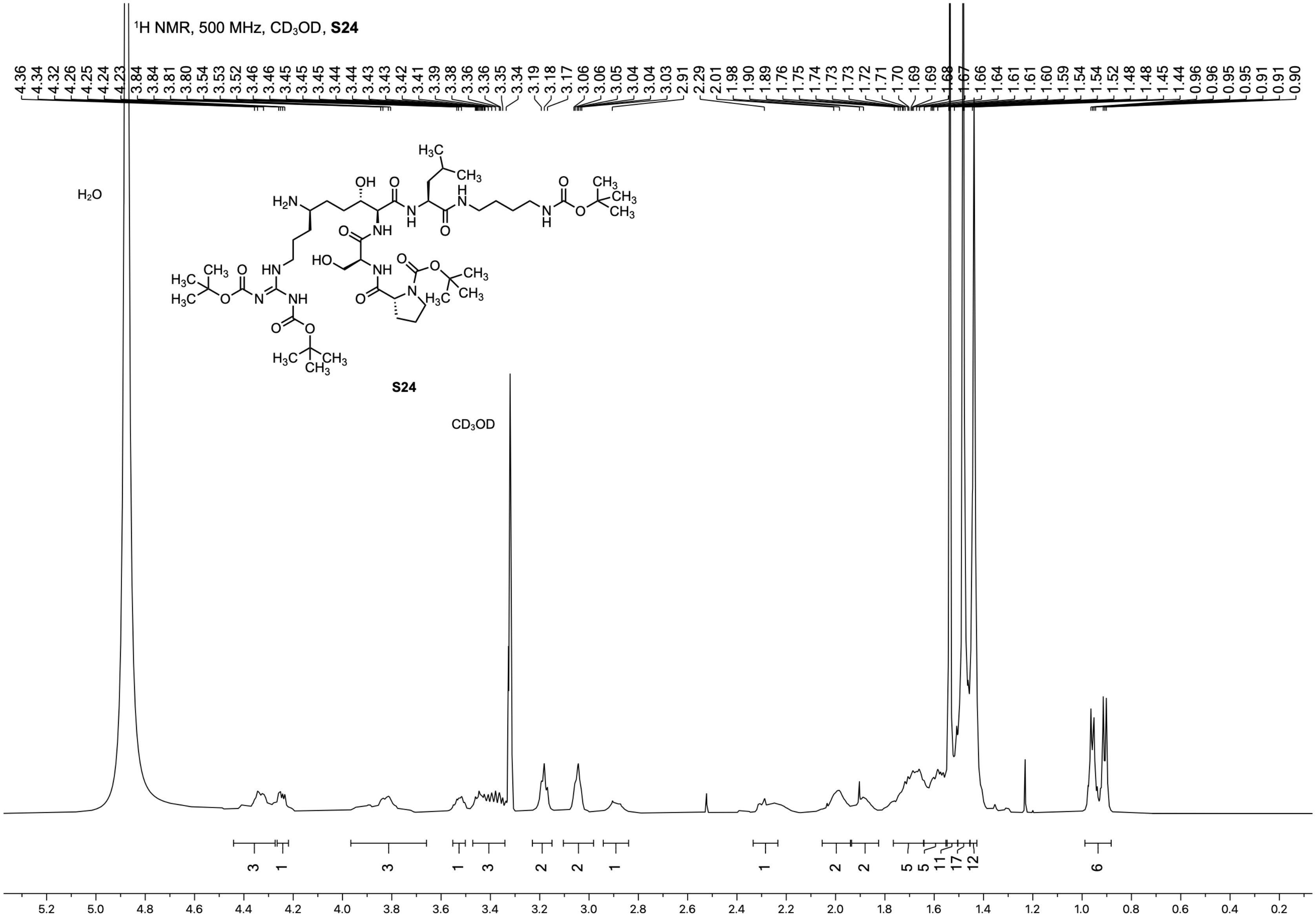

1H NMR, 500 MHz, CD3OD, S24
4.36 4.34 4.32 4.26 4.25 4.24 4.23 3.84 3.84 3.81 3.80 3.54 3.53 3.52 3.46 3.46 3.45 3.45 3.45 3.44 3.44 3.43 3.43 3.42 3.41 3.39 3.38 3.36 3.36 3.35 3.34 3.19 3.18 3.17 3.06 3.06 3.05 3.04 3.03 2.91 2.29 2.01 1.98 1.90 1.89 1.76 1.75 1.74 1.73 1.73 1.72 1.71 1.70 1.69 1.69 1.68 1.67 1.66 1.64 1.61 1.61 1.60 1.59 1.54 1.54 1.52 1.48 1.48 1.45 1.44 0.96 0.96 0.95 0.91 0.91 0.90
H2O
CD3OD
S24
3 1 3 1 3 2 2 1 1 2 2 5 5 11 17 12 6
5.2 5.0 4.8 4.6 4.4 4.2 4.0 3.8 3.6 3.4 3.2 3.0 2.8 2.6 2.4 2.2 2.0 1.8 1.6 1.4 1.2 1.0 0.8 0.6 0.4 0.2

COSY, CD_3OD, **S24**

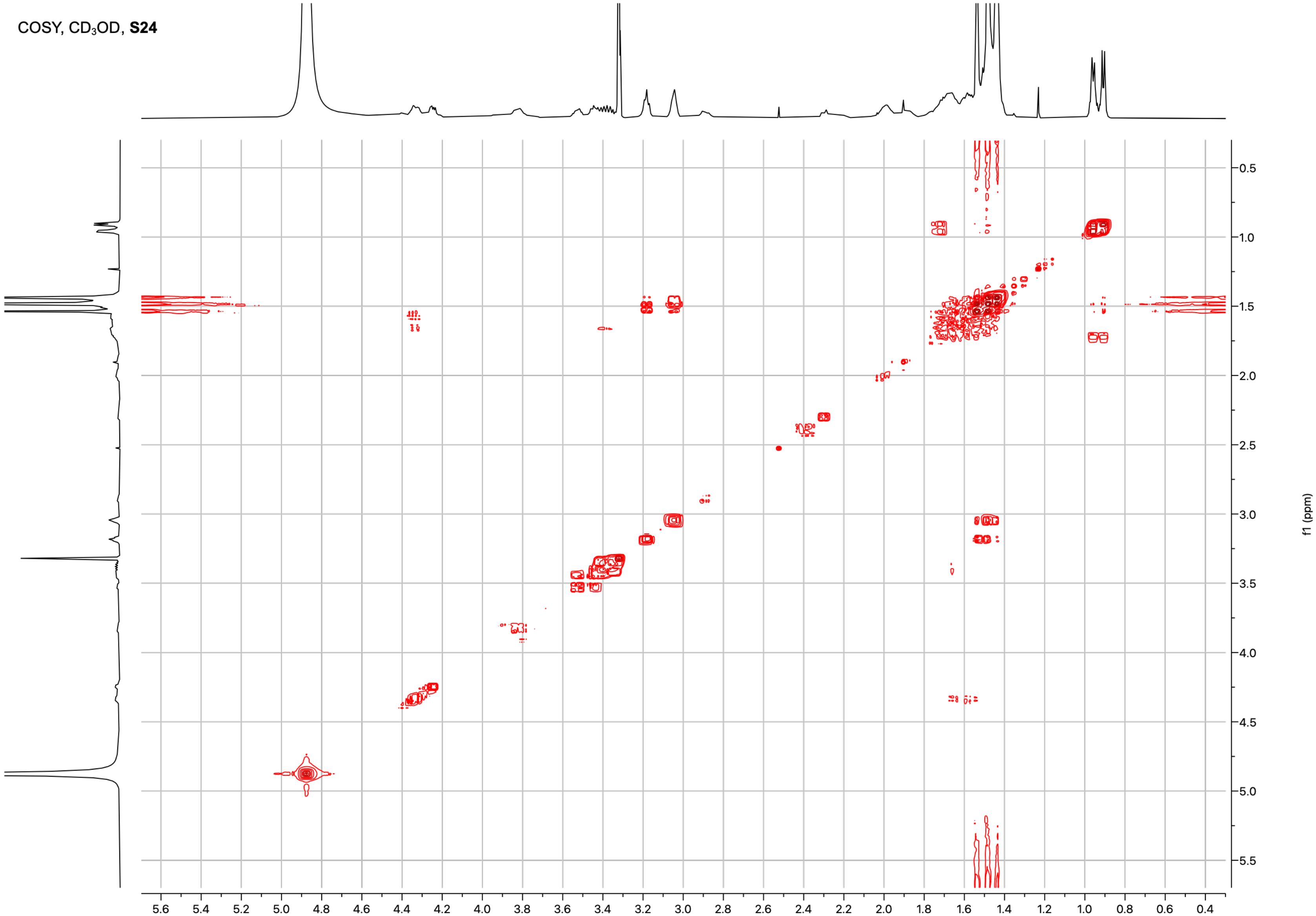

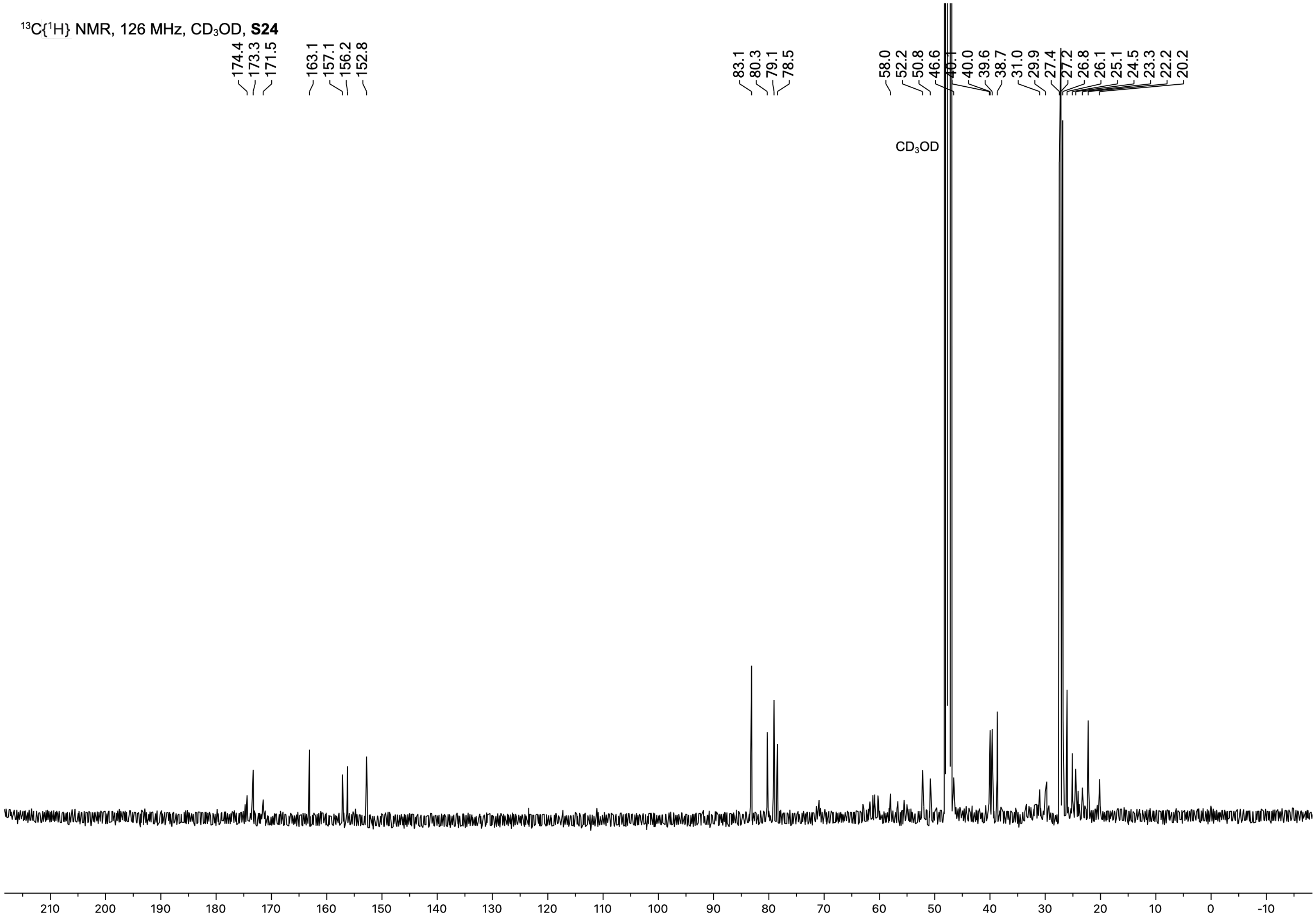

$^{13}C\{^1H\}$ NMR, 126 MHz, CD_3OD, **S24**
174.4
173.3
171.5
163.1
157.1
156.2
152.8
83.1
80.3
79.1
78.5
58.0
52.2
50.8
46.6
40.1
40.0
39.6
38.7
31.0
29.9
27.4
27.2
26.8
26.1
25.1
24.5
23.3
22.2
20.2
CD_3OD
210 200 190 180 170 160 150 140 130 120 110 100 90 80 70 60 50 40 30 20 10 0 -10

HSQC, CD_3OD, **S24**

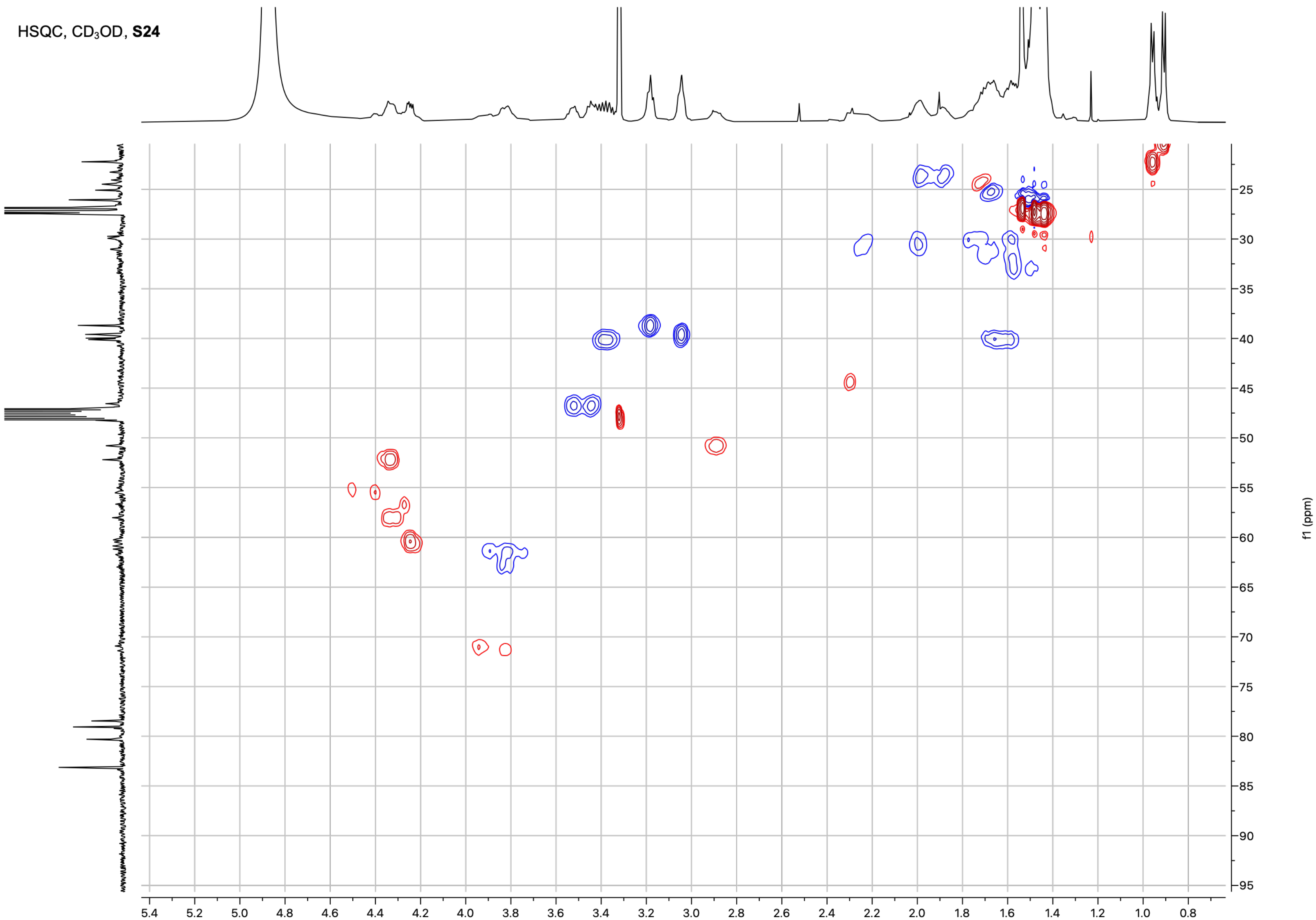

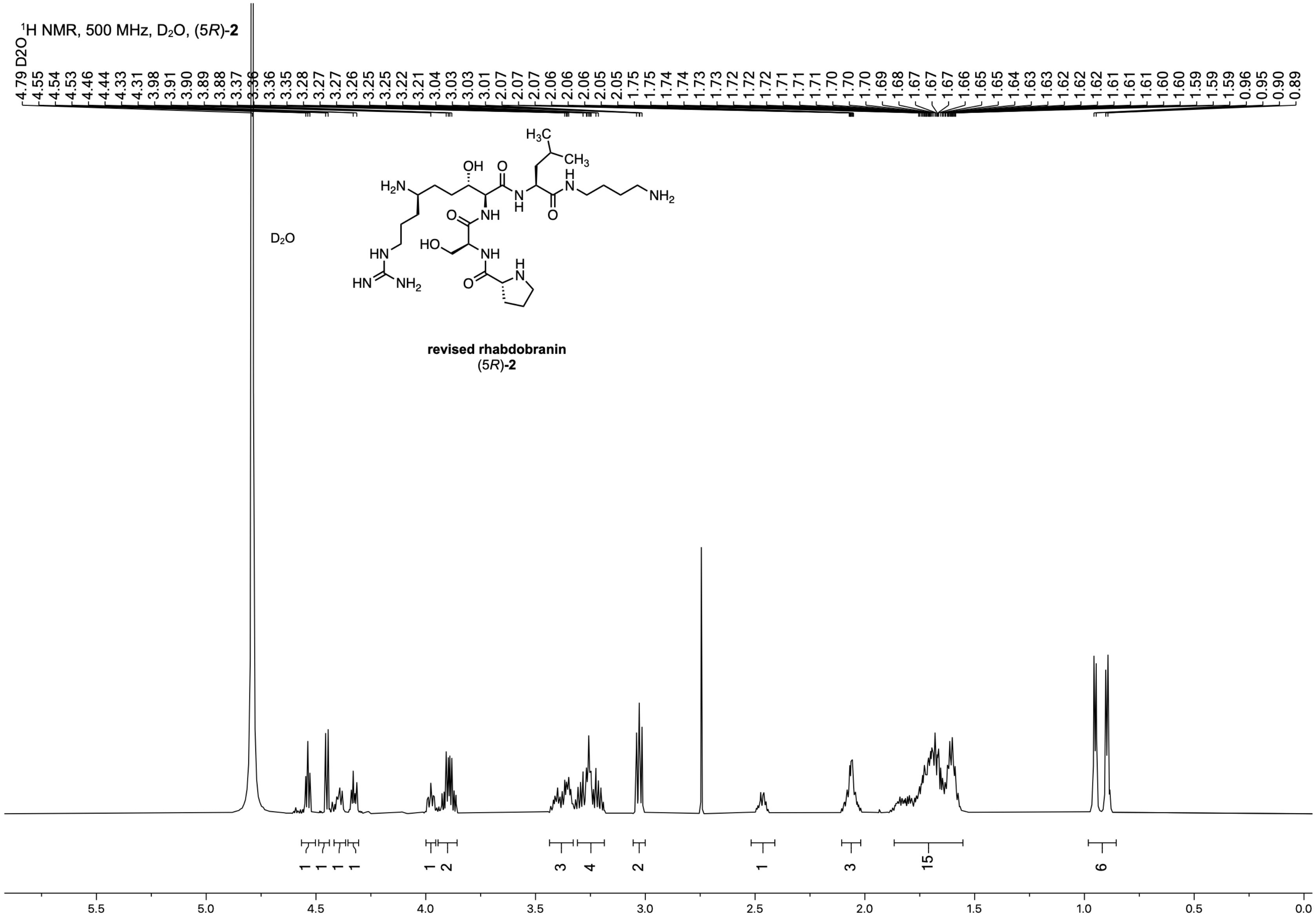

1H NMR, 500 MHz, D2O, (5R)-2
4.79 D2O 4.55 4.54 4.53 4.46 4.44 4.33 4.31 3.98 3.91 3.90 3.89 3.88 3.37 3.36 3.36 3.35 3.28 3.27 3.27 3.26 3.25 3.25 3.22 3.21 3.04 3.03 3.03 3.01 2.07 2.07 2.07 2.06 2.06 2.06 2.05 2.05 1.75 1.75 1.74 1.74 1.73 1.73 1.72 1.72 1.72 1.71 1.71 1.71 1.70 1.70 1.69 1.68 1.67 1.67 1.67 1.66 1.65 1.65 1.64 1.63 1.63 1.62 1.62 1.62 1.61 1.61 1.61 1.60 1.59 1.59 1.59 0.96 0.95 0.90 0.89
D2O
revised rhabdobranin
(5R)-2
1 1 1 1 1 2 3 4 2 1 3 15 6
5.5 5.0 4.5 4.0 3.5 3.0 2.5 2.0 1.5 1.0 0.5 0.0

COSY, D_2O, (5*R*)-**2**

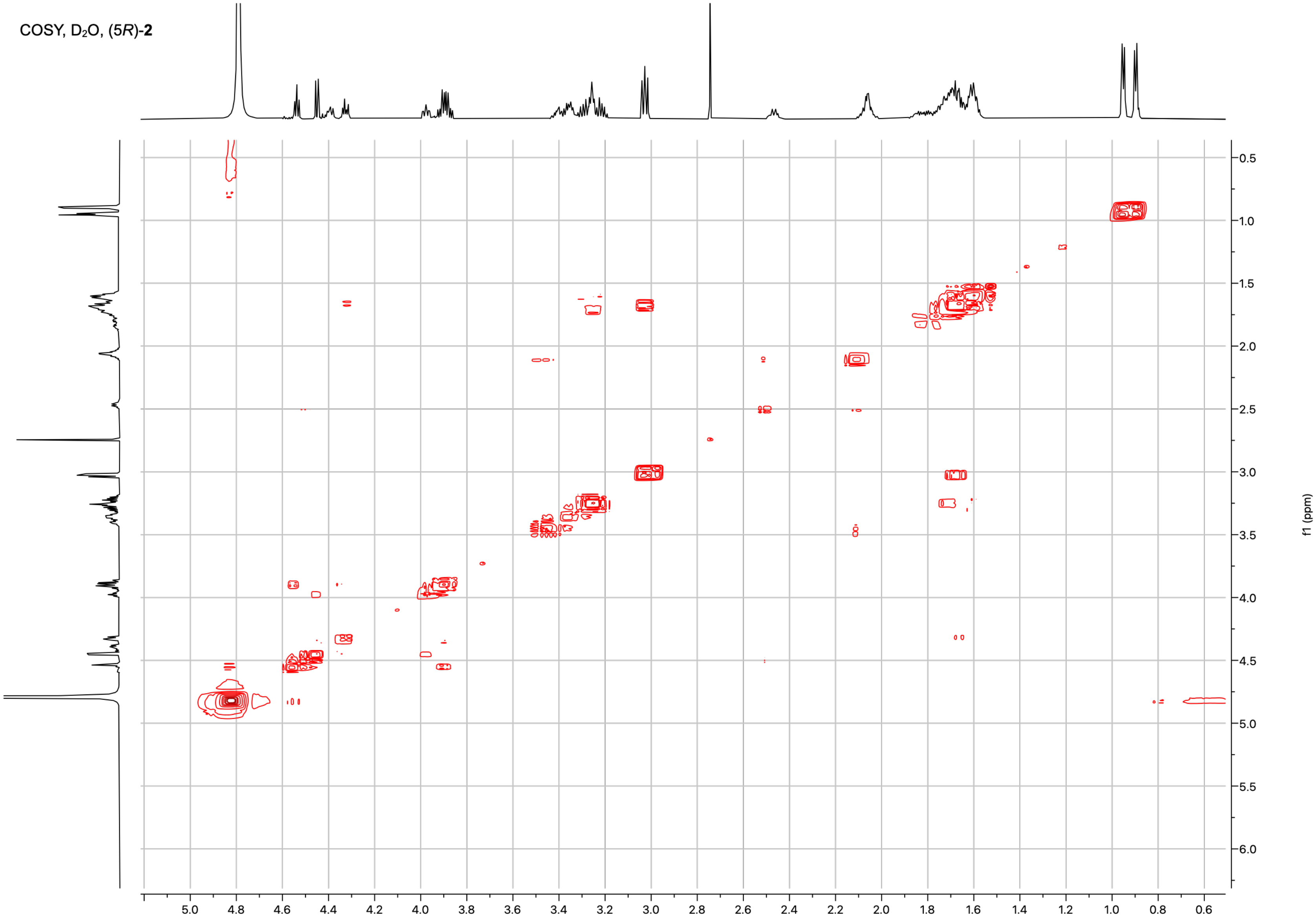

5.0
4.8
4.6
4.4
4.2
4.0
3.8
3.6
3.4
3.2
3.0
2.8
2.6
2.4
2.2
2.0
1.8
1.6
1.4
1.2
1.0
0.8
0.6
0.5
1.0
1.5
2.0
2.5
3.0
3.5
4.0
4.5
5.0
5.5
6.0
f1 (ppm)

$^{13}C\{^{1}H\}$ NMR, 126 MHz, D_2O, (5*R*)-**2**

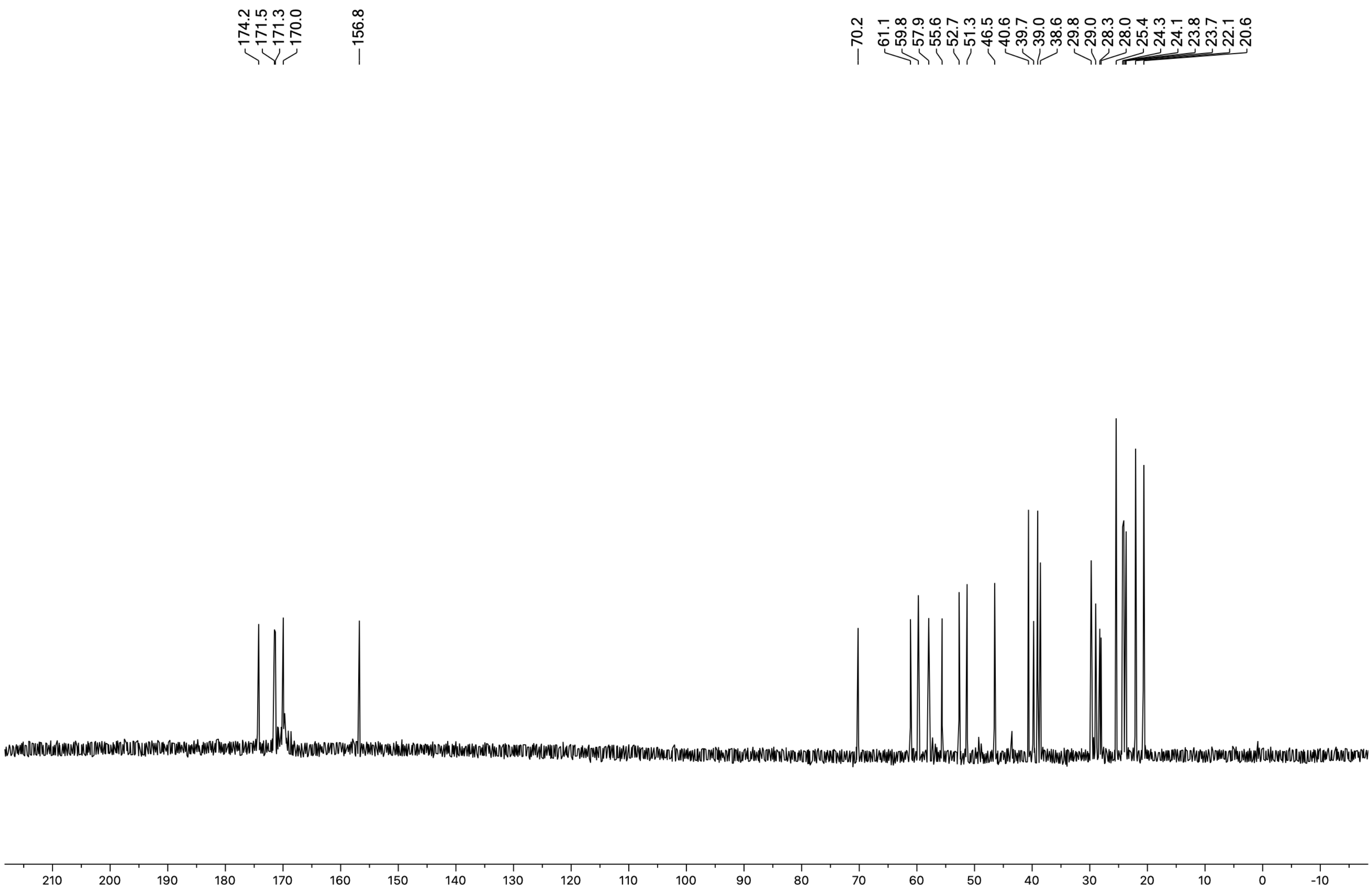

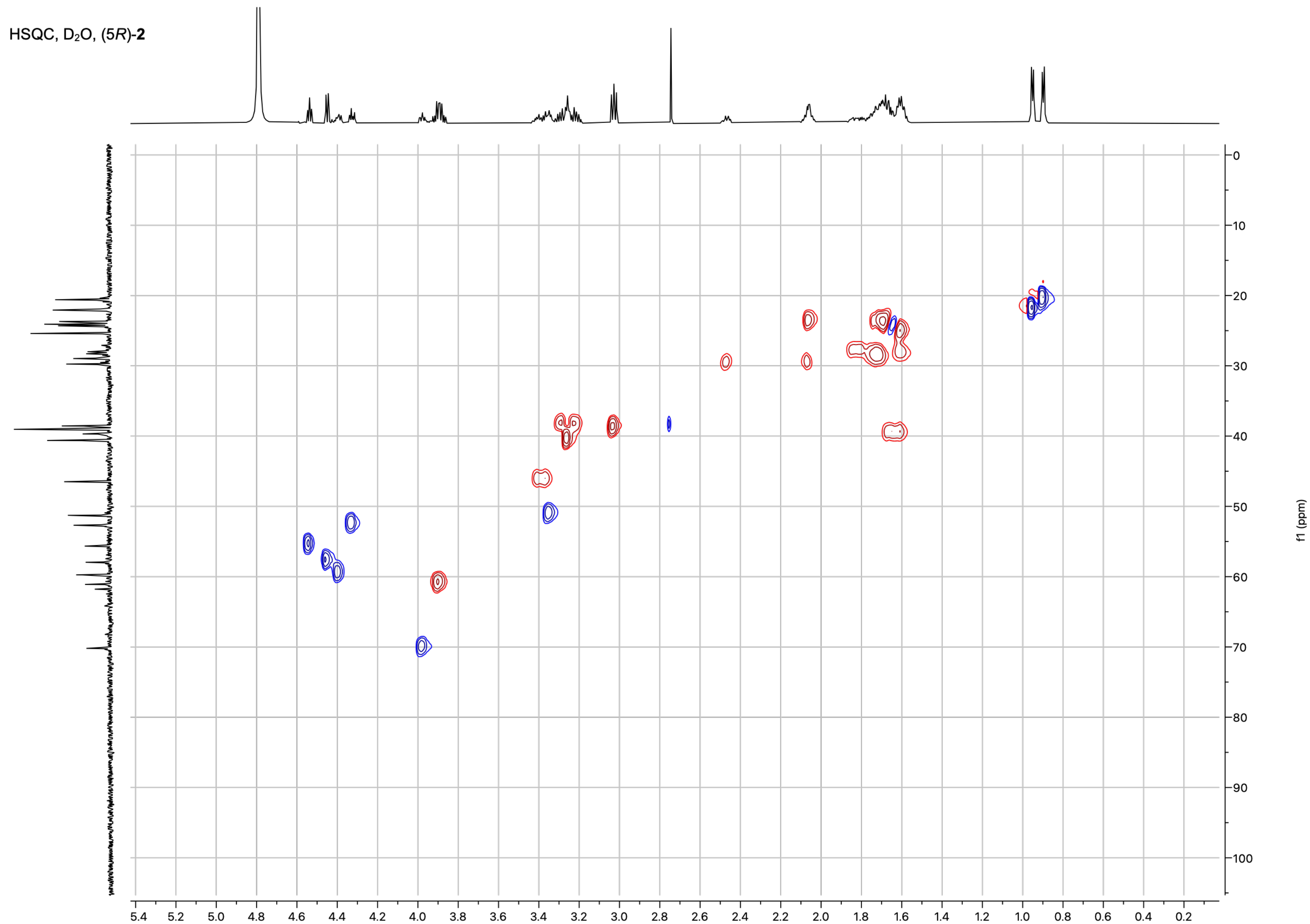
HSQC, D_2O, (5*R*)-**2**
f1 (ppm)

^1H NMR, 500 MHz, CD_3OD, **S25**

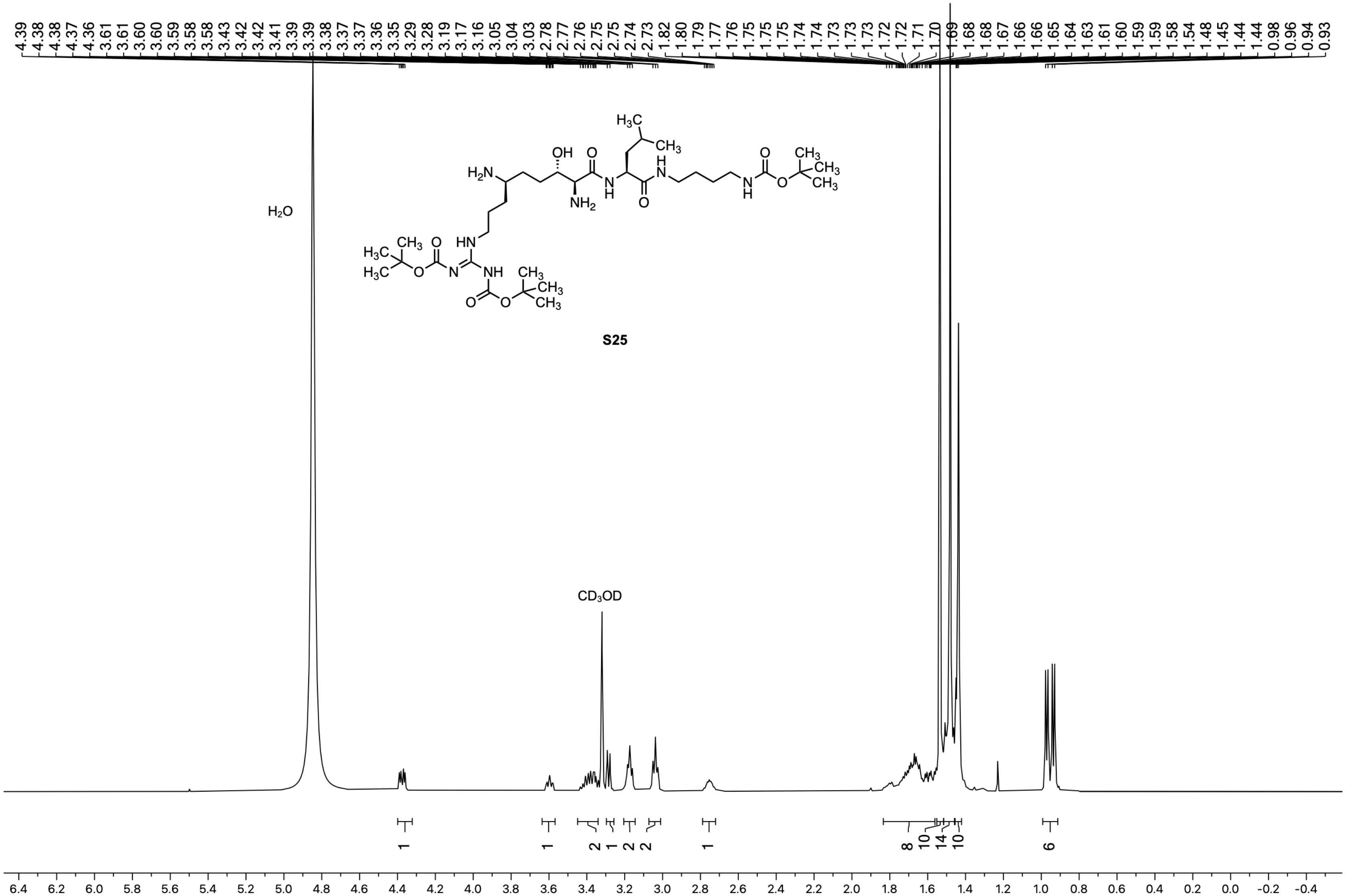

COSY, CD_3OD, **S25**

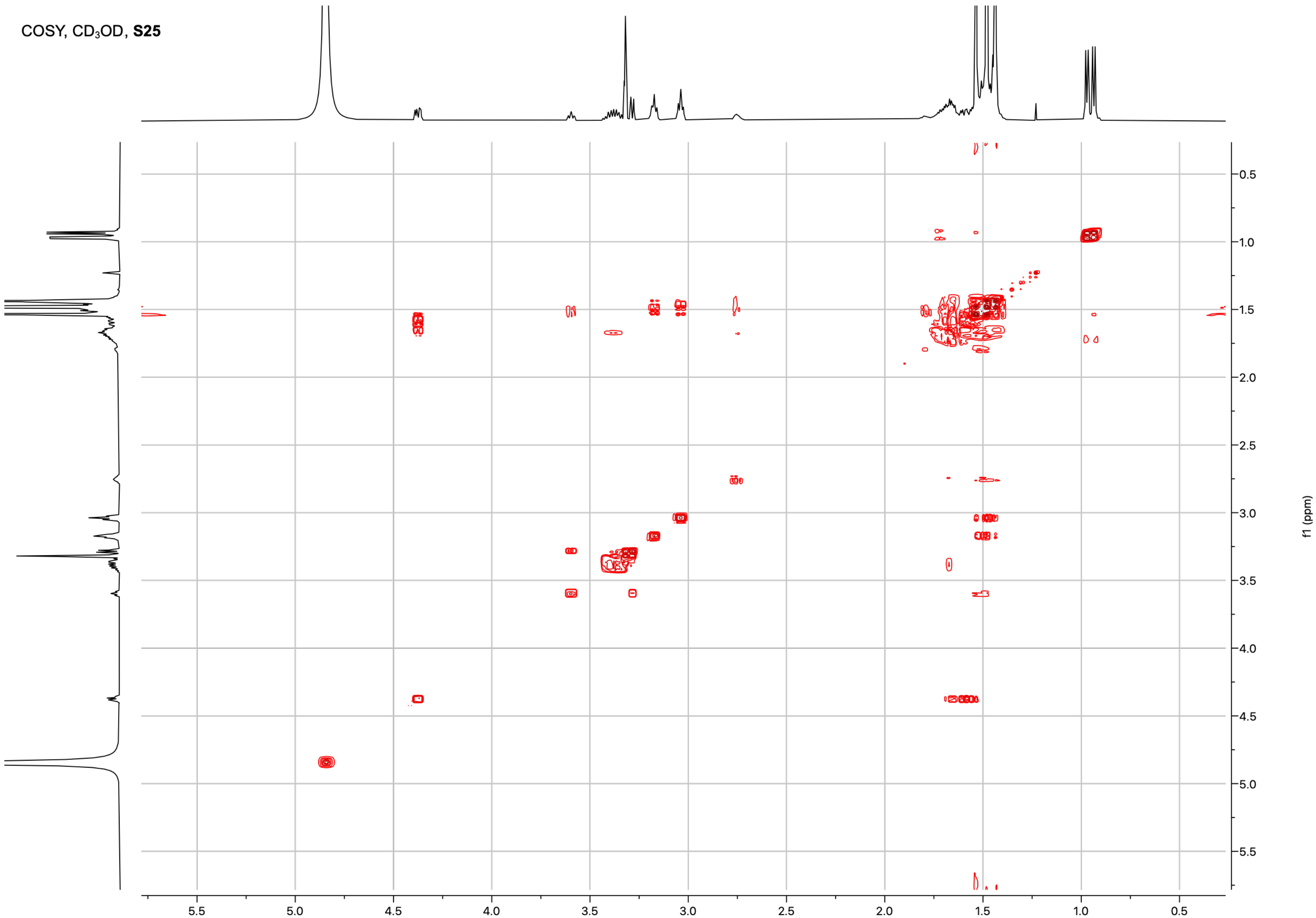

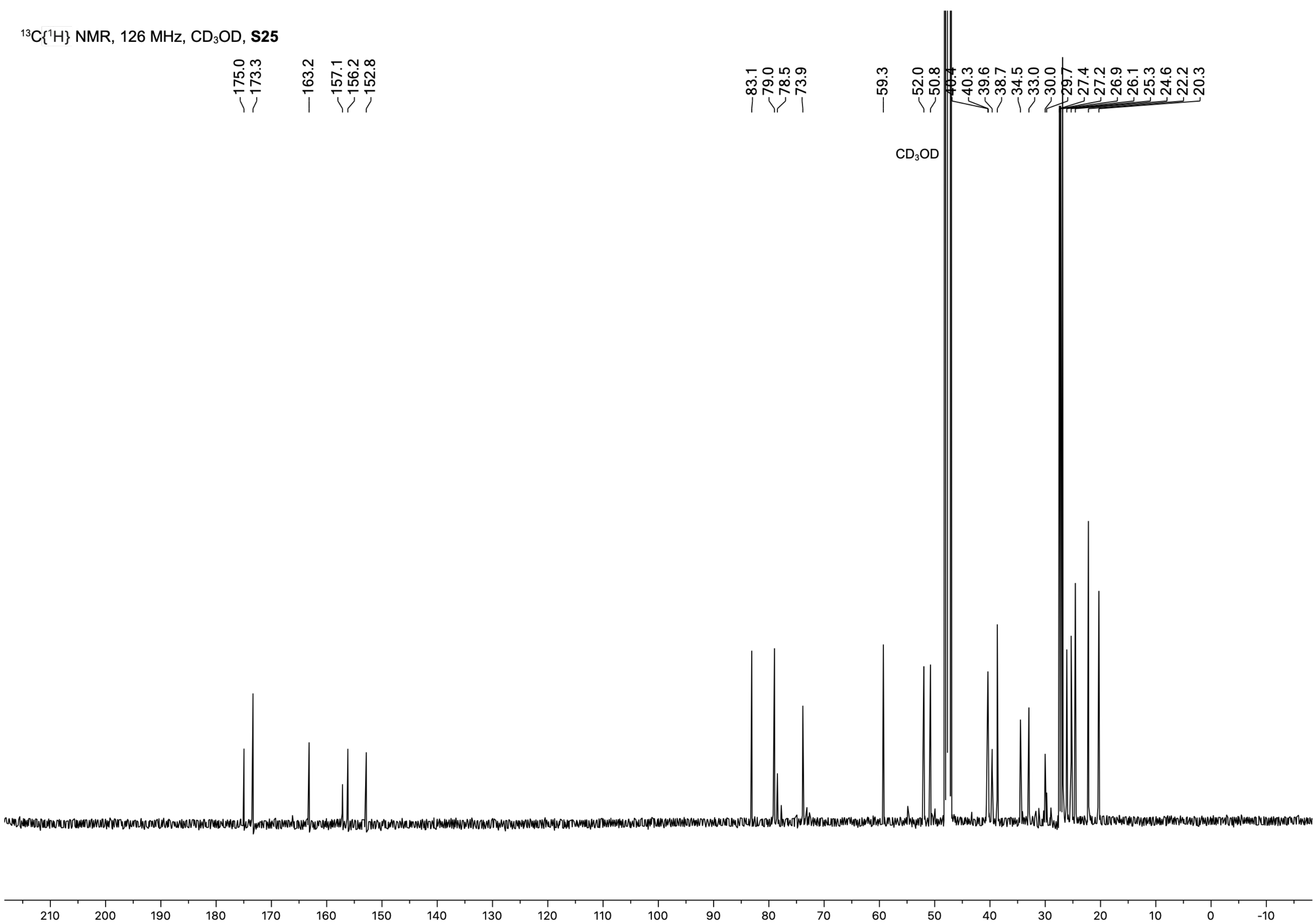
$^{13}C\{^1H\}$ NMR, 126 MHz, CD_3OD, **S25**
175.0
173.3
163.2
157.1
156.2
152.8
83.1
79.0
78.5
73.9
59.3
52.0
50.8
40.3
39.6
38.7
34.5
33.0
30.0
29.7
27.4
27.2
26.9
26.1
25.3
24.6
22.2
20.3
CD_3OD
210
200
190
180
170
160
150
140
130
120
110
100
90
80
70
60
50
40
30
20
10
0
-10

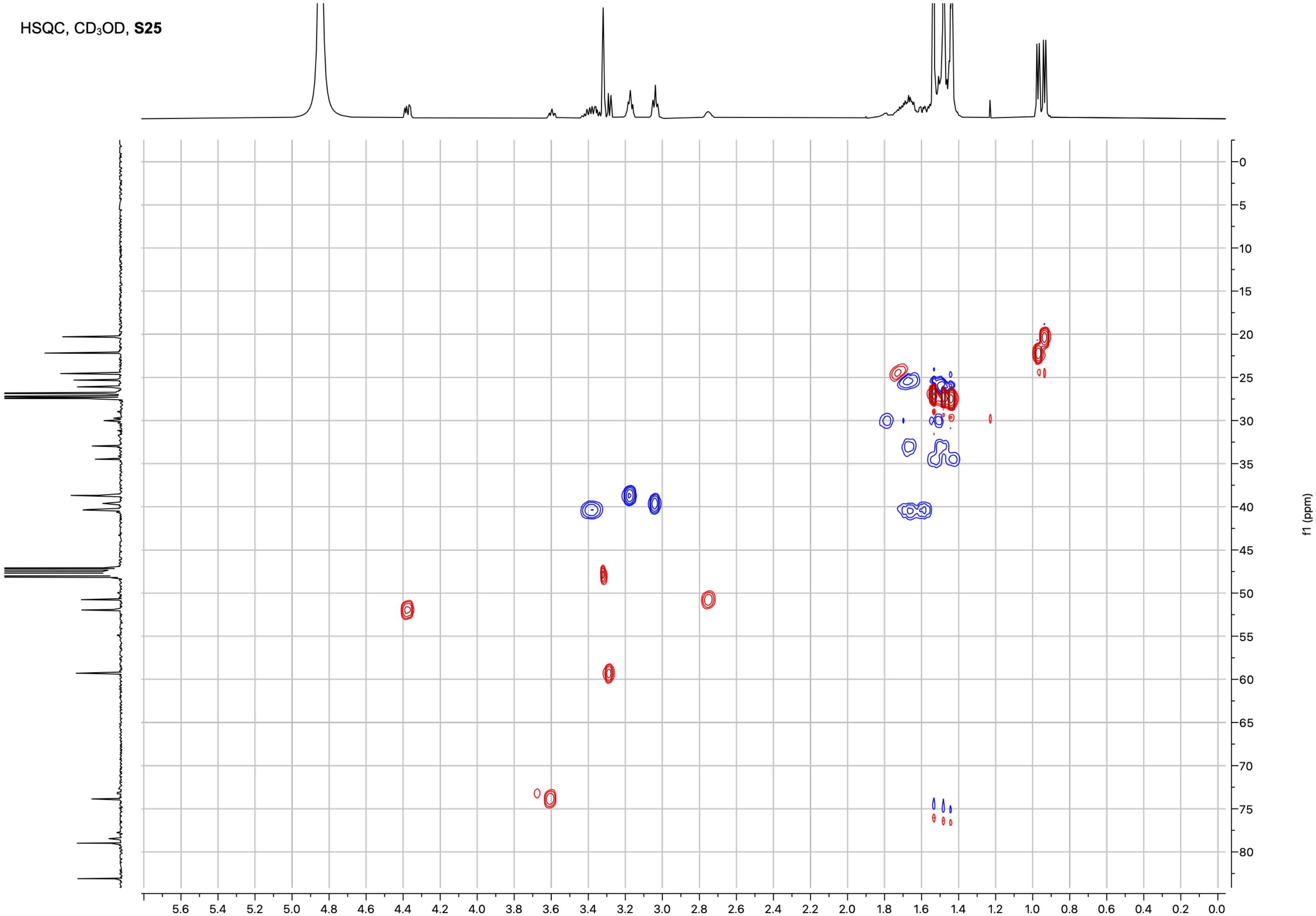
HSQC, CD3OD, S25
f1 (ppm)

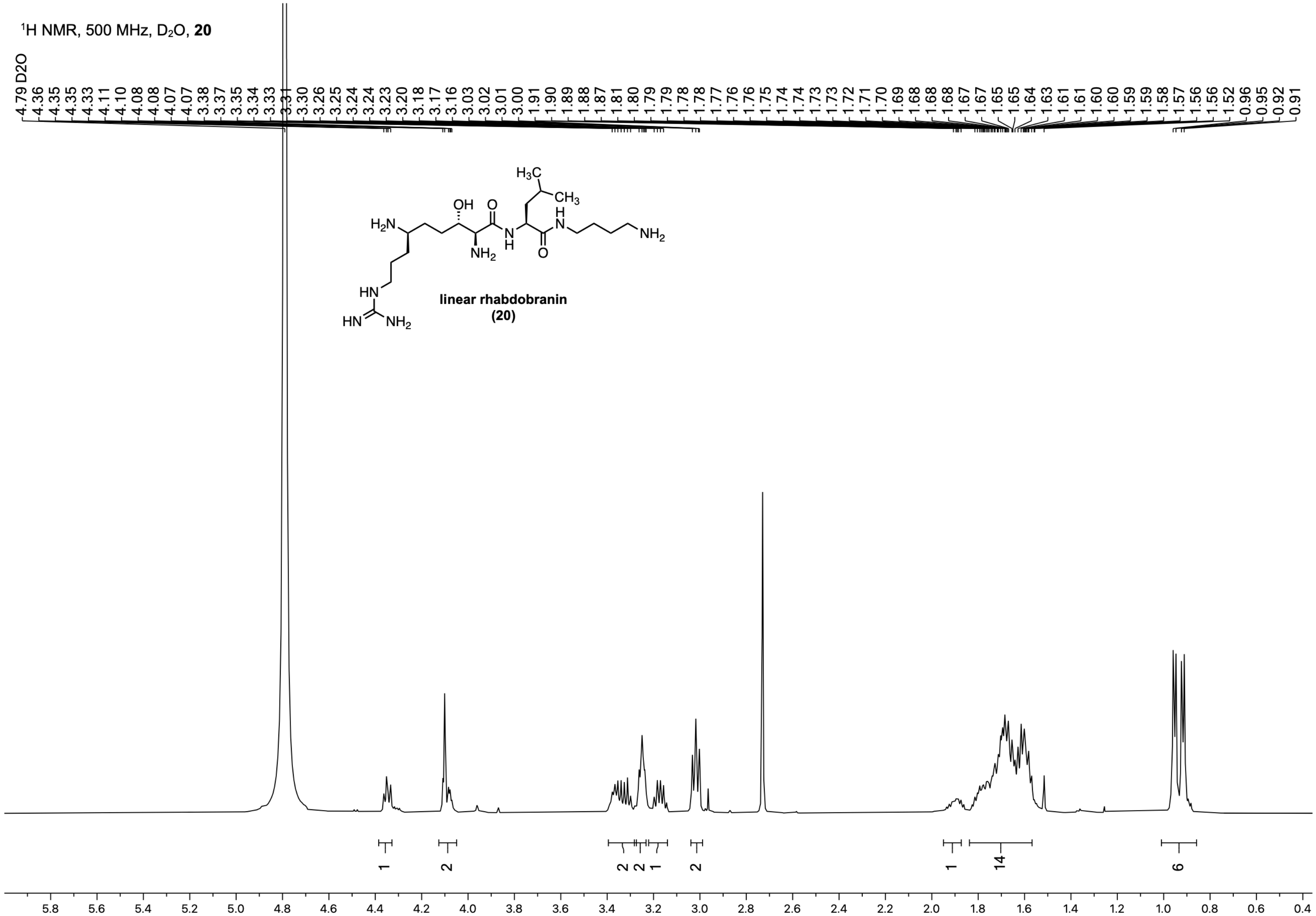

1H NMR, 500 MHz, D2O, 20
4.79 D2O
4.36 4.35 4.35 4.33 4.11 4.10 4.08 4.08 4.07 3.38 3.37 3.35 3.34 3.33 3.31 3.30 3.26 3.25 3.24 3.24 3.23 3.20 3.18 3.17 3.16 3.03 3.02 3.01 3.00 1.91 1.90 1.89 1.88 1.87 1.81 1.80 1.79 1.79 1.78 1.78 1.77 1.76 1.76 1.75 1.74 1.74 1.73 1.73 1.72 1.71 1.70 1.69 1.68 1.68 1.68 1.67 1.67 1.65 1.65 1.64 1.63 1.61 1.61 1.60 1.60 1.59 1.59 1.58 1.57 1.56 1.56 1.52 0.96 0.95 0.92 0.91
linear rhabdobranin
(20)
1 2 2 2 1 2 1 14 6
5.8 5.6 5.4 5.2 5.0 4.8 4.6 4.4 4.2 4.0 3.8 3.6 3.4 3.2 3.0 2.8 2.6 2.4 2.2 2.0 1.8 1.6 1.4 1.2 1.0 0.8 0.6 0.4

COSY, D_2O, **20**

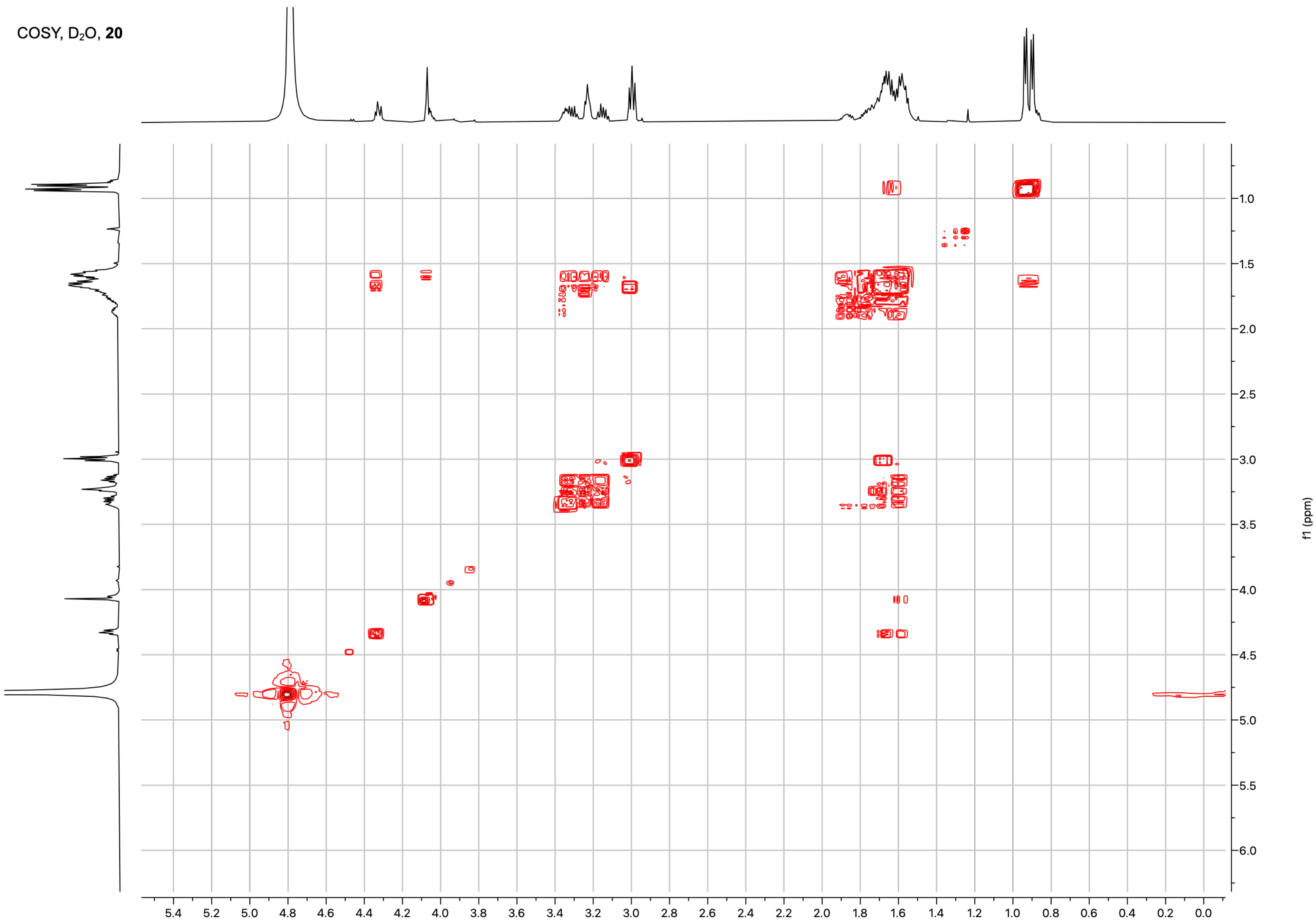

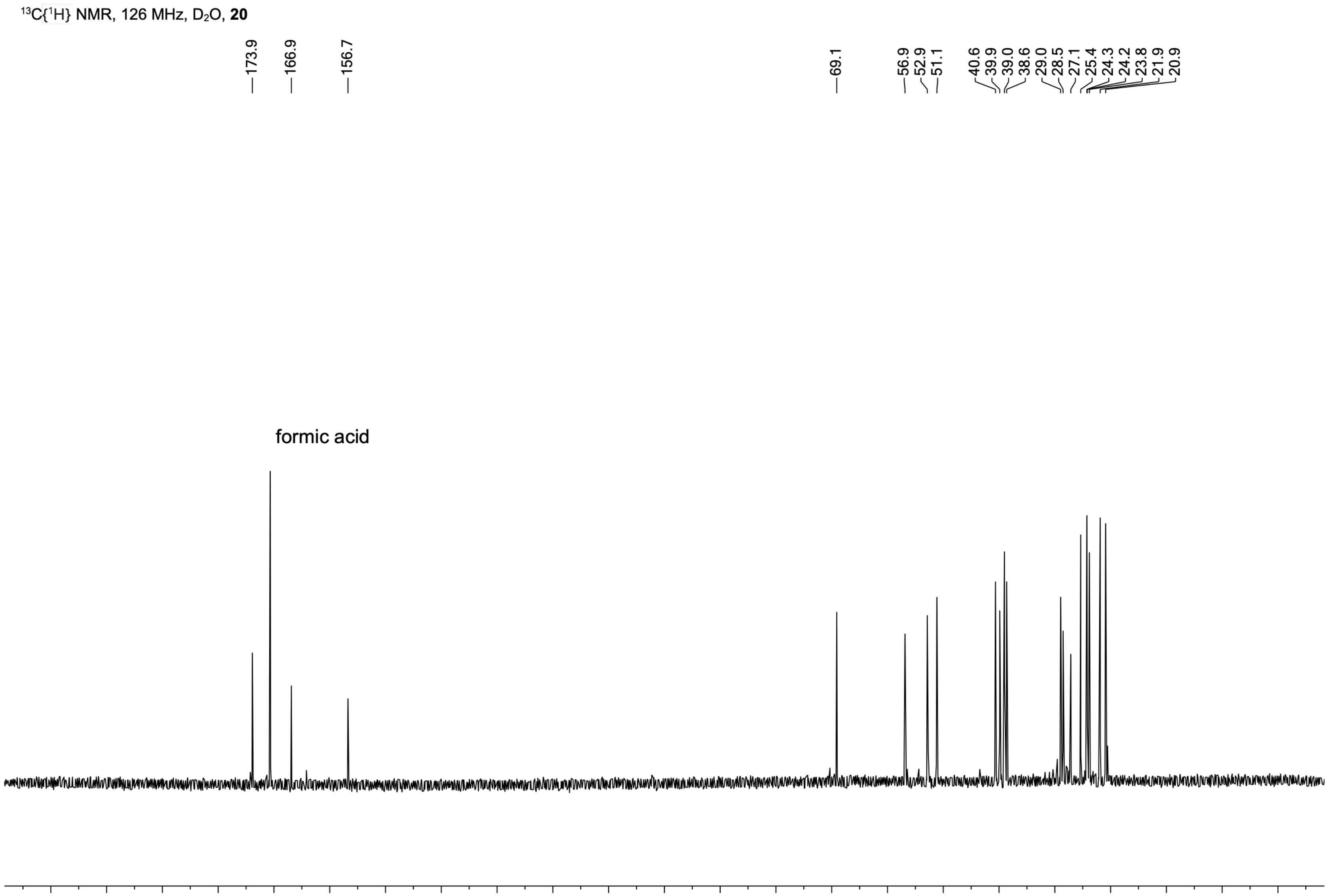
$^{13}C\{^1H\}$ NMR, 126 MHz, D_2O, **20**
173.9
166.9
156.7
69.1
56.9
52.9
51.1
40.6
39.9
39.0
38.6
29.0
28.5
27.1
25.4
24.3
24.2
23.8
21.9
20.9
formic acid
210
200
190
180
170
160
150
140
130
120
110
100
90
80
70
60
50
40
30
20
10
0
-10

HSQC, D_2O, **20**

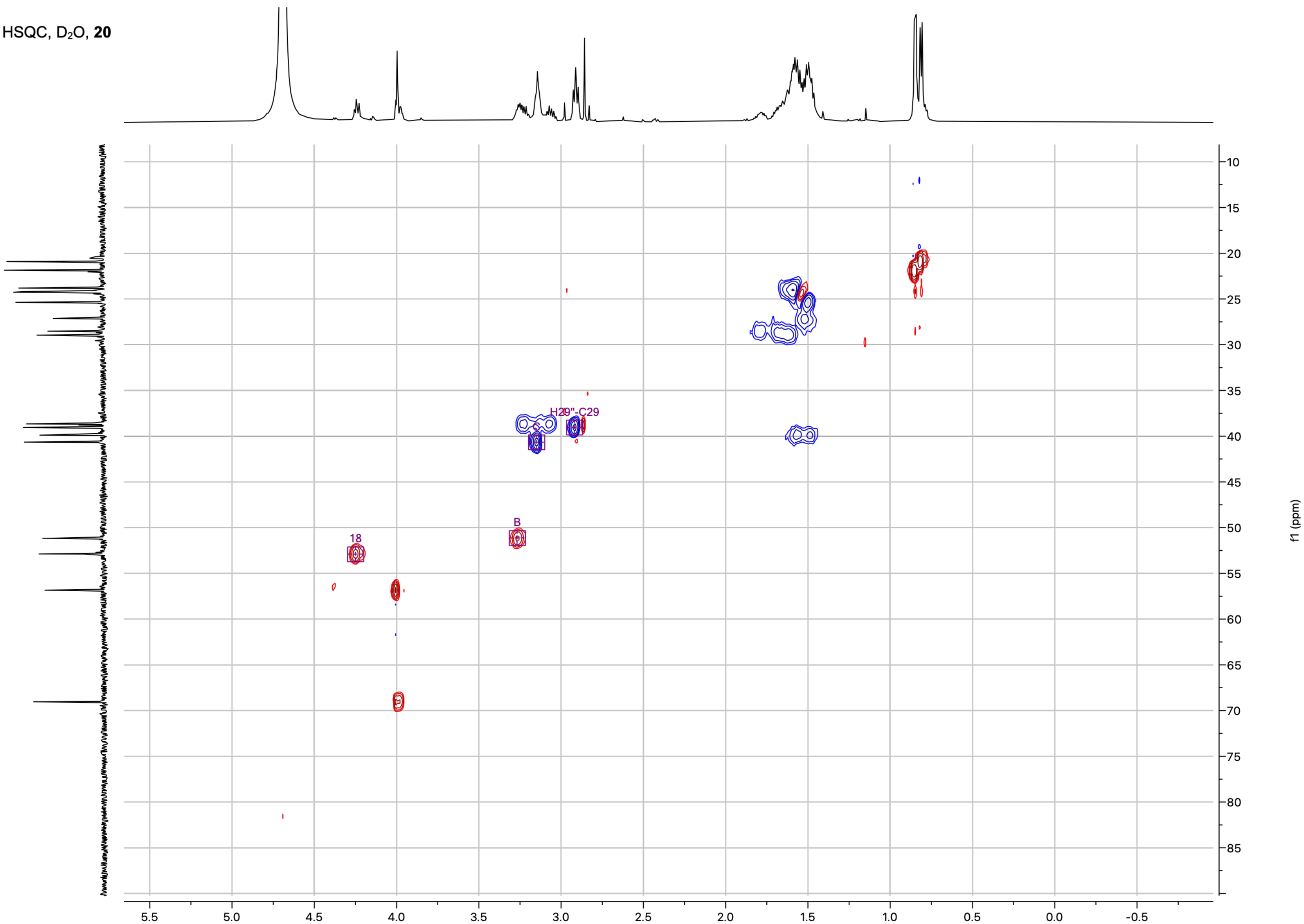

^{1}H NMR, 500 MHz, DMSO-d_6, **S26**

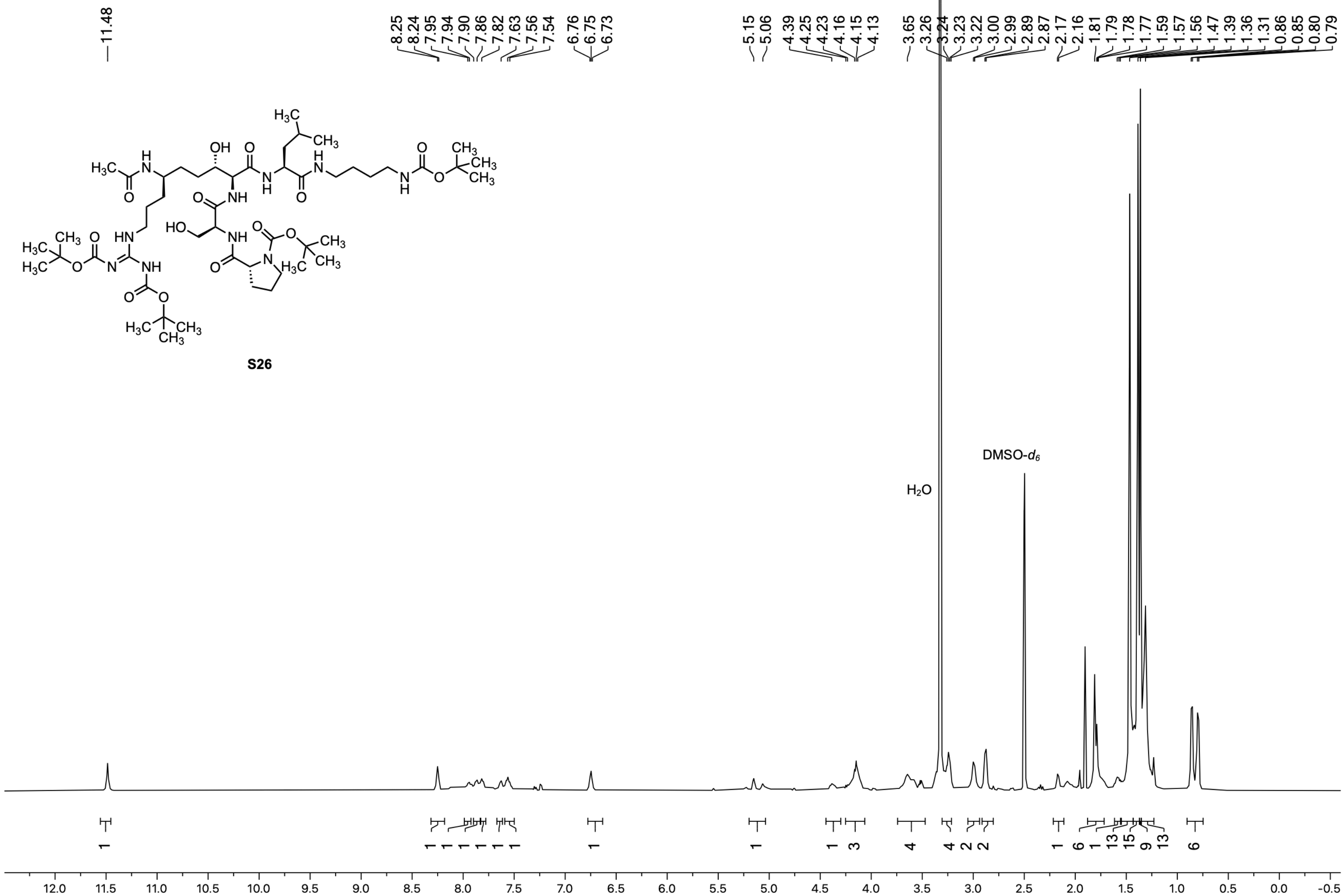

COSY, DMSO-d_6, **S26**

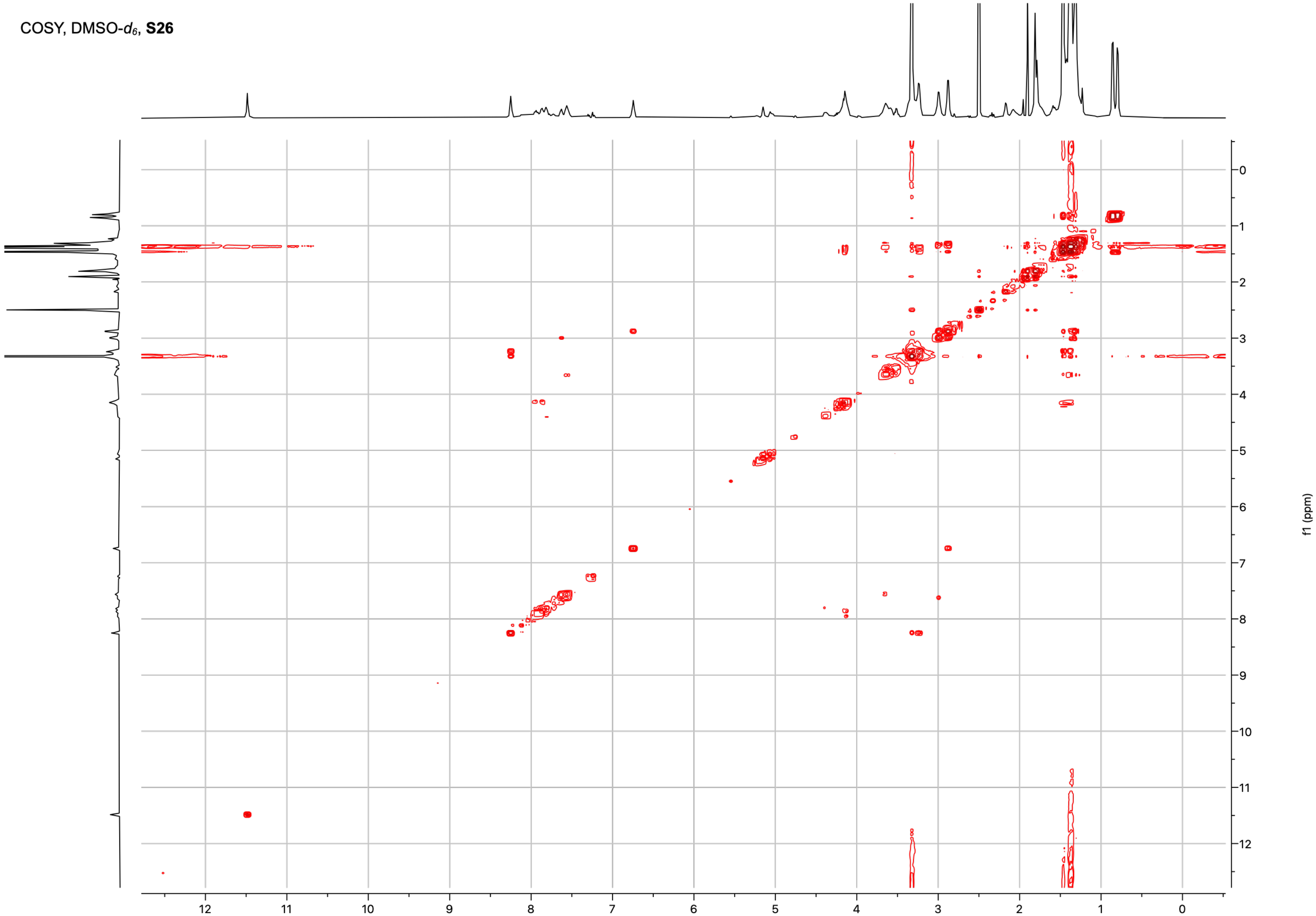

$^{13}C\{^{1}H\}$ NMR, 126 MHz, DMSO-d_6, **S26**

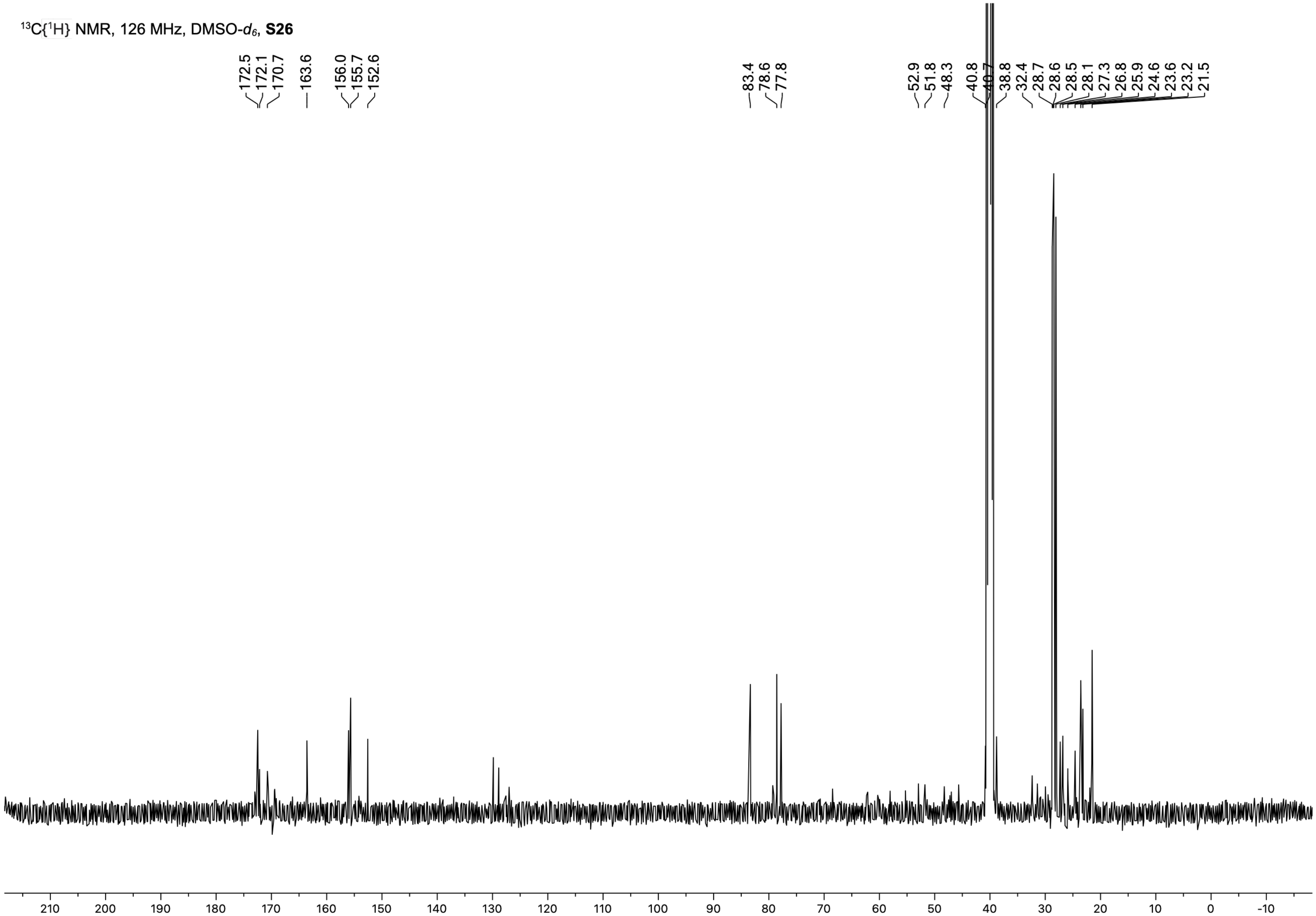

HSQC, DMSO-d_6, **S26**

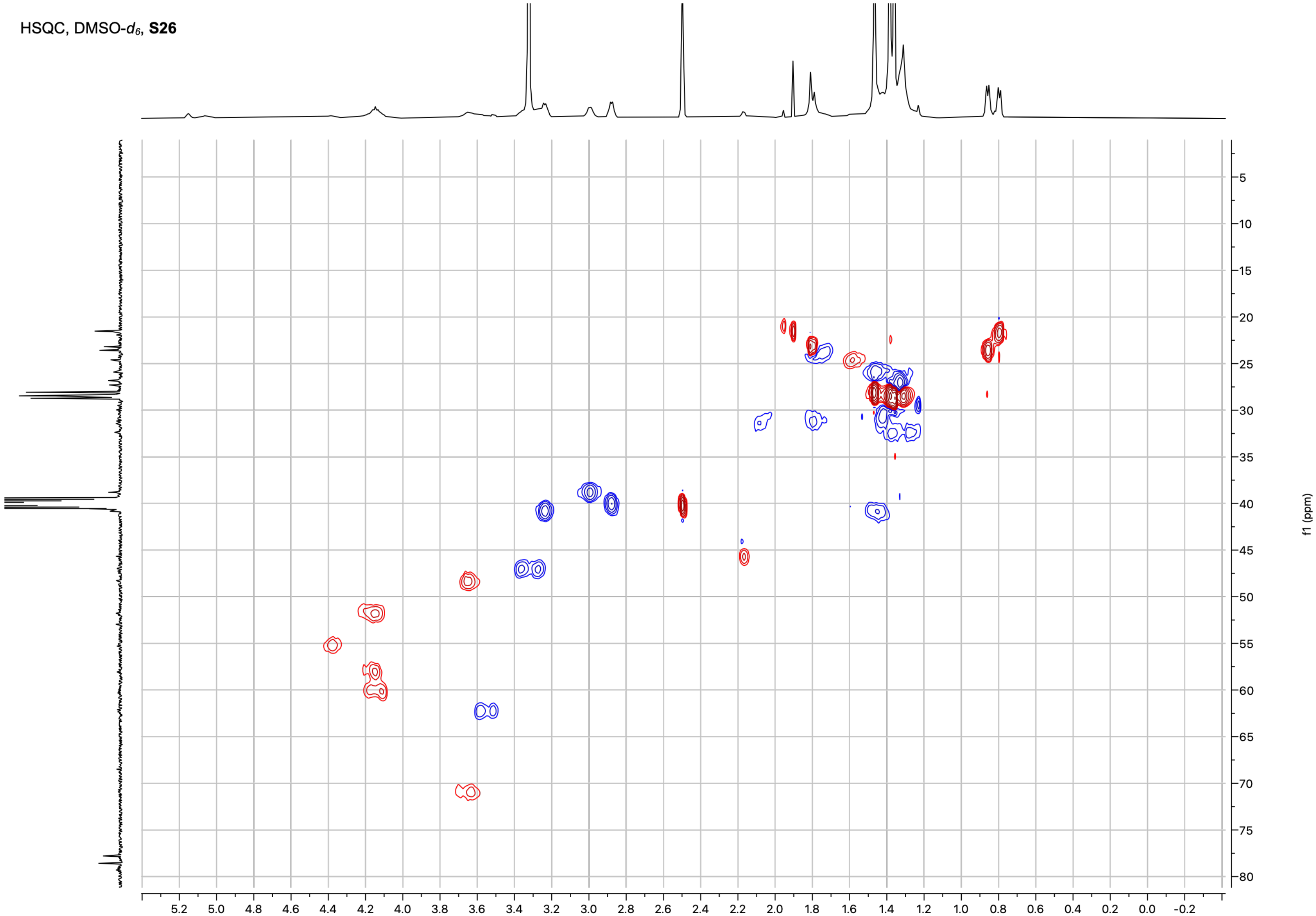

^{1}H NMR, 500 MHz, D_2O, (5*R*)-**3**

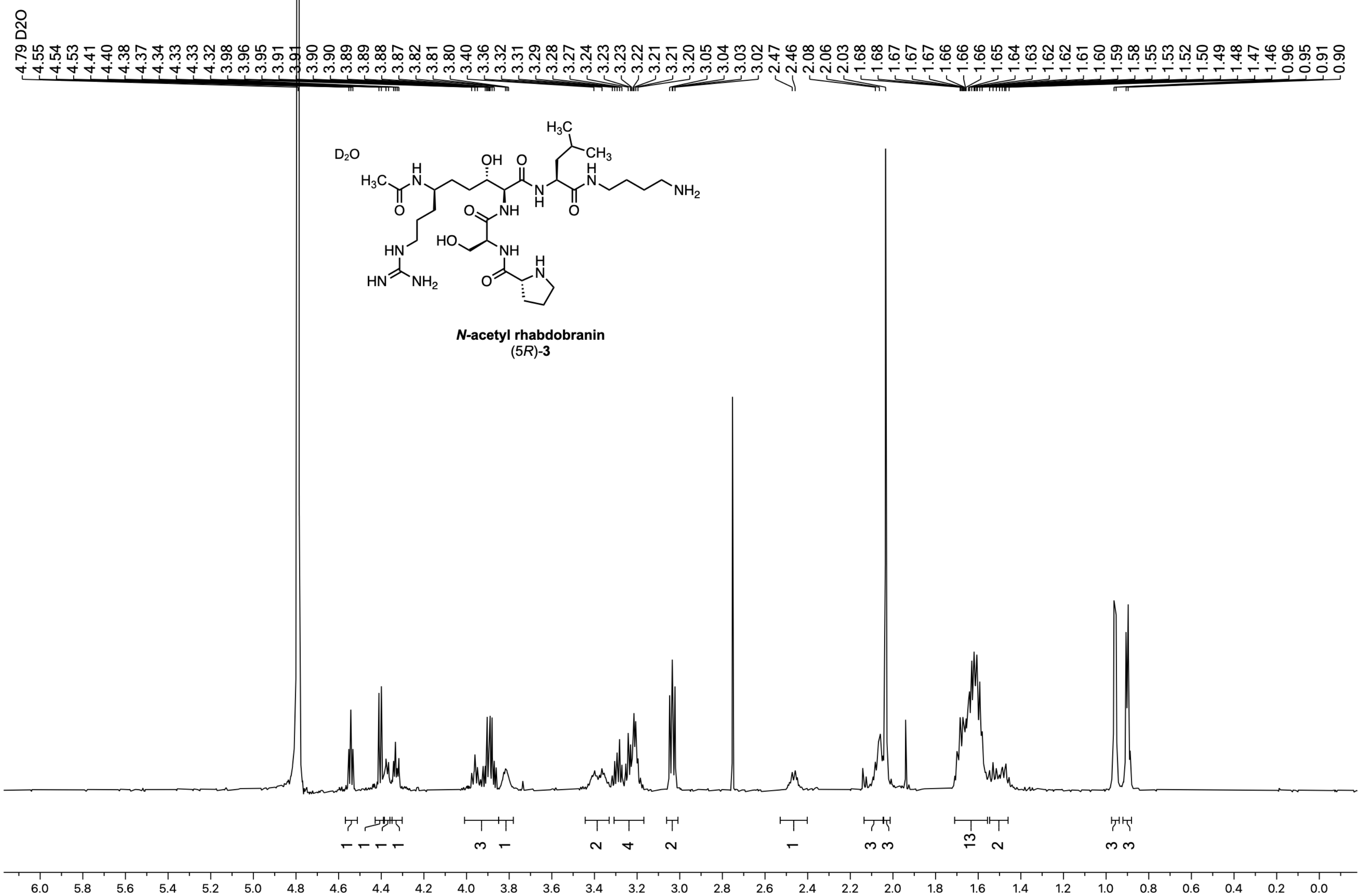

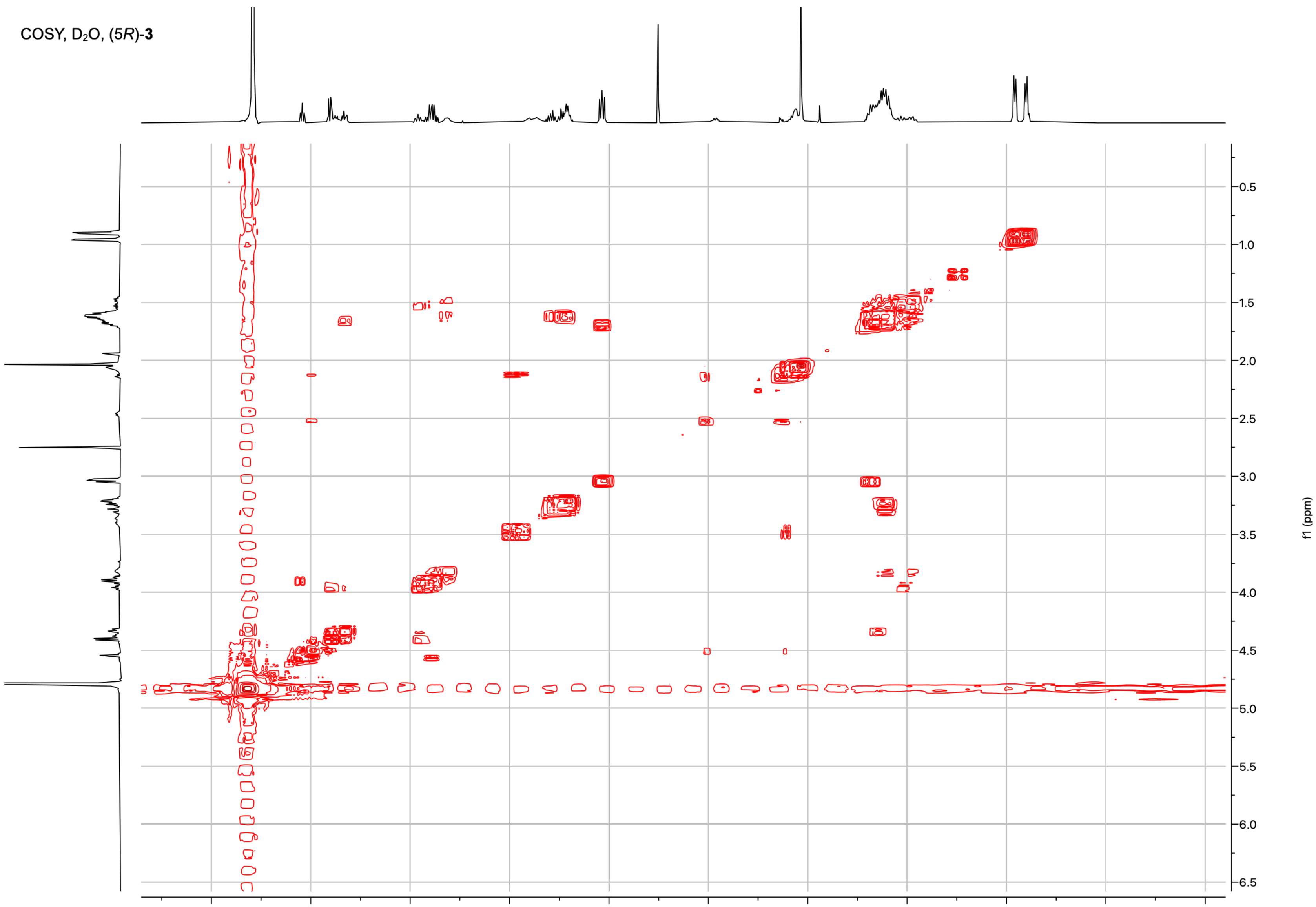
COSY, D2O, (5R)-3
f1 (ppm)
0.5
1.0
1.5
2.0
2.5
3.0
3.5
4.0
4.5
5.0
5.5
6.0
6.5
5.0
4.5
4.0
3.5
3.0
2.5
2.0
1.5
1.0
0.5
0.0

$^{13}C\{^1H\}$ NMR, 126 MHz, D_2O, (5*R*)-**3**

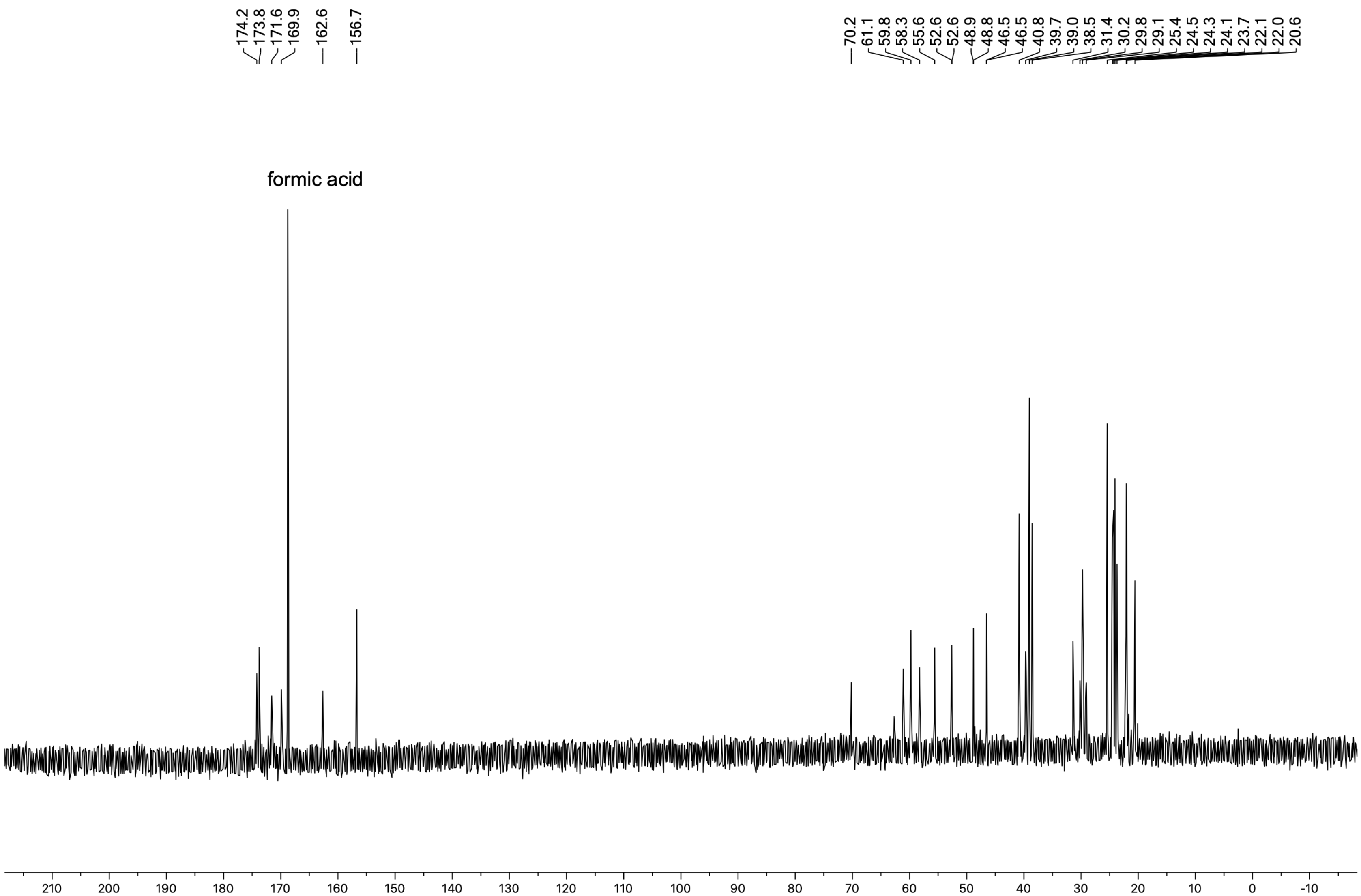

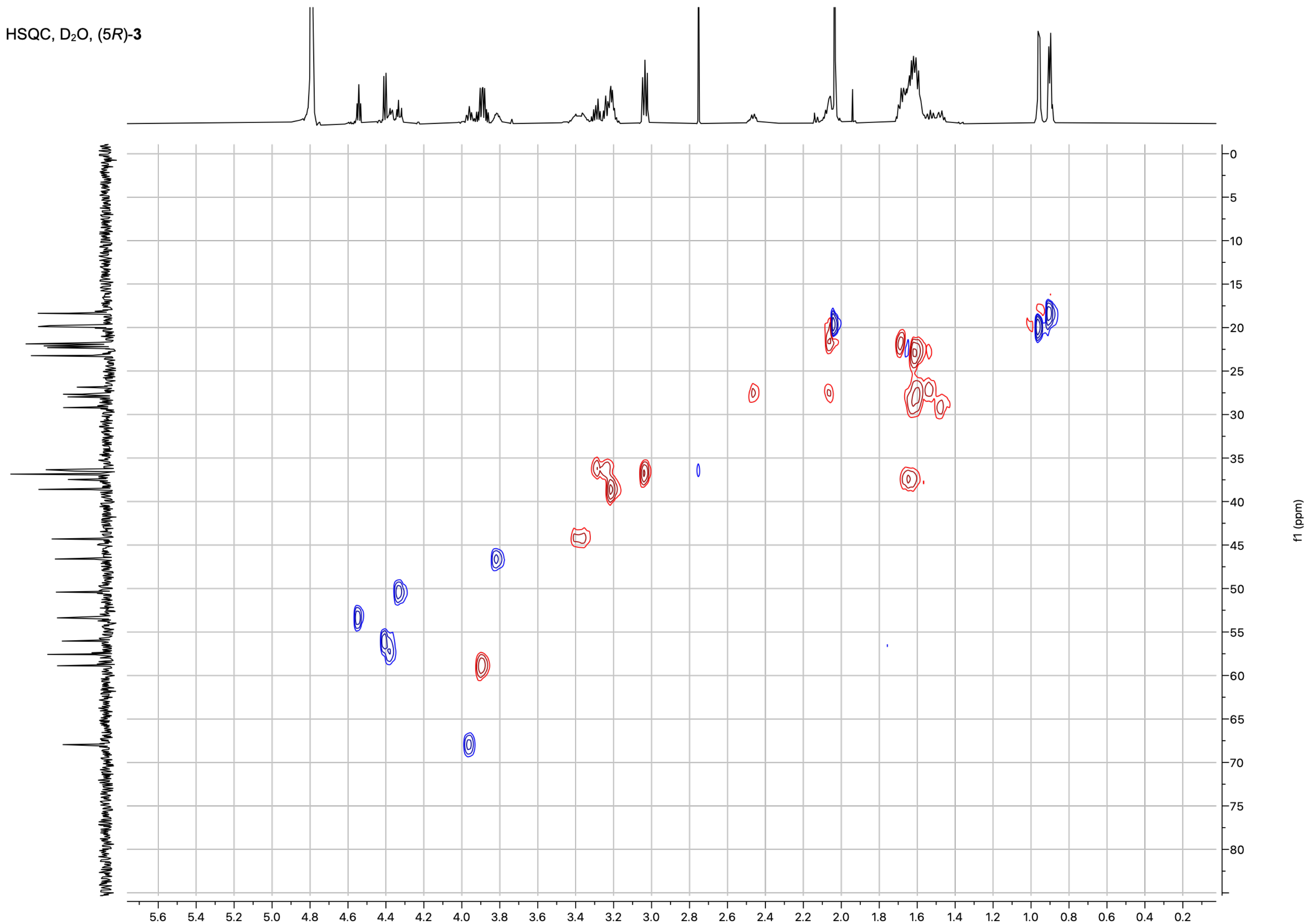
HSQC, D2O, (5R)-3
5.6 5.4 5.2 5.0 4.8 4.6 4.4 4.2 4.0 3.8 3.6 3.4 3.2 3.0 2.8 2.6 2.4 2.2 2.0 1.8 1.6 1.4 1.2 1.0 0.8 0.6 0.4 0.2
0 5 10 15 20 25 30 35 40 45 50 55 60 65 70 75 80
f1 (ppm)

^{1}H NMR, 500 MHz, DMSO-d_6, **S27**

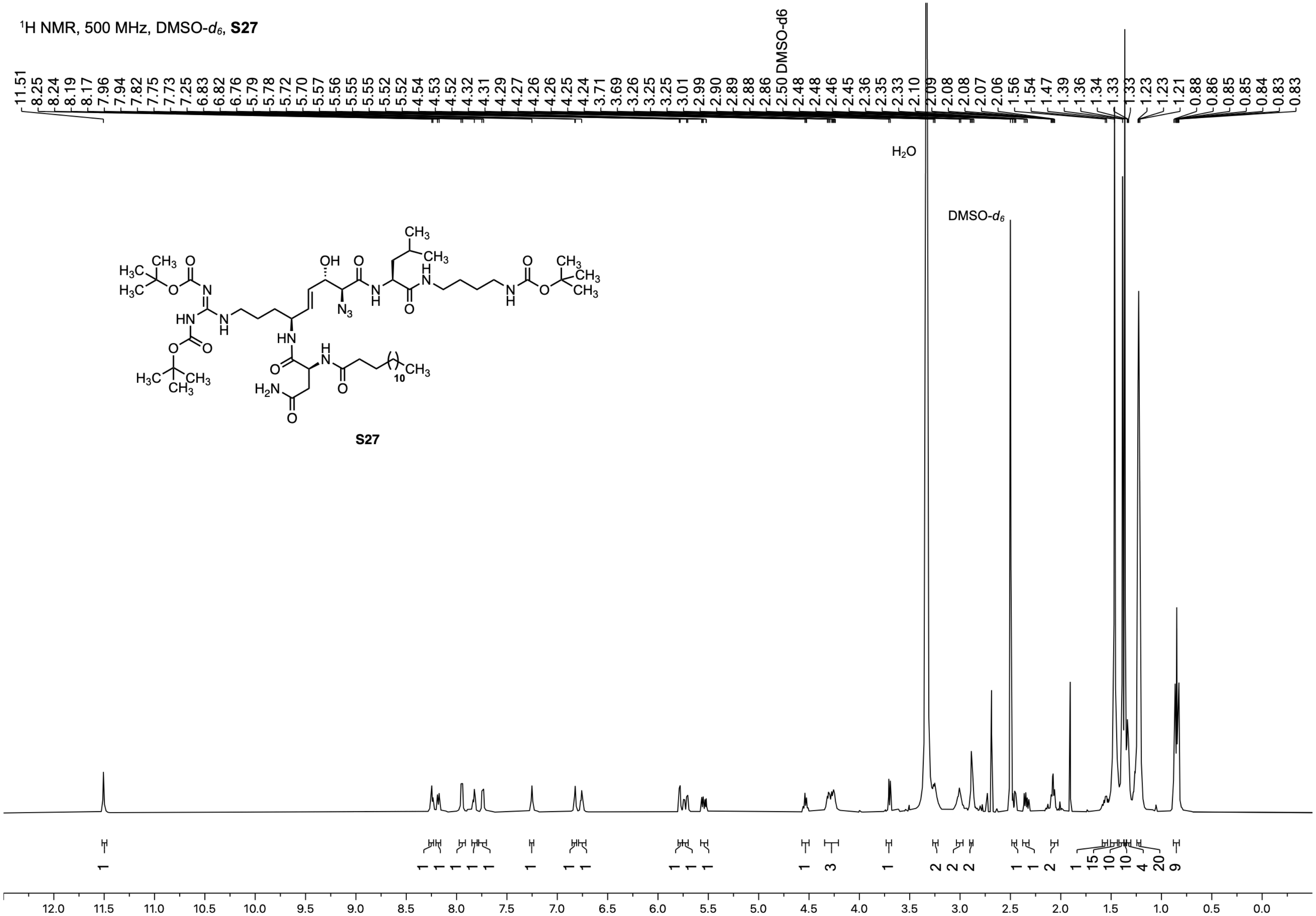

COSY, DMSO-d_6, **S27**

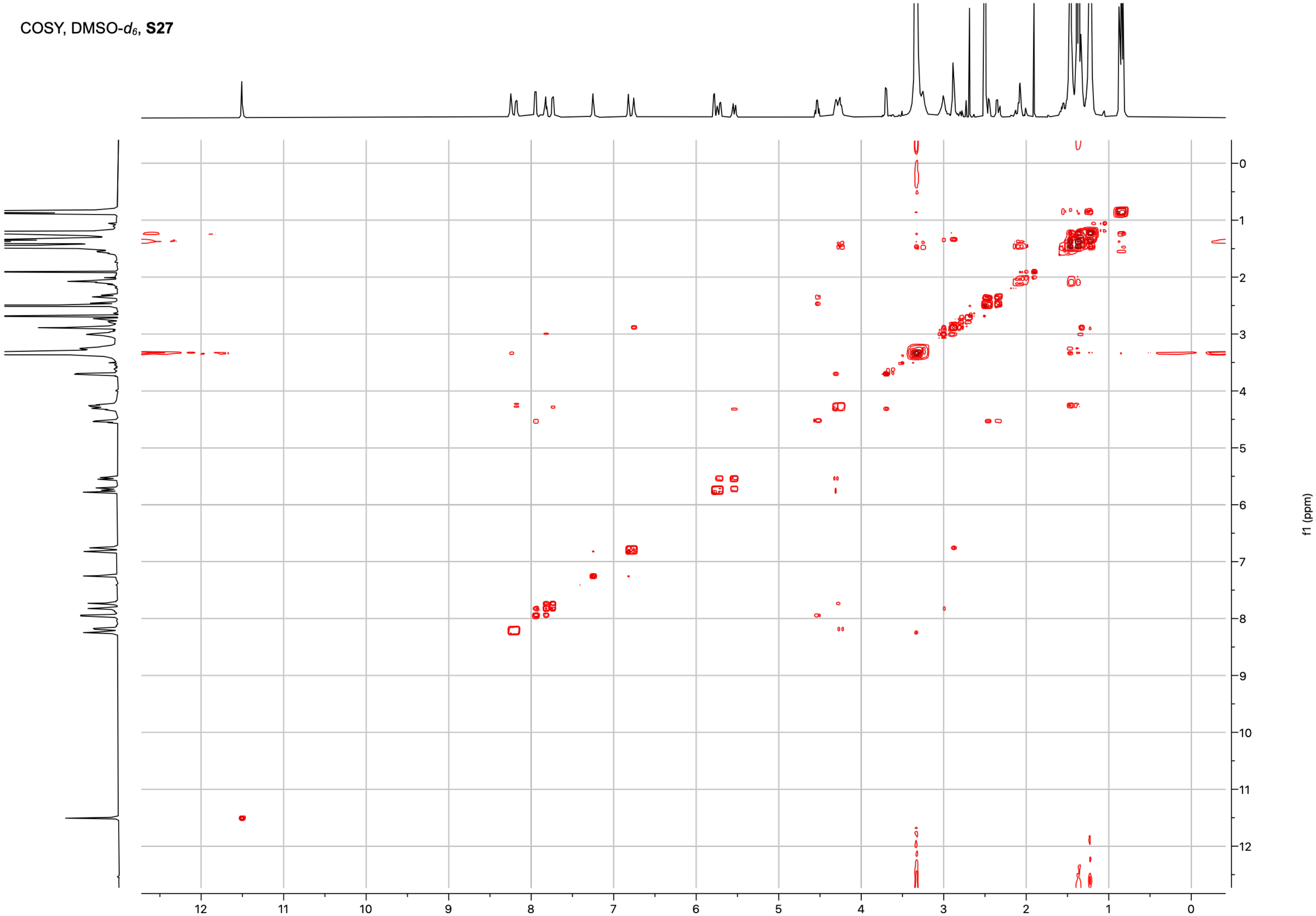

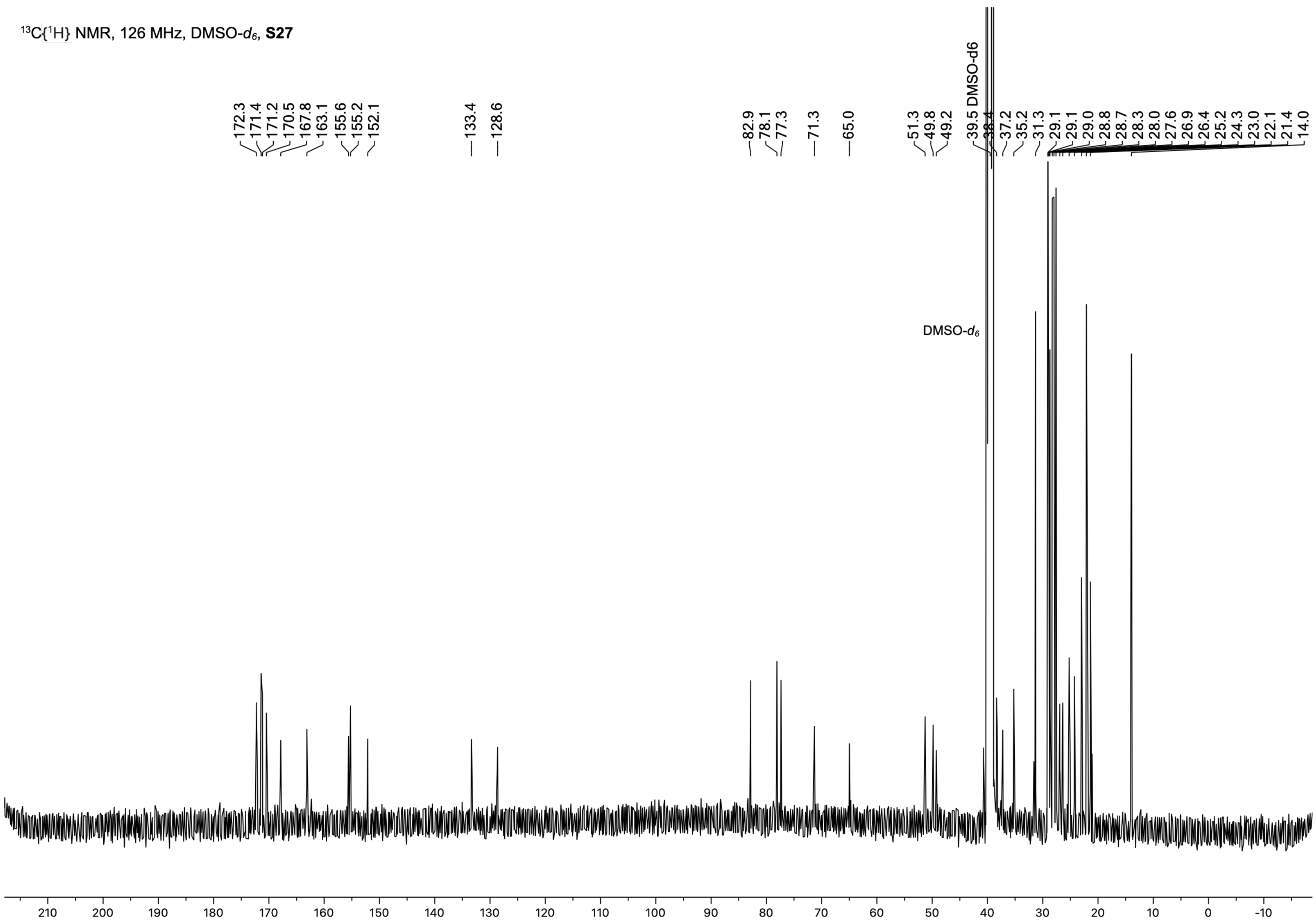

$^{13}C\{^1H\}$ NMR, 126 MHz, DMSO-d_6, **S27**
172.3
171.4
171.2
170.5
167.8
163.1
155.6
155.2
152.1
133.4
128.6
82.9
78.1
77.3
71.3
65.0
51.3
49.8
49.2
39.5 DMSO-d6
38.4
37.2
35.2
31.3
29.1
29.1
29.0
28.8
28.7
28.3
28.0
27.6
26.9
26.4
25.2
24.3
23.0
22.1
21.4
14.0
DMSO-d_6
210 200 190 180 170 160 150 140 130 120 110 100 90 80 70 60 50 40 30 20 10 0 -10

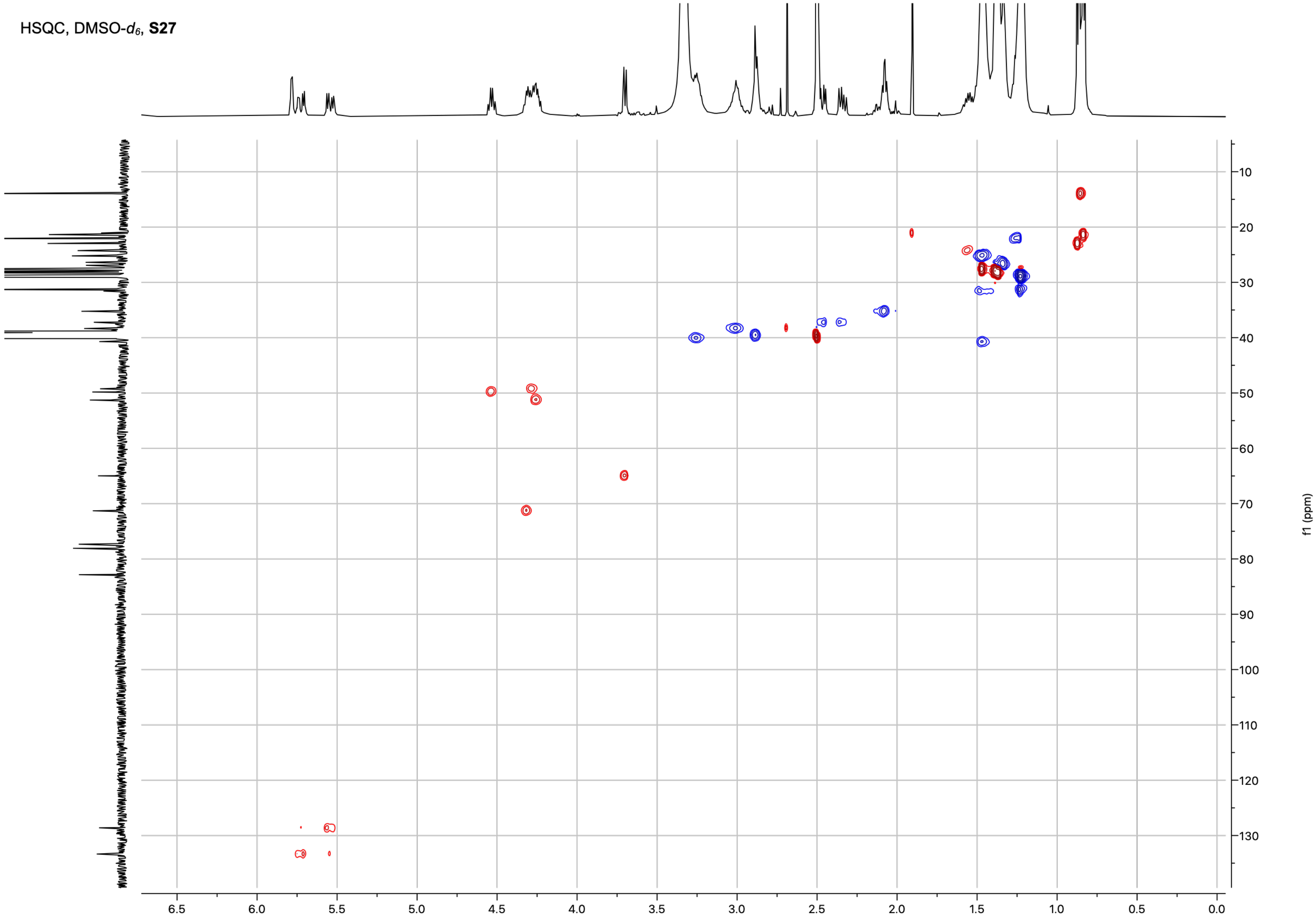
HSQC, DMSO-d_6, **S27**
f1 (ppm)
10
20
30
40
50
60
70
80
90
100
110
120
130
6.5
6.0
5.5
5.0
4.5
4.0
3.5
3.0
2.5
2.0
1.5
1.0
0.5
0.0

^{1}H NMR, 500 MHz, DMSO-d_6, **S28**

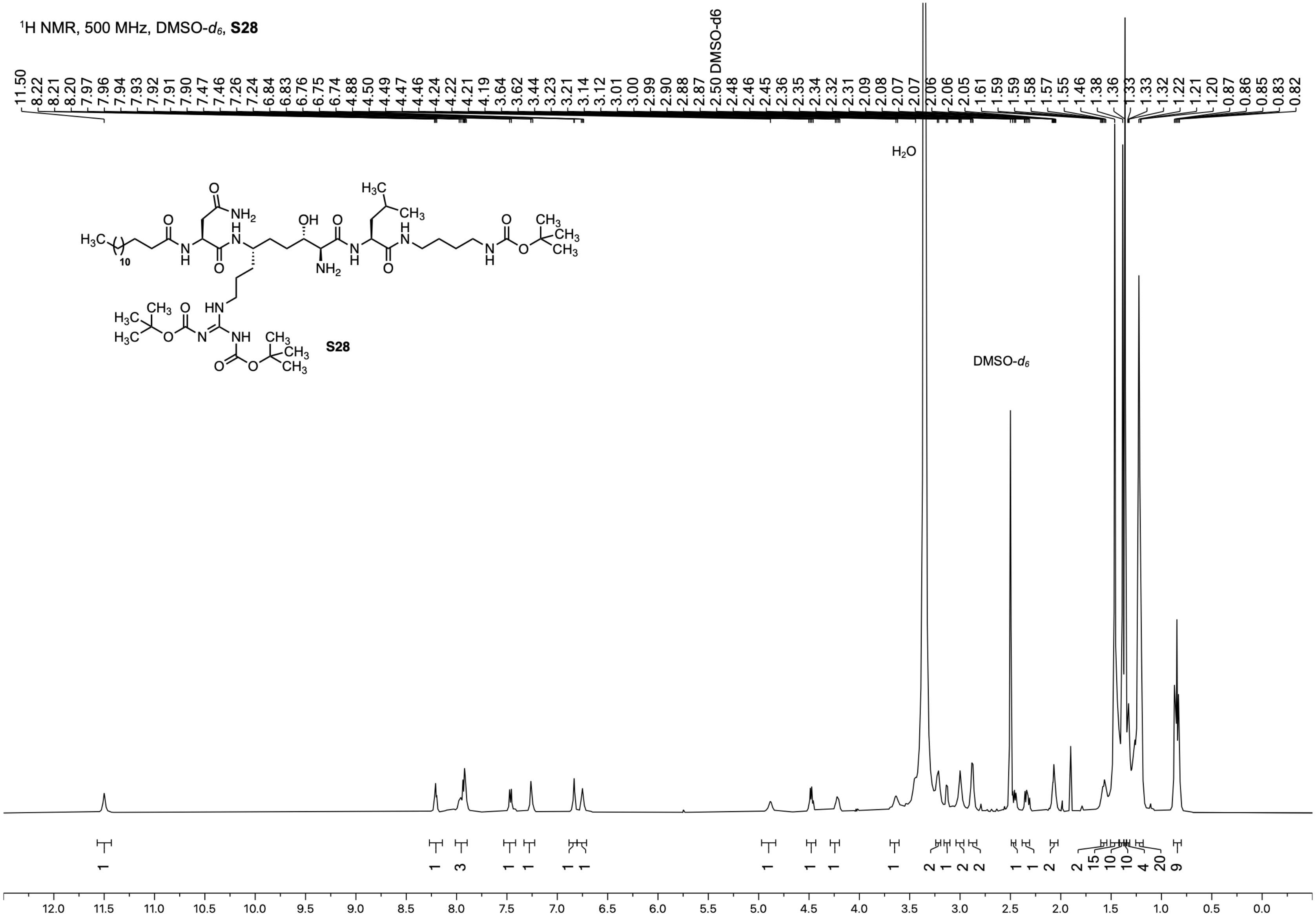

COSY, DMSO-d_6, **S28**

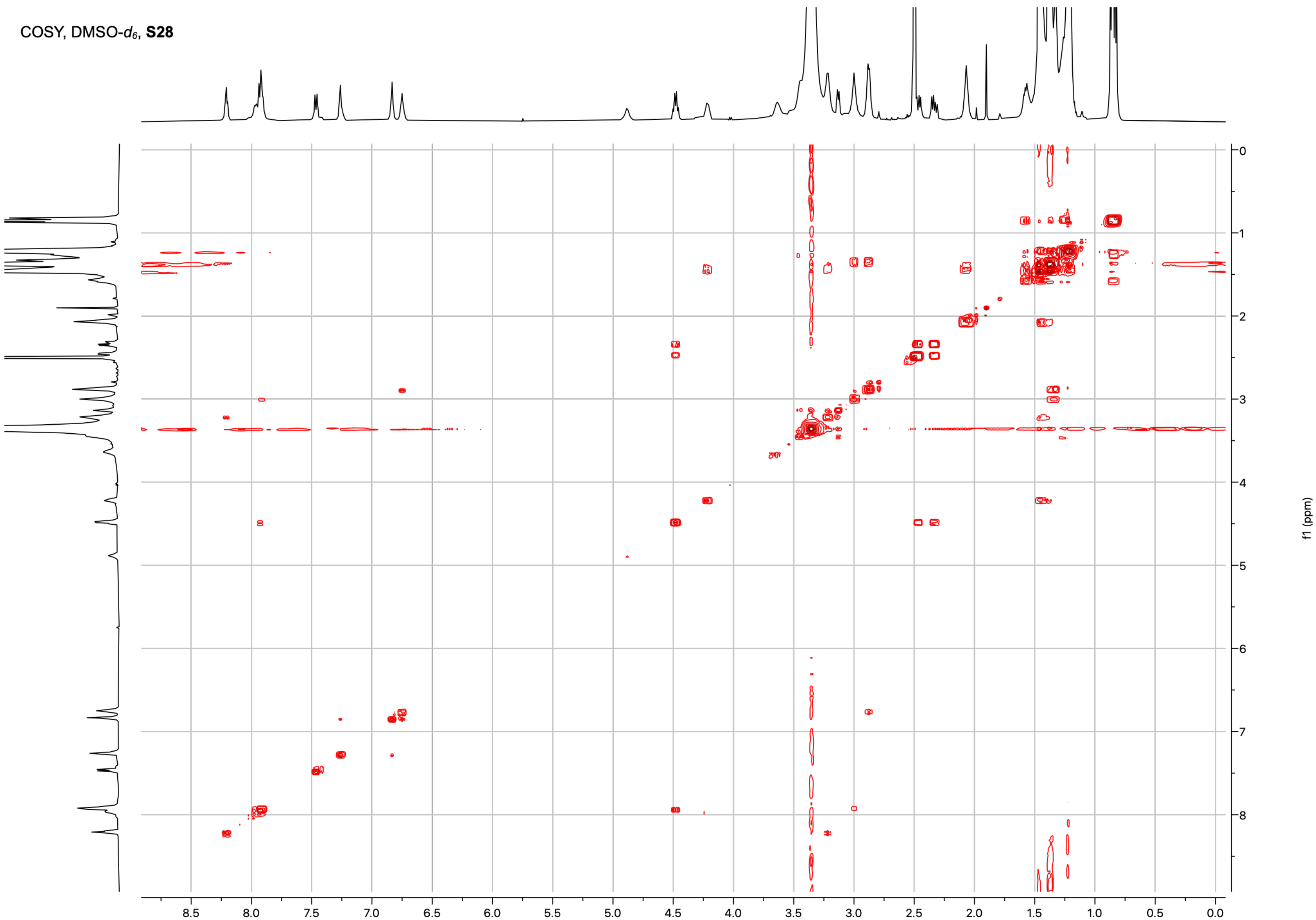

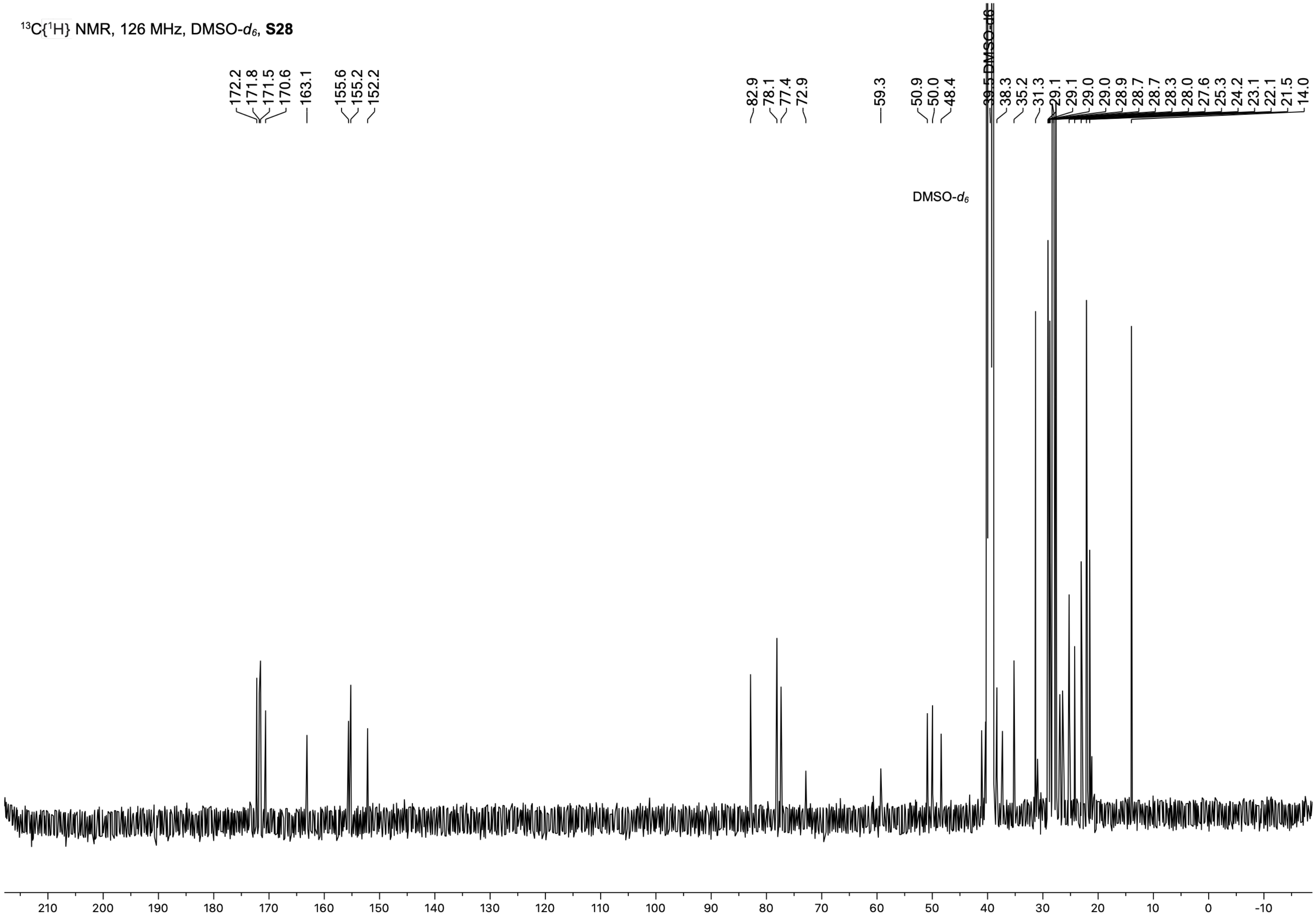

$^{13}C\{^{1}H\}$ NMR, 126 MHz, DMSO-d_6, **S28**
172.2
171.8
171.5
170.6
163.1
155.6
155.2
152.2
82.9
78.1
77.4
72.9
59.3
50.9
50.0
48.4
39.5 DMSO-d6
38.3
35.2
31.3
29.1
29.1
29.0
29.0
28.9
28.7
28.7
28.3
28.0
27.6
25.3
24.2
23.1
22.1
21.5
14.0
DMSO-d_6
210
200
190
180
170
160
150
140
130
120
110
100
90
80
70
60
50
40
30
20
10
0
-10

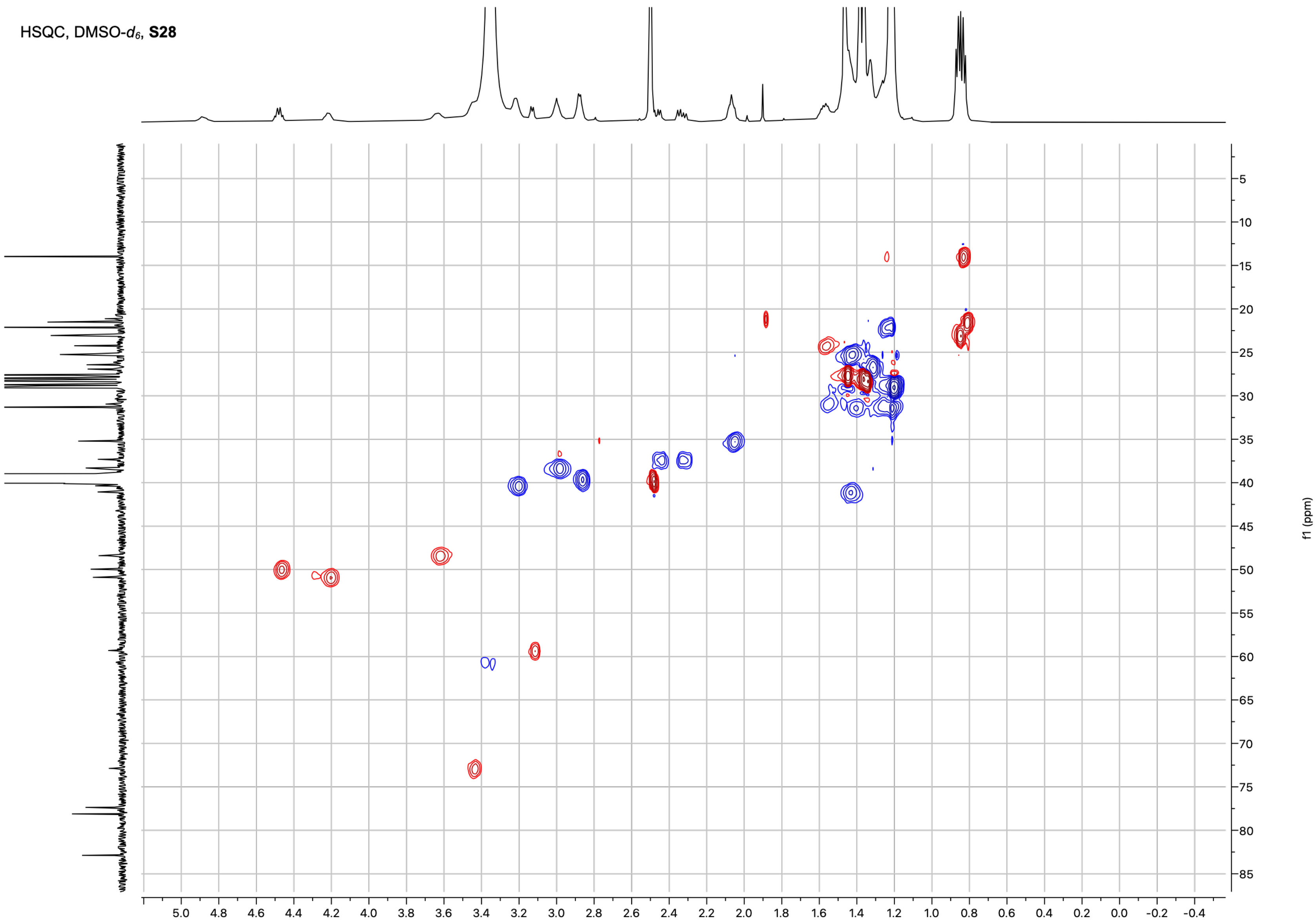
HSQC, DMSO-d_6, **S28**
f1 (ppm)

^{1}H NMR, 500 MHz, DMF-d_7, **S29**

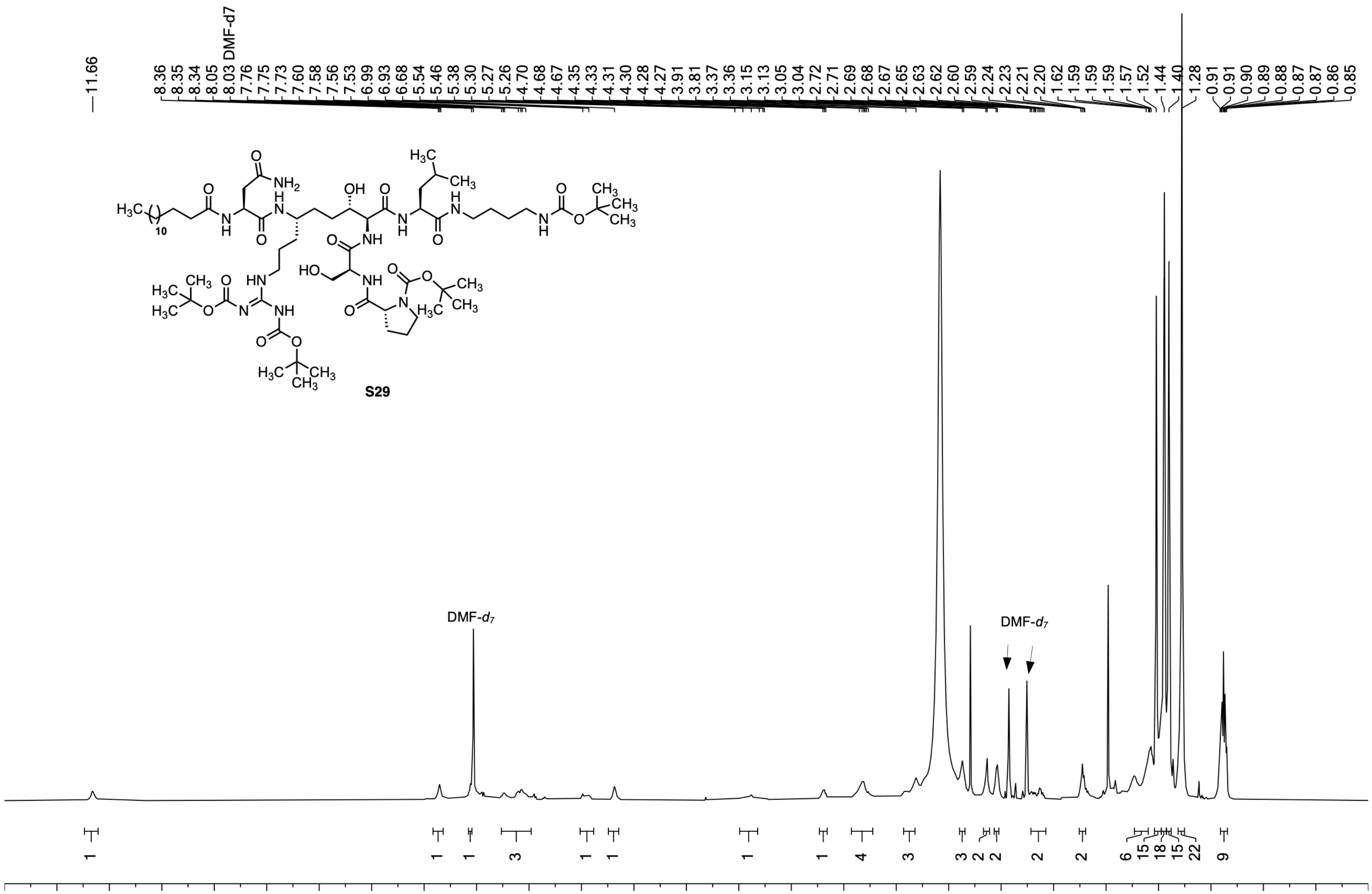

COSY, DMF-d_7, **S29**

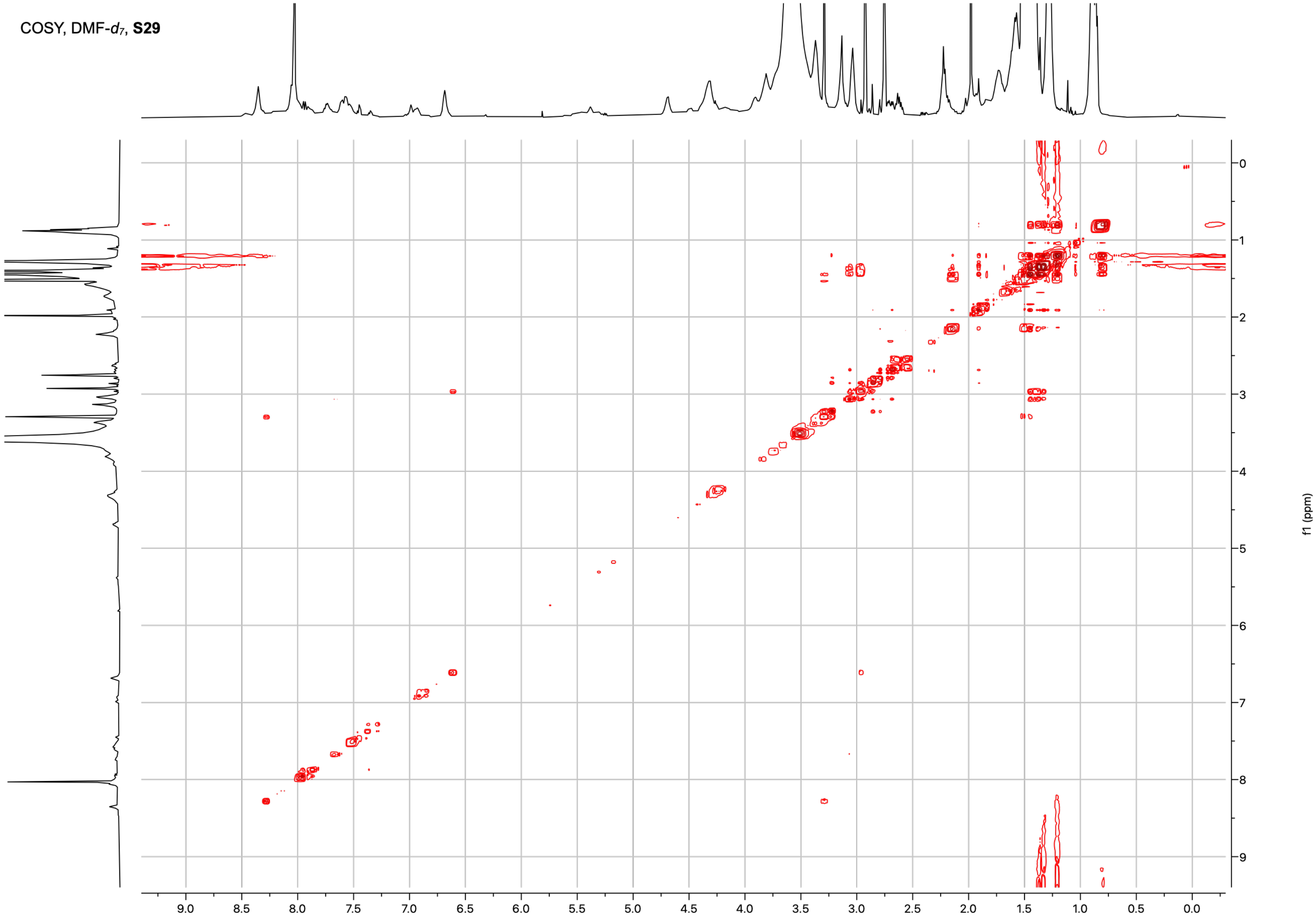

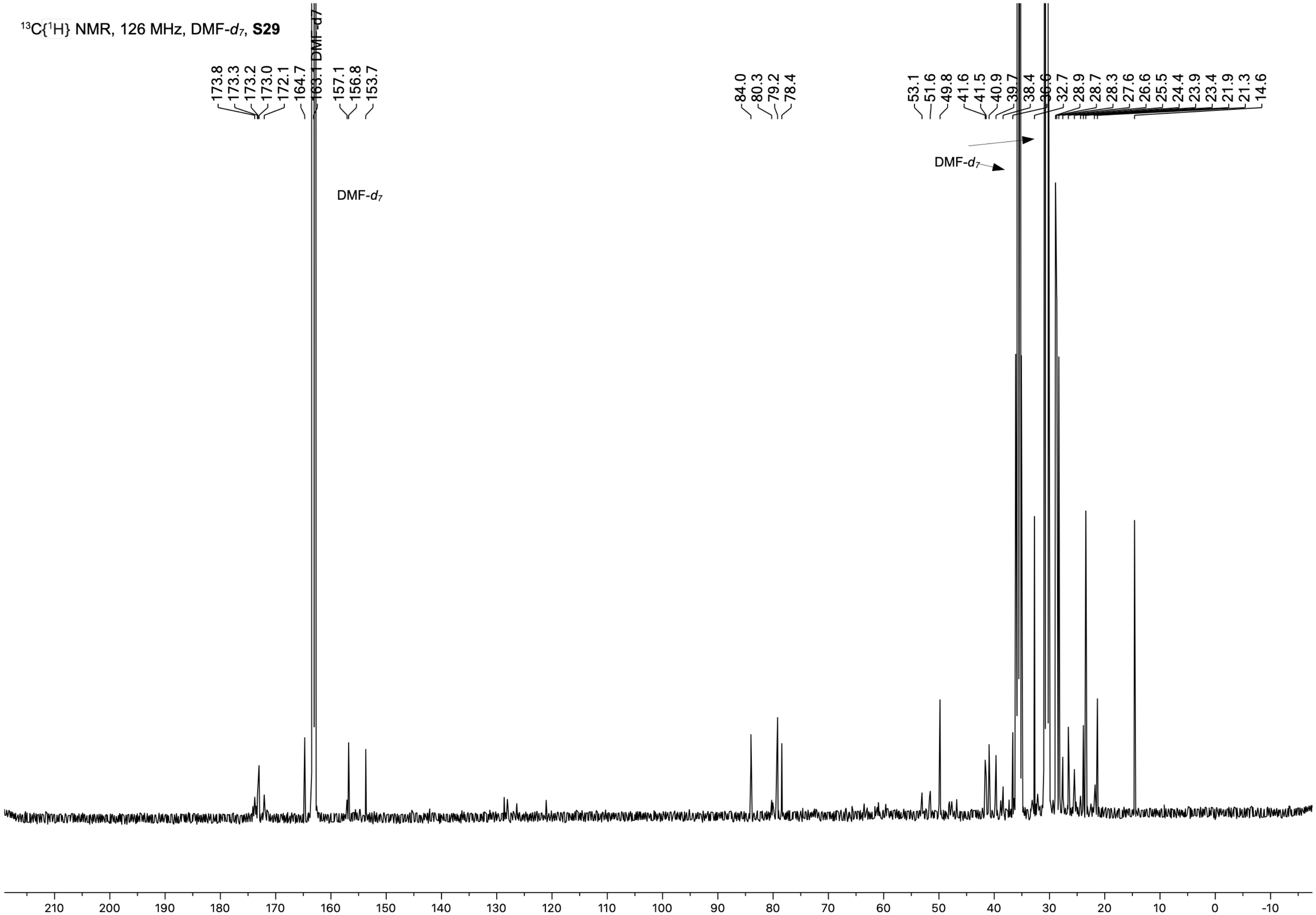

$^{13}C\{^1H\}$ NMR, 126 MHz, DMF-d_7, **S29**
173.8
173.3
173.2
173.0
172.1
164.7
163.1 DMF-d7
157.1
156.8
153.7
84.0
80.3
79.2
78.4
53.1
51.6
49.8
41.6
41.5
40.9
39.7
38.4
36.6
32.7
28.9
28.7
28.3
27.6
26.6
25.5
24.4
23.9
23.4
21.9
21.3
14.6
DMF-d_7
DMF-d_7
210
200
190
180
170
160
150
140
130
120
110
100
90
80
70
60
50
40
30
20
10
0
-10

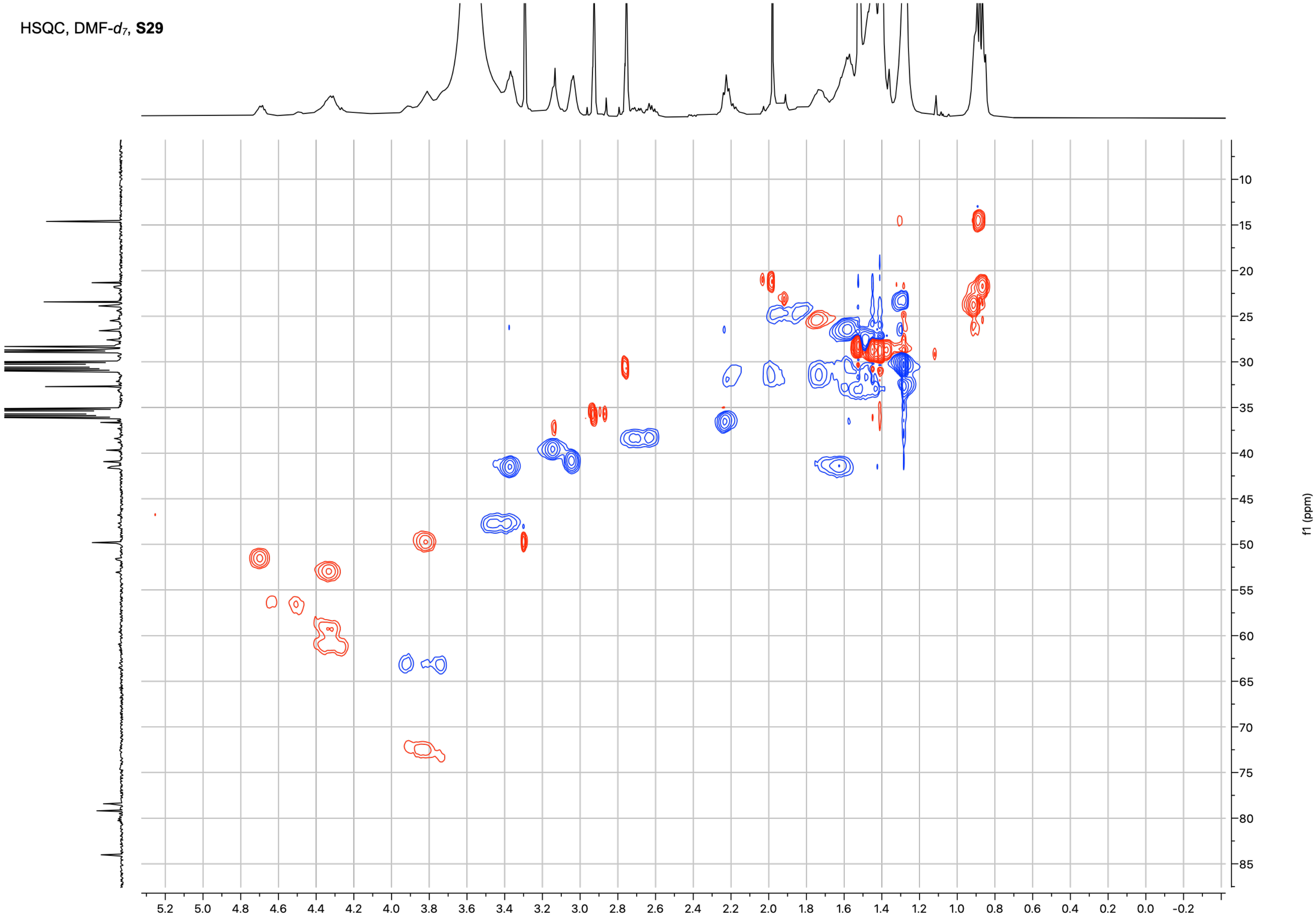
HSQC, DMF-d_7, **S29**
f1 (ppm)

^1H NMR, 500 MHz, DMSO-d_6, **S30**

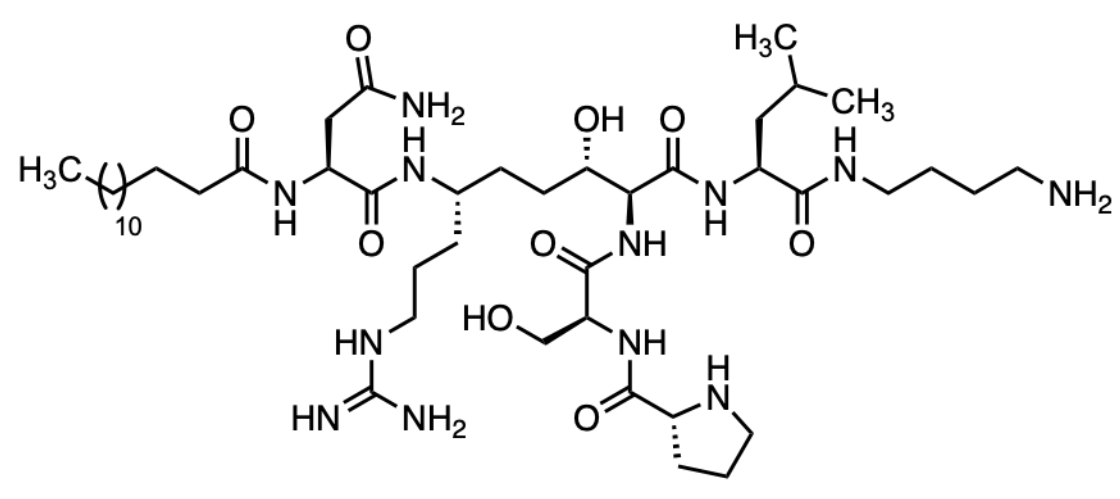

L-Asn-L-Arg Pre-rhabdobranin B
(S30)

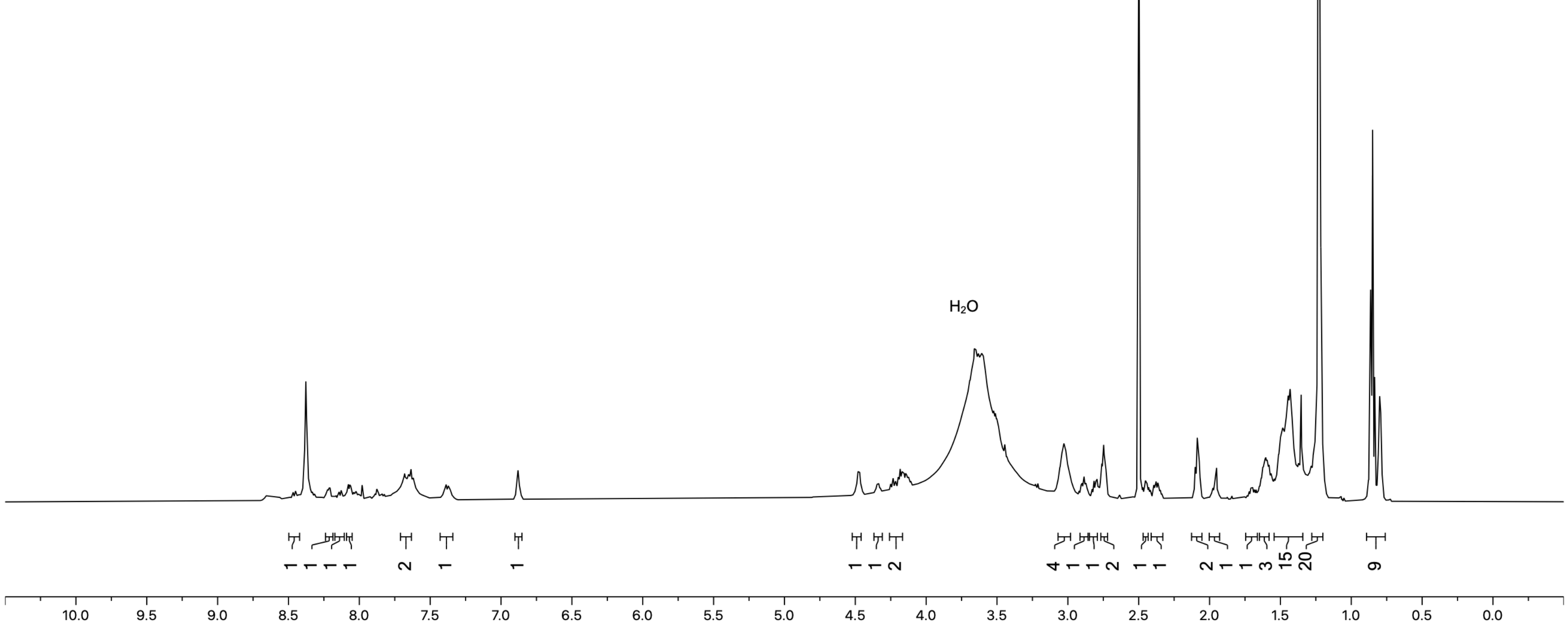

COSY, DMSO-d_6, **S30**

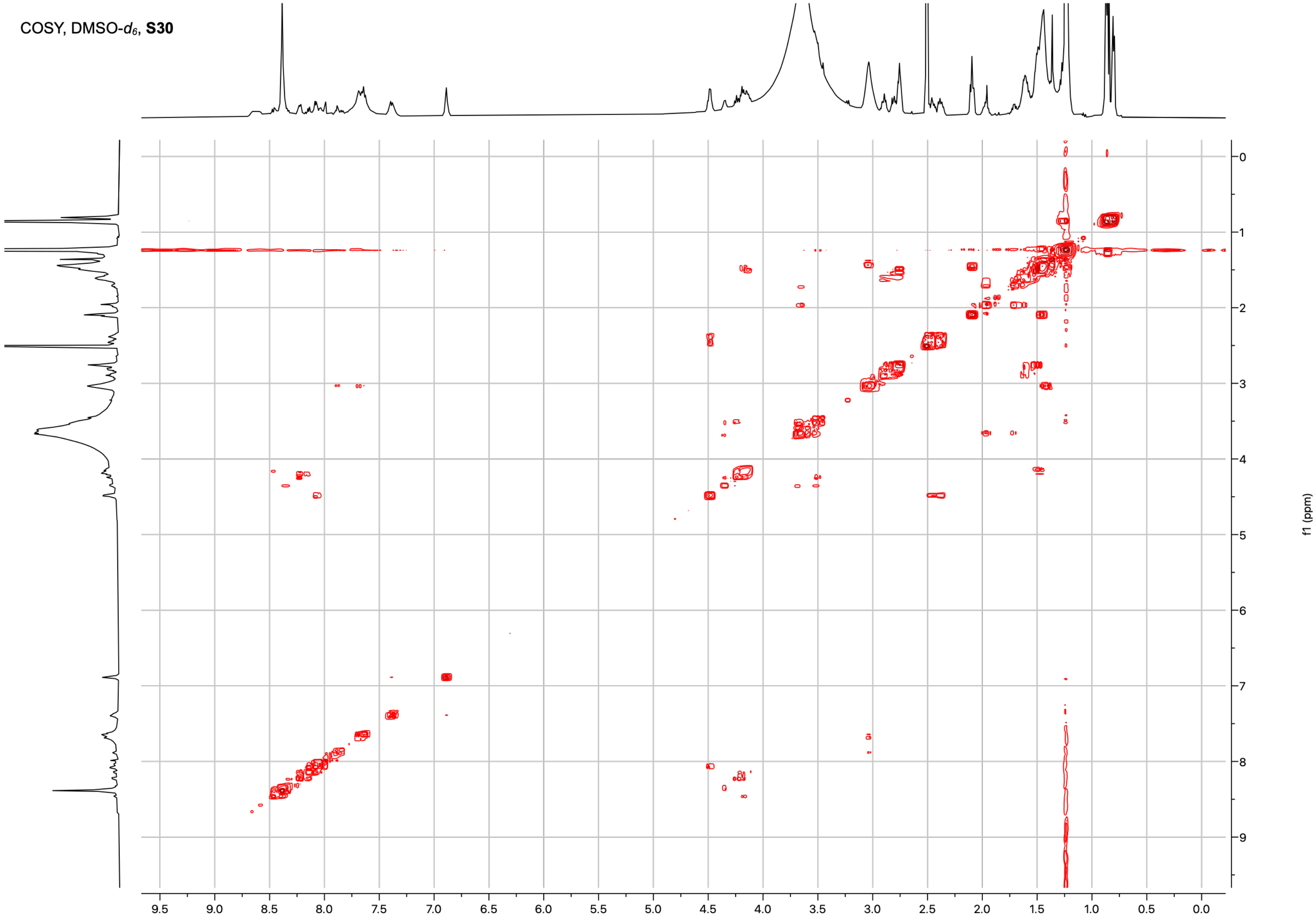

$^{13}C\{^{1}H\}$ NMR, 126 MHz, DMSO-d_6, **S30**

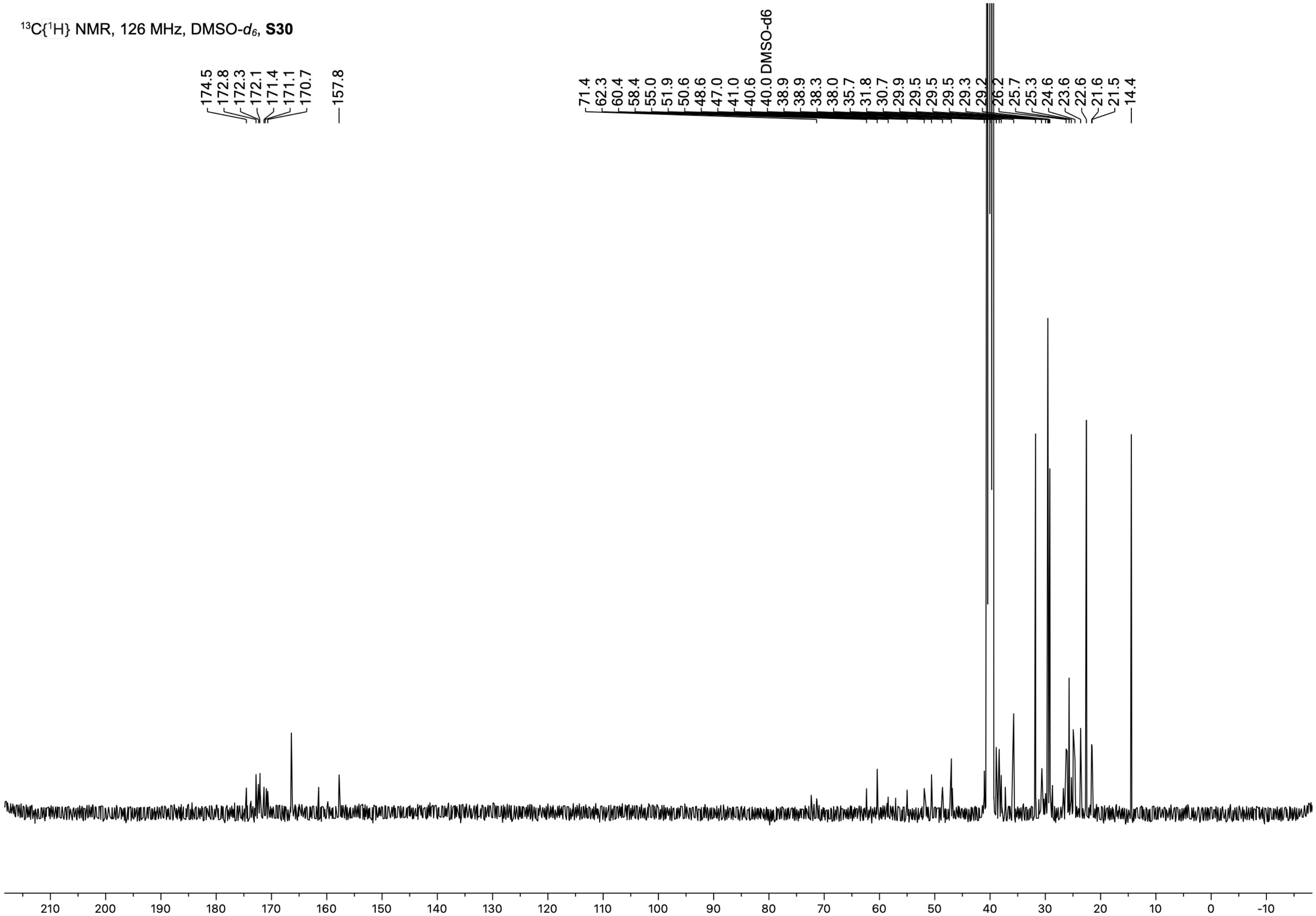

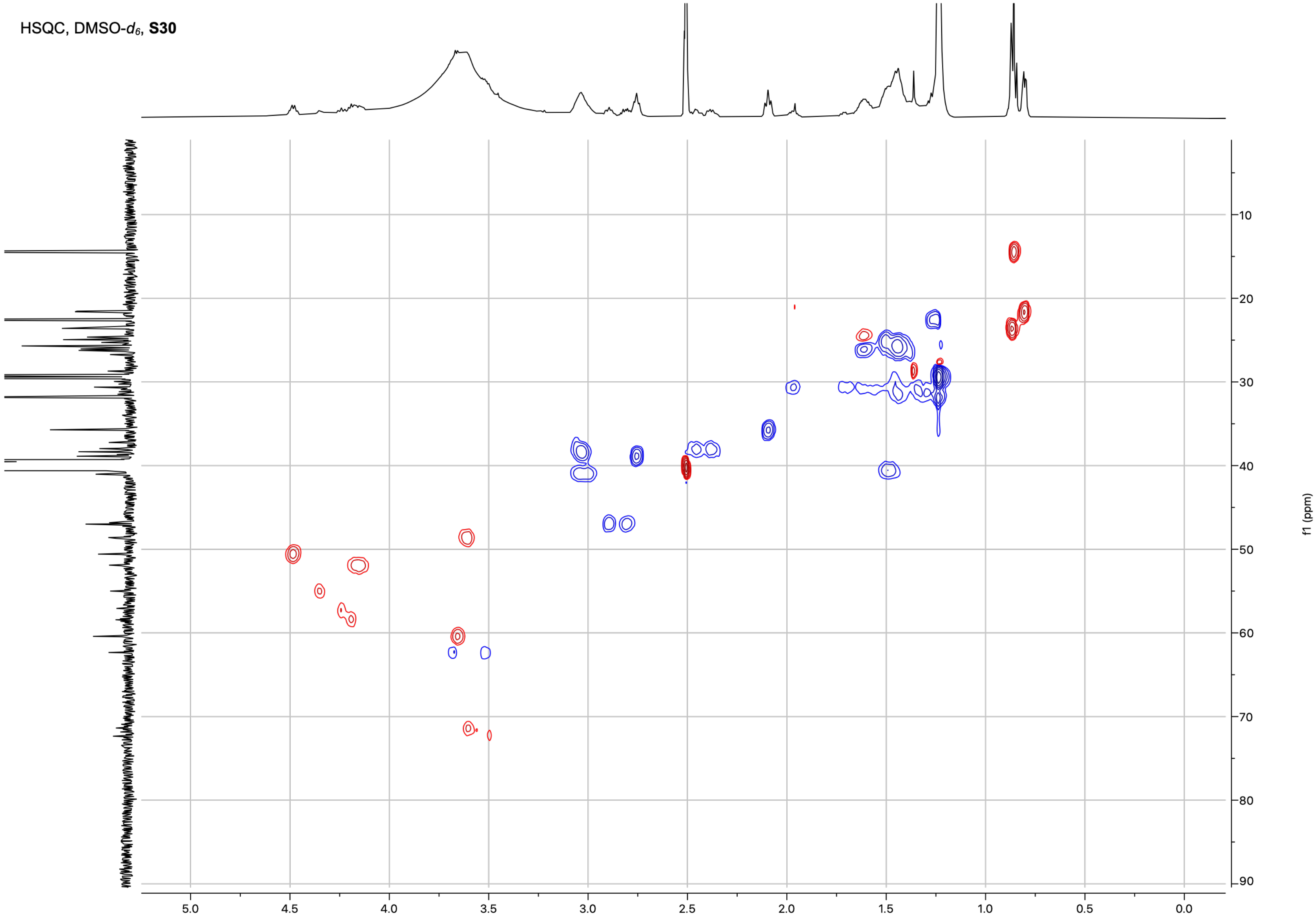
HSQC, DMSO-d_6, **S30**
f1 (ppm)
10
20
30
40
50
60
70
80
90
5.0
4.5
4.0
3.5
3.0
2.5
2.0
1.5
1.0
0.5
0.0

References

(1) Shi, Y.-M.; Hirschmann, M.; Shi, Y.-N.; Ahmed, S.; Abebew, D.; Tobias, N. J.; Grün, P.; Crames, J. J.; Pöschel, L.; Kuttenlochner, W.; Richter, C.; Herrmann, J.; Müller, R.; Thanwisai, A.; Pidot, S. J.; Stinear, T. P.; Groll, M.; Kim, Y.; Bode, H. B. Global analysis of biosynthetic gene clusters reveals conserved and unique natural products in entomopathogenic nematode-symbiotic bacteria. *Nat. Chem.* **2022**, *14* (6), 701-712. DOI. https://doi.org/10.1038/s41557-022-00923-2.

(2) Gonschorek, P.; Wilson, C. S.; Schelhas, C.; Bozhüyük, K. A. J.; Grün, P.; Bode, H. B. Split inteins for generating combinatorial non-ribosomal peptide libraries. *bioRxiv* **2025**, 2025.2010.2002.680031. DOI. https://doi.org/10.1101/2025.10.02.680031.

(3) Aldrich, L. N.; Berry, C. B.; Bates, B. S.; Konkol, L. C.; So, M.; Lindsley, C. W. Towards the Total Synthesis of Marineosin A: Construction of the Macrocyclic Pyrrole and an Advanced, Functionalized Spiroaminal Model. *Eur. J. Org. Chem.* **2013**, *2013* (20), 4215-4218. DOI. https://doi.org/10.1002/ejoc.201300643.

(4) Wiejak, S.; Masiukiewicz, E.; Rzeszotarsk, B. Improved scalable syntheses of mono- and bis-urethane derivatives of ornithine. *Chem. Pharm. Bull.* **2001**, *49* (9), 1189-1191. DOI. https://doi.org/10.1248/cpb.49.1189.

(5) Harms, H.; Klöckner, A.; Schrör, J.; Josten, M.; Kehraus, S.; Crüsemann, M.; Hanke, W.; Schneider, T.; Schäberle, T. F.; König, G. M. Antimicrobial dialkylresorcins from marine-derived microorganisms: insights into their mode of action and putative ecological relevance. *Planta Medica* **2018**, *84* (18), 1363-1371. DOI. https://doi.org/10.1055/a-0653-7451.

(6) Czekanska, M.; Akhir, A.; Arts, M.; Kany, A. M.; Saxena, D.; Maitra, R.; Dier, A.; Sokolenko, I.; Knopp, C. L.; Kinsinger, T. A new bactericidal thiourea antibiotic against drug-

resistant Staphylococcus aureus with *in vivo* activity and no detectable resistance. *Eur. J. Med. Chem.* **2026**, 119078. DOI. https://doi.org/10.1016/j.ejmech.2026.119078.

(7) Sati, H.; Carrara, E.; Savoldi, A.; Hansen, P.; Garlasco, J.; Campagnaro, E.; Boccia, S.; Castillo-Polo, J. A.; Magrini, E.; Garcia-Vello, P.; Wool, E.; Gigante, V.; Duffy, E.; Cassini, A.; Huttner, B.; Pardo, P. R.; Naghavi, M.; Mirzayev, F.; Zignol, M.; Cameron, A.; Tacconelli, E.; Aboderin, A.; Al Ghoribi, M.; Al-Salman, J.; Amir, A.; Apisarnthanarak, A.; Blaser, M.; El-Sharif, A.; Essack, S.; Harbarth, S.; Huang, X.; Kapoor, G.; Knight, G.; Muhwa, J. C.; Monnet, D. L.; Ousassa, T.; Sacsaquispe, R.; Severin, J.; Sugai, M.; Taneja, N.; Umubyeyi Nyaruhirira, A. The WHO Bacterial Priority Pathogens List 2024: a prioritisation study to guide research, development, and public health strategies against antimicrobial resistance. *Lancet Infect. Dis.* **2025**, *25* (9), 1033-1043. DOI. https://doi.org/10.1016/S1473-3099(25)00118-5.

(8) Barry, A. L.; Craig, W. A.; Nadler, H.; Reller, B. L.; Sanders, C. C.; Swenson, J. M. *Methods for determining bactericidal activity of antimicrobial agents: approved guideline*; National Committee for Clinical Laboratory Standards Wayne, PA, 1999. https://clsi.org/shop/standards/m26/.

(9) Pankey, G. A.; Sabath, L. Clinical relevance of bacteriostatic versus bactericidal mechanisms of action in the treatment of Gram-positive bacterial infections. *Clin. Infect. Dis.* **2004**, *38* (6), 864-870. DOI. https://doi.org/10.1086/381972.

(10) Crames, J. J. Biosyntheses of natural products from Proteobacteria. Ph.D. Dissertation, Philipps-Universität Marburg, Marburg, Germany, 2024.

(11) Masschelein, J.; Jenner, M.; Challis, G. L. Antibiotics from Gram-negative bacteria: a comprehensive overview and selected biosynthetic highlights. *Nat. Prod. Rep.* **2017**, *34* (7), 712-783. DOI. https://doi.org/10.1039/c7np00010c.

(12) Shimura, S.; Ishima, M.; Nakajima, S.; Fujii, T.; Himeno, N.; Ikeda, K.; Izaguirre-Carbonell, J.; Murata, H.; Takeuchi, T.; Kamisuki, S.; Suzuki, T.; Kuramochi, K.; Watashi, K.; Kobayashi, S.; Sugawara, F. Total Synthesis and Anti-Hepatitis C Virus Activity of MA026. *J. Am. Chem. Soc.* **2013**, *135* (50), 18949-18956. DOI. https://doi.org/10.1021/ja410145x.

(13) Wiedenhoeft, D.; Benoit, A. R.; Porter, J. D.; Wu, Y.; Virdi, R. S.; Shanaa, A.; Dockendorff, C. Design and synthesis of oxazoline-based scaffolds for hybrid Lewis acid/Lewis base catalysis of carbon–carbon bond formation. *Synthesis* **2016**, *48* (15), 2413-2422. DOI. https://doi.org/10.1055/s-0035-1560436.

(14) Marcovici-Mizrahi, D.; Gottlieb, H. E.; Marks, V.; Nudelman, A. On the Stabilization of the Syn-Rotamer of Amino Acid Carbamate Derivatives by Hydrogen Bonding. *J. Org. Chem.* **1996**, *61* (24), 8402-8406. DOI. https://doi.org/10.1021/jo961446u.

(15) Lin, H.; Long, J. Z.; Roche, A. M.; Svensson, K. J.; Dou, F. Y.; Chang, M. R.; Strutzenberg, T.; Ruiz, C.; Cameron, M. D.; Novick, S. J. Discovery of hydrolysis-resistant isoindoline *N*-acyl amino acid analogues that stimulate mitochondrial respiration. *J. Med. Chem.* **2018**, *61* (7), 3224-3230. DOI. https://doi.org/10.1021/acs.jmedchem.8b00029.

(16) Vizcaino, M. I.; Engel, P.; Trautman, E.; Crawford, J. M. Comparative Metabolomics and Structural Characterizations Illuminate Colibactin Pathway-Dependent Small Molecules. *J. Am. Chem. Soc.* **2014**, *136* (26), 9244-9247. DOI. https://doi.org/10.1021/ja503450q.

(17) Gołębiowski, A.; Jurczak, J.; Jacobsson, U. High pressure approach to the total synthesis of 6-epi-D-purpurosamine B. *Tetrahedron* **1987**, *43* (13), 3063-3066. DOI. https://doi.org/10.1016/S0040-4020(01)86847-2.

(18) Takacs, J. M.; Helle, M. A.; Seely, F. L. An improved procedure for the two carbon homologation of esters to α, β-unsaturated esters. *Tetrahedron Lett.* **1986**, *27* (11), 1257-1260. DOI. https://doi.org/10.1016/S0040-4039(00)84232-X.

(19) Hoye, T. R.; Kopel, L. C.; Ryba, T. D. In situ Generation and Nucleophilic Capture of 1, n-Dial Equivalents from 1, n-Dioates (α, ω-Diesters). *Synthesis* **2006**, *2006* (10), 1572-1574. DOI. https://doi.org/10.1055/s-2006-926465.

(20) Crimmins, M. T.; She, J. An improved procedure for asymmetric aldol additions with N-acyl oxazolidinones, oxazolidinethiones and thiazolidinethiones. *Synlett* **2004**, *2004* (08), 1371-1374. DOI. https://doi.org/10.1055/s-2004-825626.

(21) Evans, D. A.; Weber, A. E. Synthesis of the cyclic hexapeptide echinocandin D. New approaches to the asymmetric synthesis of β-hydroxy α-amino acids. *J. Am. Chem. Soc.* **1987**, *109* (23), 7151-7157. DOI. https://doi.org/10.1021/ja00257a041.

(22) Danda, H.; Hansen, M. M.; Heathcock, C. H. Acyclic stereoselection. 48. Reversal of stereochemistry in the aldol reactions of a chiral boron enolate. *J. Org. Chem.* **1990**, *55* (1), 173-181. DOI. https://doi.org/10.1021/jo00288a029.

(23) Lindel, T.; Hochgürtel, M.; Assmann, M.; Köck, M. Synthesis of the Marine Natural Product N α-(4-Bromopyrrolyl-2-carbonyl)-L-homoarginine, a Putative Biogenetic Precursor of the Pyrrole− Imidazole Alkaloids. *J. Nat. Prod.* **2000**, *63* (11), 1566-1569. DOI. https://doi.org/10.1021/np000160o.

(24) Evans, D. A.; Britton, T. C.; Ellman, J. A.; Dorow, R. L. The asymmetric synthesis of α-amino acids. Electrophilic azidation of chiral imide enolates, a practical approach to the synthesis of (*R*)- and (*S*)-α-azido carboxylic acids. *J. Am. Chem. Soc.* **1990**, *112* (10), 4011-4030. DOI. https://doi.org/10.1021/ja00166a045.

(25) Kinoshita, H.; Kotake, H. SELECTIVE CLEAVAGE OF N-t-BUTOXYCARBONYL PROTECTING GROUP. *Chem. Lett.* **1974**, *3* (6), 631-634. DOI. https://doi.org/10.1246/cl.1974.631.
